# Ruthenium Phenoxo Complexes: An Isolobal Ligand to Cp with Improved Properties

**DOI:** 10.1021/jacs.4c02088

**Published:** 2024-05-31

**Authors:** Tim Schulte, Zikuan Wang, Chen-Chen Li, Aboubakr Hamad, Felix Waldbach, Julius Pampel, Roland Petzold, Markus Leutzsch, Fritz Bahns, Tobias Ritter

**Affiliations:** † Max-Planck-Institut für Kohlenforschung, Kaiser-Wilhelm-Platz 1, Mülheim an der Ruhr 45470, Germany; ‡ Institute of Organic Chemistry, RWTH Aachen University, Landoltweg 1, 52074 Aachen, Germany

## Abstract

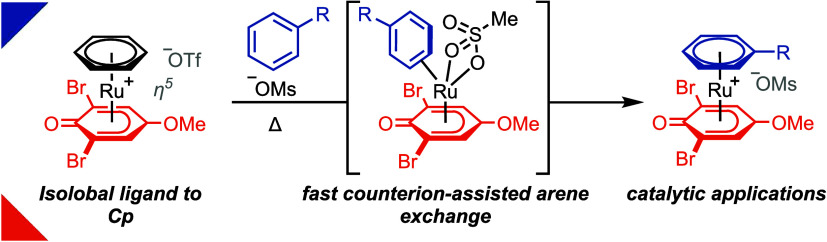

Catalytic π-arene activation is based on catalysts that allow for arene exchange. To date, cyclopentadiene (Cp)-derived catalysts are the most commonly used in π-arene activation despite their low arene exchange rates. Herein, we report the synthesis, analysis, and catalytic application of Ru(II) complexes supported by phenoxo ligands, which are isolobal alternatives to Cp. The phenoxo complexes exhibit arene exchange rates significantly faster than those of the corresponding Cp complexes. The rate can be further increased through the choice of appropriate counterions. The mechanism of the arene exchange process is elucidated by kinetic and computational analyses. We demonstrate the utility of the new catalysts through an S_N_Ar reaction between fluorobenzene and alcohols, including secondary alcohols that could not be used previously in related reactions. Moreover, the catalytic thermal decarboxylation of phenylacetic acids is presented.

## Introduction

**Scheme 1:**
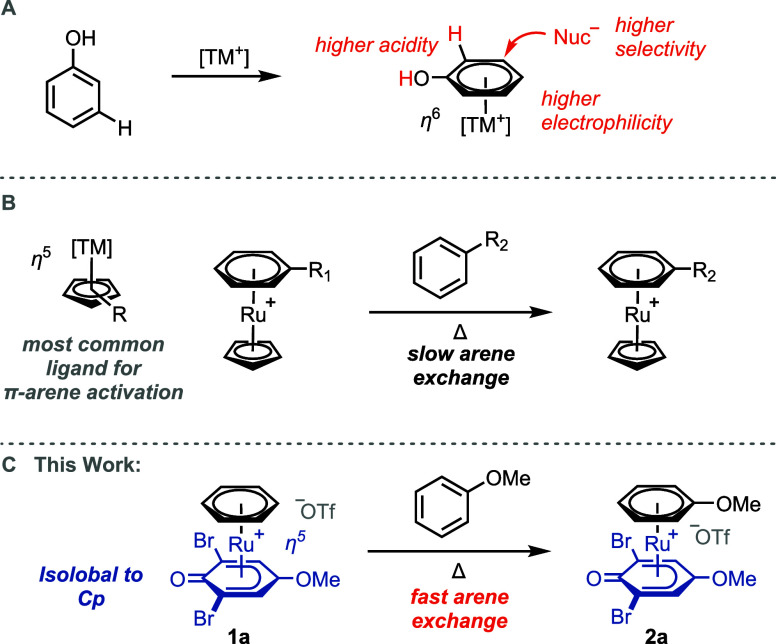
(A) Concept; (B) π-Arene Activation with RuCp Complexes; and (C) π-Arene Activation Using Ru Phenoxo Complexes

Arenes, when π-coordinated to an electron-withdrawing transition metal center, possess enhanced electrophilicity (Scheme 1A).^1^ To harness such reactivity in a catalytic reaction, transition metal catalysts must engage in arene exchange to liberate the product and coordinate another arene substrate.^2,3^ Most reported transformations via π-arene activation are based on Cp- or Cp*-ligated complexes.^4^ Cp-based Ru complexes exhibit slow ligand exchange rates (Scheme 1B),^5,6^ which can lead to reaction temperatures up to 180 °C and reaction times up to 14 days.^7^ Improvement of catalyst properties in π-arene activation is limited by the dearth of available ligands beyond Cp, and the synthetically challenging derivatization of cyclopentadiene provides only a few options for catalyst optimization. Here, we report ruthenium-based catalysts supported by phenoxo ancillary ligands as isolobal alternatives to Cp (Scheme 1C). The large number of readily available phenols facilitated the identification of catalysts that exhibit arene exchange rates several orders of magnitude faster than those of Cp-based ruthenium complexes while retaining the electrophilic activation of the arene substrates. Furthermore, we show that the counterion of the catalyst significantly influences the arene exchange rates and provide detailed mechanistic insights into the arene exchange process.

The activation of arenes for nucleophilic functionalization by π-coordination to a transition metal has been employed for more than 50 years.^2^ In early reports from the 1970s and 1980s, mainly first-row transition metal complexes, such as [(arene)_2_Cr]^+^, [(arene)Cr(CO)_3_]^+^,^8^ and [(arene)Mn(CO)_3_]^+^^9^ and later [(arene)FeCp]^+^^10^ were used to increase the electrophilicity of arenes. More recent protocols include complexes with other metals, such as [(arene)RuCp]^+^,^4^ [(arene)RhCp*]^2+^,^11^ and [(arene)IrCp*]^2+^.^12^ These complexes led to the development of several useful transformations, for example, trifluoromethylation,^13^ Csp^2^-Csp^3^-coupling,^14^ C–H-hydroxylation,^12^ or ^18^F-deoxyfluorination.^15^ Mechanistic analysis of the arene exchange process was first reported in the 1960s by Strohmeier^16^ and later by others, including Sievert,^17^ Traylor,^18^ and Kündig^19^ for (arene)Cr(CO)_3_ complexes. The first example of a catalytic functionalization by π-arene activation was reported in 1984 by Houghton and Voyle.^20^

Since then, multiple examples for catalytic π-arene activation have been published, in which predominantly Cp-based ligands were used, too. Examples include the amination,^7^ fluorination,^21^ and hexafluoroisopropoxylation^22^ of aryl chlorides, hydroxylation of aryl fluorides,^23^ amination of phenols,^24^ and benzylic deuteration.^25^ The slow arene exchange rates of the Cp/Cp* catalysts used in these reactions require reaction temperatures often above 120 °C, which limits the substrate scope and utility. Transition metal complexes containing other ligands than Cp-derivatives that exhibit faster arene exchange rates have been reported^26^ but are less electrophilic and therefore normally unsuitable for catalytic applications that require an electrophilic arene. The development of ligands that induce a similar activation of the coordinated arene as Cp-derivatives, while inducing a faster arene exchange rate, is thus highly desirable.

## Results and Discussion

**Scheme 2:**
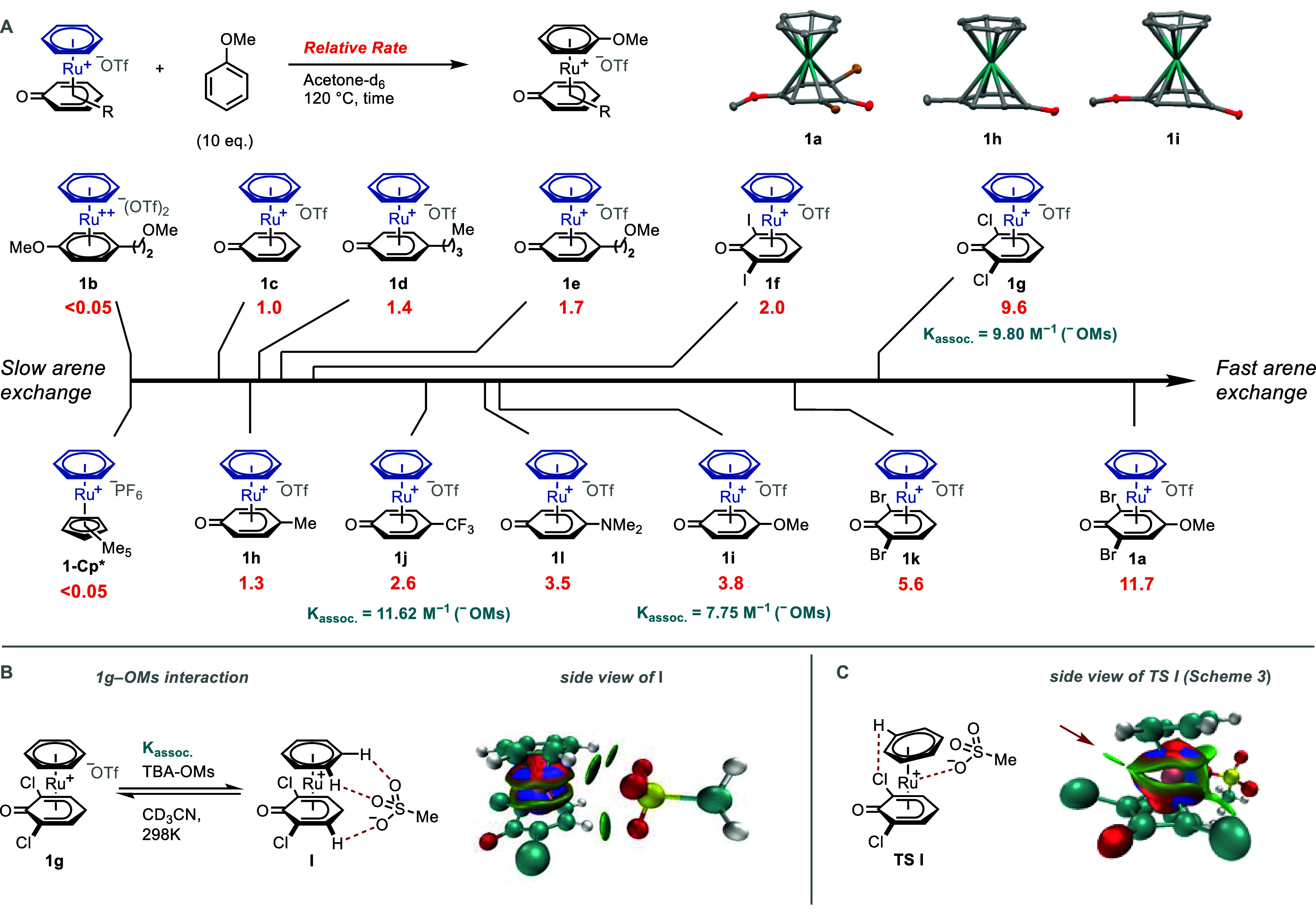
(A) Relative Arene Exchange Rates in Orange for Selected Ru Phenoxo Complexes; (B) Independent Gradient Model Based on Hirshfeld Partition (IGMH) Plot (Isovalue = 0.01) of the Noncovalent Complex between **1g** and ^–^OMs; and (C) IGMH Plot (Isovalue = 0.01) of TS I^,^ For full scope, see Table S1; complexes **1a**–**1l** with ^–^OTf counterion (Figures S1–S37); complex 1-Cp* with ^–^PF_6_ and ^–^OMs counterions (Figures S38 and S39); crystal structures of **1a**, **1h**, and **1i**; thermal ellipsoids are drawn at the 50% probability level; hydrogen atoms and counterion omitted for clarity; association constants (*K*_assoc._) in turquoise determined by ^1^H NMR titration with TBA-OMs (Figures S58–S60). Interactions shown as red dashed lines.

**Scheme 3:**
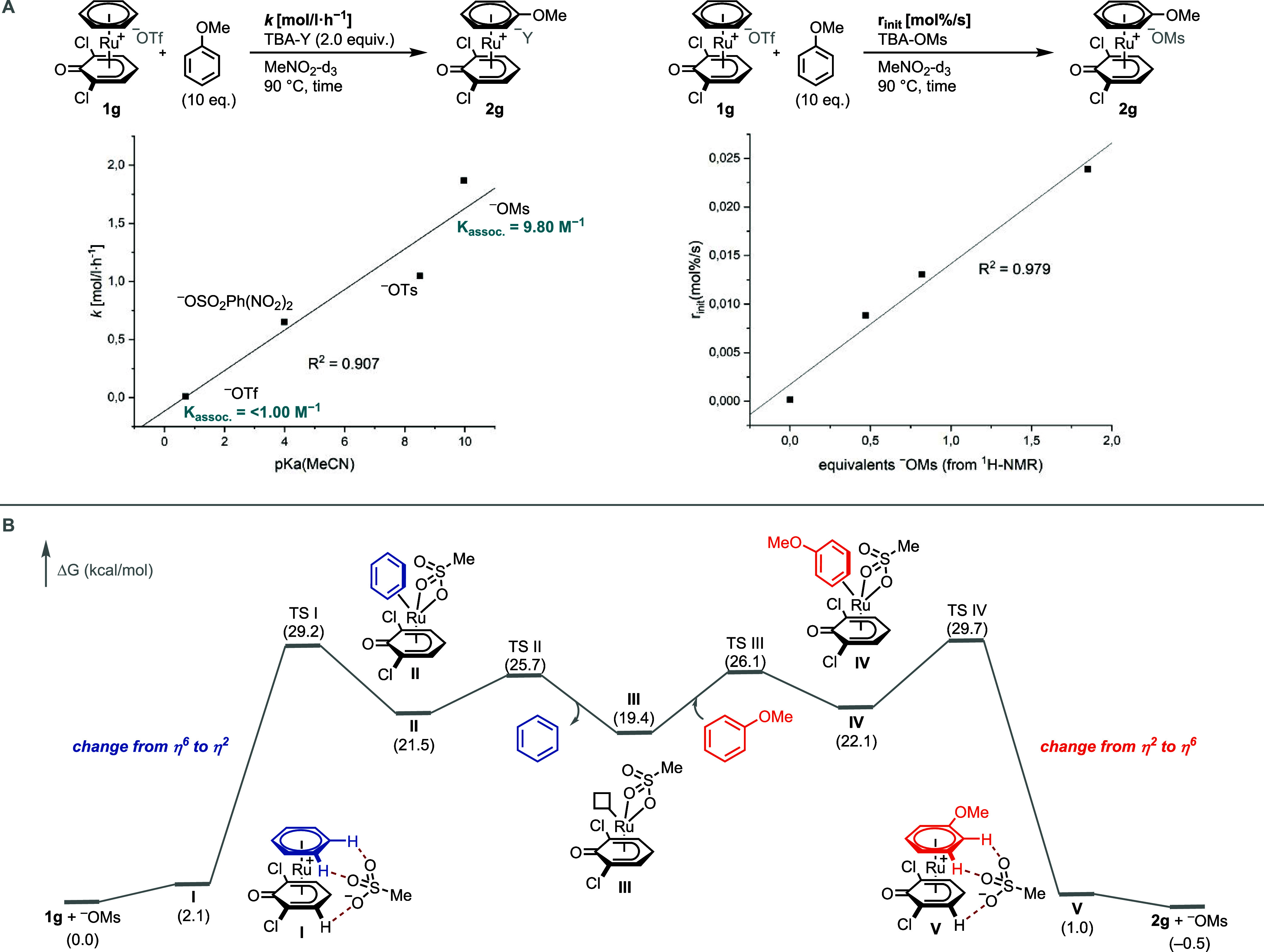
(A) Left: Counterion Influence on Arene Exchange and Correlation between Arene Exchange Rate and pKa (MeCN);^34,35^ (A) Right: Arene Exchange Rates with Different Equivalents of TBA-OMs; and (B) Free Energy Profileof the Arene Exchange Reaction between **1g** and Anisole to Form **2g**, Calculated at the DLPNO–CCSD(T1)//PBE0-D3 Level of Theory in MeNO_2_ Solution Using ORCA Association constants (*K*_assoc._) of **1g** with different counterions in turquoise determined by ^1^H NMR titration (Figures S60 and S61).

Based on the large number of available and well-defined ruthenium precatalysts^27^ and our previous experience on π-arene activation,^11,12^ we were motivated to develop a catalyst with a ruthenium metal center. The commonly used cyclopentadienyl-based ligands are facial ligands that occupy three coordination sites on the metal. Similar bonding is found for simple *η*^6^ arene ligands; therefore, a similar complex is obtained using an arene ligand as opposed to Cp.^28^ We proposed that a Ru(II) diarene complex could selectively exchange one of the two arene ligands if the other arene ligand exhibits a larger *trans* influence due to electron-donating substituents. The dicationic complex **1b** (Scheme 2A) showed slow arene exchange rates. However, by using phenol as the ligand, which can be deprotonated to obtain a monocationic *η*^5^ phenoxo complex, we observed that such Ru(II) complexes exchanged the arene significantly faster than the dicationic equivalents (Scheme 2A, compare **1b** and **1e**). A variety of different Ru(II) phenoxo complexes were synthesized,^29^ and the rate of exchange of coordinated benzene to anisole was monitored by ^1^H NMR spectroscopy (Scheme 2A). Although the mesylate counterion accelerates arene exchange (vide infra), we have compared the rates of the ruthenium complexes with ^–^OTf counterions because they are synthetically more readily accessible. No arene exchange was observed for 1-Cp*. The arene exchange rate of the phenoxo complexes can be significantly influenced by changing the electronic properties of the phenoxo ligand. A −OMe and −CF_3_ group in the 4-position of the phenoxo ligand resulted in a 3–4-fold arene exchange rate increase compared to that of the unsubstituted phenol (**1i** and **1j**). ORCA-^30^ based DFT calculations suggest that the rate acceleration of electron-donating substituents, such as in **1i** or **1l**, is a result of a noncovalent interaction between the substituent and the counterion, rather than a change of the *trans* influence of the ligand (Figures S114 and S115). We propose that the rate acceleration of more electron-deficient phenoxo ligands results partially from a stronger interaction between the cationic Ru complex and its counterion. Indeed, CF_3_-substituted complex **1j** showed a higher association constant (*K*_assoc._) with the counterion (methanesulfonate, ^–^OMs) than −OMe-substituted complex **1i** (Scheme 2A). DFT calculations of **1g** suggest that one of the optimum binding configurations with ^–^OMs (Δ*G*_bind_ = +2.1 kcal/mol) involves three C–H···O interactions between both arene ligands of **1g** and all three oxygen atoms of ^–^OMs (Scheme 2B). An independent gradient model based on Hirshfeld partition (IGMH) plot^31,32^ generated by Multiwfn^33^ shows green isosurfaces between three arene C–H bonds and the oxygen atoms (Scheme 2B), which indicates the attractive C–H···O interactions that are mainly dispersive in character. A similar conformer in which ^–^OMs forms one C–H···O interaction with the benzene ligand and two with the phenoxo ligand was also found, with very similar binding free energies (Figure S119). The noncovalent binding of ^–^OMs favors the subsequent coordination of ^–^OMs to the ruthenium center, which accelerates the arene exchange. Too much steric bulk or substituents in the 3- and 5-positions of the phenol did not result in increased rates for the arene replacement (see **S-2**, **S-3**, **S-6**). Methyl substituents in the 2- and 6-positions of the phenol decelerated the arene exchange rates significantly, while −OMe substituents resulted in ligand exchange rates similar to those of the unsubstituted phenol (see **S-4**, **S-5**). When the *ortho* positions were decorated with two halides, a significant acceleration of the ligand exchange was observed. Computational IGMH analysis of the arene exchange process of **1g** (Scheme 2C) shows an attractive interaction between one of the chlorine atoms and one of the benzene hydrogen atoms in the transition state of the rate-determining step during arene exchange (TS I, see Scheme 3B for full reaction profile), as evidenced from the green patch of the isosurface (red arrow). The interaction is not present in the noncovalent complex **I**, nor when the chlorine substituents are replaced by hydrogen atoms (Figure S112). We thus propose that the interaction is one reason for the acceleration of the arene exchange rate induced by the 2,6-dihalo substitution. As only one C–H···Cl interaction can be present during the transition state for **1g** at a time, 2-chlorophenoxo complex **SI-9** was synthesized to further analyze the influence of the *ortho*-halide substitution. Also, **SI-9** shows significantly faster arene exchange rates than **1c**, which is consistent with the accelerating effect of the *ortho*-halide substitution arising from the aforementioned C–H···Cl interaction during the transition state (TS I). The slower exchange rate of **SI-9** compared to **1g** can be explained by the higher electron-withdrawing nature of the 2,6-dichlorophenoxo over the 2-chlorophenoxo ligand. Electron-deficient ligands accelerate arene exchange by stabilizing intermediate **II** during the arene exchange process, as explained further down. For complexes with other substituents than halides in the 2- and 6-positions, such as **1c** or 2,6-dimethoxy substituted complex **SI-5**, no or very little interaction with the benzene hydrogen atoms was found (Figure S113), which is consistent with their slower arene exchange rates. Two chlorine or bromine substituents in the 2- and 6-positions of the phenol induced a 10- and 6-fold increase in arene exchange rate, respectively, compared to the unsubstituted phenol, while two iodine substituents only lead to a 2-fold increase in rate. The lower acceleration effect of iodine substituents might be a result of the higher steric hindrance of the iodide compared to chloride or bromide. The combination of two bromine substituents in the 2- and 6-positions with a methoxy group in the 4-position led to complex **1a**, which showed the fastest rate out of the tested compounds with more than an order of magnitude faster exchange than the unsubstituted phenol. The association constant of **1g** is lower than that for **1j** (Scheme 2A), which is consistent with 2,6-dihalo-substituted ligands inducing a faster arene exchange rate due to interactions with the benzene hydrogen atoms during the arene exchange and not due to a stronger complex-counterion interaction.

Generally, arene exchange is believed to follow a stepwise mechanism where the departing arene changes from *η*^6^ to *η*^4^ to *η*^2^ coordination followed by dissociation of the arene.^3^ The nascent coordination sites are occupied by incoming ligands such as solvent molecules. The change from *η*^6^ to *η*^4^ coordination is proposed to be the most endothermic and therefore the rate-determining step of the exchange process.^3^ However, a mechanistic analysis of how the *η*^6^ to *η*^4^ switch proceeds is missing in the literature. Starting from saturated complexes, an associative or associative interchange pathway, in which a 20 electron complex would be initially formed, is unlikely, as a highly energetic antibonding orbital would need to be filled.^36^ However, multiple examples have been reported, which demonstrate that the presence of hemilabile groups^37,38^ or nucleophilic arene exchange catalysts^39^ significantly accelerates arene exchange rates, which is inconsistent with a dissociative ligand exchange. The actual mechanism for the dissociation of the arene during the arene exchange process for the reported complexes thus remains unclear.

In the case of the phenoxo ligands, the presence or absence of a hemilabile group resulted in similar rates of arene exchange (Scheme 2A, **1d** and **1e**). However, we noticed that the rate can be significantly accelerated by the choice of appropriate counterion (Scheme 3A). The fastest arene exchange was obtained with methanesulfonate (^−^OMs). Some counterions, e.g., ^–^OAc, resulted in irreversible coordination to ruthenium. Counterions that were weaker coordinating than ^–^OMs, such as ^–^PF_6_ (<0.01 mol/L·h^–1^), ^–^BF_4_ (<0.01 mol/L·h^–1^), or ^–^OTf (<0.01 mol/L·h^–1^), resulted in slower arene exchange (Figures S40–S43). The stronger interaction between **1g** and ^–^OMs compared to ^–^OTf is demonstrated by the different association constants between the ruthenium cation and anion (Scheme 3A, left). The p*K*_a_ value of a given acid can be used to make conclusions about the coordination ability of the corresponding anion.^40^ A linear correlation is obtained between arene exchange rate constant of **1g** and the p*K*_a_ of the conjugate acids of different counterions in MeCN. To exclude that the rate acceleration by addition of different counterions results solely from medium effects, such as changes in the dielectric constant of the solvent, ^1^H NMR experiments with excess of TBA-OTf revealed only insignificant changes in rate (Figures S62–S64). Kinetic measurements determined the exchange reaction of **1g** with anisole in the presence of ^–^OMs to be order 0.7 in counterion (Table S3 and Figure S50). An order in counterion >0 excludes a purely dissociative pathway. Further analysis of the exchange process of **1g** by DFT calculations suggests that the departing arene does not change from *η*^6^ to *η*^4^ coordination (Scheme 3B). Instead, the noncovalent complex between **1g** and ^–^OMs (**I**) reacts to form complex **II** in which the arene is *η*^2^ coordinated and the ^–^OMs anion *κ*^2^ bound, in a concerted process with a single barrier. By comparison, the analogous reaction with the ^–^OTf anion proceeds through a higher free energy barrier (33.7 kcal/mol), which highlights the role of the ^–^OMs anion as a stronger external ligand than ^–^OTf that stabilizes the *η*^2^-benzene complex **II** (Figure S111). The coordination of the ^–^OMs anion makes the phenoxo ligand more electron-rich in TS I (sum of Hirshfeld I charges: −0.312) than in **I** (−0.288), suggesting that electron-withdrawing substituents should accelerate the reaction; this is in accord with the better performance of the CF_3_-substituted complex **1j** compared to the unsubstituted complex **1c**, and may also contribute to the good performance of halogen-substituted complexes (in addition to the aforementioned C–H···X interactions). As mentioned before, the enhanced reaction rates of the electron-rich complexes **1i** and **1l** are instead explained by C–H···O interactions between the ^–^OMs anion and the substituents (Figures S114–S115; Tables S38 and S39). From complex **II**, arene ligand dissociates to form complex **III**, which possesses a vacant coordination site that can be occupied by a solvent molecule (Figure S90). From complex **III**, the incoming arene coordinates with the vacant site, generating the product **2g** following an analogous mechanism in reversed order. An arene exchange mechanism proceeding through intermediates in which the phenoxo ligand is coordinated with lower hapticity (*η*^1^ or *η*^3^-coordination) was found to be unlikely (Figures S135–S137). The experimental and computational data therefore suggest that the arene exchange of the phenoxo complexes proceeds by a change of *η*^6^ to *η*^2^ coordination, while the nascent coordination sites on the metal are occupied by the counterion within the same elementary step.

**Scheme 4:**
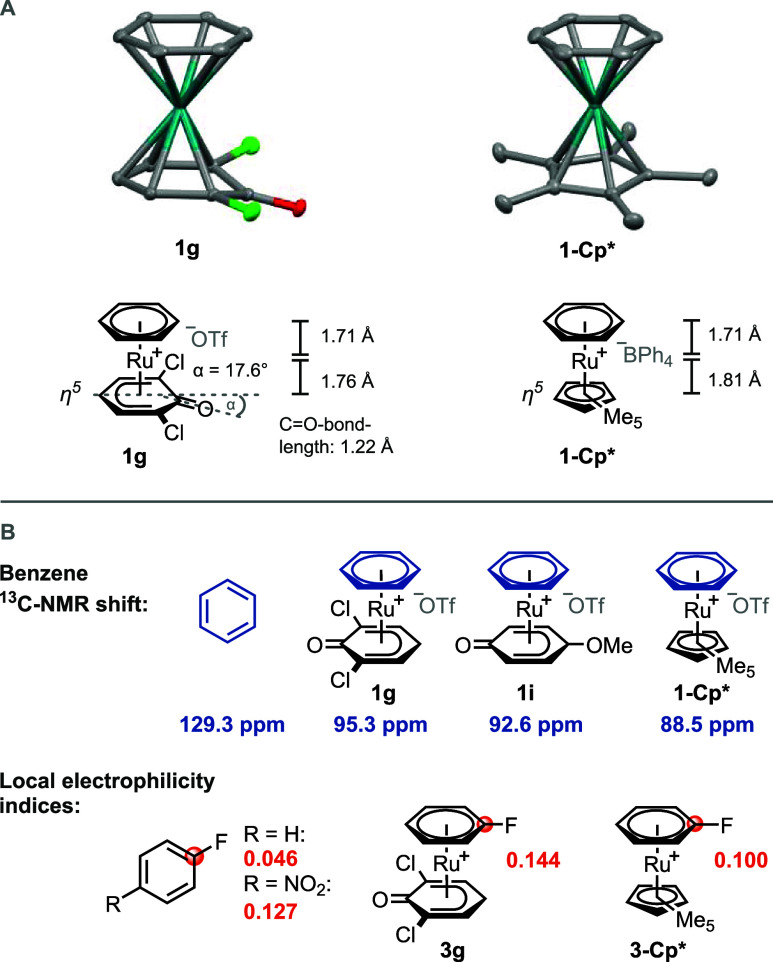
(A) Crystal Structures of **1g** and 1-Cp* and Structural Comparison, Thermal Ellipsoids Drawn at the 50% Probability Level, Hydrogen Atoms and Counterion Omitted for Clarity; (B) ^13^C NMR Resonances of Benzene and Benzene Complexes **1f**, **1h**, and 1-Cp* and Local Electrophilicity Indices Calculated at the PBE0/def2-SVP Level in the Gas Phase Using ORCA

The structural similarity between the Ru complexes with phenoxo ligands and Cp/Cp* was elucidated by single-crystal structure analysis. As presented in Scheme 4A, Cp* and the phenoxo ligand are *η*^5^ coordinated. The C=O bond length is 1.22 Å, which is similar to 1.23 Å for common carbonyl compounds. The Ru–C-distance of the carbonyl carbon is larger (2.55 Å) than the Ru–C-distance of the other carbon atoms of the phenoxo ligand (2.1–2.3 Å). Moreover, the C=O-group is bent out of the ligand plane by 17.6°, which further supports the *η*^5^ coordination and is in agreement with previously reported ruthenium phenoxo complexes.^41^ The distance between ruthenium and the benzene plane is 1.71 Å and is equal in phenoxo and Cp* complexes, which is consistent with similar metal-π-interaction in both complexes. The distance between ruthenium and the Cp* plane is 1.81 Å and slightly longer than the 1.76 Å in the case of the phenoxo complex. The computed noncovalent interactions of **1g** with the counterion in solution (as in **I**, Scheme 2) were not observed in the solid-state crystal structure, presumably due to dominating crystal packing forces. To examine the electrophilic activation of the coordinated arene, the ^13^C NMR chemical shifts of the benzene signals were compared. As shown in Scheme 4B, the ^13^C NMR resonance of benzene is significantly changed when it is coordinated to the ruthenium phenoxo or Cp* complexes. Both complexes induce a similar change of the chemical shift of the ^13^C NMR benzene signal, which indicates a similar electrophilicity increase. To compare the electrophilicity of coordinated fluorobenzene, a potential substrate for S_N_Ar reactions, the condensed local electrophilicity indices were calculated via DFT calculations. Here, the phenoxo ligand induces a higher removal of electron density from the *ipso* carbon of the C–F-bond, compared to the Cp* ligand. The weaker electron-donating ability of the phenoxo ligands compared to Cp* results in more electrophilic arenes but also facilitates ^–^OMs anion coordination to the Ru center, which is one of the reasons why phenoxo complexes exhibit higher arene exchange rates compared to the Cp* complex.

**Scheme 5:**
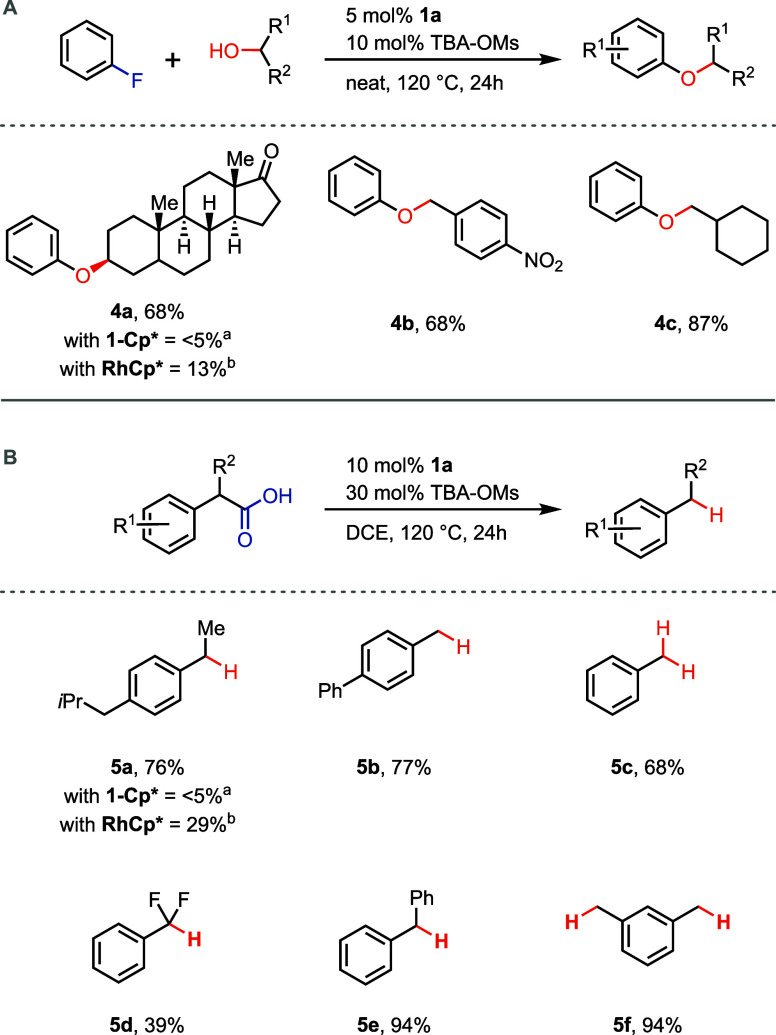
(A) Scope for the S_N_Ar Reaction between Fluorobenzene and Alcohols Catalyzed by Complex **1a**; (B) Scope for the Protodecarboxylation of Phenylacetic Acids Catalyzed by Complex **1a**; ^a^1-Cp* Used as a Catalyst Instead of **1a**; ^b^[(Anisole)Rh^III^Cp*](OTf)_2_ Used as a Catalyst Instead of **1a**

After **1a** was identified as the phenoxo complex exhibiting the fastest arene exchange rates among the tested complexes, its suitability to catalyze π-arene-activated reactions was analyzed. The S_N_Ar reaction between aryl fluorides and primary alcohols has been achieved previously using a Rh(III) Cp* complex.^23^ We analyzed whether complex **1a** could serve as a catalyst for the alkoxylation of fluoroarenes using secondary alcohols as nucleophiles (Scheme 5A). Yields up to 87% were observed using primary alcohols as nucleophiles in the absence of an exogenous base in neat fluorobenzene (∼20 equiv of fluorobenzene corresponding to the nucleophile). Secondary alcohols have not been reported previously as substrates for the S_N_Ar reaction with aryl fluorides but could be functionalized using catalyst **1a**. The previously reported Rh(III) Cp* complex gave significantly lower yields for secondary alcohols, while with 1-Cp*, no product formation was observed within the error of measurement in the example of **4a**. Furthermore, benzylic alcohol reacted with fluorobenzene with **1a** as a catalyst to form an alkyl–aryl-ether that can serve as a precursor to phenols. Concerning the mechanism, we propose that the reaction proceeds, similarly to reported arene exchange-based reactions,^23,42,43^ via π-coordination of the fluoroarene, which enhances the electrophilicity and enables the S_N_Ar reaction with alcohols (Figure S67). The key intermediates of the catalytic cycle, namely, the fluoroarene complex (intermediate **I** in Figure S67) and alkyl-aryl-ether complex (intermediate **II** in Figure S67) have been individually detected by ^1^H NMR spectroscopy and HRMS (Figures S68–S76). A mechanism in which the aryl fluoride undergoes oxidative addition to the ruthenium was ruled out by the stoichiometric reaction of fluorobenzene complex **3g** with an alcohol (Figure S76). We hypothesize that the superior performance of phenoxo catalyst **1a** compared to 1-Cp* or the Rh(III) Cp* complex is a result of the faster arene exchange rates and enhanced electrophilicity of **1a**.

Catalyst **1a** is also competent to catalyze the thermal decarboxylation of phenylacetic acids (Scheme 5B). The majority of reported decarboxylation methods is based on photochemical activation,^44,45^ while thermal protocols are scarce and typically require temperatures above 200 °C.^46,47^ Using **1a** in the presence of TBA-OMs, different phenylacetic acids smoothly underwent protodecarboxylation at 120 °C. Again, lower yields were observed with the Rh(III) Cp* catalyst, while 1-Cp* failed to deliver any product in the example of **5a**. Although carbonyl groups are generally electrophilic and prone to nucleophilic attack, no decomposition products of the phenoxo complexes were observed that would result from a nucleophilic attack on the carbonyl group of the phenoxo ligand. Mechanistically, we propose that the phenylacetic acid is first π-coordinated to the catalyst, which increases its acidity and enables thermal decarboxylation in a two-electron process (Figure S79). The resulting negative charge on the arene is stabilized by Ru(II) to give a neutral intermediate (intermediate **II** in Figure S79). We propose that the stabilization of the negative charge by the ruthenium catalyst is the major catalyst feature that enables thermal protodecarboxylation. Protonation and subsequent arene exchange complete the catalytic cycle and deliver the protodecarboxylated product. Control experiments with common radical traps, in which no reactivity was observed, are consistent with the absence of a radical mechanism (Table S6). When deuterated solvent is employed, decarboxylative deuteration and C–H-deuteration are observed (Figures S80 and S81). Benzylic C–H-deuteration has been previously described to proceed by a neutral intermediate similar to **II** in Figure S79.^25^ The observation of benzylic C–H-deuteration thus shows that the phenoxo complexes are capable of stabilizing the negatively charged arene after decarboxylation. The superior performance of **1a** compared to 1-Cp* or the Rh(III) Cp* complex as a catalyst in the protodecarboxylation might be a result of the faster arene exchange rates exhibited by **1a** in combination with its ability to stabilize the negatively charged arene after decarboxylation.

## Conclusions

In summary, phenoxo ligands have been described as an isolobal alternative to Cp-based ligands in π-arene activation. The high modularity of phenols as ligands has been used to improve the arene exchange properties of arene-Ru(II)-phenoxo complexes significantly compared with the corresponding Cp-based complex. Additionally, it was shown that the phenoxo ligands induce a similar activation on the coordinated arene as that of Cp/Cp*. Furthermore, it was shown that the counterion serves as a cocatalyst to facilitate arene replacement, and it was demonstrated that the addition of ^–^OMs results in acceleration of the arene exchange. The improved catalyst properties have subsequently been used to achieve the S_N_Ar reaction between aryl fluorides and alcohols. Here, secondary alcohols could be employed as substrates for the first time in such a reaction chemistry. Moreover, the thermal decarboxylation of phenylacetic acids was demonstrated using **1a**. Ruthenium phenoxo complexes have en utilized as catalysts for the amination of phenols.^48^ The simple synthetic access to the phenoxo complexes could enable the exploration of their properties in other areas of catalysis beyond π-arene activation, where typically, Cp-based catalysts are used.

## Supporting Information Available

Experimental and computational procedures, spectroscopic data, and NMR spectra of all products (PDF)
