# Supplementary material for: Ruthenium Phenoxo Complexes: An Isolobal Ligand to Cp with Improved Properties
Source: J Am Chem Soc. 2024 May 31;146(23):15825–32. doi: 10.1021/jacs.4c02088 (PMC11177254; doi:10.1021/jacs.4c02088)
Supplement: Supplementary file 1 — ja4c02088_si_001.pdf [file ja4c02088_si_001.pdf]

SUPPORTING INFORMATION

Ruthenium Phenoxo Complexes: An Isolobal Ligand to Cp with Improved Properties

Tim Schulte,[1,2] Zikuan Wang,[1] Chen-Chen Li,[1] Aboubakr Hamad,[1,2] Felix Waldbach,[1] Julius Pampel,[1] Roland Petzold,[1] Markus Leutzsch,[1] Fritz Bahns,[1,2] Tobias Ritter*[1,2]

[1]Max-Planck-Institut für Kohlenforschung, Kaiser-Wilhelm-Platz 1, 45470 Mülheim an der Ruhr, Germany.

[2]Institute of Organic Chemistry, RWTH Aachen University, Landoltweg 1, 52074 Aachen, Germany.

*E-mail: ritter@kofo.mpg.de

TABLE OF CONTENTS

TABLE OF CONTENTS 2

MATERIALS AND METHODS 7

EXPERIMENTAL DATA 9

Complex Synthesis 9

[η^6-Benzene-η^5-(2,6-dibromo-4-methoxy-1-phenoxo)Ru](OTf) (**1a**) 9
[η^6-Benzene-η^6-(4-(2-methoxyethyl)anisole)Ru](OTf)$_2$ (**1b**) 11
[η^6-Benzene-η^5-(phenoxo)Ru](OTf) (**1c**) 12
[η^6-Benzene-η^5-(4-butyl-1-phenoxo)Ru](OTf) (**1d**) 13
[η^6-Benzene-η^5-(4-(2-methoxyethyl)-1-phenoxo)Ru](OTf) (**1e**) 14
[η^6-Benzene-η^5-(2,6-diiodo-1-phenoxo)Ru](OTf) (**1f**) 15
[η^6-Benzene-η^5-(2,6-dichloro-1-phenoxo)Ru](OTf) (**1g**) 16
[η^6-Benzene-η^5-(4-methyl-1-phenoxo)Ru](OTf) (**1h**) 17
[η^6-Benzene-η^5-(4-methoxy-1-phenoxo)Ru](OTf) (**1i**) 18
[η^6-Benzene-η^5-(4-trifluoromethyl-1-phenoxo)Ru](OTf) (**1j**) 19
[η^6-Benzene-η^5-(2,6-dibromo-1-phenoxo)Ru](OTf) (**1k**) 20
[η^6-Benzene-η^5-(4-(N,N-dimethylamino)-1-phenoxo)Ru](OTf) (**1l**) 21
[η^6-Benzene-η^5-(1-phenoxo-4-phenyl)Ru](OTf) (**SI-1**) 22
[η^6-Benzene-η^5-(3,5-dimethoxy-1-phenoxo)Ru](OTf) (**SI-2**) 23
[η^6-Benzene-η^5-(1-phenoxo-3,4,5-trimethyl)Ru](OTf) (**SI-3**) 24
[η^6-Benzene-η^5-(1-phenoxo-2,4,6-trimethyl)Ru](OTf) (**SI-4**) 25
[η^6-Benzene-η^5-(2,6-dimethoxy-1-phenoxo)Ru](OTf) (**SI-5**) 26
[η^6-Benzene-η^5-(4-*t*Bu-1-phenoxo)Ru](OTf) (**SI-6**) 27
[η^6-Benzene-η^5-(4-amino-1-phenoxo)Ru](OTf) (**SI-7**) 28
[η^6-Ethylbenzoate-η^5-(2,6-dichloro-1-phenoxo)Ru](OTf) (**SI-8**) 30
[η^6-Benzene-η^5-(2-chloro-1-phenoxo)Ru](OTf) (**SI-9**) 31

Arene Exchange Study 32

Arene exchange study for [η^6-benzene-η^5-(2,4,6-trimethyl-1-phenoxo)Ru](OTf) (**SI-4**) 33
Arene exchange study for [η^6-benzene-η^5-(2,6-dimethoxy-1-phenoxo)Ru](OTf) (**SI-5**) 34
Arene exchange study for [η^6-benzene-η^5-(3,4,5-trimethyl-1-phenoxo)Ru](OTf) (**SI-3**) 35
Arene exchange study for [η^6-benzene-η^5-(4-*t*Bu-1-phenoxo)Ru](OTf) (**SI-6**) 36
Arene exchange study for [η^6-benzene-η^5-(phenoxo)Ru](OTf) (**1c**) 37
Arene exchange study for [η^6-benzene-η^5-(3,5-dimethoxy-1-phenoxo)Ru](OTf) (**SI-2**) 38
Arene exchange study for [η^6-benzene-η^5-(4-methyl-1-phenoxo)Ru](OTf) (**1h**) 39
Arene exchange study for [η^6-benzene-η^5-(4-*n*-butyl-1-phenoxo)Ru](OTf) (**1d**) 40
Arene exchange study for [η^6-benzene-η^5-(4-(2-methoxyethyl)-1-phenoxo)Ru](OTf) (**1e**) 41
Arene exchange study for [η^6-benzene-η^5-(2,6-diiodo-1-phenoxo)Ru](OTf) (**1f**) 42
Arene exchange study for [η^6-benzene-η^5-(4-trifluoromethyl-1-phenoxo)Ru](OTf) (**1j**) 43

Arene exchange study for [η^6-benzene-η^5-(4-amino-1-phenoxo)Ru](OTf) (**SI-7**) 44
Arene exchange study for [η^6-benzene-η^5-(4-*N*,*N*-dimethylamino-1-phenoxo)Ru](OTf) (**1l**) ... 45
Arene exchange study for [η^6-benzene-η^5-(4-methoxy-1-phenoxo)Ru](OTf) (**1i**) 47
Arene exchange study for [η^6-benzene-η^5-(2-chloro-1-phenoxo)Ru](OTf) (**SI-9**) 48
Arene exchange study for [η^6-benzene-η^5-(2,6-dibromo-1-phenoxo)Ru](OTf) (**1k**) 49
Arene exchange study for [η^6-benzene-η^5-(2,6-dichloro-1-phenoxo)Ru](OTf) (**1g**) 50
Arene exchange study for [η^6-benzene-η^5-(2,6-dibromo-4-methoxy-1-phenoxo)Ru](OTf) (**1a**) 51
Arene exchange study for [η^6-benzene-η^6-(4-methoxymethylanisole)Ru](OTf) (**1b**) 52
Arene exchange study for [η^6-benzene-η^6-(pentamethylcyclopentadienyl)Ru](OTf) (**1-Cp***) ... 53
Arene exchange study for [η^6-benzene-η^6-(pentamethylcyclopentadienyl)Ru](OTf) (**1-Cp***) in presence of TBA-OMs 54

Counterion Effect Study 55

Counterion study for $^{-}PF_6$ 56
Counterion study for $^{-}BF_4$ 56
Counterion study for $^{-}$OTf 57
Counterion study for $^{-}OSO_2Ph(NO_2)_2$ 57
Counterion study for $^{-}$OTs 58
Counterion study for $^{-}$OMs 58
Counterion study for $^{-}$OMs in presence of protic additives 59

Kinetics for Different Concentrations of $^{-}$OMs 61

Association Constants (K_a) 65

Association constant (K_a) of **1j** with $^{-}$OMs 65
Association constant (K_a) of **1i** with $^{-}$OMs 66
Association constant (K_a) of **1g** with $^{-}$OMs 68
Association constant (K_a) of **1g** with $^{-}$OTf 69

Medium Effect Experiment 71

Arylation of Alcohols with Aryl Fluorides Catalyzed by **1a** 73

Phenyl-epiandrosterone ester (**4a**) 73
Phenyl-epiandrosterone ester (**4a**) with 1-Cp* as Catalyst 73
Phenyl-epiandrosterone ester (**4a**) with RhCp* as Catalyst 74
1-Nitro-4-(phenoxymethyl)benzene (**4b**) 75
(Cyclohexylmethoxy)benzene (**4c**) 76
Mechanistic Investigation and Optimization 77

Protodecarboxylation of Phenylacetic Acids Catalyzed by **1a** 86

Decarboxylation of Ibuprofen (**6a**) (1.0 mmol scale) 86
Decarboxylation of Ibuprofen (**6a**) (1.0 mmol scale) with 1-Cp* as Catalyst 86
Decarboxylation of Ibuprofen (**6a**) (1.0 mmol scale) with RhCp* as Catalyst 87

Decarboxylation of 4-phenylphenylacetic acid (**6b**) 88
Decarboxylation phenylmalonic acid (**6c**) 89
Decarboxylation of α, α-difluorophenylacetic acid (**6d**) 89
Decarboxylation of 1,1-diphenylacetic acid (**6e**) 90
Decarboxylation of 1,3-phenyldiacetic acid (**6f**) 90
Mechanistic investigation and optimization 92

X-RAY CRYSTALLOGRAPHIC ANALYSIS 98

[η^6-Benzene-η^5-(2,6-dibromo-4-methoxy-1-phenoxo)Ru](OTf) (**1a**) 98
[η^6-Benzene-η^5-(4-methyl-1-phenoxo)Ru](OTf) (**1h**) 100
[η^6-Benzene-η^5-(4-methoxy-1-phenoxo)Ru](OTf) (**1i**) 102
[η^6-Benzene-η^5-(2,6-dichloro-1-phenoxo)Ru](OTf) (**1g**) 104

COMPUTATIONAL STUDIES 106

Geometry optimizations for local electrophilicity indices 106
Geometry optimizations for arene exchange mechanism 112
Additional computational results of the arene exchange reaction 138
Geometry optimization for additional computational results of the arene exchange reaction 147

SPECTROSCOPIC DATA 200

^{1}H NMR of [η^6-benzene-η^5-(2,6-dibromo-4-methoxy-1-phenoxo)Ru](OTf) (**1a**) 200
^{13}C NMR of [η^6-benzene-η^5-(2,6-dibromo-4-methoxy-1-phenoxo)Ru](OTf) (**1a**) 201
^{19}F NMR of [η^6-benzene-η^5-(2,6-dibromo-4-methoxy-1-phenoxo)Ru](OTf) (**1a**) 202
^{1}H NMR of [η^6-benzene-η^6-(4-methoxymethylanisole)Ru](OTf) (**1b**) 203
^{13}C NMR of [η^6-benzene-η^6-(4-methoxymethylanisole)Ru](OTf) (**1b**) 204
^{19}F NMR of [η^6-benzene-η^6-(4-methoxymethylanisole)Ru](OTf) (**1b**) 205
^{1}H NMR of [η^6-benzene-η^5-(phenoxo)Ru](OTf) (**1c**) 206
^{13}C NMR of [η^6-benzene-η^5-(phenoxo)Ru](OTf) (**1c**) 207
^{19}F NMR of [η^6-benzene-η^5-(phenoxo)Ru](OTf) (**1c**) 208
^{1}H NMR of [η^6-benzene-η^5-(4-butyl-1-phenoxo)Ru](OTf) (**1d**) 209
^{13}C NMR of [η^6-benzene-η^5-(4-butyl-1-phenoxo)Ru](OTf) (**1d**) 210
^{19}F NMR of [η^6-benzene-η^5-(4-butyl-1-phenoxo)Ru](OTf) (**1d**) 211
^{1}H NMR of [η^6-benzene-η^5-(4-(2-methoxyethyl)-1-phenoxo)Ru](OTf) (**1e**) 212
^{13}C NMR of [η^6-benzene-η^5-(4-(2-methoxyethyl)-1-phenoxo)Ru](OTf) (**1e**) 213
^{19}F NMR of [η^6-benzene-η^5-(4-(2-methoxyethyl)-1-phenoxo)Ru](OTf) (**1e**) 214
^{1}H NMR of [η^6-benzene-η^5-(2,6-diiodo-1-phenoxo)Ru](OTf) (**1f**) 215
^{13}C NMR of [η^6-benzene-η^5-(2,6-diiodo-1-phenoxo)Ru](OTf) (**1f**) 216
^{19}F NMR of [η^6-benzene-η^5-(2,6-dichloro-1-phenoxo)Ru](OTf) (**1f**) 217
^{1}H NMR of [η^6-benzene-η^5-(2,6-dichloro-1-phenoxo)Ru](OTf) (**1g**) 218
^{13}C NMR of [η^6-benzene-η^5-(2,6-dichloro-1-phenoxo)Ru](OTf) (**1g**) 219

^{19}F NMR of [*η*^{6}-benzene-*η*^{5}-(2,6-dichloro-1-phenoxo)Ru](OTf) (**1g**) 220
^{1}H NMR of [*η*^{6}-benzene-*η*^{5}-(4-methyl-1-phenoxo)Ru](OTf) (**1h**) 221
^{13}C NMR of [*η*^{6}-benzene-*η*^{5}-(4-methyl-1-phenoxo)Ru](OTf) (**1h**) 222
^{19}F NMR of [*η*^{6}-benzene-*η*^{5}-(4-methyl-1-phenoxo)Ru](OTf) (**1h**) 223
^{1}H NMR of [*η*^{6}-benzene-*η*^{5}-(4-methoxy-1-phenoxo)Ru](OTf) (**1i**) 224
^{13}C NMR of [*η*^{6}-benzene-*η*^{5}-(4-methoxy-1-phenoxo)Ru](OTf) (**1i**) 225
^{19}F NMR of [*η*^{6}-benzene-*η*^{5}-(4-methoxy-1-phenoxo)Ru](OTf) (**1i**) 226
^{1}H NMR of [*η*^{6}-benzene-*η*^{5}-(4-trifluoromethyl-1-phenoxo)-1-phenoxo)Ru](OTf) (**1j**) 227
^{13}C NMR of [*η*^{6}-benzene-*η*^{5}-(4-trifluoromethyl-1-phenoxo)Ru](OTf) (**1j**) 228
^{19}F NMR of [*η*^{6}-benzene-*η*^{5}-(4-trifluoromethyl-1-phenoxo)Ru](OTf) (**1j**) 229
^{1}H NMR of [*η*^{6}-benzene-*η*^{5}-(2,6-dibromo-1-phenoxo)Ru](OTf) (**1k**) 230
^{13}C NMR of [*η*^{6}-benzene-*η*^{5}-(2,6-dibromo-1-phenoxo)Ru](OTf) (**1k**) 231
^{19}F NMR of [*η*^{6}-benzene-*η*^{5}-(2,6-dibromo-1-phenoxo)Ru](OTf) (**1k**) 232
^{1}H NMR of [*η*^{6}-benzene-*η*^{5}-(4-(N,N-dimethylamino)-1-phenoxo)Ru](OTf) (**1l**) 233
^{13}C NMR of [*η*^{6}-benzene-*η*^{5}-(4-(N,N-dimethylamino)-1-phenoxo)Ru](OTf) (**1l**) 234
^{19}F NMR of [*η*^{6}-benzene-*η*^{5}-(4-(N,N-dimethylamino)-1-phenoxo)Ru](OTf) (**1l**) 235
^{1}H NMR of [*η*^{6}-benzene-*η*^{5}-(1-phenoxo-4-phenyl)Ru](OTf) (**SI-1**) 236
^{13}C NMR of [*η*^{6}-benzene-*η*^{5}-(1-phenoxo-4-phenyl)Ru](OTf) (**SI-1**) 237
^{19}F NMR of [*η*^{6}-benzene-*η*^{5}-(1-phenoxo-4-phenyl)Ru](OTf) (**SI-1**) 238
^{1}H NMR of [*η*^{6}-benzene-*η*^{5}-(3,5-dimethoxy-1-phenoxo)Ru](OTf) (**SI-2**) 239
^{13}C NMR of [*η*^{6}-benzene-*η*^{5}-(3,5-dimethoxy-1-phenoxo)Ru](OTf) (**SI-2**) 240
^{19}F NMR of [*η*^{6}-benzene-*η*^{5}-(3,5-dimethoxy-1-phenoxo)Ru](OTf) (**SI-2**) 241
^{1}H NMR of [*η*^{6}-benzene-*η*^{5}-(1-phenoxo-3,4,5-trimethyl)Ru](OTf) (**SI-3**) 242
^{13}C NMR of [*η*^{6}-benzene-*η*^{5}-(1-phenoxo-3,4,5-trimethyl)Ru](OTf) (**SI-3**) 243
^{19}F NMR of [*η*^{6}-benzene-*η*^{5}-(1-phenoxo-3,4,5-trimethyl)Ru](OTf) (**SI-3**) 244
^{1}H NMR of [*η*^{6}-benzene-*η*^{5}-(1-phenoxo-2,4,6-trimethyl)Ru](OTf) (**SI-4**) 245
^{13}C NMR of [*η*^{6}-benzene-*η*^{5}-(1-phenoxo-2,4,6-trimethyl)Ru](OTf) (**SI-4**) 246
^{19}F NMR of [*η*^{6}-benzene-*η*^{5}-(1-phenoxo-2,4,6-trimethyl)Ru](OTf) (**SI-4**) 247
^{1}H NMR of [*η*^{6}-benzene-*η*^{5}-(1-phenoxo-2,6-dimethoxy)Ru](OTf) (**SI-5**) 248
^{13}C NMR of [*η*^{6}-benzene-*η*^{5}-(1-phenoxo-2,6-dimethoxy)Ru](OTf) (**SI-5**) 249
^{19}F NMR of [*η*^{6}-benzene-*η*^{5}-(1-phenoxo-2,6-dimethoxy)Ru](OTf) (**SI-5**) 250
^{1}H NMR of [*η*^{6}-benzene-*η*^{5}-(4-*t*Bu-1-phenoxo)Ru](OTf) (**SI-6**) 251
^{13}C NMR of [*η*^{6}-benzene-*η*^{5}-(4-*t*Bu-1-phenoxo)Ru](OTf) (**SI-6**) 252
^{19}F NMR of [*η*^{6}-benzene-*η*^{5}-(4-*t*Bu-1-phenoxo)Ru](OTf) (**SI-6**) 253
^{1}H NMR of [*η*^{6}-benzene-*η*^{5}-(4-amino-1-phenoxo)Ru](OTf) (**SI-7**) 254
^{13}C NMR of [*η*^{6}-benzene-*η*^{5}-(4-amino-1-phenoxo)Ru](OTf) (**SI-7**) 255
^{19}F NMR of [*η*^{6}-benzene-*η*^{5}-(4-amino-1-phenoxo)Ru](OTf) (**SI-7**) 256
^{1}H NMR of [*η*^{6}-ethylbenzoate-*η*^{5}-(2,6-dichloro-1-phenoxo)Ru](OTf) (**SI-8**) 257
^{13}C NMR of [*η*^{6}-ethylbenzoate-*η*^{5}-(2,6-dichloro-1-phenoxo)Ru](OTf) (**SI-8**) 258
^{19}F NMR of [*η*^{6}-ethylbenzoate-*η*^{5}-(2,6-dichloro-1-phenoxo)Ru](OTf) (**SI-8**) 259

^{1}H NMR of [η^6-benzene-η^5-(2-chloro-1-phenoxo)Ru](OTf) (**SI-9**) 260
^{13}C NMR of [η^6-benzene-η^5-(2-chloro-1-phenoxo)Ru](OTf) (**SI-9**) 261
^{19}F NMR of [η^6-benzene-η^5-(2-chloro-1-phenoxo)Ru](OTf) (**SI-9**) 262
^{1}H NMR of phenyl-epiandrosterone ester (**4a**) 263
^{13}C NMR of phenyl-epiandrosterone ester (**4a**) 264
^{1}H NMR of 1-nitro-4-(phenoxymethyl)benzene (**4b**) 265
^{13}C NMR of 1-nitro-4-(phenoxymethyl)benzene (**4b**) 266
^{1}H NMR of (cyclohexylmethoxy)benzene (**4c**) 267
^{13}C NMR of (cyclohexylmethoxy)benzene (**4c**) 268
^{1}H NMR of protodecarboxylated Ibuprofen (**5a**) 269
^{13}C NMR of protodecarboxylated Ibuprofen (**5a**) 270
^{1}H NMR of 1-methyl-4-phenylbenzene (**5b**) 271
^{13}C NMR of 1-methyl-4-phenylbenzene (**5b**) 272
^{1}H NMR of protodecarboxylated phenylmalonic acid (**5c**) 273
^{13}C NMR of protodecarboxylated phenylmalonic acid (**5c**) 274
^{1}H NMR of difluoromethylbenzene (**5d**) 275
^{13}C NMR of difluoromethylbenzene (**5d**) 276
^{19}F NMR of difluoromethylbenzene (**5d**) 277
^{1}H NMR of 1,1-diphenylmethane (**5e**) 278
^{13}C NMR of 1,1-diphenylmethane (**5e**) 279
^{1}H NMR of 1,3-dimethylbenzene (**5f**) 280
^{13}C NMR of 1,3-dimethylbenzene (**5f**) 281

REFERENCES 282

MATERIALS AND METHODS

All air- and moisture-insensitive reactions were carried out under ambient atmosphere and monitored by thin-layer chromatography (TLC). Air- and moisture-sensitive manipulations were performed using standard Schlenk techniques under an atmosphere of argon. Concentration under reduced pressure was performed by rotary evaporation at 40 °C at an appropriate pressure if not otherwise stated. Purified compounds were further dried under high vacuum (10^{-6} – 10^{-3} bar). Yields refer to purified and spectroscopically pure compounds. 23 °C is defined as a temperature range from 22–25 °C.

Starting Materials

All substrates were used as received from *Sigma-Aldrich, Alfa Aesar, Apollo Scientific, abcr GmbH, TCI, SCBT* and *Fluorochem*, unless otherwise stated. Ligand 2,6-dibromo-4-methoxyphenol,[1] complex [η^6-anisole-η^5-(pentamethylcyclopentadienyl)Rh]$(OTf)_2$ (RhCp*),[2] (ethylbenzoate)ruthenium dichloride dimer,[3] and TBA-$OSO_2Ph(NO_2)_2$ [4] were prepared according to literature procedures. The analytical data is in agreement with the literature.

Solvents

Anhydrous toluene was obtained from *Phoenix Solvent Drying Systems*. Anhydrous acetone and 1,2-dichloroethane were obtained from *Thermo Fisher Scientific*. Anhydrous 1,4-dioxane was obtained from *Sigma-Aldrich*. All deuterated solvents were purchased from *Euriso-Top*.

Chromatography

TLC was performed using Polygram Sil G/UV254 plates from *Macherey-Nagel* pre-coated with 0.20 mm silica and a fluorescence indicator. Plates were visualized by irradiation at 254 nm from a *Herolab* UV-lamp and $KMnO_4$ stain. Flash column chromatography was performed using Biotage® Sfär Silica columns (100 Å pore size, 60 µm particle size) on an automated purification system (Biotage® Isolera One). The detailed solvent composition of the eluents is given for every compound individually.

Spectroscopy and Instruments

Chemical shifts are reported in ppm with the solvent residual peak as the internal standard. For ^1H NMR: $CDCl_3$, δ 7.26; CD_3OD, δ 3.31. For ^{13}C NMR: $CDCl_3$, δ 77.2; CD_3OD, δ 49.0. ^{19}F NMR spectra were referenced using a unified chemical shift scale based on the ^1H resonance of tetramethylsilane (1% v/v solution in the respective solvent). Data are reported as follows: s = singlet, d = doublet, t = triplet, q = quartet, quint = quintet, m = multiplet, br = broad; coupling constants in Hz. NMR spectra were recorded on a *Bruker AVANCE Neo* 600 MHz equipped with a BBO cryoprobe, a *Bruker AVANCE III* 600 MHz equipped with a TCI cryoprobe, a *Bruker AVANCE IIIHD* 500 MHz Kinetic NMR experiments at elevated temperatures were performed on a *Bruker AVANCE III* 500 MHz NMR spectrometer equipped with a BBFO probe. Liquid-chromatography-mass spectrometry (LC-MS) was performed with an Agilent Technology 1260 Infinity HPLC system coupled to an *Agilent Technologies* 6120 Quadrupole mass analyzer. Gas-chromatography-mass spectrometry (GC-MS)

was performed with a *Shimadzu* GCMS-QP2010 or *Shimadzu* GCMS-QP2020 NX gas chromatograph both equipped with a flame-ionization detector.

Elemental Analysis

Carbon, hydrogen, nitrogen, and sulfur were measured using a CHNS-Analyzer of Elementar model Vario Mikro. Fluorine, chlorine, and bromine were measured using ionchromatography on a Metrohm model 930 Compact IC Flex Oven/SeS/PP/Deg followed by combustion analysis on a Mitsubishi AQF-2100H. Ruthenium was measured using microwave analysis on a CEM Mars 6 with an ICP of Spectro model Spectro Acros.

EXPERIMENTAL DATA

Complex Synthesis

Reaction set up:

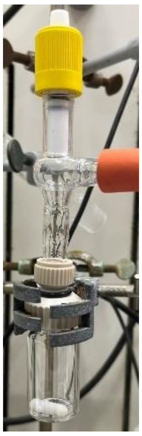

The phenoxo complexes were synthesized following a modified version of the protocol reported by Matheson.[5] Complexes bearing halide substituents in the 2,6-positions were directly isolated as the phenoxo complex, all others were first isolated as the dicationic phenol complex and subsequently deprotonated to the phenoxo complex using Diisopropylethylamin (DIPEA).[5] Ligands containing nitrogen substituents (amine, *N*,*N*-dimethylamine), had to be protonated prior to the complexation to avoid nitrogen coordination. Triflic acid was used as the acid of choice to prevent mixed counterions. Complexes were synthesized using custom-made Schlenk line adapters for 20 mL vials.

[η^6-Benzene-η^5-(2,6-dibromo-4-methoxy-1-phenoxo)Ru](OTf) (1a)

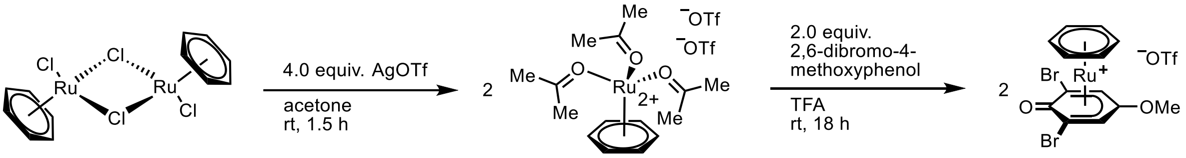

1a, 27%

A 20 mL borosilicate vial equipped with a Teflon-coated magnetic stirring bar was charged with (benzene)ruthenium dichloride dimer (0.10 g, 0.20 mmol, 1.0 equiv.) and AgOTf (0.21 g, 0.80 mmol, 4.0 equiv.). The vial was connected via a vial adapter to a Schlenk line and was evacuated and purged with argon three times. Anhydrous acetone (5.0 mL, *c* = 40 mM) was added, the vial covered with aluminum foil and the suspension stirred at 23 °C for 1.5 h under an argon atmosphere. Subsequently, the stirring bar was removed, the vial closed with a Teflon-lined screw cap and centrifuged for 6 min at 3000 rpm. 2,6-Dibromo-4-methoxyphenol (0.11 g, 0.40 mmol, 2.0 equiv.) was added to a second 20 mL borosilicate vial equipped with a Teflon-coated magnetic stirring bar and a vial adapter. The vial adapter was evacuated and purged with argon three times, after which the supernatant from the first vial was added. The solvent was evaporated under high vacuum and TFA (5.0 mL, *c* = 80 mM) added under an argon atmosphere. The resulting solution was stirred at 23 °C for 16 h. The vial was then cooled in ice water and diethyl ether (15 mL) was added to the vial to precipitate a colorless solid. The solid was filtered off, washed with diethyl ether (3 × 10 mL), and dried under high vacuum. The crude complex was recrystallized by heating an Et_2O (10 mL) suspension of the complex under reflux and adding MeOH (~2 mL) dropwise until a solution was obtained. The solution was subsequently stored in the freezer for 3 days to give yellow needles. The needles were filtered off, washed with Et_2O (10 mL) and dried in high vacuum to afford **1a** (67 mg, 0.11 mmol, 27%) as yellow needles.

NMR Spectroscopy:

^{1}H NMR (600 MHz, methanol-d_4, 23 °C, δ): 7.15 (s, 2H), 6.59 (s, 6H), 3.93 (s, 3H).

^{13}C NMR (151 MHz, methanol-d_4, 23 °C, δ): 157.3, 132.7, 121.8 (q, J = 318.6 Hz), 96.3, 84.7, 83.3,

59.6.

^{19}F NMR (565 MHz, methanol-d_4, 23 °C, δ): −80.00.

HRMS ESI (m/z) calculated for $C_{13}H_{11}Br_2O_2Ru_1$ $[M]^+$, 458.8164; found, 458.8157 deviation: +1.6 ppm.

Elemental analysis calculated for $C_{14}H_{11}Br_2F_3O_5Ru_1S_1$: C, 27.60; H, 1.82; S, 5.26; Br, 26.23; F, 9.36; O, 13.13; Ru, 16.59; found: C, 27.65; H, 1.84; S, 5.24; Br, 26.27; F, 9.31; Ru, 16.51.

Melting point: 239 °C (decomposition).

[η^6-Benzene-η^6-(4-(2-methoxyethyl)anisole)Ru](OTf)$_2$ (1b)

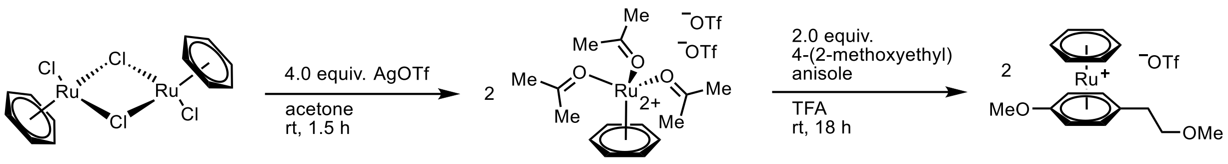

1b, 45%

A 20 mL borosilicate vial equipped with a Teflon-coated magnetic stirring bar was charged with (benzene)ruthenium dichloride dimer (0.10 g, 0.20 mmol, 1.0 equiv.) and AgOTf (0.21 g, 0.80 mmol, 4.0 equiv.). The vial was connected via a vial adapter to a Schlenk line and was evacuated and purged with argon three times. Anhydrous acetone (5.0 mL, c = 40 mM) was added, the vial covered with aluminum foil and the suspension stirred at 23 °C for 1.5 h under an argon atmosphere. Afterwards, the stirring bar was removed, the vial closed with a Teflon-lined screw cap and centrifuged for 6 min at 3000 rpm. Meanwhile, 4-(2-methoxyethyl)anisole (67 mg, 0.40 mmol, 2.0 equiv.) was added to a second 20 mL borosilicate vial equipped with a Teflon-coated magnetic stirring bar and a vial adapter. The vial adapter was evacuated and purged with argon three times, after which the supernatant from the first vial was added. The solvent was evaporated under high vacuum and TFA (5.0 mL, *c* = 80 mM) added under an argon atmosphere. The resulting solution was stirred at 23 °C for 16 h. The vial was then cooled in ice water and diethyl ether (15 mL) was added to the vial to precipitate a colorless solid. The crude complex was recrystallized by heating an Et_2O (10 mL) suspension of the complex under reflux and adding MeOH (~1.5 mL) dropwise until a solution was obtained. The solution was subsequently stored in the freezer for 3 days to give colorless crystals. The crystals were filtered off, washed with Et_2O (10 mL) and dried in high vacuum to afford **1b** (0.12 g, 0.18 mmol, 45%) as a colorless solid.

NMR Spectroscopy:

> **^{1}H NMR** (600 MHz, MeCN-d_3, 23 °C, δ): 6.79 (s, 6H), 6.95 (s, 4H), 3.99 (s, 3H), 6.66 – 6.64 (m, 2H), 3.34 (s, 3H), 2.88 – 3.87 (m, 2H).
>
> **^{13}C NMR** (151 MHz, MeCN-d_3, 23 °C, δ): 143.3, 122.1 (q, J = 320.7 Hz), 111.3, 95.6, 94.6, 80.8, 70.1, 60.3, 59.1, 33.4.
>
> **^{19}F NMR** (565 MHz, MeCN-d_3, 23 °C, δ): −79.33.

HRMS ESI (m/z) calculated for $C_{16}H_{20}O_2Ru_1$ $[M]^+$, 173.0248; found, 173.0249 deviation: −0.7 ppm.

Melting point: 115 °C(decomposition).

[η^6-Benzene-η^5-(phenoxo)Ru](OTf) (1c)

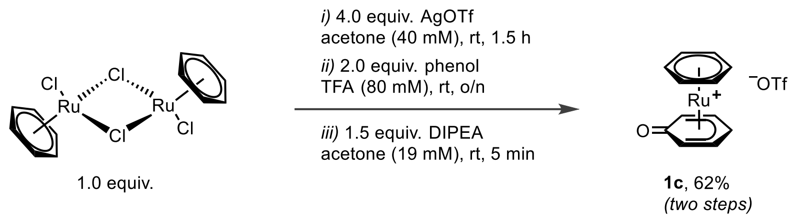

A 20 mL borosilicate vial equipped with a Teflon-coated magnetic stirring bar was charged with (benzene)ruthenium dichloride dimer (0.10 g, 0.20 mmol, 1.0 equiv.) and AgOTf (0.21 g, 0.80 mmol, 4.0 equiv.). The vial was connected via a vial adapter to a Schlenk line and was evacuated and purged with argon three times. Subsequently, anhydrous acetone (5.0 mL, *c* = 40 mM) was added, the vial was covered with aluminum foil and the suspension was stirred at 23 °C for 1.5 h under an argon atmosphere. Then, the orange reaction mixture was centrifuged at 3000 rpm for 8 min. Meanwhile, a second 20 mL borosilicate vial equipped with a Teflon-coated magnetic stir bar and a vial adapter was charged with phenol (38 mg, 0.40 mmol, 2.0 equiv.). Under an argon atmosphere, the orange supernatant from the first vial was added and the solvent was removed under high vacuum, while stirring. Afterwards, trifluoroacetic acid (5.0 mL, *c* = 80 mM, TFA) was added under an argon atmosphere, and the resulting solution was stirred at 23 °C for 16 h. Then, diethyl ether (15 mL) was added at 0 °C to precipitate a white solid. The solid was filtered off over a G4 glass frit, washed with diethyl ether (3 × 15 mL), and dried under high vacuum to afford the Ru(II) precatalyst (0.15 g, 0.26 mmol, 64%) as a white solid.

The corresponding Ru(II) pre-catalyst (0.10 g, 0.18 mmol, 1.0 equiv.) was transferred to a 20 mL borosilicate vial equipped with a Teflon-coated magnetic stir bar. After addition of acetone (5.0 mL) and DIPEA (46 µL, 0.27 mmol, 1.5 equiv.), the resulting solution was stirred at 23 °C for 5 min under ambient atmosphere. Then, diethyl ether (15 mL) was added to precipitate a solid. The solid was filtered off over a G4 glass frit, washed with diethyl ether (3 × 15 mL), and dried under high vacuum to afford Ru(II) complex **1c** (71 mg, 0.17 mmol, 97%) as a grey solid.

NMR Spectroscopy:

^{1}H NMR (600 MHz, methanol-d_4, 23 °C, δ): 6.60 (s, 1H), 6.41 – 6.24 (m, 3H), 5.78 – 5.69 (m, 2H).

^{13}C NMR (151 MHz, methanol-d_4, 23 °C, δ): 163.2, 121.8 (q, *J* = 318.4 Hz), 95.5, 92.3, 84.5, 81.0.

^{19}F NMR (565 MHz, methanol-d_4, 23 °C, δ): −80.01.

HRMS ESI (m/z) calculated for $C_{12}H_{11}O_1Ru_1$ [M]$^+$, 272.9848; found, 272.9850 deviation: −0.7 ppm.

Melting point: 193 °C (decomposition).

[η^6-Benzene-η^5-(4-butyl-1-phenoxo)Ru](OTf) (1d)

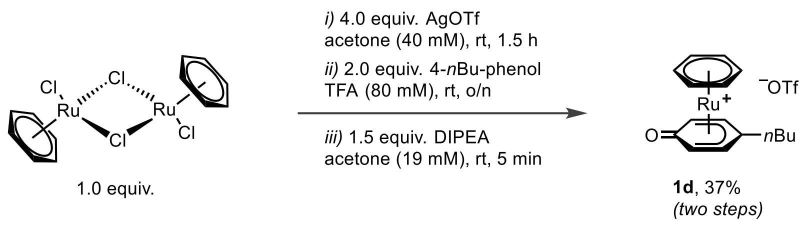

A 20 mL borosilicate vial equipped with a Teflon-coated magnetic stirring bar was charged with (benzene)ruthenium dichloride dimer (0.10 g, 0.20 mmol, 1.0 equiv.) and AgOTf (0.21 g, 0.80 mmol, 4.0 equiv.). The vial was connected via a vial adapter to a Schlenk line and was evacuated and purged with argon three times. Subsequently, anhydrous acetone (5.0 mL, *c* = 40 mM) was added, the vial was covered with aluminum foil and the suspension was stirred at 23 °C for 1.5 h under an argon atmosphere. Then, the orange reaction mixture was centrifuged at 3000 rpm for 8 min. Meanwhile, a second 20 mL borosilicate vial equipped with a Teflon-coated magnetic stir bar and a vial adapter was charged with 4-*n*-butylphenol (60 mg, 0.40 mmol, 2.0 equiv.). Under an argon atmosphere, the orange supernatant from the first vial was added and the solvent was removed under high vacuum, while stirring. Afterwards, trifluoroacetic acid (5.0 mL, *c* = 80 mM, TFA) was added under an argon atmosphere, and the resulting solution was stirred at 23 °C for 16 h. Then, diethyl ether (15 mL) was added at 0 °C to precipitate a white solid. The solid was filtered off over a G4 glass frit, washed with diethyl ether (3 × 15 mL), and dried under high vacuum to afford the Ru(II) precatalyst (0.19 g, 0.30 mmol, 75%) as a white solid.

The corresponding Ru(II) pre-catalyst (0.19 g, 0.30 mmol, 1.0 equiv.) was transferred to a 20 mL borosilicate vial equipped with a Teflon-coated magnetic stir bar. After addition of acetone (5.0 mL) and DIPEA (0.10 mL, 77 mg, 0.60 mmol, 1.5 equiv.), the resulting solution was stirred at 23 °C for 5 min under ambient atmosphere. Then, diethyl ether (15 mL) was added to precipitate a solid. The solid was filtered off over a G4 glass frit, washed with diethyl ether (3 × 15 mL), and dried under high vacuum to afford Ru(II) complex **1d** (70 mg, 0.15 mmol, 49%) as a beige solid.

NMR Spectroscopy:

> **^{1}H NMR** (600 MHz, methanol-d_4, 23 °C, δ): 6.57 (s, 6H), 6.31 (d, *J* = 7.2 Hz, 2H), 5.66 (d, *J* = 7.2 Hz, 2H), 2.76 – 2.38 (m, 2H), 1.65 (dddd, *J* = 9.0, 7.8, 6.8, 5.8 Hz, 2H), 1.56 – 1.32 (m, 2H), 1.00 (t, *J* = 7.4 Hz, 3H).

> **^{13}C NMR** (151 MHz, methanol-d_4, 23 °C, δ): 163.0, 121.8 (q, *J* = 318.6 Hz), 106.9, 95.5, 92.6, 80.0, 34.2, 34.0, 23.3, 14.0.

> **^{19}F NMR** (565 MHz, methanol-d_4, 23 °C, δ): −80.04.

HRMS ESI (m/z) calculated for $C_{16}H_{19}O_1Ru_1$ $[M]^+$, 329.0474; found, 329.0475 deviation: −0.4 ppm.

Melting point: 134 °C (decomposition).

[η^6-Benzene-η^5-(4-(2-methoxyethyl)-1-phenoxo)Ru](OTf) (1e)

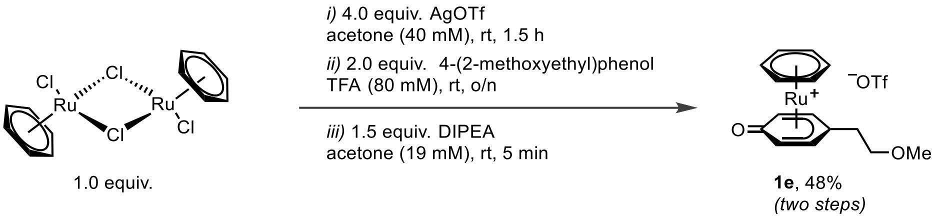

A 20 mL borosilicate vial equipped with a Teflon-coated magnetic stirring bar was charged with (benzene)ruthenium dichloride dimer (0.10 g, 0.20 mmol, 1.0 equiv.) and AgOTf (0.21 g, 0.80 mmol, 4.0 equiv.). The vial was connected via a vial adapter to a Schlenk line and was evacuated and purged with argon three times. Subsequently, anhydrous acetone (5.0 mL, *c* = 40 mM) was added, the vial was covered with aluminum foil and the suspension was stirred at 23 °C for 1.5 h under an argon atmosphere. Then, the orange reaction mixture was centrifuged at 3000 rpm for 8 min. Meanwhile, a second 20 mL borosilicate vial equipped with a Teflon-coated magnetic stir bar and a vial adapter was charged with 4-(2-methoxyethyl)phenol (61 mg, 0.40 mmol, 2.0 equiv.). Under an argon atmosphere, the orange supernatant from the first vial was added and the solvent was removed under high vacuum, while stirring. Afterwards, trifluoroacetic acid (5.0 mL, *c* = 80 mM, TFA) was added under an argon atmosphere, and the resulting solution was stirred at 23 °C for 16 h. Then, diethyl ether (15 mL) was added at 0 °C to precipitate a white solid. The solid was filtered off over a G4 glass frit, washed with diethyl ether (3 × 15 mL), and dried under high vacuum to afford the Ru(II) precatalyst (0.14 g, 0.22 mmol, 55%) as a white solid.

The corresponding Ru(II) pre-catalyst (0.14 g, 0.22 mmol, 1.0 equiv.) was transferred to a 20 mL borosilicate vial equipped with a Teflon-coated magnetic stir bar. After addition of acetone (5.0 mL) and DIPEA (58 µL, 0.33 mmol, 1.5 equiv.), the resulting solution was stirred at 23 °C for 5 min under ambient atmosphere. Then, diethyl ether (15 mL) was added to precipitate a solid. The solid was filtered off over a G4 glass frit, washed with diethyl ether (3 × 15 mL), and dried under high vacuum to afford Ru(II) complex **1e** (89 mg, 0.19 mmol, 84%) as a beige solid.

NMR Spectroscopy:

^{1}**H NMR** (600 MHz, methanol-d_4, 23 °C, δ): 6.48 (s, 6H), 6.25 (d, *J* = 7.3 Hz, 2H), 5.59 (d, *J* = 7.3 Hz, 2H), 3.60 (m, 2H), 3.31 (s, 3H), 2,72 (m, 2H).

^{13}**C NMR** (151 MHz, methanol-d_4, 23 °C, δ): 163.1, 121.82 (q, *J* = 318.5 Hz), 104,1, 95.8, 92.7, 79.9, 71.6, 59.06, 34.2.

^{19}**F NMR** (565 MHz, methanol-d_4, 23 °C, δ): −80.2.

HRMS ESI (m/z) calculated for $C_{15}H_{17}O_2Ru_1$ [M]$^+$, 331.0267; found, 331.0268, deviation: −0.5 ppm.

Melting point: 168 °C.

[η^6-Benzene-η^5-(2,6-diiodo-1-phenoxo)Ru](OTf) (1f)

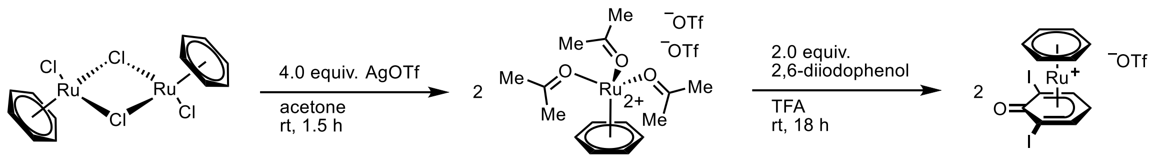

1f, 64%

A 20 mL borosilicate vial equipped with a Teflon-coated magnetic stirring bar was charged with (benzene)ruthenium dichloride dimer (0.10 g, 0.20 mmol, 1.0 equiv.) and AgOTf (0.21 g, 0.80 mmol, 4.0 equiv.). The vial was connected via a vial adapter to a Schlenk line and was evacuated and purged with argon three times. Anhydrous acetone (5.0 mL, c = 40 mM) was added, the vial covered with aluminum foil and the suspension stirred at 23 °C for 1.5 h under an argon atmosphere. Afterwards, the stirring bar was removed, the vial closed with a Teflon-lined screw cap and centrifuged for 6 min at 3000 rpm. Meanwhile, 2,6-diiodophenol (0.14 g, 0.40 mmol, 2.0 equiv.) was added to a second 20 mL borosilicate vial equipped with a Teflon-coated magnetic stirring bar and a vial adapter. The vial adapter was evacuated and purged with argon three times, after which the supernatant from the first vial was added. The solvent was evaporated under high vacuum and TFA (5.0 mL, c = 80 mM) added under an argon atmosphere. The resulting solution was stirred at 23 °C for 16 h. The vial was then cooled in ice water and diethyl ether (15 mL) was added to the vial to precipitate a colorless solid. The solid was filtered off, washed with diethyl ether (3 × 10 mL), and dried under high vacuum. The crude complex was recrystallized by heating an Et_2O (10 mL) suspension of the complex under reflux and adding MeOH (~2 mL) dropwise until a solution was obtained. The solution was subsequently stored in the freezer for 3 days to give light-yellow needles. The needles were filtered off, washed with Et_2O (10 mL) and dried in high vacuum to afford **1f** (0.17 g, 0.37 mmol, 64%) as a light-yellow solid.

NMR Spectroscopy:

^{1}H NMR (600 MHz, methanol-*d*$_4$, 23 °C, δ): 6.99 (d, *J* = 5.4 Hz, 2H), 6.49 (s, 6H), 6.10 (t, *J* = 5.5 Hz, 1H).

^{13}C NMR (151 MHz, methanol-*d*$_4$, 23 °C, δ): 161.5, 121.8 (q, *J* = 318.4 Hz), 103.5, 96.0, 85.9, 56.1.

^{19}F NMR (565 MHz, methanol-*d*$_4$, 23 °C, δ): –80.1.

HRMS ESI (m/z) calculated for $C_{12}H_9O_1I_2Ru_1$ $[M]^+$, 524.7781; found, 524.7785, deviation: –0.9 ppm.

Melting point: 236 °C (decomposition).

[η^6-Benzene-η^5-(2,6-dichloro-1-phenoxo)Ru](OTf) (1g)

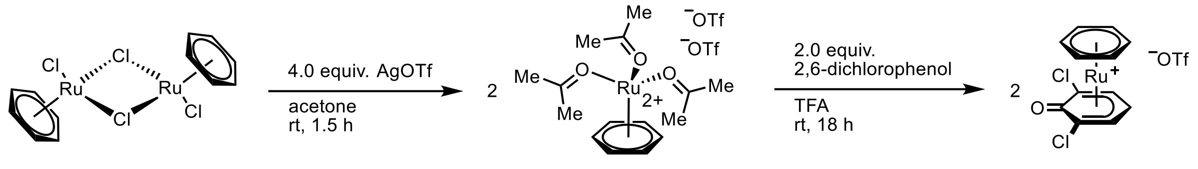

1g, 79%

A 20 mL borosilicate vial equipped with a Teflon-coated magnetic stirring bar was charged with (benzene)ruthenium dichloride dimer (0.10 g, 0.20 mmol, 1.0 equiv.) and AgOTf (0.21 g, 0.80 mmol, 4.0 equiv.). The vial was connected via a vial adapter to a Schlenk line and was evacuated and purged with argon three times. Anhydrous acetone (5.0 mL, c = 40 mM) was added, the vial covered with aluminum foil and the suspension stirred at 23 °C for 1.5 h under an argon atmosphere. Afterwards, the stirring bar was removed, the vial closed with a Teflon-lined screw cap and centrifuged for 6 min at 3000 rpm. Meanwhile, 2,6-dichlorophenol (65 mg, 0.40 mmol, 2.0 equiv.) was added to a second 20 mL borosilicate vial equipped with a Teflon-coated magnetic stirring bar and a vial adapter. The vial adapter was evacuated and purged with argon three times, after which the supernatant from the first vial was added. The solvent was evaporated under high vacuum and TFA (5.0 mL) added under an argon atmosphere. The resulting solution was stirred at 23 °C for 16 h. The vial was then cooled in ice water and diethyl ether (15 mL) was added to the vial to precipitate a colorless solid. The solid was filtered off, washed with diethyl ether (3 × 10 mL), and dried under high vacuum to afford **1g** (0.15 g, 0.32 mmol, 79%) as a light-yellow solid.

NMR Spectroscopy:

^{1}**H NMR** (600 MHz, methanol-d_4, 23 °C, δ): 6.88 (d, J = 5.6 Hz, 2H), 6.63 (s, 6H), 6.31 (t, J = 5.6 Hz, 1H).

^{13}**C NMR** (151 MHz, methanol-d_4, 23 °C, δ): 157.4, 121.8 (q, J = 318.5 Hz), 100.8, 95.6, 95.3, 81.5.

^{19}**F NMR** (565 MHz, methanol-d_4, 23 °C, δ): –80.0.

HRMS ESI (m/z) calculated for $C_{12}H_9Cl_2O_1Ru_1$ [M]$^+$, 340.9069; found, 340.9070, deviation: –0.4 ppm.

Elemental analysis calculated for $C_{13}H_9Cl_2F_3O_4Ru_1S_1$: C, 31.85; H, 1.85; S, 6.54; Cl, 14.46; F, 11.63; O, 13.05; Ru, 20.62; found: C, 31.78; H, 1.85; S, 6.55; Cl, 14.43; F, 11.57; Ru, 20.59.

Melting point: 167 °C.

[η^6-Benzene-η^5-(4-methyl-1-phenoxo)Ru](OTf) (1h)

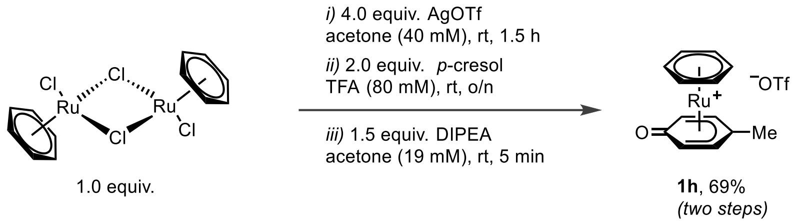

A 20 mL borosilicate vial equipped with a Teflon-coated magnetic stirring bar was charged with (benzene)ruthenium dichloride dimer (0.10 g, 0.20 mmol, 1.0 equiv.) and AgOTf (0.21 g, 0.80 mmol, 4.0 equiv.). The vial was connected via a vial adapter to a Schlenk line and was evacuated and purged with argon three times. Subsequently, anhydrous acetone (5.0 mL, *c* = 40 mM) was added, the vial was covered with aluminum foil and the suspension was stirred at 23 °C for 1.5 h under an argon atmosphere. Then, the orange reaction mixture was centrifuged at 3000 rpm for 8 min. Meanwhile, a second 20 mL borosilicate vial equipped with a Teflon-coated magnetic stir bar and a vial adapter was charged with *p*-cresol (43 mg, 0.40 mmol, 2.0 equiv.). Under an argon atmosphere, the orange supernatant from the first vial was added and the solvent was removed under high vacuum, while stirring. Afterwards, trifluoroacetic acid (5.0 mL, *c* = 80 mM, TFA) was added under an argon atmosphere, and the resulting solution was stirred at 23 °C for 16 h. Then, diethyl ether (15 mL) was added at 0 °C to precipitate a white solid. The solid was filtered off over a G4 glass frit, washed with diethyl ether (3 × 15 mL), and dried under high vacuum to afford the Ru(II) precatalyst (0.17 g, 0.28 mmol, 71%) as a grey solid.

The corresponding Ru(II) pre-catalyst (0.17 g, 0.28 mmol, 1.0 equiv.) was transferred to a 20 mL borosilicate vial equipped with a Teflon-coated magnetic stir bar. After addition of acetone (5.0 mL) and DIPEA (74 µL, 0.43 mmol, 1.5 equiv.), the resulting solution was stirred at 23 °C for 5 min under ambient atmosphere. Then, diethyl ether (15 mL) was added to precipitate a solid. The solid was filtered off over a G4 glass frit, washed with diethyl ether (3 × 15 mL), and dried under high vacuum to afford Ru(II) complex **1h** (0.12 gg, 0.28 mmol, 97%) as a gray solid.

NMR Spectroscopy:

^{1}**H NMR** (600 MHz, methanol-*d*$_4$, 23 °C, δ): 6.56 (s, 6H), 6.31 (d, *J* = 7.1 Hz, 2H), 5.64 (d, *J* = 7.2 Hz, 2H), 2.32 (s, 3H).

^{13}**C NMR** (151 MHz, methanol-*d*$_4$, 23 °C, δ): 162.9, 121.8 (q, *J* = 318.5 Hz), 102.9, 96.2, 92.6, 79.8, 19.2.

^{19}**F NMR** (565 MHz, methanol-*d*$_4$, 23 °C, δ): −80.10.

HRMS ESI (m/z) calculated for $C_{13}H_{13}O_1Ru_1$ [M]$^+$, 287.0004; found, 287.0004, deviation: +0.1 ppm.

Melting point: 221 °C (decomposition).

[η^6-Benzene-η^5-(4-methoxy-1-phenoxo)Ru](OTf) (1i)

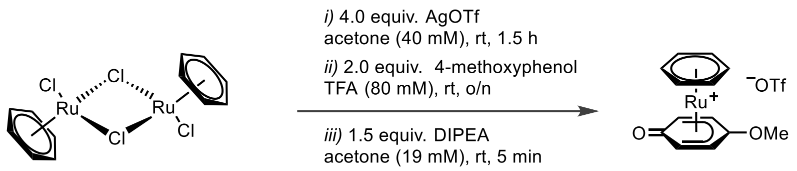

1.0 equiv.

1i, 68%
(two steps)

A 20 mL borosilicate vial equipped with a Teflon-coated magnetic stirring bar was charged with (benzene)ruthenium dichloride dimer (0.10 g, 0.20 mmol, 1.0 equiv.) and AgOTf (0.21 g, 0.80 mmol, 4.0 equiv.). The vial was connected via a vial adapter to a Schlenk line and was evacuated and purged with argon three times. Subsequently, anhydrous acetone (5.0 mL, c = 40 mM) was added, the vial was covered with aluminum foil and the suspension was stirred at 23 °C for 1.5 h under an argon atmosphere. Then, the orange reaction mixture was centrifuged at 3000 rpm for 8 min. Meanwhile, a second 20 mL borosilicate vial equipped with a Teflon-coated magnetic stir bar and a vial adapter was charged with 4-methoxyphenol (50 mg, 0.40 mmol, 2.0 equiv.). Under an argon atmosphere, the orange supernatant from the first vial was added and the solvent was removed under high vacuum, while stirring. Afterwards, trifluoroacetic acid (5.0 mL, c = 80 mM, TFA) was added under an argon atmosphere, and the resulting solution was stirred at 23 °C for 16 h. Then, diethyl ether (15 mL) was added at 0 °C to precipitate a white solid. The solid was filtered off over a G4 glass frit, washed with diethyl ether (3 × 15 mL), and dried under high vacuum to afford the Ru(II) precatalyst (0.17 g, 0.28 mmol, 69%) as a white solid.

The corresponding Ru(II) pre-catalyst (0.10 g, 0.17 mmol, 1.0 equiv.) was transferred to a 20 mL borosilicate vial equipped with a Teflon-coated magnetic stir bar. After addition of acetone (5.0 mL) and DIPEA (44 µL, 0.25 mmol, 1.5 equiv.), the resulting solution was stirred at 23 °C for 5 min under ambient atmosphere. Then, diethyl ether (15 mL) was added to precipitate a solid. The solid was filtered off over a G4 glass frit, washed with diethyl ether (3 × 15 mL), and dried under high vacuum to afford Ru(II) complex **1i** (74 mg, 0.16 mmol, 99%) as a beige solid.

NMR Spectroscopy:

> **^{1}H NMR** (600 MHz, methanol-d_4, 23 °C, δ): 6.60 (s, 6H), 6.41 (d, J = 7.4 Hz, 2H), 5.55 (d, J = 7.4 Hz, 2H), 3.87 (s, 1H).
>
> **^{13}C NMR** (151 MHz, methanol-d_4, 23 °C, δ): 161.4, 135.0, 121.8 (q, J = 318.3 Hz), 92.6, 81.3, 76.6, 58.7.
>
> **^{19}F NMR** (565 MHz, methanol-d_4, 23 °C, δ): −80.05.

HRMS ESI (m/z) calculated for $C_{13}H_{13}O_2Ru_1$ [M]$^+$, 302.9954; found, 302.9955, deviation: −0.4 ppm.

Elemental analysis calculated for $C_{14}H_{13}F_3O_5Ru_1S_1$: C, 37.25; H, 2.90; S, 7.10; F, 12.63; O, 17.72; Ru, 22.39; found: C, 37.21; H, 2.90; S, 7.09; F, 12.58; Ru, 22.34.

Melting point: 198 °C (decomposition).

[η^6-Benzene-η^5-(4-trifluoromethyl-1-phenoxo)Ru](OTf) (1j)

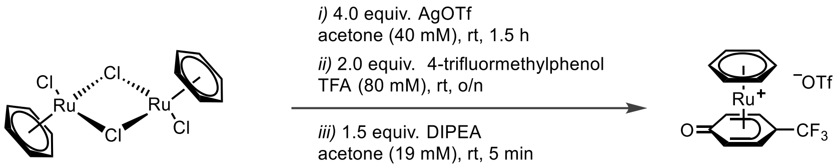

1.0 equiv.

1j, 48%
(two steps)

A 20 mL borosilicate vial equipped with a Teflon-coated magnetic stirring bar was charged with (benzene)ruthenium dichloride dimer (0.10 g, 0.20 mmol, 1.0 equiv.) and AgOTf (0.21 g, 0.80 mmol, 4.0 equiv.). The vial was connected via a vial adapter to a Schlenk line and was evacuated and purged with argon three times. Subsequently, anhydrous acetone (5.0 mL, c = 40 mM) was added, the vial was covered with aluminum foil and the suspension was stirred at 23 °C for 1.5 h under an argon atmosphere. Then, the orange reaction mixture was centrifuged at 3000 rpm for 8 min. Meanwhile, a second 20 mL borosilicate vial equipped with a Teflon-coated magnetic stir bar and a vial adapter was charged with 4-trifluoromethylphenol (57 mg, 0.40 mmol, 2.0 equiv.). Under an argon atmosphere, the orange supernatant from the first vial was added and the solvent was removed under high vacuum, while stirring. Afterwards, trifluoroacetic acid (5.0 mL, c = 80 mM, TFA) was added under an argon atmosphere, and the resulting solution was stirred at 23 °C for 16 h. Then, diethyl ether (15 mL) was added at 0 °C to precipitate a white solid. The solid was filtered off over a G4 glass frit, washed with diethyl ether (3 × 15 mL), and dried under high vacuum to afford the Ru(II) precatalyst (0.13 g, 0.20 mmol, 50%) as a yellowish solid.

The corresponding Ru(II) pre-catalyst (0.13 g, 0.20 mmol, 1.0 equiv.) was transferred to a 20 mL borosilicate vial equipped with a Teflon-coated magnetic stir bar. After addition of acetone (5.0 mL) and DIPEA (70 µL, 0.4 mmol, 2.0 equiv.), the resulting solution was stirred at 23 °C for 5 min under ambient atmosphere. Then, diethyl ether (15 mL) was added to precipitate a solid. The solid was filtered off over a G4 glass frit, washed with diethyl ether (3 × 15 mL), and dried under high vacuum to afford Ru(II) complex **1j** (94 mg, 0.19 mmol, 96%) as a yellowish solid.

NMR Spectroscopy:

^{1}H NMR (600 MHz, MeCN-d_3, 23 °C, δ): 6.54 (s, 6H), 6.49 (d, J = 7.6 Hz, 2H), 5.59 (d, J = 7.6 Hz, 2H).

^{13}C NMR (151 MHz, MeCN-d_3, 23 °C, δ): 164.4, 123.7 (q, J = 274.1f Hz), 93.1, 92.9 (q, J = 2.3 Hz), 89.4 (q, J = 38.1 Hz), 78.6.

^{19}F NMR (565 MHz, MeCN-d_3, 23 °C, δ): −61.91, −79.35.

HRMS ESI (m/z) calculated for $C_{13}H_{10}F_3O_1Ru_1$ [M]$^+$, 340.9722; found, 340.9723, deviation: −0.4 ppm.

Elemental analysis calculated for $C_{14}H_{10}F_6O_4Ru_1S_1$: C, 34.36; H, 2.06; S, 6.55; F, 23.29; O, 13.08; Ru, 20.65; found: C, 34.47; H, 2.11; S, 6.48; F, 23.07; Ru, 20.44.

Melting point: 239 °C (decomposition).

[η^6-Benzene-η^5-(2,6-dibromo-1-phenoxo)Ru](OTf) (1k)

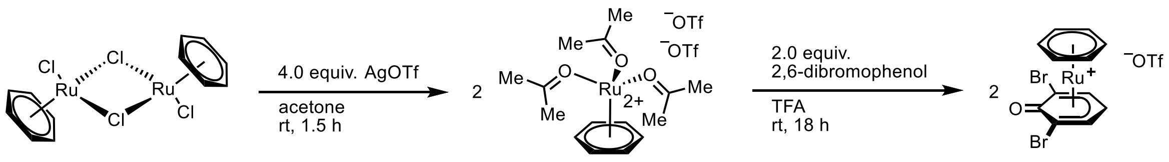

1k, 43%

A 20 mL borosilicate vial equipped with a Teflon-coated magnetic stirring bar was charged with (benzene)ruthenium dichloride dimer (0.10 g, 0.20 mmol, 1.0 equiv.) and AgOTf (0.21 g, 0.80 mmol, 4.0 equiv.). The vial was connected via a vial adapter to a Schlenk line and was evacuated and purged with argon three times. Anhydrous acetone (5.0 mL, c = 40 mM) was added, the vial covered with aluminum foil and the suspension stirred at 23 °C for 1.5 h under an argon atmosphere. Afterwards, the stirring bar was removed, the vial closed with a Teflon-lined screw cap and centrifuged for 6 min at 3000 rpm. Meanwhile, 2,6-dibromophenol (0.10 g, 0.40 mmol, 2.0 equiv.) was added to a second 20 mL borosilicate vial equipped with a Teflon-coated magnetic stirring bar and a vial adapter. The vial adapter was evacuated and purged with argon three times, after which the supernatant from the first vial was added. The solvent was evaporated under high vacuum and TFA (5.0 mL, c = 80 mM) added under an argon atmosphere. The resulting solution was stirred at 23 °C for 16 h. The vial was then cooled in ice water and diethyl ether (15 mL) was added to the vial to precipitate a colorless solid. The solid was filtered off, washed with diethyl ether (3 × 10 mL), and dried under high vacuum. The crude complex was recrystallized by heating an Et_2O (10 mL) suspension of the complex under reflux and adding MeOH (~1.5 mL) dropwise until a solution was obtained. The solution was subsequently stored in the freezer for 3 days to give light-yellow needles. The needles were filtered off, washed with Et_2O (10 mL) and dried in high vacuum to afford **1k** (0.11 g, 0.17 mmol, 43%) as a light-yellow solid.

NMR Spectroscopy:

^{1}H NMR (600 MHz, MeCN-d_3, 23 °C, δ): 6.69 (d, *J* = 5.5 Hz, 2H), 6.44 (s, 6H), 6.07 (t, J = 5.5 Hz, 1H).

^{13}C NMR (151 MHz, MeCN-d_3, 23 °C, δ): 158.5, 122.2 (q, *J* = 321.0 Hz), 98.0, 95.3, 86.5, 82.9.

^{19}F NMR (565 MHz, MeCN-d_3, 23 °C, δ): −79.35.

HRMS ESI (m/z) calculated for $C_{12}H_9Br_2O_1Ru_1$ $[M]^+$, 428.8058; found, 428.8053, deviation: +1.1 ppm.

Melting point: 198 °C (decomposition).

[η^6-Benzene-η^5-(4-(N,N-dimethylamino)-1-phenoxo)Ru](OTf) (1l)

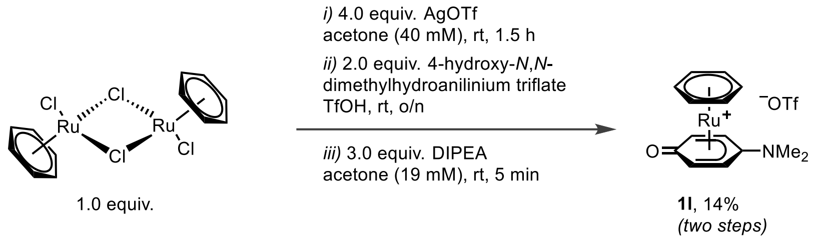

Note: For phenol ligands containing amino groups, protonation of the amine prior to complexation is required as described in the following procedure.

A 20 mL borosilicate vial equipped with a Teflon-coated magnetic stirring bar was charged with (benzene)ruthenium dichloride dimer (0.10 g, 0.20 mmol, 1.0 equiv.) and AgOTf (0.21 g, 0.80 mmol, 4.0 equiv.). The vial was connected via a vial adapter to a Schlenk line and was evacuated and purged with argon three times. Subsequently, anhydrous acetone (5.0 mL, *c* = 40 mM) was added, the vial was covered with aluminum foil and the suspension was stirred at 23 °C for 1.5 h under an argon atmosphere. Then, the orange reaction mixture was centrifuged at 3000 rpm for 8 min. Meanwhile, a second 20 mL borosilicate vial equipped with a Teflon-coated magnetic stir bar and a vial adapter was charged with 4-*N*,*N*-dimethylaminophenol (55 mg, 0.40 mmol, 2.0 equiv.). Under an argon atmosphere, TfOH (2.0 ml) was added to the phenol and the mixture stirred for 2 min. In parallel, a third 20 mL borosilicate vial equipped with a Teflon-coated magnetic stir bar and a vial adapter was evacuated and purged with argon three times. The orange supernatant from the first vial was added to the empty third vial and the solvent was removed under high vacuum, while stirring. Subsequently, TfOH (2.0 mL) was added under an argon atmosphere. Then, the aminophenol solution in TfOH from the second vial was added and the resulting solution was stirred at 23 °C for 16 h. Afterwards, diethyl ether (15 mL) was added at 0 °C to precipitate a white solid. The solid was filtered off over a G4 glass frit, washed with diethyl ether (3 × 15 mL), and dried under high vacuum to afford the Ru(II) precatalyst (70 mg, 90 µmol, 23%) as a yellowish solid. In case no precipitation was observed, the TfOH was removed by vacuum distillation and the precatalyst was precipitated using diethyl ether from a methanol solution.

The corresponding Ru(II) pre-catalyst (70 mg, 90 µmol, 1.0 equiv.) was transferred to a 20 mL borosilicate vial equipped with a Teflon-coated magnetic stir bar. After addition of acetone (5.0 mL, *c* = 53 mM) and DIPEA (47 µL, 0.27 mmol, 3.0 equiv.), the resulting solution was stirred at 23 °C for 15 min under ambient atmosphere. Then, diethyl ether (15 mL) was added to precipitate a solid. The solid was filtered off over a G4 glass frit, washed with diethyl ether (3 × 15 mL), and dried under high vacuum to afford Ru(II) complex **1l** (25 mg, 79 µmol, 59%) as a yellowish solid.

NMR Spectroscopy:

^{1}**H NMR** (600 MHz, methanol-d_4, 23 °C, δ): 6.56 (s, 6H), 5.96 (d, *J* = 7.4 Hz, 2H), 5.77 (d, *J* = 7.4 Hz, 2H), 3.04 (s, 6H).

^{13}**C NMR** (151 MHz, methanol-d_4, 23 °C, δ): 152.4, 133.8, 121.8 (q, *J* = 318.5 Hz), 92.2, 77.0, 70.3,

40.4.

^{19}F NMR (565 MHz, methanol-d_4, 23 °C, δ): −80.04.

HRMS ESI (m/z) calculated for $C_{14}H_{16}N_1O_1Ru_1$ $[M]^+$, 316.0270; found, 316.0270, deviation: −0.1 ppm.

Melting point: 54 °C (decomposition).

[η^6-Benzene-η^5-(1-phenoxo-4-phenyl)Ru](OTf) (SI-1)

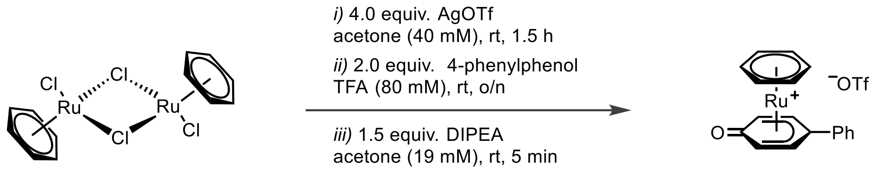

SI-1, 84%
(two steps)

A 20 mL borosilicate vial equipped with a Teflon-coated magnetic stirring bar was charged with (benzene)ruthenium dichloride dimer (0.10 g, 0.20 mmol, 1.0 equiv.) and AgOTf (0.21 g, 0.80 mmol, 4.0 equiv.). The vial was connected via a vial adapter to a Schlenk line and was evacuated and purged with argon three times. Subsequently, anhydrous acetone (5.0 mL, *c* = 40 mM) was added, the vial was covered with aluminum foil and the suspension was stirred at 23 °C for 1.5 h under an argon atmosphere. Then, the orange reaction mixture was centrifuged at 3000 rpm for 8 min. Meanwhile, a second 20 mL borosilicate vial equipped with a Teflon-coated magnetic stir bar and a vial adapter was charged with 4-phenylphenol (68 mg, 0.40 mmol, 2.0 equiv.). Under an argon atmosphere, the orange supernatant from the first vial was added and the solvent was removed under high vacuum, while stirring. Afterwards, trifluoroacetic acid (5.0 mL, *c* = 80 mM, TFA) was added under an argon atmosphere, and the resulting solution was stirred at 23 °C for 16 h. Then, diethyl ether (15 mL) was added at 0 °C to precipitate a light-yellow solid. The solid was filtered off over a G4 glass frit, washed with diethyl ether (3 × 15 mL), and dried under high vacuum to afford the Ru(II) pre-catalyst (0.23 g, 0.36 mmol, 90%) as a light-yellow solid.

The corresponding Ru(II) pre-catalyst (37 mg, 58 µmol, 1.0 equiv.) was transferred to a 20 mL borosilicate vial equipped with a Teflon-coated magnetic stir bar. After addition of acetone (3.0 mL, *c* = 19 mM) and DIPEA (15 µL, 11 mg, 87 µmol, 1.5 equiv.), the resulting solution was stirred at 23 °C for 5 min under ambient atmosphere. Then, diethyl ether (15 mL) was added to precipitate a solid. The solid was filtered off over a G4 glass frit, washed with diethyl ether (3 × 15 mL), and dried under high vacuum to afford Ru(II) complex **SI-1** (27 mg, 54 µmol, 93%) as an orange solid.

NMR Spectroscopy:

^{1}H NMR (600 MHz, MeCN-d_3, 23 °C, δ): 7.72 – 7.70 (m, 2H), 7.58 – 7.51 (m, 3H), 6.56 (d, *J* = 7.5 Hz, 2H), 6.34 (s, 6H), 5.59 (d, *J* = 7.5 Hz, 2H).

^{13}C NMR (151 MHz, MeCN-d_3, 23 °C, δ): 164.2, 133.6, 131.6, 130.4, 129.1, 122.2 (q, *J* = 321.6 Hz), 102.5, 93.7, 92.3, 78.4.

^{19}F NMR (470 MHz, MeCN-d_3, 23 °C, δ): −79.37.

HRMS ESI (m/z) calculated for $C_{18}H_{15}O_1Ru_1$ $[M]^+$, 349.0161; found, 349.0165; deviation: −1.2 ppm.

Melting point: 174 °C (decomposition).

[η^6-Benzene-η^5-(3,5-dimethoxy-1-phenoxo)Ru](OTf) (SI-2)

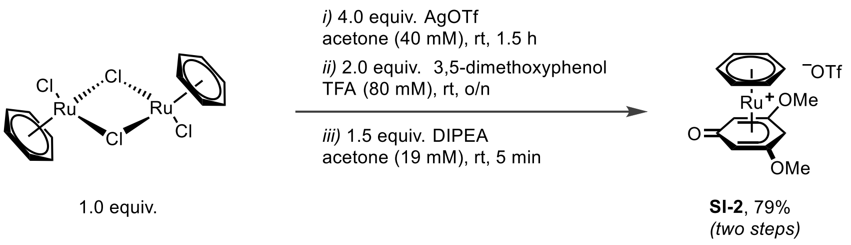

A 20 mL borosilicate vial equipped with a Teflon-coated magnetic stirring bar was charged with (benzene)ruthenium dichloride dimer (0.10 g, 0.20 mmol, 1.0 equiv.) and AgOTf (0.21 g, 0.80 mmol, 4.0 equiv.). The vial was connected via a vial adapter to a Schlenk line and was evacuated and purged with argon three times. Subsequently, anhydrous acetone (5.0 mL, *c* = 40 mM) was added, the vial was covered with aluminum foil and the suspension was stirred at 23 °C for 1.5 h under an argon atmosphere. Then, the orange reaction mixture was centrifuged at 3000 rpm for 8 min. Meanwhile, a second 20 mL borosilicate vial equipped with a Teflon-coated magnetic stir bar and a vial adapter was charged with 3,5-dimethoxyphenol (62 mg, 0.40 mmol, 2.0 equiv.). Under an argon atmosphere, the orange supernatant from the first vial was added and the solvent was removed under high vacuum, while stirring. Afterwards, trifluoroacetic acid (5.0 mL, *c* = 80 mM, TFA) was added under an argon atmosphere, and the resulting solution was stirred at 23 °C for 16 h. Then, diethyl ether (15 mL) was added at 0 °C to precipitate a light-yellow solid. The solid was filtered off over a G4 glass frit, washed with diethyl ether (3 × 15 mL), and dried under high vacuum to afford the Ru(II) pre-catalyst (0.22 g, 0.36 mmol, 87%) as a light-yellow solid.

The corresponding Ru(II) pre-catalyst (0.20 g, 0.31 mmol, 1.0 equiv.) was transferred to a 20 mL borosilicate vial equipped with a Teflon-coated magnetic stir bar. After addition of acetone (6.0 mL, *c* = 52 mM) and DIPEA (82 µL, 61 mg, 0.47 mmol, 1.5 equiv.), the resulting solution was stirred at 23 °C for 5 min under ambient atmosphere. Then, diethyl ether (15 mL) was added to precipitate a solid. The solid was filtered off over a G4 glass frit, washed with diethyl ether (3 × 15 mL), and dried under high vacuum to afford Ru(II) complex **SI-2** (0.14 g, 0.28 mmol, 91%) as a light-yellow solid.

NMR Spectroscopy:

^{1}**H NMR** (600 MHz, MeCN-d_3, 23 °C, δ): 6.31 (t, *J* = 1.5 Hz, 1H), 6.30 (s, 6H), 5.50 (d, *J* = 1.5 Hz, 2H), 3.71 (s, 6H).

^{13}**C NMR** (151 MHz, MeCN-d_3, 23 °C, δ): 161.3, 141.6, 122.2 (q, *J* = 320.7 Hz), 90.2, 65.1, 62.5, 58.4.

^{19}**F NMR** (470 MHz, MeCN-d_3, 23 °C, δ): −79.33.

HRMS ESI (m/z) calculated for $C_{14}H_{15}O_3Ru_1$ $[M]^+$, 333.0059; found, 333.0061; deviation: −0.6 ppm.

Melting point: 241 °C (decomposition).

[η^6-Benzene-η^5-(1-phenoxo-3,4,5-trimethyl)Ru](OTf) (SI-3)

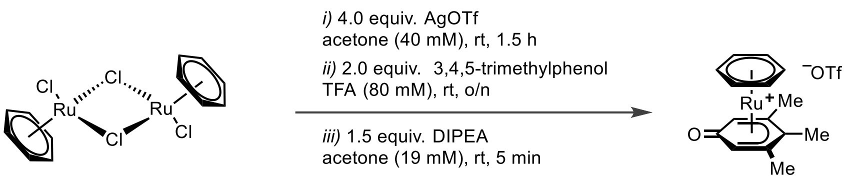

1.0 equiv.

SI-3, 63% *(two steps)*

A 20 mL borosilicate vial equipped with a Teflon-coated magnetic stirring bar was charged with (benzene)ruthenium dichloride dimer (0.10 g, 0.20 mmol, 1.0 equiv.) and AgOTf (0.21 g, 0.80 mmol, 4.0 equiv.). The vial was connected via a vial adapter to a Schlenk line and was evacuated and purged with argon three times. Subsequently, anhydrous acetone (5.0 mL, *c* = 40 mM) was added, the vial was covered with aluminum foil and the suspension was stirred at 23 °C for 1.5 h under an argon atmosphere. Then, the orange reaction mixture was centrifuged at 3000 rpm for 8 min. Meanwhile, a second 20 mL borosilicate vial equipped with a Teflon-coated magnetic stir bar and a vial adapter was charged with 3,4,5-trimethylphenol (55 mg, 0.40 mmol, 2.0 equiv.). Under an argon atmosphere, the orange supernatant from the first vial was added and the solvent was removed under high vacuum, while stirring. Afterwards, trifluoroacetic acid (5.0 mL, *c* = 80 mM, TFA) was added under an argon atmosphere, and the resulting solution was stirred at 23 °C for 16 h. Then, diethyl ether (15 mL) was added at 0 °C to precipitate a grey solid. The solid was filtered off over a G4 glass frit, washed with diethyl ether (3 × 15 mL), and dried under high vacuum to afford the Ru(II) pre-catalyst (0.19 g, 0.30 mmol, 75%) as a grey solid.

The corresponding Ru(II) pre-catalyst (0.16 g, 0.26 mmol, 1.0 equiv.) was transferred to a 20 mL borosilicate vial equipped with a Teflon-coated magnetic stir bar. After addition of acetone (4.5 mL, *c* = 58 mM) and DIPEA (68 µL, 50 mg, 0.39 mmol, 1.5 equiv.), the resulting solution was stirred at 23 °C for 5 min under ambient atmosphere. Then, diethyl ether (15 mL) was added to precipitate a solid. The solid was filtered off over a G4 glass frit, washed with diethyl ether (3 × 15 mL), and dried under high vacuum to afford Ru(II) complex **SI-3** (0.10 g, 0.22 mmol, 84%) as a light-brown solid.

NMR Spectroscopy:

^{1}H NMR (600 MHz, MeCN-d_3, 23 °C, δ): 6.25 (s, 6H), 5.47 (s, 2H), 2.27 (s, 3H), 2.23 (s, 6H).

^{13}C NMR (151 MHz, MeCN-d_3, 23 °C, δ): 163.5, 122.2 (q, J = 320.9 Hz), 111.8, 100.6, 92.0, 80.5, 10.1, 15.0.

^{19}F NMR (470 MHz, MeCN-d_3, 23 °C, δ): −79.34.

HRMS ESI (m/z) calculated for $C_{15}H_{17}O_1Ru_1$ $[M]^+$, 315.0317; found, 315.0321; deviation: −1.1 ppm.

Melting point: 250 °C (decomposition).

[η^6-Benzene-η^5-(1-phenoxo-2,4,6-trimethyl)Ru](OTf) (SI-4)

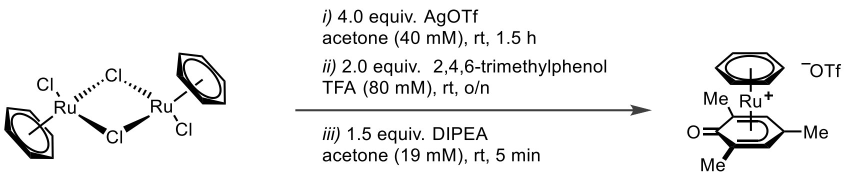

1.0 equiv.

SI-4, 72% *(two steps)*

A 20 mL borosilicate vial equipped with a Teflon-coated magnetic stirring bar was charged with (benzene)ruthenium dichloride dimer (0.10 g, 0.20 mmol, 1.0 equiv.) and AgOTf (0.21 g, 0.80 mmol, 4.0 equiv.). The vial was connected via a vial adapter to a Schlenk line and was evacuated and purged with argon three times. Subsequently, anhydrous acetone (5.0 mL, *c* = 40 mM) was added, the vial was covered with aluminum foil and the suspension was stirred at 23 °C for 1.5 h under an argon atmosphere. Then, the orange reaction mixture was centrifuged at 3000 rpm for 8 min. Meanwhile, a second 20 mL borosilicate vial equipped with a Teflon-coated magnetic stir bar and a vial adapter was charged with 2,4,6-trimethylphenol (55 mg, 0.40 mmol, 2.0 equiv.). Under an argon atmosphere, the orange supernatant from the first vial was added and the solvent was removed under high vacuum, while stirring. Afterwards, trifluoroacetic acid (5.0 mL, *c* = 80 mM, TFA) was added under an argon atmosphere, and the resulting solution was stirred at 23 °C for 16 h. Then, diethyl ether (15 mL) was added at 0 °C to precipitate a white solid. The solid was filtered off over a G4 glass frit, washed with diethyl ether (3 × 15 mL), and dried under high vacuum to afford the Ru(II) precatalyst (0.19 g, 0.31 mmol, 78%) as a white solid.

The corresponding Ru(II) pre-catalyst (0.16 g, 0.27 mmol, 1.0 equiv.) was transferred to a 20 mL borosilicate vial equipped with a Teflon-coated magnetic stir bar. After addition of acetone (5.0 mL, *c* = 53 mM) and DIPEA (70 µL, 52 mg, 0.40 mmol, 1.5 equiv.), the resulting solution was stirred at 23 °C for 5 min under ambient atmosphere. Then, diethyl ether (15 mL) was added to precipitate a solid. The solid was filtered off over a G4 glass frit, washed with diethyl ether (3 × 15 mL), and dried under high vacuum to afford Ru(II) complex **SI-4** (0.11 g, 0.25 mmol, 92%) as a beige solid.

NMR Spectroscopy:

^{1}H NMR (600 MHz, methanol-d_4, 23 °C, δ): 6.38 (s, 6H), 6.38 (s, 2H), 2.26 (s, 3H), 2.09 (s, 6H).

^{13}C NMR (151 MHz, methanol-d_4, 23 °C, δ): 163.3, 121.8 (q, J = 318.6 Hz), 99.6, 96.8, 96.1, 92.8, 19.1, 16.9.

^{19}F NMR (470 MHz, methanol-d_4, 23 °C, δ): −80.0.

HRMS ESI (m/z) calculated for $C_{15}H_{17}O_1Ru_1$ $[M]^+$, 315.0317; found, 315.0318; deviation: −0.3 ppm.

Melting point: 200 °C (decomposition).

[η^6-Benzene-η^5-(2,6-dimethoxy-1-phenoxo)Ru](OTf) (SI-5)

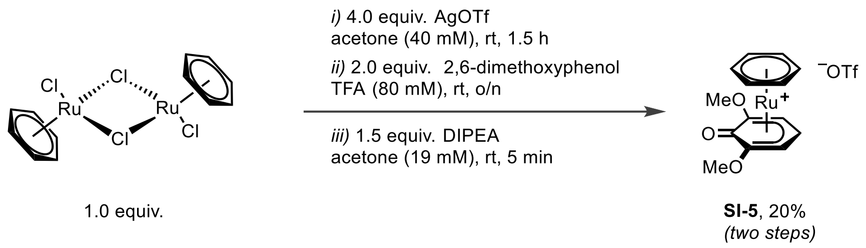

A 20 mL borosilicate vial equipped with a Teflon-coated magnetic stirring bar was charged with (benzene)ruthenium dichloride dimer (0.10 g, 0.20 mmol, 1.0 equiv.) and AgOTf (0.21 g, 0.80 mmol, 4.0 equiv.). The vial was connected via a vial adapter to a Schlenk line and was evacuated and purged with argon three times. Subsequently, anhydrous acetone (5.0 mL, *c* = 40 mM) was added, the vial was covered with aluminum foil and the suspension was stirred at 23 °C for 1.5 h under an argon atmosphere. Then, the orange reaction mixture was centrifuged at 3000 rpm for 8 min. Meanwhile, a second 20 mL borosilicate vial equipped with a Teflon-coated magnetic stir bar and a vial adapter was charged with 2,6-dimethoxyphenol (62 mg, 0.40 mmol, 2.0 equiv.). Under an argon atmosphere, the orange supernatant from the first vial was added and the solvent was removed under high vacuum, while stirring. Afterwards, trifluoroacetic acid (5.0 mL, *c* = 80 mM, TFA) was added under an argon atmosphere, and the resulting solution was stirred at 23 °C for 16 h. Then, diethyl ether (15 mL) was added at 0 °C to precipitate a white solid. The solid was filtered off over a G4 glass frit, washed with diethyl ether (3 × 15 mL), and dried under high vacuum to afford the Ru(II) precatalyst (0.13 g, 0.20 mmol, 50%) as a white solid.

The corresponding Ru(II) pre-catalyst (0.13 g, 0.2 mmol, 1.0 equiv.) was transferred to a 20 mL borosilicate vial equipped with a Teflon-coated magnetic stir bar. After addition of acetone (5.0 mL) and DIPEA (70 µL, 52 mg, 0.40 mmol, 2.0 equiv.), the resulting solution was stirred at 23 °C for 5 min under ambient atmosphere. Then, diethyl ether (15 mL) was added to precipitate a solid. The solid was filtered off over a G4 glass frit, washed with diethyl ether (3 × 15 mL), and dried under high vacuum to afford Ru(II) complex **SI-5** (39 mg, 80 µmol, 40%) as a beige solid.

NMR Spectroscopy:

> **^{1}H NMR** (600 MHz, methanol-*d*$_4$, 23 °C, δ): 6.48 (s, 6H), 6.34 (d, *J* = 5.9 Hz, 2H), 5.85 (t, *J* = 5.8 Hz, 2H), 3.90 (s, 6H).

> **^{13}C NMR** (151 MHz, methanol-*d*$_4$, 23 °C, δ): 150.4, 131.8, 128.8 (q, *J* = 318.5 Hz), 91.1, 76.5, 75.0, 58.3.

> **^{19}F NMR** (565 MHz, methanol-*d*$_4$, 23 °C, δ): −80.08.

HRMS ESI (m/z) calculated for $C_{14}H_{15}O_3Ru_1$ [M]$^+$, 333.0059; found, 333.0060; deviation: −0.3 ppm.

Melting point: 208 °C (decomposition).

[η^6-Benzene-η^5-(4-*t*Bu-1-phenoxo)Ru](OTf) (SI-6)

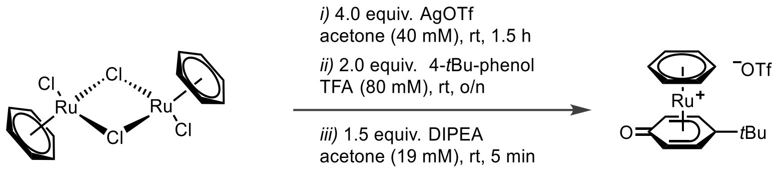

1.0 equiv. **SI-6**, 41% *(two steps)*

A 20 mL borosilicate vial equipped with a Teflon-coated magnetic stirring bar was charged with (benzene)ruthenium dichloride dimer (0.10 g, 0.20 mmol, 1.0 equiv.) and AgOTf (0.21 g, 0.80 mmol, 4.0 equiv.). The vial was connected via a vial adapter to a Schlenk line and was evacuated and purged with argon three times. Subsequently, anhydrous acetone (5.0 mL, *c* = 40 mM) was added, the vial was covered with aluminum foil and the suspension was stirred at 23 °C for 1.5 h under an argon atmosphere. Then, the orange reaction mixture was centrifuged at 3000 rpm for 8 min. Meanwhile, a second 20 mL borosilicate vial equipped with a Teflon-coated magnetic stir bar and a vial adapter was charged with 4-*tert*-butylphenol (60 mg, 0.40 mmol, 2.0 equiv.). Under an argon atmosphere, the orange supernatant from the first vial was added and the solvent was removed under high vacuum, while stirring. Afterwards, trifluoroacetic acid (5.0 mL, *c* = 80 mM, TFA) was added under an argon atmosphere, and the resulting solution was stirred at 23 °C for 16 h. Then, diethyl ether (15 mL) was added at 0 °C to precipitate a white solid. The solid was filtered off over a G4 glass frit, washed with diethyl ether (3 × 15 mL), and dried under high vacuum to afford the Ru(II) precatalyst (0.12 g, 0.20 mmol, 50%) as a white solid.

The corresponding Ru(II) pre-catalyst (0.12 g, 0.20 mmol, 1.0 equiv.) was transferred to a 20 mL borosilicate vial equipped with a Teflon-coated magnetic stir bar. After addition of acetone (5.0 mL) and DIPEA (69 µL, 51 mg, 0.39 mmol, 2.0 equiv.), the resulting solution was stirred at 23 °C for 5 min under ambient atmosphere. Then, diethyl ether (15 mL) was added to precipitate a solid. The solid was filtered off over a G4 glass frit, washed with diethyl ether (3 × 15 mL), and dried under high vacuum to afford Ru(II) complex **SI-6** (77 mg, 0.16 mmol, 81%) as a beige solid.

NMR Spectroscopy:

> **^{1}H NMR** (600 MHz, methanol-*d*$_4$, 23 °C, δ): 6.62 (s, 6H), 6.40 (d, *J* = 7.4 Hz, 2H), 5.66 (d, *J* = 7.4 Hz, 2H), 1.38 (s, 9H).
>
> **^{13}C NMR** (151 MHz, methanol-*d*$_4$, 23 °C, δ): 163.5, 121.8 (q, *J* = 318.4 Hz), 117.6, 92.5, 92.3, 79.1, 34.9, 30.7.
>
> **^{19}F NMR** (565 MHz, methanol-*d*$_4$, 23 °C, δ): −80.07.

HRMS ESI (m/z) calculated for $C_{16}H_{19}O_1Ru_1$ [M]$^+$, 329.0474; found, 329.0476; deviation: −0.7 ppm.

Melting point: 206 °C (decomposition).

[η^6-Benzene-η^5-(4-amino-1-phenoxo)Ru](OTf) (SI-7)

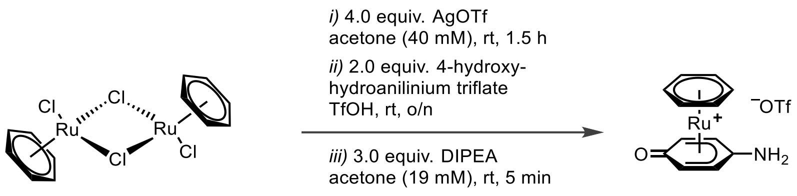

SI-7, 37%
(two steps)

Note: For phenol ligands containing amino groups, protonation of the amine prior to complexation is required as described in the following procedure.

A 20 mL borosilicate vial equipped with a Teflon-coated magnetic stirring bar was charged with (benzene)ruthenium dichloride dimer (0.10 g, 0.20 mmol, 1.0 equiv.) and AgOTf (0.21 g, 0.80 mmol, 4.0 equiv.). The vial was connected via a vial adapter to a Schlenk line and was evacuated and purged with argon three times. Subsequently, anhydrous acetone (5.0 mL, c = 40 mM) was added, the vial was covered with aluminum foil and the suspension was stirred at 23 °C for 1.5 h under an argon atmosphere. Then, the orange reaction mixture was centrifuged at 3000 rpm for 8 min. Meanwhile, a second 20 mL borosilicate vial equipped with a Teflon-coated magnetic stir bar and a vial adapter was charged with 4-aminophenol (44 mg, 0.40 mmol, 2.0 equiv.). Under an argon atmosphere, TfOH (2.0 ml) was added to the phenol and the mixture stirred for 2 min. In parallel, a third 20 mL borosilicate vial equipped with a Teflon-coated magnetic stir bar and a vial adapter was evacuated and purged with argon three times. The orange supernatant from the first vial was added to the empty third vial and the solvent was removed under high vacuum, while stirring. Subsequently, TfOH (2.0 mL) was added under an argon atmosphere. Then, the aminophenol solution in TfOH from the second vial was added and the resulting solution was stirred at 23 °C for 16 h. Afterwards, diethyl ether (15 mL) was added at 0 °C to precipitate a white solid. The solid was filtered off over a G4 glass frit, washed with diethyl ether (3 × 15 mL), and dried under high vacuum to afford the Ru(II) precatalyst (0.13 g, 0.18 mmol, 44%) as a white solid. In case no precipitation was observed, the TfOH was removed by vacuum distillation and the precatalyst was precipitated using diethyl ether from a methanol solution.

The corresponding Ru(II) pre-catalyst (0.13 g, 0.18 mmol, 1.0 equiv.) was transferred to a 20 mL borosilicate vial equipped with a Teflon-coated magnetic stir bar. After addition of acetone (5.0 mL) and DIPEA (94 µL, 0.54 mmol, 3.0 equiv.), the resulting solution was stirred at 23 °C for 15 min under ambient atmosphere. Then, diethyl ether (15 mL) was added to precipitate a solid. The solid was filtered off over a G4 glass frit, washed with diethyl ether (3 × 15 mL), and dried under high vacuum to afford Ru(II) complex **SI-7** (50 mg, 0.15 mmol, 84%) as a crème-white solid.

NMR Spectroscopy:

^{1}**H NMR** (600 MHz, methanol-d_4, 23 °C, δ): 6.39 (s, 6H), 5.87 (d, J = 7.2 Hz, 2H), 5.46 (d, J = 7.2 Hz, 2H).

^{13}**C NMR** (151 MHz, methanol-d_4, 23 °C, δ): 158.7, 132.0, 121.8 (q, J = 318.4 Hz), 92.1, 76.9, 74.3.

^{19}F NMR (565 MHz, methanol-*d*$_4$, 23 °C, δ): −80.10.

HRMS ESI (m/z) calculated for $C_{12}H_{12}N_1O_1Ru_1$ [M]$^+$, 287.9957; found, 287.9959, deviation: −0.6 ppm.

Melting point: 198 °C (decomposition).

[η^6-Ethylbenzoate-η^5-(2,6-dichloro-1-phenoxo)Ru](OTf) (SI-8)

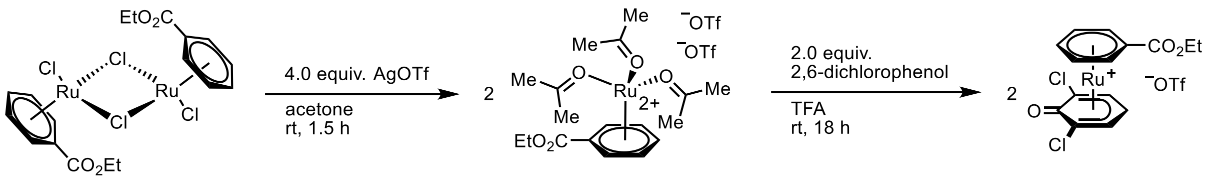

SI-8, 50%

A 20 mL borosilicate vial equipped with a Teflon-coated magnetic stirring bar was charged with (ethylbenzoate)ruthenium dichloride dimer (0.13 g, 0.20 mmol, 1.0 equiv.) and AgOTf (0.21 g, 0.80 mmol, 4.0 equiv.). The vial was connected via a vial adapter to a Schlenk line and was evacuated and purged with argon three times. Anhydrous acetone (5.0 mL, c = 40 mM) was added, the vial covered with aluminum foil and the suspension stirred at 23 °C for 1.5 h under an argon atmosphere. Afterwards, the stirring bar was removed, the vial closed with a Teflon-lined screw cap and centrifuged for 6 min at 3000 rpm. Meanwhile, 2,6-dichlorophenol (65 mg, 0.40 mmol, 2.0 equiv.) was added to a second 20 mL borosilicate vial equipped with a Teflon-coated magnetic stirring bar and a vial adapter. The vial adapter was evacuated and purged with argon three times, after which the supernatant from the first vial was added. The solvent was evaporated under high vacuum and TFA (5.0 mL) added under an argon atmosphere. The resulting solution was stirred at 23 °C for 16 h. The vial was then cooled in ice water and diethyl ether (15 mL) was added to the vial to precipitate a colorless solid. The solid was filtered off, washed with diethyl ether (3 × 10 mL), and dried under high vacuum to afford **1g** (94 mg, 0.2 mmol, 50%) as a light-yellow solid.

NMR Spectroscopy:

^{1}H NMR (600 MHz, MeCN-d_3, 23 °C, δ): 6.91–6.66 (m, 2H), 6.64–6.60 (m, 5H), 6.19 (td, J = 5.6 Hz, J = 0.5 Hz 1H), 4.46 (q, J = 7.1 Hz, 2H), 1.43 (t, J = 7.1 Hz, 3H).

^{13}C NMR (151 MHz, MeCN-d_3, 23 °C, δ): 166.2, 156.8, 122.2 (q, J = 320.8 Hz), 101.2, 96.4, 96.3, 96.1, 94.4, 93.9, 82.0, 65.1, 14.5.

^{19}F NMR (565 MHz, MeCN-d_3, 23 °C, δ): –79.3.

HRMS ESI (m/z) calculated for $C_{15}H_{13}Cl_2O_3Ru_1$ [M]$^+$, 412.9280; found, 412.9275, deviation: 1.1 ppm.

Melting point: 171 °C.

[η^6-Benzene-η^5-(2-chloro-1-phenoxo)Ru](OTf) (SI-9)

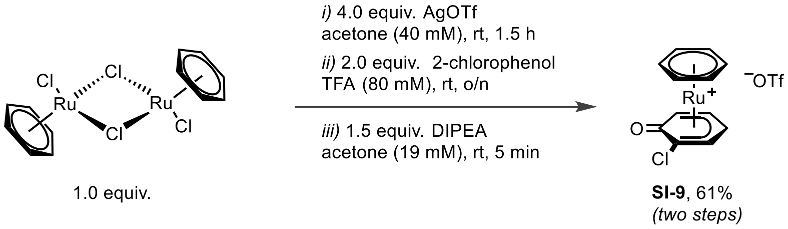

A 20 mL borosilicate vial equipped with a Teflon-coated magnetic stirring bar was charged with (benzene)ruthenium dichloride dimer (0.10 g, 0.20 mmol, 1.0 equiv.) and AgOTf (0.21 g, 0.80 mmol, 4.0 equiv.). The vial was connected via a vial adapter to a Schlenk line and was evacuated and purged with argon three times. Subsequently, anhydrous acetone (5.0 mL, *c* = 40 mM) was added, the vial was covered with aluminum foil and the suspension was stirred at 23 °C for 1.5 h under an argon atmosphere. Then, the orange reaction mixture was centrifuged at 3000 rpm for 8 min. Meanwhile, a second 20 mL borosilicate vial equipped with a Teflon-coated magnetic stir bar and a vial adapter was charged with 4-methoxyphenol (51 mg, 0.40 mmol, 2.0 equiv.). Under an argon atmosphere, the orange supernatant from the first vial was added and the solvent was removed under high vacuum, while stirring. Afterwards, trifluoroacetic acid (5.0 mL, *c* = 80 mM, TFA) was added under an argon atmosphere, and the resulting solution was stirred at 23 °C for 16 h. Then, diethyl ether (15 mL) was added at 0 °C to precipitate a white solid. The solid was filtered off over a G4 glass frit, washed with diethyl ether (3 × 15 mL), and dried under high vacuum to afford the Ru(II) precatalyst (0.53 g, 0.25 mmol, 63%) as a white solid.

The corresponding Ru(II) pre-catalyst (50 mg, 82 µmol, 1.0 equiv.) was transferred to a 20 mL borosilicate vial equipped with a Teflon-coated magnetic stir bar. After addition of acetone (2.0 mL) and DIPEA (22 µL, 0.12 mmol, 1.5 equiv.), the resulting solution was stirred at 23 °C for 5 min under ambient atmosphere. Then, diethyl ether (15 mL) was added to precipitate a solid. The solid was filtered off over a G4 glass frit, washed with diethyl ether (3 × 15 mL), and dried under high vacuum to afford Ru(II) complex **1i** (36 mg, 79 mmol, 96%) as a beige solid.

NMR Spectroscopy:

^{1}**H NMR** (600 MHz, MeCN-d_3, 23 °C, δ): 6.64 (dd, *J* = 5.6, 1.5 Hz, 1H), 6.44 (s, 6H), 6.12 (td, *J* = 5.5, 1.0 Hz, 1H), 6.04 (ddd, *J* = 6.9, 5.3, 1.5 Hz, 1H), 5.75 (dd, *J* = 7.0, 1.0 Hz, 1H).

^{13}**C NMR** (151 MHz, MeCN-d_3, 23 °C, δ): 160.2, 122.2 (q, *J* = 320.8 Hz), 100.3, 95.8, 94.5, 93.3, 82.6, 79.2.

^{19}**F NMR** (565 MHz, MeCN-d_3, 23 °C, δ): −79.25.

HRMS ESI (m/z) calculated for $C_{12}H_{10}Cl_1O_1Ru_1$ [M]$^+$, 306.9458; found, 306.9457, deviation: +0.5 ppm.

Melting point: 164–166 °C (decomposition).

Arene Exchange Study

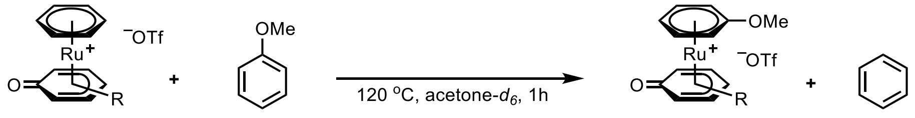

I (10.0 eq.) II

A 5 mm J-Young NMR tube was charged with ruthenium phenoxo complexes (**1**) (20 µmol, 1.0 equiv.) and anisole (22 mg, 0.20 mmol, 10 equiv.). Deuterated acetone (0.5 mL, *c* = 40 mM) was added, the tube inverted several times, and the initial ^{1}H NMR spectrum was measured. After the initial measurement, the tube was placed in an 120 ˚C oil bath and was heated for 60 min. Then, the ^{1}H NMR spectrum was measured for determination of the conversion of **I** to **II** and to calculate the rate constant (mol%·s^{-1}) of arene exchange. Additional spectra were measured for several compounds to further analyze the arene exchange properties. The rate constants were determined by plotting of the conversion in mol%, which was extracted from the ^{1}H-NMR integrals, against the reaction time. For a better comparison of the arene exchange properties of different ligands, a relative value was calculated to compare each phenoxo ligand respective to the unsubstituted phenoxo ligand by dividing k_{init}(phenoxo-R$_n$) by k_{init}(phenoxo-H).

Table S1 Arene exchange rates of benzene Ru–phenoxo complexes:

**Compound**	**Ligand**	**k_{init} (Formation of anisole complex) [mol%·h^{-1}]**	**Relative value of arene exchange to unsubstituted phenol**
**SI-4**	2,4,6-trimethylphenol	1.22	0.21
**SI-5**	2,6-dimethoxyphenol	4.40	0.75
**SI-3**	3,4,5-trimethylphenol	4.56	0.77
**SI-6**	4-*t*-butylphenol	4.65	0.79
**1c**	phenol	5.89	1.00
**SI-2**	3,5-dimethoxyphenol	6.16	1.05
**1h**	*p*-cresol	7.85	1.33
**1d**	4-*n*-butylphenol	8.26	1.40
**1e**	2-methoxyethylphenol	9.76	1.66
**1f**	2,6-diiodophenol	11.78	2.00
**1j**	4-trifluoromethylphenol	15.32	2.60
**SI-7**	4-aminophenol	19.57	3.32
**1l**	4-dimethylaminophenol	20.35	3.46
**1i**	4-methoxyphenol	22.23	3.77
**SI-9**	2-chlorphenol	33.03	5.61
**1k**	2,6-dibromophenol	33.05	5.61
**1g**	2,6-dichlorophenol	56.53	9.60
**1a**	2,6-dibromo-4-methoxyphenol	68.75	11.67

Note: For compound **1b** and **1-Cp***, No significant arene exchange was observed. For compound **SI-1**, no meaningful rate could be extracted from the **^{1}H-NMR** spectra, due to overlapping signals.

Arene exchange study for [η^6-benzene-η^5-(2,4,6-trimethyl-1-phenoxo)Ru](OTf) (SI-4)

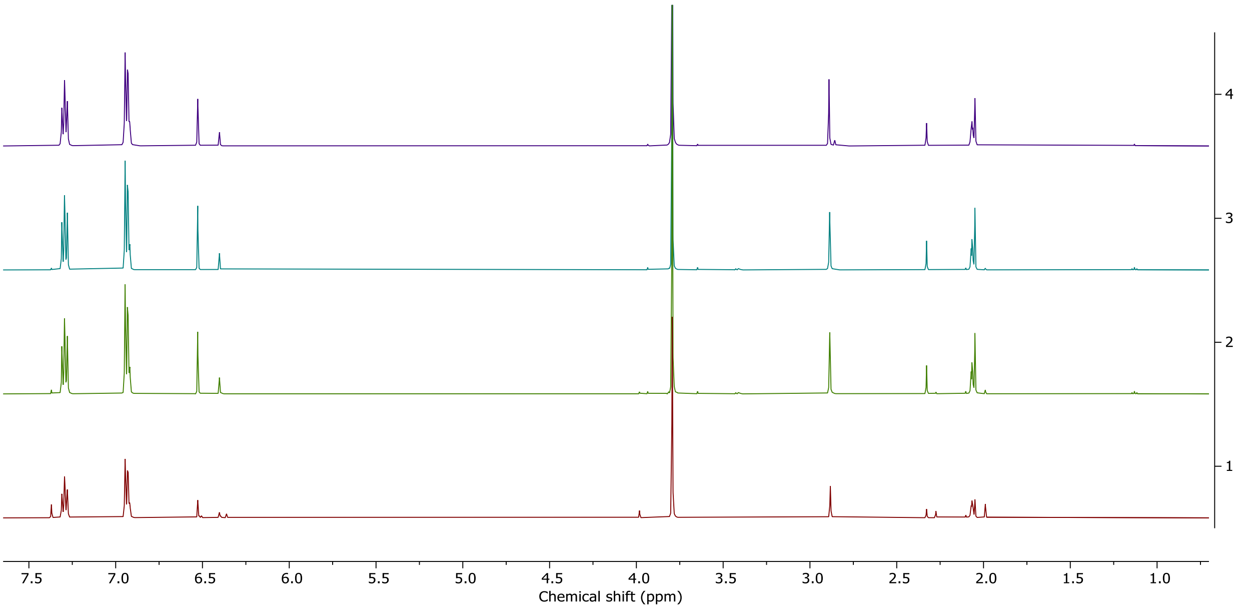

Fig S1: Series of ^{1}H-NMR spectra at 500 MHz, 23 °C, acetone-d_6; 4 = initial spectrum, 3 = 1h at 120 °C, 2 = 2h at 120 °C, 1 = 18h at 120 °C.

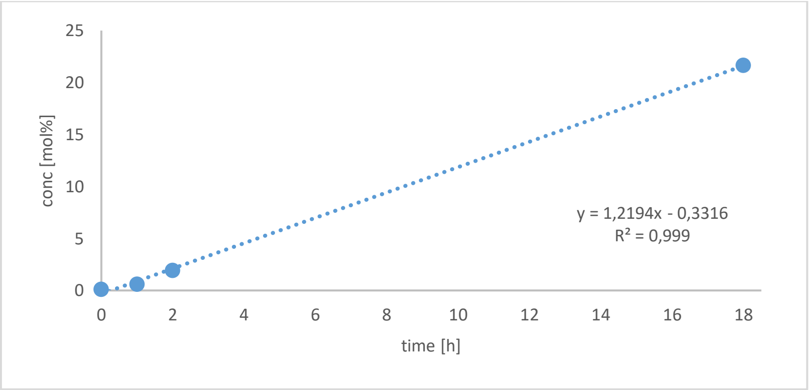

Fig S2: Plot to determine the rate constant of the arene exchange of **SI-4**.

Arene exchange study for [η^6-benzene-η^5-(2,6-dimethoxy-1-phenoxo)Ru](OTf) (SI-5)

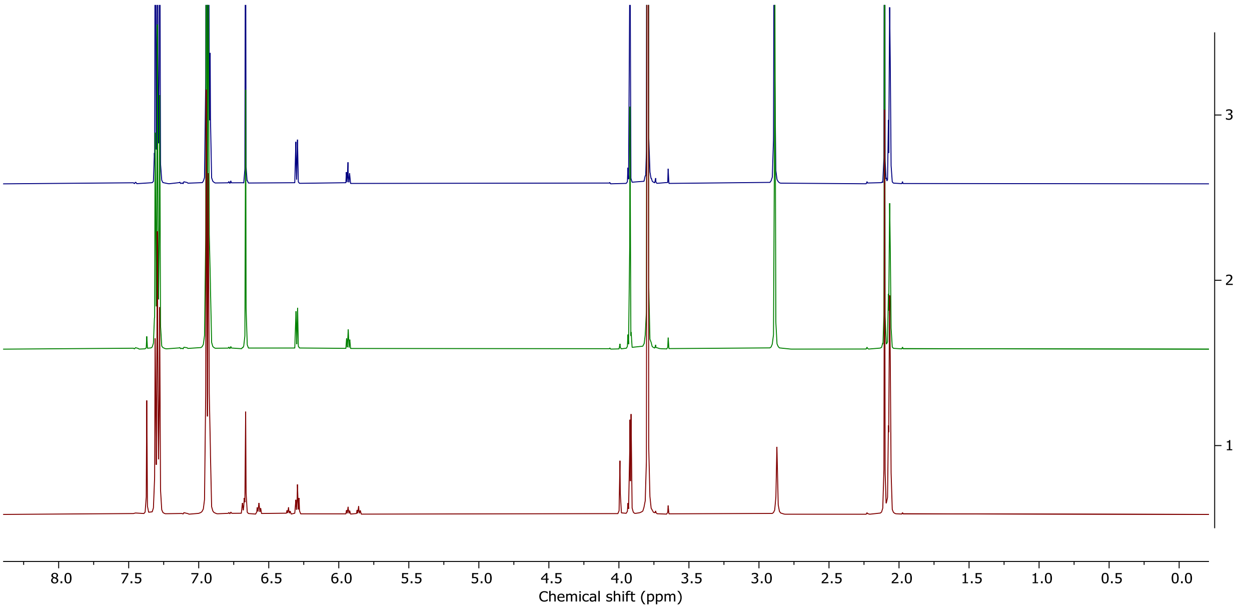

Fig S3: Series of ^{1}H-NMR spectra at 500 MHz, 23 °C, acetone-d_6; 3 = initial spectrum, 2 = 1h at 120 °C, 1 = 18h at 120 °C.

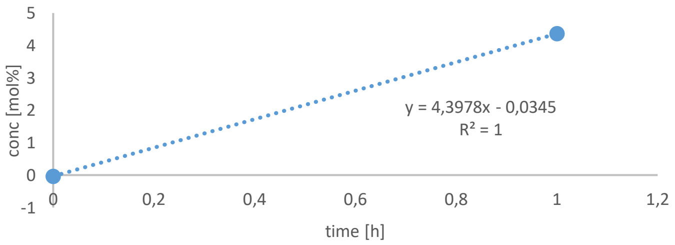

Fig S4: Plot to determine the rate constant of the arene exchange of **SI-5**.

Arene exchange study for [η^6-benzene-η^5-(3,4,5-trimethyl-1-phenoxo)Ru](OTf) (SI-3)

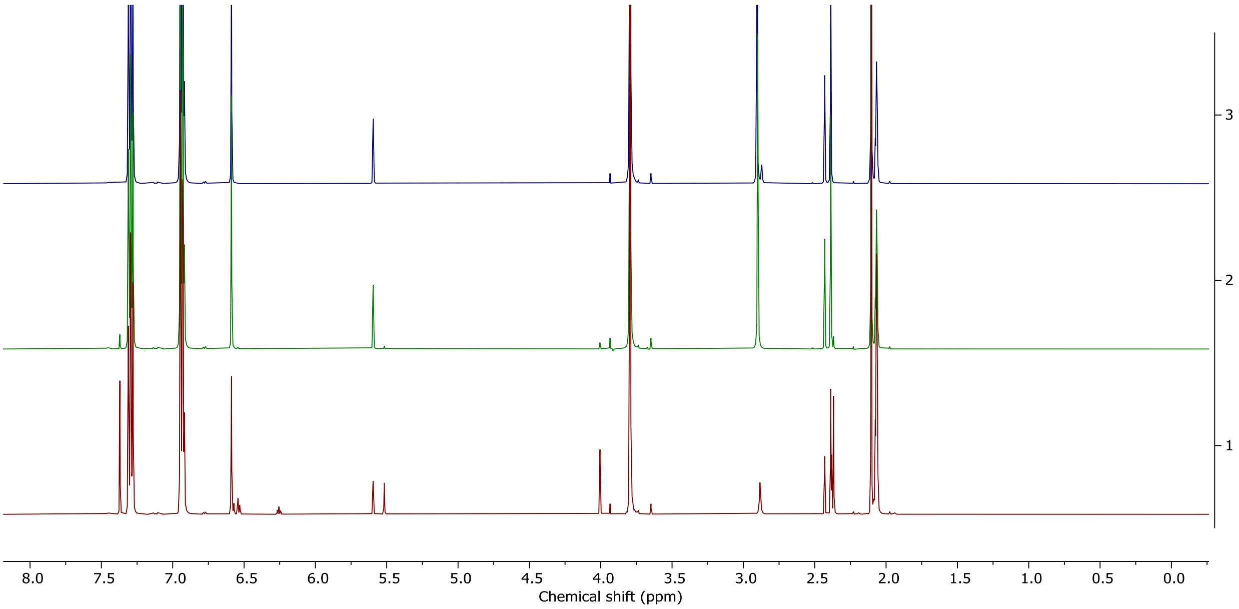

Fig S5: Series of ^{1}H-NMR spectra at 500 MHz, 23 °C, acetone-d_6; 3 = initial spectrum, 2 = 1h at 120 °C, 1 = 18h at 120 °C.

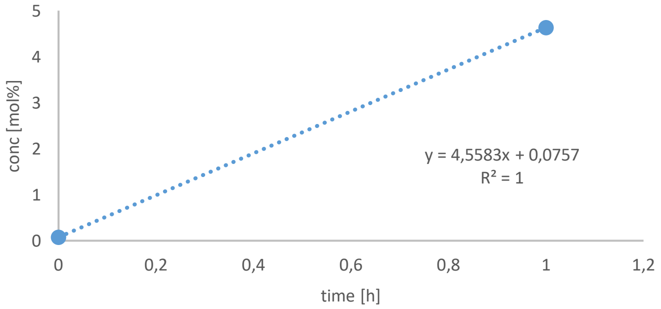

Fig S6: Plot to determine the rate constant of the arene exchange of **SI-3**.

Arene exchange study for [η^6-benzene-η^5-(4-*t*Bu-1-phenoxo)Ru](OTf) (SI-6)

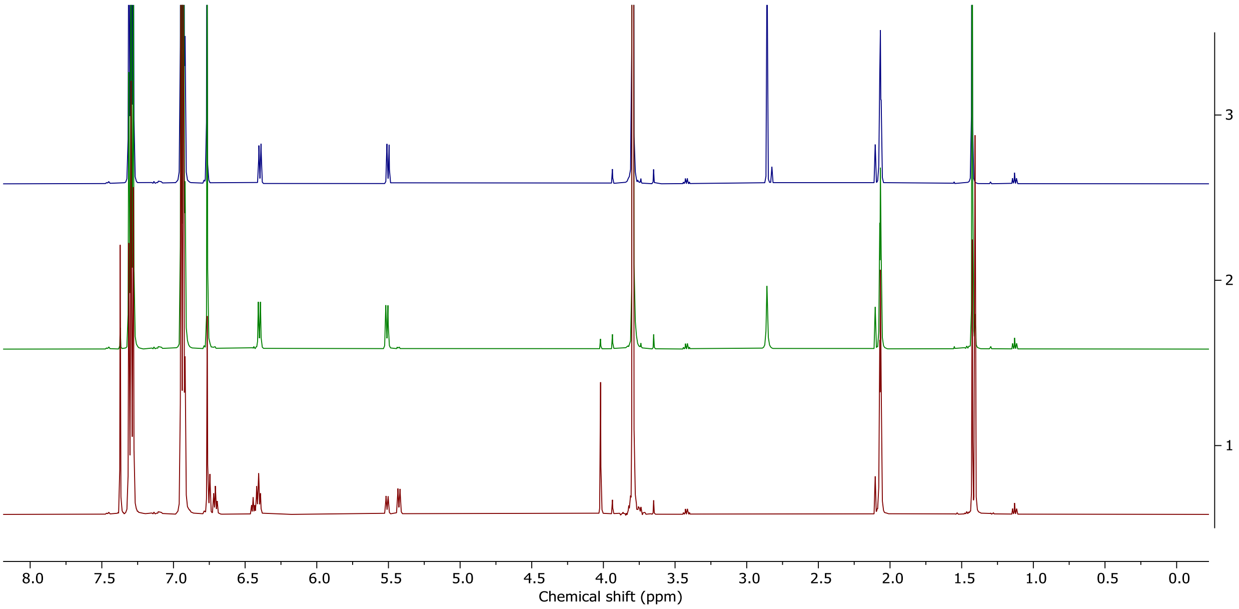

Fig S7: Series of ^{1}H-NMR spectra at 500 MHz, 23 °C, acetone-d_6; 3 = initial spectrum, 2 = 1h at 120 °C, 1 = 18h at 120 °C.

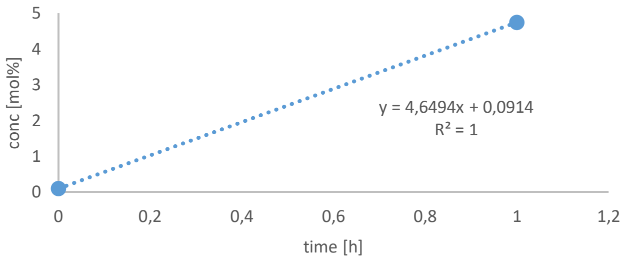

Fig S8: Plot to determine the rate constant of the arene exchange of **SI-6**.

Arene exchange study for [η^6-benzene-η^5-(phenoxo)Ru](OTf) (1c)

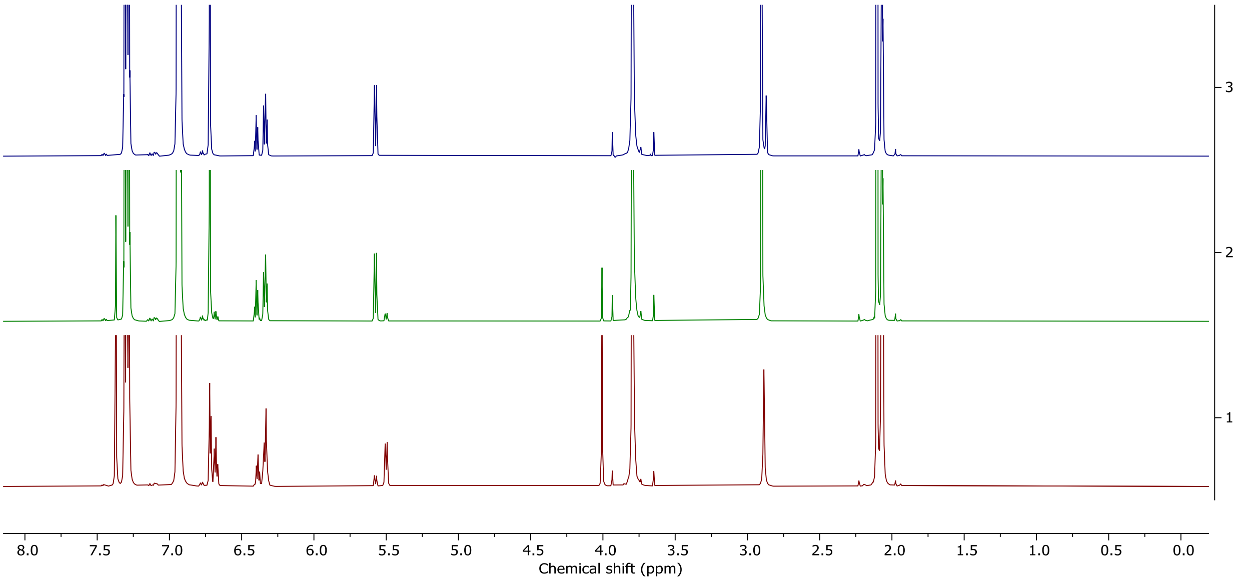

Fig S9: Series of ^{1}H-NMR spectra at 500 MHz, 23 °C, acetone-d_6; 3 = initial spectrum, 2 = 1h at 120 °C, 1 = 18h at 120 °C.

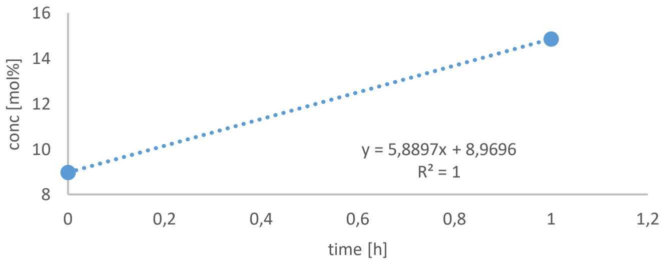

Fig S10: Plot to determine the rate constant of the arene exchange of **1c**. .

Arene exchange study for [η^6-benzene-η^5-(3,5-dimethoxy-1-phenoxo)Ru](OTf) (SI-2)

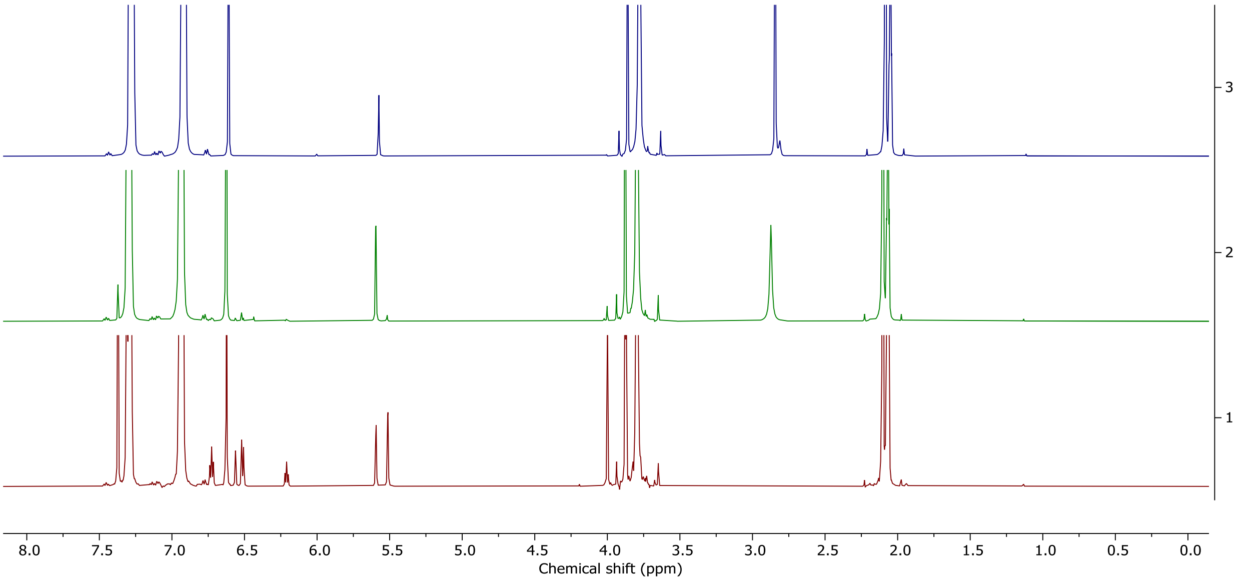

Fig S11: Series of ^{1}H-NMR spectra at 500 MHz, 23 °C, acetone-d_6; 3 = initial spectrum, 2 = 1h at 120 °C, 1 = 18h at 120 °C.

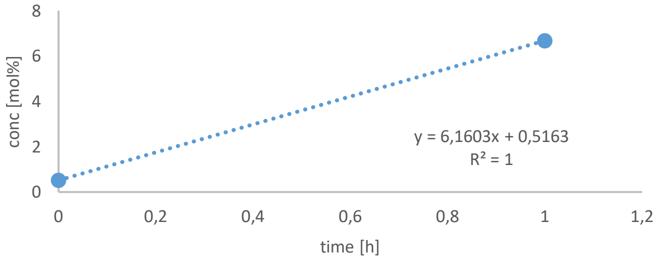

Fig S12: Plot to determine the rate constant of the arene exchange of **SI-2**.

Arene exchange study for [η^6-benzene-η^5-(4-methyl-1-phenoxo)Ru](OTf) (1h)

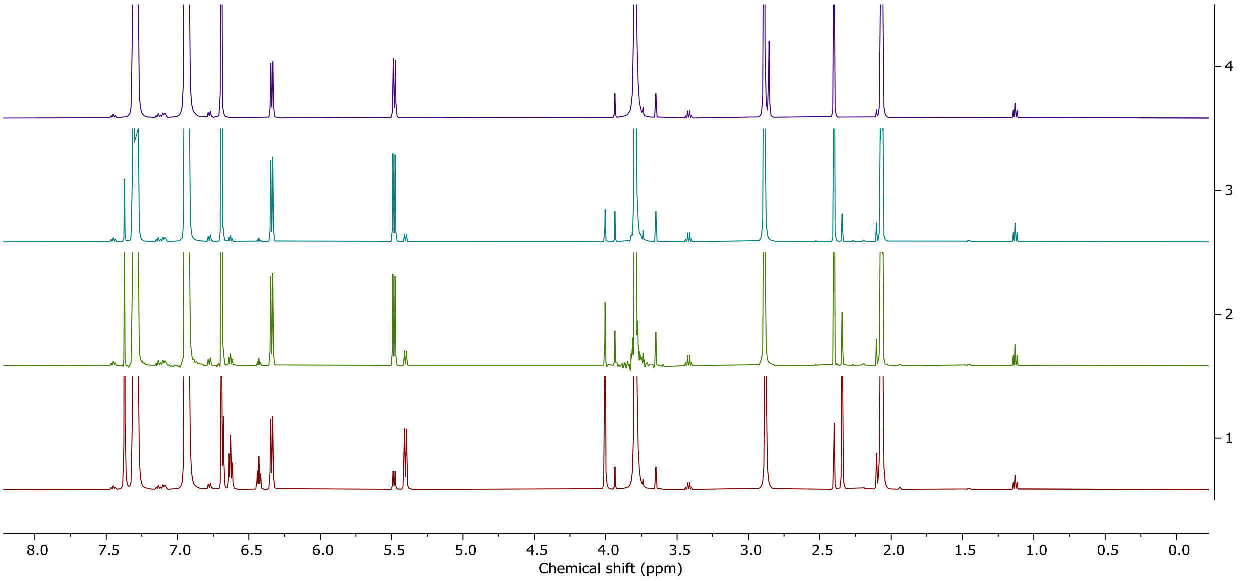

Fig S13: Series of ^{1}H-NMR spectra at 500 MHz, 23 °C, acetone-d_6; 4 = initial spectrum, 3 = 1h at 120 °C, 2 = 2h at 120 °C, 1 = 18h at 120 °C.

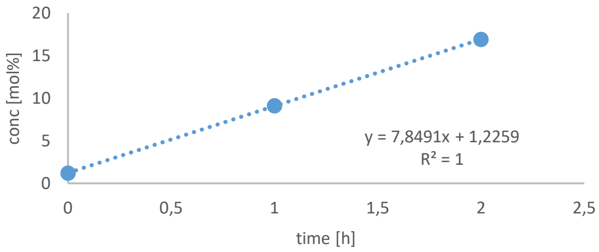

Fig S14: Plot to determine the rate constant of the arene exchange of **1h**.

Arene exchange study for [η^6-benzene-η^5-(4-*n*-butyl-1-phenoxo)Ru](OTf) (1d)

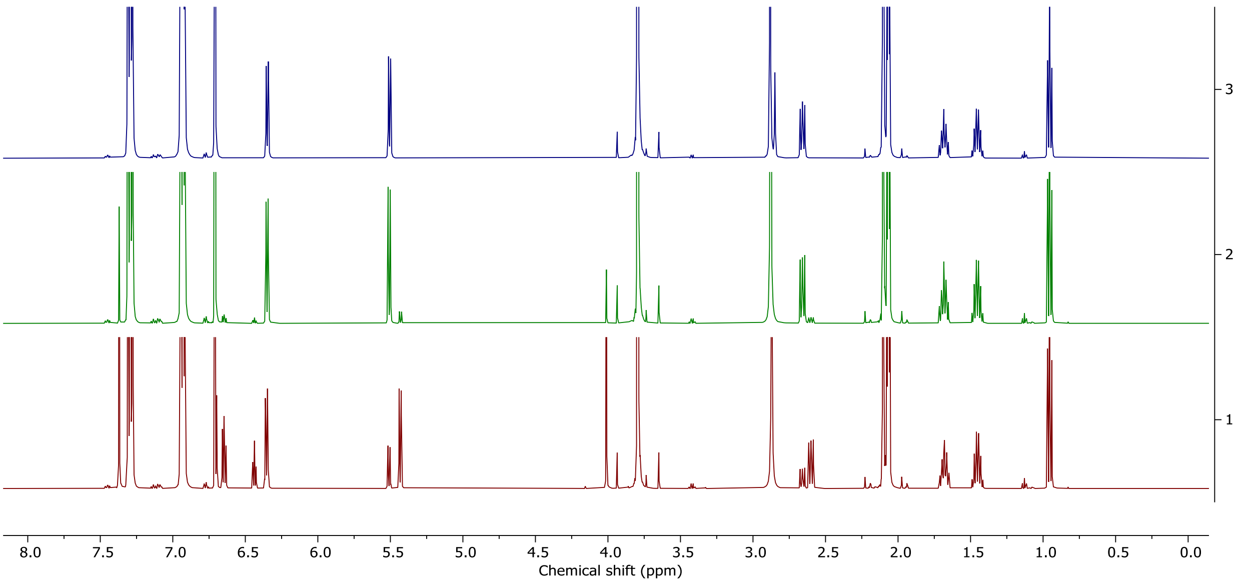

Fig S15: Series of ^{1}H-NMR spectra at 500 MHz, 23 °C, acetone-d_6; 3 = initial spectrum, 2 = 1h at 120 °C, 1 = 18h at 120 °C.

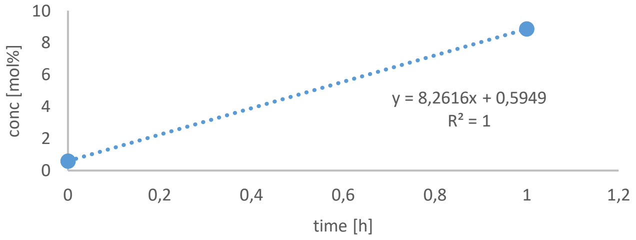

Fig S16: Plot to determine the rate constant of the arene exchange of **1d**.

Arene exchange study for [η^6-benzene-η^5-(4-(2-methoxyethyl)-1-phenoxo)Ru](OTf) (1e)

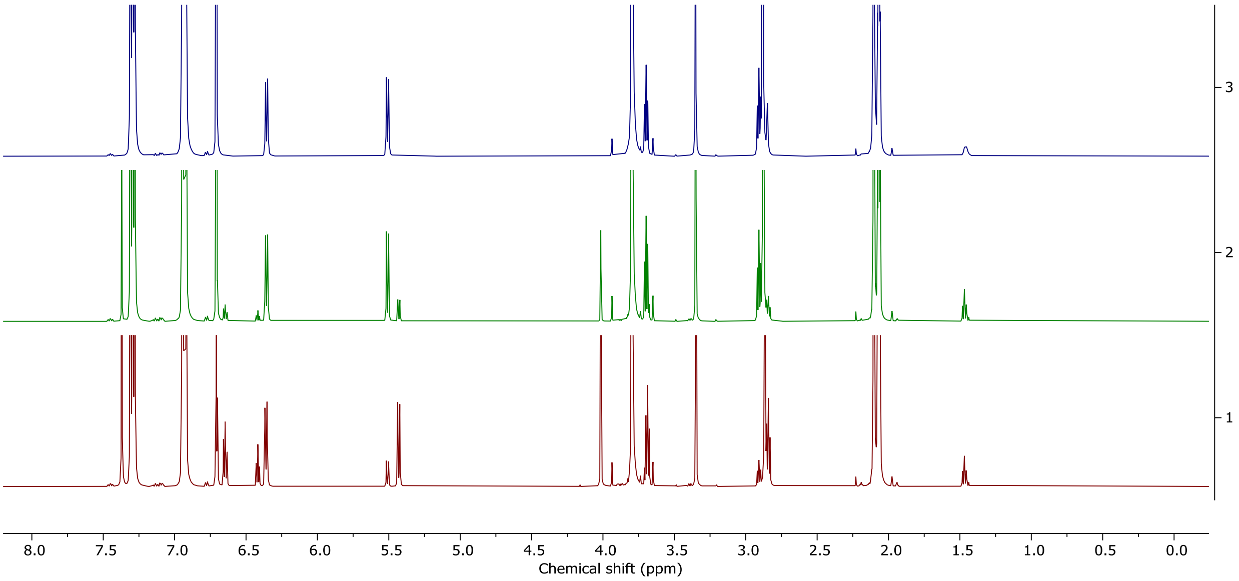

Fig S17: Series of ^{1}H-NMR spectra at 500 MHz, 23 °C, acetone-d_6; 3 = initial spectrum, 2 = 1h at 120 °C, 1 = 18h at 120 °C.

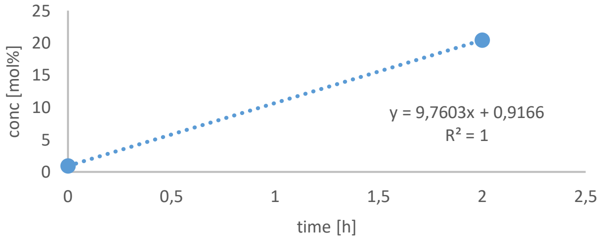

Fig S18: Plot to determine the rate constant of the arene exchange of **1e**.

Arene exchange study for [η^6-benzene-η^5-(2,6-diiodo-1-phenoxo)Ru](OTf) (1f)

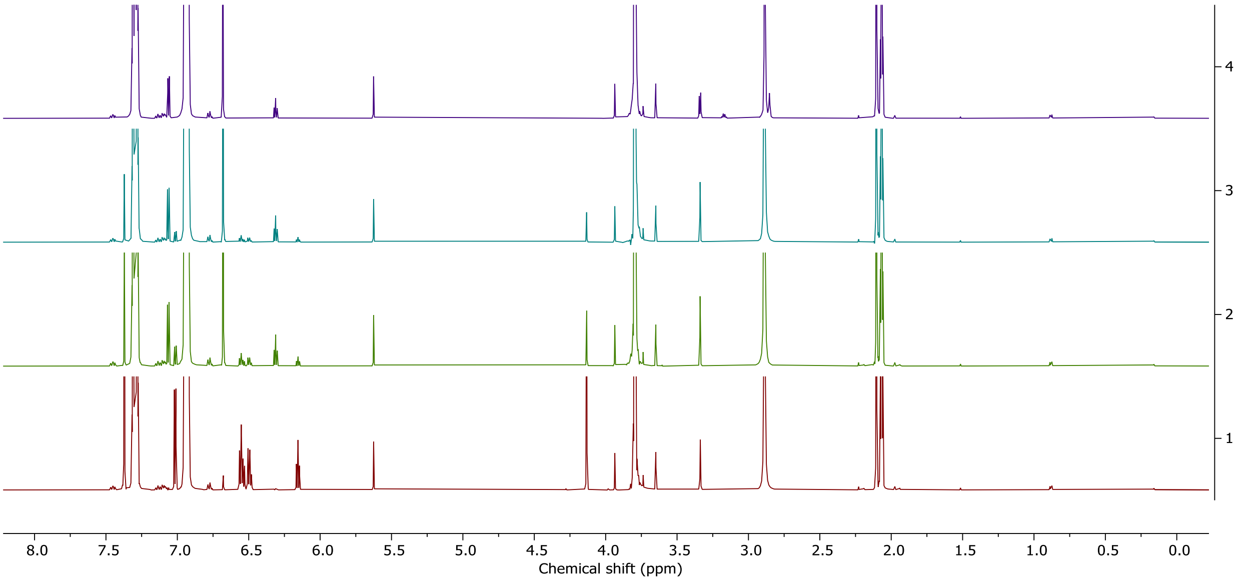

Fig S19: Series of ^{1}H-NMR spectra at 500 MHz, 23 °C, acetone-d_6; 4 = initial spectrum, 3 = 1h at 120 °C, 2 = 1h at 120 °C, 1 = 18h at 120 °C.

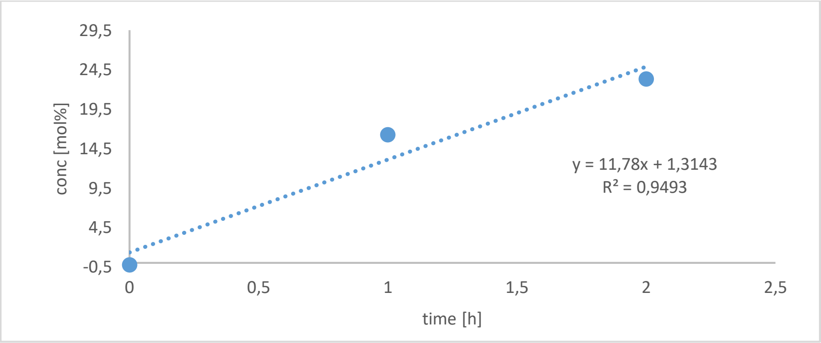

Fig S20: Plot to determine the rate constant of the arene exchange of **1f**.

Arene exchange study for [η^6-benzene-η^5-(4-trifluoromethyl-1-phenoxo)Ru](OTf) (1j)

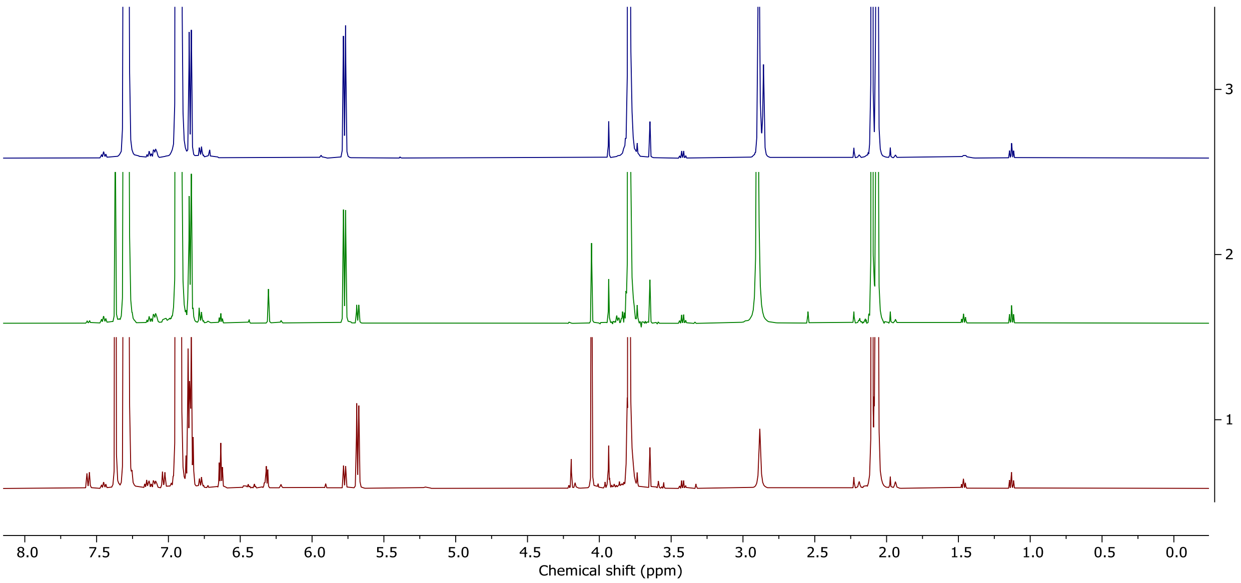

Fig S21: Series of ^{1}H-NMR spectra at 500 MHz, 23 °C, acetone-d_6; 3 = initial spectrum, 2 = 1h at 120 °C, 1 = 18h at 120 °C.

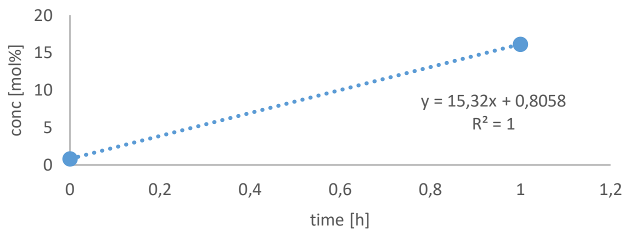

Fig S22: Plot to determine the rate constant of the arene exchange of **1j**.

Arene exchange study for [η^6-benzene-η^5-(4-amino-1-phenoxo)Ru](OTf) (SI-7)

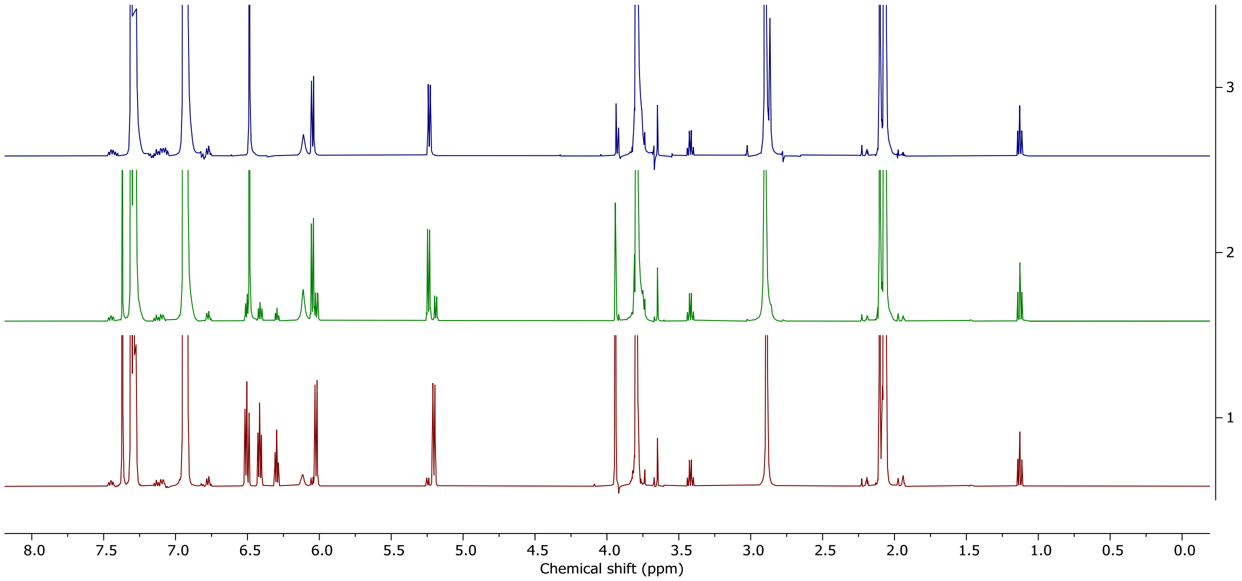

Fig S23: Series of ^{1}H-NMR spectra at 500 MHz, 23 °C, acetone-d_6; 3 = initial spectrum, 2 = 1h at 120 °C, 1 = 18h at 120 °C.

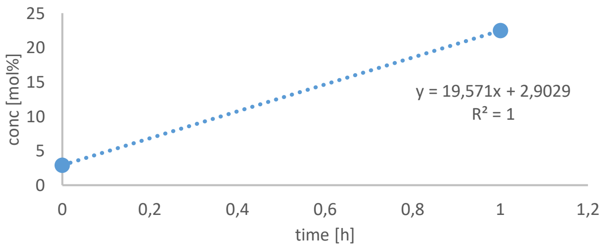

Fig S24: Plot to determine the rate constant of the arene exchange of **SI-7**.

Arene exchange study for [η^6-benzene-η^5-(4-*N*,*N*-dimethylamino-1-phenoxo)Ru](OTf) (1l)

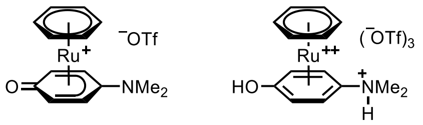

1l **1l-Prot**

Note: The arene exchange is strongly depended on the protonation state of the complex. The arene exchange experiment was carried out three times: 1) with complex **1l** with the addition of 34 mol% of TfOH (25% aqueous), 2) with complex **1l** before deprotonation, and 3) with complex **1l**. Experiment 1) was used for the determination of the arene exchange rate in Fig. S26 and Table S1.

1) 1l with 34 mol% of TfOH (25%, aqueous):

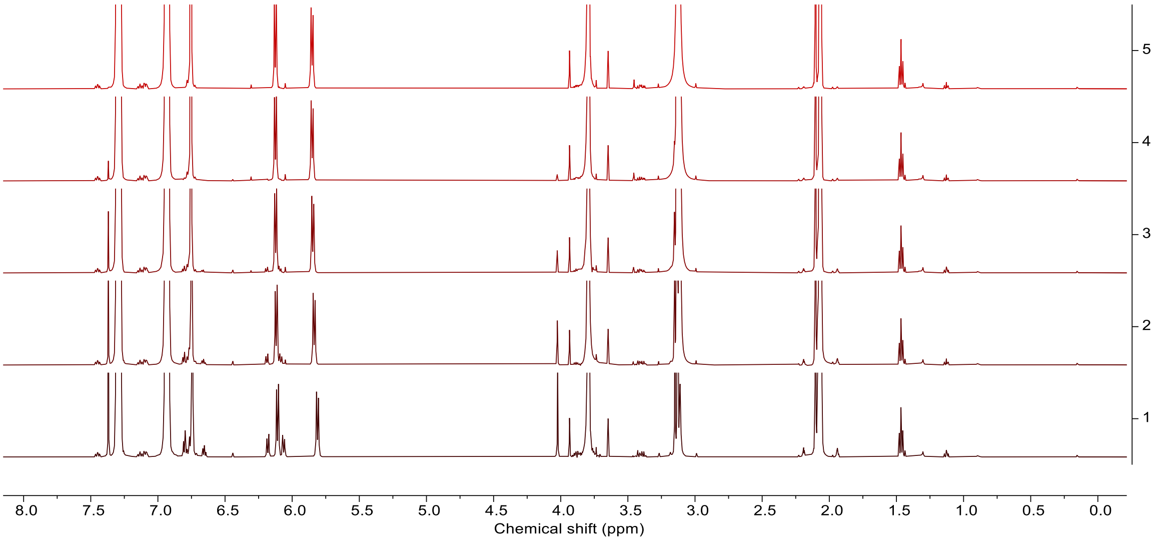

2) 1l before deprotonation:

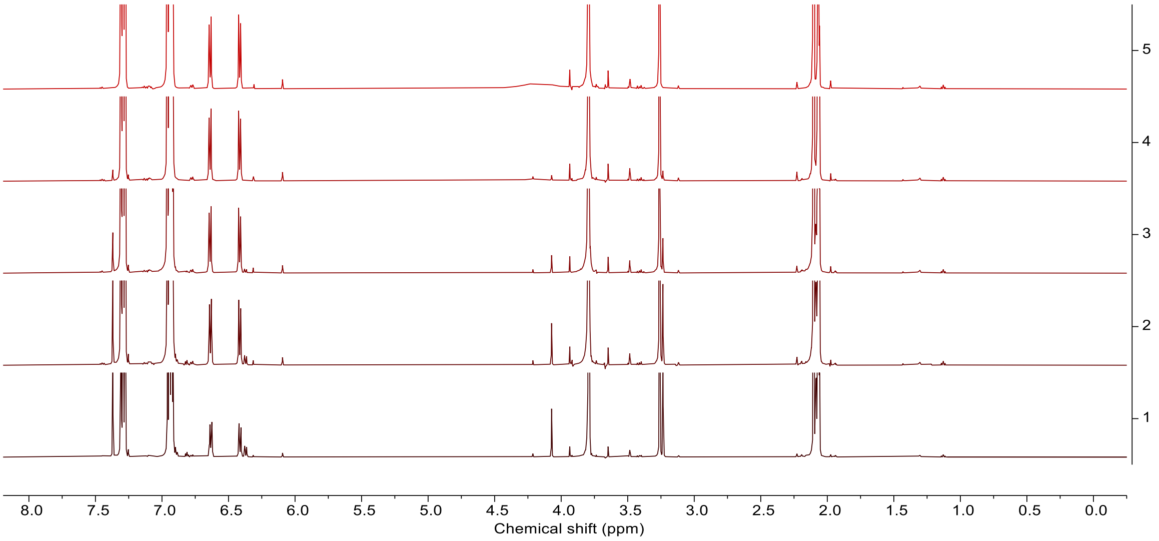

3) 1l after deprotonation:

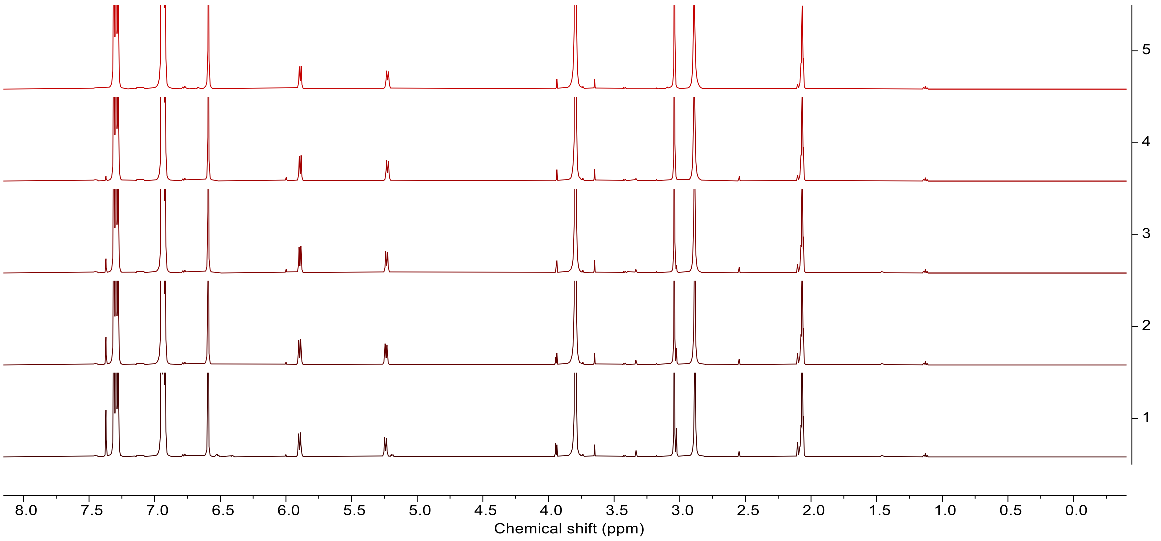

Fig S25: Series of ^{1}H-NMR spectra at 500 MHz, 23 °C, acetone-d_6; 5 = initial spectrum, 4 = 5 min at 120 °C, 3 = 15 min at 120 °C, 2 = 30 min at 120 °C, 1 = 60 min at 120 °C; top: series of ^{1}H-NMR spectra of the arene exchange experiment of **1l** with anisole in the presence of 34 mol% TfOH. Middle: series of ^{1}H-NMR spectra of the arene exchange experiment of **1l** before deprotonation with anisole. Bottom: series of ^{1}H-NMR spectra of the arene exchange experiment of **1l** with anisole.

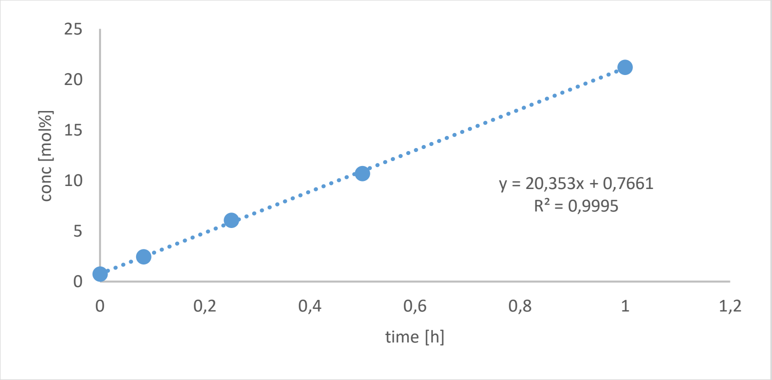

Fig S26: Plot to determine the rate constant of the arene exchange of **1l** using the NMR data of the measurement of complex **1l** under the addition of 34 mol% TfOH (25% aqueous).

Arene exchange study for [η^6-benzene-η^5-(4-methoxy-1-phenoxo)Ru](OTf) (1i)

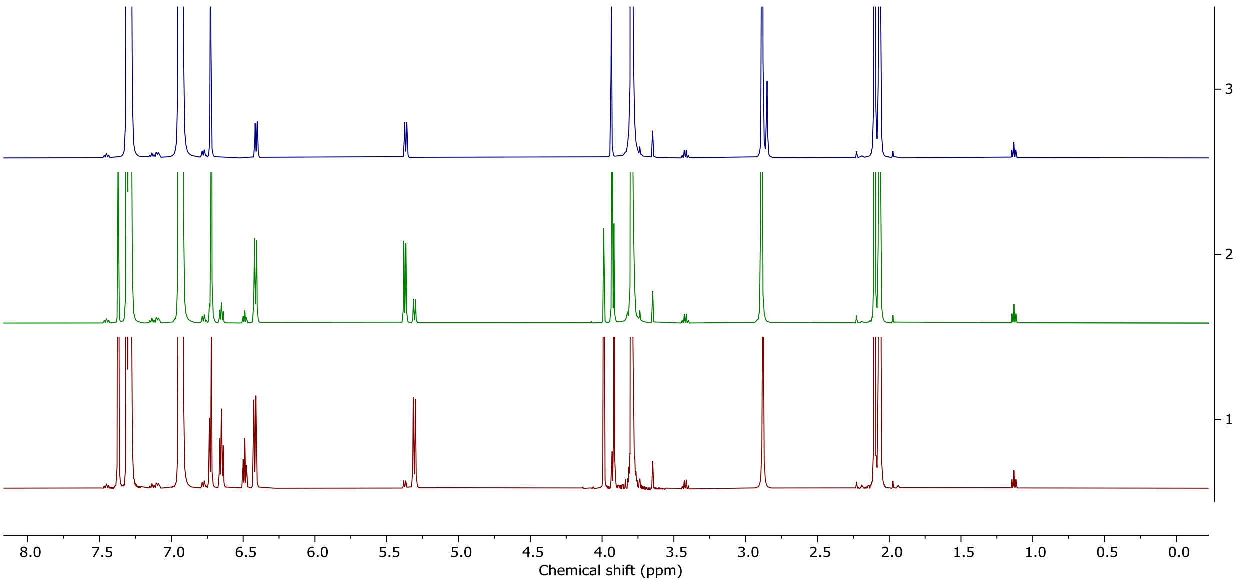

Fig S27: Series of ^{1}H-NMR spectra at 500 MHz, 23 °C, acetone-d_6; 3 = initial spectrum, 2 = 1h at 120 °C, 1 = 18h at 120 °C.

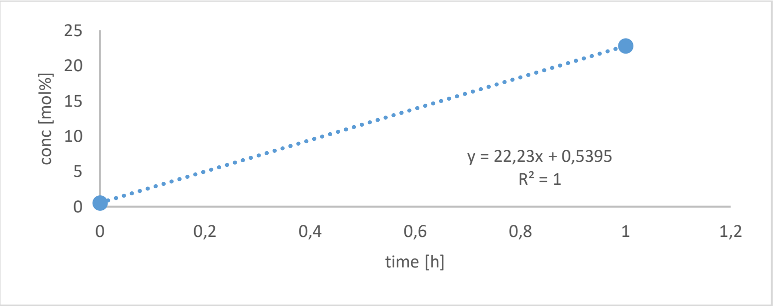

Fig S28: Plot to determine the rate constant of the arene exchange of **1i**.

Arene exchange study for [η^6-benzene-η^5-(2-chloro-1-phenoxo)Ru](OTf) (SI-9)

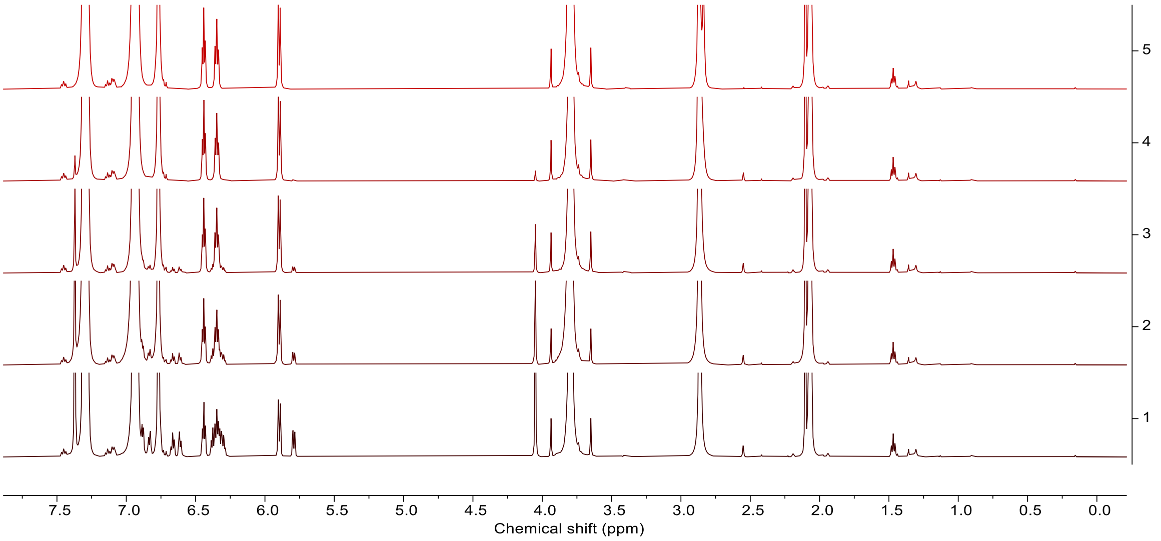

Fig S29: Series of ^{1}H-NMR spectra at 500 MHz, 23 °C, acetone-d_6; 5 = initial spectrum, 4 = 5 min at 120 °C, 3 = 15 min at 120 °C, 2 = 30 min at 120 °C, 1 = 60 min at 120 °C.

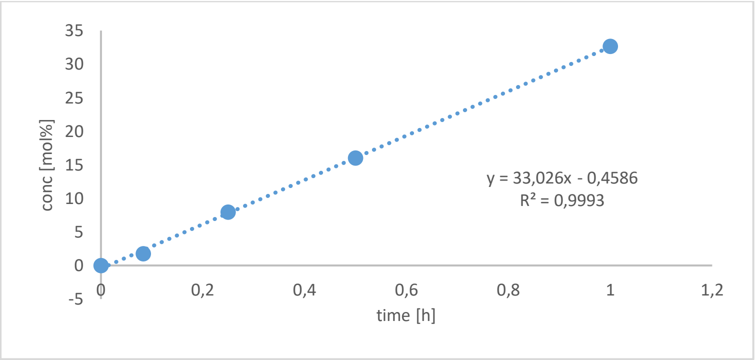

Fig S30: Plot to determine the rate constant of the arene exchange of **SI-9**.

Arene exchange study for [η^6-benzene-η^5-(2,6-dibromo-1-phenoxo)Ru](OTf) (1k)

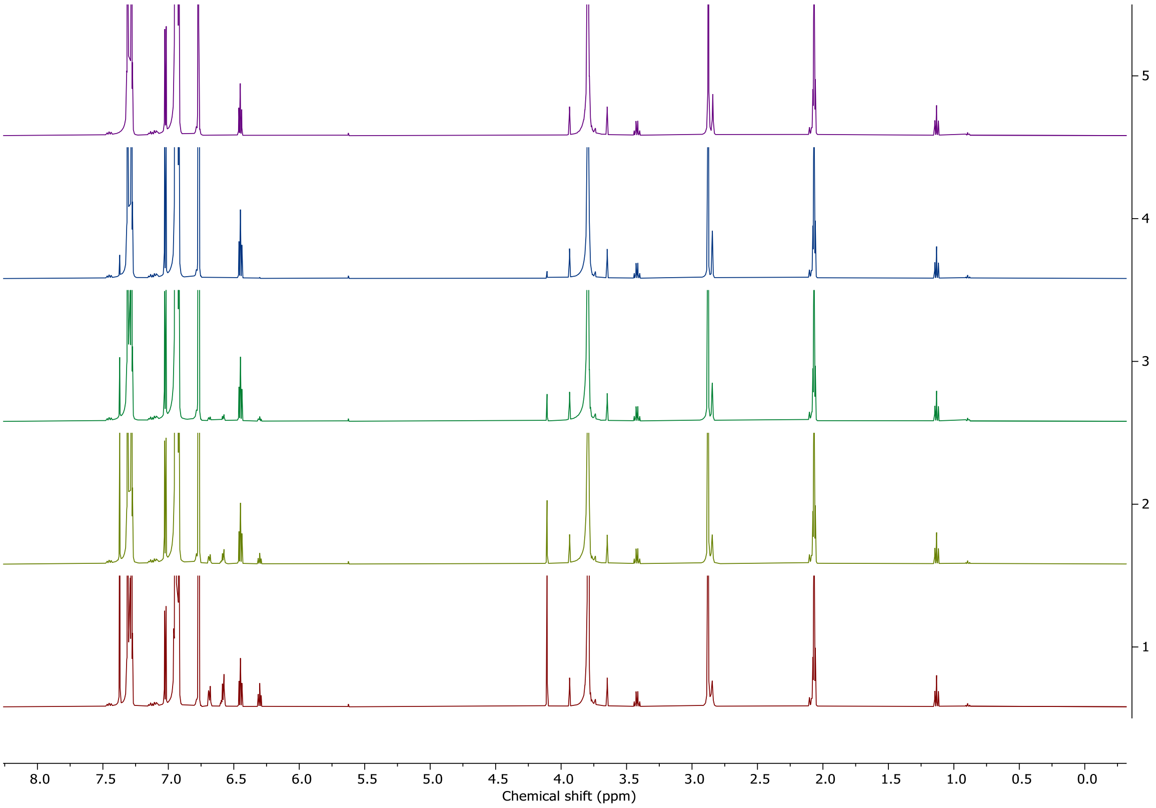

Fig S31: Series of ^{1}H-NMR spectra at 500 MHz, 23 °C, acetone-d_6; 5 = initial spectrum, 4 = 5 min at 120 °C, 3 = 15 min at 120 °C, 2 = 30 min at 120 °C, 1 = 60 min at 120 °C.

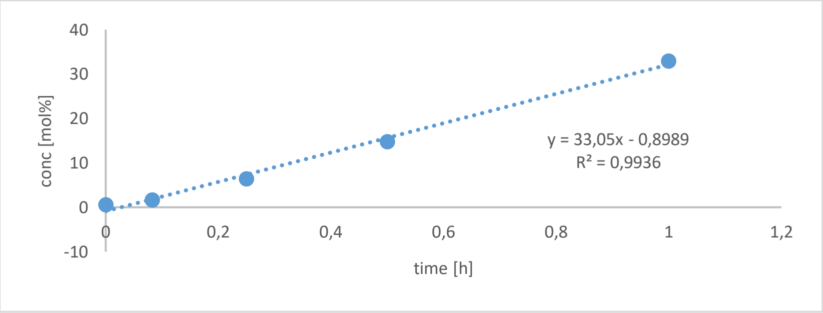

Fig S32: Plot to determine the rate constant of the arene exchange of **1k**.

Arene exchange study for [η^6-benzene-η^5-(2,6-dichloro-1-phenoxo)Ru](OTf) (1g)

Acetone-d_6, 500 MHz, 23 °C; 3 = initial spectrum, 2 = 1h at 120 °C, 1 = 18h at 120 °C

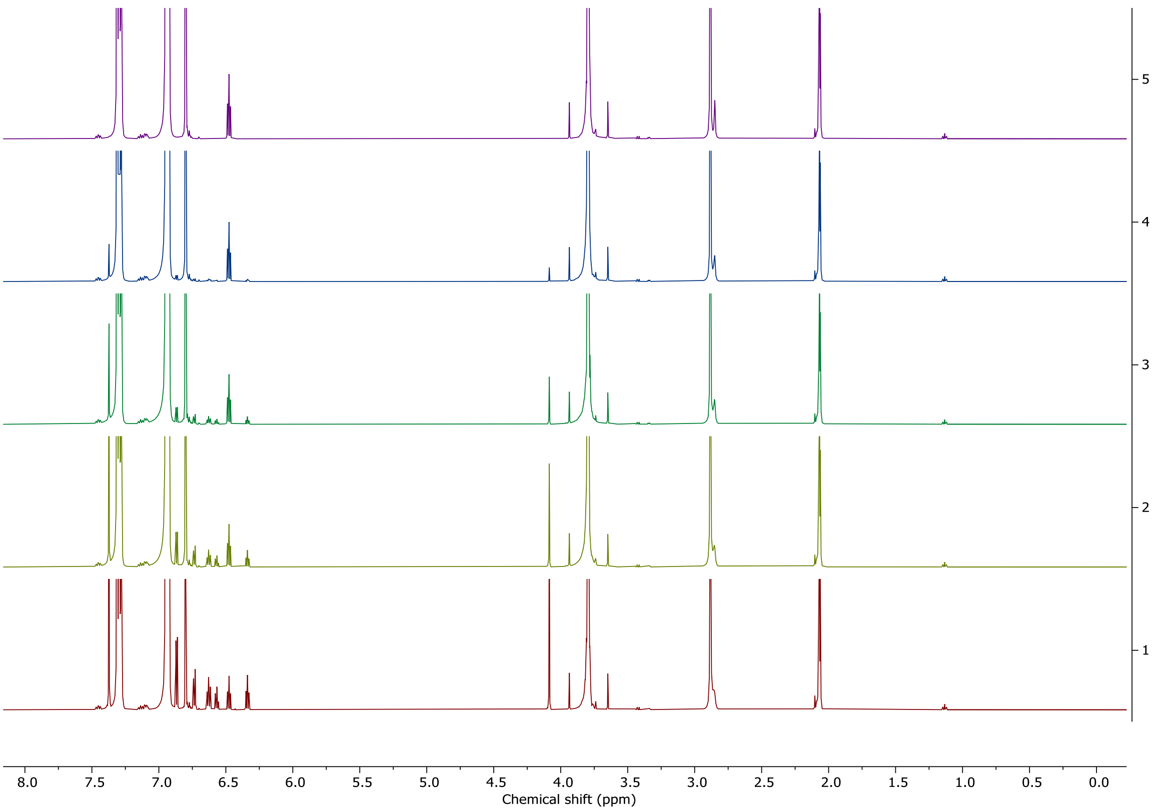

Fig S33: Series of ^{1}H-NMR spectra at 500 MHz, 23 °C, acetone-d_6; 5 = initial spectrum, 4 = 5 min at 120 °C, 3 = 15 min at 120 °C, 2 = 30 min at 120 °C, 1 = 60 min at 120 °C.

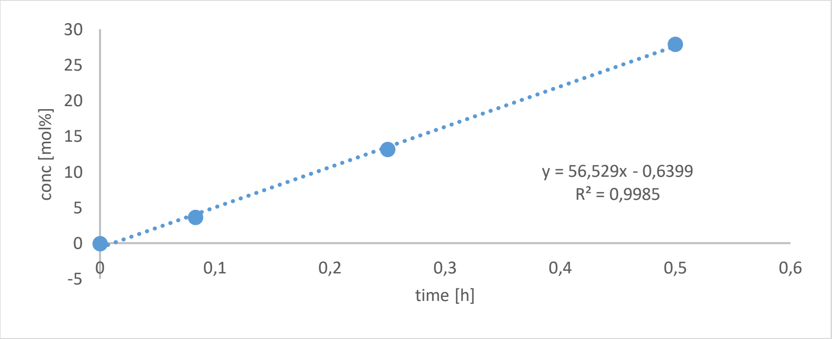

Fig S34: Plot to determine the rate constant of the arene exchange of **1g**.

Arene exchange study for [η^6-benzene-η^5-(2,6-dibromo-4-methoxy-1-phenoxo)Ru](OTf) (1a)

Acetone-d_6, 500 MHz, 23 °C; 4 = initial spectrum, 3 = 1h at 120 °C , 2 = 2h at 120 °C, 1 = 18h at 120 °C

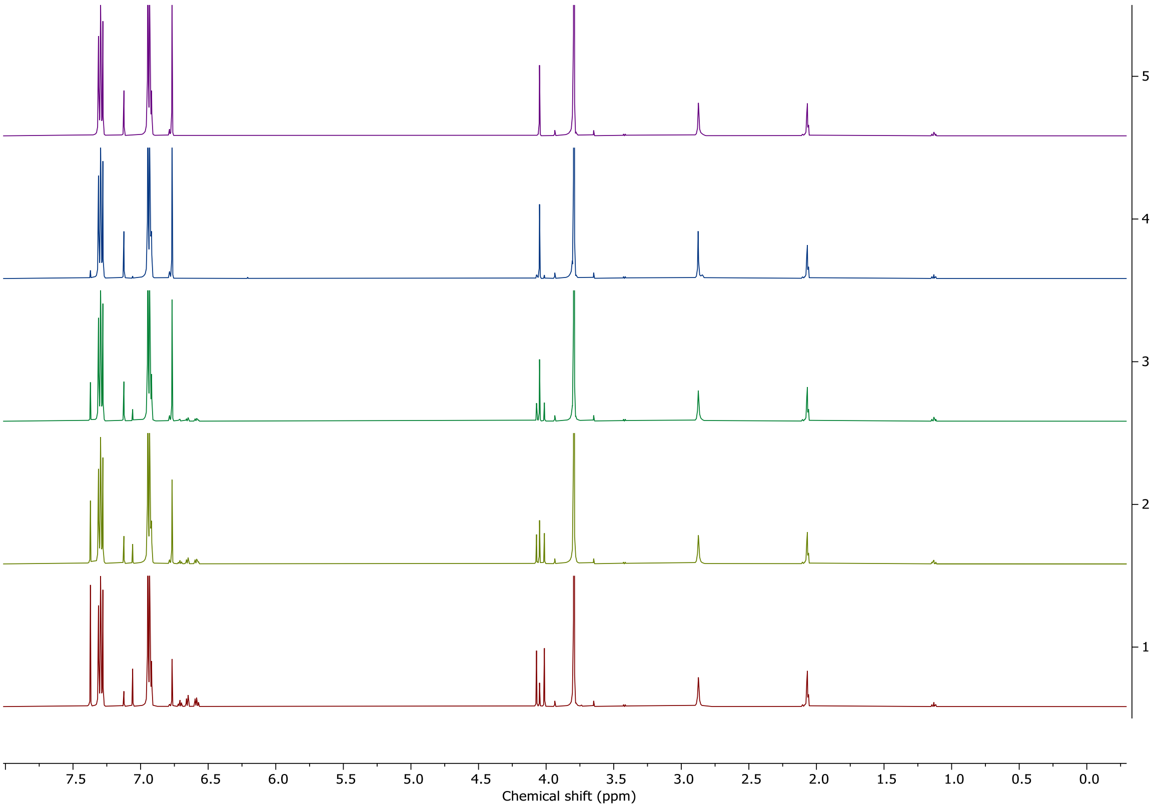

Fig S35: Series of ^{1}H-NMR spectra at 500 MHz, 23 °C, acetone-d_6; 5 = initial spectrum, 4 = 5 min at 120 °C, 3 = 15 min at 120 °C, 2 = 30 min at 120 °C, 1 = 60 min at 120 °C.

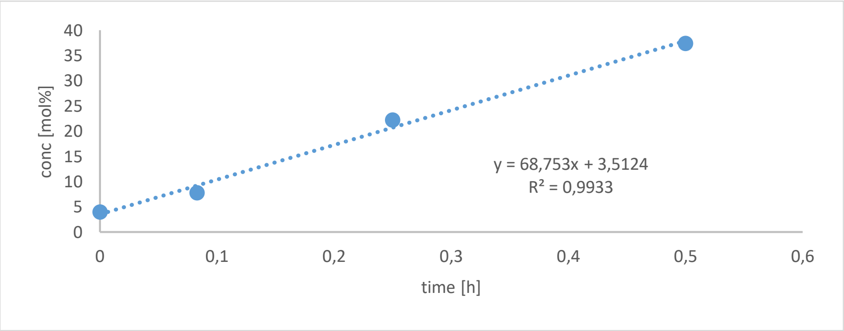

Fig S36: Plot to determine the rate constant of the arene exchange of **1a**.

Arene exchange study for [η^6-benzene-η^6-(4-methoxymethylanisole)Ru](OTf) (1b)

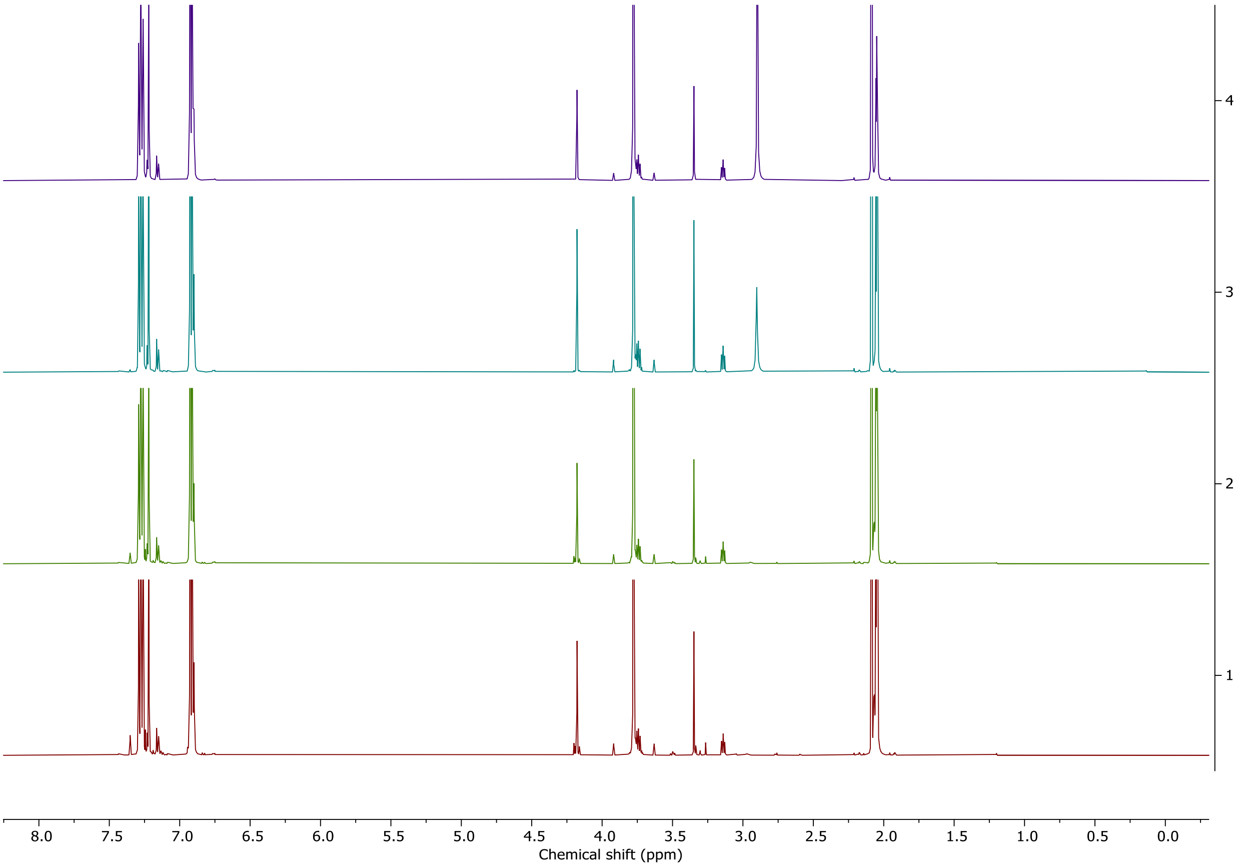

Fig S37: Series of ^{1}H-NMR spectra at 500 MHz, 23 °C, acetone-d_6; 4 = initial spectrum, 3 = 1h at 120 °C, 2 = 2h at 120 °C, 1 = 18h at 120 °C; **No arene exchange visible**, only decomposition, anisole complex not detected by HRMS.

Arene exchange study for [η^6-benzene-η^6-(pentamethylcyclopentadienyl)Ru](OTf) (1-Cp*)

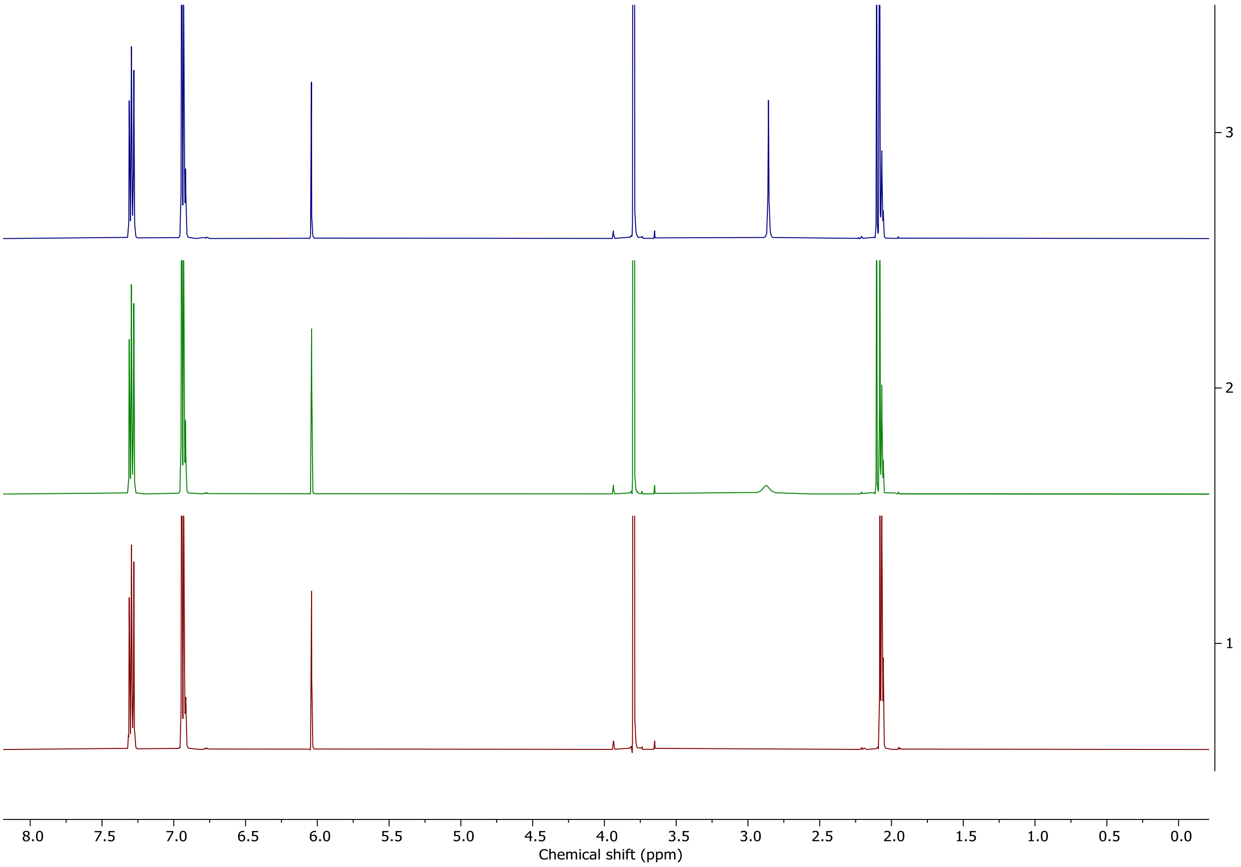

Fig S38: Series of ^{1}H-NMR spectra at 500 MHz, 23 °C, acetone-d_6; 3 = initial spectrum, 2 = 1h at 120 °C, 1 = 18h at 120 °C; **No arene exchange visible**; anisole complex not detected by HRMS.

Arene exchange study for [η^6-benzene-η^6-(pentamethylcyclopentadienyl)Ru](OTf) (1-Cp*) in presence of TBA-OMs

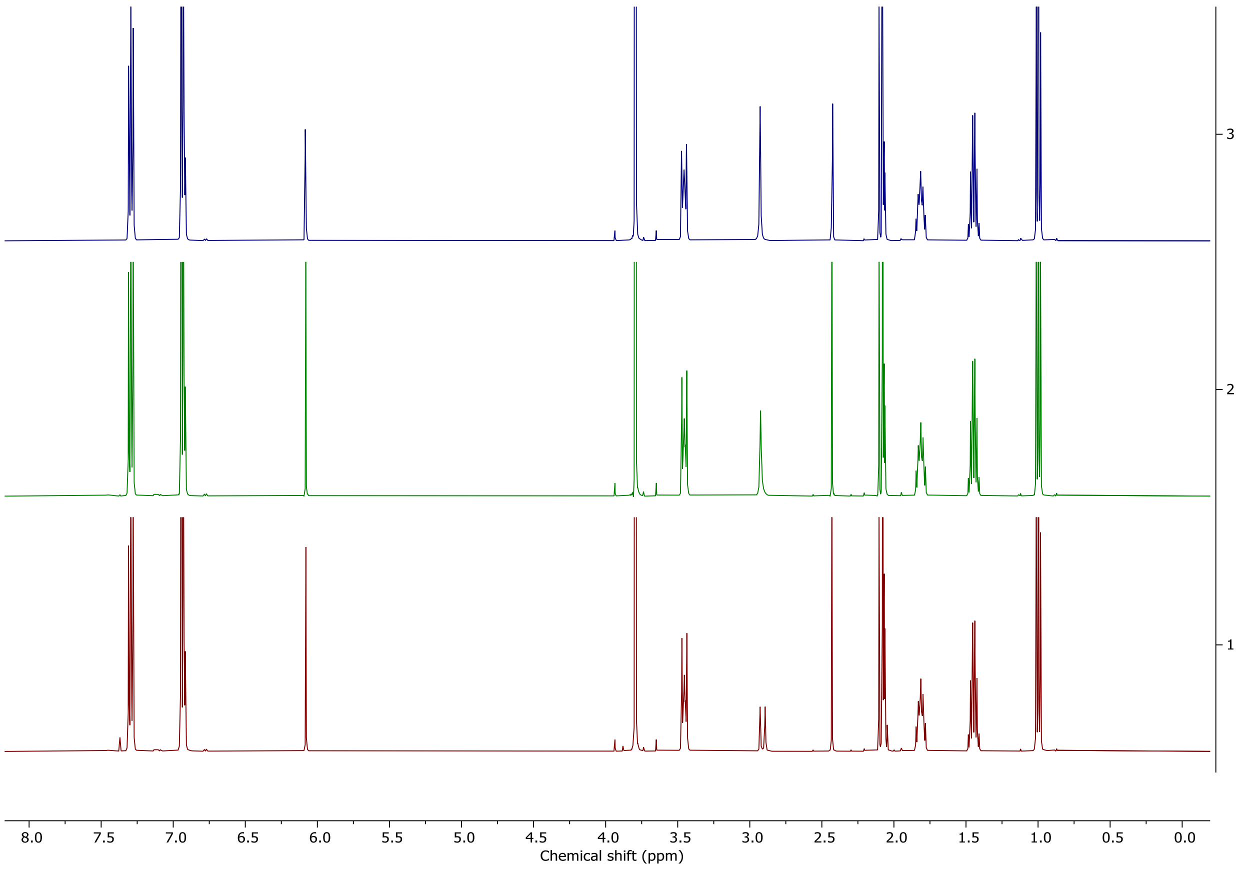

Fig S39: Series of ^{1}H-NMR spectra at 500 MHz, 23 °C, 2.0 equiv. of TBA-OMs added subsequently to the addition of complex to the J-Young tube; acetone-d_6; 3 = initial spectrum, 2 = 1h at 120 °C, 1 = 18h at 120 °C; **No arene exchange visible**; anisole complex not detected by HRMS.

Counterion Effect Study

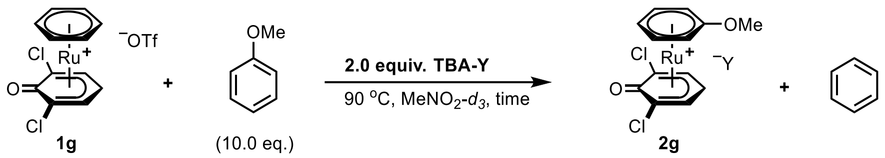

A 5 mm J-Young NMR tube was charged with **1g** (10 mg, 20 µmol, 1.0 equiv.), anisole (22 µL, 22 mg, 0.20 mmol, 10 equiv.) and tetrabutylammonium salt (40 µmol, 2.0 equiv.). Deuterated nitromethane (0.50 mL, *c* = 40 mM) was added and the initial ^{1}H NMR spectrum was measured. Subsequently, the tube was placed in a preheated (90 °C) NMR sample head. ^{1}H NMR spectra were recorded every 5 min at 90 °C for several hours. The rate constants were determined by plotting of the conversion in mol%, which was extracted from the ^{1}H-NMR integrals, against the reaction time. To convert the rate constant unit to mol·L^{-1}·h^{-1} the values were multiplied by the concentration (0.04 M) of the reaction solution.

Note: For $^-$Br and $^-$OAc, sidereactivity was observed, therefore no significant rate constant was extracted from the NMR spectra.

Table S2 Counter-ion effect study of Ru–phenoxo complexes:

Counterion (Y)	k_{init} [mol%·h^{-1}]	k_{init} [mol/l·h^{-1}]
$^{\ominus}$Br	-	-
$^{\ominus}$OAc	-	-
$^{\ominus}PF_6$	<0.28	<0.01
$^{\ominus}BF_4$	<0.28	<0.01
$^{\ominus}$OTf	<0.28	<0.01
$(NO_2)_2PhSO_3^{\ominus}$	7.89	0.65
$^{\ominus}$OTs	26.12	1.04
$^{\ominus}$OMs	46.66	1.86

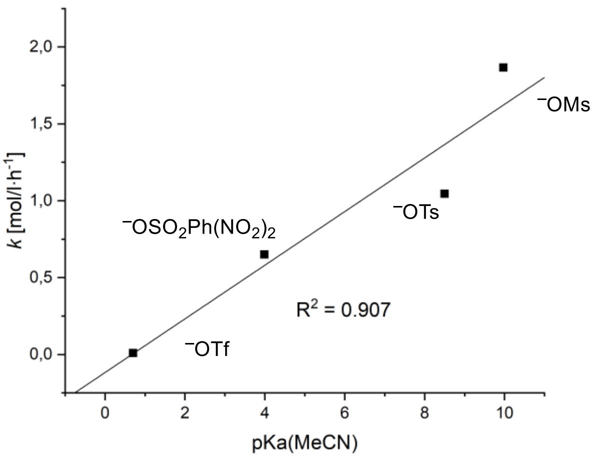

Fig S40: Linear fit of rates for selected counterions from Table S2.

Counterion study for $^{-}PF_6$

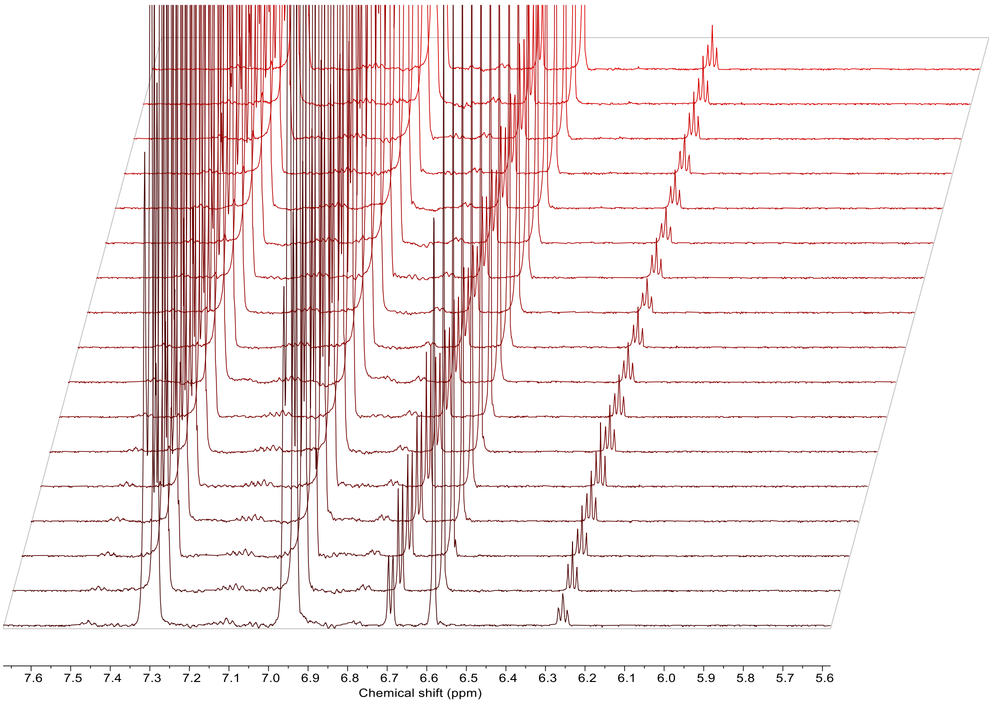

Fig S41: Series of ^{1}H-NMR spectra at 500 MHz, 363 K, $MeNO_2$-d_3 measured in 5 min intervals. No significant arene exchange observed.

Counterion study for $^{-}BF_4$

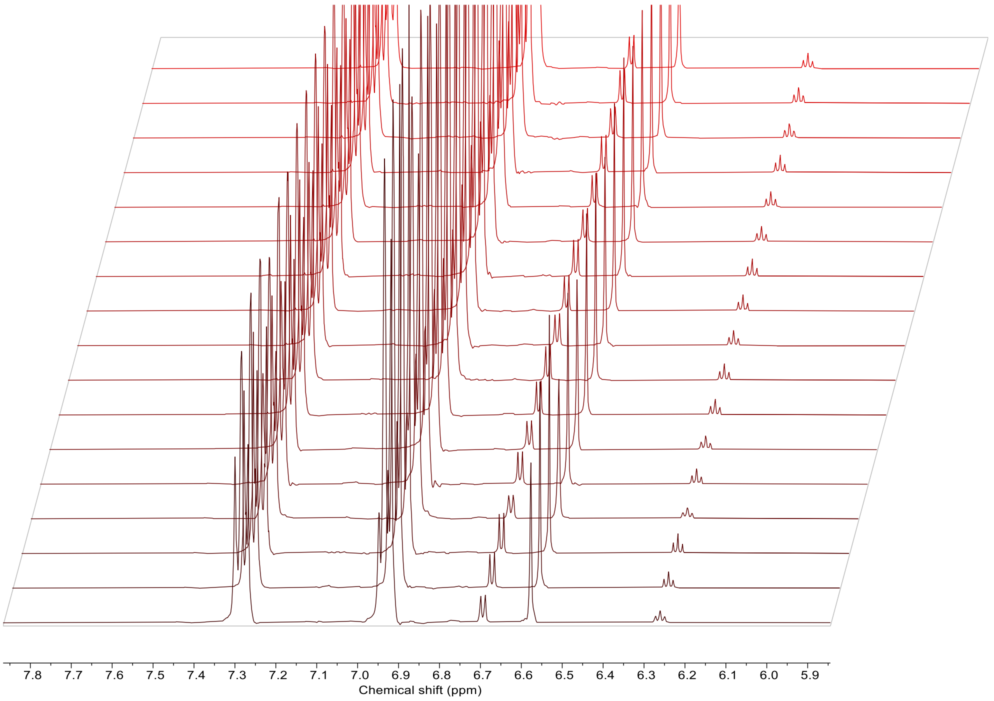

Fig S42: Series of ^{1}H-NMR spectra at 500 MHz, 363 K, $MeNO_2$-d_3 measured in 5 min intervals. No significant arene exchange observed.

Counterion study for $^{-}$OTf

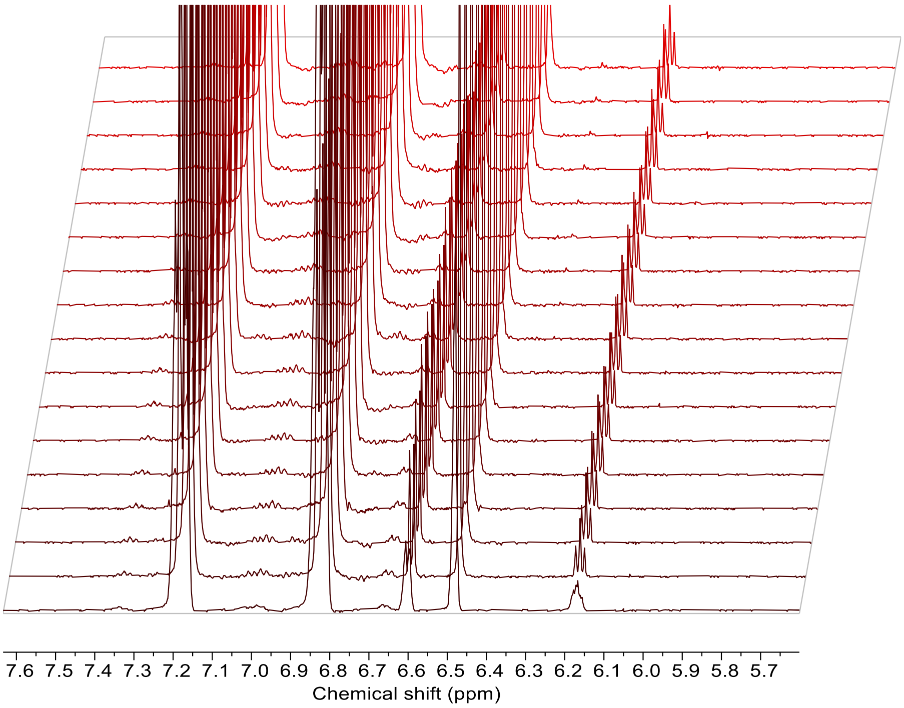

Fig S43: Series of ^{1}H-NMR spectra at 500 MHz, 363 K, $MeNO_2$-d_3 measured in 5 min intervals. No significant arene exchange observed.

Counterion study for $^{-}OSO_2Ph(NO_2)_2$

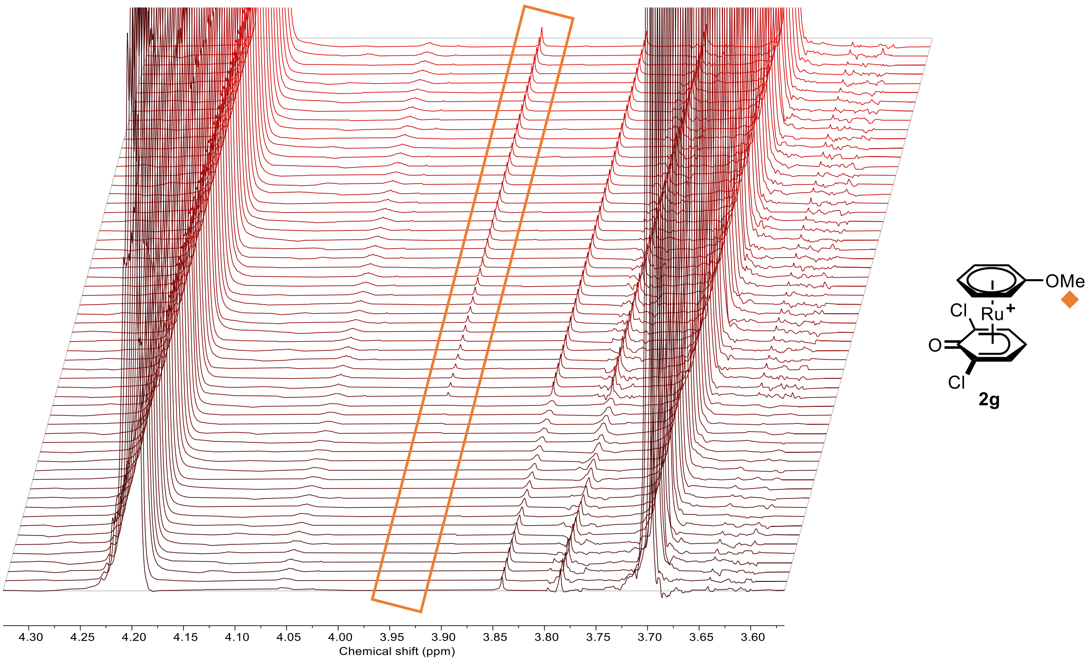

Fig S44: Series of ^{1}H-NMR spectra at 500 MHz, 363 K, $MeNO_2$-d_3 measured in 5 min intervals. Orange box = singlet at 3.95 ppm used for determination of rate constant; orange diamond = signal of **2g** used for determination of rate constant.

Counterion study for ⁻OTs

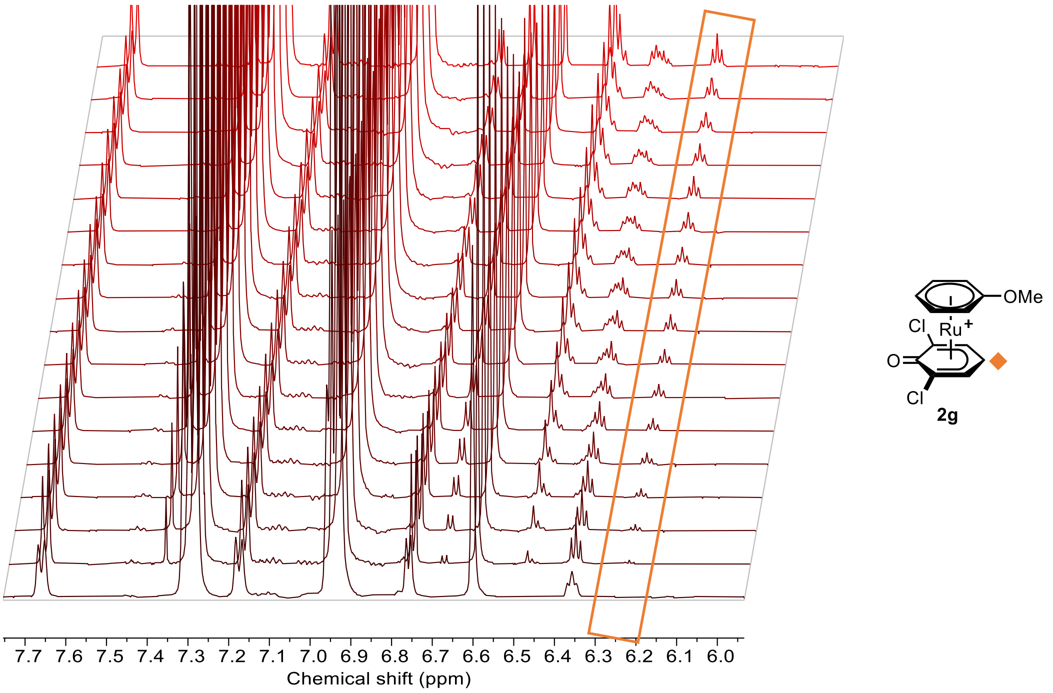

Fig S45: Series of ^{1}H-NMR spectra at 500 MHz, 363 K, MeNO$_2$-d_3 measured in 5 min intervals. Orange box = triplet at 6.25 ppm used for determination of rate constant; orange diamond = signal of **2g** used for determination of rate constant.

Counterion study for ⁻OMs

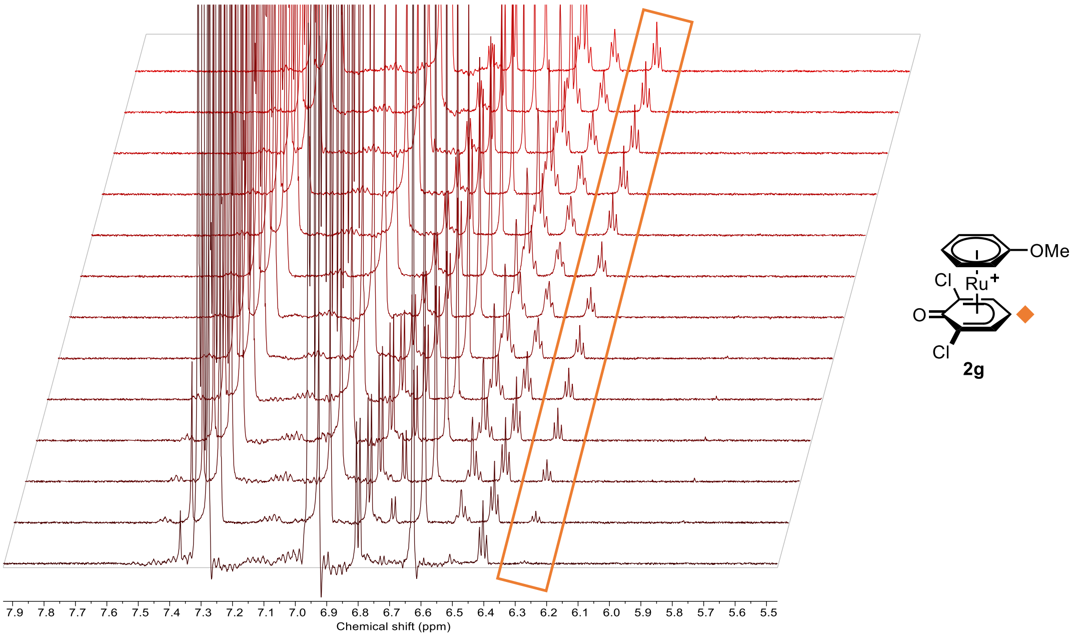

Fig S46: Series of ^{1}H-NMR spectra at 500 MHz, 363 K, MeNO$_2$-d_3 measured in 5 min intervals. Orange box = triplet at 6.3 ppm used for determination of rate constant; orange diamond = signal of **2g** used for determination of rate constant.

Counterion study for ⁻OMs in presence of protic additives

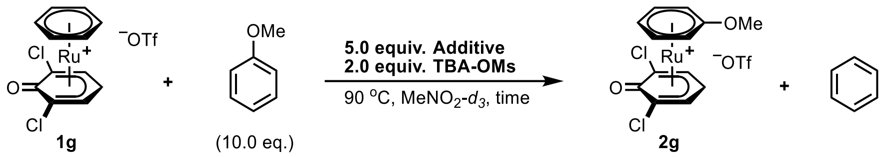

A 5 mm J-Young NMR tube was charged with **1g** (10 mg, 20 µmol, 1.0 equiv.), anisole (22 µL, 22 mg, 0.20 mmol, 10 equiv.), ***n*-pentanol** (11 µL, 8.8 mg, 0.1 mmol, 5.0 equiv.) or **pelargonic acid** (18 µL, 16 mg, 0.1 mmol, 5.0 equiv.) and TBA-OMs (14 mg, 40 µmol, 2.0 equiv.). Deuterated nitromethane (0.50 mL, c = 40 mM) was added and the initial ^{1}H NMR spectrum was measured. Subsequently, the tube was placed in a preheated (90 °C) NMR sample head. ^{1}H NMR spectra were recorded every 5 min at 90 °C for several hours. The rate constants were determined by plotting of the conversion in mol%, which was extracted from the ^{1}H-NMR integrals, against the reaction time.

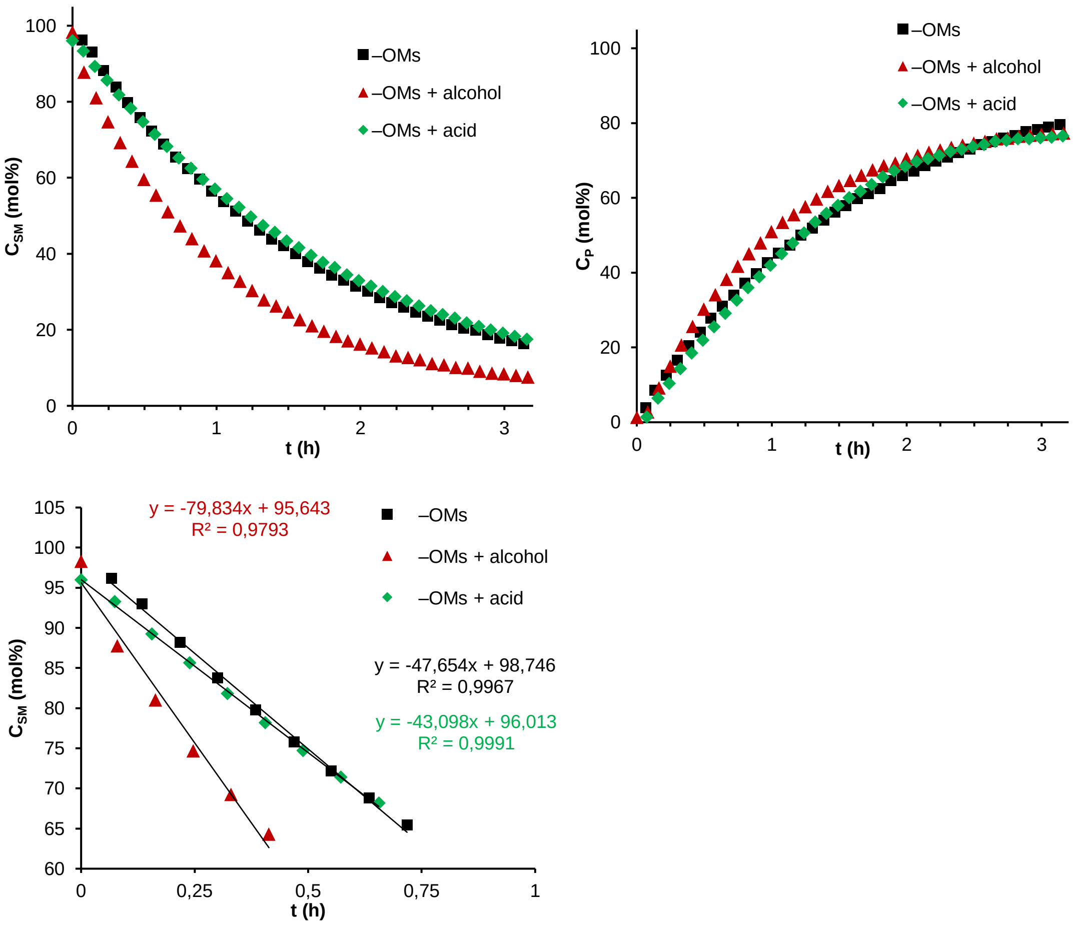

Fig S47: Top left: concentration decrease of benzene complex **1g** in presence of ⁻OMs with different protic additives plotted against the reaction time; black squares ⁻OMs, green diamonds = ⁻OMs and pelargonic acid, red triangles = ⁻OMs and *n*-pentanol. **Top right:** concentration increase of anisole complex **2g** in presence of ⁻OMs with different protic additives plotted against the reaction time; black squares = ⁻OMs, green diamonds = ⁻OMs and pelargonic acid, red triangles = ⁻OMs and *n*-pentanol. **Bottom:** initial rates for benzene dissociation from complex **1g**; black squares = ⁻OMs, green diamonds = ⁻OMs and pelargonic acid, red triangles = ⁻OMs and *n*-pentanol.

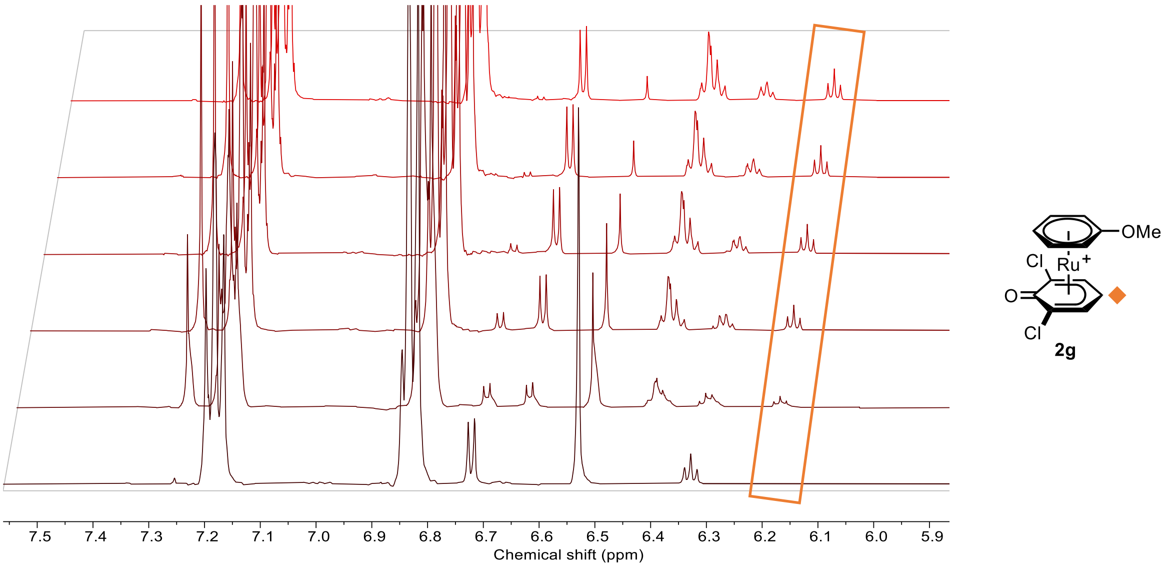

Fig S48: Series of ^{1}H-NMR spectra at 500 MHz, 363 K, MeNO$_2$-d_3 measured in 5 min intervals of **1g** in presence of $^-$OMs and *n*-pentanol at 90 °C. Orange box = ^{1}H-NMR signal of **2g**; orange diamond = proton of **2g** responsible for the signal in orange box.

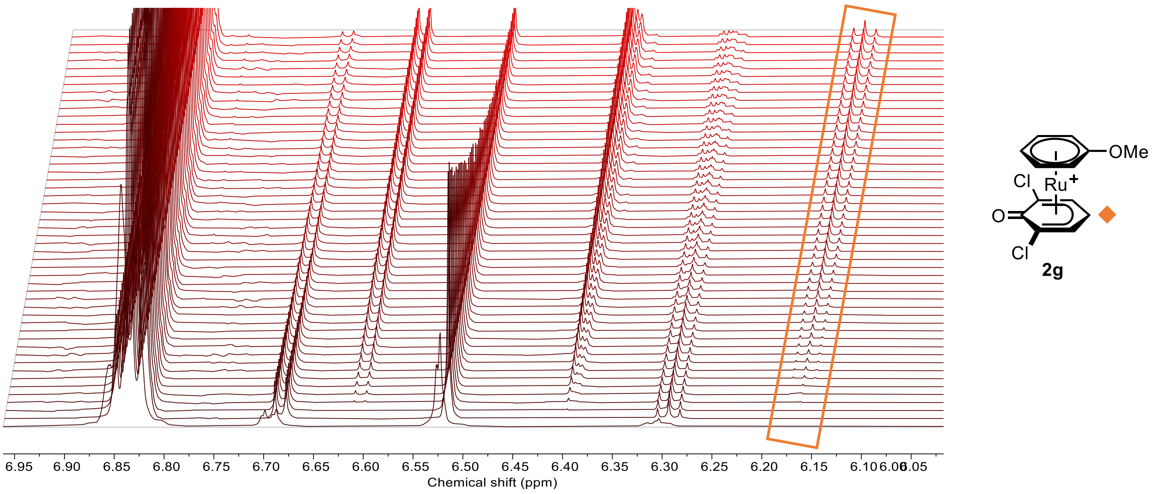

Fig S49: Series of ^{1}H-NMR spectra at 500 MHz, 363 K, MeNO$_2$-d_3 measured in 5 min intervals of **1g** in presence of $^-$OMs and pelargonic acid at 90 °C. Orange box = ^{1}H-NMR signal of **2g**; orange diamond = proton of **2g** responsible for the signal in orange box.

Kinetics for Different Concentrations of $^{-}$OMs

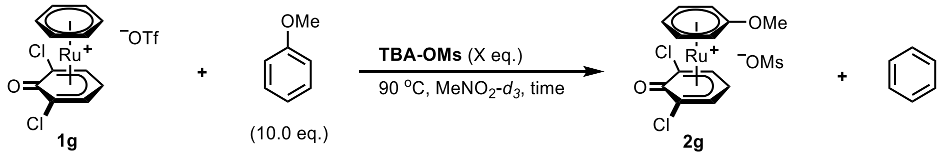

A 5 mm J-Young NMR tube was charged with **1g** (10 mg, 20 µmol, 1.0 equiv.), anisole (22 µL, 22 mg, 0.20 mmol, 10 equiv.) and tetrabutylammonium salt (0-1.85 equiv.). Deuterated nitromethane (0.50 mL, c = 40 mM) was added, the tube was closed and placed in a preheated (90 °C) NMR sample head. ^{1}H NMR spectra were recorded every 5 min at 90 °C for several hours. The rate constants were determined by plotting the ^{1}H-NMR integrals against the reaction time.

Table S3 Equivalents and kinetic values for the arene exchange experiment with $^{-}$OMs:

Equivalents ($^{-}$OMs)	r_{init}(mol% s^{-1})	log (equiv.)	log (r_{init})
0	1.48×10^{-4}		
0,47	8.84×10^{-3}	−0.3279	−2.0537
0,82	1.30×10^{-2}	−0.0862	−1.8847
1,85	2.39×10^{-2}	0.2672	−1.6216

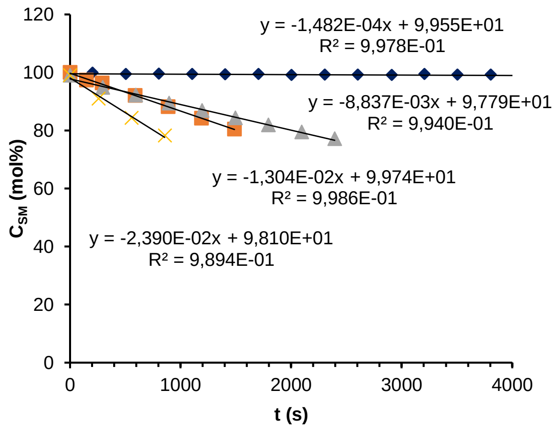

Fig S50: Concentration decrease of benzene complex **1g** with in presence of different concentrations of $^{-}$OMs plotted against the reaction time; blue diamonds = 0 equiv., gray triangles = 0.47 equiv., orange squares = 0.82 equiv., yellow crosses = 1.85 equiv.

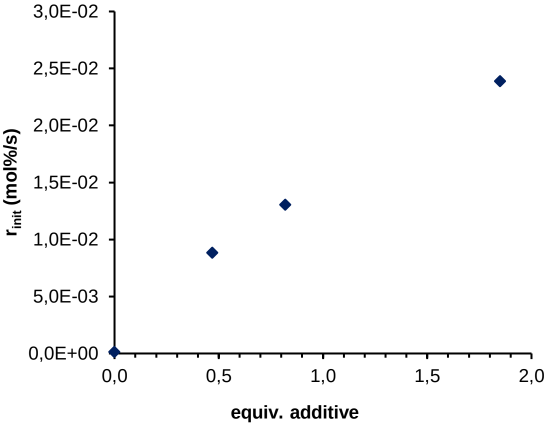

Fig S51: Slopes of concentration plot (Fig S50) plotted against equivalents of ⁻OMs.

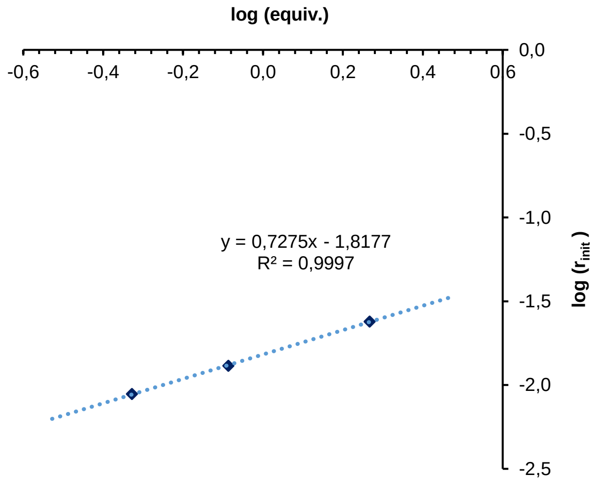

Fig S52: Double logarithmic plot of slopes of concentration plot (Fig S50) and equivalents of ⁻OMs.

Note: The reaction is approximately order 0.73 in ⁻OMs. At higher concentrations, byproducts can be observed from the NMR data. This might influence the kinetics. However, as the initial concentration of **1g** is assumed constant it is just a constant factor in the analysis and should not qualitatively change the result. Changes of the volume of the reaction solution by the addition of TBA-OMs and the formation of byproducts at higher concentrations of ⁻OMs could be reasons for the order being lower than 1.0.

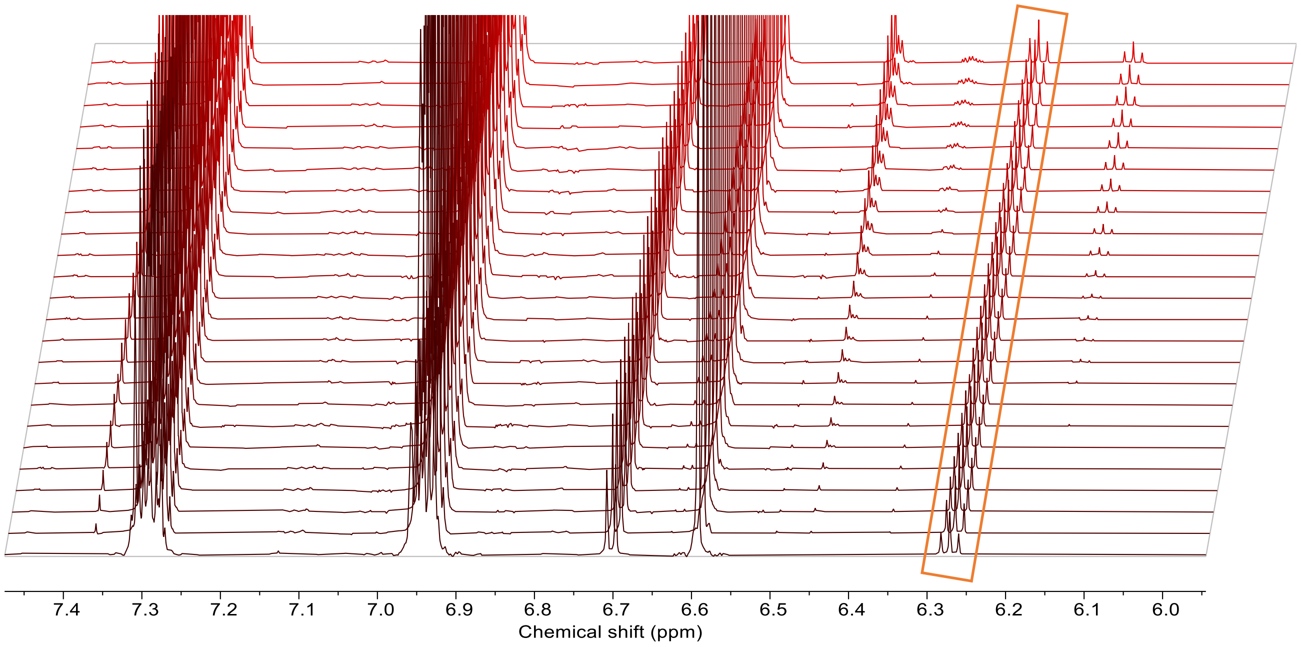

Fig S53: Series of ^{1}H-NMR spectra at 500 MHz, 363 K, $MeNO_2$-d_3 of the exchange of **1g** to **2g** with **0 equiv. of TBA-OMs** measured in 5 min intervals; orange box = signal used for rate determination.

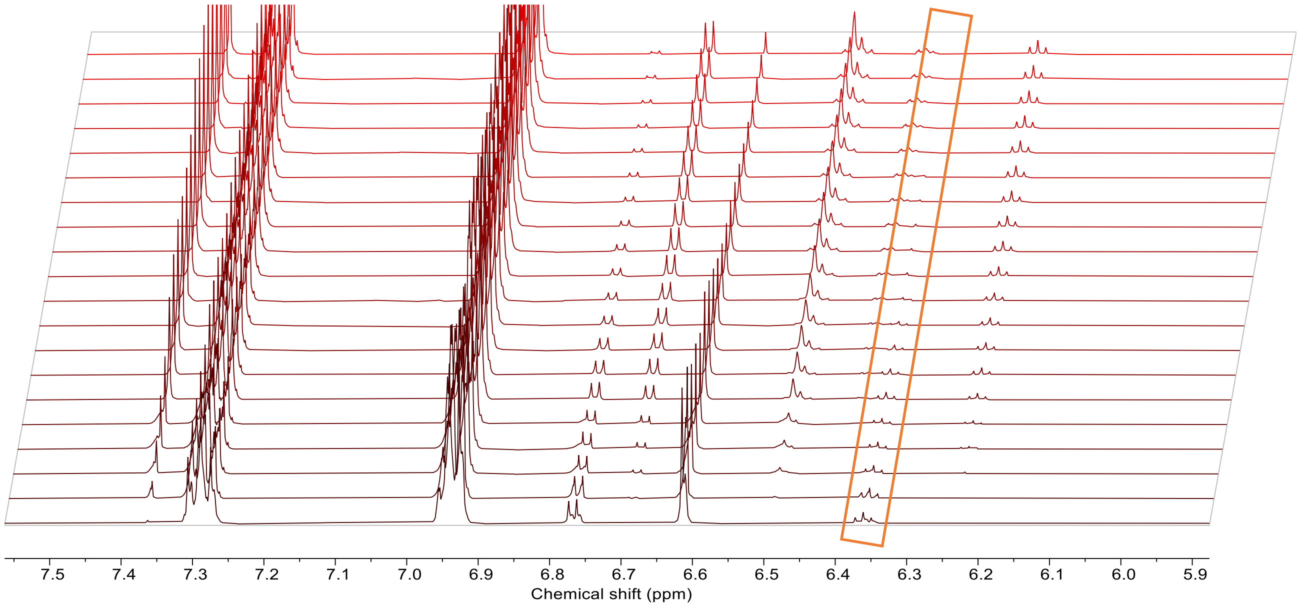

Fig S54: Series of ^{1}H-NMR spectra at 500 MHz, 363 K, $MeNO_2$-d_3 of the exchange of **1g** to **2g** with **0.47 equiv. of TBA-OMs** measured in 5 min intervals; orange box = signal used for rate determination.

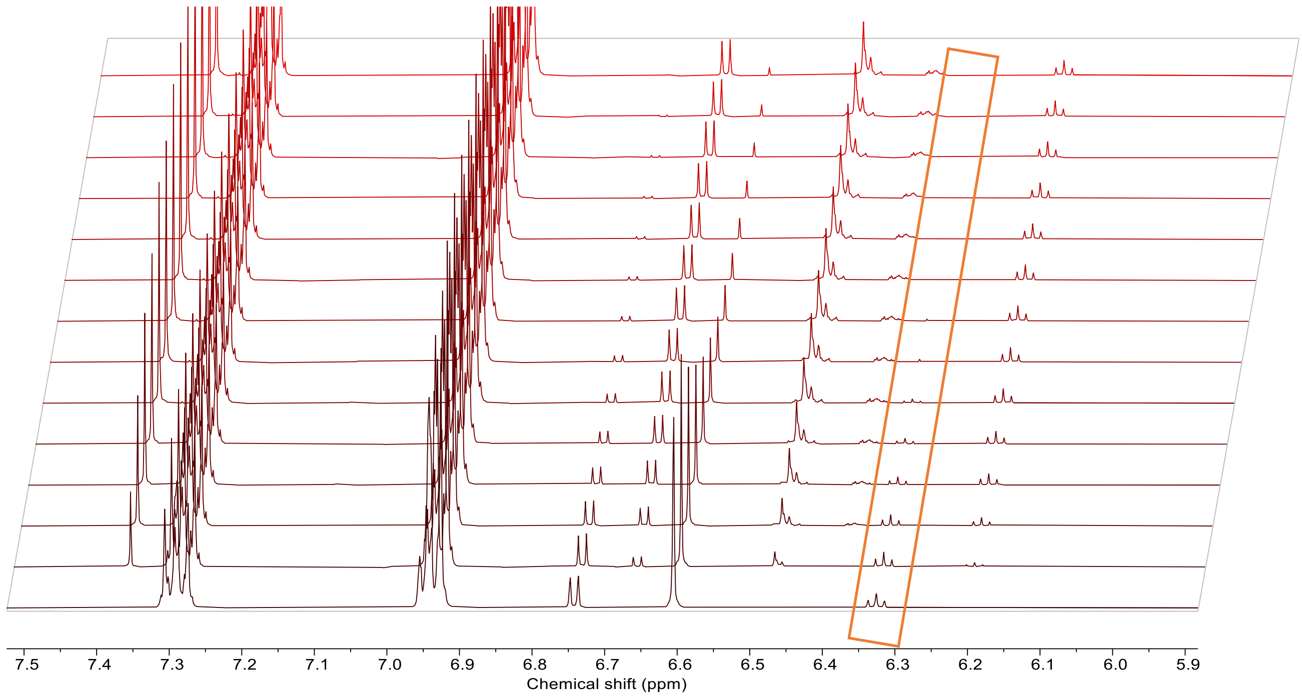

Fig S55: Series of ^{1}H-NMR spectra at 500 MHz, 363 K, MeNO$_2$-d_3 of the exchange of **1g** to **2g** with **0.82 equiv. of TBA-OMs** measured in 5 min intervals; orange box = signal used for rate determination.

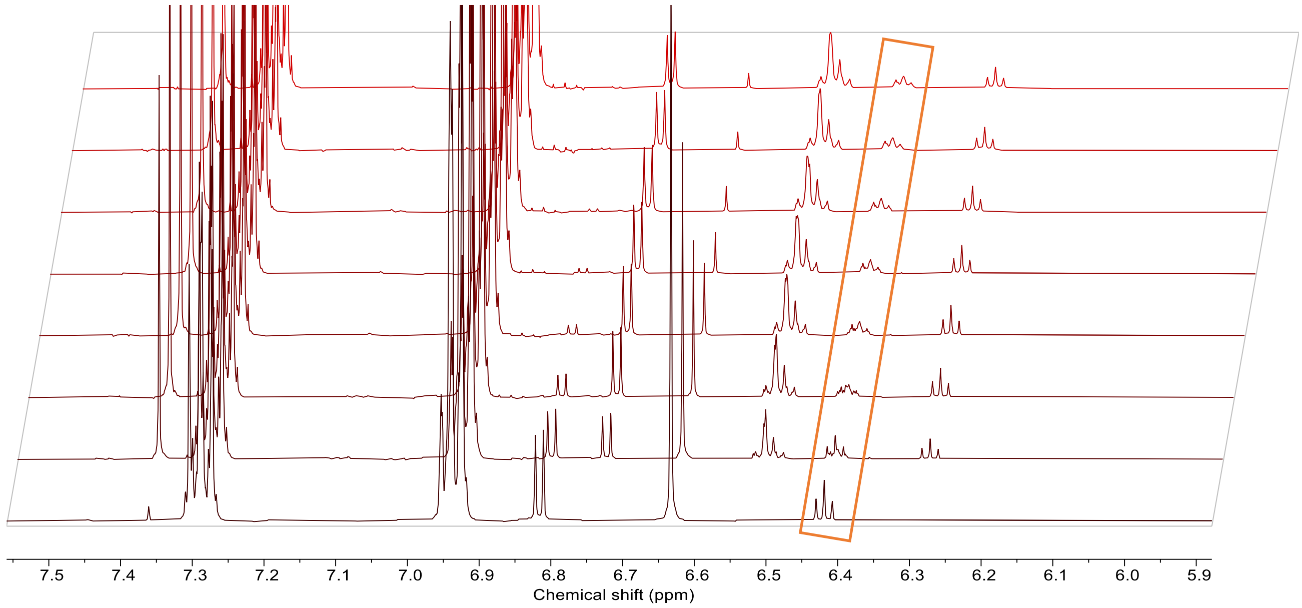

Fig S56: Series of ^{1}H-NMR spectra at 500 MHz, 363 K, MeNO$_2$-d_3 of the exchange of **1g** to **2g** with **1.85 equiv. of TBA-OMs** measured in 5 min intervals; orange box = signal used for rate determination.

Association Constants (Ka)

^{1}H NMR studies were carried out to determine the strength of the interaction between phenoxo complexes and different counterions. Therefore, the association constants (Ka) of different phenoxo complexes with different counterions was determined by ^{1}H NMR titrations. The change in shift of the signals corresponding to the benzene ligand of the phenoxo complexes when varying the concentration was plotted using the BindFit online tool (http://app.supramolecular.org/bindfit/), NMR model 1:1, as described by Thodarson.[6]

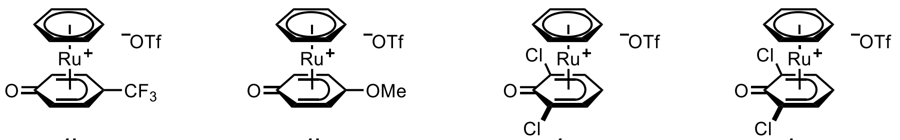

1j $K_a(^{-}OMs) = 11.62\ M^{-1}$ | **1i** $K_a(^{-}OMs) = 7.75\ M^{-1}$ | **1g** $K_a(^{-}OMs) = 9.80\ M^{-1}$ | **1g** $K_a(^{-}OTf) = <1.00\ M^{-1}$

Fig S57: Summary of association constants obtained by ^{1}H NMR titration experiments between phenoxo complexes and anilines.

Association constant (K_a) of 1j with $^{-}$OMs

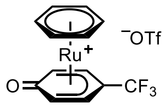

1j

$K_a(^{-}OMs) = 11.62\ M^{-1}$

Under an ambient atmosphere to a 5 mm J-Young NMR tube was added **1j** (9.0 mg, 0.020 mmol, 1.0 equiv.), and $MeNO_2$-d_3 (0.5 mL, *c* = 0.040 M). The NMR tube was inverted several times and the first ^{1}H-NMR spectrum recorded. Meanwhile, a stock solution of TBA-OMs (62 mg, 0.19 mmol) in $MeNO_2$-d_3 (0.5 mL, *c* = 0.38 M) was prepared. 20 µL of the stock solution (0.4 equiv.) was added to the J-Young tube and a ^{1}H-NMR spectrum recorded. The procedure was repeated four times. Subsequently 40 µL of the stock solution (0.8 equiv.) was added to the J-Young tube and a ^{1}H-NMR spectrum recorded. The procedure was repeated five times until saturation was observed.

Concentration 1j (M)	Concentration $^{-}$OMs (M)	Benzene ^{1}H-NMR signal of 1j shift (ppm)
0.037	0.000	6.717
0.035	0.014	6.727
0.034	0.027	6.735
0.033	0.039	6.742
0.032	0.051	6.747
0.031	0.061	6.751
0.029	0.081	6.757
0.027	0.097	6.762
0.026	0.112	6.766
0.024	0.126	6.769
0.023	0.138	6.772

0.022	0.149	6.774

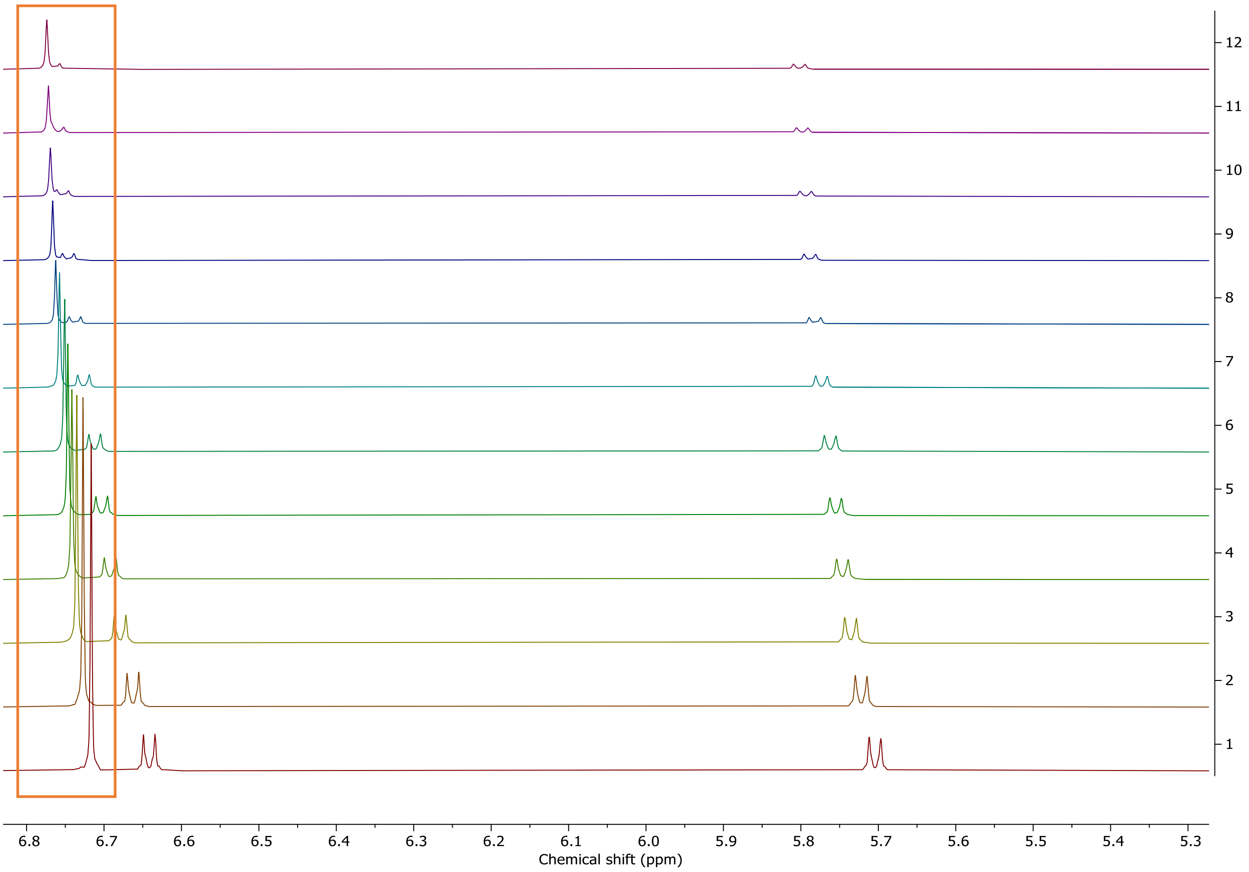

Fig S58: Series of ^{1}H-NMR spectra at 500 MHz, 23 °C, $MeNO_2$-d_3 of **1j** with different concentrations of TBA-OMs; orange box = singlet used for determination of the association constant.

Association constant (K_a) of 1i with $^-$OMs

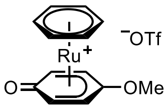

1i

K_a($^-$OMs) = 7.75 M^{-1}

Under an ambient atmosphere to a 5 mm J-Young NMR tube was added **1i** (10 mg, 0.022 mmol, 1.0 equiv.), and $MeNO_2$-d_3 (0.5 mL, c = 0.044 M). The NMR tube was inverted several times and the first ^{1}H-NMR spectrum recorded. Meanwhile, a stock solution of TBA-OMs (75 mg, 0.22 mmol) in $MeNO_2$-d_3 (0.5 mL, c = 0.44 M) was prepared. 20 µL of the stock solution (0.4 equiv.) was added to the J-Young tube and a ^{1}H-NMR spectrum recorded. The procedure was repeated 11 times. Subsequently 40 µL of the stock solution (0.8 equiv.) was added to the J-Young tube and a ^{1}H-NMR spectrum recorded. The procedure was repeated four times until saturation was observed.

Concentration 1i (M)	Concentration $^-$OMs (M)	Benzene ^{1}H-NMR signal of 1i shift (ppm)
0.044	0.000	6.554

0.043	0.017	6.564
0.041	0.033	6.571
0.040	0.048	6.577
0.038	0.061	6.582
0.037	0.074	6.586
0.036	0.086	6.590
0.035	0.097	6.592
0.034	0.108	6.596
0.033	0.118	6.598
0.032	0.127	6.600
0.031	0.136	6.601
0.030	0.144	6.603
0.029	0.152	6.605
0.028	0.167	6.607
0.026	0.180	6.610
0.025	0.192	6.612

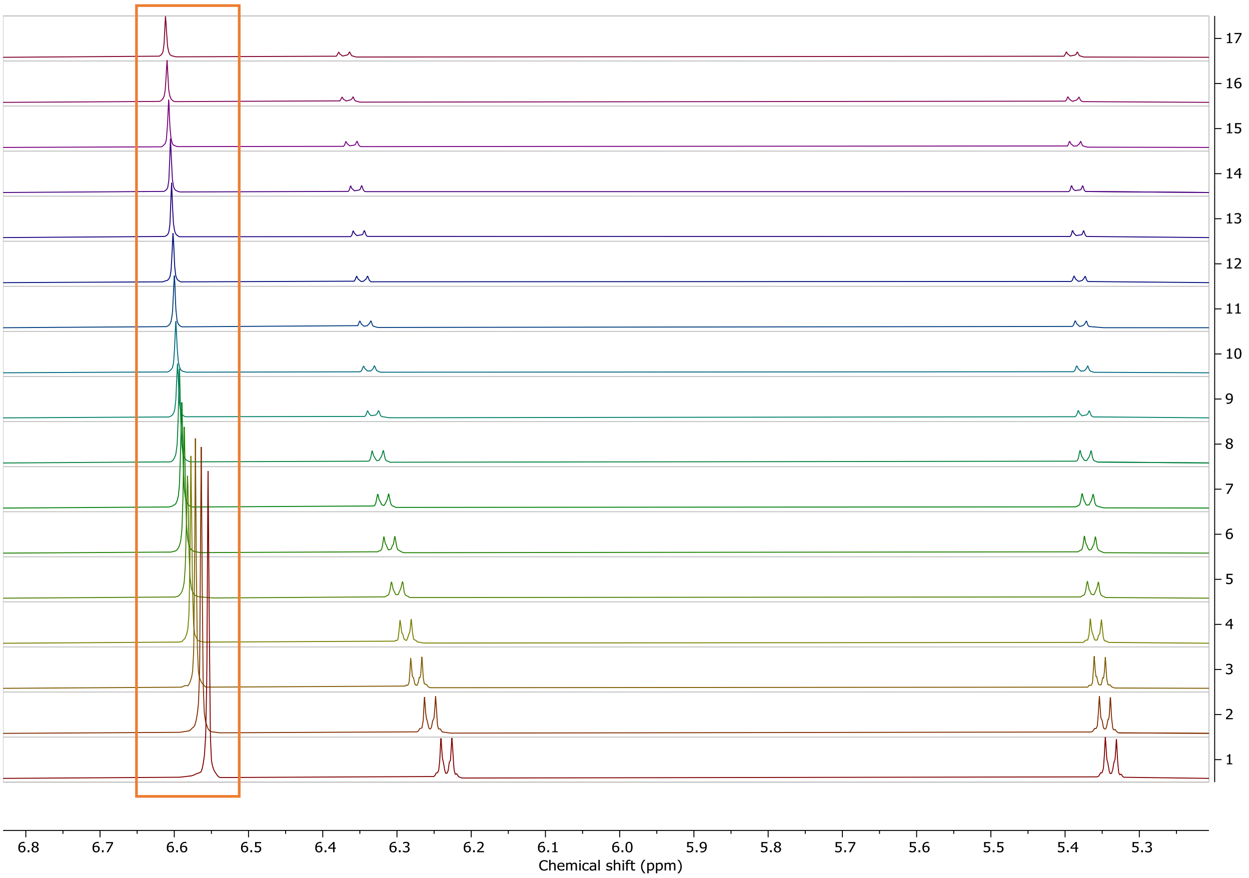

Fig S59: Series of ^{1}H-NMR spectra at 500 MHz, 23 °C, MeNO$_2$-*d*$_3$ of **1i** with different concentrations of TBA-OMs; orange box = singlet used for determination of the association constant.

Association constant (K_a) of 1g with $^{-}$OMs

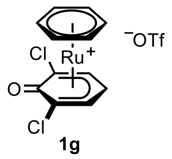

1g

K_a($^{-}$OMs) = 9.80 M^{-1}

Under an ambient atmosphere to a 5 mm J-Young NMR tube was added **1g** (12 mg, 0.025 mmol, 1.0 equiv.), and $MeNO_2$-d_3 (0.5 mL, c = 0.05 M). The NMR tube was inverted several times and the first ^{1}H-NMR spectrum recorded. Meanwhile, a stock solution of TBA-OMs (84 mg, 0.25 mmol) in $MeNO_2$-d_3 (0.5 mL, c = 0.5 M) was prepared. 20 µL of the stock solution (0.4 equiv.) was added to the J-Young tube and a ^{1}H-NMR spectrum recorded. The procedure was repeated four times. Subsequently 40 µL of the stock solution (0.8 equiv.) was added to the J-Young tube and a ^{1}H-NMR spectrum recorded. The procedure was repeated five times until saturation was observed.

Concentration 1g (M)	Concentration $^{-}$OMs (M)	Benzene ^{1}H-NMR signal of 1g shift (ppm)
0.050	0.000	6.628
0.048	0.019	6.638
0.046	0.037	6.647
0.045	0.054	6.650
0.043	0.069	6.659
0.042	0.083	6.664
0.039	0.109	6.670
0.037	0.132	6.674
0.035	0.153	6.678
0.033	0.171	6.680
0.031	0.188	6.682

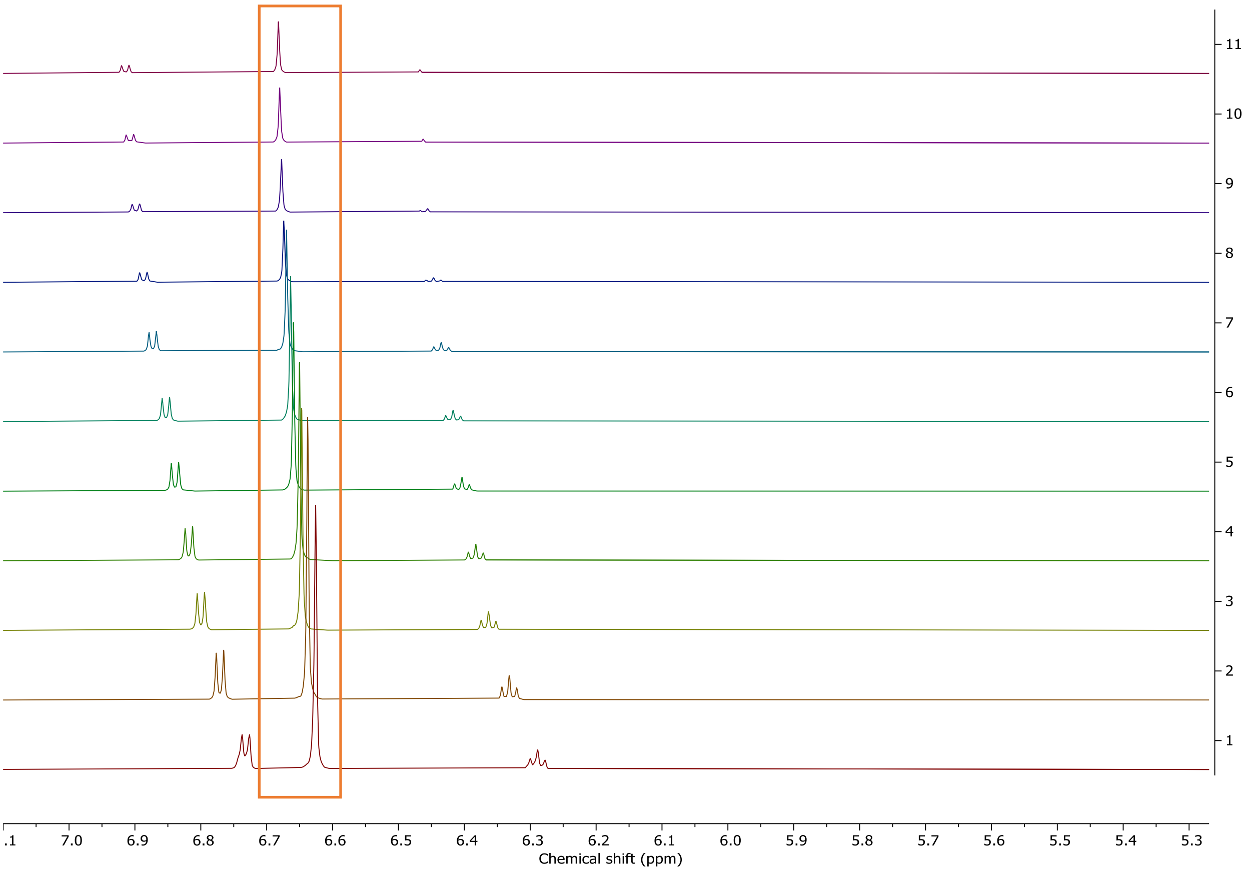

Fig S60: Series of ^{1}H-NMR spectra at 500 MHz, 23 °C, MeNO$_2$-*d*$_3$ of **1g** with different concentrations of TBA-OMs; orange box = singlet used for determination of the association constant.

Association constant (K_a) of 1g with $^-$OTf

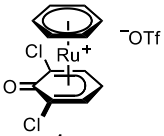

1g

K_a($^-$OTf) = <1.00 M^{-1}

Under an ambient atmosphere to a 5 mm J-Young NMR tube was added **1g** (12 mg, 0.025 mmol, 1.0 equiv.), and MeNO$_2$-*d*$_3$ (0.5 mL, *c* = 0.05 M). The NMR tube was inverted several times and the first ^{1}H-NMR spectrum recorded. Meanwhile, a stock solution of TBA-OTf (98 mg, 0.25 mmol) in MeNO$_2$-*d*$_3$ (0.5 mL, *c* = 0.5 M) was prepared. 20 µL of the stock solution (0.4 equiv.) was added to the J-Young tube and a ^{1}H-NMR spectrum recorded. The procedure was repeated four times. Subsequently 40 µL of the stock solution (0.8 equiv.) was added to the J-Young tube and a ^{1}H-NMR spectrum recorded. The procedure was repeated five times until saturation was observed.

Concentration 1g (M)	Concentration $^-$OTf (M)	Benzene ^{1}H-NMR signal of 1g shift (ppm)
0.050	0.050	6.628
0.048	0.067	6.628

0.046	0.083	6.629
0.045	0.098	6.630
0.043	0.112	6.630
0.042	0.125	6.630
0.039	0.148	6.631
0.037	0.169	6.632
0.035	0.188	6.632
0.033	0.204	6.632
0.031	0.219	6.632

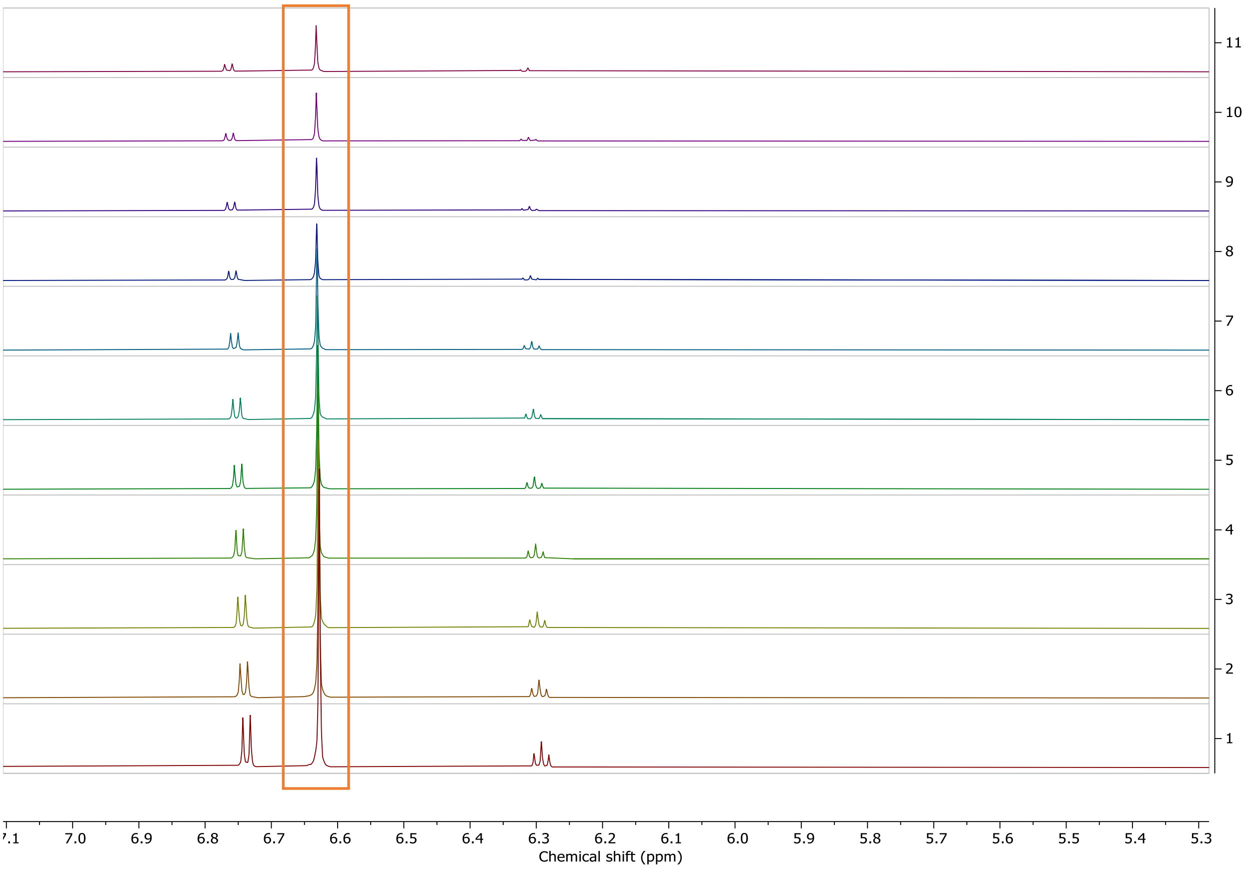

Fig S61: Series of ^{1}H-NMR spectra at 500 MHz, 23 °C, $MeNO_2$-d_3 of **1g** with different concentrations of TBA-OTf; orange box = singlet used for determination of the association constant.

Medium Effect Experiment

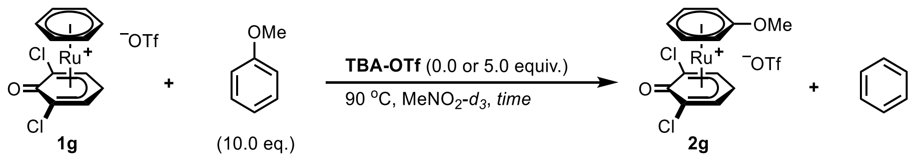

A 5 mm J-Young NMR tube was charged with **1g** (10 mg, 20 µmol, 1.0 equiv.), anisole (22 µL, 22 mg, 0.20 mmol, 10 equiv.) and TBA-OTf (0 equiv; or 40 mg, 0.10 mmol, 5.0 equiv.). Deuterated nitromethane (0.50 mL, *c* = 40 mM) was added and the initial ^{1}H NMR spectrum was measured. Subsequently, the tube was placed in a preheated (90 °C) NMR sample head. ^{1}H NMR spectra were recorded every 5 min at 90 °C for several hours. The rate constants were determined by plotting of the conversion in mol%, which was extracted from the ^{1}H-NMR integrals, against the reaction time.

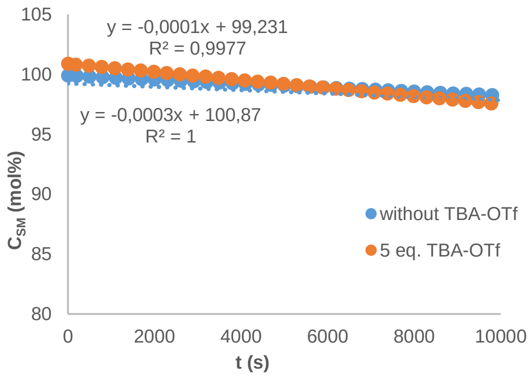

Fig S62: Plot of decreasing concentration of **1g** against the reaction time.

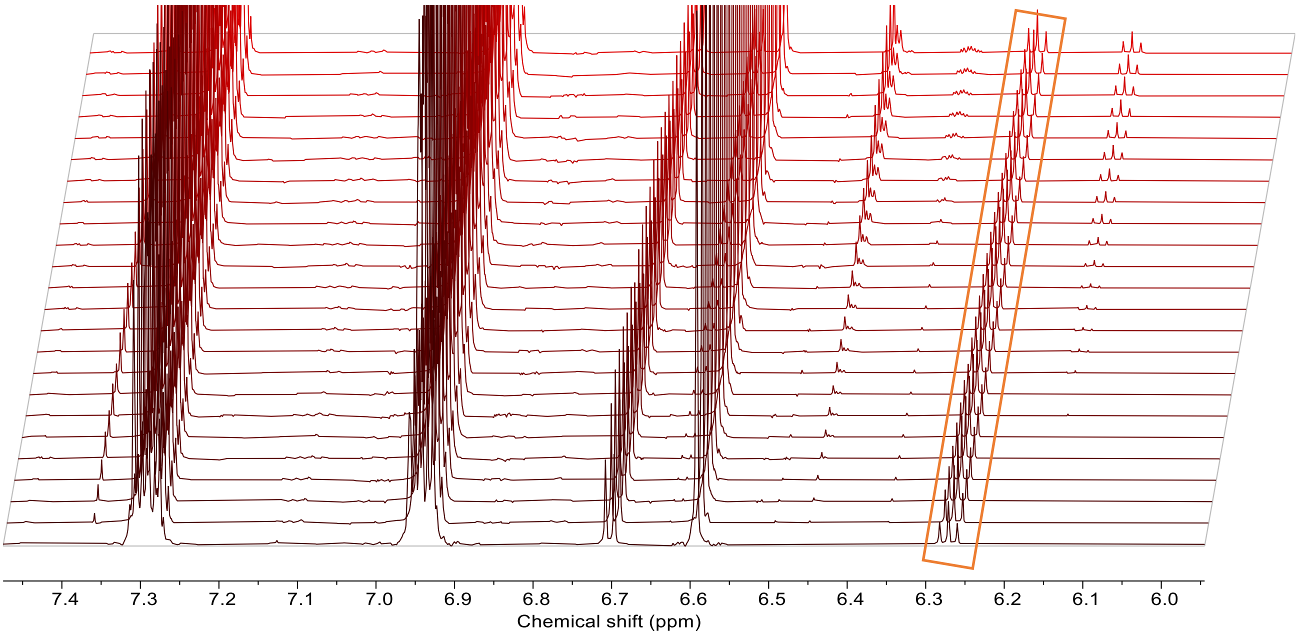

Fig S63: Series of ^{1}H-NMR spectra at 500 MHz, 363 K, $MeNO_2$-d_3 of the exchange of **1g** to **2g** with **0 equiv. of TBA-OTf** measured in 5 min intervals; orange box = signal used for rate determination.

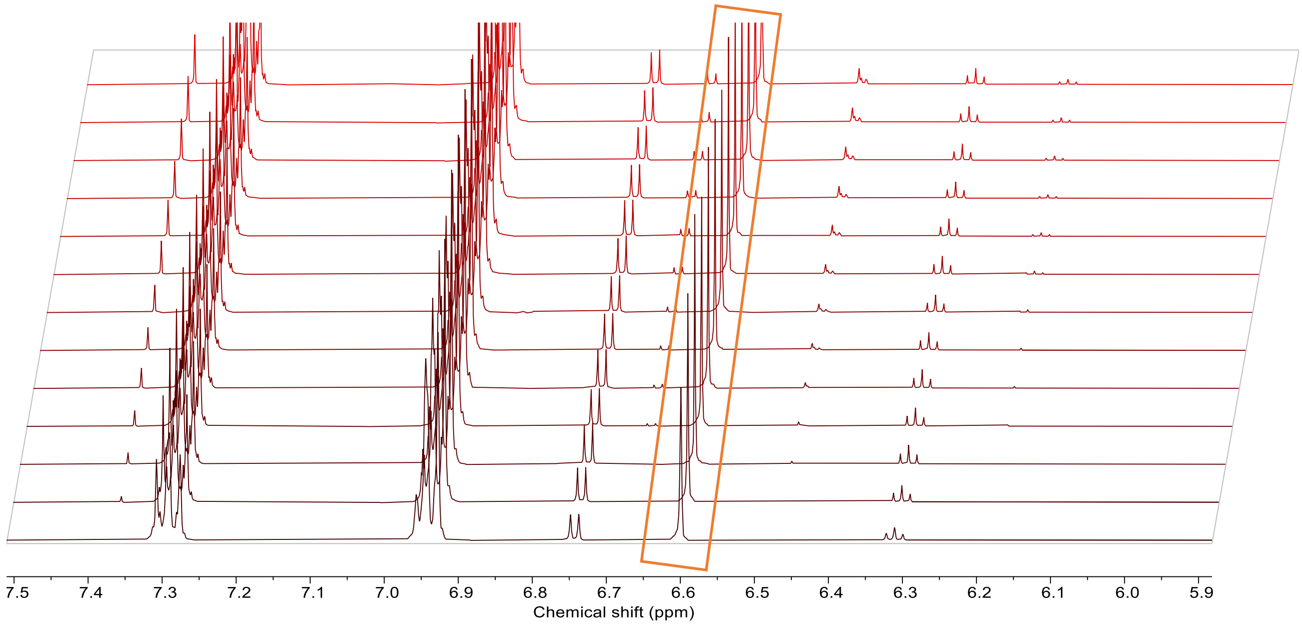

Fig S64: Series of ^{1}H-NMR spectra at 500 MHz, 363 K, $MeNO_2$-d_3 of the exchange of **1g** to **2g** with **5.0 equiv. of TBA-OTf** measured in 5 min intervals; orange box = signal used for rate determination.

Arylation of Alcohols with Aryl Fluorides Catalyzed by 1a

Phenyl-epiandrosterone ester (4a)

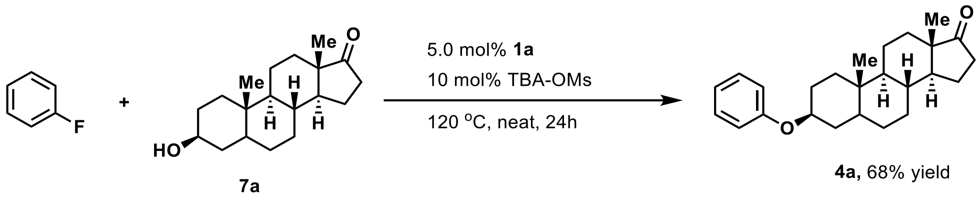

7a **4a,** 68% yield

A 4 mL borosilicate vial equipped with a Teflon-coated magnetic stirring bar was charged with [η^6-benzene-η^5-(2,6-dibromo-4-methoxy-1-phenoxo)Ru](OTf) **1a** (6.0 mg, 10 µmol, 5.0 mol%), Epiandrosterone **7a** (58 mg, 0.20 mmol, 1.0 equiv.), and tetrabutylammonium methylsulfonate (TBA-OMs) (7 mg, 20 µmol, 10 mol%). The vial was connected via a vial adapter to a Schlenk line and was evacuated and purged with argon three times. Fluorobenzene (0.40 mL, 41 mg, 4.3 mmol, 21 equiv.) was then added, the adapter closed, and the suspension stirred at 120 °C for 24 h under an argon atmosphere. Subsequently, the mixture was filtered through a thin celite layer (2.0 cm) loaded on a glass pipette followed by washing with DCM (3 × 1 mL). Volatiles were removed from the reaction mixture under reduced pressure and the residue was purified by column chromatography on silica gel eluting with a solvent mixture of hexanes / ethyl acetate (100:0 gradient to 90:10 (v/v)) to afford 50 mg, (14 mmol, 68% yield) of desired product **4a** as a colorless solid.

R_f = 0.39 (hexanes:ethyl acetate, 5:1, (v:v)).

NMR Spectroscopy:

^{1}H NMR (500 MHz, $CDCl_3$, 23 °C, δ): 7.33 – 7.19 (m, 2H), 6.97 – 6.83 (m, 3H), 4.19 (tt, *J* = 11.1, 4.7 Hz, 1H), 2.44 (dd, *J* = 19.3, 8.8 Hz, 1H), 2.13 – 1.88 (m, 3H), 1.85 – 1.74 (m, 4H), 1.72 – 1.42 (m, 5H), 1.41 – 1.16 (m, 6H), 1.12 – 0.94 (m, 2H), 0.88 (d, *J* = 10.7 Hz, 6H), 0.78 – 0.67 (m, 1H).

^{13}C NMR (126 MHz, $CDCl_3$, 23 °C, δ): 221.3, 157.9, 129.6, 120.7, 116.2, 76.7, 54.6, 51.6, 47.9, 45.0, 37.0, 36.0, 36.0, 35.2, 34.5, 31.7, 31.0, 28.5, 28.1, 21.9, 20.6, 14.0, 12.5.

HRMS API (m/z) calculated for $C_{25}H_{34}O_2$ $[M]^+$, 366.2553; found, 366.2554 deviation: −0.2 ppm.

Phenyl-epiandrosterone ester (4a) with 1-Cp* as Catalyst

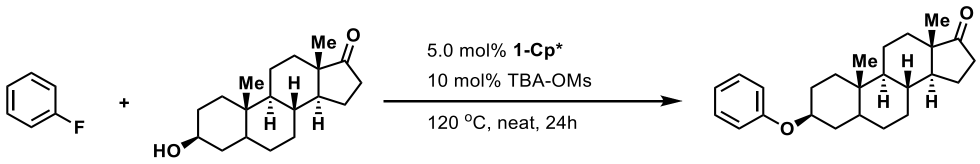

7a **4a,** n.r.

A 4 mL borosilicate vial equipped with a Teflon-coated magnetic stirring bar was charged with [η^6-benzene-η^5-(pentamethylcyclopentadienyl)Ru](OTf) **1-Cp*** (3.9 mg, 10 µmol, 5.0 mol%), Epiandrosterone **7a** (58 mg, 0.20 mmol, 1.0 equiv.), and tetrabutylammonium methylsulfonate (TBA-OMs) (7.0 mg, 20 µmol, 10 mol%). The vial was connected via a vial adapter to a Schlenk line and was evacuated and purged with argon three times. Fluorobenzene (0.40 mL, 41 mg, 4.3 mmol, 21

equiv.) was then added, the adapter closed, and the suspension stirred at 120 °C for 24 h under an argon atmosphere. After completion, trimethoxybenzene (11 mg, 67 µmol, 33 mol%) was added as internal standard. <5 % ^{1}H-NMR yield of **4a** was observed via ^{1}H-NMR.

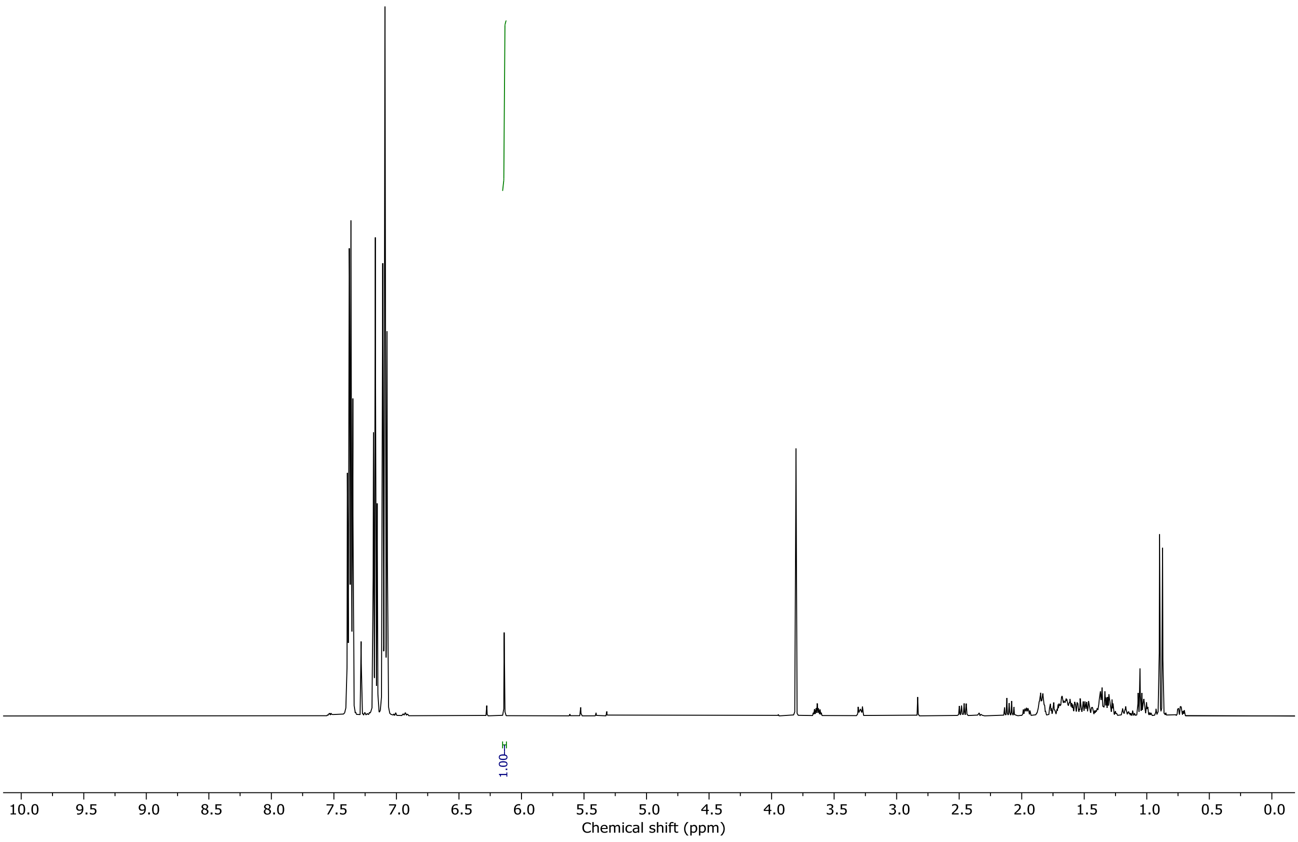

Fig S65: ^{1}H-NMR spectrum after the reaction; 500 MHz, 23 °C, $CDCl_3$.

Phenyl-epiandrosterone ester (4a) with RhCp* as Catalyst

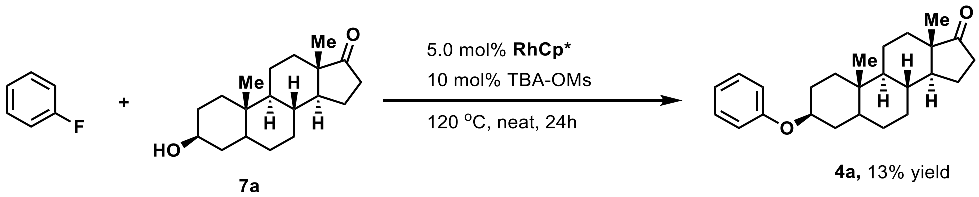

A 4 mL borosilicate vial equipped with a Teflon-coated magnetic stirring bar was charged with [η^6-anisole-η^5-(pentamethylcyclopentadienyl)Rh]$(OTf)_2$ **RhCp*** (6.1 mg, 10 µmol, 5.0 mol%), Epiandrosterone **7a** (58 mg, 0.20 mmol, 1.0 equiv.), and tetrabutylammonium methylsulfonate (TBA-OMs) (7.0 mg, 20 µmol, 10 mol%). The vial was connected via a vial adapter to a Schlenk line and was evacuated and purged with argon three times. Fluorobenzene (0.40 mL, 41 mg, 4.3 mmol, 21 equiv.) was then added, the adapter closed, and the suspension stirred at 120 °C for 24 h under an argon atmosphere. After completion, trimethoxybenzene (11 mg, 67 µmol, 33 mol%) was added as internal standard. 13 % NMR yield of **4a** was observed via ^{1}H-NMR spectroscopy by comparing the integrals of the standard signal at 6.11 ppm to the integrals of the signal of **4a** at 4.09 ppm.

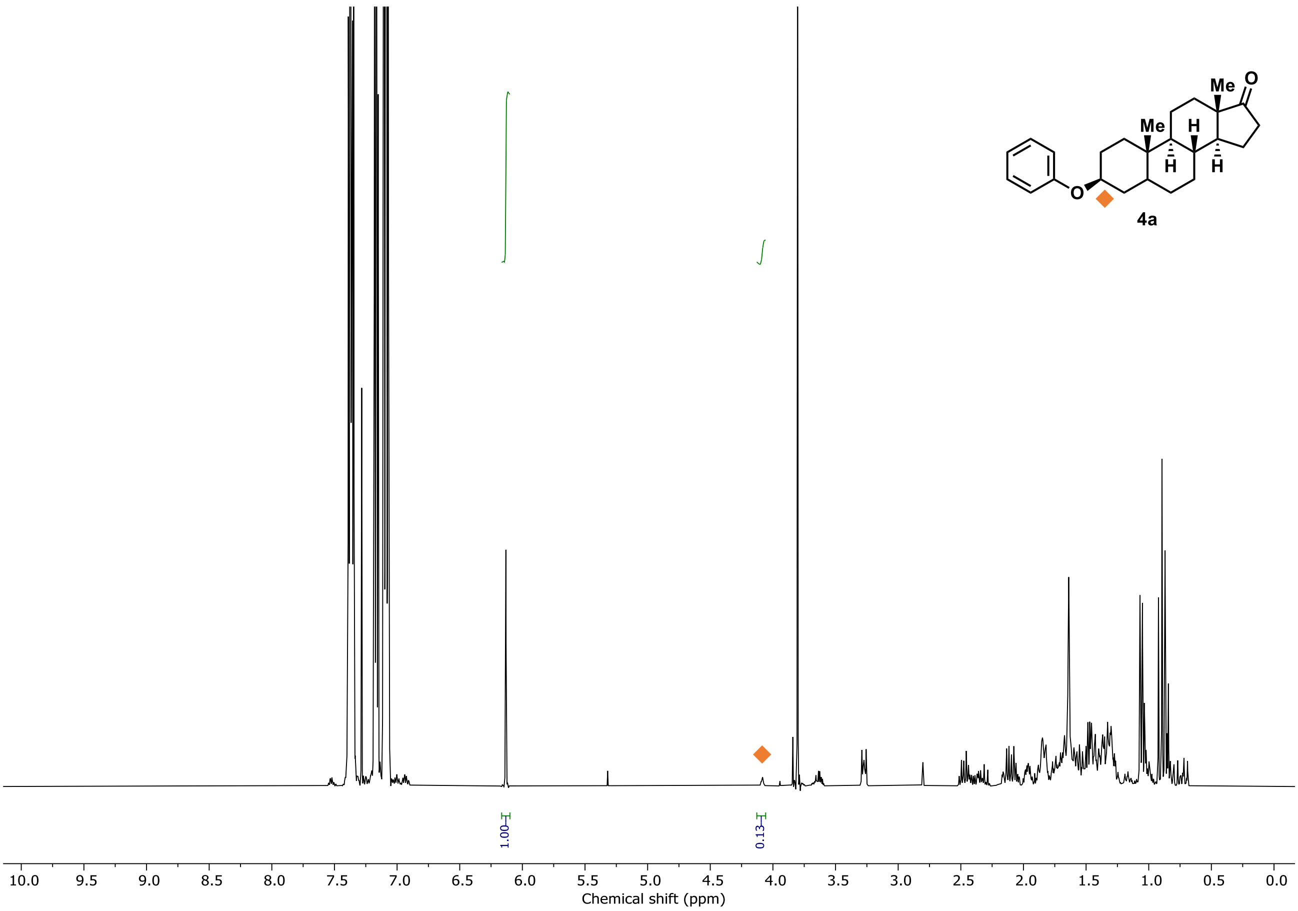

Fig S66: ^{1}H-NMR spectrum after the reaction; 500 MHz, 23 °C, $CDCl_3$; orange diamond: signal of **4a**.

1-Nitro-4-(phenoxymethyl)benzene (4b)

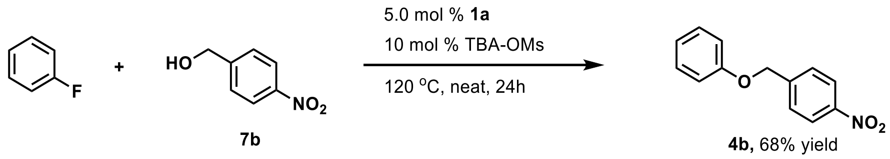

A 4 mL borosilicate vial equipped with a Teflon-coated magnetic stirring bar was charged with [η^6-benzene-η^5-(2,6-dibromo-4-methoxy-1-phenoxo)Ru](OTf) **1a** (6.0 mg, 10 µmol, 5.0 mol%), 4-nitrobenzylalcohol **7b** (31 mg, 0.20 mmol, 1.0 equiv.), and tetrabutylammonium methylsulfonate (TBA-OMs) (7.0 mg, 20 µmol, 10 mol%). The vial was connected via a vial adapter to a Schlenk line and was evacuated and purged with argon three times. Fluorobenzene (0.40 mL, 41 mg, 4.3 mmol, 21 equiv.) was then added, the adapter closed, and the suspension stirred at 120 °C for 24 h under an argon atmosphere. Subsequently, the mixture was filtered through a thin celite layer (2.0 cm) loaded on a glass pipette followed by washing with DCM (3 × 1 mL). Volatiles were removed from the reaction mixture under reduced pressure and the residue was purified by column chromatography on silica gel eluting with a solvent mixture of hexanes / ethyl acetate (100:0 gradient to 90:10 (v/v)) to afford 21 mg (0.14 mmol, 68% yield) of desired product **4b** as a colorless solid.

R*f* = 0.40 (hexanes:ethyl acetate, 5:1, (v:v)).

NMR Spectroscopy:

^{1}H NMR (500 MHz, $CDCl_3$, 23 °C, δ): 8.30 – 8.20 (m, 2H), 7.67 – 7.57 (m, 2H), 7.38 – 7.28 (m, 2H), 7.07 – 6.92 (m, 3H), 5.18 (s, 2H).

^{13}C NMR (126 MHz, $CDCl_3$, 23 °C, δ): 158.2, 147.7, 144.7, 129.8, 127.7, 123.9, 121.7, 114.9, 68.7.

HRMS EI (m/z) calculated for $C_{13}H_{11}N_1O_3$ $[M]^+$, 229.0733; found, 229.0737 deviation: −1.4 ppm.

(Cyclohexylmethoxy)benzene (4c)

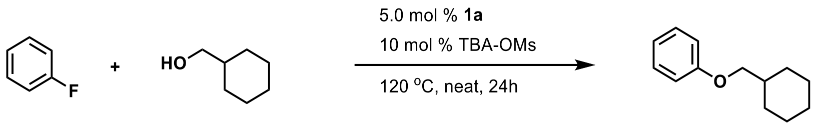

7c

4c, 87% yield

A 4 mL borosilicate vial equipped with a Teflon-coated magnetic stirring bar was charged with [η^6-benzene-η^5-(2,6-dibromo-4-methoxy-1-phenoxo)Ru](OTf) **1a** (6.0 mg, 10 µmol, 5.0 mol%), cyclohexylmethanol **7c** (31 mg, 0.20 mmol, 1.0 equiv.), and tetrabutylammonium methylsulfonate (TBA-OMs) (7.0 mg, 20 µmol, 10 mol%). The vial was connected via a vial adapter to a Schlenk line and was evacuated and purged with argon three times. Fluorobenzene (0.40 mL, 41 mg, 4.3 mmol, 21 equiv.) was then added, the adapter closed, and the suspension stirred at 120 °C for 24 h under an argon atmosphere. Subsequently, the mixture was filtered through a thin celite layer (2.0 cm) loaded on a glass pipette followed by washing with DCM (3 × 1 mL). Volatiles were removed from the reaction mixture under reduced pressure and the residue was purified by column chromatography on silica gel eluting with a solvent mixture of hexanes / ethyl acetate (100:0 gradient to 98:2 (v/v)) to afford 34 mg (0.17 mmol, 87% yield) of desired product **4c** as a colorless oil.

R_f = 0.25 (hexanes).

NMR Spectroscopy:

^{1}H NMR (500 MHz, $CDCl_3$, 23 °C, δ): 7.35 – 7.24 (m, 2H), 7.00 – 6.86 (m, 3H), 3.77 (d, J = 6.4 Hz, 2H), 1.96 – 1.86 (m, 2H), 1.87 – 1.67 (m, 4H), 1.40 – 1.15 (m, 3H), 1.15 – 0.97 (m, 2H).

^{13}C NMR (126 MHz, $CDCl_3$, 23 °C, δ): 159.4, 129.5, 120.5, 114.6, 73.5, 37.8, 30.1, 26.7, 26.0.

HRMS EI (m/z) calculated for $C_{13}H_{18}O_3$ $[M]^+$, 190.1352; found, 190.1352 deviation: −0.13 ppm.

Mechanistic Investigation and Optimization

Proposed catalytic cycle

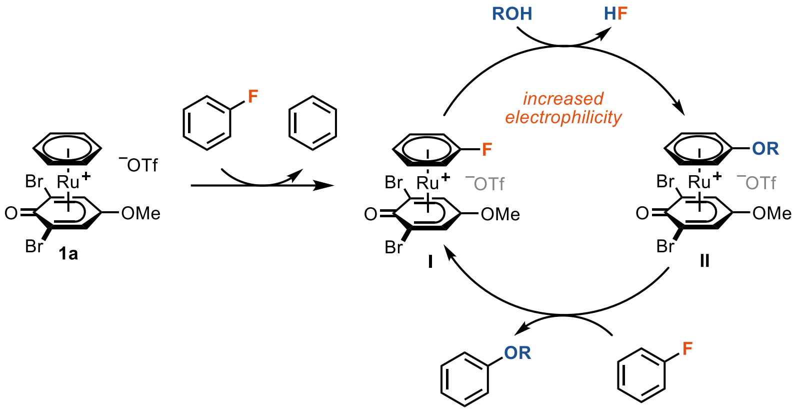

Fig S67: Proposed catalytic cycle for the arylation of alcohols with **1a**.

Optimization

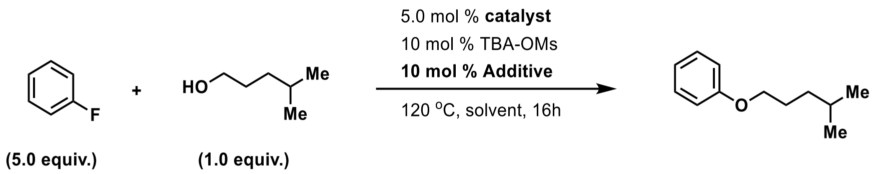

A 4 mL borosilicate vial equipped with a Teflon-coated magnetic stirring bar was charged with **catalyst** (10 µmol, 5.0 mol%), isohexanol (23 ul, 18 mg, 0.20 mmol, 1.0 equiv.), additive (20 µmol, 10 mol%), and tetrabutylammonium methylsulfonate (TBA-OMs) (7.0 mg, 20 µmol, 10 mol%). The vial was connected via a vial adapter to a Schlenk line and was evacuated and purged with argon three times. Fluorobenzene (0.40 mL, 41 mg, 4.3 mmol, 21 equiv.) was then added, the adapter closed, and the suspension stirred at 120 °C for 16 h under an argon atmosphere. Subsequently, $CDCl_3$ (0.5 mL) and CH_2Br_2 as internal standard were added. NMR yield was determined via ^{1}H-NMR spectroscopy by comparing the integrals of the standard signal at 4.96 ppm to the integrals of the signal at 3.98 ppm.

Table S4 Results for the optimization of the arylation of alcohols with different catalysts, NMR yield was determined via ^{1}H-NMR spectroscopy by comparing the integrals of the standard signal at 4.96 ppm to the integrals of the signal at 3.98 ppm:

Catalyst	Solvent	Additive	Result
**None**	Neat PhF	-	<5% yield
**SI-8**	Neat PhF	-	75% yield
**SI-8**	DCE	-	34% yield
**1g**	DCE	pyridine	3% yield
**1g**	DCE	PhCN	15% yield

**1g**	DCE	MeCN	16% yield
**1g**	DCE	*t*-BuCN	16% yield

Stoichiometric arene exchange experiments

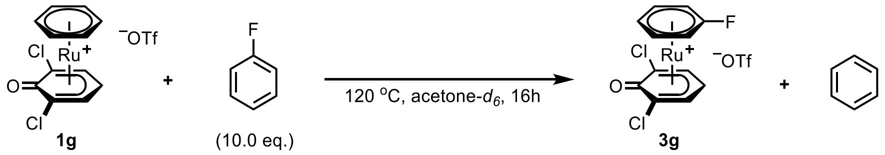

A 5 mm J-Young NMR tube was charged with **1g** (10 mg, 20 µmol, 1.0 equiv.) and fluorobenzene (19 µL, 19 mg, 0.20 mmol, 10 equiv.). Deuterated acetone (0.5 mL, c = 40 mM) was added, the tube inverted several times, and the initial ^{1}H NMR spectrum was measured. After the initial measurement, the tube was placed in an 120 ˚C oil bath and was heated for 16 h. Then, the ^{1}H NMR and ^{19}F NMR spectrum was measured for determination of the conversion of **1g** to **3g**. Conversion ~53%.

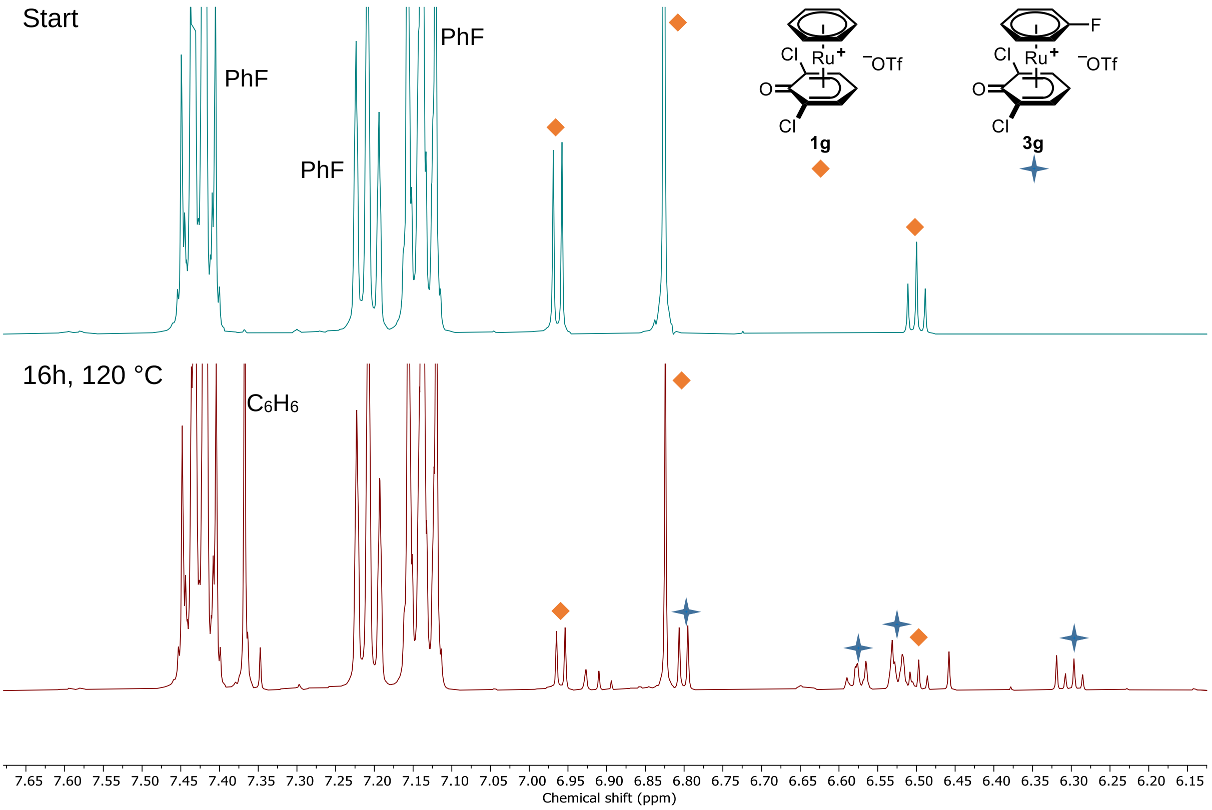

Fig S68: Series of ^{1}H-NMR spectra at 500 MHz, 23 °C, Acetone-d_6; **top:** initial ^{1}H NMR spectrum; **bottom:** ^{1}H NMR spectrum after heating at 120 °C for 16 h. Conversion to **3g** ~53%.

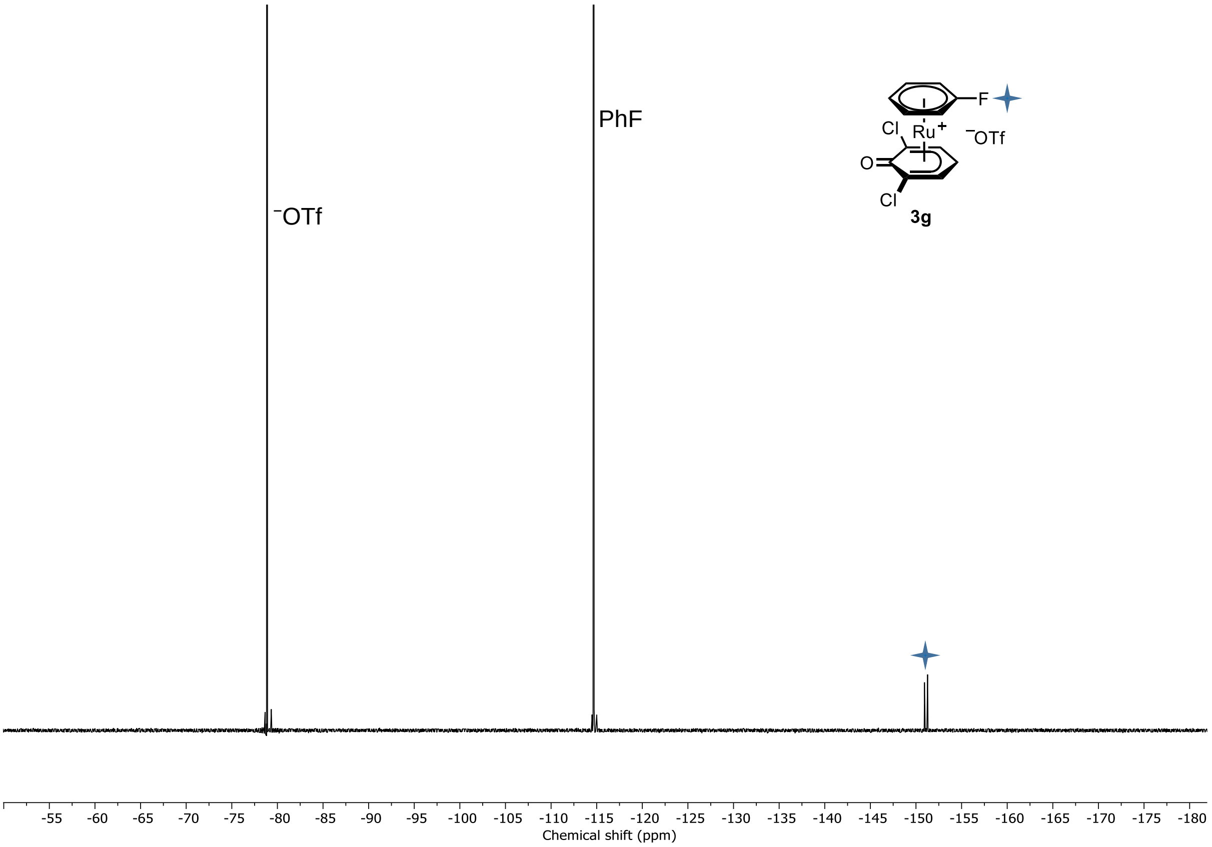

Fig S69: ^{19}F-NMR spectra at 565 MHz, 23 °C, Acetone-d_6; after heating at 120 °C for 16 h.

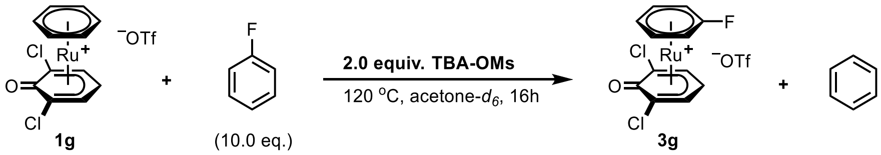

A 5 mm J-Young NMR tube was charged with **1g** (10 mg, 20 µmol, 1.0 equiv.), TBA-OMs (14 mg, 40 µmol, 2.0 equiv.), and fluorobenzene (19 µL, 19 mg, 0.20 mmol, 10 equiv.). Deuterated acetone (0.5 mL, c = 40 mM) was added, the tube inverted several times, and the initial ^{1}H NMR spectrum was measured. After the initial measurement, the tube was placed in an 120 ˚C oil bath and was heated for 16 h. Then, the ^{1}H NMR spectrum was measured for determination of the conversion of **1g** to **3g**.

Note: mixture heterogeneous at the beginning of the reaction. Not all solids solubilized due to the addition of large excess of fluorobenzene. Formation of multiple species during the reaction.

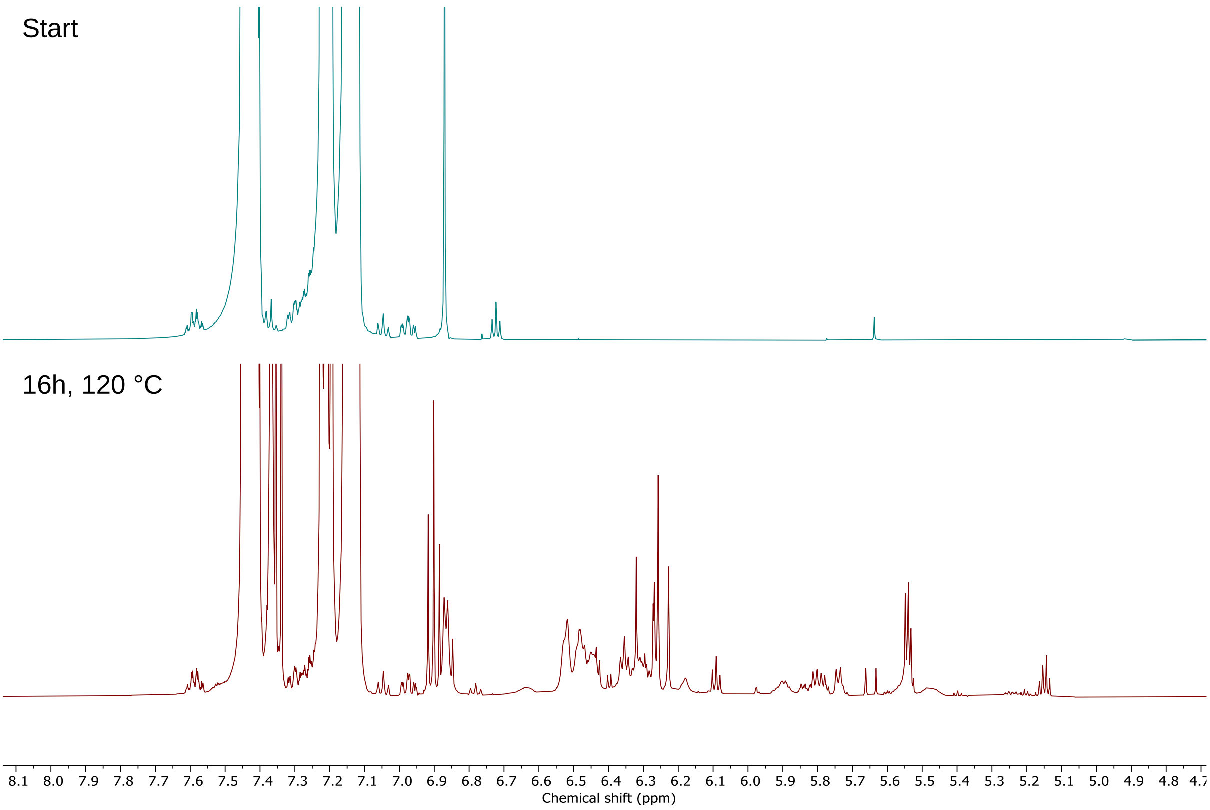

Fig S70: Series of ^{1}H-NMR spectra at 500 MHz, 23 °C, Acetone-d_6; **top:** initial ^{1}H NMR spectrum; **bottom:** ^{1}H NMR spectrum after heating at 120 °C for 16 h.

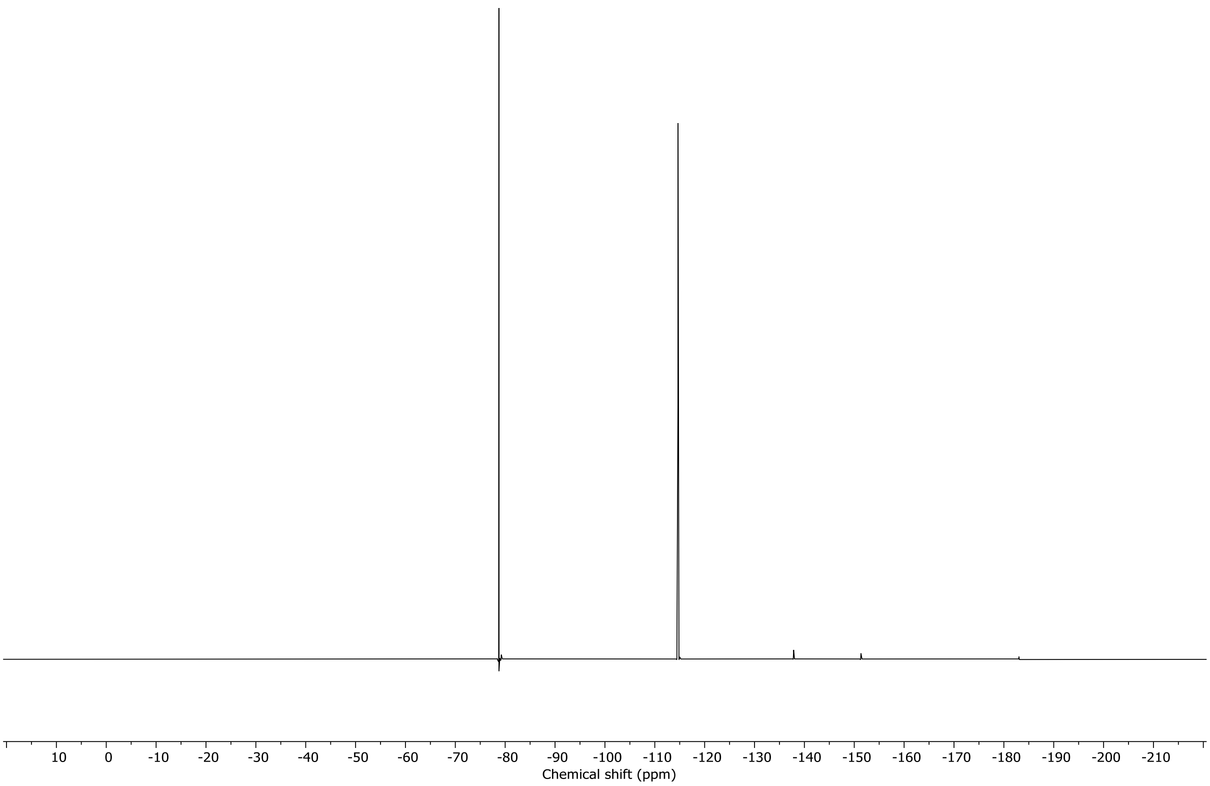

Fig S71: ^{19}F-NMR spectra at 565 MHz, 23 °C, Acetone-d_6; after heating at 120 °C for 16 h.

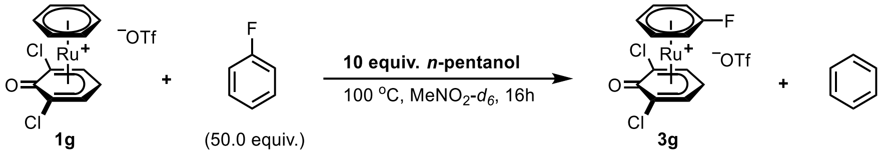

A 5 mm J-Young NMR tube was charged with **1g** (10 mg, 20 µmol, 1.0 equiv.), *n*-pentanol (22 µL, 18 mg, 0.2 mmol, 10 equiv.), and fluorobenzene (93 µL, 96 mg, 1.0 mmol, 50 equiv.). Deuterated nitromethane (0.5 mL, *c* = 40 mM) was added, the tube inverted several times, and the initial ^{1}H NMR spectrum was measured. Subsequently, the tube was placed in a preheated (100 °C) NMR sample head. ^{1}H NMR spectra were recorded every 5 min at 100 °C for several hours.

Note: Only pentylphenyl ether complex **I** was observed during the reaction, no detection of the fluorobenzene complex **3g** by ^{1}H or ^{19}F NMR spectroscopy.

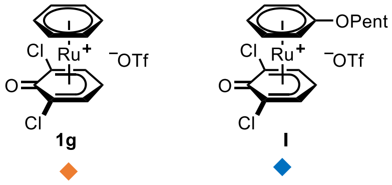

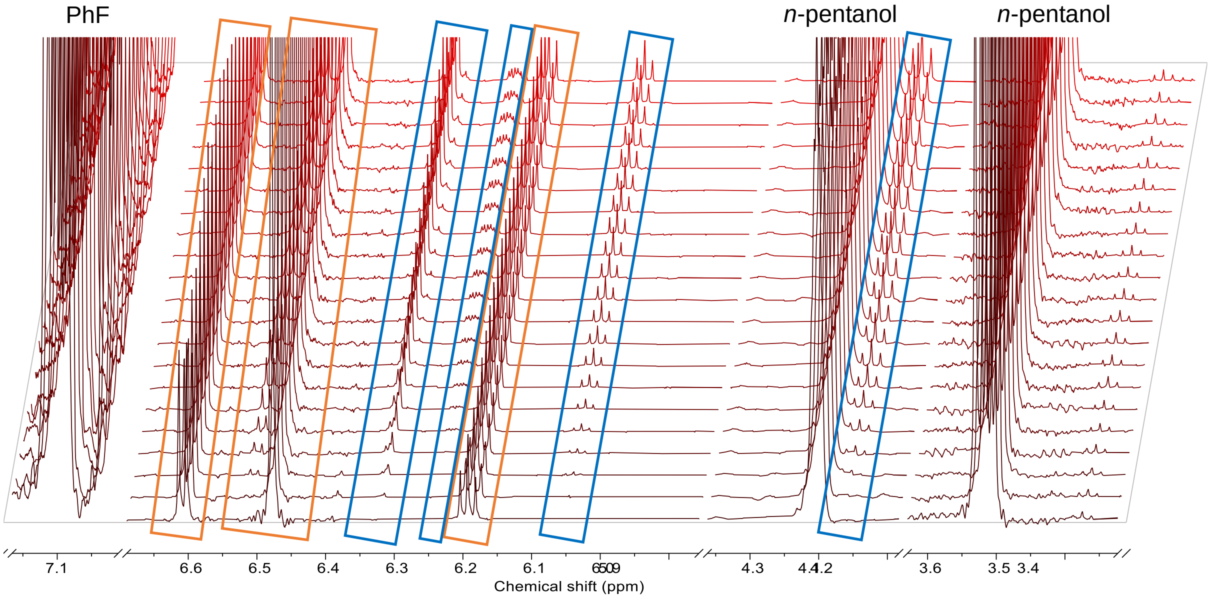

Fig S72: Series of ^{1}H-NMR spectra at 500 MHz, 373 K, MeNO2-d_3; **only formation of [η^6-pentylphenyl ether-η^5-(2,6-dichloro-1-phenoxo)Ru](OTf) I** observed. Orange box: signals corresponding to **1g**; blue boxes: signals corresponding to **I**.

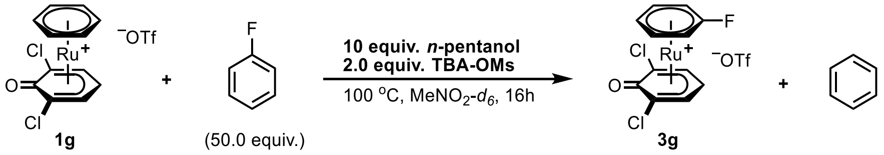

A 5 mm J-Young NMR tube was charged with **1g** (10 mg, 20 µmol, 1.0 equiv.), TBA-OMs (14 mg, 40 µmol, 2.0 equiv.),, *n*-pentanol (22 µL, 18 mg, 0.2 mmol, 10 equiv.), and fluorobenzene (93 µL, 96 mg, 1.0 mmol, 50 equiv.). Deuterated nitromethane (0.5 mL, *c* = 40 mM) was added, the tube inverted

several times, and the initial ^{1}H NMR spectrum was measured. Subsequently, the tube was placed in a preheated (100 °C) NMR sample head. ^{1}H NMR spectra were recorded every 5 min at 100 °C for several hours.

Note: Due to overlapping signals, no clear identification of fluorobenzene complex **3g** possible by ^{1}H NMR spectroscopy. However, by ^{19}F NMR spectroscopy, **3g** was detected.

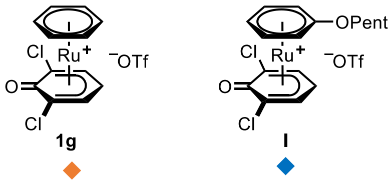

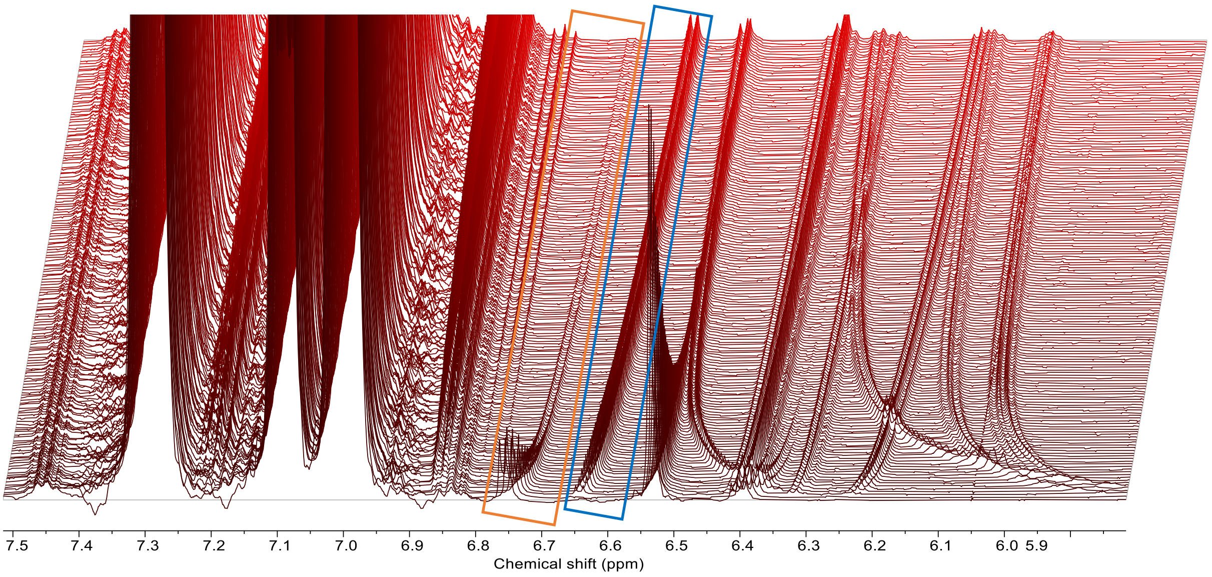

Fig S73: Series of ^{1}H-NMR spectra at 500 MHz, 373 K, $MeNO_2$-d_3; orange box: signals corresponding to **1g**; blue boxes: signals corresponding to **I**.

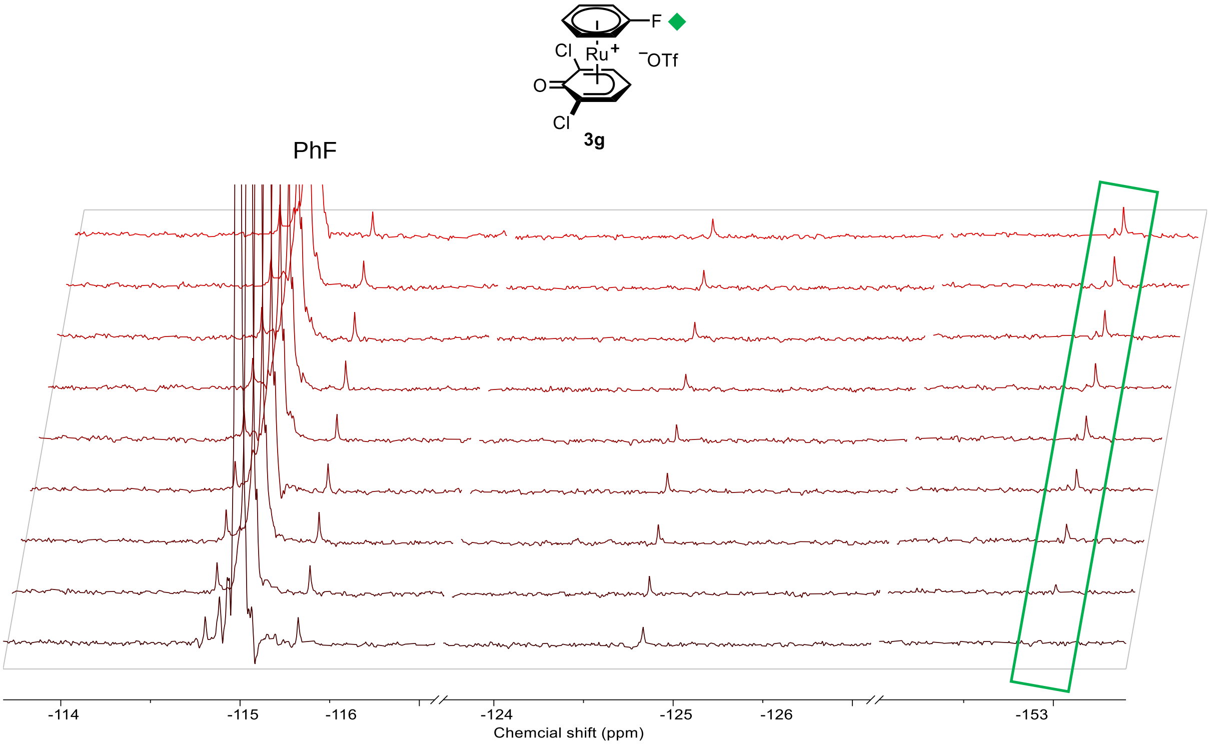

Fig S74: Series of ^{19}F-NMR spectra at 565 MHz, 373 K, $MeNO_2$-d_3; green box: signals corresponding to **3g**.

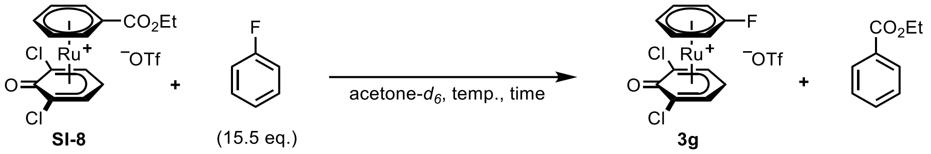

A 5 mm J-Young NMR tube was charged with **SI-8** (10 mg, 18 µmol, 1.0 equiv.) and dry fluorobenzene (30 µL, 30 mg, 0.28 mmol, 16 equiv.). Dry deuterated acetone (0.5 mL, *c* = 40 mM) was added, the tube inverted several times, and the initial ^{1}H NMR spectrum was measured. After the initial measurement, the tube was placed in an oil bath heated at the temperature specified in Fig S75. Then, a ^{1}H NMR spectrum was measured and the heating process continued.

Note: The formation of the fluorobenzene complex **3g** was confirmed by HRMS:

HRMS ESI (m/z) calculated for $C_{12}H_8Cl_2F_1O_1Ru_1$ [M]$^+$, 358.8974; found, 358.8970, deviation: 1.2 ppm.

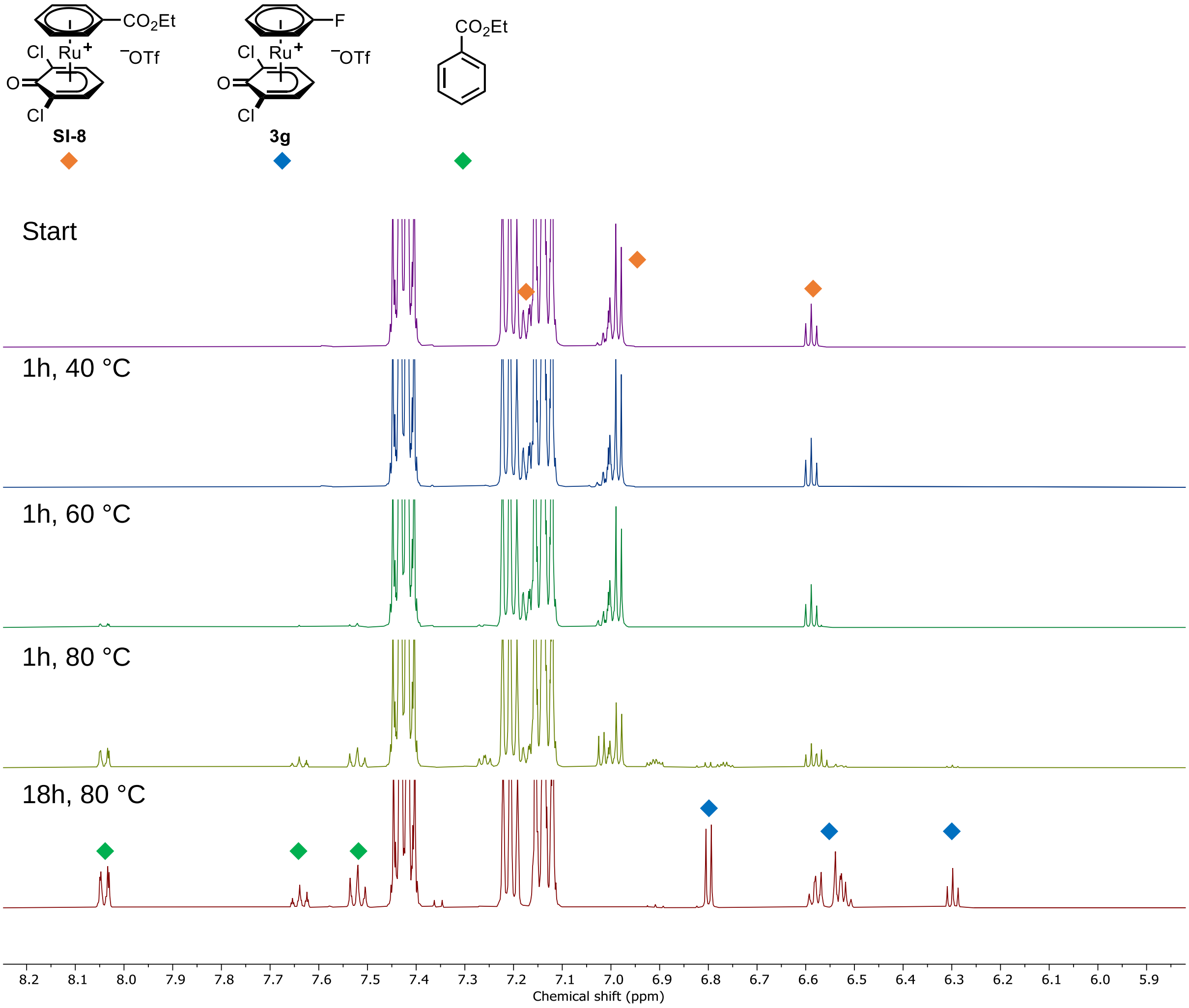

Fig S75: Series of ^{1}H-NMR spectra at 500 MHz, 23 °C, acetone-d_6 of the arene exchange experiment of **SI-8** with fluorobenzene; orange diamond: signals of **SI-8**, blue diamond: signals of **3g**, green diamond: signals of ethylbenzoate (not coordinated).

Stoichiometric control experiment

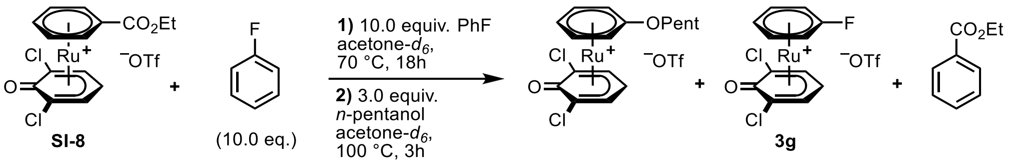

A 5 mm J-Young NMR tube was charged with **SI-8** (11 mg, 20 µmol, 1.0 equiv.) and dry fluorobenzene (19 µL, 19 mg, 0.20 mmol, 10 equiv.). Dry deuterated acetone (0.5 mL, c = 40 mM) was added, the tube filled with Ar, closed, inverted several times, and the initial ^{1}H NMR spectrum was measured. After the initial measurement, the tube was placed in an oil bath heated at 70 °C for 18h. After cooling to 25 °C, a ^{1}H NMR spectrum was measured. Subsequently, n-pentanol (7.0 µL, 5.0 mg, 60 µmol, 3.0 equiv.) was added, the tube was refilled with Ar and heated at 100 °C for 3h. After cooling to 25 °C, another ^{1}H NMR spectrum was measured.

Note: The triplet at 6.91 ppm (Fig S76) presumably corresponds to coordinated phenol as a result of hydrolysis of the coordinated fluorobenzene. The formation of the fluorobenzene complex **3g** and the alkyl-aryl ether complex were also confirmed by HRMS:

HRMS ESI (m/z) calculated for $C_{12}H_8Cl_2F_1O_1Ru_1$ [M]$^+$, 358.8974; found, 358.8979, deviation: 1.3 ppm.

HRMS ESI (m/z) calculated for $C_{17}H_{19}Cl_2O_2Ru_1$ [M]$^+$, 426.9800; found, 426.9799, deviation: 0.4 ppm.

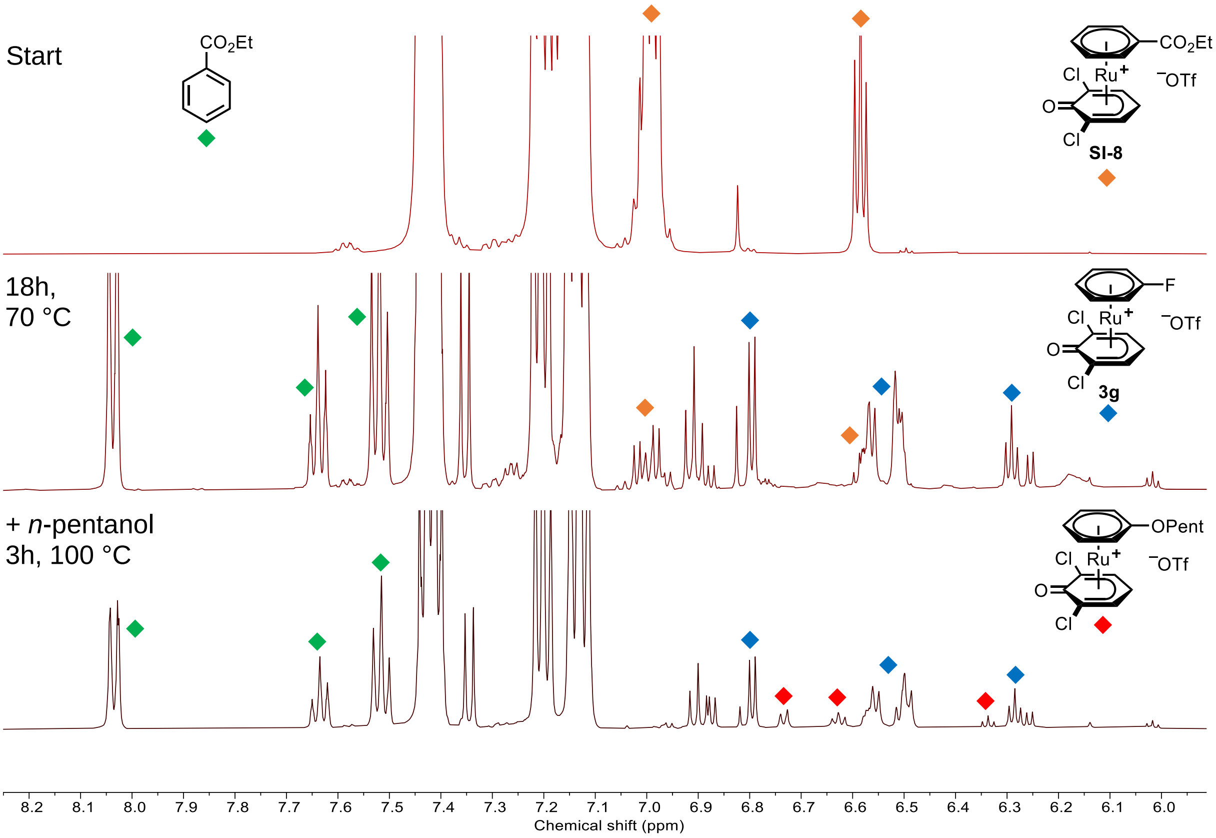

Fig S76: Series of ^{1}H-NMR spectra at 500 MHz, 23 °C, acetone-d_6 of the stoichiometric experiment of **SI-8** with fluorobenzene, followed by the reaction with n-pentanol; orange diamond: signals of **SI-8**, blue diamond: signals of **3g**, green diamond: signals of ethylbenzoate (not coordinated); red

diamonds: signals of the alkyl-arylether complex.

Protodecarboxylation of Phenylacetic Acids Catalyzed by 1a

Decarboxylation of Ibuprofen (6a) (1.0 mmol scale)

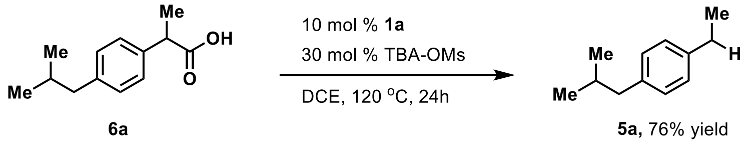

A 20 mL borosilicate vial equipped with a Teflon-coated magnetic stirring bar was charged with [η^6-benzene-η^5-(2,6-dibromo-4-methoxy-1-phenoxo)Ru](OTf) **1a** (62 mg, 0.10 mmol, 10 mol%), Ibuprofen **6a** (0.21 g, 1.0 mmol, 1.0 equiv.), and tetrabutylammonium methylsulfonate (TBA-OMs) (0.10 g, 0.30 mmol, 30 mol%). The vial was connected via a vial adapter to a Schlenk line and was evacuated and purged with argon three times. DCE (2.0 mL, c = 0.25 M) was then added, the adapter closed, and the suspension stirred at 120 °C for 24 h under an argon atmosphere. Subsequently, the mixture was filtered through a thin layer of Celite loaded on a pipette followed by washing with DCM (3 x 1 mL). The volatiles was evaporated under reduced pressure and the residue was purified by column chromatography on silica gel eluting with a solvent mixture of hexanes to afford **5a** (0.12 g, 0.76 mmol, 76%) as a colorless oil.

R_f = 0.8 (hexanes).

NMR Spectroscopy:

^{1}H NMR (500 MHz, $CDCl_3$, 23 °C, δ): 7.11 (d, *J* = 7.1 Hz, 2H), 7.06 (d, *J* = 7.1 Hz, 2H), 2.63 (q, *J* = 7.6 Hz, 2H), 2.44 (d, *J* = 7.2 Hz, 2H), 1.85 (hept, 1H), 1.23 (t, *J* = 7.6 Hz, 3H), 0.90 (d, *J* = 6.6 Hz, 6H).

^{13}C NMR (126 MHz, $CDCl_3$, 23 °C, δ): 141.6, 139.0, 129.2, 45.20, 30.41, 28.58, 22.55, 15.78.

HRMS EI (m/z) calculated for $C_{12}H_{18}$ $[M]^+$, 162.1403; found, 162.1406 deviation: −1.7 ppm.

Decarboxylation of Ibuprofen (6a) (1.0 mmol scale) with 1-Cp* as Catalyst

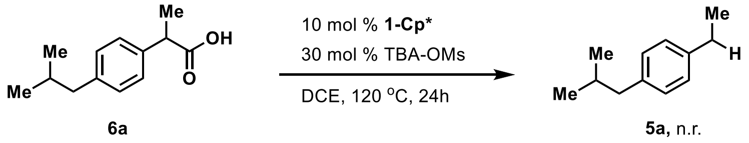

With **1-Cp*** as catalyst: A 20 mL borosilicate vial equipped with a Teflon-coated magnetic stirring bar was charged with [η^6-benzene-η^5-(pentamethylcyclopentadienyl)Ru](OTf) **1-Cp*** (7.8 mg, 20 µmol, 10 mol%), Ibuprofen **6a** (0.21 g, 1.0 mmol, 1.0 equiv.), and tetrabutylammonium methylsulfonate (TBA-OMs) (0.10 g, 0.30 mmol, 30 mol%). The vial was connected via a vial adapter to a Schlenk line and was evacuated and purged with argon three times. DCE (2 mL, c = 0.25 M) was then added, the adapter closed, and the suspension stirred at 120 °C for 24 h under an argon atmosphere. After completion, trimethoxybenzene (11 mg, 67 µmol, 33 mol%) was added as internal standard. <5 % ^{1}H-NMR yield of **5a** was observed via ^{1}H-NMR.

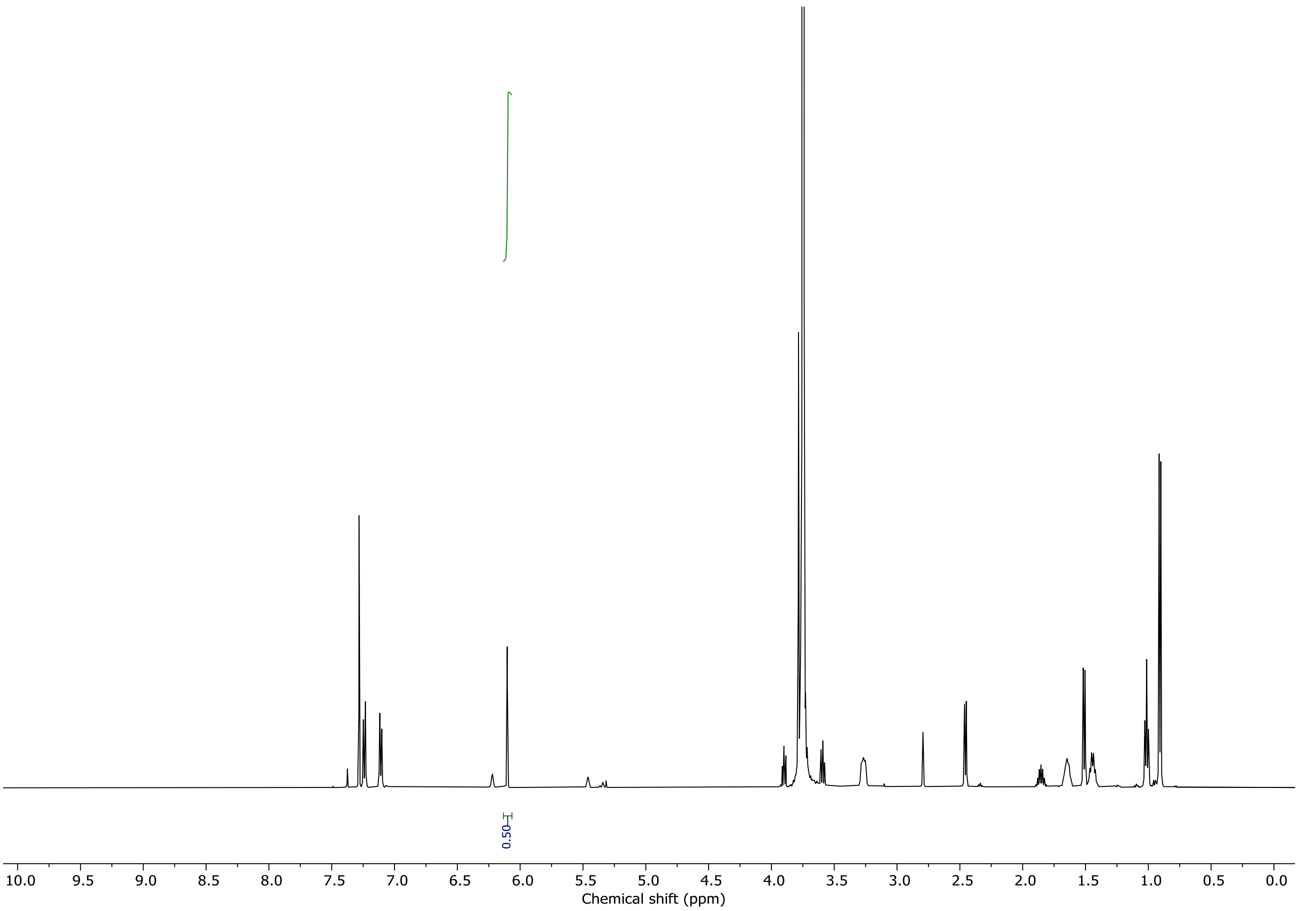

Fig S77: ^{1}H-NMR spectrum after the reaction; 500 MHz, 23 °C, $CDCl_3$.

Decarboxylation of Ibuprofen (6a) (1.0 mmol scale) with RhCp* as Catalyst

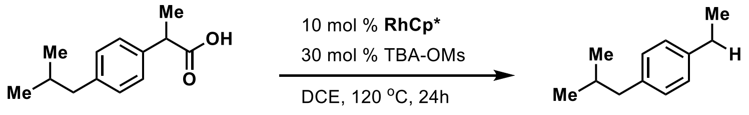

With **RhCp*** as catalyst: A 20 mL borosilicate vial equipped with a Teflon-coated magnetic stirring bar was charged with [η^6-anisole-η^5-(pentamethylcyclopentadienyl)Rh]$(OTf)_2$ **RhCp*** (12 mg, 20 µmol, 10 mol%), Ibuprofen **6a** (0.21 g, 1.0 mmol, 1.0 equiv.), and tetrabutylammonium methylsulfonate (TBA-OMs) (0.10 g, 0.30 mmol, 30 mol%). The vial was connected via a vial adapter to a Schlenk line and was evacuated and purged with argon three times. DCE (2 mL, c = 0.25 M) was then added, the adapter closed, and the suspension stirred at 120 °C for 24 h under an argon atmosphere. After completion, trimethoxybenzene (11 mg, 67 µmol, 33 mol%) was added as internal standard. 29 % NMR yield of **5a** was observed via ^{1}H-NMR spectroscopy by comparing the integrals of the standard signal at 6.11 ppm to the integrals of the signal of **5a** at 2.64 ppm.

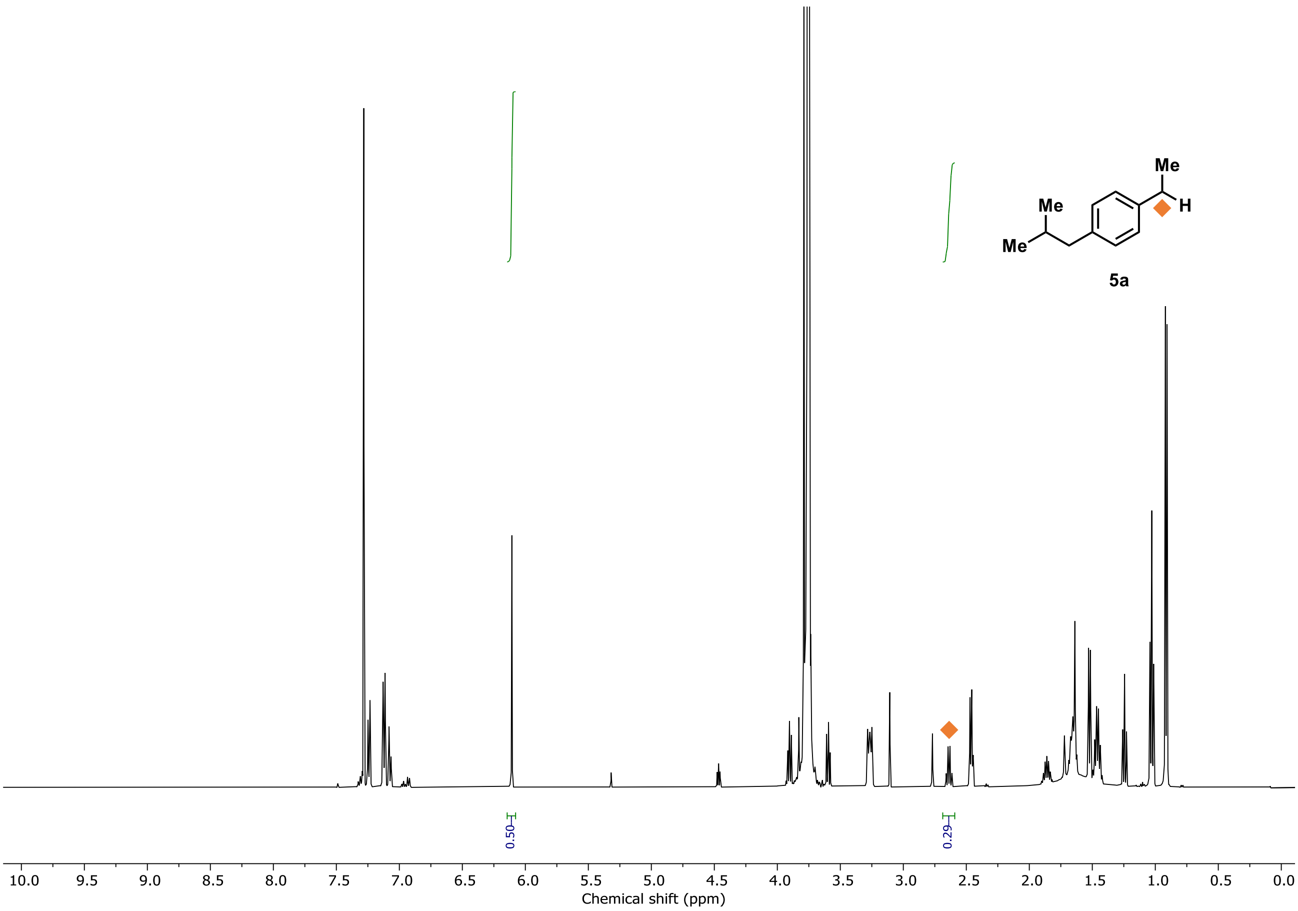

Fig S78: ^{1}H-NMR spectrum after the reaction; 500 MHz, 23 °C, $CDCl_3$; orange diamond: signal of **5a**.

Decarboxylation of 4-phenylphenylacetic acid (6b)

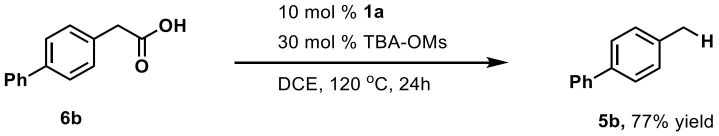

A 20 mL borosilicate vial equipped with a Teflon-coated magnetic stirring bar was charged with [*η*^{6}-benzene-*η*^{5}-(2,6-dibromo-4-methoxy-1-phenoxo)Ru](OTf) **1a** (31 mg, 50 µmol, 10 mol%), 4-phenylphenylacetic acid **6b** (0.11 g, 0.50 mmol, 1.0 equiv.), and tetrabutylammonium methylsulfonate (TBA-OMs) (51 mg, 0.15 mmol, 30 mol%). The vial was connected via a vial adapter to a Schlenk line and was evacuated and purged with argon three times. DCE (2.0 mL, c = 0.25 M) was then added and the suspension stirred at 120 °C for 24 h under an argon atmosphere. Subsequently, the mixture was filtered through a thin celite layer loaded on a pipette followed by washing with DCM (3 x 1 mL). Subsequently, volatiles were removed under reduced pressure and the residue was purified by column chromatography on silica gel eluting with hexanes to afford **5b** (65 mg, 0.39 mmol, 77%) as a colorless oil.

R*f* = 0.7 (hexanes).

NMR Spectroscopy:

^{1}H NMR (500 MHz, $CDCl_3$, 23 °C, δ): 7.63 – 7.61 (m, 2H), 7.56 – 7.51 (m, 2H), 7.56 – 7.51 (m,

2H), 7.39 – 7.33 (m, 1H), 7.32 – 7.26 (m, 2H), 2.44 (s, 3H).

^{13}C NMR (126 MHz, $CDCl_3$, 23 °C, δ): 141.3, 138.5, 137.2, 129.6, 128.9, 127.1 (m), 21.23.

HRMS EI (m/z) calculated for $C_{13}H_{12}$ $[M]^+$, 168.0936; found, 168.0934 deviation: −1.6 ppm.

Decarboxylation phenylmalonic acid (6c)

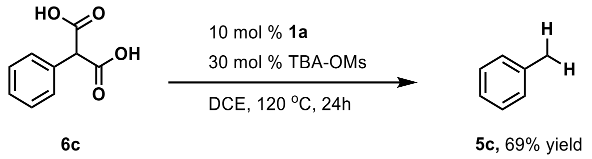

A 20 mL borosilicate vial equipped with a Teflon-coated magnetic stirring bar was charged with [η^6-benzene-η^5-(2,6-dibromo-4-methoxy-1-phenoxo)Ru](OTf) **1a** (31 mg, 50 µmol, 10 mol%), phenylmalonic acid **6c** (0.18 g, 0.50 mmol, 1.0 equiv.), and tetrabutylammonium methylsulfonate (TBA-OMs) (51 mg, 0.15 mmol, 30 mol%). The vial was connected via a vial adapter to a Schlenk line and was evacuated and purged with argon three times. DCE (2.0 mL, c = 0.25 M) was then added, the adapter closed, and the suspension stirred at 120 °C for 24 h under an argon atmosphere. Subsequently, Silica (ca. 1.0 g) was added to the reaction mixture and the volatiles were care evaporated carefully by rotovap and the residue was purified by column chromatography on silica gel eluting with pentane to afford **5c** (46 mg, 0.44 mmol, 69%) as a colorless oil.

R_f = 0.8 (hexanes).

NMR Spectroscopy:

^{1}H NMR (500 MHz, $CDCl_3$, 23 °C, δ): 7.32 – 7.24 (m, 2H), 7.23 – 7.16 (m, 3H), 2.39 (s, 3H).

^{13}C NMR (126 MHz, $CDCl_3$, 23 °C, δ): 138.0, 129.2, 128.4, 125.5, 21.60.

HRMS EI (m/z) calculated for C_7H_9 $[M]^+$, 92.0621; found, 92.0620 deviation: −0.2 ppm.

Decarboxylation of α, α-difluorophenylacetic acid (6d)

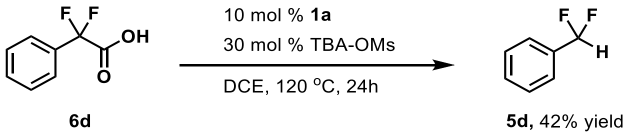

A 20 mL borosilicate vial equipped with a Teflon-coated magnetic stirring bar was charged with [η^6-benzene-η^5-(2,6-dibromo-4-methoxy-1-phenoxo)Ru](OTf) **1a** (31 mg, 50 µmol, 10 mol%), α,α-difluorophenylacetic acid **6d** (0.17 g, 0.50 mmol, 1.0 equiv.), and tetrabutylammonium methylsulfonate (TBA-OMs) (51 mg, 0.15 mmol, 30 mol%). The vial was connected via a vial adapter to a Schlenk line and was evacuated and purged with argon three times. DCE (2.0 mL, c = 0.25 M) was then added, the adapter closed and the suspension stirred at 120 °C for 24 h under an argon atmosphere. Afterwards, the reaction mixture directly purified by column chromatography on silica gel eluting with pentane. The fractions containing product were combined and pentane was removed by distillation at 40 °C to afford **5d** (27 mg, 0.20 mmol, 42%) as a colorless oil.

R*f* = 0.9 (hexanes).

NMR Spectroscopy:

^{1}H NMR (500 MHz, $CDCl_3$, 23 °C, δ): 7.56 – 7.44 (m, 5H), 6.66 (t, *J* = 56.5 Hz, 1H).

^{13}C NMR (126 MHz, $CDCl_3$, 23 °C, δ): 134.5 (t, *J* = 22.3 Hz), 130.9 (t, *J* = 2.1 Hz), 128.8, 125.67 (t, *J* = 6.2 Hz), 116.5, 114.9, 113.3.

^{19}F NMR (565 MHz, $CDCl_3$, 23 °C, δ): −110.59 (d, *J* = 56.8 Hz).

HRMS EI (m/z) calculated for $C_7H_6F_2$ $[M]^+$, 128.0432; found, 128.0432, deviation: −0.4 ppm.

Decarboxylation of 1,1-diphenylacetic acid (6e)

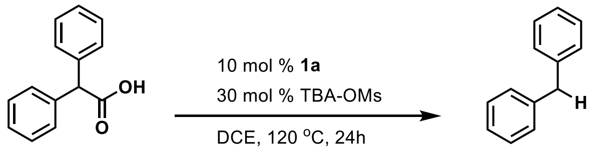

6e 5e, 94% yield

A 20 mL borosilicate vial equipped with a Teflon-coated magnetic stirring bar was charged with [*η*^{6}-benzene-*η*^{5}-(2,6-dibromo-4-methoxy-1-phenoxo)Ru](OTf) **1a** (31 mg, 50 µmol, 10 mol%), 1,1-diphenylacetic acid **6e** (0.11 g, 0.50 mmol, 1.0 equiv.), and tetrabutylammonium methylsulfonate (TBA-OMs) (51 mg, 0.15 mmol, 30 mol%). The vial was connected via a vial adapter to a Schlenk line and was evacuated and purged with argon three times. DCE (2.0 mL, c = 0.25 M) was then added, the adapter closed, and the suspension stirred at 120 °C for 24 h under an argon atmosphere. Afterwards, Silica (ca. 1.0 g) was added to the reaction mixture and the volatiles were removed under reduced pressure. Subsequently, the residue was purified by column chromatography on silica gel eluting with pentane to afford **5e** (80 mg, 0.47 mmol, 94%) as a colorless oil.

R*f* = 0.9 (*i*-hexanes, (v:v))

NMR Spectroscopy:

^{1}H NMR (500 MHz, $CDCl_3$, 23 °C, δ): 7.34 – 7.28 (m, 4H), 7.25 – 7.18 (m, 5H), 4.01 (s, 2H).

^{13}C NMR (126 MHz, $CDCl_3$, 23 °C, δ): 141.3, 129.1, 128.6, 126.2, 42.1.

HRMS EI (m/z) calculated for $C_{13}H_{12}$ $[M]^+$, 168.0934; found, 168.0932, deviation: −1.2 ppm.

Decarboxylation of 1,3-phenyldiacetic acid (6f)

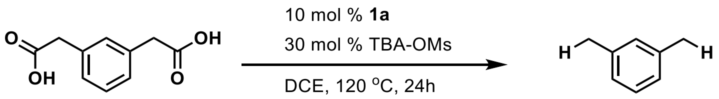

6f 5f, 94% yield

A 20 mL borosilicate vial equipped with a Teflon-coated magnetic stirring bar was charged with [*η*^{6}-benzene-*η*^{5}-(2,6-dibromo-4-methoxy-1-phenoxo)Ru](OTf) **1a** (31 mg, 50 µmol, 10 mol%), 1,3-phenyldiacetic acid **6f** (0.19 g, 0.50 mmol, 1.0 equiv.), and tetrabutylammonium methylsulfonate (TBA-OMs) (51 mg, 0.15 mmol, 30 mol%). The vial was connected via a vial adapter to a Schlenk line

and was evacuated and purged with argon three times. DCE (2.0 mL, c = 0.25 M) was then added, the adapter closed, and the suspension stirred at 120 °C for 24 h under an argon atmosphere. Afterwards, Silica (ca. 1.0 g) was added to the reaction mixture and the volatiles were removed carefully under reduced pressure. Subsequently, the residue was purified by column chromatography on silica gel eluting with pentane to afford **5f** (53 mg, 0.47 mmol, 94%) as a colorless oil.

R_f = 0.9 (hexanes)

NMR Spectroscopy:

^{1}H NMR (500 MHz, $CDCl_3$, 23 °C, δ): 7.17 (t, *J* = 7.5 Hz, 1H), 7.04 – 6.97 (m, 3H), 2.34 (s, 6H).

^{13}C NMR (126 MHz, $CDCl_3$, 23 °C, δ): 141.3, 129.1, 128.6, 126.2, 42.10.

HRMS EI (m/z) calculated for $C_7H_6F_2$ $[M]^+$, 106.0778; found, 106.0777 deviation: −0.5 ppm.

Mechanistic investigation and optimization

Proposed catalytic cycle

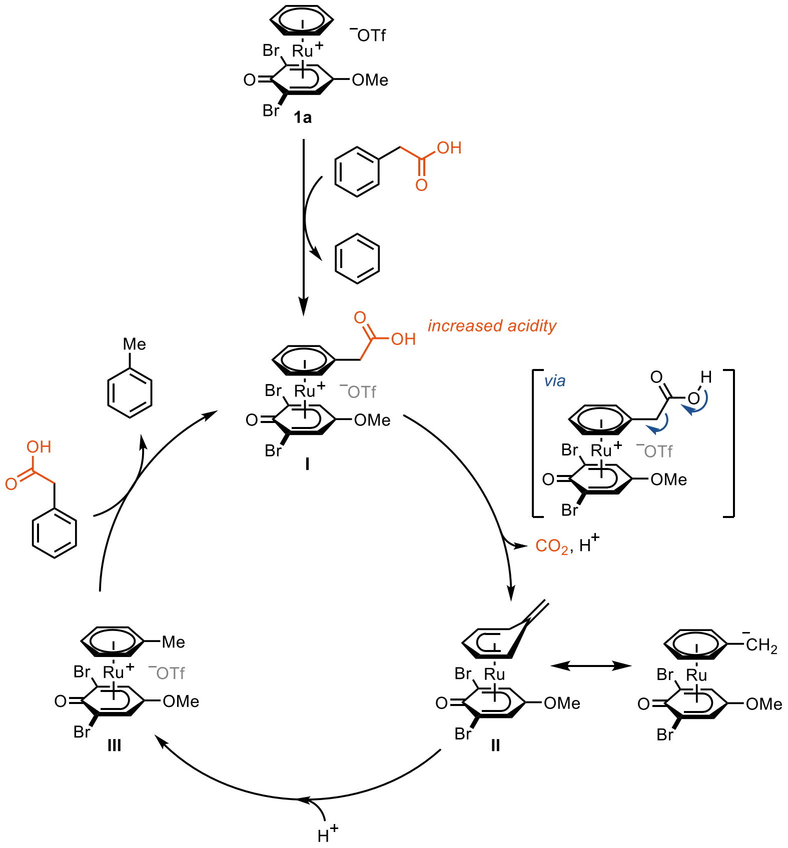

Fig S79: Proposed catalytic cycle for the protodecarboxylation of phenylacetic acids with **1a**.

Optimization

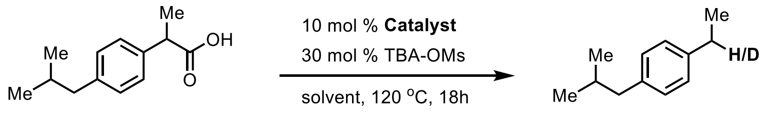

6a

A 4 mL borosilicate vial equipped with a Teflon-coated magnetic stirring bar was charged with **catalyst** (10 µmol, 10 mol%), Ibuprofen **6a** (21 mg, 0.10 mmol, 1.0 equiv.), and tetrabutylammonium methylsulfonate (10 mg, 30 µmol, 30 mol%). The vial was connected via a vial adapter to a Schlenk line and was evacuated and purged with argon three times. Solvent (0.4 mL, c = 0.25 M) was then added, the adapter closed, and the suspension stirred at 120 °C for 18h under an argon atmosphere. Subsequently, the mixture was cooled to 25 °C, the solvent was evaporated and $CDCl_3$ (0.5 mL) and CH_2Br_2 as internal standard were added. NMR yield was determined via ^{1}H-NMR spectroscopy by comparing the integrals of the standard signal at 4.96 ppm to the integrals of the signal at 7.06 ppm.

Table S5 Results for the proto/deuterodecarboxylation of **6a** with different catalysts, NMR yield was determined via ^{1}H-NMR spectroscopy by comparing the integrals of the standard signal at 4.96 ppm to the integrals of the signal at 7.06 ppm:

Catalyst	Arene exchange rate (Table S1)	Solvent	H/D	Result
**1i**	22.23 mol%·h^{-1}	DCE	H	5% yield
**1i**	22.23 mol%·h^{-1}	Acetone-d_6	D	15% yield
**1g**	56.53 mol%·h^{-1}	DCE	H	62% Yield
**1g**	56.53 mol%·h^{-1}	Acetone-d_6	D	51% yield
**1a**	68.75 mol%·h^{-1}	DCE	H	**69% yield**
**1a**	68.75 mol%·h^{-1}	Acetone-d_6	D	68% yield

Control experiments

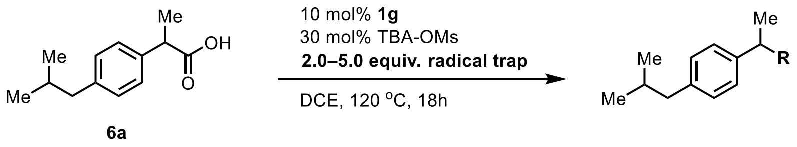

A 4 mL borosilicate vial equipped with a Teflon-coated magnetic stirring bar was charged with **1g** (5.0 mg, 10 µmol, 10 mol%), Ibuprofen **6a** (21 mg, 0.10 mmol, 1.0 equiv.), radical trap (0.2–0.5 mmol, 2.0–5.0 equiv.), and tetrabutylammonium methylsulfonate (10 mg, 30 µmol, 30 mol%). The vial was connected via a vial adapter to a Schlenk line and was evacuated and purged with argon three times. DCE (0.4 mL, c = 0.25 M) was then added, the adapter closed, and the suspension stirred at 120 °C for 18h under an argon atmosphere. Subsequently, the mixture was cooled to 25 °C, the solvent was evaporated and $CDCl_3$ (0.5 mL) and CH_2Br_2 as internal standard were added. Desired products were not detected.

Table S6 Results for the control experiments using different radical traps:

Radical trap	Equivalents	R	Result
CCl_4	5.0	Cl	<5% yield
CBr_4	5.0	Br	<5% yield
Selectfluor	3.0	F	<5% yield
1,2-dibromoethane	5.0	Br	<5% yield
I_2	3.0	I	<5% yield
B_2Pin_2	2.0	BPin	<5% yield

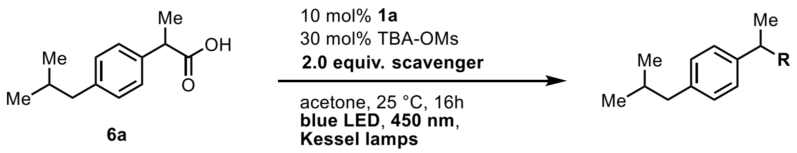

A 4 mL borosilicate vial equipped with a Teflon-coated magnetic stirring bar was charged with **1a** (12 mg, 20 µmol, 10 mol%), Ibuprofen **6a** (42 mg, 0.20 mmol, 1.0 equiv.), scavenger (0.2 mmol, 1.0 equiv.), and tetrabutylammonium methylsulfonate (20 mg, 60 µmol, 30 mol%). The vial was connected via a vial adapter to a Schlenk line and was evacuated and purged with argon three times. acetone (0.8 mL, c = 0.25 M) was then added, the adapter closed, and the suspension stirred at for 18h under blue LED irradiation under an argon atmosphere. Subsequently, the solvent was evaporated and $CDCl_3$ (0.5 mL) and CH_2Br_2 as internal standard were added. Desired products were not detected.

Table S7 Results for the control experiments using different scavengers **under irradiation with blue LEDs**:

Scavenger	Equivalents	R	Result
Acetone-d_6	1.0	H/D	<5% yield
AIBN	1.0	CMe_2CN	<5% yield
B_2Pin_2	1.0	BPin	<5% yield

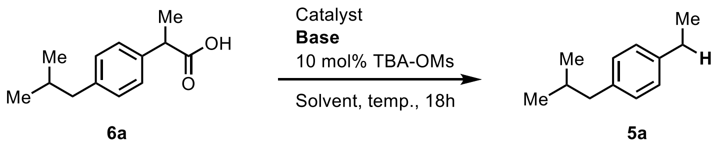

A 4 mL borosilicate vial equipped with a Teflon-coated magnetic stirring bar was charged with **catalyst** (0–5.0 µmol, 0–5 mol%), Ibuprofen **6a** (0.10 g, 0.5 mmol, 1.0 equiv.), base (0.5–1.0 mmol, 1.0–2.0 equiv.), and tetrabutylammonium methylsulfonate (17 mg, 50 µmol, 10 mol%). The vial was connected via a vial adapter to a Schlenk line and was evacuated and purged with argon three times. solvent (2.0 mL, c = 0.25 M) was then added, the adapter closed, and the suspension stirred at 120 °C for 18h under an argon atmosphere. Subsequently, the mixture was cooled to 25 °C, the solvent was evaporated and $CDCl_3$ (0.5 mL) and CH_2Br_2 (24 µL, 87 mg, 0.5 mmol, 1.0 equiv.) as internal standard were added. Desired products were not detected.

Table S8 Results for the control experiments using different bases:

Base	Catalyst	Conditions	Result
Cs_2CO_3 (1.0 equiv.)	**No Catalyst**	DCE, 120 °C	<5% yield
Cs_2CO_3 (1.0 equiv.)	**1a** (1 mol%)	DCE, 120 °C	<5% yield
Cs_2CO_3 (1.0 equiv.)	**1a** (5 mol%)	DCE, 100 °C	<5% yield
Cs_2CO_3 (1.0 equiv.)	**1a** (5 mol%)	1,4-dioxane, 100 °C	<5% yield
Cs_2CO_3 (1.0 equiv.)	**1a** (5 mol%)	1,4-dioxane, 120 °C	<5% yield
CsF (2.0 equiv.)	**1a** (5 mol%)	DCE, 120 °C	<5% yield

DIPEA (2.0 equiv.)	**1a** (5 mol%)	1,4-dioxane, 120 °C	<5% yield
2,6-*t*Bu-pyridine (2.0 equiv)	**1a** (5 mol%)	1,4-dioxane, 120 °C	<5% yield

Deuteration experiments

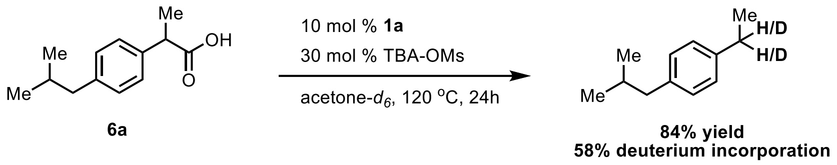

A 20 mL borosilicate vial equipped with a Teflon-coated magnetic stirring bar was charged with **1a** (58 mg, 0.10 mmol, 10 mol%), Ibuprofen **6a** (0.21 g, 1.0 mmol, 1.0 equiv.), and tetrabutylammonium methylsulfonate (0.10 g, 0.30 mmol, 30 mol%). The vial was connected via a vial adapter to a Schlenk line and was evacuated and purged with argon three times. Acetone-d_6 (4.0 mL, c = 0.25 M) was then added, the adapter closed, and the suspension stirred at 120 °C for 18h under an argon atmosphere. Subsequently, the mixture was cooled to 25 °C and the solvent was evaporated. The mixture was filtered through a thin layer of Celite loaded on a pipette followed by washing with DCM (3 x 1 mL). The volatiles were evaporated under reduced pressure and the residue was purified by column chromatography on silica gel eluting with hexanes to afford the desired product (0.14 g, 0.84 mmol, 84%) as a colorless oil.

Note: The signal at 2.63 ppm (Fig S80) refers to the benzylic position on which deuteration is observed. The integral of 0.85 refers to the average deuterium incorporation. As the benzylic position bears two hydrogens, a maximum integral of 2.0 would be possible. An integral of 0.85 thus shows a ratio of 0.85:1.15 proton:deuterium (or 0.425:0.575), which translates to 58% deuterium incorporation. A deuterium incorporation of >50% shows that partially dideuteration is obtained, caused by benzylic C–H-deuteration. Therefore the result demonstrates that partially benzylic C–H-deuteration is obtained.

The substrate **6a** bears two benzylic position. No deuteration is observed on the second benzylic position which we propose is caused by the higher sterical hindrance due to the adjacent isopropyl group.

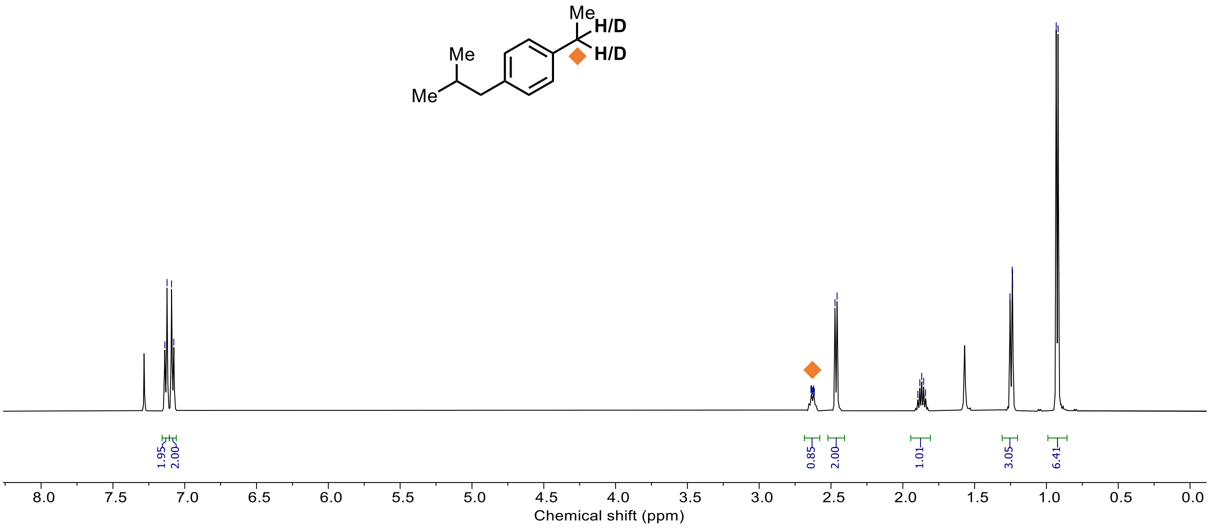

Fig S80: ^{1}H-NMR of isolated product; 500 MHz, 23 °C, $CDCl_3$; orange diamond: signal of benzylic position on which deuteration is observe, integral: 0.85.

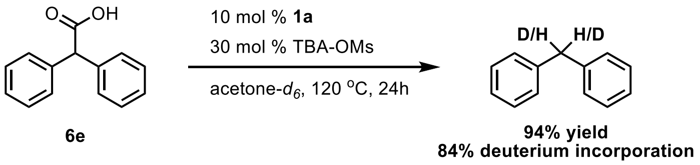

A 20 mL borosilicate vial equipped with a Teflon-coated magnetic stirring bar was charged with **1a** (29 mg, 0.05 mmol, 10 mol%), 1,1-diphenylacetic acid **6e** (0.11 g, 0.5 mmol, 1.0 equiv.), and tetrabutylammonium methylsulfonate (50 mg, 0.15 mmol, 30 mol%). The vial was connected via a vial adapter to a Schlenk line and was evacuated and purged with argon three times. Acetone-d_6 (4.0 mL, c = 0.25 M) was then added, the adapter closed, and the suspension stirred at 120 °C for 18h under an argon atmosphere. Subsequently, the mixture was cooled to 25 °C and the solvent was evaporated. The mixture was filtered through a thin layer of Celite loaded on a pipette followed by washing with DCM (3 x 1 mL). The volatiles were evaporated under reduced pressure and the residue was purified by column chromatography on silica gel eluting with hexanes to afford the desired product (80 mg, 0.47 mmol, 94%) as a colorless oil.

Note: The signal at 4.00 ppm (Fig S81) refers to the benzylic position on which deuteration is observed. The integral of 0.32 refers to the average deuterium incorporation. As the benzylic position bears two hydrogens, a maximum integral of 2.0 would be possible. An integral of 0.32 thus shows a ratio of 0.32:1.68 proton:deuterium (or 0.16:0.84), which translates to 84% deuterium incorporation. A deuterium incorporation of >50% shows that partially dideuteration is obtained, caused by benzylic C–H-deuteration. Therefore the result demonstrates that partially benzylic C–H-deuteration is obtained.

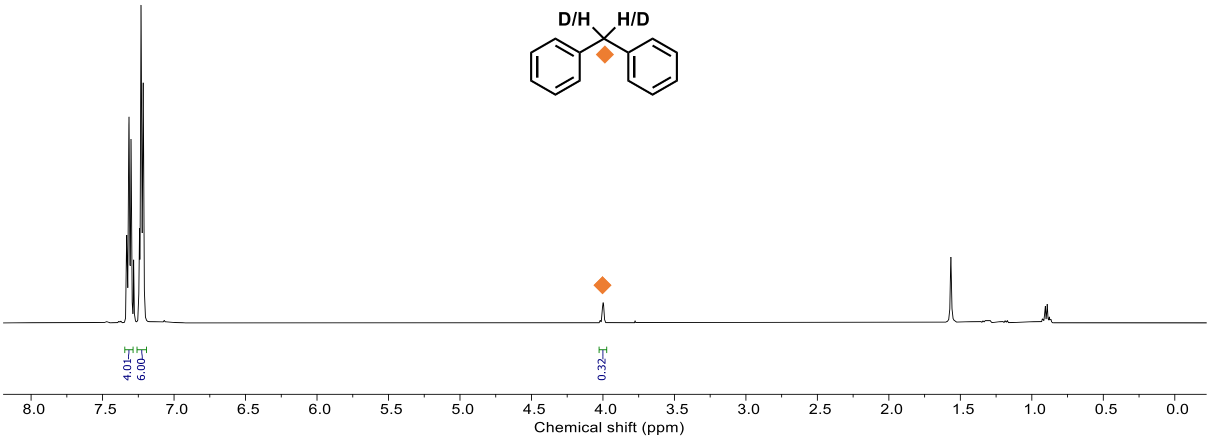

Fig S81: ^{1}H-NMR of isolated product; 500 MHz, 23 °C, $CDCl_3$; orange diamond: signal of benzylic position on which deuteration is observe, integral: 0.32.

X-RAY CRYSTALLOGRAPHIC ANALYSIS

Single crystals suitable for X-ray crystallographic analysis were obtained by vial in vial gas phase diffusion of diethyl ether into a concentrated MeOH solution. The atoms are depicted with 50% probability ellipsoids. The crystallographic data is summarized in the following tables. The crystallographic data for complex **1-Cp*** was extracted form a report by Gasparetto et al.[7]

[η^6-Benzene-η^5-(2,6-dibromo-4-methoxy-1-phenoxo)Ru](OTf) (1a)

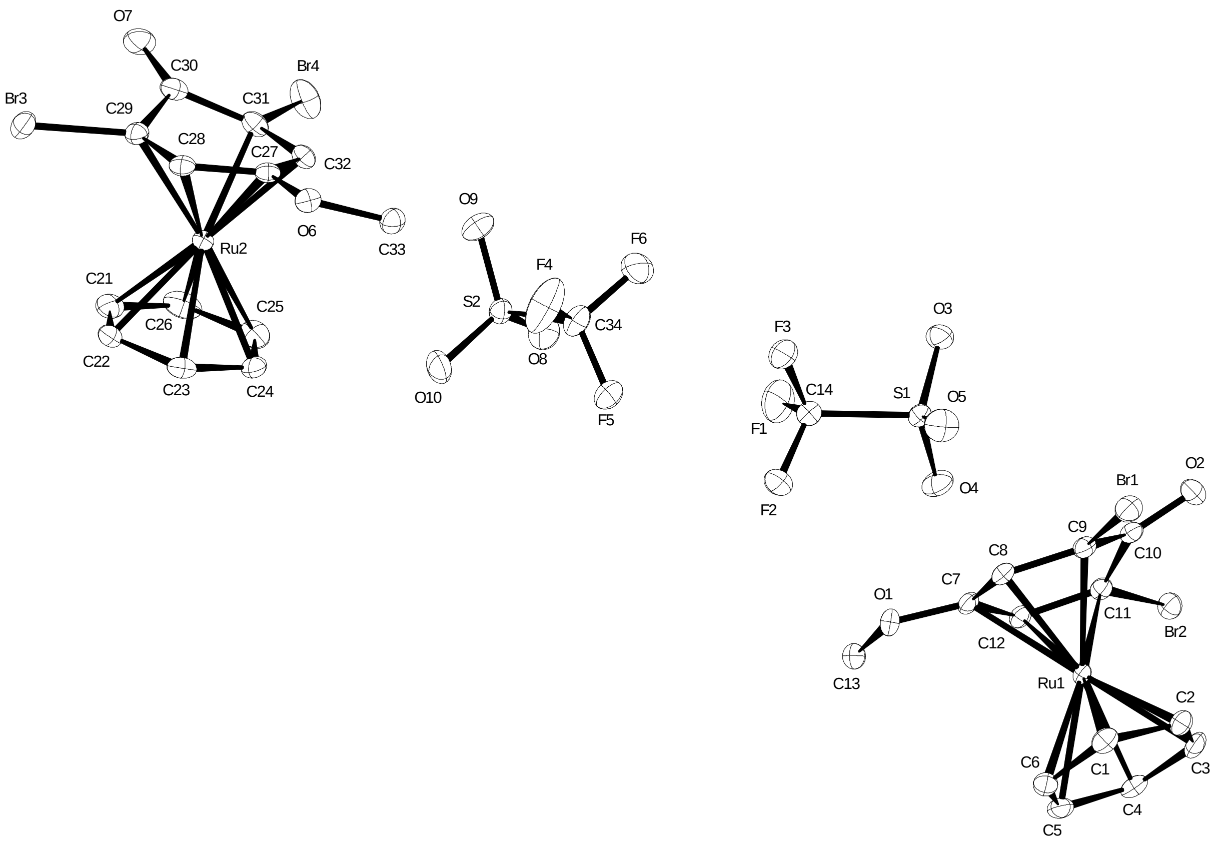

Fig S82: X-ray structure of compound **1a**.

Table S9 Crystal data and structure refinement of compound **1a**:

Identification code	14868	
Empirical formula	$C_{28}H_{22}Br_4F_6O_{10}Ru_2S_2$	
Color	yellow	
Formula weight	1218.35 g · mol^{-1}	
Temperature	100(2) K	
Wavelength	0.71073 Å	
Crystal system	ORTHORHOMBIC	
Space group	**Pbca, (no. 61)**	
Unit cell dimensions	a = 13.581(2) Å	α = 90°.

	b = 13.699(2) Å	β = 90°.
	c = 38.053(6) Å	γ = 90°.
Volume	7080(2) $Å^3$	
Z	8	
Density (calculated)	2.286 Mg · m^{-3}	
Absorption coefficient	5.572 mm^{-1}	
F(000)	4672 e	
Crystal size	0.204 x 0.17 x 0.06 mm^3	
θ range for data collection	2.141 to 30.508°.	
Index ranges	$-19 \leq h \leq 19$, $-19 \leq k \leq 19$, $-54 \leq l \leq 54$	
Reflections collected	504442	
Independent reflections	10776 [R_{int} = 0.0551]	
Reflections with I>2σ(I)	10080	
Completeness to θ = 25.242°	99.4 %	
Absorption correction	Semi-empirical from equivalents	
Max. and min. transmission	0.83 and 0.46	
Refinement method	Full-matrix least-squares on F^2	
Data / restraints / parameters	10776 / 0 / 471	
Goodness-of-fit on F^2	1.086	
Final R indices [I>2σ(I)]	R_1 = 0.0221	wR^2 = 0.0429
R indices (all data)	R_1 = 0.0251	wR^2 = 0.0437
Largest diff. peak and hole	1.0 and −1.0 e · $Å^{-3}$.	

Selected Bond lengths [Å]

Distances between phenoxo ligand and Ru-central atom are shown. Distance between carbonyl carbon (C10) and Ru-central atom in bold to highlight the elongated bond resulting from the η^5-coordination of the phenoxo ligand. For atom numbers see Fig S82.

Ru(1)–C(7): 2.2914
Ru(1)–C(8): 2.1952
Ru(1)–C(9): 2.1818
Ru(1)–C(10): 2.5490
Ru(1)–C(11): 2.1882
Ru(1)–C(12): 2.1986

[η^6-Benzene-η^5-(4-methyl-1-phenoxo)Ru](OTf) (1h)

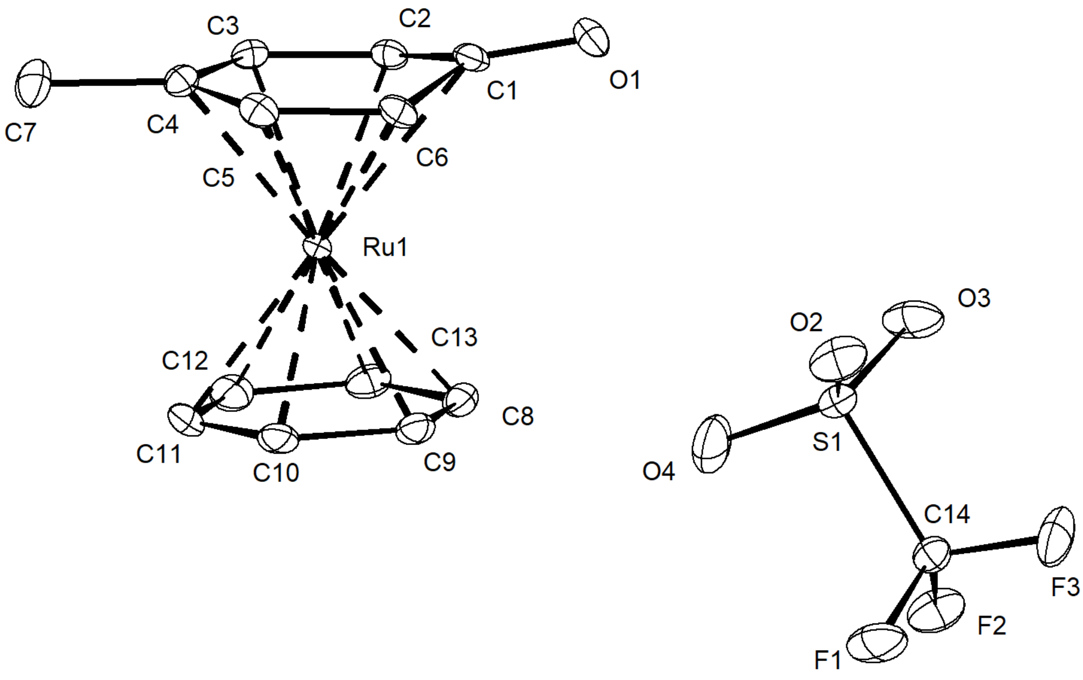

Fig S83: X-ray structure of compound **1h**.

Table S10 Crystal data and structure refinement of compound **1h**:

Identification code	14698	
Empirical formula	$C_{14}H_{13}F_3O_4RuS$	
Color	colourless	
Formula weight	435.37 g·mol^{-1}	
Temperature	100(2) K	
Wavelength	0.71073 Å	
Crystal system	Monoclinic	
Space group	***P*2$_1$/*c*, (no. 14)**	
Unit cell dimensions	a = 12.3998(10) Å	α = 90°.
	b = 7.8115(6) Å	β = 101.003(3)°.
	c = 15.2189(12) Å	γ = 90°.
Volume	1447.0(2) Å^3	
Z	4	
Density (calculated)	1.998 Mg·m^{-3}	
Absorption coefficient	1.279 mm^{-1}	
F(000)	864 e	
Crystal size	0.081 x 0.057 x 0.034 mm^3	
θ range for data collection	1.673 to 34.540°.	
Index ranges	$-19 \le h \le 19$, $-12 \le k \le 12$, $-24 \le l \le 24$	
Reflections collected	91991	

Independent reflections	6142 [R_{int} = 0.0347]
Reflections with I>2σ(I)	5536
Completeness to θ = 25.242°	100.0 %
Absorption correction	Gaussian
Max. and min. transmission	0.96465 and 0.91146
Refinement method	Full-matrix least-squares on F^2
Data / restraints / parameters	6142 / 0 / 209
Goodness-of-fit on F^2	1.066
Final R indices [I>2σ(I)]	R_1 = 0.0179	wR^2 = 0.0437
R indices (all data)	R_1 = 0.0225	wR^2 = 0.0455
Extinction coefficient	n/a
Largest diff. peak and hole	0.561 and −0.865 e·Å^{-3}.

Selected Bond lengths [Å]

Distances between phenoxo ligand and Ru-central atom are shown. Distance between carbonyl carbon (C1) and Ru-central atom in bold to highlight the elongated bond resulting from the *η*^{5}-coordination of the phenoxo ligand. For atom numbers see Fig S83.

Ru(1)–C(1): 2.4953
Ru(1)–C(2): 2.2223
Ru(1)–C(3): 2.1942
Ru(1)–C(4): 2.2097
Ru(1)–C(5): 2.1861
Ru(1)–C(6): 2.2054

[η^6-Benzene-η^5-(4-methoxy-1-phenoxo)Ru](OTf) (1i)

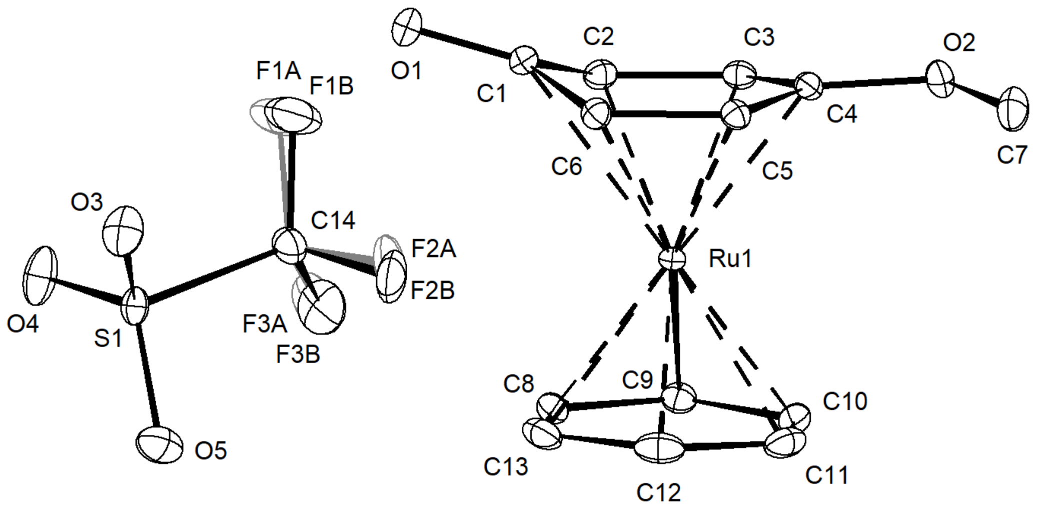

Fig S84: X-ray structure of compound **1i**.

Table S11 Crystal data and structure refinement of compound **1i**:

Identification code	14697	
Empirical formula	$C_{14}H_{13}F_3O_5RuS$	
Color	yellow	
Formula weight	451.37 g·mol^{-1}	
Temperature	100(2) K	
Wavelength	0.71073 Å	
Crystal system	Monoclinic	
Space group	***P*2$_1$/*c*, (no. 14)**	
Unit cell dimensions	a = 10.4902(6) Å	α = 90°.
	b = 8.7025(5) Å	β = 95.338(2)°.
	c = 16.5627(10) Å	γ = 90°.
Volume	1505.47(15) Å^3	
Z	4	
Density (calculated)	1.991 Mg·m^{-3}	
Absorption coefficient	1.238 mm^{-3}	
F(000)	896 e	
Crystal size	0.315 x 0.311 x 0.06 mm^3	
θ range for data collection	2.470 to 52.156°.	
Index ranges	−23 ≤ h ≤ 23, −19 ≤ k ≤ 19, −36 ≤ l ≤ 36	

Reflections collected	656152	
Independent reflections	17310 [R_{int} = 0.0419]	
Reflections with I>2σ(I)	16353	
Completeness to θ = 25.242°	99.9 %	
Absorption correction	Gaussian	
Max. and min. transmission	0.929 and 0.697	
Refinement method	Full-matrix least-squares on F^2	
Data / restraints / parameters	17310 / 0 / 246	
Goodness-of-fit on F^2	1.131	
Final R indices [I>2σ(I)]	R_1 = 0.0180	wR^2 = 0.0450
R indices (all data)	R_1 = 0.0198	wR^2 = 0.0457
Extinction coefficient	n/a	
Largest diff. peak and hole	0.982 and −1.084 e·Å^{-3}.	

Selected Bond lengths [Å]

Distances between phenoxo ligand and Ru-central atom are shown. Distance between carbonyl carbon (C1) and Ru-central atom in bold to highlight the elongated bond resulting from the η^5-coordination of the phenoxo ligand. For atom numbers see Fig S84.

Ru(1)–C(1): 2.5450
Ru(1)–C(2): 2.2160
Ru(1)–C(3): 2.1935
Ru(1)–C(4): 2.2643
Ru(1)–C(5): 2.2007
Ru(1)–C(6): 2.2132

[η^6-Benzene-η^5-(2,6-dichloro-1-phenoxo)Ru](OTf) (1g)

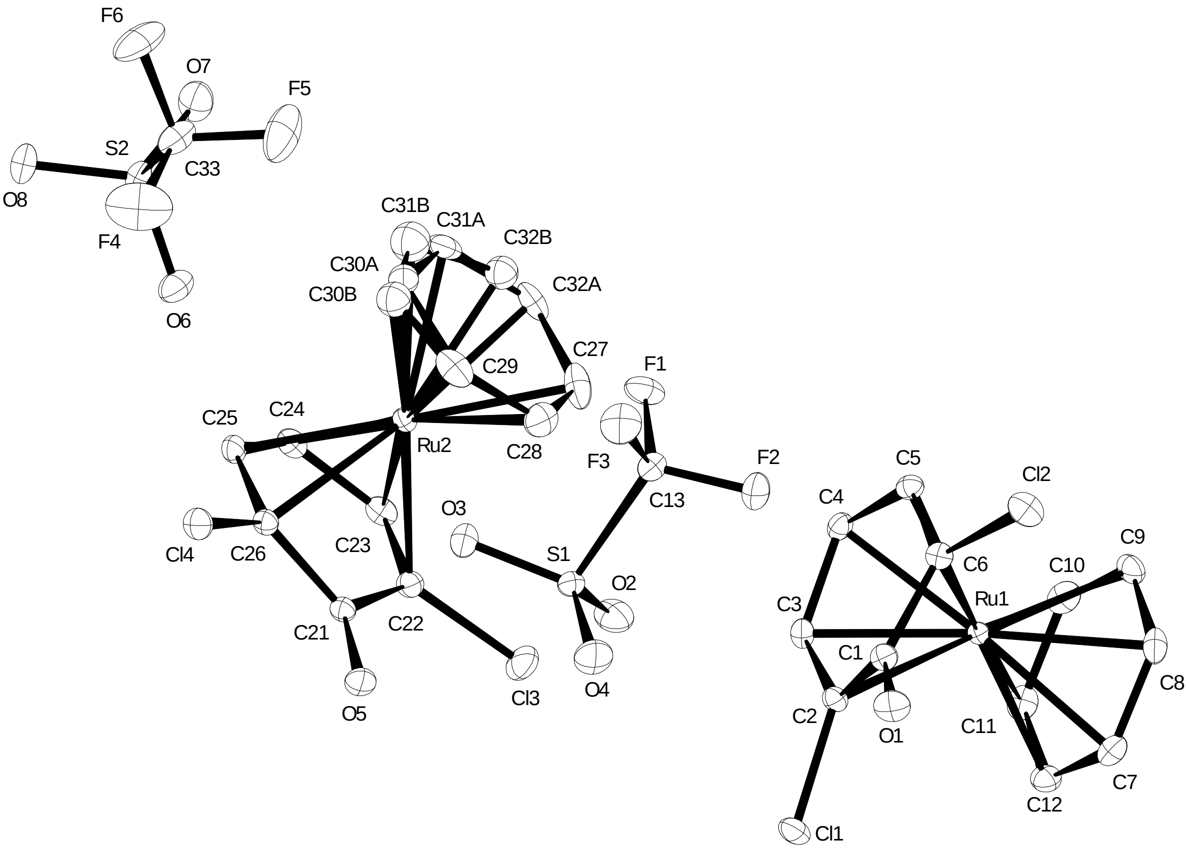

Fig S85: X-ray structure of compound **1g**.

Table S12 Crystal data and structure refinement of compound **1g**:

Identification code	14859	
Empirical formula	$C_{26}H_{18}Cl_4F_6O_8Ru_2S_2$	
Color	yellow	
Formula weight	980.46 g · mol^{-1}	
Temperature	100(2) K	
Wavelength	0.71073 Å	
Crystal system	MONOCLINIC	
Space group	**P2$_1$/c, (no. 14)**	
Unit cell dimensions	a = 14.7510(7) Å	α = 90°.
	b = 16.4841(8) Å	β = 104.099(2)°.
	c = 13.1324(6) Å	γ = 90°.
Volume	3097.0(3) Å^3	
Z	4	
Density (calculated)	2.103 Mg · m^{-3}	
Absorption coefficient	1.542 mm^{-1}	

F(000)	1920 e
Crystal size	0.182 x 0.117 x 0.101 mm^3
θ range for data collection	1.885 to 41.785°.
Index ranges	−27 ≤ h ≤ 27, −30≤ k ≤ 30, −24 ≤ l ≤ 24
Reflections collected	720757
Independent reflections	21368 [R_{int} = 0.0979]
Reflections with I>2σ(I)	17427
Completeness to θ = 25.242°	100.0 %
Absorption correction	Semi-empirical from equivalents
Max. and min. transmission	0.87 and 0.82
Refinement method	Full-matrix least-squares on F^2
Data / restraints / parameters	21368 / 0 / 445
Goodness-of-fit on F^2	1.029
Final R indices [I>2σ(I)]	R_1 = 0.0258 wR^2 = 0.0531
R indices (all data)	R_1 = 0.0401 wR^2 = 0.0568
Largest diff. peak and hole	1.8 and −1.4 e · $Å^{-3}$.

Selected Bond lengths [Å]

Distances between phenoxo ligand and Ru-central atom are shown. Distance between carbonyl carbon (C1) and Ru-central atom in bold to highlight the elongated bond resulting from the η^5-coordination of the phenoxo ligand. For atom numbers see Fig S85.

Ru(1)–C(1): 2.5578
Ru(1)–C(2): 2.2186
Ru(1)–C(3): 2.1995
Ru(1)–C(4): 2.1864
Ru(1)–C(5): 2.1977
Ru(1)–C(6): 2.2266

COMPUTATIONAL STUDIES

Geometry optimizations for local electrophilicity indices

The condensed local electrophilicities of structures shown in Scheme 4B of the main text were determined using the method reported by H. Shi with slight modifications.[2] Orca 4.2.1.[8] was used as a software for computational analysis. Geometry optimizations were accomplished at PBE0[9]/def2-SVP[10] level in the gas phase. Single point tasks were calculated at the BP86[11-12]/ZORA[13-15] ZORA-def2-TZVP[10, 16] level in the gas phase. The freeware program Multiwfn 3.7[17] (function 100-16) was used to analyze the different electronic states (N, N+1, N-1, N-2) obtained as .wfn files from the single point calculations. Multiwfn delivers a CDFT.txt file from which the electrophilicities can be read out directly.

Fluorobenzene

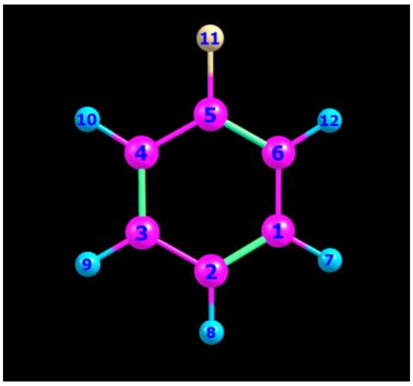

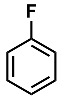

Fig S86: Structure of fluorobenzene. Left: With numbering of atoms respective to table S13.

Table S13 XYZ-coordinates for fluorobenzene:

Atom	X-Coordinate	Y-Coordinate	Z-Coordinate	Electrophilicity
C1	-1.119712000000	0.369004000000	0.000001000000	0.09381
C2	-2.219310000000	-0.488579000000	0.000001000000	0.05169
C3	-2.020747000000	-1.869061000000	0.000000000000	0.09344
C4	-0.730998000000	-2.396445000000	0.000001000000	0.09316
**C5**	**0.347942000000**	**-1.522134000000**	**0.000001000000**	**0.04661**
C6	0.176464000000	-0.143267000000	0.000001000000	0.09271
H7	-1.271560000000	1.451289000000	0.000000000000	
H8	-3.231339000000	-0.078357000000	0.000000000000	
H9	-2.877088000000	-2.546750000000	-0.000002000000	
H10	-0.542565000000	-3.472081000000	0.000000000000	
F11	1.581480000000	-2.022264000000	0.000000000000	
H12	1.055542000000	0.503535000000	0.000003000000	

4-Nitro-fluorobenzene

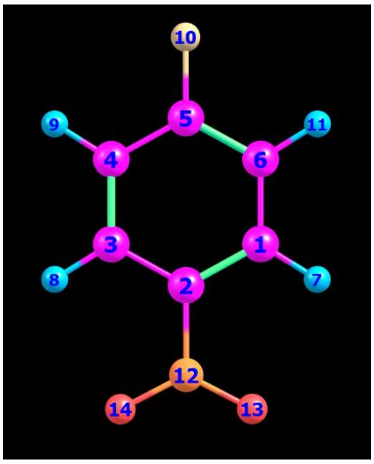

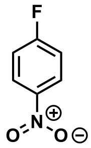

Fig S87: Structure of 4-nitrofluorobenzene. Left: With numbering of atoms respective to table S14.

Table S14 XYZ-coordinates for 4-nitrofluorobenzene:

Atom	X-Coordinate	Y-Coordinate	Z-Coordinate	Electrophilicity
C1	-1.157729000000	0.405706000000	-0.000002000000	0.09248
C2	-2.241639000000	-0.467648000000	-0.000003000000	0.07210
C3	-2.077956000000	-1.850129000000	-0.000002000000	0.09203
C4	-0.791691000000	-2.373730000000	-0.000002000000	0.08616
**C5**	**0.290987000000**	**-1.499845000000**	**0.000003000000**	**0.12767**
C6	0.128787000000	-0.117277000000	-0.000001000000	0.08586
H7	-1.346212000000	1.479666000000	-0.000005000000	
H8	-2.959925000000	-2.490042000000	-0.000006000000	
H9	-0.605530000000	-3.449285000000	-0.000003000000	
F10	1.514899000000	-2.003148000000	0.000011000000	
H11	1.010270000000	0.525558000000	-0.000003000000	
N12	-3.598478000000	0.087386000000	-0.000005000000	
O13	-3.706606000000	1.293718000000	0.000006000000	
O14	-4.521586000000	-0.696339000000	0.000011000000	

[η^6-Fluorobenzene-η^5-(2,6-dichloro-1-phenoxo)Ru]$^+$ (3g)

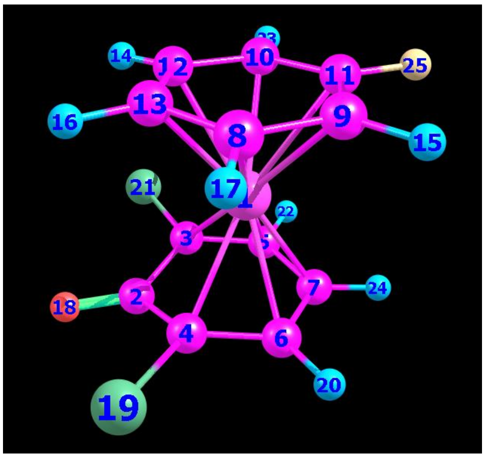

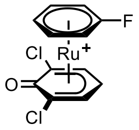

3g

Fig S88: Structure of **3g**. Left: With numbering of atoms respective to table S15.

Table S15 XYZ-coordinates for **3g**:

Atom	X-Coordinate	Y-Coordinate	Z-Coordinate	Electrophilicity
Ru1	-1.663211000000	0.864015000000	-0.270937000000	
C2	-2.704980000000	-1.275195000000	-1.305811000000	
C3	-1.281826000000	-0.902021000000	-1.551743000000	
C4	-3.056109000000	-0.829087000000	0.073112000000	
C5	-0.308055000000	-0.846219000000	-0.524861000000	
C6	-2.114338000000	-0.760773000000	1.126519000000	
C7	-0.725303000000	-0.767784000000	0.827380000000	
C8	-2.973768000000	2.536356000000	0.265498000000	0.15374
C9	-1.742427000000	2.741812000000	0.933885000000	0.32044
C10	-0.564029000000	2.516796000000	-1.222193000000	0.16867
**C11**	**-0.547549000000**	**2.743176000000**	**0.176173000000**	**0.14366**
C12	-1.811841000000	2.355711000000	-1.884760000000	0.13229
C13	-3.016232000000	2.367026000000	-1.146949000000	0.17941
H14	-1.827219000000	2.130409000000	-2.953538000000	
H15	-1.690769000000	2.859135000000	2.018350000000	
H16	-3.965726000000	2.157023000000	-1.643223000000	
H17	-3.895385000000	2.458894000000	0.846237000000	
O18	-3.486296000000	-1.635386000000	-2.140766000000	
Cl19	-4.726817000000	-0.697836000000	0.431368000000	
H20	-2.458744000000	-0.619164000000	2.153804000000	
Cl21	-0.771879000000	-0.868741000000	-3.188655000000	
H22	0.750707000000	-0.769608000000	-0.784265000000	
H23	0.380427000000	2.459681000000	-1.767578000000	

H24	0.011605000000	-0.664998000000	1.626203000000	
F25	0.598965000000	2.900518000000	0.789830000000	

[η^6-Fluorobenzene-η^5-(pentamethylcyclopentadienyl)Ru]$^+$ (3-Cp*)

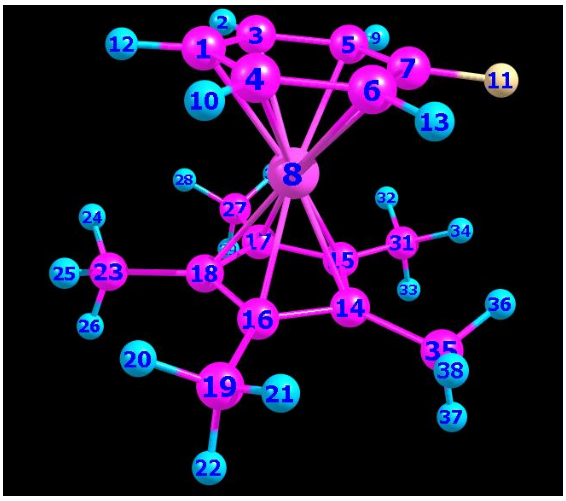

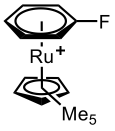

3-Cp*

Fig S89: Structure of **3-Cp***. Left: With numbering of atoms respective to table S16.

Table S16 XYZ-coordinates for **3-Cp***:

Atom	X-Coordinate	Y-Coordinate	Z-Coordinate	Electrophilicity
C1	5.637778000000	0.545912000000	0.530744000000	0.09549
H2	4.364021000000	1.619755000000	-0.876049000000	
C3	4.403462000000	1.063329000000	0.061873000000	0.10873
C4	5.702095000000	-0.118022000000	1.782122000000	0.10904
C5	3.225814000000	0.909727000000	0.837633000000	0.23361
C6	4.530181000000	-0.276412000000	2.565611000000	0.23760
**C7**	**3.304184000000**	**0.227545000000**	**2.073384000000**	**0.09997**
Ru8	4.783371000000	1.862487000000	2.079763000000	
H9	2.269858000000	1.329123000000	0.519272000000	
H10	6.660712000000	-0.470785000000	2.166370000000	
F11	2.218270000000	0.115551000000	2.814775000000	
H12	6.550052000000	0.711283000000	-0.044597000000	
H13	4.557152000000	-0.751464000000	3.547775000000	
C14	4.651770000000	2.941534000000	3.958849000000	
C15	3.883403000000	3.630518000000	2.959233000000	
C16	6.023193000000	2.907425000000	3.520975000000	
C17	4.778733000000	4.024742000000	1.902276000000	
C18	6.101986000000	3.579747000000	2.251203000000	
C19	7.171998000000	2.350310000000	4.289211000000	
H20	7.985381000000	2.027487000000	3.626225000000	
H21	6.870968000000	1.493391000000	4.906320000000	
H22	7.580992000000	3.117474000000	4.966430000000	

C23	7.346565000000	3.844948000000	1.475159000000	
H24	7.148434000000	3.908240000000	0.397097000000	
H25	8.103526000000	3.067028000000	1.639770000000	
H26	7.786962000000	4.805664000000	1.788095000000	
C27	4.415418000000	4.832628000000	0.703693000000	
H28	5.081216000000	4.625045000000	-0.144251000000	
H29	4.499179000000	5.906780000000	0.935440000000	
H30	3.382075000000	4.644771000000	0.383626000000	
C31	2.426680000000	3.934400000000	3.041329000000	
H32	1.982643000000	4.071699000000	2.046760000000	
H33	2.267477000000	4.865964000000	3.608069000000	
H34	1.874928000000	3.136049000000	3.555296000000	
C35	4.125210000000	2.414803000000	5.249990000000	
H36	3.094244000000	2.050013000000	5.149114000000	
H37	4.121830000000	3.213136000000	6.009629000000	
H38	4.742989000000	1.593413000000	5.636367000000	

Geometry optimizations for arene exchange mechanism

Gibbs free energy calculations were carried out with a development version of ORCA.[18] Geometry optimizations and frequency calculations were performed using the PBE0[9]-D3BJ[19–20] functional, where scalar relativistic effects were considered at the zeroth order regular approximation (ZORA) level.[13–15] The SARC-ZORA-TZVP,[21] ma-ZORA-def2-TZVP,[22] and ZORA-def2-TZVP[10, 16] basis sets were used for Ru, O and all other atoms, respectively, with SARC/J[16, 23] as the Coulomb auxiliary basis set (except for O where the AutoAux method[24] was used to generate the Coulomb auxiliary basis). Solvation effects of $MeNO_2$ were taken into account at the CPCM level.[25] The RIJCOSX method[26–29] was used to accelerate the calculations. All optimized structures were verified to have the correct number of imaginary frequencies (0 for minima, 1 for transition states). When a species has multiple low-lying conformers, the lowest conformer was located by a conformation/configuration search by the DOCKER (when the species is a non-covalent complex) or GOAT module of ORCA, at the GFN2-xTB level of theory.[30] IGMH calculations were performed by Multiwfn 3.8(dev),[17] using the converged SCF wavefunctions of the geometry optimization runs.

Single point energies were calculated at the DLPNO-CCSD(T1)[31–33] level of theory, with the x2c-QZVPPall[34] basis set on Ru and Br, and jun-cc-pV(T+d)Z[35] for other elements. The TightPNO keyword was used to minimize error due to pair natural orbital (PNO) cutoffs. The SARC/J and aug-cc-pVTZ/C[36] basis sets were used as the Coulomb and correlation auxiliary basis sets, respectively, except for Ru where the AutoAux method was used to generate the correlation auxiliary basis. Scalar relativistic effects were accounted for at the spin-free X2C level[37–40] (together with a finite nucleus treatment of the nuclear attraction potential), and solvation effects were described at the SMD level.[41]

In addition to the mechanism shown in Scheme 3B, where the benzene ligand dissociates directly from **II** to yield **III**, the κ^2-OMs ligand of **II** may also reversibly isomerize to a κ^1 configuration (giving **VI**), allowing a solvent ($MeNO_2$) molecule to coordinate to the Ru center and substitute the benzene ligand via an associative mechanism (Fig S90). Despite the extra electron donating ability that an extra $MeNO_2$ ligand can provide, the dissociative mechanism shown in Scheme 3B was found to be more facile than the associative one, due to entropy reasons. Nevertheless, intermediate **VIII** may also be generated by reversible coordination of **III** with $MeNO_2$, which stabilizes the vacant coordination site of **III**.

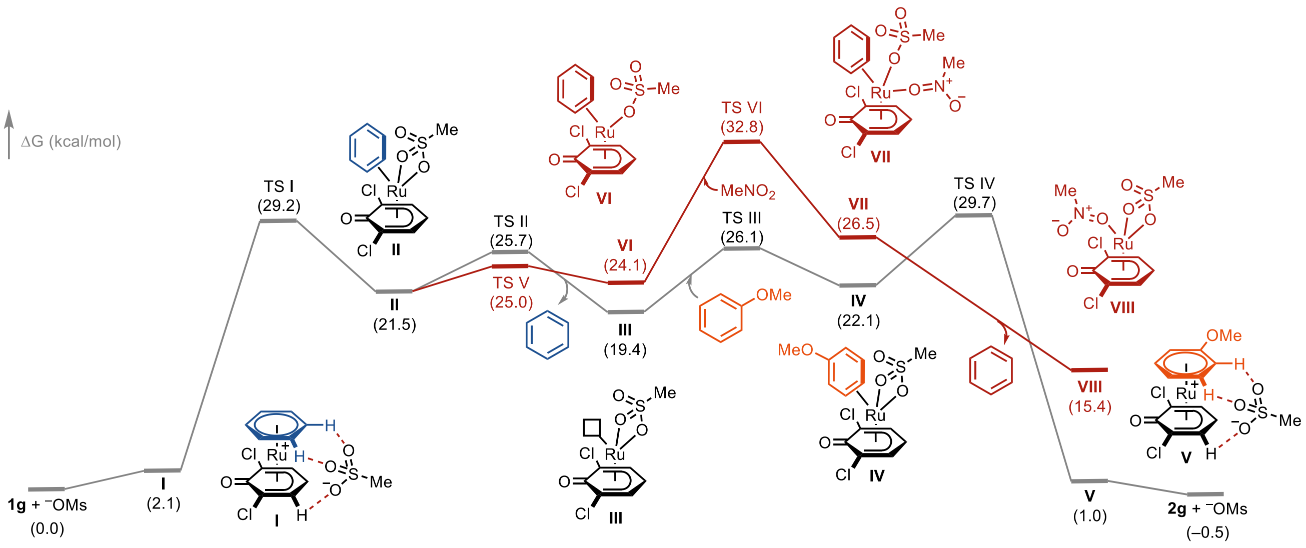

Fig S90: Gibbs free energy profile of the arene exchange reaction of **1g** with anisole. The solvent assisted pathway is highlighted in red.

Geometry of [η^6-benzene-η^5-(2,6-dichloro-1-phenoxo)Ru]$^+$ (1g)

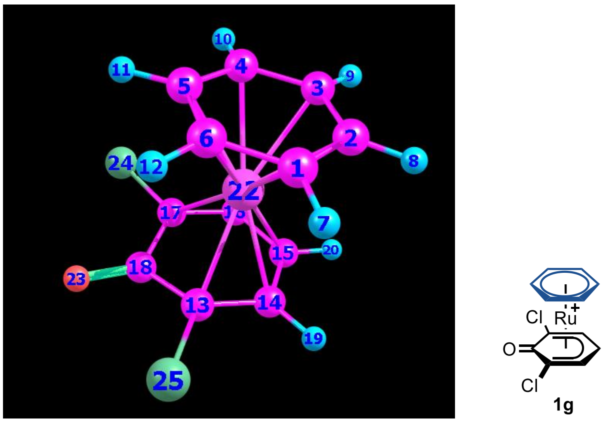

Fig S91: Structure of **1g**. Left: With numbering of atoms respective to table S17.

Table S17 XYZ-coordinates for **1g**:

Atom	X-Coordinate	Y-Coordinate	Z-Coordinate
C1	1.991243379	11.46722051	-1.325215616
C2	3.34283251	11.86979631	-1.293588798
C3	3.665390968	13.23660461	-1.160890375
C4	2.635041342	14.19451135	-1.064259244
C5	1.279744834	13.78738718	-1.062632868
C6	0.958881691	12.42547646	-1.191768728
H7	1.742051197	10.42977008	-1.505968168
H8	4.127174145	11.13974283	-1.444627592
H9	4.697695643	13.55737263	-1.210512917

H10	2.879806697	15.24853544	-1.046829108
H11	0.493116029	14.53037687	-1.04439942
H12	-0.075635292	12.11829689	-1.274313273
C13	1.147762037	12.221316	-4.629571407
C14	2.503114267	11.85226551	-4.733667911
C15	3.502767954	12.84551657	-4.681858654
C16	3.126750177	14.19387826	-4.510372254
C17	1.762518689	14.52964922	-4.410105701
C18	0.672707431	13.60663056	-4.759003359
H19	2.773278557	10.80454355	-4.772535266
H20	4.548257147	12.56854034	-4.69808236
H21	3.880171786	14.95967812	-4.376175013
Ru22	2.278050382	13.00027797	-2.878660078
O23	-0.487108385	13.93173623	-4.929296726
Cl24	1.33055297	16.16763467	-4.143241286
Cl25	-0.047410514	10.99154516	-4.636653651

Geometry of [η^6-benzene-η^5-(2,6-dichloro-1-phenoxo)Ru]OMs (I)

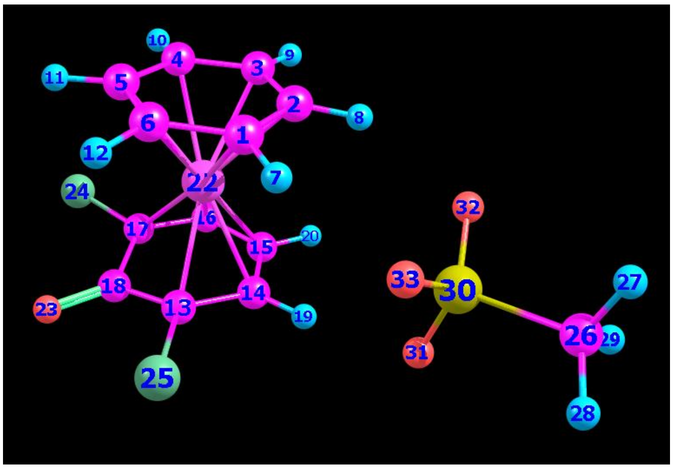

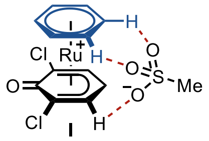

Fig S92: Structure of **I**. Left: With numbering of atoms respective to table S18.

Table S18 XYZ-coordinates for **I**:

Atom	X-Coordinate	Y-Coordinate	Z-Coordinate
C1	1.729212976	11.59248758	-1.37429369
C2	3.118269882	11.84043362	-1.367299202
C3	3.595246417	13.15984453	-1.220972391
C4	2.68181336	14.2263816	-1.086396685
C5	1.289482606	13.97378957	-1.060375866
C6	0.814807511	12.65845015	-1.203245879

H7	1.379250907	10.58777116	-1.584610686
H8	3.796281476	11.01901239	-1.569186947
H9	4.655449187	13.36519985	-1.291647828
H10	3.043515319	15.24594757	-1.058630193
H11	0.592330158	14.80028169	-1.014056462
H12	-0.249463303	12.47280491	-1.269919037
C13	0.916354203	12.47664568	-4.641771804
C14	2.219309987	11.95970598	-4.777319014
C15	3.320042785	12.84070192	-4.737103259
C16	3.098205988	14.22042746	-4.546636508
C17	1.780687468	14.70126383	-4.415401878
C18	0.590216317	13.90549335	-4.745112362
H19	2.377750951	10.88417035	-4.816818203
H20	4.328753805	12.45149736	-4.773609363
H21	3.932535162	14.89904367	-4.422211671
Ru22	2.154909091	13.11129731	-2.908765878
O23	-0.532133868	14.35738915	-4.884444269
Cl24	1.535817404	16.37539619	-4.127065699
Cl25	-0.406938943	11.38255622	-4.631405745
C26	3.282111302	6.661069917	-3.294266216
H27	3.39955528	6.299921855	-2.273173502
H28	2.445959186	6.158596022	-3.779202194
H29	4.199280757	6.501026086	-3.859909681
S30	2.945233016	8.398003198	-3.242609953
O31	2.797037788	8.822983398	-4.637153354
O32	4.098210498	9.017351788	-2.587033844
O33	1.708554449	8.550582333	-2.474503688

Geometry of TS I

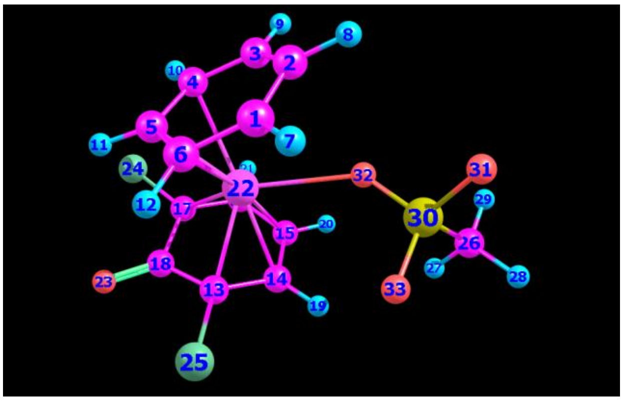

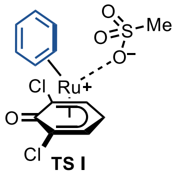

TS I

Fig S93: Structure of **TS I**. Left: With numbering of atoms respective to table S19.

Table S19 XYZ-coordinates for **TS I**:

Atom	X-Coordinate	Y-Coordinate	Z-Coordinate
C1	2.97561	11.38477	-1.00266
C2	3.987009	12.22373	-0.56423
C3	3.844924	13.60061	-0.72505
C4	2.633606	14.14676	-1.17388
C5	1.553299	13.30057	-1.49826
C6	1.776199	11.91403	-1.50998
H7	3.112802	10.31078	-0.98701
H8	4.914994	11.81154	-0.1942
H9	4.665347	14.26559	-0.48439
H10	2.508243	15.22067	-1.21785
H11	0.583528	13.70936	-1.75071
H12	0.986677	11.24858	-1.83555
C13	1.828613	12.36491	-4.90689
C14	3.196411	12.40726	-5.25549
C15	3.914222	13.60832	-5.09923
C16	3.263242	14.73045	-4.55748
C17	1.878414	14.64633	-4.25273
C18	1.002437	13.56976	-4.73316
H19	3.703661	11.48864	-5.51777
H20	4.983026	13.62599	-5.26215
H21	3.816197	15.62861	-4.31247
Ru22	2.936262	13.09581	-3.21427
O23	-0.21342	13.59628	-4.72734
Cl24	1.098245	16.03528	-3.59978
Cl25	0.992343	10.867	-5.03301
C26	6.787811	10.72268	-4.61505
H27	6.352461	11.23681	-5.47163
H28	7.143709	9.734957	-4.90595
H29	7.60152	11.31094	-4.19298
S30	5.5357	10.52446	-3.38233
O31	6.164437	9.856727	-2.2504
O32	5.113677	11.90114	-3.04168
O33	4.461817	9.75269	-3.99656

Geometry of [η^2-benzene-η^5-(2,6-dichloro-1-phenoxo)- κ^2-OMs-Ru] (II)

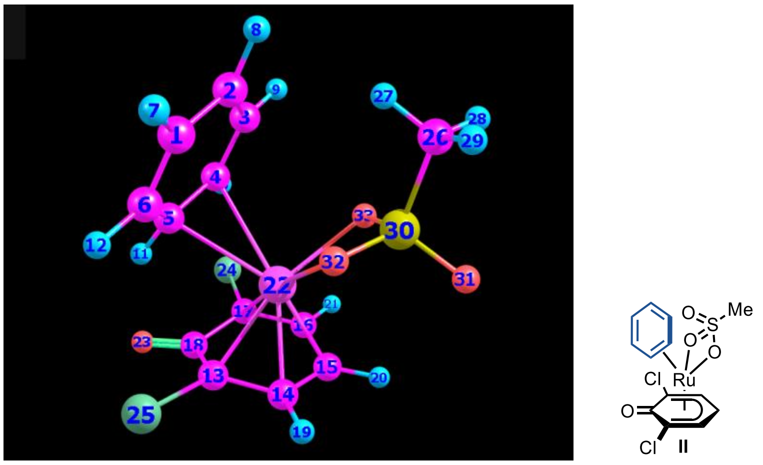

Fig S94: Structure of **II**. Left: With numbering of atoms respective to table S20.

Table S20 XYZ-coordinates for **II**:

Atom	X-Coordinate	Y-Coordinate	Z-Coordinate
C1	3.186949	11.13676	-0.51818
C2	4.339553	11.91644	-0.37239
C3	4.402341	13.17645	-0.92283
C4	3.295696	13.69925	-1.60854
C5	2.126156	12.93425	-1.7195
C6	2.090744	11.63677	-1.18269
H7	3.153513	10.1419	-0.08939
H8	5.185673	11.52323	0.179853
H9	5.296579	13.77826	-0.81627
H10	3.297638	14.74005	-1.91135
H11	1.20593	13.39184	-2.05864
H12	1.18484	11.04966	-1.26942
C13	1.140691	12.51849	-4.71794
C14	2.104822	12.08782	-5.66067
C15	3.078318	12.99024	-6.13477
C16	3.102033	14.29319	-5.58122
C17	2.117144	14.68318	-4.64823
C18	0.875535	13.93163	-4.43216
H19	2.149524	11.04195	-5.93974
H20	3.862123	12.65834	-6.80231
H21	3.926886	14.96136	-5.79754
Ru22	3.157356	12.89071	-4.00658
O23	-0.10646	14.35559	-3.84626

Cl24	2.197572	16.26963	-3.97559
Cl25	-0.01742	11.3735	-4.14935
C26	6.718261	11.0408	-2.80277
H27	6.388037	11.33603	-1.81138
H28	7.671752	11.50592	-3.05193
H29	6.791367	9.956929	-2.88746
S30	5.551636	11.58277	-3.98917
O31	6.027342	11.16804	-5.28312
O32	4.217374	11.01522	-3.62395
O33	5.383516	13.05583	-3.84065

Geometry of TS II

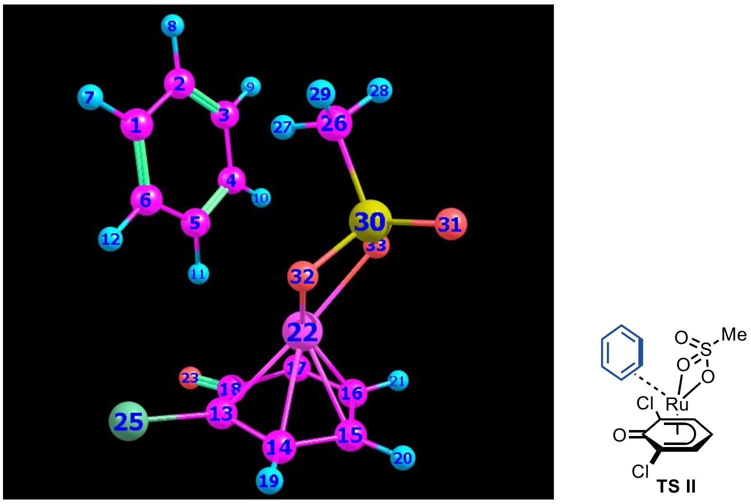

Fig S95: Structure of **TS II**. Left: With numbering of atoms respective to table S21.

Table S21 XYZ-coordinates for **TS II**:

Atom	X-Coordinate	Y-Coordinate	Z-Coordinate
C1	3.400394	11.08509	-0.15139
C2	4.345674	11.92932	0.419085
C3	4.483432	13.23541	-0.03589
C4	3.662441	13.70492	-1.05169
C5	2.70202	12.86601	-1.60639
C6	2.577795	11.55256	-1.1668
H7	3.304581	10.06351	0.198128
H8	4.98582	11.56413	1.214281
H9	5.230162	13.88674	0.4036
H10	3.757198	14.7253	-1.40511
H11	2.005141	13.25693	-2.33888

H12	1.831158	10.90308	-1.60924
C13	1.063209	12.50973	-5.01283
C14	1.774732	12.15558	-6.17598
C15	2.685401	13.07977	-6.74476
C16	2.877356	14.32743	-6.10196
C17	2.140023	14.62932	-4.9401
C18	0.939538	13.8906	-4.52894
H19	1.712888	11.14519	-6.5613
H20	3.299585	12.79677	-7.58982
H21	3.666515	14.99289	-6.42996
Ru22	3.195686	12.75242	-4.73358
O23	0.137572	14.26977	-3.69393
Cl24	2.472986	16.10251	-4.10854
Cl25	0.046266	11.32905	-4.27431
C26	6.14855	11.19479	-2.62838
H27	5.41267	11.54246	-1.90706
H28	7.096638	11.71817	-2.50685
H29	6.29262	10.11706	-2.55624
S30	5.56348	11.53989	-4.23559
O31	6.525619	11.08673	-5.19449
O32	4.206914	10.91192	-4.3759
O33	5.264449	13.00993	-4.30702

Geometry of [η^5-(2,6-dichloro-1-phenoxo)- κ^2-OMs-Ru] (III)

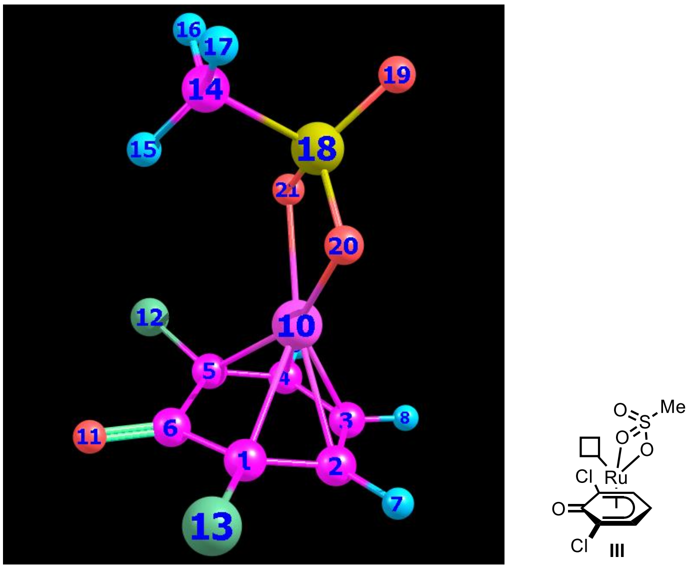

Fig S96: Structure of **III**. Left: With numbering of atoms respective to table S22.

Table S22 XYZ-coordinates for **III**:

Atom	X-Coordinate	Y-Coordinate	Z-Coordinate
C1	1.332747	12.38642	-5.15835
C2	1.630602	12.23297	-6.52826
C3	2.341497	13.26025	-7.18684
C4	2.743238	14.40955	-6.47212
C5	2.417148	14.50319	-5.10312
C6	1.398129	13.67111	-4.44494
H7	1.428973	11.2956	-7.0305
H8	2.68622	13.11038	-8.20246
H9	3.395494	15.14178	-6.93036
Ru10	3.423403	12.66164	-5.45661
O11	0.916325	13.8897	-3.34932
Cl12	3.039931	15.82599	-4.1947
Cl13	0.603358	11.07238	-4.3189
C14	5.611007	11.48806	-2.60637
H15	4.695879	11.94912	-2.23901
H16	6.49111	12.03867	-2.27509
H17	5.675442	10.44265	-2.30542
S18	5.600428	11.5257	-4.35393
O19	6.794624	10.92187	-4.85431
O20	4.322032	10.87262	-4.80722
O21	5.39186	12.95701	-4.77058

Geometry of TS III

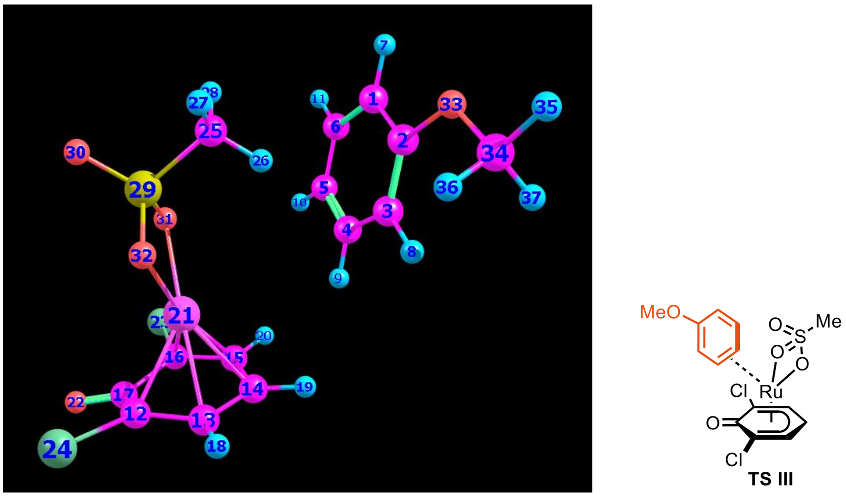

Fig S97: Structure of **TS III**. Left: With numbering of atoms respective to table S23.

Table S23 XYZ-coordinates for **TS III**:

Atom	X-Coordinate	Y-Coordinate	Z-Coordinate
C1	4.314499	11.7663	0.798421
C2	5.273995	12.55455	0.161434
C3	4.880202	13.47572	-0.80558
C4	3.530896	13.59757	-1.12152
C5	2.574777	12.81315	-0.49853
C6	2.978561	11.89603	0.466525
H7	4.638082	11.05202	1.546933
H8	5.605648	14.0977	-1.31244
H9	3.232472	14.32852	-1.86317
H10	1.528298	12.9124	-0.76061
H11	2.244843	11.27174	0.964138
C12	2.932611	13.34937	-6.83092
C13	3.728842	14.16516	-5.99568
C14	3.154608	14.68513	-4.82083
C15	1.807814	14.38877	-4.50295
C16	1.057345	13.58912	-5.38694
C17	1.461136	13.30408	-6.77104
H18	4.78893	14.27297	-6.18575
H19	3.7768	15.20573	-4.10346
H20	1.3925	14.67729	-3.54555
Ru21	2.862625	12.57397	-4.86319
O22	0.74003	12.83443	-7.63062
Cl23	-0.5357	13.12265	-4.9308
Cl24	3.673548	12.5805	-8.18081
C25	4.327362	10.0938	-2.37707
H26	4.506262	11.11707	-2.0511
H27	5.262825	9.565061	-2.55877
H28	3.713863	9.555199	-1.65596
S29	3.450624	10.13108	-3.88637
O30	3.196743	8.796671	-4.33036
O31	2.212102	10.96288	-3.67996
O32	4.236627	10.97165	-4.85451
O33	6.556323	12.35358	0.540677
C34	7.560482	13.12077	-0.09746
H35	8.505159	12.80518	0.340396
H36	7.576343	12.93171	-1.17477

H37	7.41498	14.19002	0.081146

Geometry of [η^2-anisole-η^5-(2,6-dichloro-1-phenoxo)- κ^2-OMs-Ru] (IV)

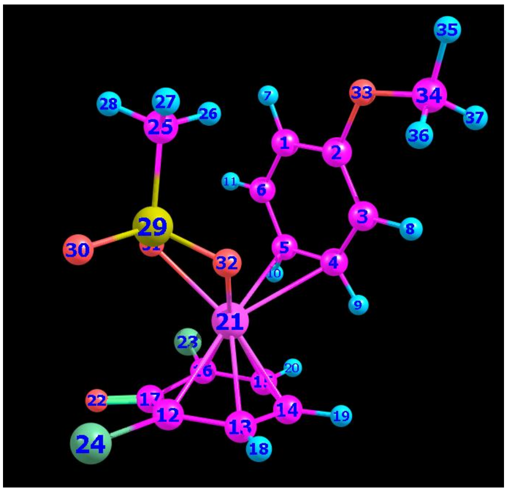

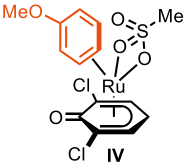

Fig S98: Structure of **IV**. Left: With numbering of atoms respective to table S24.

Table S24 XYZ-coordinates for **IV**:

Atom	X-Coordinate	Y-Coordinate	Z-Coordinate
C1	3.728646	11.81612	-0.12288
C2	4.917731	12.53452	-0.3607
C3	4.894693	13.68143	-1.13317
C4	3.68135	14.11502	-1.67649
C5	2.502437	13.37995	-1.48831
C6	2.552121	12.22003	-0.68904
H7	3.772752	10.93948	0.513305
H8	5.789055	14.26441	-1.3021
H9	3.645235	15.10069	-2.12132
H10	1.535915	13.81051	-1.71263
H11	1.642512	11.66242	-0.50195
C12	2.769598	12.84717	-6.03476
C13	3.59767	13.93083	-5.65729
C14	3.125875	14.90255	-4.75569
C15	1.848336	14.71558	-4.18525
C16	1.069111	13.59491	-4.54354
C17	1.335492	12.77968	-5.7359
H18	4.620807	13.96645	-6.0119
H19	3.764375	15.7126	-4.43073
H20	1.503465	15.37603	-3.39857

Ru21	2.992001	12.94119	-3.8718
O22	0.553451	11.98662	-6.23092
Cl23	-0.45233	13.35901	-3.76348
Cl24	3.370972	11.72635	-7.19441
C25	4.792862	9.509167	-2.34292
H26	4.670414	10.06483	-1.41978
H27	5.844903	9.313568	-2.54862
H28	4.233324	8.574602	-2.31218
S29	4.16998	10.44062	-3.68878
O30	4.372182	9.640753	-4.86569
O31	2.743838	10.78981	-3.41956
O32	4.858519	11.76468	-3.70837
O33	6.01804	12.02448	0.212349
C34	7.254397	12.68153	-0.01946
H35	8.007598	12.09413	0.500216
H36	7.482794	12.71251	-1.08803
H37	7.238563	13.69778	0.382456

Geometry of TS IV

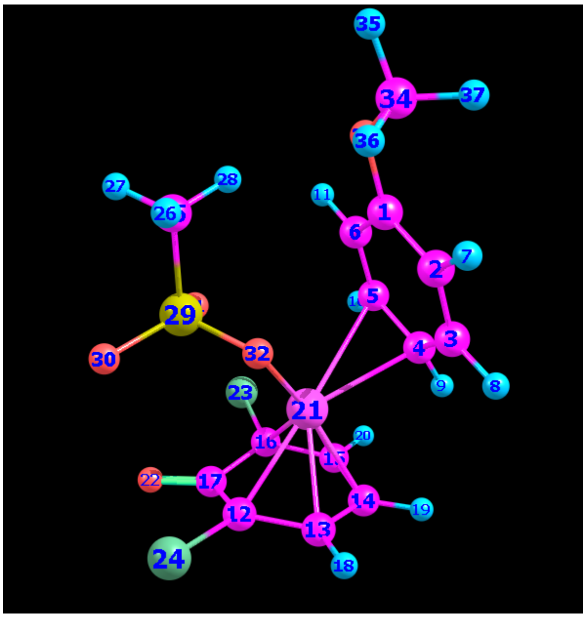

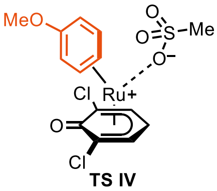

TS IV

Fig S99: Structure of **TS IV**. Left: With numbering of atoms respective to table S25.

Table S25 XYZ-coordinates for **TS IV**:

Atom	X-Coordinate	Y-Coordinate	Z-Coordinate
C1	4.60815	11.84647	-0.53517
C2	5.377457	12.94483	-0.93696

C3	4.756708	14.04693	-1.48439
C4	3.361974	14.0839	-1.65173
C5	2.605434	12.95426	-1.289
C6	3.231233	11.8431	-0.74138
H7	6.451558	12.94331	-0.81371
H8	5.347205	14.91404	-1.75571
H9	2.873018	15.03134	-1.82995
H10	1.523534	12.98481	-1.34587
H11	2.657495	10.97244	-0.44923
C12	3.262419	13.35174	-6.0042
C13	3.665628	14.53746	-5.34974
C14	2.783675	15.18394	-4.45306
C15	1.556502	14.57627	-4.13675
C16	1.254068	13.32974	-4.73924
C17	1.896465	12.83492	-5.97022
H18	4.663847	14.92746	-5.50592
H19	3.110866	16.0685	-3.92171
H20	0.917976	14.97555	-3.35924
Ru21	3.139341	13.18208	-3.81328
O22	1.452706	11.95411	-6.68683
Cl23	-0.16301	12.50662	-4.2197
Cl24	4.352033	12.6373	-7.12293
C25	4.759297	9.206143	-2.86048
H26	5.82709	9.200707	-3.07581
H27	4.331205	8.222369	-3.05065
H28	4.573676	9.512216	-1.83467
S29	3.980844	10.32673	-3.96712
O30	4.225334	9.828653	-5.30169
O31	2.573236	10.45023	-3.60076
O32	4.644033	11.65525	-3.7173
O33	5.119398	10.75261	0.050288
C34	6.525375	10.67938	0.237696
H35	6.711452	9.718071	0.71044
H36	7.04852	10.72509	-0.7209
H37	6.875919	11.48335	0.889205

Geometry of [η^6-anisole-η^5-(2,6-dichloro-1-phenoxo)-Ru]OMs (V)

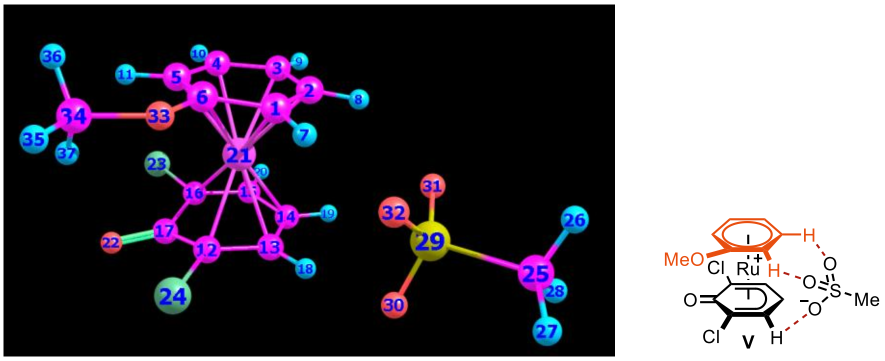

Fig S100: Structure of **V**. Left: With numbering of atoms respective to table S26.

Table S26 XYZ-coordinates for **V**:

Atom	X-Coordinate	Y-Coordinate	Z-Coordinate
C1	1.907445	11.66	-1.38
C2	3.264086	12.03812	-1.35903
C3	3.619074	13.39589	-1.21133
C4	2.602873	14.36559	-1.12772
C5	1.237511	13.99287	-1.13846
C6	0.883583	12.62302	-1.17847
H7	1.634482	10.62491	-1.56423
H8	4.015001	11.27899	-1.5356
H9	4.65619	13.69975	-1.25865
H10	2.859115	15.4172	-1.11789
H11	0.479822	14.76328	-1.12131
C12	0.921703	12.69473	-4.64852
C13	2.134761	11.98773	-4.77618
C14	3.353679	12.69415	-4.76622
C15	3.339384	14.09587	-4.60404
C16	2.106992	14.76943	-4.48425
C17	0.810656	14.15134	-4.79545
H18	2.134177	10.9004	-4.79211
H19	4.292732	12.15798	-4.79762
H20	4.265684	14.64646	-4.49991
Ru21	2.251484	13.18583	-2.94263
O22	-0.2331	14.76164	-4.94882
Cl23	2.112938	16.47058	-4.24143
Cl24	-0.54711	11.80676	-4.60271
C25	3.157567	6.747433	-3.24054

H26	3.463557	6.462329	-2.23459
H27	2.295978	6.159453	-3.5547
H28	3.982484	6.612981	-3.93929
S29	2.701176	8.457406	-3.22756
O30	2.299519	8.780859	-4.59899
O31	3.889137	9.19722	-2.80081
O32	1.592466	8.574717	-2.27742
O33	-0.35342	12.1664	-1.16564
C34	-1.42976	13.10733	-1.17486
H35	-2.33645	12.50941	-1.18835
H36	-1.40306	13.7268	-0.27703
H37	-1.38001	13.7286	-2.07105

Geometry of [η^6-anisole-η^5-(2,6-dichloro-1-phenoxo)-Ru]$^+$ (2g)

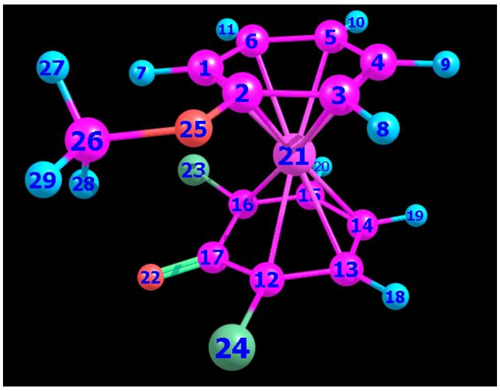

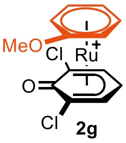

2g

Fig S101: Structure of **2g**. Left: With numbering of atoms respective to table S27.

Table S27 XYZ-coordinates for **2g**:

**Atom**	**X-Coordinate**	**Y-Coordinate**	**Z-Coordinate**
C1	2.295968	12.34286	-0.7651
C2	3.483175	11.73565	-1.24015
C3	4.505563	12.56822	-1.76975
C4	4.424741	13.97024	-1.65815
C5	3.283198	14.56877	-1.08368
C6	2.217585	13.75183	-0.66459
H7	1.442458	11.74491	-0.47917
H8	5.35122	12.10089	-2.2577
H9	5.215213	14.58491	-2.06811
H10	3.187679	15.64572	-1.05135
H11	1.296572	14.20232	-0.31755
C12	2.26842	11.9185	-4.4853

C13	3.127554	12.96618	-4.87633
C14	2.734622	14.30441	-4.67793
C15	1.492898	14.57218	-4.06354
C16	0.656158	13.50209	-3.68764
C17	0.853018	12.11934	-4.14618
H18	4.11631	12.73659	-5.25299
H19	3.41077	15.11351	-4.91887
H20	1.211767	15.58784	-3.81548
Ru21	2.65385	13.2584	-2.75826
O22	0.023823	11.23221	-4.06047
Cl23	-0.82902	13.84862	-2.89918
Cl24	2.795252	10.29897	-4.68647
O25	3.672925	10.43705	-1.3459
C26	2.598591	9.55723	-1.00003
H27	2.330322	9.679679	0.050417
H28	1.735833	9.745542	-1.64142
H29	2.97931	8.555154	-1.17422

Geometry of TS V

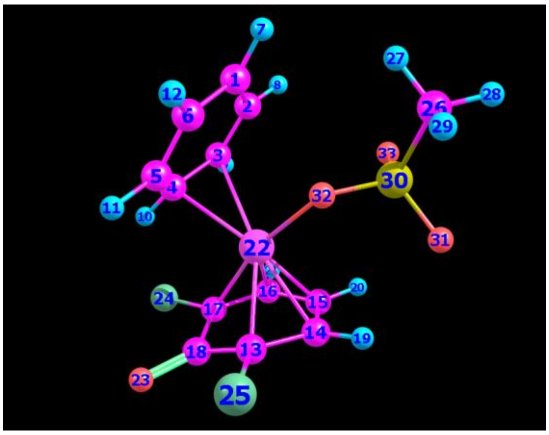

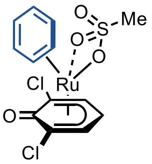

TS V

Fig S102: Structure of **TS V**. Left: With numbering of atoms respective to table S28.

Table S28 XYZ-coordinates for **TS V**:

Atom	X-Coordinate	Y-Coordinate	Z-Coordinate
C1	4.157744	11.9746	-0.56337
C2	4.656059	13.19824	-0.99501
C3	3.801612	14.16283	-1.53022
C4	2.421245	13.89834	-1.6259
C5	1.957655	12.6032	-1.33974
C6	2.825311	11.666	-0.76967

H7	4.822131	11.24711	-0.1143
H8	5.712329	13.41753	-0.90254
H9	4.177567	15.15019	-1.77019
H10	1.721814	14.69831	-1.82403
H11	0.903768	12.3761	-1.44578
H12	2.445939	10.68753	-0.50004
C13	1.586401	12.48916	-5.13062
C14	2.8749	12.46605	-5.70973
C15	3.698788	13.60113	-5.59481
C16	3.187992	14.74697	-4.93684
C17	1.851677	14.7712	-4.49325
C18	0.869698	13.73272	-4.79581
H19	3.285903	11.53489	-6.0761
H20	4.731317	13.56555	-5.91545
H21	3.83191	15.59606	-4.74198
Ru22	3.001262	13.06253	-3.64701
O23	-0.32628	13.809	-4.57377
Cl24	1.253294	16.21661	-3.77308
Cl25	0.664378	11.03691	-5.12155
C26	5.874591	9.51828	-2.98261
H27	5.896102	9.742847	-1.91775
H28	6.862764	9.220852	-3.33219
H29	5.143437	8.741074	-3.1991
S30	5.421395	10.97722	-3.85706
O31	5.388762	10.6517	-5.27135
O32	4.045051	11.25397	-3.31779
O33	6.356508	12.02737	-3.5035

Geometry of VI

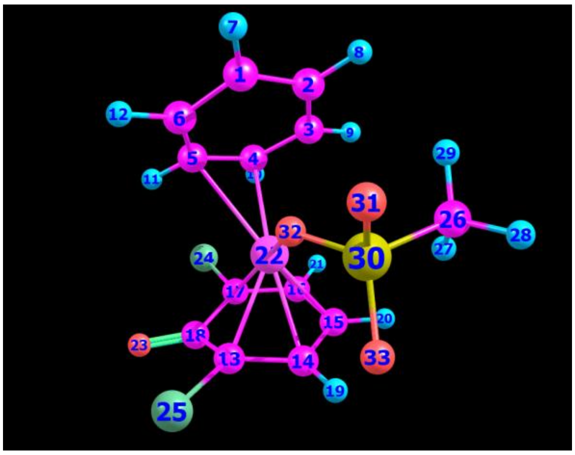

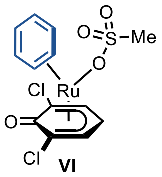

VI

Fig S103: Structure of **VI**. Left: With numbering of atoms respective to table S29.

Table S29 XYZ-coordinates for **VI**:

Atom	X-Coordinate	Y-Coordinate	Z-Coordinate
C1	3.408023723	11.45664295	-0.420154961
C2	4.474070922	12.30100273	-0.671396756
C3	4.270088161	13.53304467	-1.295391678
C4	2.965575997	13.96284711	-1.580842235
C5	1.891411241	13.06659539	-1.394672399
C6	2.126803884	11.81797213	-0.823647086
H7	3.573653933	10.4992848	0.057837475
H8	5.477937656	12.00560344	-0.390507477
H9	5.102549026	14.20663579	-1.458658916
H10	2.777088198	15.00067218	-1.818151649
H11	0.877483804	13.38927348	-1.598901045
H12	1.298142428	11.13553156	-0.676847029
C13	1.908388093	12.44890095	-5.366231218
C14	3.265314245	12.6329741	-5.723066826
C15	3.920260128	13.81339958	-5.33035268
C16	3.195750478	14.79873362	-4.613741491
C17	1.824246718	14.60808351	-4.36427822
C18	1.017215428	13.55391289	-4.985031921
H19	3.824787755	11.80799467	-6.143762517
H20	4.985226681	13.92668807	-5.488419062
H21	3.7028249	15.6670528	-4.211480558
Ru22	3.0212549	12.92741862	-3.604891826
O23	-0.198020763	13.49489322	-4.955980662
Cl24	0.955135145	15.82573043	-3.511596887
Cl25	1.156729134	10.94291515	-5.716672549
C26	6.304617543	11.10348186	-3.540386951
H27	6.212245357	12.04723303	-4.075907759
H28	7.130040177	10.51870089	-3.944608493
H29	6.448024577	11.27957974	-2.476385286
S30	4.831972093	10.16328167	-3.771013459
O31	4.949814168	8.954569685	-2.98301508
O32	3.724491776	10.99966367	-3.196569499
O33	4.679876875	9.951739222	-5.197398851

Geometry of TS VI

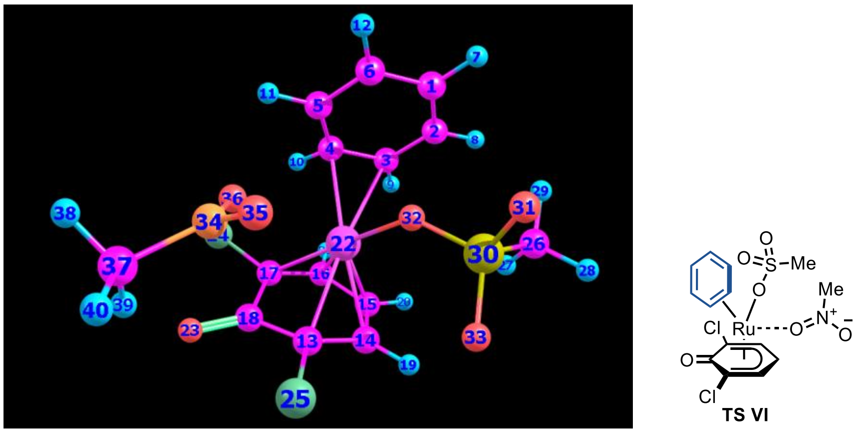

Fig S104: Structure of **TS VI**. Left: With numbering of atoms respective to table S30.

Table S30 XYZ-coordinates for **TS VI**:

Atom	X-Coordinate	Y-Coordinate	Z-Coordinate
C1	4.408531	11.9725	-0.27881
C2	4.966638	12.99922	-1.01676
C3	4.146737	13.94551	-1.63236
C4	2.751557	13.87452	-1.46546
C5	2.199279	12.81179	-0.73151
C6	3.025009	11.86867	-0.15011
H7	5.049829	11.24102	0.199127
H8	6.042222	13.08258	-1.11348
H9	4.591222	14.80134	-2.12592
H10	2.127073	14.71813	-1.72898
H11	1.126294	12.75901	-0.59804
H12	2.595097	11.05286	0.418966
C13	2.081151	12.46586	-5.60394
C14	3.433665	12.8578	-5.68554
C15	3.834782	14.0794	-5.0986
C16	2.86394	14.87839	-4.44607
C17	1.525892	14.44863	-4.40463
C18	0.99109	13.39527	-5.27002
H19	4.167931	12.16823	-6.08094
H20	4.880051	14.35929	-5.07354
H21	3.163196	15.7651	-3.9008
Ru22	2.872982	12.91153	-3.62873
O23	-0.19006	13.1888	-5.4758

Cl24	0.367988	15.404	-3.55718
Cl25	1.598973	10.95393	-6.26778
C26	6.268903	10.96375	-3.48602
H27	6.233545	11.9342	-3.97993
H28	7.017016	10.32563	-3.95522
H29	6.477964	11.07829	-2.42495
S30	4.709597	10.16874	-3.68183
O31	4.726464	8.954896	-2.89366
O32	3.707913	11.11375	-3.0728
O33	4.503108	9.985122	-5.10237
N34	0.025611	10.34931	-3.28183
O35	0.612245	9.300159	-3.14596
O36	0.42399	11.41662	-2.85521
C37	-1.26859	10.3524	-3.99531
H38	-2.03761	10.49832	-3.23686
H39	-1.2527	11.19664	-4.68088
H40	-1.38455	9.398913	-4.4988

Geometry of VII

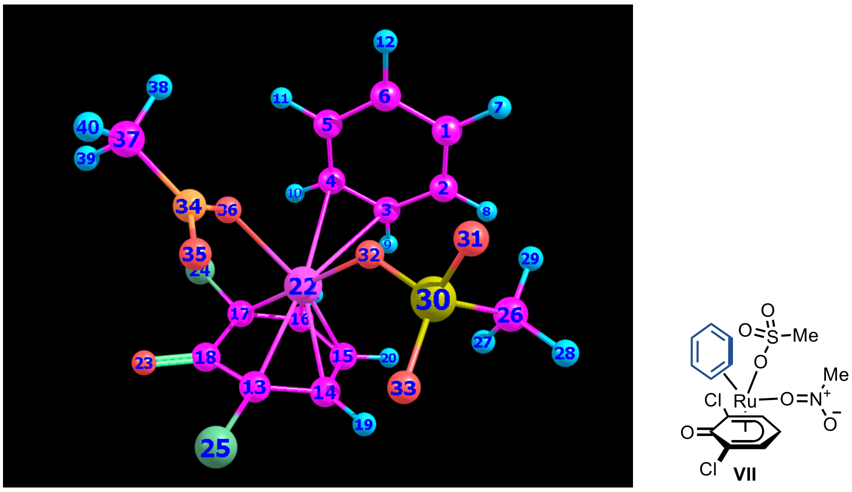

Fig S105: Structure of **VII**. Left: With numbering of atoms respective to table S31.

Table S31 XYZ-coordinates for **VII**:

Atom	X-Coordinate	Y-Coordinate	Z-Coordinate
C1	4.253268	11.91247	-0.22304
C2	4.728713	12.95344	-0.99456
C3	3.834205	13.81348	-1.62953
C4	2.456237	13.62968	-1.47508

C5	1.988127	12.57777	-0.67445
C6	2.879431	11.72331	-0.06279
H7	4.949135	11.24245	0.269523
H8	5.794459	13.11462	-1.10236
H9	4.2163	14.67746	-2.15745
H10	1.757608	14.37701	-1.83525
H11	0.922159	12.44668	-0.53545
H12	2.515027	10.90903	0.552762
C13	2.389147	12.61181	-6.08775
C14	3.723771	12.96426	-5.75777
C15	3.973135	14.09894	-4.96706
C16	2.857497	14.82438	-4.47978
C17	1.547658	14.44657	-4.8302
C18	1.244041	13.51934	-5.92026
H19	4.529341	12.30101	-6.04644
H20	4.977832	14.35258	-4.65697
H21	2.998684	15.62813	-3.76752
Ru22	2.615285	12.77837	-3.94914
O23	0.142672	13.35387	-6.41525
Cl24	0.222679	15.31933	-4.15132
Cl25	2.123107	11.2479	-7.10387
C26	6.092553	10.91317	-3.42334
H27	6.093297	11.88827	-3.90824
H28	6.885711	10.28765	-3.83156
H29	6.207497	11.02173	-2.34766
S30	4.563172	10.10119	-3.75432
O31	4.539831	8.886784	-2.96336
O32	3.49119	11.02016	-3.24718
O33	4.488278	9.908526	-5.18783
N34	0.343329	10.77546	-3.54711
O35	0.849205	10.04547	-4.34592
O36	0.814625	11.86357	-3.19203
C37	-0.9421	10.42282	-2.91781
H38	-0.82947	10.56549	-1.84552
H39	-1.68197	11.11905	-3.31174
H40	-1.17069	9.396869	-3.18392

Geometry of VIII

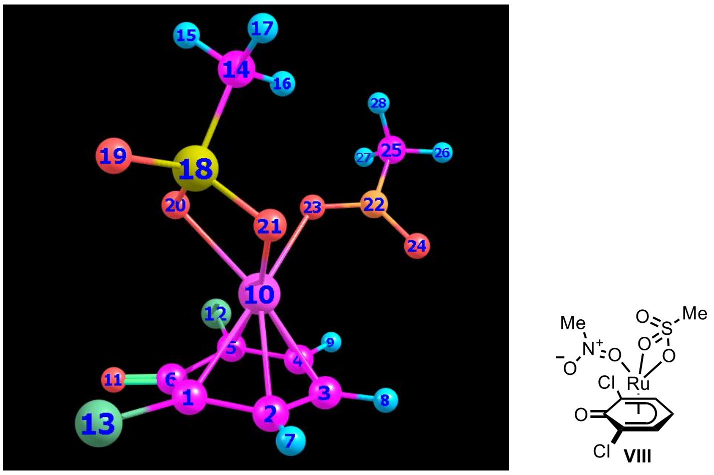

Fig S106: Structure of **VIII**. Left: With numbering of atoms respective to table S32.

Table S32 XYZ-coordinates for **VIII**:

Atom	X-Coordinate	Y-Coordinate	Z-Coordinate
C1	1.718548	12.84172	-5.89793
C2	2.974498	13.45143	-6.12578
C3	3.385232	14.55025	-5.33949
C4	2.53859	14.96422	-4.29029
C5	1.296422	14.31862	-4.08397
C6	0.659394	13.45165	-5.0837
H7	3.660899	13.00499	-6.8354
H8	4.369476	14.98022	-5.45966
H9	2.879602	15.7014	-3.57355
Ru10	2.869985	12.87407	-4.0962
O11	-0.5036	13.08679	-5.05684
Cl12	0.329334	14.80291	-2.73745
Cl13	1.284498	11.46416	-6.8435
C14	4.143292	9.737833	-2.0889
H15	3.469961	9.120066	-1.49542
H16	4.426036	10.63704	-1.54587
H17	5.022112	9.167127	-2.38719
S18	3.2895	10.2006	-3.5479
O19	2.918151	9.005921	-4.25107
O20	2.136087	11.06144	-3.14422
O21	4.17322	11.12429	-4.31827
N22	4.763111	13.7528	-1.91274
O23	3.746682	13.08512	-2.14743

O24	5.374476	14.35756	-2.74349
C25	5.191955	13.76665	-0.50394
H26	6.096152	14.36084	-0.43808
H27	4.372281	14.19419	0.071546
H28	5.351932	12.73085	-0.20907

Geometry of Benzene

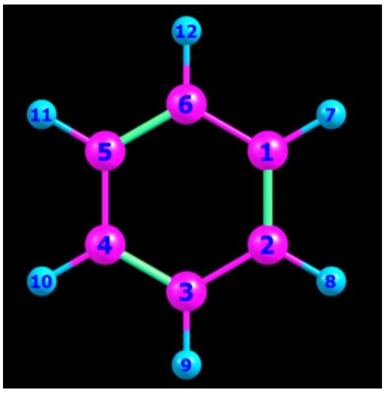

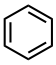

Fig S107: Structure of benzene. Left: With numbering of atoms respective to table S33.

Table S33 XYZ-coordinates for benzene:

Atom	X-Coordinate	Y-Coordinate	Z-Coordinate
C1	4.155267	12.75754	6.54E-05
C2	5.544229	12.75747	0.000491
C3	6.238781	13.96034	-6.8E-05
C4	5.54435	15.16329	-0.00105
C5	4.155388	15.16336	-0.00148
C6	3.460836	13.96048	-0.00092
H7	3.613003	11.81845	0.000506
H8	6.086399	11.81833	0.001262
H9	7.323177	13.96029	0.000262
H10	6.086614	16.10238	-0.00149
H11	3.613219	16.1025	-0.00225
H12	2.37644	13.96054	-0.00125

Geometry of Anisole

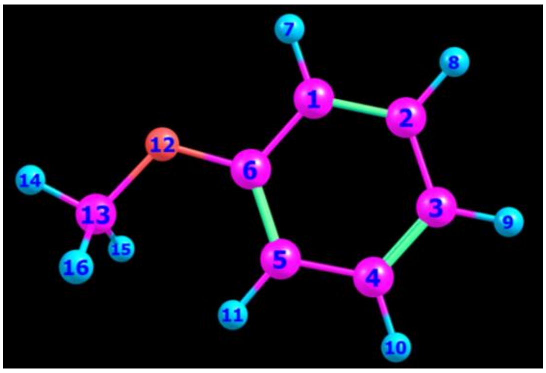

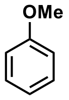

Fig S108: Structure of anisole. Left: With numbering of atoms respective to table S34.

Table S34 XYZ-coordinates for anisole:

Atom	X-Coordinate	Y-Coordinate	Z-Coordinate
C1	4.22994	12.67641	-0.03229
C2	5.584815	12.74269	0.235326
C3	6.26084	13.95716	0.16839
C4	5.55817	15.10173	-0.1705
C5	4.195085	15.05238	-0.44281
C6	3.527738	13.83288	-0.3732
H7	3.694801	11.73485	0.017477
H8	6.119096	11.83666	0.499213
H9	7.322626	14.00641	0.378548
H10	6.069841	16.05625	-0.22736
H11	3.669428	15.9606	-0.70564
O12	2.20598	13.67477	-0.6185
C13	1.458137	14.82379	-0.96932
H14	0.435497	14.48416	-1.12018
H15	1.479983	15.56973	-0.1695
H16	1.832916	15.2732	-1.8936

Geometry of $^{-}$OMs

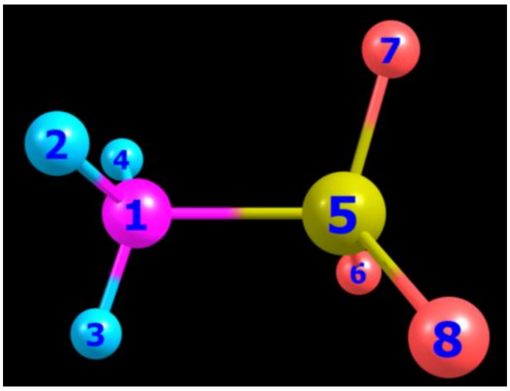

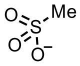

Fig S109: Structure of $^{-}$OMs. Left: With numbering of atoms respective to table S35.

Table S35 XYZ-coordinates for $^{-}$OMs:

Atom	X-Coordinate	Y-Coordinate	Z-Coordinate
C1	-7.7E-08	-0.00025	-1.64554
H2	-0.89372	0.516116	-1.99433
H3	-2.5E-06	-1.03214	-1.99502
H4	0.893725	0.516112	-1.99433
S5	-1.9E-07	-0.00022	0.127509
O6	1.21898	-0.70337	0.53031
O7	-2.6E-06	1.407129	0.530063
O8	-1.21898	-0.70337	0.53031

Geometry of $MeNO_2$

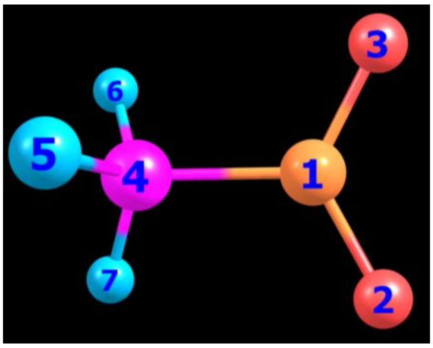

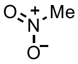

Fig S110: Structure of $MeNO_2$. Left: With numbering of atoms respective to table S36.

Table S36 XYZ-coordinates for $MeNO_2$:

Atom	X-Coordinate	Y-Coordinate	Z-Coordinate
N1	-7.3182	6.287632	-0.00726
O2	-6.11436	6.252178	-0.14481
O3	-7.95751	7.303655	0.162692

C4	-8.05765	5.00584	-0.01533
H5	-8.2118	4.736606	1.029804
H6	-9.01272	5.174038	-0.50425
H7	-7.44965	4.263102	-0.52084

Additional computational results of the arene exchange reaction

Interactions of 1g with $^{-}$OTf

To gain insight into the arene exchange reaction in the absence of $^{-}$OMs, in addition to calculating the activation free energy of TS I where $^{-}$OMs has been replaced by $^{-}$OTf (TS VIII, as mentioned in the main text), we have also calculated the IGMH plot of the complex between **1g** and $^{-}$OTf (**IX**), shown below. It is evident that $^{-}$OTf can form C–H…O interactions with **1g** in a similar way as in **I** (Scheme 2B). However, the complexation Gibbs free energy (+3.9 kcal/mol) is higher than that of **I** (+2.1 kcal/mol), in accord with the lower basicity of $^{-}$OTf, as well as the experimental observation that $^{-}$OMs binds more readily with **1g** than $^{-}$OTf does.

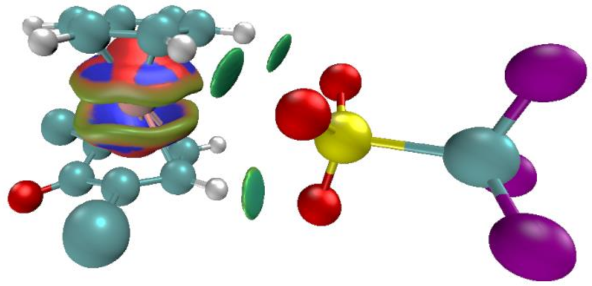

Fig S111: IGMH plot (isovalue=0.01) of **IX**.

Intermediates with *η¹* or *η³*-coordinated phenoxo ligands

To rule out the possibility that the phenoxo ligand changes coordination to an η^1 or η^3 configuration before the arene exchange reaction, we have performed three potential energy surface scans of **I**, by stretching the bonds between Ru and the 1-, 2- and 6-carbons of the phenoxo ligand, respectively, to drive the phenoxo ligand coordination towards lower hapticity. However, no local minimum was found after scanning the Ru-C bonds until a bond length of 4.5 Å, at which point the energy is already 65.9, 34.5 and 72.0 kcal/mol above that of the reactant, respectively, for the three potential energy surface scans. The structures at the end of the potential energy surface scans are given in the list of computed atomic coordinates (Fig S134–S136). The fact that the second scan yields a slightly lower energy is due to the spontaneous coordination of the $^{-}$OMs anion to the Ru center upon stretching the Ru–C bond. Nevertheless, even the energy rise of this scan is well above the barrier of **TS I** (which has an activation Gibbs free energy of 27.1 kcal/mol, and a ΔΔG = of 27.2 kcal/mol, starting from **I**). We therefore think that the intermediacy of η^1 or η^3-coordinated phenoxo ligands is unlikely.

These results can be rationalized by the fact that the phenoxo ligand coordinates more strongly than benzene (L_2X-type ligand vs. L_3-type ligand), so it may be lower in energy for benzene to change to an η^2 configuration than for the phenoxo ligand to isomerize to an η^1 configuration, and there is no reason for the phenoxo ligand to isomerize sooner than the benzene ligand does. However, once the benzene ligand coordination changes to an η^2 hapticity, the Ru center would

already have easily accessible coordination sites such that the phenoxo ligand no longer has to isomerize for the ⁻OMs to coordinate and the arene exchange reaction to proceed.

Non-covalent interactions of 1c, 1i, 1j, 1k, and SI-5

To see whether the chlorine substituents in **I** contribute to the interaction between **1g** and ⁻OMs, we have calculated the non-covalent complex of **1c** and ⁻OMs (**XI**), as well as the following transition state (**TS VII**) for the arene exchange process. The IGMH plot of **XI** is shown in Fig S112, left, which shows similar non-covalent interactions between **1c** and ⁻OMs as observed in **I**. Furthermore, the binding Gibbs free energy of **1c** and ⁻OMs (2.4 kcal/mol) is very similar to that of **1g** (2.1 kcal/mol). However, the subsequent transition state (**TS VII**), where the benzene ligand coordination changes from η^6 to η^2 with concomitant coordination of ⁻OMs to the Ru center, does not show any noticeable weak interaction between the benzene hydrogens and the phenoxo ligand (Fig S112, right), unlike in **TS I** (Scheme 2C, main text). The missing interaction in case of **1c** is one of the reasons (although not necessarily the main reason; see discussions of Table S37 later) that the ΔΔG upon going from **XI** to **TS VII** is higher (29.2 kcal/mol) than the corresponding barrier for **1g** (27.1 kcal/mol). The higher ΔΔG leads to a notably higher total free energy barrier of **1c** (31.6 kcal/mol) than **1g** (29.2 kcal/mol), in accord with the experimental observation that the arene exchange reaction of **1c** is appreciably slower than **1g**.

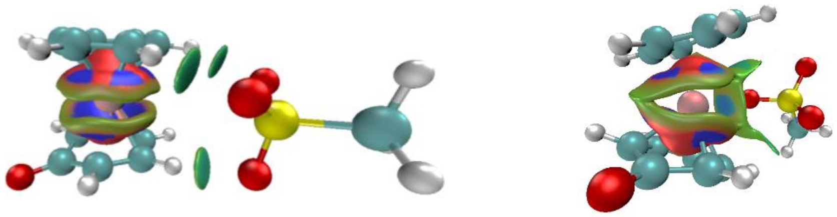

Fig S112: Left: IGMH plot (isovalue=0.01) of **XI**. Right: IGMH plot (isovalue=0.01) of TS VII.

To elucidate the effects of other substituents involved in the study, we have also performed IGMH plot and ΔΔG calculations for the 2,6-dimethoxy (**SI-5**), 4-methoxy (**1i**), 4-dimethylamino (**1l**), 4-CF_3 (**1j**) and 2,6-Br (**1k**) substituted catalysts.

From Table S1, one can see that the 2,6-dimethoxy substituted catalyst (**SI-5**) performs worse than the unsubstituted complex (**1c**), despite that catalysts with *para*-substituted electron donating groups (e.g. **1i**, **1l**), as well as *ortho*-substituted lone-pair containing groups (e.g. **1g**, **1k**), display higher arene exchange rates than **1c**. Our ΔΔG calculations suggest that the formation of the non-covalent complex **XII** from the binding of **SI-5** and ⁻OMs is even more favored (+1.6 kcal/mol) compared to **1g** (+2.1 kcal/mol). However, the following attack of ⁻OMs onto the Ru center has a much ΔΔG (31.6 kcal/mol) than the corresponding reaction of **1g** (27.1 kcal/mol), leading to a high total free energy barrier (33.3 kcal/mol) for the two steps.

The IGMH plots of **XII** as well as the transition state **TS IX** are given below. While the IGMH plot of **XII** is very similar with those of **I** (Scheme 2B) and **XI** (Fig S112), the IGMH plot of **TS IX** shows three

distinctive weakly attractive interactions: a very weak interaction between one of the benzene hydrogen atoms and one of the methoxy hydrogens (labeled A in Fig S113); an interaction between an ⁻OMs oxygen atom and a phenoxo hydrogen atom (labeled B); and an interaction between the same ⁻OMs oxygen atom with one of the hydrogen atoms of the methoxy group (labeled C). In particular, there are no IGMH isosurfaces that correspond to a C–H…O interaction between the benzene hydrogen atoms and the methoxy oxygen atoms, as opposed to the case of **1g**. We believe that the lack of the C–H…O interaction is due to the fact that the H…O vector does not lie in the C–O–C plane of the methoxy group in **TS IX**, but is instead roughly perpendicular to it. The new IGMH isosurface patches (A, B, C) do not necessarily provide sufficient stabilization to counteract the absence of benzene-methoxy C–H…O interaction, either. The non-covalent interaction between the ⁻OMs oxygen atoms and the phenoxide hydrogen atoms, which give the isosurface patch B in Fig S113, are in fact also present in **TS I** (Scheme 2C) and **TS VII** (Fig S112), except that the latter are so large that they are connected to the "main" isosurface patch that surrounds the Ru atom. The exact amount of energy stabilization provided by the additional C–H…H–C dispersion interaction (patch A) and the ⁻OMs-methoxy C–H…O interaction (patch C) cannot be determined from the IGMH plots. We will return to this issue later in the following subsections on natural bonding orbital (NBO) and local energy decomposition (LED) calculations.

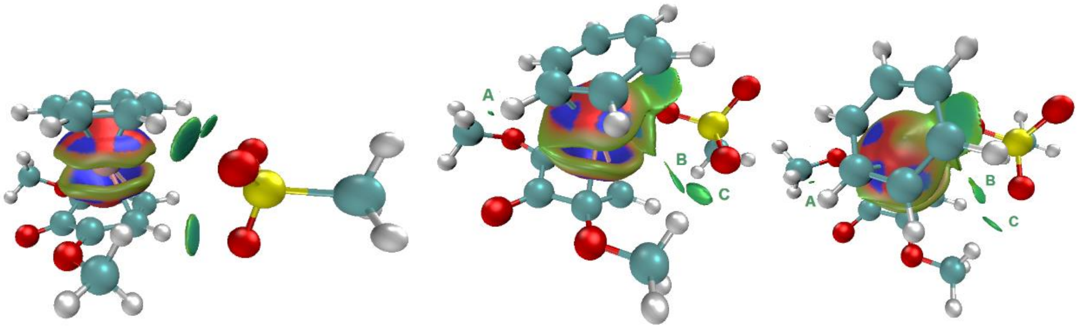

Fig S113: Left: IGMH plot (isovalue=0.01) of **XII**. Middle: IGMH plot of **TS IX** (side view). Right: IGMH plot (isovalue=0.01) of **TS IX** (top view).

The IGMH plots of the TS corresponding to the 4-methoxy substituted catalyst (**1i**), **TS X** (Fig S114), show a weak C–H…O interaction between the methyl group of ⁻OMs and the 4-methoxy group (labeled A). Furthermore, one of the ⁻OMs oxygen atoms participate in a C–H…O interaction with one of the phenoxo hydrogens (labeled B), and another oxygen atom engage in dispersion interaction with the methoxy oxygen atom (labeled C). While the interaction B is generally present in the other (**1g** or **1c**) substituted catalysts as well, the interactions A and C (which are not present in the reactant **XIII**

and involve the methoxy substituent) are expected to contribute to the lower ΔΔG for arene exchange of **1i**.

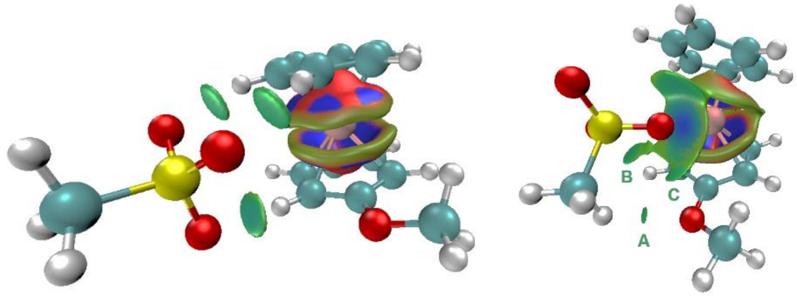

Fig S114: Left: IGMH plot (isovalue=0.01) of **XIII** (note that a different viewing angle than Figs S111–S113 was chosen, to show the absence of isosurfaces corresponding to $^-$OMs…methoxy interactions). Right: IGMH plot (isovalue=0.01) of **TS X**.

The other strongly electron donating catalyst, the NMe_2-substituted complex **1l**, similarly shows enhanced arene exchange rates compared to the unsubstituted complex **1c** (Scheme 2A). IGMH results show that the corresponding transition state (**TS XI**) is indeed characterized by a significant weak interaction between the NMe_2 group and the $^-$OMs anion; however, different from **TS X**, in **TS XI** the interaction is not a C–H…N interaction between the $^-$OMs methyl group and the NMe_2 group, but rather a C–H…O interaction between one of the C−H bonds of the NMe_2 group and one of the $^-$OMs oxygen atoms (Fig S115). Similar to the case of **1i**, the interaction is not present in the reactant complex **XIV**, and is therefore expected to contribute to a decrease of the ΔΔG of the arene exchange process.

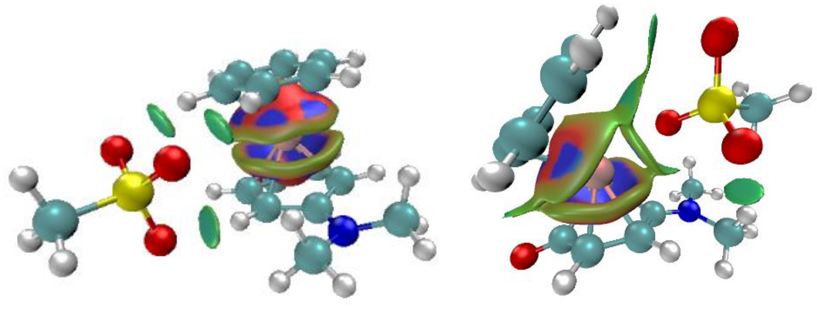

Fig S115: Left: IGMH plot (isovalue=0.01) of **XIV** (note that a different viewing angle than Figs S111–S113 was chosen, to show the absence of isosurfaces corresponding to $^-$OMs…NMe_2 interactions). Right: IGMH plot (isovalue=0.01) of **TS XI**; the C–H…O interaction (green patch of isosurface) can be seen on the lower right.

Furthermore, we also studied catalyst **1j**, with an electron withdrawing 4-CF_3 substituent on the phenoxo ligand (Fig S116). Different from **TS X** and **TS XI**, IGMH plot of the TS of **1j**, **TS XII**, shows no observable C–H…F interactions involving the CF_3 group (despite that the fluorine atoms possess lone pair electrons), nor any other kinds of interactions that involve the CF_3 group. This suggests that

the rate enhancement of **1j** compared to **1c** (Scheme 2A) is probably primarily due to stronger cation-anion non-covalent binding in the reactant complex **XV** (as discussed in the main text), and not due to non-covalent stabilization effects that arise in the transition state.

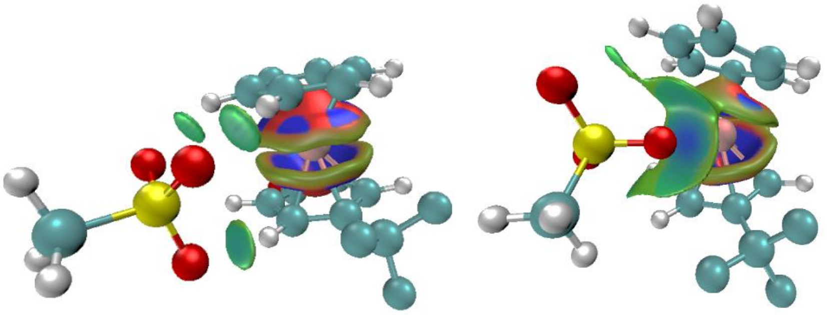

Fig S116: Left: IGMH plot (isovalue=0.01) of **XV** (note that a different viewing angle than Figs S111–S113 was chosen, to show the absence of isosurfaces corresponding to $^{-}$OMs…CF_3 interactions). Right: IGMH plot (isovalue=0.01) of TS XII.

Eventually, to verify that bromine substituents can engage in C–H…Br interactions in the transition state, similar to the chlorine substituents of **1g**, we have also made IGMH plots of the reactant complex (**XVI**) and TS (**TS XIII**) of **1k**. In **TS XIII**, a green isosurface is present between one of the C–H bonds and one of the bromine atoms (Fig S117, right; the isosurface patch is visible on the left); furthermore, the interaction is not present in the reactant complex **XVI** (Fig S117, left). Therefore, the C–H…Br interaction in **TS XIII** is expected to lower the ΔΔG similar to **TS I**.

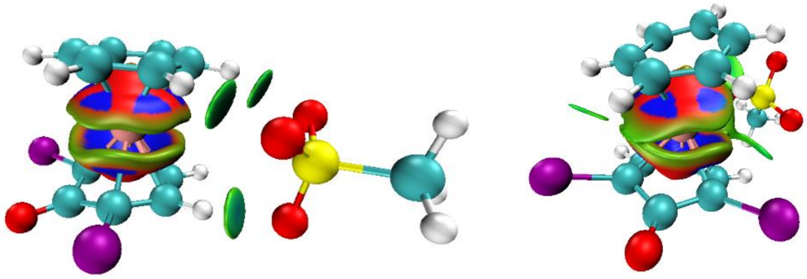

Fig S117: Left: IGMH plot (isovalue=0.01) of **XVI**. Right: IGMH plot (isovalue=0.01) of **TS XIII**.

Steric repulsion, dispersion, electrostatic, and charge transfer energies

While the IGMH plot in Scheme 2B indicates a weak attractive interaction between one of the benzene hydrogen atoms and one of the chlorine atoms in **TS I**, The IGMH plots do not provide much information on the origin of the C–H…X interaction. Therefore, herein we estimate the sterical, dispersion, electrostatic and charge transfer contributions to the C–H…X interactions in **1g**, **SI-5**, **1i** and **1l**, as well as the corresponding contributions in the unsubstituted catalyst **1c**. The interactions can be classified into three categories: (1) the C–H bond comes from the benzene ligand, and the acceptor X is an *ortho* substituent of the phenoxo ligand (**1g**, **1k**); (2) the C–H bond comes from the $^{-}$OMs anion, and the acceptor X is a *para* substituent of the phenoxo ligand (**1i**); and (3) the C–H

bond comes from the substituent, and the acceptor X is the ⁻OMs anion (**1l**, **SI-5**).

Natural bonding orbital (NBO) analyses,[42] including second-order perturbative estimates of NBO interactions and natural steric analysis,[43-45] were performed by the NBO 7.0 package[46] via its interface with ORCA, under the level of theory used for geometry optimization. Local energy decomposition (LED) analyses[47-50] were conducted under the level of theory used for single point energy calculations.

Sterical contributions: The steric repulsions of the C–H bond and the acceptor X are obtained by natural sterical analysis, which gives the sterical interactions as a list over pairs of NBOs. The steric repulsions between the C–H bonding NBO and all bonding NBOs that belong to the acceptor X (including the C(phenoxo)–X bonding NBO, if any) are summed.

However, the NBO approach to determine the sterical contributios fails to account for geometry preparation contributions. In the example of **SI-5** the methoxy group of **SI-5** that will eventually form the IGMH isosurface patch "A" in Fig S113 rotates away from the benzene ligand when going from **XII** to **TS IX**, with the C(carbonyl)–C–O–Me dihedral angle changing from 40.9º to 24.1º. Clearly, the methoxy group is influenced by the steric repulsion from the tilted benzene ligand in **TS IX**, and to reduce the steric repulsion energy, it rotates to a dihedral angle that would not be optimal if this steric repulsion were not present, leading to an energy increase. The energy that would be obtained by analyzing the structure of **TS IX** itself cannot capture this secondary energy increase, and would underestimate the total influence of steric repulsion. The geometry preparation energy can thus be defined as the energy increase during rotating the methoxy group from 40.9º to 24.1º in **XII**, but while keeping all other atoms frozen; as the geometry preparation energy is mostly a result of steric repulsion, it adds to the total sterical contribution.

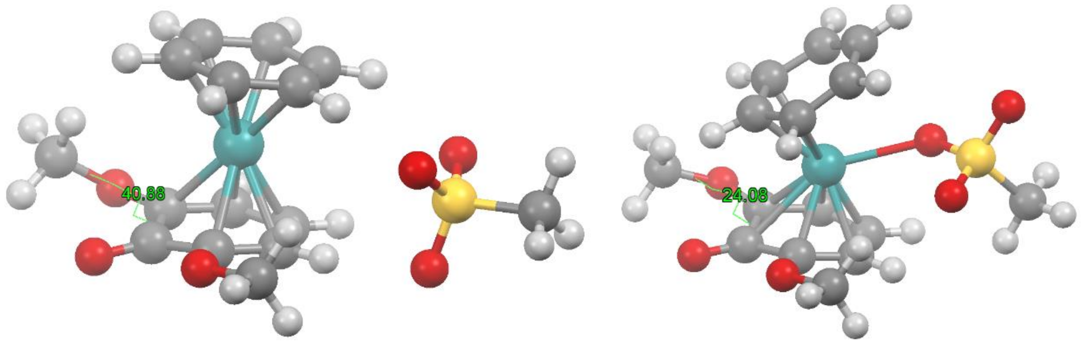

Fig S118: Structures of **XII** (left) and **TS IX** (right), highlighting the C(carbonyl)–C–O–Me dihedral angle change due to the steric repulsion of the benzene ligand.

Dispersion contributions: Dispersion energies between the C–H bond and acceptor X on the phenoxo ligand are obtained by LED analysis. The dispersion energies are given as a list over localized molecular orbital (LMO) pairs. The dispersion interactions between the C–H bonding LMO and all bonding LMOs that belong to the acceptor X (including the C(phenoxo)–X bonding NBO, if any) are summed.

Electrostatic contributions: While it is possible to calculate how much electrostatic interaction

energy a pair of orbitals contributes, the value is not very useful since the electrostatic interaction energy between two orbitals is always repulsive and large, and will be largely offset by the effects of the surrounding atomic nuclei anyway. Therefore, for the electrostatic term we performed a partition over atoms instead of orbitals. We calculated the electrostatic energy between the C–H bond and acceptor X from the Hirshfeld I charges of the H atom, as well as all the constituent atoms of the X group, using an undamped Coulomb energy formula with a dielectric constant of 1 (since the atoms in question are in direct touch with each other, we expect the solvent screening effects on the Coulomb interactions are small).

Charge transfer contributions: Charge transfer contributions to the C–H…X interaction energies are obtained by second-order perturbation theory analysis (E2PERT) under the NBO basis. The perturbatively estimated interaction energies due to electron donation from the NBOs of the substituent X to the antibonding NBO of the C–H bond are summed.

The computed contributions for **1c**, **1g** and **SI-5** due to C–H(benzene)…X(*ortho* substituent) interactions are listed in Table S37. For the unsubstituted catalyst, the non-covalent interaction between the *ortho*-hydrogen and the benzene hydrogen is overall repulsive, with the major contributors being steric repulsion and Coulomb repulsion. Though substitutions at the *ortho* positions generally increase the steric hindrance, therefore making a positive contribution to the ΔΔG of the arene exchange process, the barrier can still be lowered if the substituents can engage in favorable dispersion, electrostatic and charge transfer interactions with the benzene hydrogen atoms. This is indeed the case for **1g**: since the chlorine atom in **1g** carry much less positive charge than the corresponding hydrogen atom in **1c** (Hirshfeld I charges: +0.026 and +0.197, respectively, in the reactant complexes), **1g** has a lower ΔΔG for the arene exchange process than **1c,** due to less electrostatic repulsion, although the C–H…Cl electrostatic energy is still positive. Overall, the C–H…Cl electrostatic, dispersion and charge transfer contributions in **1g** yield 0.7, 0.1 and 0.2 kcal/mol of ΔΔG decrease, respectively, which more than compensates for the larger steric hindrance (0.8 kcal/mol) of the Cl atom versus the H atom. Therefore, although electrostatic contributions are not the single largest contributor to the C–H…Cl interaction energy, they are the main contributor to the reduction of the reaction barrier due to the C–H…Cl interaction. Although the electrostatic contribution of the C–H…Cl interaction to the reduction of the barrier (0.7 kcal/mol) is much smaller than the free energy barrier difference between the reactions **1c** + $^{-}$OMs (31.6 kcal/mol) and **1g** + $^{-}$OMs (29.2 kcal/mol), the remaining barrier reduction can be explained by the arene exchange reaction being accelerated by electron-deficient ligands, as mentioned in the main text.

By comparison, **SI-5** provides a slightly larger electrostatic and dispersive stabilization of the TS (which should include the contribution of isosurface patch A in Fig S113), but also a much larger steric repulsion contribution to the barrier (2.5 kcal/mol larger than in **1c**). The charge transfer contribution to the C–H…O interaction is negligible, in accord with the fact that the hydrogen atom is not lying in the C–O–C plane of the methoxy group. Overall, C–H(benzene)…OMe non-covalent interactions contribute +2.7 kcal/mol to the reaction barrier, where the corresponding C–H(benzene)…H interaction in **1c** contributes only +1.5 kcal/mol. This is in accord with the higher ΔΔG of **SI-5**

compared to **1c**.

Table S37 Steric repulsion (including geometry preparation), dispersion, electrostatic and, charge transfer contributions to the reactant complexes and transition states of **1c**, **1g** and **SI-5**. The tabulated energies refer to the interaction between one of the benzene C–H bonds (the closest to the substituent) and the substituent. For substituents that possess rotatable dihedral angles, geometry preparation contributions to the steric energies are listed in brackets.

Substituent(s)	Species	Steric (kcal/mol)	Dispersion (kcal/mol)	Electrostatic (kcal/mol)	Charge transfer (kcal/mol)
2,6-H	Reactant (**1c**)	0.0	0.0	3.6	0.0
	TS (**TS VII**)	0.6	−0.1	4.6	0.0
	Difference	0.6	−0.1	1.0	0.0
2,6-Cl	Reactant (**1g**)	0.1	−0.1	0.4	0.0
	TS (**TS I**)	1.5	−0.3	0.7	−0.2
	Difference	1.4	−0.2	0.3	−0.2
2,6-OMe	Reactant (**SI-5**)	0.0	−0.2	−0.9	0.0
	TS (**TS I**)	3.1 [1.2]	−0.5	−1.0	0.0
	Difference	3.1	−0.3	−0.1	0.0

The computed contributions for **1c** and **1i** due to C–H($^{-}$OMs)…X(*para* substituent) interactions are listed in Table S38. The Coulomb interaction between the positively charged $^{-}$OMs methyl hydrogen atom and the *para* hydrogen atom of the phenoxo ligand contributes +1.9 kcal/mol to the reaction barrier of **1c**. By contrast, substituting the *para* hydrogen in **1c** by the OMe group changes the electrostatic interaction from positive to negative, due to the oxygen atom carrying negative charge. Furthermore, dispersion and charge transfer in **TS X** also provide extra stabilization for the TS. Although steric repulsion leads to a positive contribution to the barrier, its magnitude is small (0.8 kcal/mol), and in particular, is basically free from geometry preparation energy contributions. Overall, the C–H…OMe interaction in **TS X** contributes −0.8 kcal/mol to the barrier, which is 2.7 kcal/mol lower than the corresponding contribution in the unsubstituted catalyst **1c**. This explains why **1i** has a faster rate of arene exchange than **1c** (Scheme 2A), despite our Hirshfeld I charge analyses suggest that electron donating substituents should in principle give slower arene exchange rates (see main text).

Table S38 Steric repulsion (including geometry preparation), dispersion, electrostatic, and charge transfer contributions to the reactant complexes and transition states of **1c** and **1i**. The tabulated energies refer to the interaction between one of the $^{-}$OMs C–H bonds (the closest to the substituent) and the substituent. For substituents that possess rotatable dihedral angles, geometry preparation contributions to the steric energies are listed in brackets.

Substituent(s)	Species	Steric (kcal/mol)	Dispersion (kcal/mol)	Electrostatic (kcal/mol)	Charge transfer (kcal/mol)
4-H	Reactant (**1c**)	0.0	0.0	1.9	0.0

	TS (TS VII)	0.0	0.0	3.8	0.0
	Difference	0.0	0.0	1.9	0.0
4-OMe	Reactant (**1i**)	0.0	0.0	−0.5	0.0
	TS (TS X)	0.8 [0.0]	−0.5	−1.4	−0.2
	Difference	0.8	−0.5	−0.9	−0.2

The computed contributions for **SI-5** and **1l** due to C–H(substituent)…X(⁻OMs) interactions are listed in Table S39. Since C–H(substituent)…X(⁻OMs) interactions are not present in **1c**, the corresponding contributions in **1c** are by definition all zero and thus not listed. The C–H(OMe)…X(⁻OMs) interaction in **SI-5** gives a contribution of −0.5 kcal/mol to the barrier, which is consistent with the attractive interaction shown in the isosurface patch C in Fig S113; however this attractive interaction is not large enough to compensate for the +1.2 kcal/mol repulsive contribution to the barrier due to the C–H(benzene)… OMe repulsion, compared to the case of **1c** (Table S37). Therefore, the net contribution of non-covalent interactions to the arene exchange barrier of **SI-5** is more positive compared to **1c**, consistent with the experimental finding that **SI-5** has a lower arene exchange rate than **1c** (Table S1).

By contrast, the C–H(NMe_2)… ⁻OMs interaction in **TS XI**, as already shown in the IGMH plots in Fig S115, provides a much larger ΔΔG reduction (−4.9 kcal/mol). The major contributor to the ΔΔG decrease is the electrostatic term, due to the large negative charge of the ⁻OMs oxygen atom participating in the C–H…O interaction (−0.930) and the positive charge of the H atom (+0.147), followed by charge transfer and dispersion contributions. The steric repulsion between the C–H bond and the ⁻OMs anion is considerable (4.4 kcal/mol), but much smaller than the sum of the attractive contributions. Thus, even though electron donating groups are predicted to slow down the arene exchange (see main text), **1l** has a higher arene exchange rate than **1c**, due to the very favorable non-covalent interactions that lower the ΔΔG for the arene exchange process.

Table S39 Steric repulsion (including geometry preparation), dispersion, electrostatic, and charge transfer contributions to the reactant complexes and transition states of **SI-5** and **1l**. The tabulated energies refer to the interaction between one of the substituent's C–H bonds (the closest to the ⁻OMs anion) and the ⁻OMs anion. Geometry preparation contributions to the steric energies are listed in brackets.

Substituent(s)	Species	Steric (kcal/mol)	Dispersion (kcal/mol)	Electrostatic (kcal/mol)	Charge transfer (kcal/mol)
2,6-OMe	Reactant (**SI-5**)	0.6	−0.4	−11.3	−0.4
	TS (TS VII)	1.5	−0.5	−12.0	−1.0
	Difference	0.9	−0.1	−0.7	−0.6
4-NMe_2	Reactant (**1l**)	0.0	0.0	−6.7	0.0
	TS (TS XI)	4.4 [1.1]	−1.1	−13.0	−1.9
	Difference	4.4	−1.1	−6.3	−1.9

Other conformers of intermediate I

The conformer of **I** reported in Scheme 2B has two C–H…O interactions between the benzene ligand and the $^{-}$OMs anion, and one C–H…O interaction between the phenoxo ligand and the $^{-}$OMs anion. However, this is not the only thermally accessible conformer; our DOCKER run at the GFN2-xTB level of theory yielded 13 conformers, of which 11 inequivalent conformers remain after DFT geometry optimization and removal of imaginary frequencies (Fig. S119). The Gibbs free energies of all conformers are within 4.0 kcal/mol of the lowest conformer (**I-conf0**), while 5 of them are within 2.0 kcal/mol of the lowest conformer.

The 11 conformers can be categorized into 3 types: (1) those with three C–H…O interactions between arene C–H bonds and the $^{-}$OMs anion (**I-conf0**, **I**, **I-conf2**); (2) those with phenoxo…O arene-π interactions (**I-conf3** - **I-conf9**); and (3) a single conformer with one arene C–H…OMs interaction and one CH_3(OMs)…O(phenoxo) interaction (**I-conf10**). The (1)-type conformers are the most important and dominate the whole Boltzmann population. Although the conformer **I-conf0** possessing one benzene–OMs C–H…O interaction and two phenoxo–OMs C–H…O interactions has the lowest Gibbs free energy, the other two conformers **I** and **I-conf2** both have two benzene–OMs C–H…O interaction and one phenoxo–OMs C–H…O interaction, and their summed Boltzmann weight is higher than that of **I-conf0**. (The conformers **I** and **I-conf2** differ slightly in the dihedral rotation angle of the two arene ligands, and this difference does not vanish even with tight optimization criteria, i.e. using the !TightOpt keyword in ORCA. This suggests that they are distinct conformers). Therefore, within the current computational error bar, we cannot determine whether it is more likely for the $^{-}$OMs anion to form one or two C–H…O interactions with the benzene ligand. We thus choose **I**, rather than **I-conf0**, as the “representative” conformer on which most discussions are based. Given that **I-conf0** also has three C–H…O interactions between the arene ligands and the $^{-}$OMs anion, and that **I-conf0** and **I** have very similar Gibbs free energies, our conclusions about **I** should remain largely unchanged if we based our discussions on **I-conf0** instead.

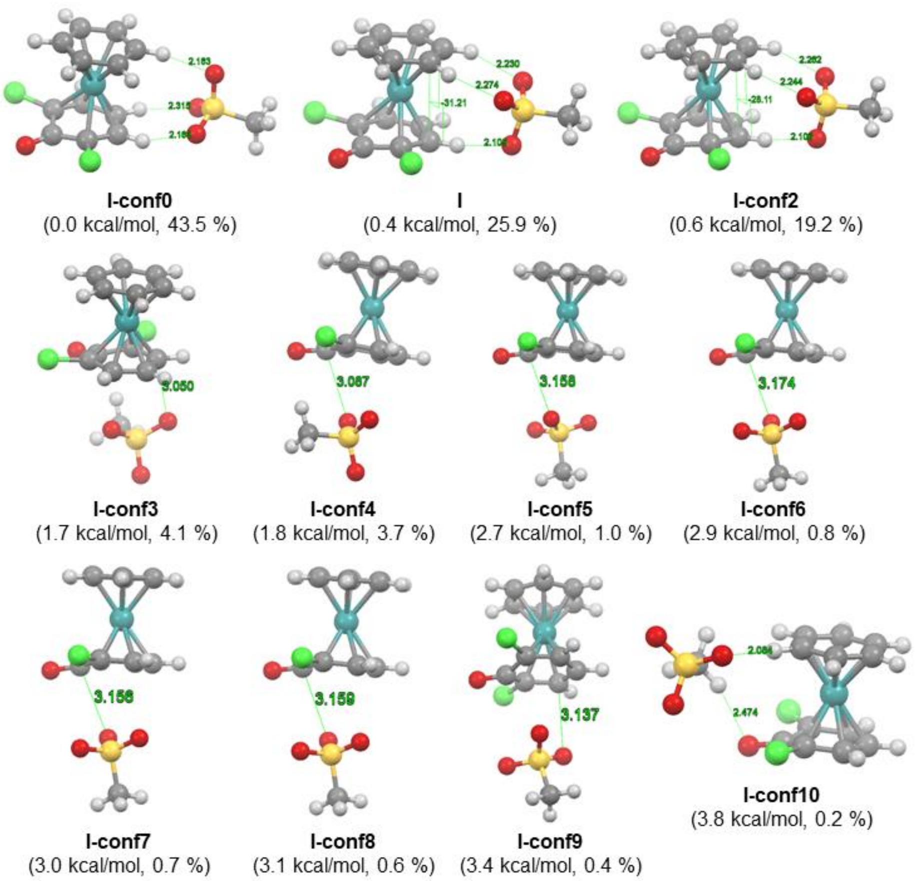

Fig S119: All conformers located for **I**. Relative Gibbs free energies and Boltzmann ratios at 90 °C, as well as key distances (Å) and dihedral angles (°), are shown.

Geometry optimization for additional computational results of the arene exchange reaction

Geometry of 1c

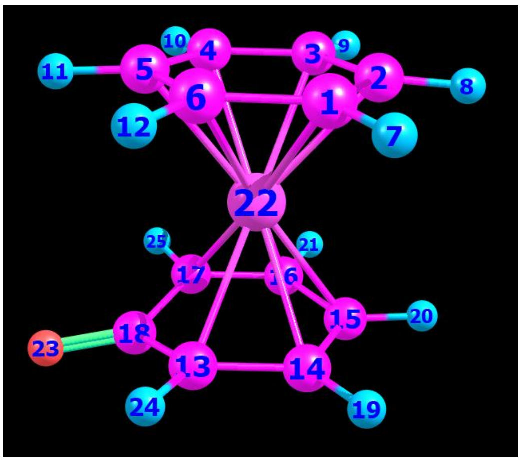

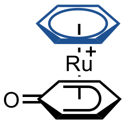

1c

Fig S120: Structure of **1c**. Left: With numbering of atoms respective to table S40.

Table S40 XYZ-coordinates for **1c**:

Atom	X-Coordinate	Y-Coordinate	Z-Coordinate

C1	2.017562555	11.51113239	-1.377286938
C2	3.324313668	12.04744468	-1.369907407
C3	3.51610908	13.43541455	-1.193550891
C4	2.400447134	14.2862669	-1.06285607
C5	1.090515654	13.75633223	-1.084592634
C6	0.904921467	12.36645236	-1.232784978
H8	1.866582146	10.45565751	-1.561120675
H9	4.174998104	11.4010074	-1.541357852
H10	4.512547076	13.85494084	-1.236584865
H11	2.545310805	15.35767296	-1.013898565
H12	0.23581464	14.41801382	-1.043014684
H13	-0.096277836	11.9635271	-1.313868622
C14	0.748462134	12.73175321	-4.591166593
C15	1.916520155	11.96838379	-4.770104963
C16	3.180772773	12.60002387	-4.771694515
C17	3.243563558	13.99915666	-4.580468905
C18	2.060452952	14.73988326	-4.405424072
C19	0.740289499	14.18808586	-4.695200422
H20	1.856529333	10.88759575	-4.807751667
H21	4.087444837	12.01366771	-4.836979257
H22	4.206638883	14.48278596	-4.472249346
Ru23	2.149911704	13.0999488	-2.90797445
O24	-0.278884762	14.86451269	-4.814401854
H25	-0.205721522	12.22605323	-4.50194169
H26	2.127049418	15.79588396	-4.171721735

Geometry of XI

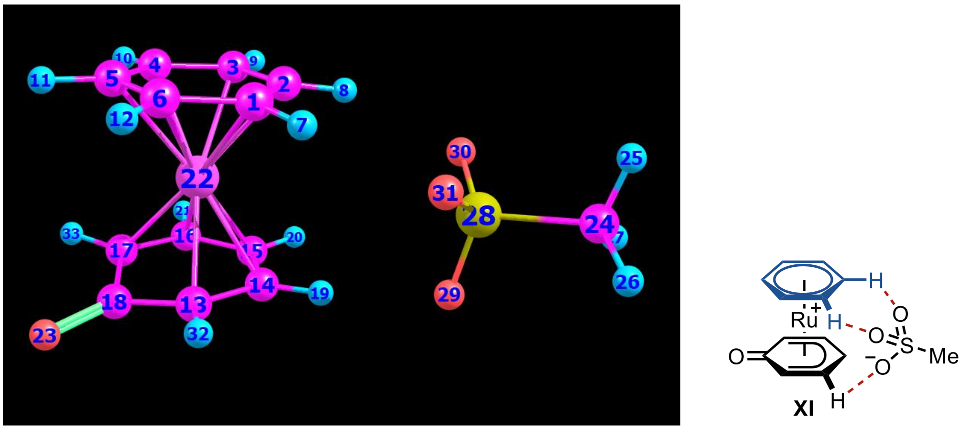

Fig S121: Structure of **XI**. Left: With numbering of atoms respective to table S41.

Table S41 XYZ-coordinates for **XI**:

Atom	X-Coordinate	Y-Coordinate	Z-Coordinate
C1	1.732483544	11.58905836	-1.383087515
C2	3.121247358	11.83582669	-1.375872236
C3	3.59947086	13.15396423	-1.22190316
C4	2.687098966	14.21982452	-1.080333364
C5	1.293398406	13.96825077	-1.052087304
C6	0.817756913	12.65452824	-1.201920137
H7	1.381735633	10.58510445	-1.593509689
H8	3.798659986	11.01450566	-1.57936874
H9	4.660030329	13.35890128	-1.288967139
H10	3.0497623	15.23855543	-1.041839427
H11	0.597109921	14.79420186	-0.990449266
H12	-0.246803232	12.46841161	-1.258058109
C13	0.904611471	12.49409489	-4.631984101
C14	2.206488676	11.98215264	-4.782734404
C15	3.314328874	12.85909482	-4.741946551
C16	3.088813387	14.23969469	-4.539540275
C17	1.77623449	14.72405303	-4.393939059
C18	0.603283352	13.91903126	-4.719137431
H19	2.370731304	10.90879917	-4.819200628
H20	4.322500373	12.46858011	-4.781862224

H21	3.930595803	14.90677358	-4.399141394
Ru22	2.15531507	13.11693987	-2.905060692
O23	-0.530657839	14.37786881	-4.852686091
C24	3.289787785	6.634157248	-3.270552414
H25	3.404236751	6.288079763	-2.243882164
H26	2.454919716	6.12449407	-3.750266439
H27	4.20849173	6.464928194	-3.831081778
S28	2.95357501	8.372454937	-3.246598452
O29	2.809541957	8.776881634	-4.64688859
O30	4.105156889	8.999766889	-2.595824917
O31	1.714653447	8.536002545	-2.483842886
H32	0.070595413	11.80452481	-4.57149309
H33	1.621082098	15.76885672	-4.151781622

Geometry of TS VII

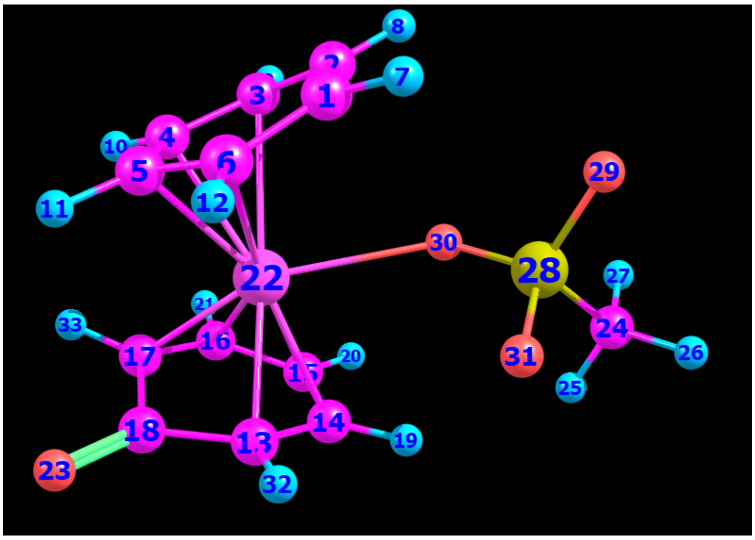

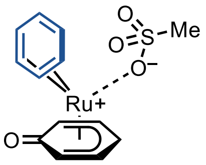

TS VII

Fig S122: Structure of **TS VII**. Left: With numbering of atoms respective to table S42.

Table S42 XYZ-coordinates for **TS VII**:

Atom	X-Coordinate	Y-Coordinate	Z-Coordinate
C1	3.198207858	11.46480024	-0.922699107
C2	4.116067688	12.43772095	-0.593197674
C3	3.814037786	13.78291674	-0.856977534
C4	2.518759883	14.16110087	-1.234878626

C5	1.556589642	13.16896513	-1.518011437
C6	1.953285681	11.8250155	-1.477457689
H7	3.451710497	10.41771704	-0.822334093
H8	5.094879739	12.16663919	-0.222053692
H9	4.559539436	14.55083877	-0.690442936
H10	2.253880015	15.20835438	-1.300972958
H11	0.543056332	13.44107152	-1.781935345
H12	1.250740803	11.05504512	-1.770731694
C13	2.051534802	12.41843251	-5.023130188
C14	3.402476516	12.69044251	-5.309817902
C15	3.955144764	13.95243696	-5.006220902
C16	3.128703476	14.92725519	-4.40955018
C17	1.763181705	14.6321631	-4.169523115
C18	1.085234287	13.48012598	-4.75536706
H19	4.045347095	11.88455767	-5.632980763
H20	5.016597478	14.12761046	-5.12018268
H21	3.554959822	15.85836358	-4.055554131
Ru22	3.000122142	13.16437853	-3.216017596
O23	-0.134885914	13.33047253	-4.774273243
C24	6.650284475	10.35206798	-4.591923546
H25	6.364039089	10.91609756	-5.479035831
H26	6.859514773	9.315323163	-4.853520824
H27	7.519800916	10.80651826	-4.118773602
S28	5.306230136	10.37509036	-3.442254136
O29	5.747810747	9.619057575	-2.27676958
O30	5.094743815	11.80528263	-3.130603765
O31	4.160439311	9.78196201	-4.122016678
H32	1.678034245	11.40948327	-5.153939914
H33	1.154346801	15.35517392	-3.638458431

Geometry of X

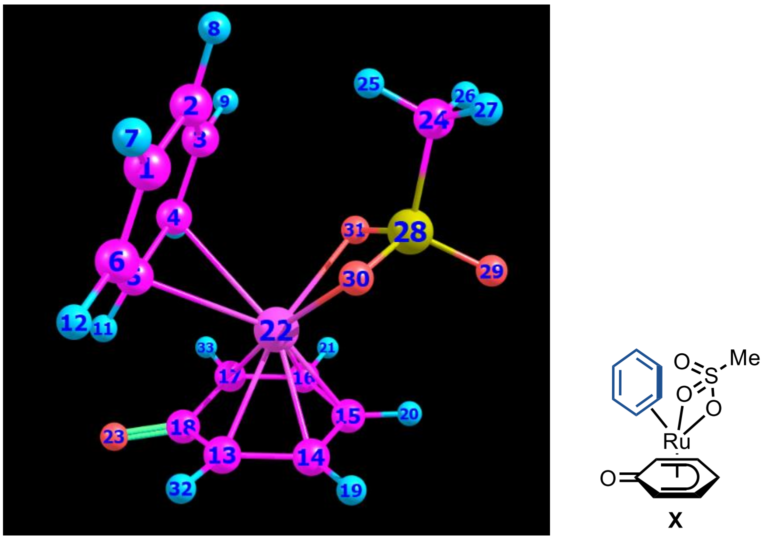

X

Fig S123: Structure of **X**. Left: With numbering of atoms respective to table S43.

Table S43 XYZ-coordinates for **X**:

Atom	X-Coordinate	Y-Coordinate	Z-Coordinate
C1	3.156517085	11.10833363	-0.541533554
C2	4.319391889	11.86857954	-0.37242578
C3	4.405219424	13.13690107	-0.900381762
C4	3.312954789	13.68778542	-1.587985104
C5	2.134571683	12.94230189	-1.725073744
C6	2.07544949	11.63633193	-1.208709829
H7	3.104442797	10.10687183	-0.130064214
H8	5.154891499	11.45344205	0.180110281
H9	5.307252399	13.72344467	-0.774081856
H10	3.333243111	14.73191143	-1.877444968
H11	1.225688715	13.41880512	-2.067639529
H12	1.161292737	11.06402828	-1.314890287
C13	1.122157758	12.52295948	-4.67593175
C14	2.06747976	12.06772879	-5.620580348
C15	3.053252823	12.94656875	-6.125469293
C16	3.094602401	14.26468446	-5.60258076
C17	2.134404661	14.69443042	-4.663910875
C18	0.919038593	13.93853987	-4.399201991

H19	2.110858481	11.01533261	-5.876485326
H20	3.825644111	12.58975855	-6.793626171
H21	3.934895239	14.90544982	-5.843845124
Ru22	3.164574673	12.89059892	-4.00224075
O23	-0.043768069	14.38281069	-3.766759702
C24	6.76009967	11.08864949	-2.797787557
H25	6.425621732	11.38235421	-1.807205166
H26	7.706714259	11.56705772	-3.04786222
H27	6.850192557	10.00570151	-2.877665334
S28	5.582769373	11.61022623	-3.985103429
O29	6.069957906	11.20041919	-5.278042051
O30	4.262556965	11.01759204	-3.620067726
O31	5.39838711	13.08000169	-3.838836393
H32	0.425833716	11.81563917	-4.240966506
H33	2.229341333	15.67981407	-4.223000533

Geometry of IX

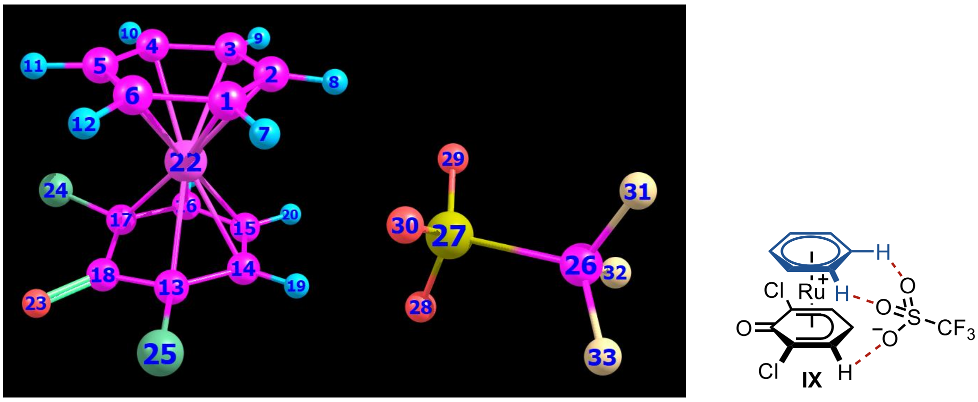

Fig S124: Structure of **IX**. Left: With numbering of atoms respective to table S44.

Table S44 XYZ-coordinates for **IX**:

Atom	X-Coordinate	Y-Coordinate	Z-Coordinate
C1	1.731223974	11.61314047	-1.363480905
C2	3.120346316	11.8592244	-1.359380014
C3	3.599078668	13.17882797	-1.221876598
C4	2.687105636	14.24685856	-1.090451483
C5	1.294655087	13.99590373	-1.060516691

C6	0.817997135	12.6808585	-1.196915333
H7	1.372201073	10.61010785	-1.559993919
H8	3.803751087	11.04037327	-1.548135366
H9	4.659614233	13.38178256	-1.294406871
H10	3.050048985	15.26615487	-1.068015231
H11	0.598490952	14.82345705	-1.016911223
H12	-0.246471654	12.49517205	-1.2599469
C13	0.918372442	12.48471992	-4.641592026
C14	2.227091928	11.98442711	-4.783776588
C15	3.319566008	12.87498886	-4.741262555
C16	3.082992082	14.25114971	-4.543648459
C17	1.760560641	14.71718247	-4.407877504
C18	0.577524116	13.91069657	-4.740328313
H19	2.394183189	10.91359362	-4.838729518
H20	4.331962092	12.49618087	-4.783827905
H21	3.910314808	14.93802244	-4.417396649
Ru22	2.155487162	13.12553224	-2.907731661
O23	-0.549001875	14.35134147	-4.877419876
Cl24	1.498346147	16.38667586	-4.110434019
Cl25	-0.391517329	11.3758026	-4.633445933
C26	3.273744534	6.602678731	-3.292346753
S27	2.947960721	8.417344288	-3.258884569
O28	2.857542745	8.7664254	-4.663465136
O29	4.106414914	8.954494565	-2.573575594
O30	1.70268429	8.521842034	-2.52532139
F31	3.383818791	6.118281607	-2.055455952
F32	4.406049508	6.335655679	-3.942969746
F33	2.28299476	5.953764145	-3.903724788

Geometry of TS VIII

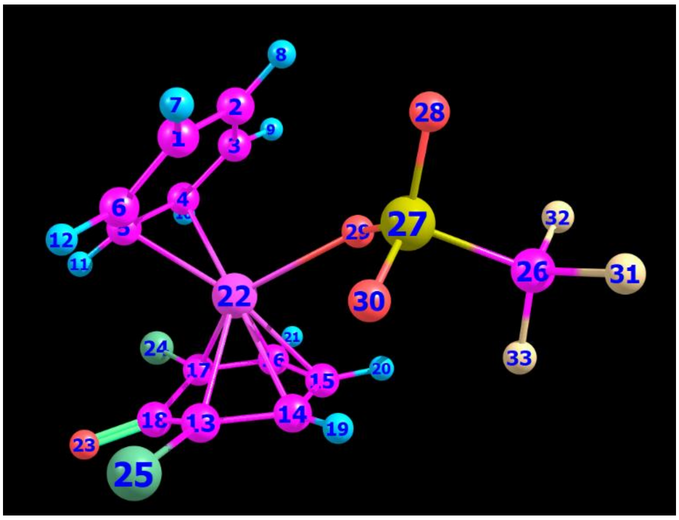

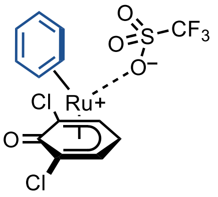

TS VIII

Fig S125: Structure of **TS VIII**. Left: With numbering of atoms respective to table S45.

Table S45 XYZ-coordinates for **TS VIII**:

Atom	X-Coordinate	Y-Coordinate	Z-Coordinate
C1	3.017606279	11.37518158	-0.963145895
C2	4.024329354	12.21791831	-0.526957313
C3	3.890331481	13.59147084	-0.719049515
C4	2.688388148	14.13324776	-1.193744805
C5	1.609649974	13.28407278	-1.51723214
C6	1.83204845	11.89799332	-1.508441153
H7	3.14573373	10.30065208	-0.915278603
H8	4.942952504	11.81093402	-0.127285538
H9	4.709115162	14.25839932	-0.477565068
H10	2.564127448	15.20700685	-1.246726654
H11	0.637910442	13.68898156	-1.768415089
H12	1.044233025	11.22839956	-1.830121547
C13	1.807693684	12.3867774	-4.941972994
C14	3.162870042	12.46827554	-5.333536446
C15	3.859871306	13.68111054	-5.16949897
C16	3.200372007	14.77551991	-4.582239193
C17	1.826391983	14.65554346	-4.242725876
C18	0.960411101	13.57083918	-4.723154207

H19	3.679398049	11.56981469	-5.642762922
H20	4.922846687	13.72903512	-5.364035566
H21	3.742073532	15.6788827	-4.330835226
Ru22	2.95457234	13.10341296	-3.273453011
O23	-0.254234472	13.57001294	-4.683934449
Cl24	1.036446914	16.01303795	-3.539406201
Cl25	1.002790284	10.8734877	-5.068632322
C26	6.825001916	10.67585034	-4.526790321
S27	5.418056289	10.49508904	-3.348281306
O28	6.000760284	9.82983974	-2.209439182
O29	5.050466172	11.89199291	-3.097192241
O30	4.431686689	9.745523545	-4.088740825
F31	7.302827133	9.481033241	-4.864933049
F32	7.81160554	11.38281811	-3.982012095
F33	6.430702125	11.29730883	-5.637638777

Geometry of XVII

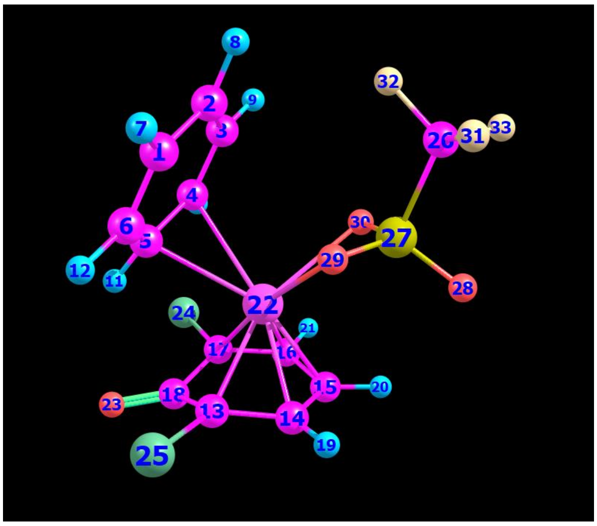

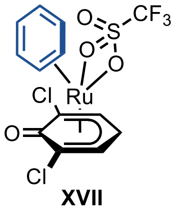

XVII

Fig S126: Structure of **XVII**. Left: With numbering of atoms respective to table S46.

Table S46 XYZ-coordinates for **XVII**:

Atom	X-Coordinate	Y-Coordinate	Z-Coordinate
C1	3.035900123	11.24940195	-0.526284129
C2	4.23737762	11.94537056	-0.369667505
C3	4.389912854	13.20255921	-0.911753972

C4	3.325395586	13.80614859	-1.595709238
C5	2.099547114	13.12845564	-1.712177172
C6	1.977301905	11.82903619	-1.189844269
H7	2.932505348	10.25417228	-0.109976749
H8	5.053962029	11.48742845	0.174269776
H9	5.324946776	13.73767419	-0.803765912
H10	3.402522641	14.84510977	-1.896951877
H11	1.209079274	13.65805163	-2.025878627
H12	1.03382637	11.30584962	-1.282813787
C13	1.219235428	12.38122801	-4.731936857
C14	2.257304809	12.06937276	-5.652218892
C15	3.15648976	13.0747554	-6.043242844
C16	3.0361497	14.35315992	-5.445268343
C17	1.959274313	14.62363883	-4.572435284
C18	0.773575738	13.75981997	-4.474465305
H19	2.416862456	11.04161808	-5.955290858
H20	4.008764937	12.83843481	-6.666521484
H21	3.809304664	15.09815606	-5.590612159
Ru22	3.105257139	12.91806138	-3.899391994
O23	-0.289392821	14.07657092	-3.979134754
Cl24	1.849675568	16.18266384	-3.849665424
Cl25	0.168225715	11.11263809	-4.231144585
C26	6.87125454	10.88504736	-2.956014064
S27	5.544149098	11.58524805	-4.036760657
O28	5.89728405	11.11082001	-5.342295924
O29	4.276422389	11.05296071	-3.491947526
O30	5.569203887	13.03214087	-3.827199379
F31	6.723595335	9.569068063	-2.87543558
F32	6.842060911	11.39772942	-1.738392088
F33	8.050891824	11.15356654	-3.500765698

Geometry of 1k

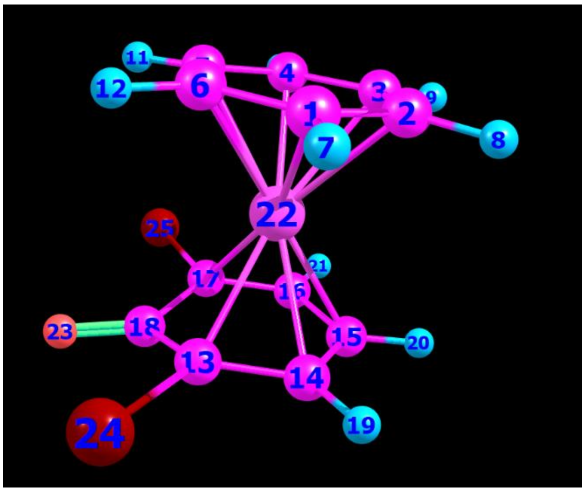

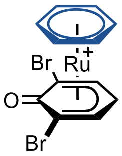

1k

Fig S127: Structure of **1k.** Left: With numbering of atoms respective to table S47.

Table S47 XYZ-coordinates for **1k**:

Atom	X-Coordinate	Y-Coordinate	Z-Coordinate
C1	1.727233936	11.58969134	-1.369180883
C2	3.11623754	11.83521651	-1.363889185
C3	3.59549951	13.15437175	-1.22251056
C4	2.683602647	14.22176854	-1.09093493
C5	1.290926638	13.97145515	-1.062011843
C6	0.813979028	12.65728309	-1.20029051
H7	1.357274966	10.59016734	-1.556953124
H8	3.808783707	11.02286421	-1.541476623
H9	4.656359596	13.35637249	-1.292249688
H10	3.047304348	15.24079008	-1.066773024
H11	0.594815439	14.79897147	-1.016727724
H12	-0.250410458	12.47099162	-1.263066767
C13	0.907680271	12.48199376	-4.640827231
C14	2.209326461	11.96367694	-4.783009852
C15	3.315058572	12.8390368	-4.744516498
C16	3.095169399	14.21833824	-4.545465058
C17	1.778642988	14.69891994	-4.407760087
C18	0.586547077	13.91214978	-4.756390524
H19	2.364975174	10.89374783	-4.838500527

H20	4.322609253	12.4476163	-4.788013036
H21	3.934533207	14.89004939	-4.417202774
Ru22	2.152608507	13.10273508	-2.911516043
O23	-0.529649161	14.36747668	-4.914386784
Br24	-0.542625302	11.28930127	-4.631880939
Br25	1.515104835	16.52877535	-4.07976354

Geometry of XVI

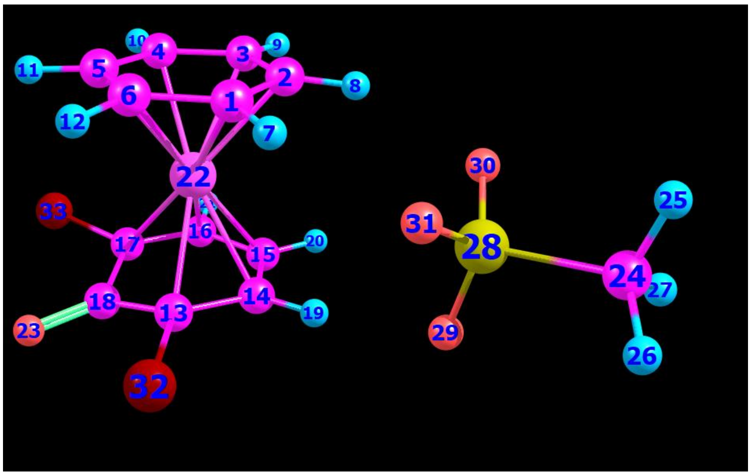

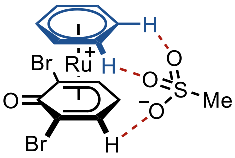

XVI

Fig S128: Structure of **XVI**. Left: With numbering of atoms respective to table S48.

Table S48 XYZ-coordinates for **XVI**:

Atom	X-Coordinate	Y-Coordinate	Z-Coordinate
C1	1.725931206	11.5913586	-1.373097344
C2	3.115212149	11.83743903	-1.368627185
C3	3.594242684	13.15622989	-1.222889477
C4	2.682487035	14.22375703	-1.086630587
C5	1.289656067	13.9729685	-1.058039224
C6	0.813078291	12.65853909	-1.200109367
H7	1.374725395	10.58661454	-1.581081872
H8	3.791772586	11.01518604	-1.572624514
H9	4.654617717	13.36028862	-1.295422024
H10	3.045380988	15.24301309	-1.059921605
H11	0.593820355	14.8006399	-1.010905076
H12	-0.251606688	12.47423201	-1.265221681

C13	0.910530345	12.48210584	-4.63809653
C14	2.210072562	11.95911532	-4.77713633
C15	3.314046611	12.83720171	-4.739381935
C16	3.096270942	14.2176971	-4.545239086
C17	1.779873285	14.69933343	-4.410717008
C18	0.587791772	13.91093347	-4.751826459
H19	2.370839846	10.88388451	-4.815960482
H20	4.321686025	12.44500898	-4.777574303
H21	3.935819467	14.88947002	-4.418834024
Ru22	2.150413283	13.11039442	-2.909080464
O23	-0.530266212	14.36700615	-4.906446101
C24	3.288342582	6.665289696	-3.294576636
H25	3.408461715	6.302792801	-2.274256164
H26	2.452299493	6.161764812	-3.778677847
H27	4.204752066	6.507993168	-3.862235083
S28	2.947536119	8.401400028	-3.239718685
O29	2.795600734	8.827952568	-4.633369218
O30	4.1003861	9.022782022	-2.585712322
O31	1.712345952	8.549741164	-2.4686102
Br32	-0.54188149	11.28880153	-4.627614432
Br33	1.517073958	16.53254217	-4.092820644

Geometry of TS XIII

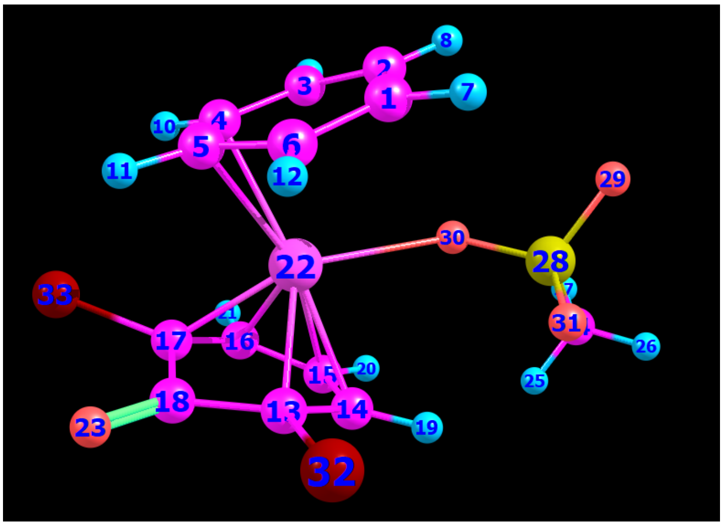

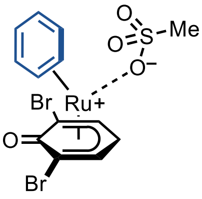

TS XIII

Fig S129: Structure of **TS XIII**. Left: With numbering of atoms respective to table S49.

Table S49 XYZ-coordinates for **TS XIII**:

Atom	X-Coordinate	Y-Coordinate	Z-Coordinate
C1	2.962402198	11.37445951	-1.00650775
C2	3.980614836	12.2036582	-0.564134231
C3	3.848046445	13.5820912	-0.715447206
C4	2.641905381	14.1391385	-1.164356794
C5	1.553881912	13.3033336	-1.490275613
C6	1.765130258	11.91516287	-1.506174379
H7	3.091775116	10.29935647	-0.997506356
H8	4.905900114	11.78233538	-0.197537946
H9	4.673138466	14.23974761	-0.470582453
H10	2.525258285	15.21424829	-1.204682852
H11	0.586744964	13.72098098	-1.738542999
H12	0.969455561	11.25695366	-1.831509297
C13	1.807705506	12.36642646	-4.895653983
C14	3.17480864	12.38687787	-5.248785816
C15	3.906426539	13.581993	-5.105563319
C16	3.269661203	14.71437993	-4.567461095
C17	1.884953248	14.64530376	-4.259748094
C18	0.99386871	13.58244302	-4.742783116
H19	3.675782172	11.46245796	-5.503122392
H20	4.975050485	13.58654397	-5.271041164
H21	3.83692021	15.60548564	-4.329737985
Ru22	2.926843487	13.09097069	-3.214720524
O23	-0.220637737	13.62834496	-4.754604043
C24	6.796149828	10.75289745	-4.61685577
H25	6.346793964	11.25326978	-5.474356133
H26	7.168613056	9.770018797	-4.903441702
H27	7.601158713	11.35878733	-4.203070117
S28	5.554557699	10.53966197	-3.376014904

O29	6.200915727	9.888404493	-2.244392628
O30	5.111587453	11.91068031	-3.038821645
O31	4.490393249	9.746947302	-3.980175196
Br32	0.872290893	10.73710189	-5.023932793
Br33	1.047310059	16.1882152	-3.573084163

Geometry of XVIII

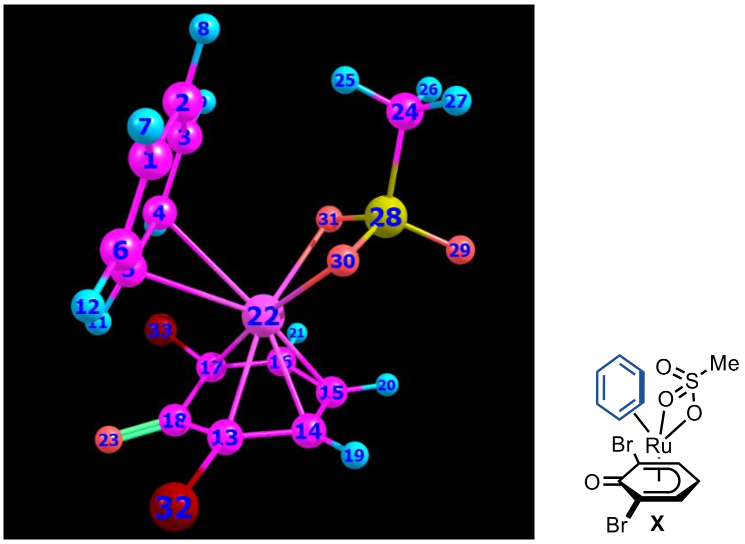

Fig S130: Structure of **XVIII**. Left: With numbering of atoms respective to table S50.

Table S50 XYZ-coordinates for **XVIII**:

Atom	X-Coordinate	Y-Coordinate	Z-Coordinate
C1	3.166899428	11.14532789	-0.497567329
C2	4.337429256	11.89962342	-0.361148451
C3	4.425214093	13.1553161	-0.918084795
C4	3.327994306	13.69800697	-1.603058656
C5	2.141858978	12.95868079	-1.70725737
C6	2.079596189	11.66599978	-1.160722003
H7	3.112462813	10.1542331	-0.062193531
H8	5.177205806	11.49034471	0.189203362
H9	5.332531998	13.7384361	-0.817786428
H10	3.3528025	14.73601329	-1.914485309
H11	1.230981004	13.43385118	-2.048326715
H12	1.160428088	11.09894364	-1.239281002

C13	1.147092474	12.50170719	-4.727261861
C14	2.117153714	12.08582967	-5.670813098
C15	3.080679054	13.00242916	-6.137986993
C16	3.08874482	14.30204967	-5.574523534
C17	2.095499521	14.67149529	-4.64279028
C18	0.856738666	13.90980535	-4.442441734
H19	2.178960085	11.04294185	-5.957341627
H20	3.871058967	12.68308376	-6.804159619
H21	3.908987556	14.97749826	-5.785413717
Ru22	3.154311545	12.89049181	-4.009416572
O23	-0.138231052	14.32325053	-3.872675297
C24	6.718092585	11.0453048	-2.809776117
H25	6.392722573	11.34638943	-1.818549992
H26	7.672079225	11.50615651	-3.065004092
H27	6.787406122	9.960779438	-2.889595092
S28	5.548855979	11.58528488	-3.994481136
O29	6.02024147	11.16584838	-5.288367148
O30	4.214485341	11.02003467	-3.624066655
O31	5.381890802	13.05882634	-3.850030599
Br32	-0.112223159	11.22895435	-4.130567747
Br33	2.157472453	16.40640296	-3.903041033

Geometry of SI-5

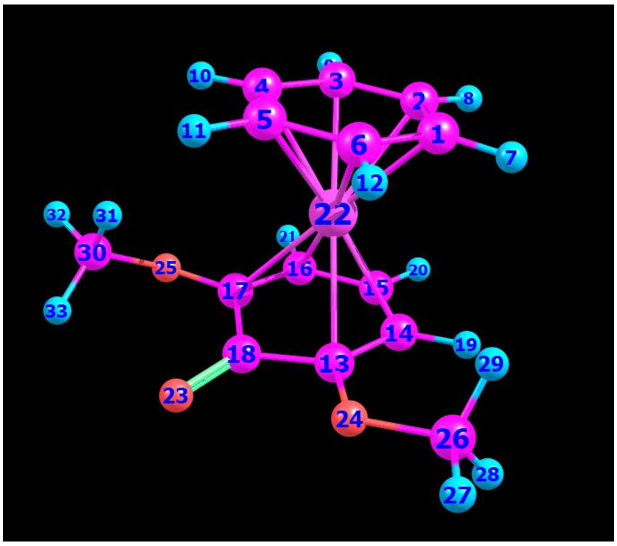

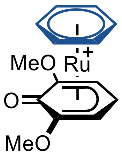

SI-5

Fig S131: Structure of **SI-5**. Left: With numbering of atoms respective to table S51.

Table S51 XYZ-coordinates for **SI-5**:

Atom	X-Coordinate	Y-Coordinate	Z-Coordinate
C1	1.930621582	11.48140802	-1.378088072
C2	3.222597582	12.04866552	-1.327507461
C3	3.370703286	13.4461224	-1.188723569
C4	2.228434639	14.27027892	-1.107966895
C5	0.9355172	13.7039504	-1.152949744
C6	0.789755009	12.30941366	-1.290777462
H7	1.812977082	10.41922708	-1.547568819
H8	4.094992603	11.42028893	-1.449589952
H9	4.35646444	13.89185194	-1.209170144
H10	2.345613696	15.34554538	-1.070974007
H11	0.061649845	14.34172045	-1.143566144
H12	-0.197169886	11.87866366	-1.398935529
C13	0.754835817	12.52742731	-4.748099493
C14	1.985251215	11.83887894	-4.771825242
C15	3.201162627	12.55644216	-4.725142805
C16	3.167291109	13.95766724	-4.596133507
C17	1.935721663	14.64854812	-4.554679238
C18	0.668960955	13.9907466	-4.878957128
H19	2.009956937	10.75779452	-4.777942327
H20	4.144569059	12.02762363	-4.724115348
H21	4.081449144	14.52471714	-4.471139153
Ru22	2.082286115	13.03414611	-2.934083736
O23	-0.385346379	14.57950838	-5.095835212
O24	-0.430796737	11.92543754	-4.77083132
O25	2.028519432	15.97546838	-4.417165945
C26	-0.475971082	10.5067278	-4.665447339
H28	-1.531225528	10.24677032	-4.652966944
H29	0.008790562	10.03790456	-5.524519247

H30	-0.002921982	10.17248306	-3.738647614
C31	0.981299139	16.69137527	-3.757076421
H32	0.633339706	16.13685987	-2.88401315
H33	1.430949529	17.62978516	-3.438825424
H34	0.14694119	16.87389134	-4.429812333

Geometry of XII

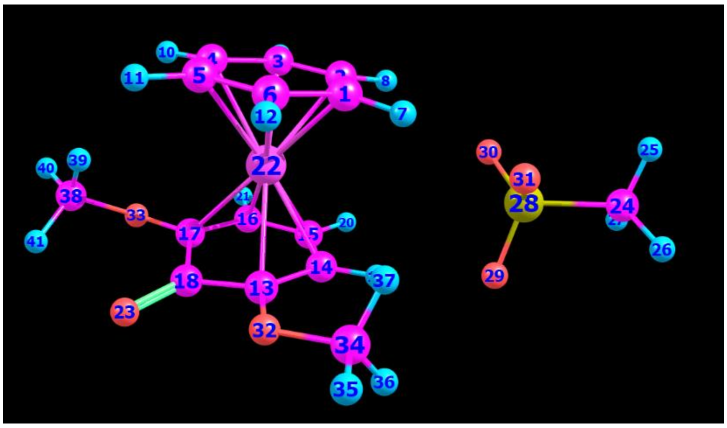

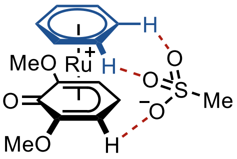

XII

Fig S132: Structure of **XII**. Left: With numbering of atoms respective to table S52.

Table S52 XYZ-coordinates for **XII**:

Atom	X-Coordinate	Y-Coordinate	Z-Coordinate
C1	1.869132721	11.49370778	-1.386255028
C2	3.182665539	12.00987381	-1.338397681
C3	3.388553979	13.39886264	-1.192023257
C4	2.280487538	14.26812738	-1.103736553
C5	0.965205968	13.75344666	-1.143281907
C6	0.763394091	12.36671323	-1.286784359
H7	1.719538513	10.437177	-1.581703233
H8	4.01689429	11.3369672	-1.487825297
H9	4.390902112	13.80586395	-1.216824241
H10	2.439429038	15.33801083	-1.064574581
H11	0.117675148	14.42554194	-1.124271197
H12	-0.241002436	11.97763679	-1.393411554
C13	0.762522425	12.52031555	-4.746696946
C14	1.998940989	11.84261673	-4.768825902

C15	3.205133443	12.57555558	-4.719396393
C16	3.157673633	13.97687903	-4.593588445
C17	1.918495279	14.65412713	-4.557402769
C18	0.659310497	13.98162109	-4.877308814
H19	2.047937318	10.75856438	-4.768382898
H20	4.153271874	12.05491914	-4.710080144
H21	4.065154613	14.55433142	-4.467663363
Ru22	2.076162258	13.04859213	-2.933178204
O23	-0.402878937	14.56011021	-5.092948576
C24	3.64488692	6.711348115	-3.240557791
H25	3.861110352	6.433263102	-2.209612538
H26	2.865863954	6.069119654	-3.649816105
H27	4.547391919	6.644476248	-3.847149216
S28	3.069824246	8.385743193	-3.264383251
O29	2.794857798	8.700142579	-4.668344576
O30	4.151429204	9.197761868	-2.706811684
O31	1.861100663	8.410680611	-2.437491465
O32	-0.416046617	11.90218054	-4.768493624
O33	1.996799911	15.98496684	-4.428964424
C34	-0.433771466	10.48193116	-4.66641767
H35	-1.483993339	10.20237203	-4.643598305
H36	0.051658681	10.0258202	-5.531821985
H37	0.059623073	10.15041477	-3.749832501
C38	0.952300318	16.68822687	-3.753157554
H39	0.638726094	16.13875823	-2.863941297
H40	1.389139348	17.64047176	-3.458733065
H41	0.09669753	16.84358621	-4.40587296

Geometry of TS IX

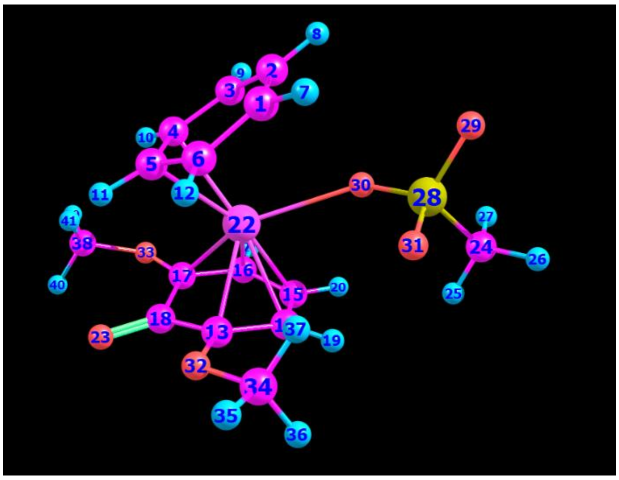

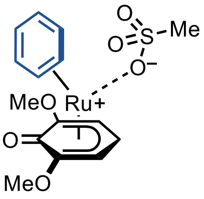

TS IX

Fig S133: Structure of **TS IX**. Left: With numbering of atoms respective to table S53.

Table S53 XYZ-coordinates for **TS IX**:

Atom	X-Coordinate	Y-Coordinate	Z-Coordinate
C1	3.22406368	11.21610074	-0.99510217
C2	3.978310957	12.24557987	-0.468459949
C3	3.550232005	13.56259229	-0.65462092
C4	2.286146002	13.83773649	-1.190325899
C5	1.466825523	12.78262509	-1.643964888
C6	2.009599225	11.48415997	-1.652606798
H7	3.584790215	10.19624395	-0.950713381
H8	4.933534128	12.04510485	-0.003229488
H9	4.176460957	14.38841911	-0.338774414
H10	1.926152318	14.85774579	-1.212830434
H11	0.447719921	12.95541083	-1.964315041
H12	1.426782827	10.66781508	-2.060336578
C13	1.810769249	12.32696693	-4.967682702
C14	3.1923449	12.33274815	-5.2740135
C15	3.934036226	13.51948765	-5.11265559
C16	3.313311445	14.64967302	-4.563539424
C17	1.920231284	14.64254336	-4.278228529
C18	1.045025611	13.56473607	-4.738899974
H19	3.700315146	11.41727598	-5.543470042

H20	5.002071008	13.51727521	-5.277869247
H21	3.885824249	15.53572999	-4.31761574
Ru22	2.977801277	12.99846014	-3.228154959
O23	-0.182654457	13.59714458	-4.73253475
C24	6.976968863	11.00862606	-4.593779078
H25	6.445195499	11.40158003	-5.459620398
H26	7.43173579	10.0484541	-4.835031438
H27	7.7362437	11.71502528	-4.260797896
S28	5.81905854	10.7593185	-3.279809231
O29	6.578930803	10.21951411	-2.158701926
O30	5.2746346	12.10017457	-2.976773428
O31	4.803049407	9.846366411	-3.790720097
O32	1.040225087	11.2400899	-5.055380101
O33	1.466904213	15.80322171	-3.782170786
C34	1.677763196	9.981461004	-5.251675745
H35	0.891049526	9.236249911	-5.162799263
H36	2.123641012	9.926844069	-6.247676634
H37	2.445430947	9.812589582	-4.49326567
C38	0.169755974	15.91892215	-3.194823144
H39	0.205121276	16.84013663	-2.615834021
H40	-0.599383775	15.98373678	-3.961355475
H41	-0.048593579	15.08144737	-2.534934103

Geometry of XIX

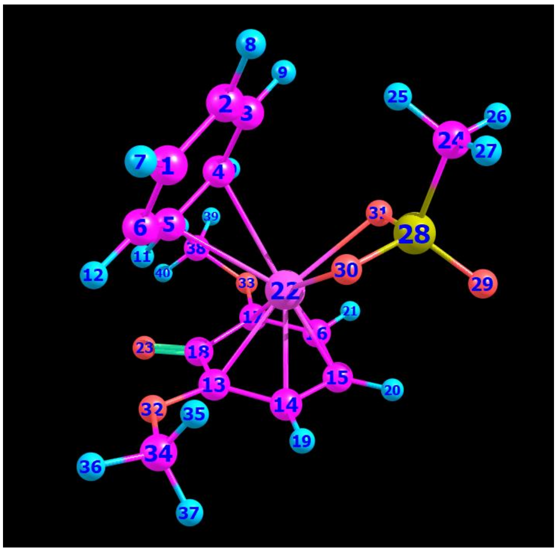

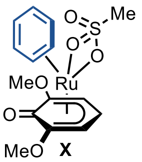

Fig S134: Structure of **XIX**. Left: With numbering of atoms respective to table S54.

Table S54 XYZ-coordinates for **XIX**:

Atom	X-Coordinate	Y-Coordinate	Z-Coordinate
C1	2.934670118	10.91368755	-0.743786321
C2	4.101479613	11.63692531	-0.470088921
C3	4.228761263	12.93613745	-0.903295192
C4	3.183062167	13.55002849	-1.609602803
C5	1.99879305	12.84117736	-1.851934179
C6	1.894529837	11.50608245	-1.421099916
H7	2.845292743	9.888266445	-0.403873617
H8	4.905262576	11.16835581	0.086618456
H9	5.129635278	13.50011666	-0.693136721
H10	3.248126436	14.60956662	-1.827589935
H11	1.110042983	13.35163765	-2.200033629
H12	0.974974159	10.96219448	-1.598820418
C13	1.091100131	12.42275581	-4.823827148
C14	2.120224829	11.99748639	-5.692839195
C15	3.069099957	12.92430578	-6.181933055
C16	3.004085581	14.25133671	-5.703345778
C17	1.969672432	14.6713668	-4.835032116
C18	0.789412177	13.84437206	-4.603757102

H19	2.244470249	10.94749922	-5.923984669
H20	3.883005004	12.59980578	-6.81611193
H21	3.795137953	14.95611725	-5.932798202
Ru22	3.122622432	12.92785513	-4.066821654
O23	-0.24533533	14.23271039	-4.064132375
C24	6.852934696	11.42542639	-2.769738443
H25	6.46097723	11.65049742	-1.782073183
H26	7.759263718	11.99658817	-2.968871833
H27	7.046112361	10.35872981	-2.879767416
S28	5.669953362	11.87698139	-3.982613618
O29	6.235975406	11.55079821	-5.268808989
O30	4.404932859	11.1499182	-3.679164394
O31	5.354583398	13.31852789	-3.805013171
O32	0.167003343	11.59360196	-4.308011444
O33	2.019214609	15.97822068	-4.492784948
C34	0.375492685	10.19739486	-4.452176381
H35	1.34578021	9.901940704	-4.043720543
H36	-0.418540355	9.717357208	-3.884656004
H37	0.307877061	9.896064939	-5.500672231
C38	1.406635519	16.41907419	-3.283665072
H39	1.936697058	17.32711939	-2.999140652
H40	0.350119793	16.63240855	-3.436023958
H41	1.510071099	15.67834613	-2.49156221

Geometry of Scan 1

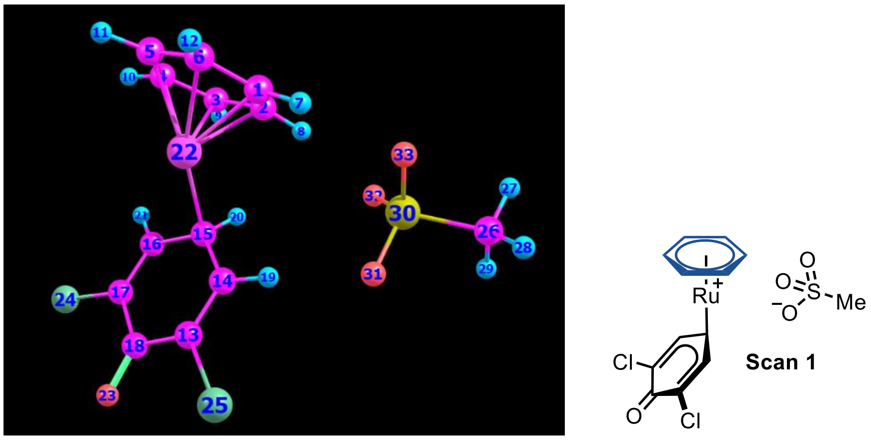

Fig S135: Structure of **Scan 1**. Left: With numbering of atoms respective to table S55.

Table S55 XYZ-coordinates for **Scan 1**:

Atom	X-Coordinate	Y-Coordinate	Z-Coordinate
C1	2,098697399	11,41842105	-0,674079008
C2	3,360924891	11,79359584	-1,202007196
C3	3,764770337	13,14746721	-1,126351166
C4	2,897351878	14,09542817	-0,524674103
C5	1,680943436	13,69117462	0,085385977
C6	1,283199766	12,35570779	0,01068574
H7	1,742814274	10,41168516	-0,871578733
H8	3,923128645	11,06515869	-1,777197171
H9	4,663197264	13,48261783	-1,629148012
H10	3,137486713	15,14980989	-0,589356823
H11	1,00808117	14,44001355	0,483381429
H12	0,297990443	12,0624045	0,350633802
C13	0,896677415	12,52124797	-5,821648344
C14	1,688043577	12,0741042	-4,812010101
C15	2,583977823	12,95631538	-4,103881815
C16	2,58139652	14,33430447	-4,531999609
C17	1,784107171	14,77100613	-5,542146411
C18	0,928195333	13,89176421	-6,356564736
H19	1,683283434	11,01539279	-4,561916494
H20	3,571164709	12,53817347	-3,906914726

H21	3,268830643	15,03146016	-4,064388554
Ru22	1,859213549	13,03198862	-2,038695889
O23	0,308514333	14,26028948	-7,341822363
Cl24	1,819453576	16,42705462	-6,013964392
Cl25-	0,155959619	11,42721321	-6,638199578
C26	3,279722229	6,848718923	-3,493307598
H27	3,990606736	6,546279132	-2,725217182
H28	2,417662861	6,182601463	-3,494300992
H29	3,757990906	6,849637947	-4,4721337
S30	2,724408418	8,490946133	-3,135187417
O31	1,776712892	8,841040686	-4,19413087
O32	3,920920593	9,33568816	-3,148747312
O33	2,094139809	8,428816237	-1,814669602

Geometry of Scan 2

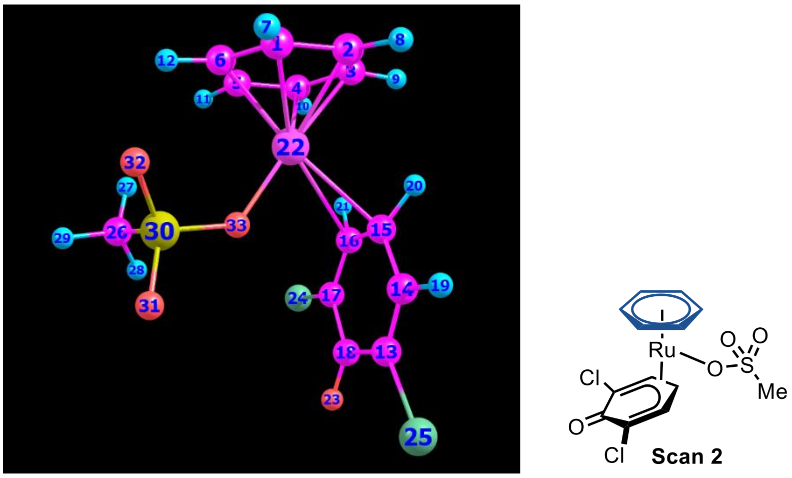

Fig S136: Structure of **Scan 2**. Left: With numbering of atoms respective to table S56.

Table S56 XYZ-coordinates for **Scan 2**:

Atom	X-Coordinate	Y-Coordinate	Z-Coordinate
C1	4,342057759	11,26339823	-0,863352398
C2	4,75551213	12,53650162	-1,337522366
C3	3,89287772	13,6442085	-1,233544871
C4	2,596390804	13,46177622	-0,722627571
C5	2,186442676	12,19386883	-0,232822608

C6	3,071551247	11,10175829	-0,267483382
H7	4,969838467	10,39859883	-1,035358895
H8	5,695339512	12,63007538	-1,86681798
H9	4,167115434	14,58964942	-1,683527394
H10	1,876522498	14,26838794	-0,782260216
H11	1,159656511	12,04766756	0,078443921
H12	2,730263337	10,11477192	0,011330587
C13	2,393279154	12,34749152	-6,710756497
C14	3,36062188	12,46205886	-5,745903243
C15	3,110636188	13,08851113	-4,486679288
C16	1,808003695	13,59075822	-4,262795919
C17	0,807441754	13,34379013	-5,167287901
C18	1,011287676	12,73236615	-6,465133286
H19	4,369509404	12,13412271	-5,97054113
H20	3,959575621	13,57253305	-4,012155135
H21	1,594217556	14,21493716	-3,400896066
Ru22	2,903606529	11,97957936	-2,254950575
O23	0,103840635	12,57244703	-7,294764097
Cl24	-0,803458519	13,86693892	-4,813935961
Cl25	2,761228452	11,65385475	-8,243307914
C26	0,148168096	9,173207063	-2,233144245
H27	0,210299462	9,776218701	-1,328481752
H28	-0,51861103	9,631818167	-2,961575512
H29	-0,179309452	8,161515703	-1,995101773
S30	1,74961196	9,047607534	-2,948130832
O31	1,642731387	8,294577288	-4,173625483
O32	2,662030218	8,531943259	-1,954481967
O33	2,099370365	10,49058823	-3,316961197

Geometry of Scan 3

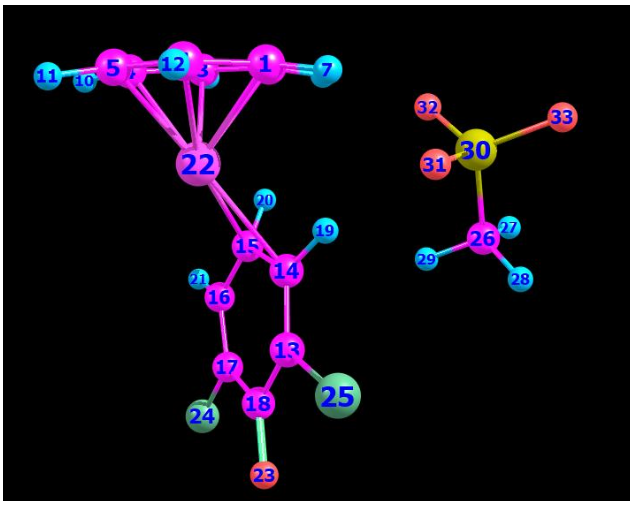

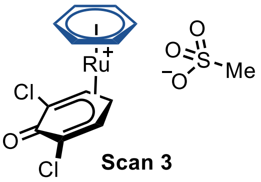

Fig S137: Structure of **Scan 3**. Left: With numbering of atoms respective to table S57.

Table S57 XYZ-coordinates for **Scan 3**:

Atom	X-Coordinate	Y-Coordinate	Z-Coordinate
C1	1,752527	11,49613628	-0,364288337
C2	3,087661217	11,71977429	-0,79756842
C3	3,632222569	13,02637344	-0,755263437
C4	2,826071723	14,10169159	-0,316022503
C5	1,499857577	13,86260027	0,119950091
C6	0,967303796	12,56125199	0,122939403
H7	1,314249455	10,51907526	-0,526253068
H8	3,629527913	10,90701794	-1,276628883
H9	4,610680142	13,22197433	-1,175205477
H10	3,18486	15,11903753	-0,408512291
H11	0,856391496	14,7060266	0,339148819
H12	-0,077479982	12,40323964	0,3577392
C13	0,800984169	12,07158166	-5,077810262
C14	1,731931707	11,96764288	-4,075453573
C15	2,847413607	12,83755142	-4,053459852
C16	3,031895201	13,7089986	-5,185899616
C17	2,222514895	13,67596218	-6,292557518
C18	0,996590605	12,88297528	-6,289763398
H19	1,645628612	11,13435442	-3,358516089

H20	3,762951011	12,51394026	-3,567241343
H21	3,916227303	14,33663535	-5,204333528
Ru22	1,805958608	13,0241122	-1,859548124
O23	0,190120828	12,88013912	-7,218515099
Cl24	2,554974289	14,62645699	-7,677940721
Cl25	-0,62237395	11,11674658	-5,037972411
C26	3,103010619	8,603632799	-4,667474452
H27	4,005327696	8,090136098	-4,997154033
H28	2,219009967	8,11945199	-5,081007173
H29	3,135242792	9,650459972	-4,967520502
S30	3,017955537	8,517670832	-2,900849059
O31	1,775176295	9,200224822	-2,520723886
O32	4,206538716	9,216190459	-2,402863697
O33	3,006697714	7,098464653	-2,559583709

Geometry of XIII

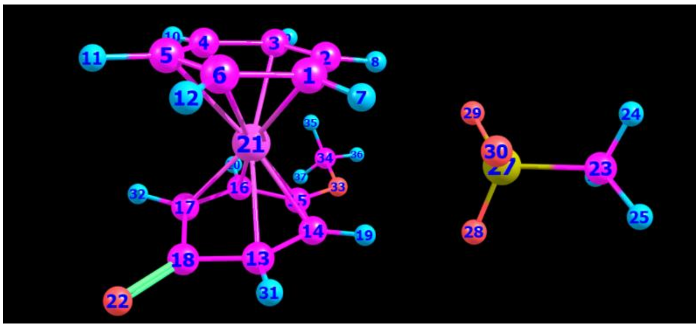

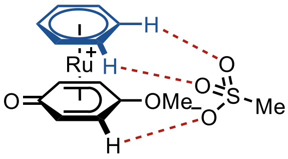

XIII

Fig S138: Structure of **XIII**. Left: With numbering of atoms respective to table S58.

Table S58 XYZ-coordinates for **XIII**:

Atom	X-Coordinate	Y-Coordinate	Z-Coordinate
C1	1,672982142	11,60502425	-1,374337502
C2	3,059996097	11,86889752	-1,333188787
C3	3,515045044	13,18904969	-1,154646871
C4	2,58357771	14,24264435	-1,027480656
C5	1,193620694	13,9719178	-1,018228743
C6	0,739832575	12,65306524	-1,18877199
H7	1,340257278	10,59902269	-1,602004837

H8	3,752867029	11,05930996	-1,531675147
H9	4,573854999	13,40962804	-1,195062037
H10	2,931738493	15,2655924	-0,972228933
H11	0,485052542	14,78715415	-0,953648725
H12	-0,320915287	12,4505213	-1,259187904
C13	0,894969768	12,53966089	-4,619841134
C14	2,197621791	12,02709191	-4,747922677
C15	3,313452126	12,90312201	-4,749754571
C16	3,083591827	14,27562064	-4,47276996
C17	1,764317233	14,75592891	-4,34485912
C18	0,594793181	13,96409972	-4,704932232
H19	2,377757216	10,95595343	-4,793362435
H20	3,914648393	14,94920256	-4,311975509
Ru21	2,097257878	13,15377517	-2,87167559
O22	-0,537943568	14,42699841	-4,84620127
C23	3,406254215	6,705161188	-3,29021561
H24	3,545501088	6,359416439	-2,26649427
H25	2,583474635	6,168775565	-3,761594394
H26	4,323138654	6,569811604	-3,862815557
S27	3,010809443	8,430747051	-3,254738554
O28	2,834948487	8,83488189	-4,651542393
O29	4,148351365	9,094858811	-2,616049582
O30	1,776741128	8,54876394	-2,476109438
H31	0,063117953	11,84652077	-4,575323359
H32	1,613498555	15,79877428	-4,092317439
O33	4,508873813	12,3483403	-4,917010075
C34	5,664417949	13,14912296	-4,681788998
H35	5,676985883	13,50706639	-3,649494561
H36	6,514221419	12,49442397	-4,854948795
H37	5,6997449	13,99217533	-5,374459553

Geometry of TS X

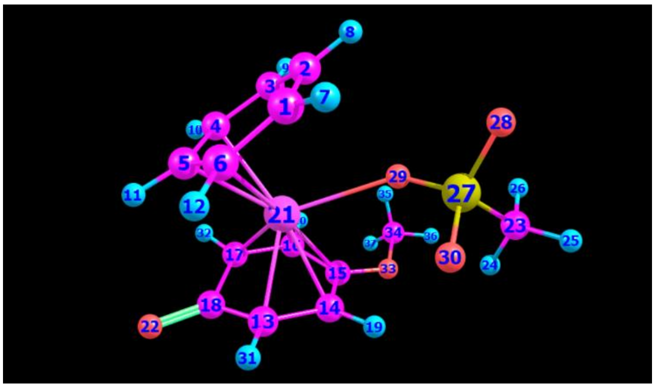

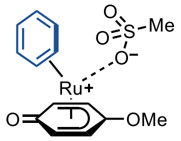

TS X

Fig S139: Structure of **TS X**. Left: With numbering of atoms respective to table S59.

Table S59 XYZ-coordinates for **TS X**:

Atom	X-Coordinate	Y-Coordinate	Z-Coordinate
C1	3,037818415	11,32117377	-1,008509042
C2	3,883409144	12,28136479	-0,4857188
C3	3,53964494	13,63012798	-0,611865948
C4	2,278991357	14,009481	-1,092347496
C5	1,373267353	13,02698559	-1,549165374
C6	1,815312977	11,69034649	-1,599205216
H7	3,329943706	10,27857405	-1,008859425
H8	4,8414304	12,00134103	-0,070589057
H9	4,233709524	14,39953308	-0,295276663
H10	1,985706408	15,0510269	-1,079801981
H11	0,366994378	13,29336309	-1,846300391
H12	1,160524229	10,92782128	-2,001434367
C13	1,73583883	12,55972467	-4,861791006
C14	3,096832516	12,58665818	-5,224259052
C15	3,848626658	13,78141731	-5,102668584
C16	3,259488884	14,85875566	-4,402760652
C17	1,880794461	14,79630031	-4,063610212
C18	0,978162349	13,77801667	-4,586686339
H19	3,604614498	11,68430218	-5,539565939
H20	3,845402612	15,72192082	-4,116674601
Ru21	2,863446872	13,15894167	-3,139157971

O22	-0,250127482	13,83673459	-4,527976596
C23	6,900495876	11,0380364	-4,54106903
H24	6,47618157	11,5540142	-5,400577072
H25	7,29720706	10,06575715	-4,831334153
H26	7,681019446	11,64482712	-4,083495711
S27	5,615558653	10,78244577	-3,353442404
O28	6,231524662	10,10544549	-2,217470667
O29	5,139808993	12,13452065	-2,996722147
O30	4,588408479	9,986207165	-4,017003275
H31	1,19206606	11,62427031	-4,92932238
H32	1,443187715	15,62561979	-3,519373814
O33	5,102626072	13,7447009	-5,526727383
C34	5,983146485	14,79166309	-5,129438848
H35	6,053472213	14,83498097	-4,040733144
H36	6,951722732	14,53609871	-5,551144953
H37	5,648518895	15,75190141	-5,527015066

Geometry of XV

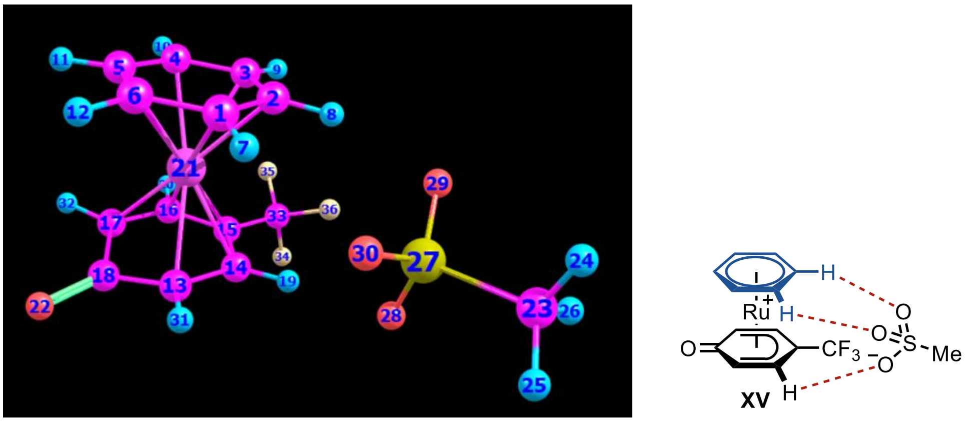

Fig S140: Structure of **XV**. Left: With numbering of atoms respective to table S60.

Table S60 XYZ-coordinates for **XV**:

Atom	X-Coordinate	Y-Coordinate	Z-Coordinate
C1	1,632231233	11,63716959	-1,317800749
C2	3,039143617	11,69553663	-1,352256377
C3	3,689861244	12,94538612	-1,260620168

C4	2,933330486	14,12743904	-1,105296286
C5	1,523429127	14,05954983	-1,045244956
C6	0,869294961	12,8179064	-1,159318366
H7	1,147636398	10,68584694	-1,500176957
H8	3,599967022	10,7876457	-1,549831478
H9	4,765606436	13,00470809	-1,365003792
H10	3,42772992	15,08978719	-1,093892918
H11	0,94338894	14,97201108	-0,995699719
H12	-0,211179215	12,77505313	-1,192436715
C13	1,115519512	12,17774975	-4,6290874
C14	2,492117823	11,94346642	-4,764974821
C15	3,389360922	13,03917993	-4,714948496
C16	2,881805489	14,35033415	-4,538636764
C17	1,498758183	14,54283102	-4,405746349
C18	0,5239531	13,50782016	-4,742152852
H19	2,864546713	10,92212198	-4,79292665
H20	3,56319647	15,18130686	-4,404972347
Ru21	2,213041865	13,06109159	-2,897980981
O22	-0,67306504	13,71932074	-4,905010504
C23	2,963994081	6,591007378	-3,253483692
H24	2,851000188	6,217461756	-2,236348916
H25	2,149819583	6,23066403	-3,881257767
H26	3,92421826	6,282338102	-3,665207452
S27	2,906402564	8,35982671	-3,214376574
O28	3,063275842	8,801436942	-4,602362685
O29	4,018696464	8,784291916	-2,361595752
O30	1,600957306	8,71354119	-2,655692068
H31	0,447309432	11,32710876	-4,563890786
H32	1,127808728	15,53345027	-4,171588698
C33	4,870565061	12,80943032	-4,845273848
F34	5,231541841	12,8470596	-6,134373647

F35	5,578556296	13,74350833	-4,206916348
F36	5,235453201	11,62101657	-4,363456079

Geometry of TS XII

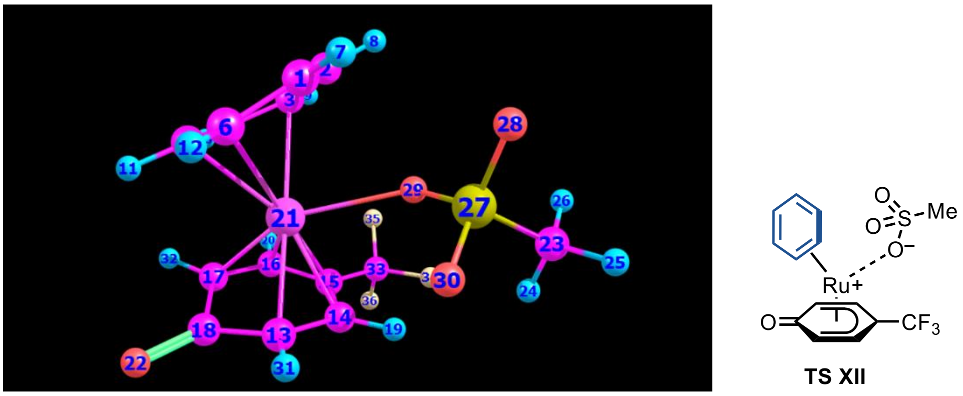

Fig S141: Structure of **TS XII**. Left: With numbering of atoms respective to table S61.

Table S61 XYZ-coordinates for **TS XII**:

Atom	X-Coordinate	Y-Coordinate	Z-Coordinate
C1	3,238966478	11,50233794	-0,903217762
C2	4,18550503	12,46671894	-0,642937248
C3	3,897755408	13,81115903	-0,940371737
C4	2,588922891	14,20397748	-1,253541774
C5	1,6039883	13,21947846	-1,475832612
C6	1,981458566	11,871636	-1,426720911
H7	3,480131521	10,45454785	-0,785462325
H8	5,174292308	12,18964869	-0,304201243
H9	4,664002754	14,56870892	-0,832011198
H10	2,336857221	15,25349459	-1,33382941
H11	0,586983039	13,50133004	-1,714374335
H12	1,257898295	11,1083287	-1,684095192
C13	2,175919133	12,37282842	-5,064430882
C14	3,508049004	12,74284813	-5,305735501
C15	3,956731563	14,03326265	-4,946396192
C16	3,040782175	14,94225424	-4,362331945
C17	1,698348412	14,54284343	-4,18012216
C18	1,126462008	13,35850455	-4,817460448

H19	4,217555656	11,99801569	-5,633857815
H20	3,385380162	15,89759617	-3,983624921
Ru21	3,029857738	13,16473792	-3,205628331
O22	-0,074906694	13,12959475	-4,896298777
C23	6,609643382	10,13317394	-4,499531069
H24	6,444950403	10,72217293	-5,400637403
H25	6,763440893	9,08491061	-4,753504103
H26	7,464302231	10,51892299	-3,945360577
S27	5,173469736	10,24762913	-3,474535761
O28	5,445022567	9,442672715	-2,290456056
O29	5,048009031	11,68658504	-3,152445749
O30	4,05033255	9,761158397	-4,267094341
H31	1,886763701	11,34157653	-5,225065417
H32	1,023549333	15,21045767	-3,656922321
C33	5,384832494	14,45762892	-5,145553246
F34	6,181945537	13,44123655	-5,464112489
F35	5,887794774	15,04704744	-4,055691906
F36	5,461945259	15,35163778	-6,144030963

Geometry of XIV

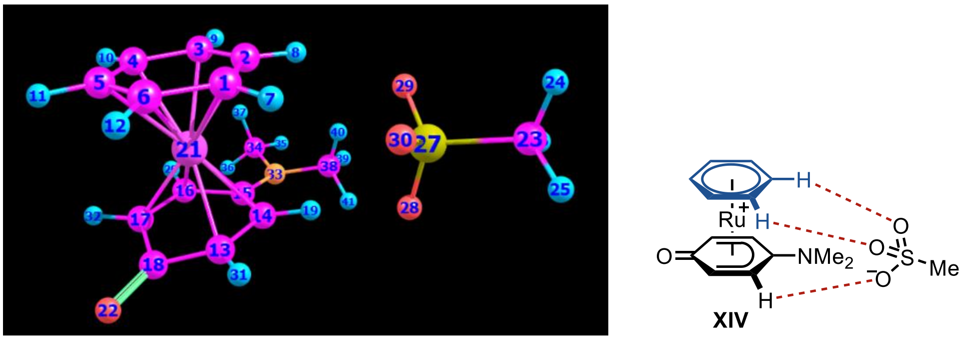

Fig S142: Structure of **XIV**. Left: With numbering of atoms respective to table S62.

Table S62 XYZ-coordinates for **XIV**:

Atom	X-Coordinate	Y-Coordinate	Z-Coordinate
C1	1,736851218	11,58226509	-1,349652768
C2	3,130367943	11,81326952	-1,3569967
C3	3,622382332	13,1260012	-1,244518687

C4	2,717395518	14,20523664	-1,104593279
C5	1,32575845	13,96602946	-1,024913142
C6	0,833423971	12,65625604	-1,157237249
H7	1,369915906	10,57930024	-1,536498055
H8	3,795994841	10,98038353	-1,547779255
H9	4,68368678	13,31982092	-1,328924494
H10	3,089620745	15,22096848	-1,085603054
H11	0,639644331	14,79924237	-0,945695821
H12	-0,233486968	12,47645799	-1,177178893
C13	1,02793567	12,29300437	-4,619993554
C14	2,386895173	11,94036736	-4,740965554
C15	3,405663071	12,93913187	-4,856668213
C16	2,982572045	14,26518327	-4,525024246
C17	1,616733622	14,59196678	-4,404021605
C18	0,54871449	13,66120702	-4,734605917
H19	2,642702747	10,88710047	-4,737595907
H20	3,715174008	15,04295302	-4,362965154
Ru21	2,137740313	13,0835819	-2,898765017
O22	-0,637036521	13,97934131	-4,871169245
C23	2,88232622	6,502913978	-3,180935478
H24	2,96198855	6,12558535	-2,162015537
H25	2,028894844	6,05112762	-3,685391909
H26	3,799199657	6,297921569	-3,732517996
S27	2,640245263	8,256252376	-3,118026401
O28	2,540408522	8,700232655	-4,509918408
O29	3,81213863	8,8041127	-2,43361969
O30	1,399494854	8,467732493	-2,369759478
H31	0,293694283	11,49903849	-4,546575813
H32	1,346945388	15,61400272	-4,165189108
N33	4,692585168	12,62942925	-5,086942995
C34	5,720839536	13,62136141	-4,850216617

H35	6,666625208	13,23356952	-5,221358966
H36	5,498996961	14,54364862	-5,387409951
H37	5,827184458	13,84756273	-3,782192545
C38	5,105422457	11,24006882	-5,08090165
H39	6,129538109	11,18332075	-5,442952637
H40	5,05741623	10,80639299	-4,075507622
H41	4,475173269	10,64910902	-5,744894498

Geometry of TS XI

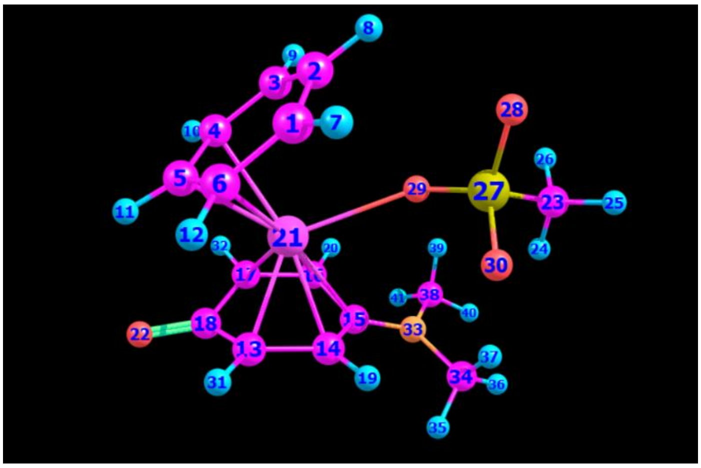

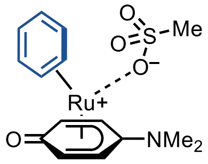

TS XI

Fig S143: Structure of **TS XI**. Left: With numbering of atoms respective to table S63.

Table S63 XYZ-coordinates for **TS XI**:

Atom	X-Coordinate	Y-Coordinate	Z-Coordinate
C1	3,032562684	11,33136017	-0,926651918
C2	3,555416145	12,34205669	-0,133656162
C3	3,005836526	13,61770103	-0,22001693
C4	1,857312537	13,85987987	-0,98465795
C5	1,244396652	12,80692155	-1,701676456
C6	1,90129079	11,55451868	-1,728943257
H7	3,506103409	10,35714524	-0,94515528
H8	4,443639679	12,16559773	0,454593827
H9	3,460686737	14,44174554	0,317366469
H10	1,399905551	14,84079402	-0,975679744
H11	0,283348446	12,94536307	-2,18115795
H12	1,474044914	10,73773816	-2,296419632

C13	2,037365957	12,6591603	-4,773181788
C14	3,438989622	12,67303288	-4,978753484
C15	4,172493812	13,90092956	-4,931868052
C16	3,519739766	14,92396944	-4,175680977
C17	2,114798475	14,89017456	-3,98667995
C18	1,261271547	13,8788006	-4,594821623
H19	3,945818865	11,75286743	-5,234135395
H20	4,077430854	15,77580103	-3,811593687
Ru21	2,943511416	13,24254397	-2,951210567
O22	0,029369338	13,94603855	-4,661558125
C23	7,877713233	12,59299092	-2,705463261
H24	7,826590741	13,03335206	-3,699108276
H25	8,608936999	11,7854838	-2,689667194
H26	8,137412812	13,35261717	-1,969304915
S27	6,299128406	11,91443409	-2,279740496
O28	6,464648868	11,32915521	-0,953929168
O29	5,373094506	13,06325083	-2,286532094
O30	5,972331617	10,92891817	-3,305429706
H31	1,499494023	11,72468898	-4,888979608
H32	1,63593827	15,72235453	-3,482545629
N33	5,424875909	14,00496995	-5,363360607
C34	6,061262862	12,89226481	-6,044618258
H35	5,395180372	12,494953	-6,812341364
H36	6,96479299	13,25486261	-6,52937979
H37	6,319546158	12,08834214	-5,35098557
C38	6,214293598	15,17614716	-5,037854241
H39	6,450514737	15,21247616	-3,970558043
H40	7,143317169	15,13875601	-5,600976057
H41	5,681098707	16,08788805	-5,313205109

Geometry of I-conf0

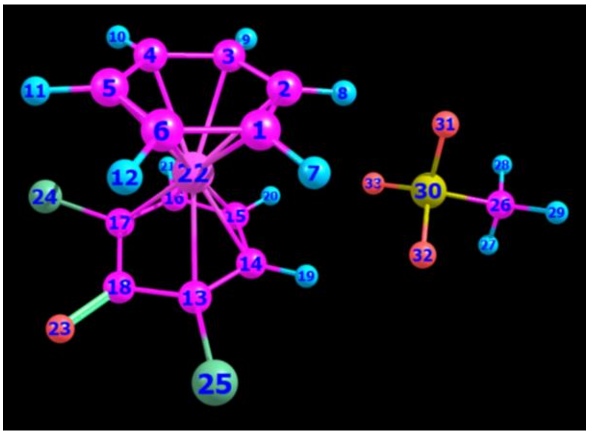

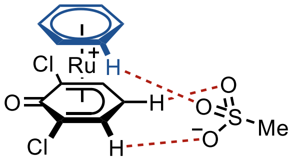

I-conf0

Fig S144: Structure of **I-conf0**. Left: With numbering of atoms respective to table S64.

Table S64 XYZ-coordinates for **I-conf0**:

Atom	X-Coordinate	Y-Coordinate	Z-Coordinate
C1	2.02756008	11.51107353	-1.436337833
C2	3.375582517	11.92523761	-1.462695086
C3	3.687021785	13.29279034	-1.309507808
C4	2.65410302	14.23613505	-1.124706319
C5	1.305763237	13.81188212	-1.060717417
C6	0.992087504	12.45101063	-1.217844345
H7	1.780290668	10.47739677	-1.641354496
H8	4.156617906	11.20639734	-1.692914967
H9	4.711365167	13.6279194	-1.40829205
H10	2.887951143	15.29203188	-1.085764887
H11	0.513314161	14.54414107	-0.975786813
H12	-0.042130753	12.13407508	-1.254050745
C13	1.062243869	12.28428561	-4.675761436
C14	2.432397778	12.00815614	-4.845100917
C15	3.364765945	13.0659351	-4.80907307
C16	2.90907701	14.38111295	-4.584346829
C17	1.530752386	14.62107491	-4.416415149
C18	0.489725957	13.63584298	-4.74176302
H19	2.78819584	10.98294837	-4.898113162
H20	4.42150473	12.8360202	-4.860609022
H21	3.614089973	15.19326854	-4.459245124
Ru22	2.211300288	13.09354216	-2.952861798
O23	-0.697109809	13.88750317	-4.850017635

Cl24	1.003030508	16.22092515	-4.086469819
Cl25	-0.049506601	10.9754088	-4.663600508
C26	6.503851672	8.490956241	-4.568009224
H27	6.436678286	8.445876636	-5.654424941
H28	7.510697429	8.773672111	-4.262700479
H29	6.232099304	7.530264212	-4.13207437
S30	5.370613836	9.718545941	-3.983262054
O31	5.504251039	9.732788583	-2.524560383
O32	4.041306142	9.285300156	-4.420585962
O33	5.782345672	10.98132586	-4.597554145

Geometry of I-conf2

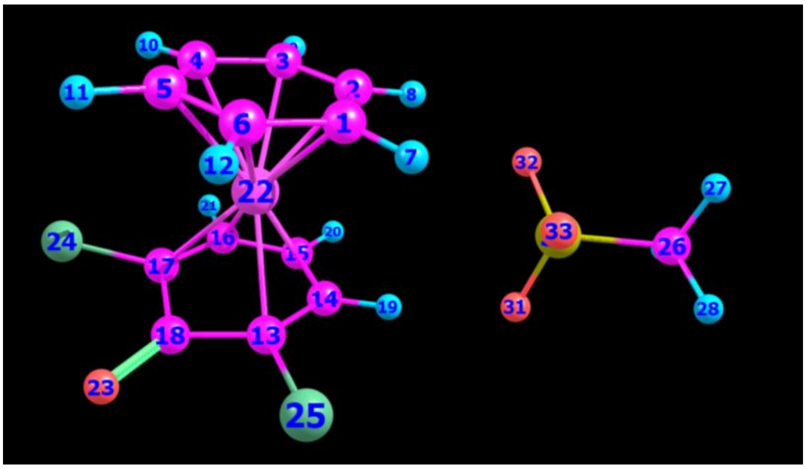

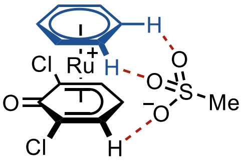

I-conf2

Fig S145: Structure of **I-conf2**. Left: With numbering of atoms respective to table S65.

Table S65 XYZ-coordinates for **I-conf2**:

Atom	X-Coordinate	Y-Coordinate	Z-Coordinate
C1	1.732099571	11.59112942	-1.372133053
C2	3.120761013	11.8416401	-1.365990646
C3	3.595498103	13.16199659	-1.220762288
C4	2.680204888	14.22700526	-1.086763794
C5	1.28828445	13.97197014	-1.060038001
C6	0.815910834	12.6556199	-1.201503722
H7	1.38380438	10.58562393	-1.58140802
H8	3.800084292	11.02126889	-1.567890671
H9	4.655345894	13.36922973	-1.291928299
H10	3.040096511	15.24728617	-1.06000686

H11	0.589669172	14.79729254	-1.013919542
H12	-0.248100775	12.46801553	-1.267512111
C13	0.913381007	12.47606676	-4.640706468
C14	2.215232461	11.95628729	-4.776670597
C15	3.317782341	12.8350495	-4.737928424
C16	3.098882293	14.2153924	-4.548397317
C17	1.782396507	14.69905846	-4.416890359
C18	0.590089838	13.90552437	-4.74563166
H19	2.371608056	10.88038397	-4.81588668
H20	4.325697092	12.44372706	-4.774910727
H21	3.934696646	14.89246243	-4.425093947
Ru22	2.154549786	13.10946121	-2.908504062
O23	-0.531273267	14.35969979	-4.885538715
Cl24	1.541029581	16.37404231	-4.130036219
Cl25	-0.4122717	11.38467408	-4.628798495
C26	3.284875023	6.666147486	-3.293131944
H27	3.403241492	6.306976755	-2.271426398
H28	2.449841149	6.160908656	-3.777161781
H29	4.202386374	6.507209078	-3.858565731
S30	2.94409006	8.402438469	-3.244483839
O31	2.794817839	8.824657331	-4.639774814
O32	4.095762245	9.025634685	-2.590218275
O33	1.707175972	8.553647395	-2.476539489

Geometry of I-conf3

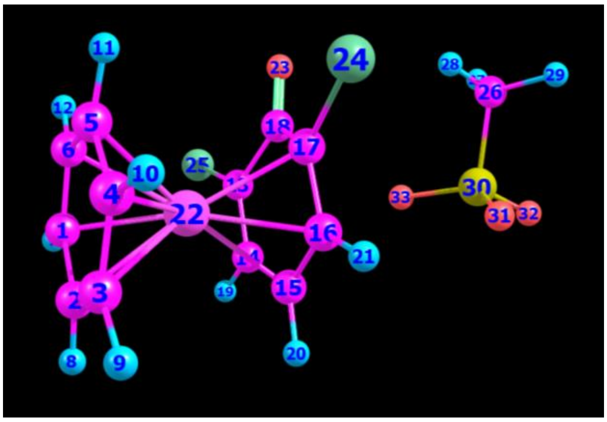

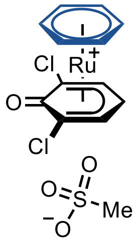

I-conf3

Fig S146: Structure of **I-conf3**. Left: With numbering of atoms respective to table S66.

Table S66 XYZ-coordinates for **I-conf3**:

Atom	X-Coordinate	Y-Coordinate	Z-Coordinate
C1	2.210084566	11.47546684	-1.165497553
C2	3.515531244	11.98771706	-1.314676828
C3	3.729816618	13.38190305	-1.288297526
C4	2.637923498	14.25708446	-1.110762608
C5	1.333327475	13.74057663	-0.925248544
C6	1.119366374	12.35209028	-0.952681299
H7	2.0335506	10.4126458	-1.267378015
H8	4.338233904	11.31667579	-1.524098432
H9	4.717298588	13.78231629	-1.47678047
H10	2.78960089	15.3269024	-1.16979554
H11	0.492075213	14.41689344	-0.846240577
H12	0.113438196	11.95728952	-0.893808432
C13	0.975482845	11.92738514	-4.385939949
C14	2.346450522	11.73753794	-4.637904346
C15	3.194916614	12.85762173	-4.745127679
C16	2.655762769	14.14846327	-4.583584262
C17	1.282475889	14.29829684	-4.321884905
C18	0.300026792	13.22484845	-4.523170919
H19	2.75441441	10.73522073	-4.66989771
H20	4.259719644	12.72414737	-4.880242397
H21	3.302696244	15.01530238	-4.57350448
Ru22	2.176779196	12.93777127	-2.809878309
O23	-0.905050604	13.38166463	-4.574452557
Cl24	0.661430827	15.87726205	-4.059665143
Cl25	-0.031009371	10.54825577	-4.204999804
C26	-0.228744843	15.16785646	-7.984779024
H27	-0.993060902	14.58352328	-8.496059817
H28	-0.474647994	15.26477252	-6.928074804
H29	-0.137229415	16.15229543	-8.442417869
S30	1.323059019	14.32411338	-8.134286883
O31	2.305095574	15.15832931	-7.44003701
O32	1.588187259	14.21997203	-9.570315845

O33	1.133412956	13.02020577	-7.498886179

Geometry of I-conf4

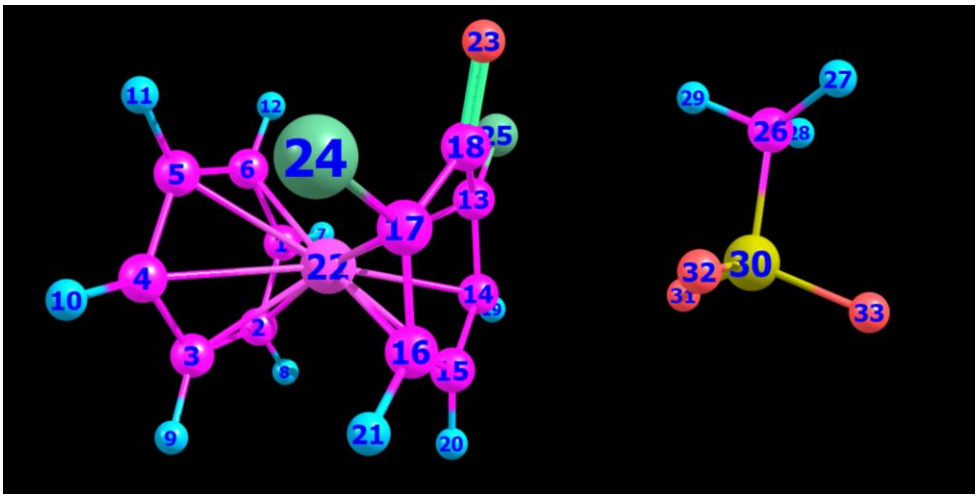

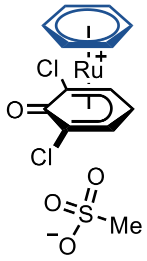

I-conf4

Fig S147: Structure of **I-conf4**. Left: With numbering of atoms respective to table S67.

Table S67 XYZ-coordinates for **I-conf4**:

Atom	X-Coordinate	Y-Coordinate	Z-Coordinate
C1	1.935067489	11.42814819	-1.387736851
C2	3.315604766	11.70895548	-1.461092897
C3	3.779347149	13.0199633	-1.223078218
C4	2.860076306	14.04483388	-0.917754122
C5	1.478470181	13.75629383	-0.811023998
C6	1.017541652	12.44961765	-1.044778881
H7	1.570808767	10.44230645	-1.645515148
H8	4.00778153	10.93663688	-1.769962297
H9	4.827985565	13.25546447	-1.349315304
H10	3.205217422	15.06562643	-0.817778523
H11	0.772212178	14.5560684	-0.629883
H12	-0.044718278	12.24215842	-1.045712078
C13	0.833568296	12.63127256	-4.474177307
C14	2.118097668	12.14745871	-4.778432543
C15	3.218044871	13.0255216	-4.737437482
C16	3.010636916	14.3748327	-4.38829866
C17	1.709304601	14.82498911	-4.100665245
C18	0.498347952	14.06057399	-4.428138257

H19	2.266483354	11.09099258	-4.956482183
H20	4.219875923	12.65286055	-4.902650226
H21	3.851767879	15.04506906	-4.264192992
Ru22	2.212063962	13.09620867	-2.794945317
O23	-0.630641042	14.51328155	-4.440279305
Cl24	1.486155119	16.45941754	-3.62511918
Cl25	-0.481447592	11.52755924	-4.463700632
C26	-0.710636087	13.25322842	-8.177118862
H27	-1.083315817	14.21519972	-8.527473321
H28	-1.065900927	12.45233196	-8.824660803
H29	-1.030611864	13.07759701	-7.15083506
S30	1.061186769	13.28018076	-8.226289614
O31	1.497862036	11.96640093	-7.7511604
O32	1.471427231	14.36982882	-7.341044919
O33	1.421533553	13.52157985	-9.624546345

Geometry of I-conf5

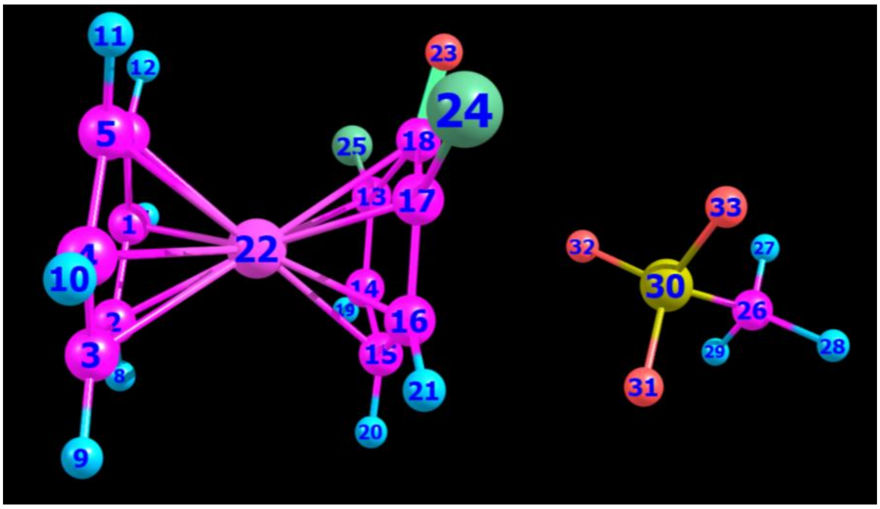

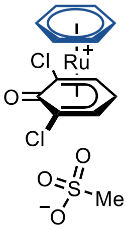

I-conf5

Fig S148: Structure of **I-conf5**. Left: With numbering of atoms respective to table S68.

Table S68 XYZ-coordinates for **I-conf5**:

**Atom**	**X-Coordinate**	**Y-Coordinate**	**Z-Coordinate**
C1	2.004252218	11.37602453	-1.248525874
C2	3.364429213	11.74745679	-1.258694889
C3	3.724972016	13.09755477	-1.064748389
C4	2.723282493	14.06858556	-0.85413436

C5	1.361146164	13.68820939	-0.807053815
C6	1.001006188	12.34449048	-1.005372947
H7	1.722842842	10.35712745	-1.480705649
H8	4.123050477	11.01173438	-1.491808377
H9	4.760877368	13.39935421	-1.147424331
H10	2.992675248	15.1141592	-0.781806683
H11	0.593933059	14.44452423	-0.703148574
H12	-0.043114771	12.06372	-1.052019481
C13	1.095106017	12.27046401	-4.474845796
C14	2.462897494	11.99524636	-4.656311189
C15	3.399936807	13.04413423	-4.576012082
C16	2.950246598	14.34995607	-4.301192741
C17	1.575771142	14.58745289	-4.118725945
C18	0.531334581	13.62684883	-4.501356734
H19	2.794425836	10.97184173	-4.778024227
H20	4.458497897	12.83777534	-4.656122617
H21	3.65993004	15.15243065	-4.148839207
Ru22	2.248236325	12.99750727	-2.71668363
O23	-0.651474292	13.88903744	-4.607780838
Cl24	1.058460352	16.17284832	-3.713101332
Cl25	-0.022871835	10.96883428	-4.523893431
C26	2.168973225	14.79768648	-9.651437816
H27	1.168862616	14.68075771	-10.06761641
H28	2.6036453	15.74295658	-9.974915774
H29	2.803832905	13.96684554	-9.95763418
S30	2.051272089	14.80081327	-7.882179388
O31	3.419698268	14.96273202	-7.389049944
O32	1.463528347	13.51221357	-7.516101933
O33	1.189665162	15.93286568	-7.538225647

Geometry of I-conf6

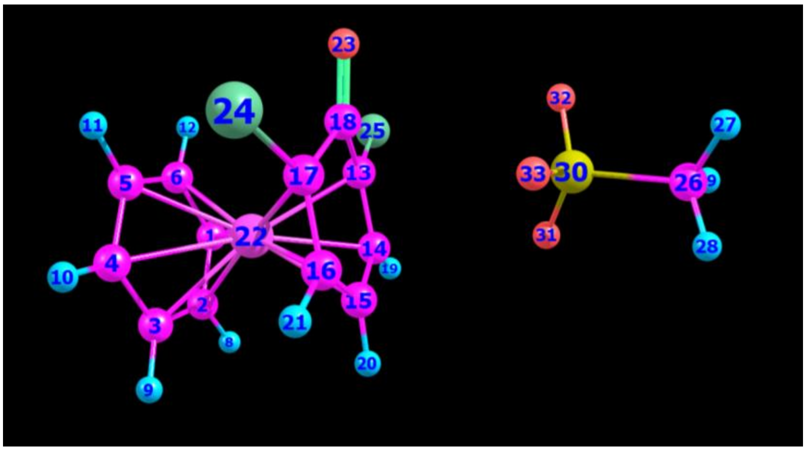

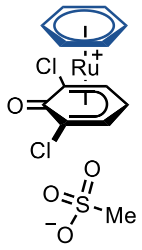

I-conf6

Fig S149: Structure of **I-conf6**. Left: With numbering of atoms respective to table S69.

Table S69 XYZ-coordinates for **I-conf6**:

Atom	X-Coordinate	Y-Coordinate	Z-Coordinate
C1	2.028070931	11.50707199	-1.123161243
C2	3.390928957	11.86405457	-1.181790274
C3	3.765182954	13.22173115	-1.096799676
C4	2.774819962	14.21532464	-0.947104517
C5	1.410452481	13.85266852	-0.851079805
C6	1.036816188	12.50099524	-0.940720569
H7	1.7344012	10.47620632	-1.272383158
H8	4.139990968	11.10578674	-1.368915619
H9	4.802137859	13.5067467	-1.217340838
H10	3.054057761	15.26074835	-0.959630553
H11	0.651043679	14.62169448	-0.793964274
H12	-0.010168867	12.22722049	-0.951548943
C13	1.058805259	12.17096201	-4.386673135
C14	2.414240881	11.84605078	-4.575469871
C15	3.380234973	12.87095053	-4.589786589
C16	2.970172881	14.20706372	-4.410926205
C17	1.604886452	14.49612122	-4.232357888
C18	0.528909702	13.53555249	-4.512337482
H19	2.716292998	10.80831615	-4.624161744
H20	4.431321656	12.62830341	-4.668437926
H21	3.703055321	14.99984855	-4.330920238
Ru22	2.262900388	13.00823118	-2.714709171

O23	-0.649116826	13.82016036	-4.615761858
Cl24	1.13381448	16.12442642	-3.962472979
Cl25	-0.089686024	10.89857131	-4.303639808
C26	1.628561613	12.65476755	-9.849391451
H27	0.968572967	13.434474	-10.22828787
H28	2.670179636	12.94490116	-9.983460772
H29	1.431116462	11.71279194	-10.36008551
S30	1.317555488	12.43931966	-8.117099469
O31	2.230234272	11.38417984	-7.673830608
O32	-0.089565935	12.05214104	-8.005457427
O33	1.598189376	13.73269723	-7.49395049

Geometry of I-conf7

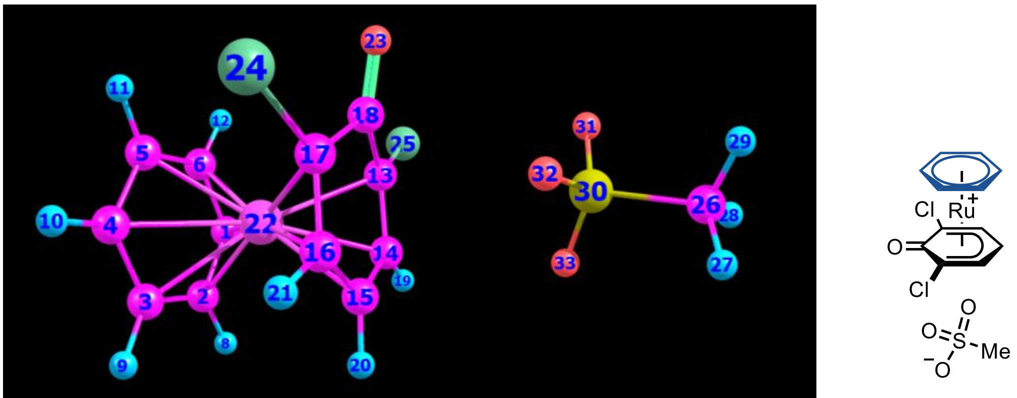

I-conf7

Fig S150: Structure of **I-conf7**. Left: With numbering of atoms respective to table S70.

Table S70 XYZ-coordinates for **I-conf7**:

Atom	X-Coordinate	Y-Coordinate	Z-Coordinate
C1	2.041251338	11.51118933	-1.106967386
C2	3.399671977	11.885803	-1.165716516
C3	3.755967644	13.24880161	-1.089729023
C4	2.752155344	14.23038588	-0.953697698
C5	1.391974716	13.85118698	-0.858877083
C6	1.03694588	12.49377762	-0.935918646
H7	1.761434973	10.47518743	-1.246712582
H8	4.159244745	11.13589146	-1.343912277
H9	4.789494287	13.54643861	-1.209165632
H10	3.017811192	15.27920171	-0.97601714

H11	0.622408842	14.61060958	-0.810921204
H12	-0.00635103	12.20617455	-0.947663689
C13	1.042040428	12.16697311	-4.373881931
C14	2.391231712	11.81347817	-4.557313933
C15	3.376900934	12.81935919	-4.583718392
C16	2.992240945	14.1651632	-4.42138986
C17	1.632774733	14.48289955	-4.247256556
C18	0.538598012	13.54007137	-4.516412524
H19	2.67323844	10.76945575	-4.592959113
H20	4.423162472	12.55573286	-4.658837813
H21	3.740324914	14.94446916	-4.350381836
Ru22	2.261600604	13.00047439	-2.711377198
O23	-0.633705392	13.8455903	-4.623636658
Cl24	1.193138587	16.12315279	-3.997560279
Cl25	-0.131367966	10.91867454	-4.274864696
C26	1.608327194	12.69290369	-9.861175677
H27	2.629625787	13.04055985	-10.01353821
H28	1.450666281	11.74801494	-10.38023965
H29	0.899392818	13.44043107	-10.2157737
S30	1.34738245	12.4399158	-8.125766359
O31	-0.034072546	11.97588397	-7.989689064
O32	1.57221731	13.73881825	-7.491466057
O33	2.324843721	11.43014059	-7.716039601

Geometry of I-conf8

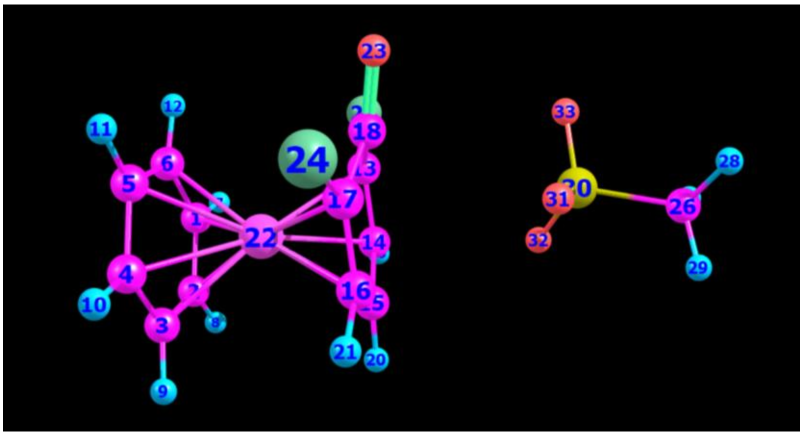

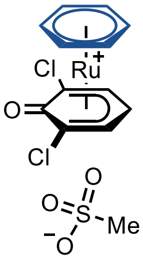

I-conf8

Fig S151: Structure of **I-conf8**. Left: With numbering of atoms respective to table S71.

Table S71 XYZ-coordinates for **I-conf8**:

**Atom**	**X-Coordinate**	**Y-Coordinate**	**Z-Coordinate**
C1	2.016664178	11.51246723	-1.117283804
C2	3.382316159	11.85764514	-1.180655489
C3	3.768303542	13.21230604	-1.100517104
C4	2.786859794	14.2146916	-0.950510454
C5	1.419786871	13.86387576	-0.849557383
C6	1.03436562	12.5152264	-0.934675228
H7	1.71381876	10.48376535	-1.263102768
H8	4.124360803	11.092535	-1.36787558
H9	4.807285003	13.48817132	-1.224795592
H10	3.07492454	15.25765329	-0.966485936
H11	0.667116428	14.63948588	-0.792303727
H12	-0.014922011	12.25032514	-0.941775985
C13	1.046485292	12.17198631	-4.379554438
C14	2.399768177	11.83957873	-4.571283667
C15	3.370932346	12.85956805	-4.591192061
C16	2.968178197	14.19819984	-4.414759337
C17	1.60483857	14.49466265	-4.233102482
C18	0.523247393	13.53895553	-4.50826284
H19	2.696521808	10.8001689	-4.617875864
H20	4.420499371	12.61130687	-4.672402579
H21	3.705334346	14.98743169	-4.338923175
Ru22	2.259556907	13.00734497	-2.713470532
O23	-0.653521446	13.82910542	-4.609725283
Cl24	1.142864862	16.12607797	-3.966100918
Cl25	-0.108552387	10.90608006	-4.287404874
C26	1.626597351	12.67333789	-9.854735136
H27	1.484517072	11.72482785	-10.37166305
H28	0.914775749	13.41245105	-10.22094202
H29	2.646287179	13.03028974	-9.995862322
S30	1.346617586	12.42464042	-8.121648812
O31	1.550662741	13.72818751	-7.489802071
O32	2.328759889	11.42622572	-7.695618311

O33	-0.031823519	11.94763973	-8.000923234

Geometry of I-conf9

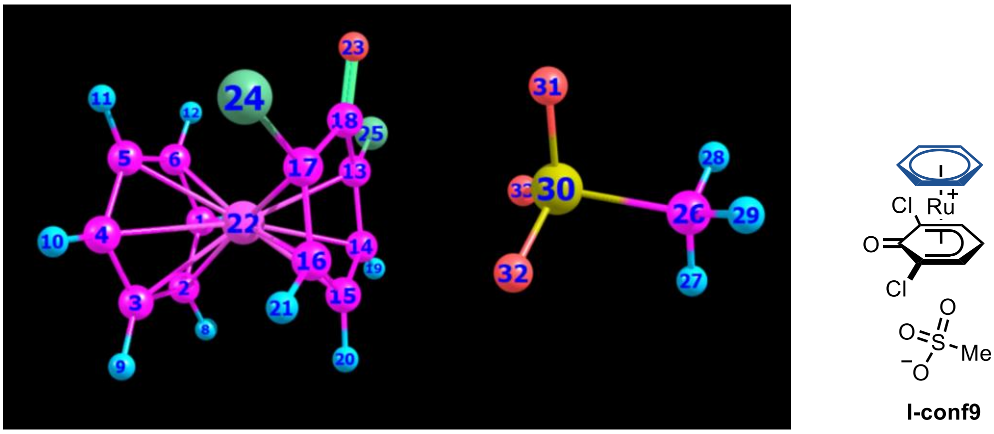

Fig S152: Structure of **I-conf9**. Left: With numbering of atoms respective to table S72.

Table S72 XYZ-coordinates for **I-conf9**:

Atom	X-Coordinate	Y-Coordinate	Z-Coordinate
C1	2.129098206	11.58795763	-0.971666453
C2	3.464258616	11.99588193	-1.170927506
C3	3.770361527	13.3689551	-1.277868416
C4	2.739965154	14.32755147	-1.181855647
C5	1.405748679	13.91875716	-0.946017113
C6	1.100242148	12.5512075	-0.841422466
H7	1.881856435	10.53438016	-0.972155943
H8	4.23901827	11.25465112	-1.316968695
H9	4.780702576	13.68296033	-1.505134218
H10	2.961205093	15.37463863	-1.342250617
H11	0.611578205	14.65387112	-0.930322815
H12	0.070830318	12.23167881	-0.74406742
C13	0.901905476	11.81036766	-4.216901333
C14	2.255547729	11.50949983	-4.453782985
C15	3.17362687	12.55659738	-4.66823942
C16	2.719843336	13.88924262	-4.629251105
C17	1.360758346	14.15319834	-4.380814141
C18	0.308848801	13.13097061	-4.46785934
H19	2.597770517	10.48421813	-4.392381529
H20	4.226457	12.3422668	-4.792544189

H21	3.421410048	14.709309	-4.704476473
Ru22	2.179387522	12.88677877	-2.747391184
O23	-0.884418536	13.36037924	-4.521110646
Cl24	0.846111311	15.78636546	-4.267551235
Cl25	-0.187774634	10.521283	-3.904880913
C26	1.439650708	13.49730546	-9.833976258
H27	2.293942333	12.84511413	-10.01284713
H28	0.52134242	13.01482101	-10.16720184
H29	1.575704456	14.44668848	-10.35089835
S30	1.317647047	13.80945331	-8.092801462
O31	0.158413035	14.68640677	-7.921689109
O32	2.574905591	14.45514826	-7.71125813
O33	1.136200411	12.50264327	-7.460832769

Geometry of I-conf10

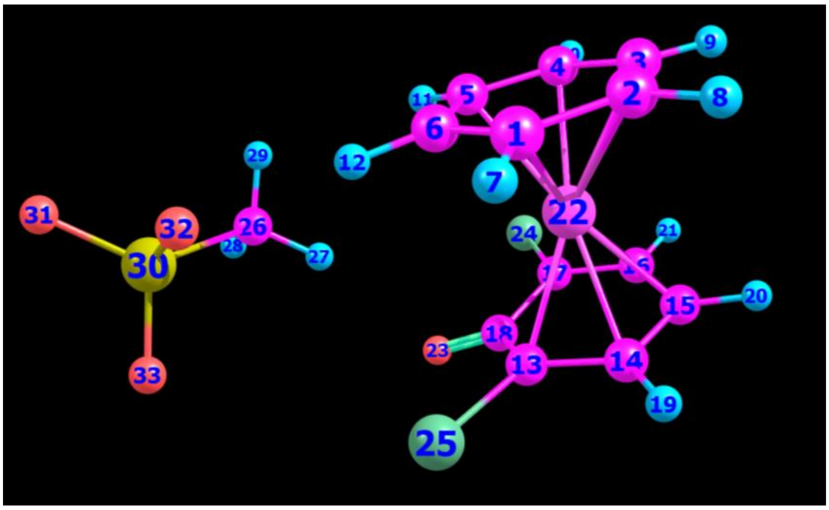

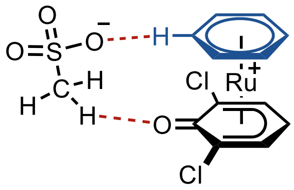

I-conf10

Fig S153: Structure of **I-conf10**. Left: With numbering of atoms respective to table S73.

Table S73 XYZ-coordinates for **I-conf10**:

Atom	X-Coordinate	Y-Coordinate	Z-Coordinate
C1	1.978221029	11.34767168	-1.484517112
C2	3.21220126	11.99135165	-1.256231965
C3	3.253894221	13.39294025	-1.102255885
C4	2.061455171	14.14233266	-1.177949519
C5	0.820983599	13.48960615	-1.371558469
C6	0.775672832	12.09311281	-1.524202479

H7	1.955995221	10.28416113	-1.684461531
H8	4.131790039	11.42105025	-1.274865232
H9	4.205002305	13.89944696	-1.002507877
H10	2.101936405	15.22314578	-1.141273086
H11	-0.081890004	14.07590832	-1.48324696
H12	-0.16280554	11.59382582	-1.75275271
C13	1.569912051	11.94407694	-4.898582827
C14	2.971165975	11.8732825	-4.771805307
C15	3.71541306	13.05650544	-4.583865078
C16	3.039146919	14.29144435	-4.503383157
C17	1.636552996	14.32594782	-4.631263529
C18	0.844535536	13.19875068	-5.143302897
H19	3.460308127	10.90788115	-4.74238202
H20	4.784269299	13.00870202	-4.424656641
H21	3.580352855	15.19853964	-4.265810658
Ru22	2.212253889	12.92372477	-3.004218848
O23	-0.308786135	13.27423292	-5.523275335
Cl24	0.825375608	15.82921135	-4.471826816
Cl25	0.678957721	10.4900384	-5.075346862
C26	-2.705095356	12.81827274	-2.982533102
H27	-1.799883092	12.93532497	-3.57802756
H28	-3.545933305	13.29760966	-3.48285921
H29	-2.567054728	13.24318728	-1.988518004
S30	-3.057930863	11.0904927	-2.816337968
O31	-4.289307047	10.99911417	-2.032707613
O32	-1.905018287	10.51114784	-2.118189834
O33	-3.207973672	10.57377802	-4.175997592

SPECTROSCOPIC DATA

^{1}H NMR of [η^6-benzene-η^5-(2,6-dibromo-4-methoxy-1-phenoxo)Ru](OTf) (1a)

CD_3OD, 600 MHz, 23 °C.

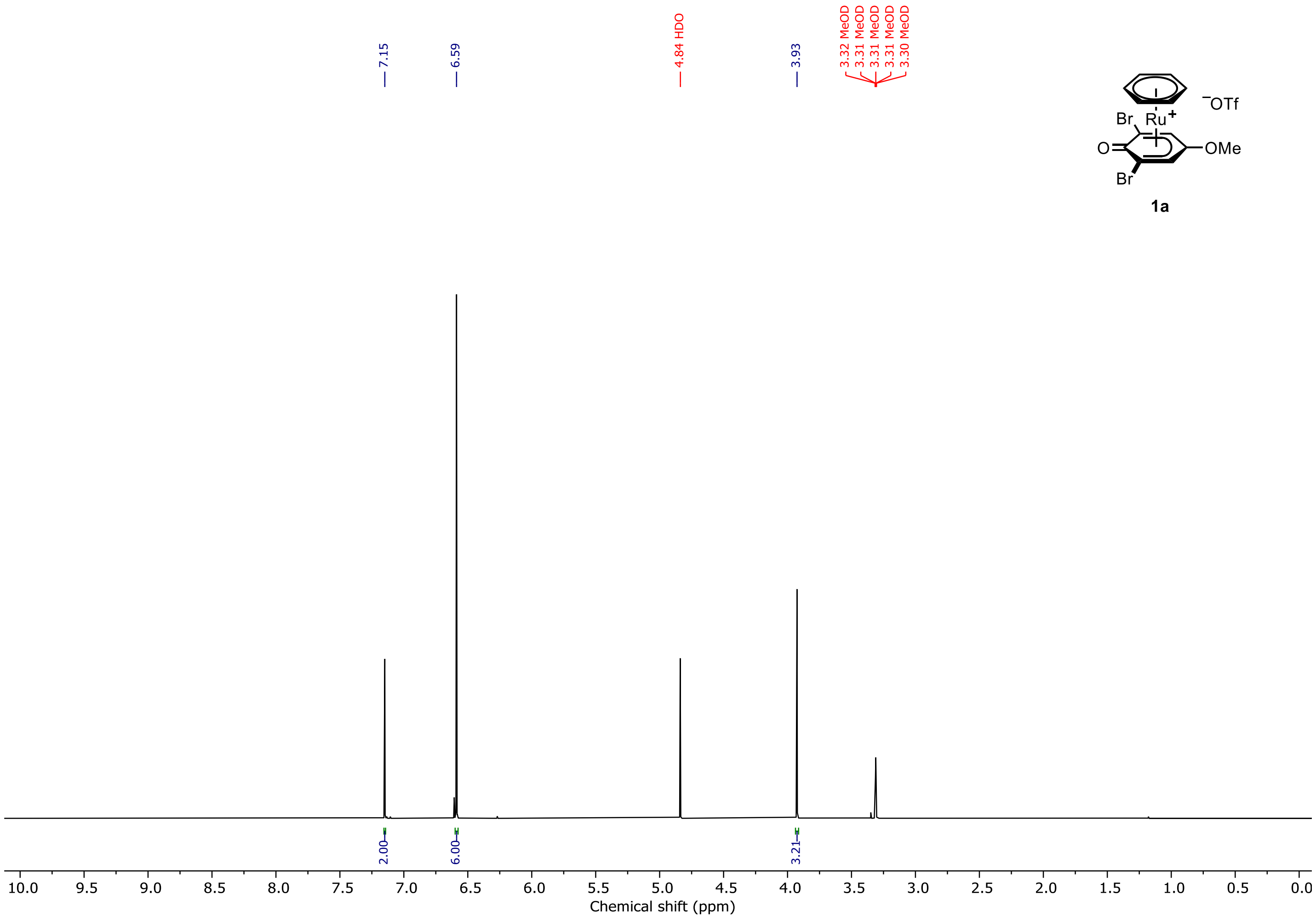

1a

^{13}C NMR of [η^6-benzene-η^5-(2,6-dibromo-4-methoxy-1-phenoxo)Ru](OTf) (1a)

CD_3OD, 151 MHz, 23 °C.

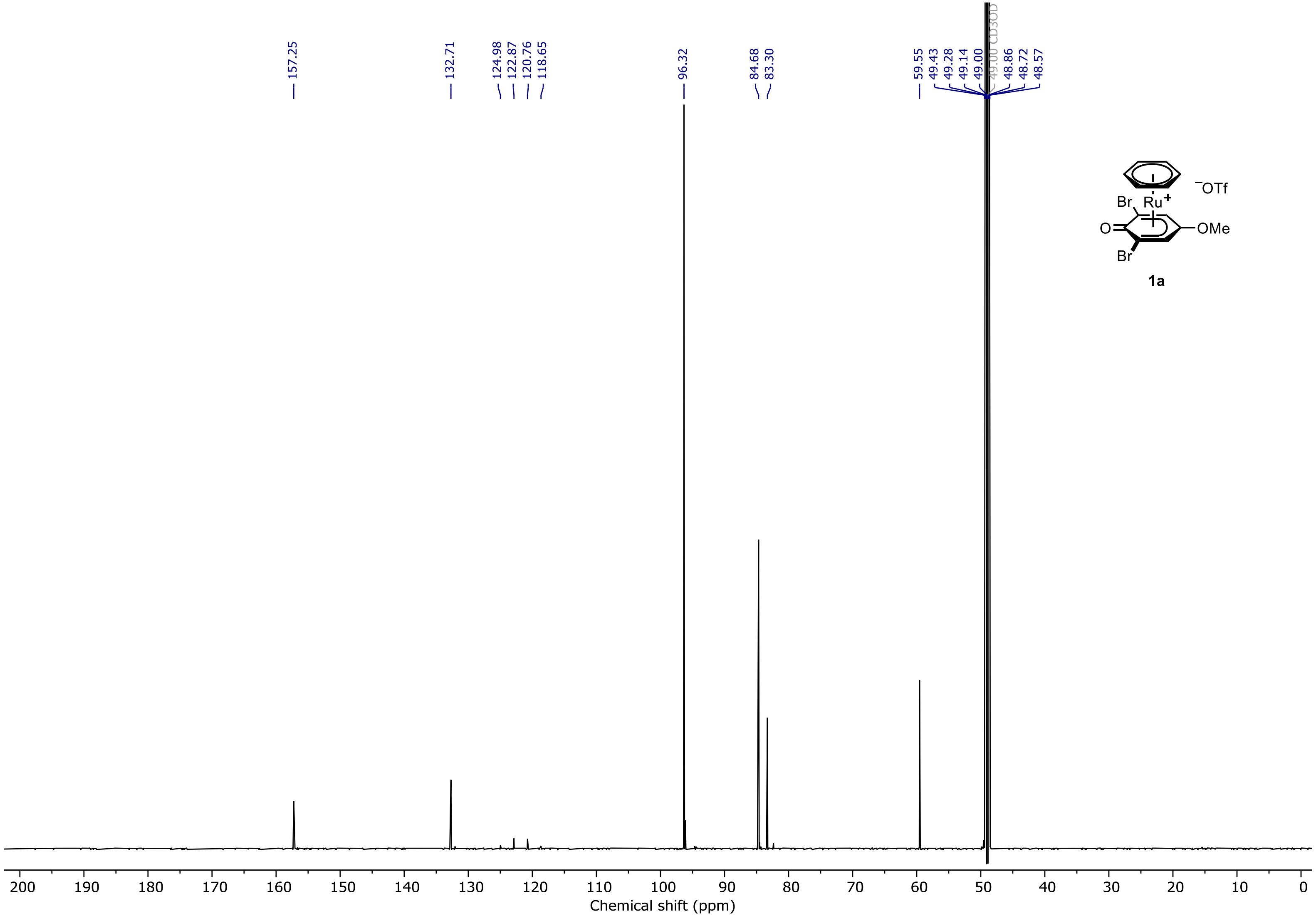

^{19}F NMR of [η^6-benzene-η^5-(2,6-dibromo-4-methoxy-1-phenoxo)Ru](OTf) (1a)

CD_3OD, 565 MHz, 23 °C.

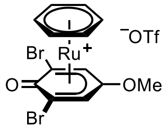

1a

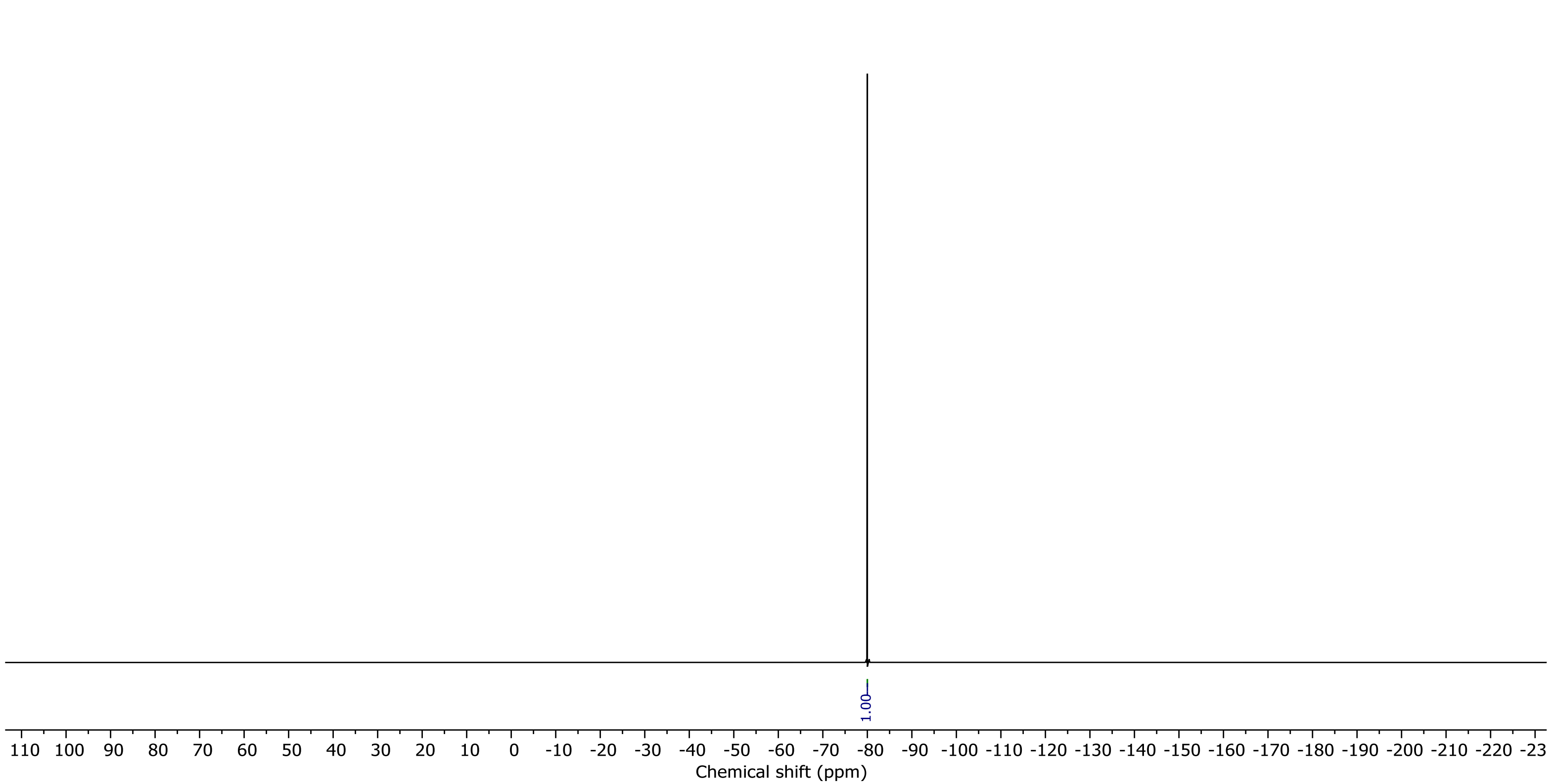

^{1}H NMR of [η^6-benzene-η^6-(4-methoxymethylanisole)Ru](OTf) (1b)

CD_3CN, 600 MHz, 23 °C.

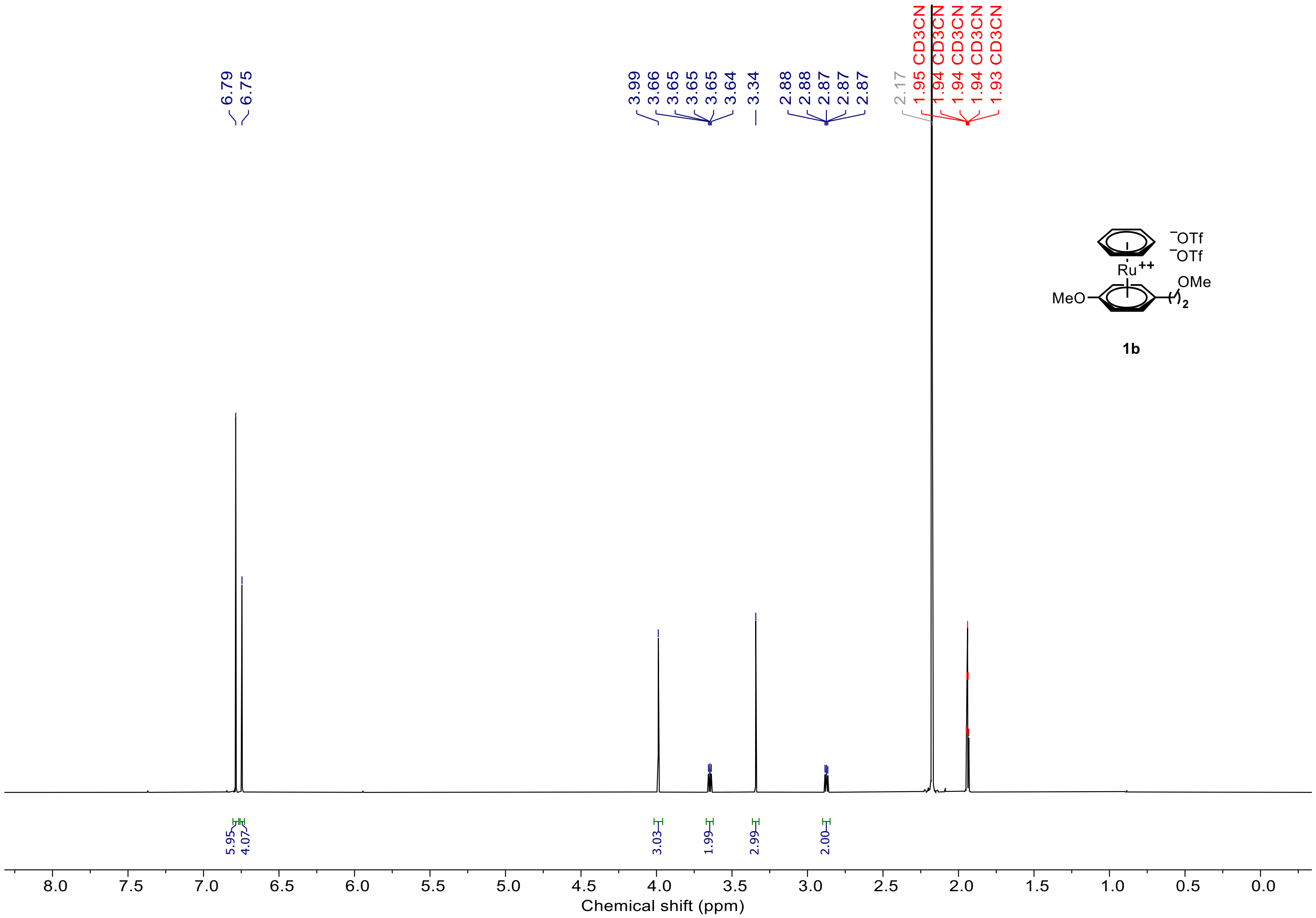

^{13}C NMR of [η^6-benzene-η^6-(4-methoxymethylanisole)Ru](OTf) (1b)

CD_3CN, 151 MHz, 23 °C.

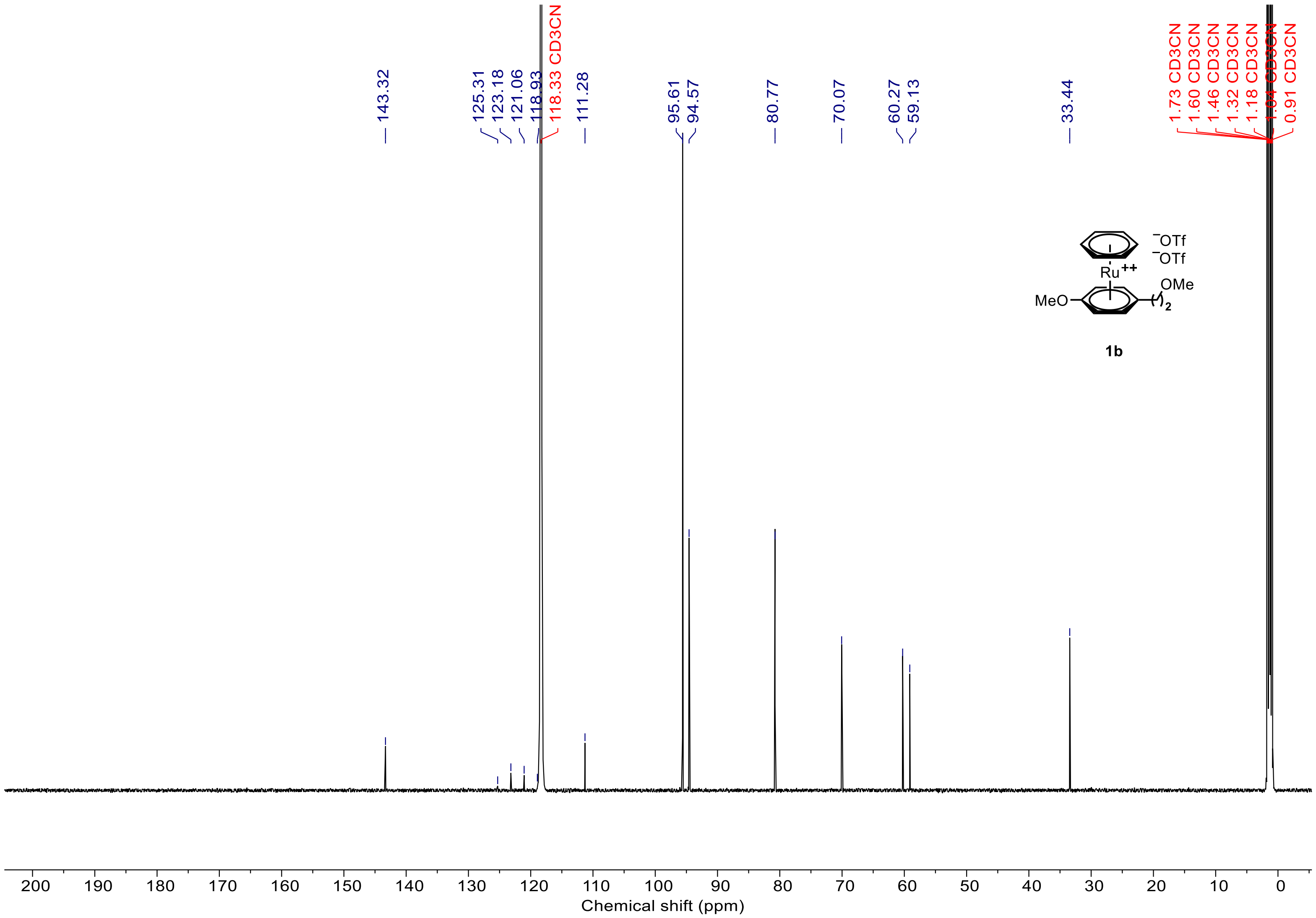

^{19}F NMR of [η^6-benzene-η^6-(4-methoxymethylanisole)Ru](OTf) (1b)

CD_3CN, 600 MHz, 23 °C.

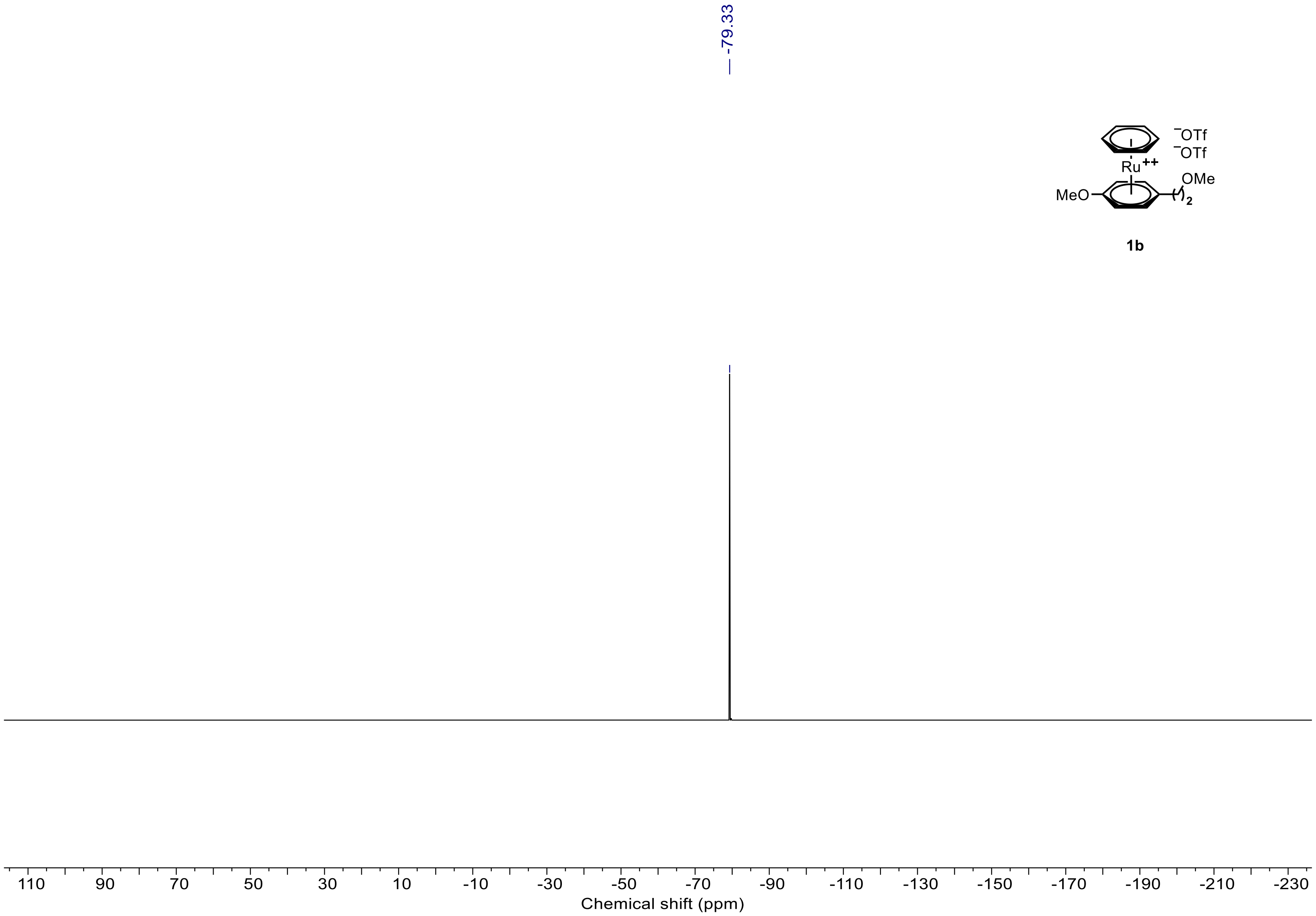

1b

^{1}H NMR of [η^6-benzene-η^5-(phenoxo)Ru](OTf) (1c)

CD_3OD, 600 MHz, 23 °C.

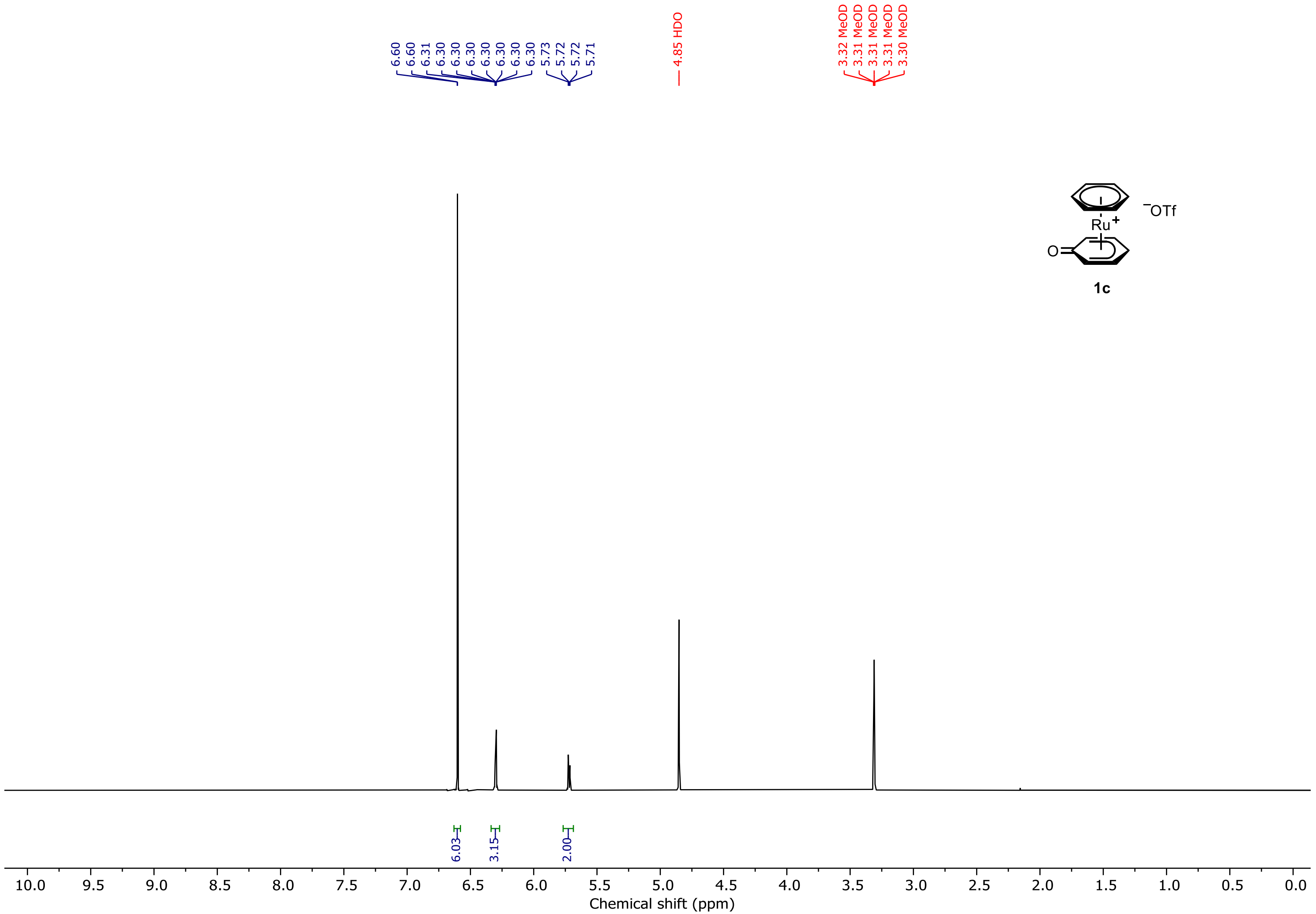

^{13}C NMR of [η^6-benzene-η^5-(phenoxo)Ru](OTf) (1c)

CD_3OD, 151 MHz, 23 °C.

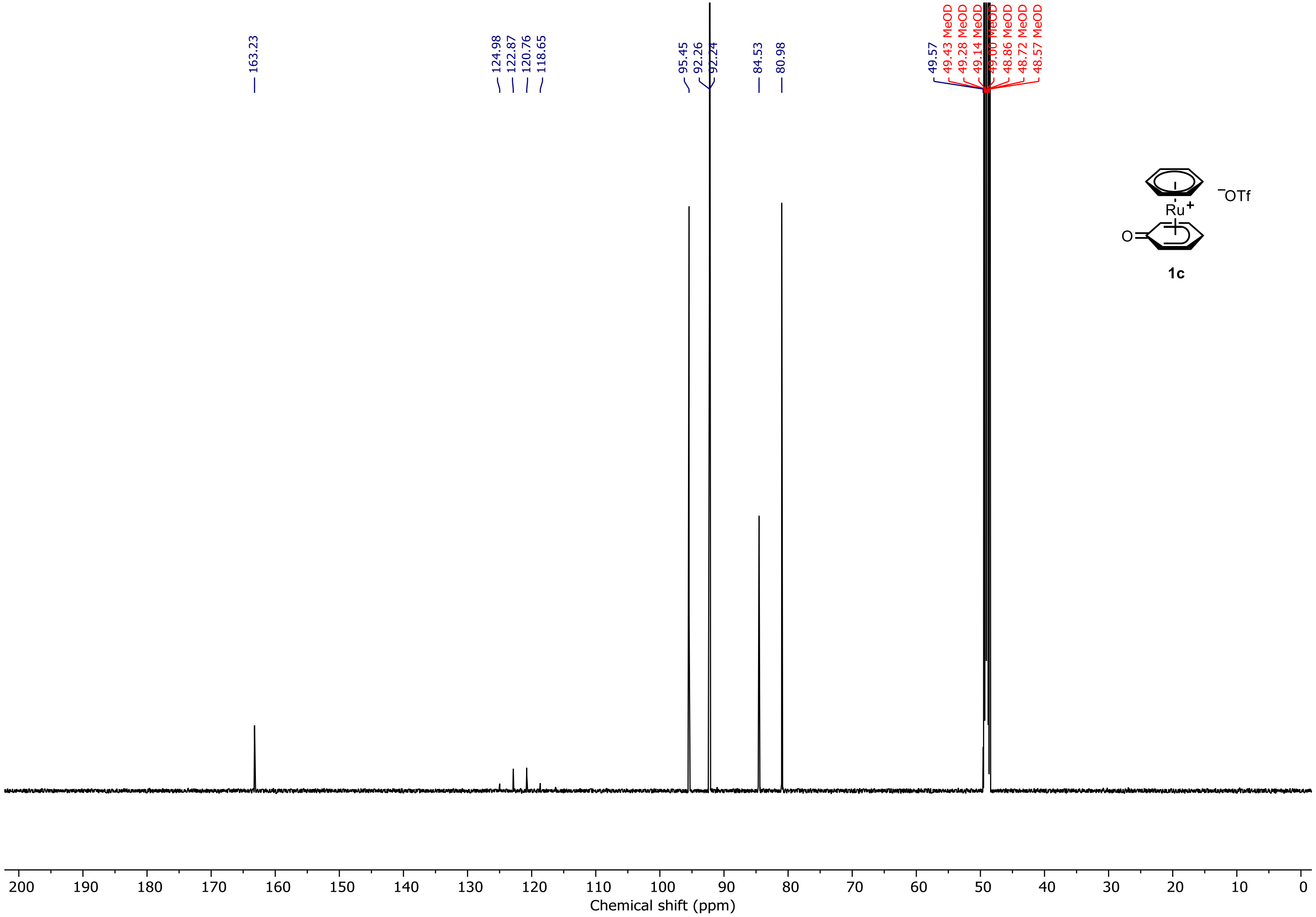

1c

^{19}F NMR of [η^6-benzene-η^5-(phenoxo)Ru](OTf) (1c)

CD_3OD, 600 MHz, 23 °C.

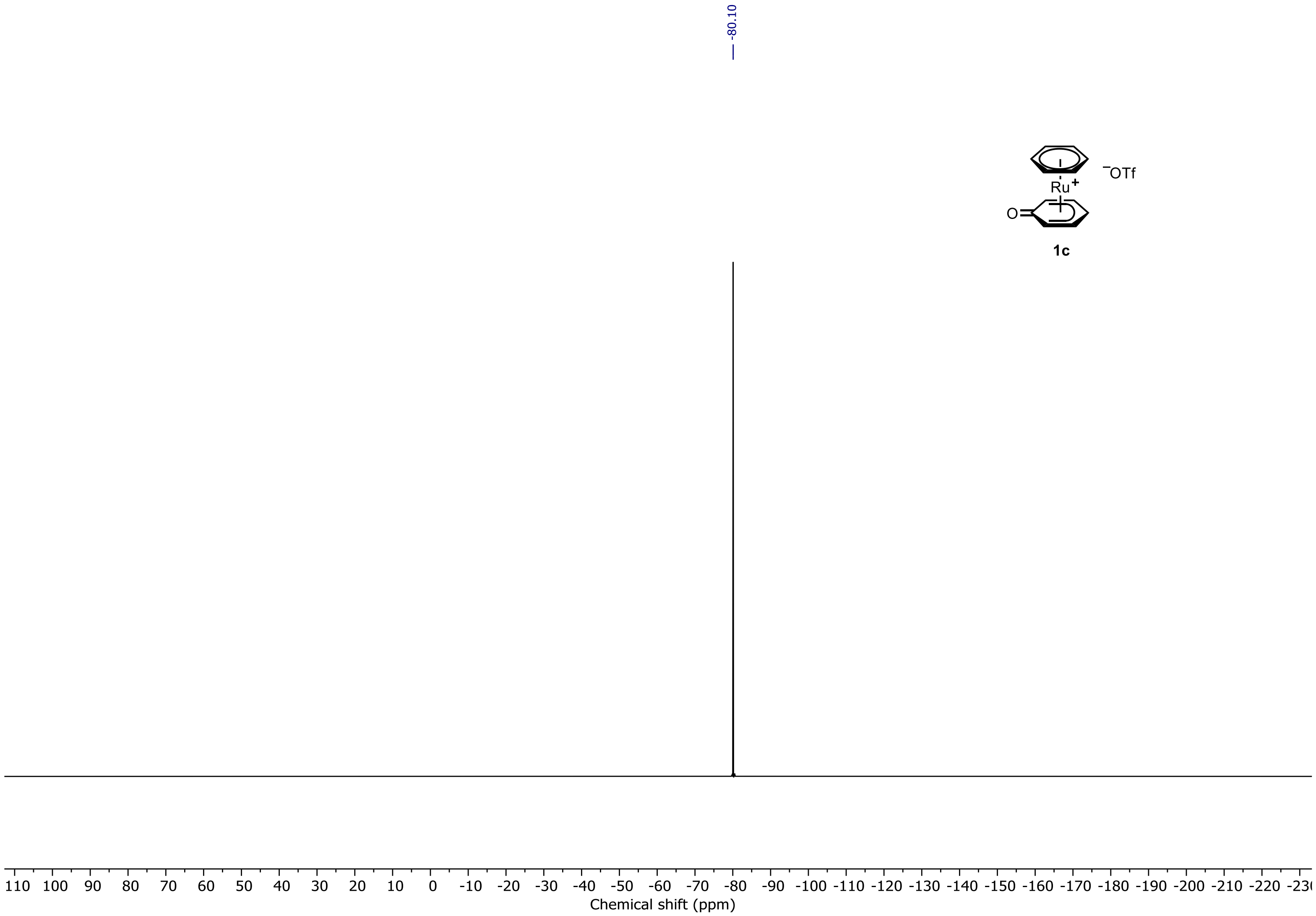

^{1}H NMR of [η^6-benzene-η^5-(4-butyl-1-phenoxo)Ru](OTf) (1d)

CD_3OD, 600 MHz, 23 °C.

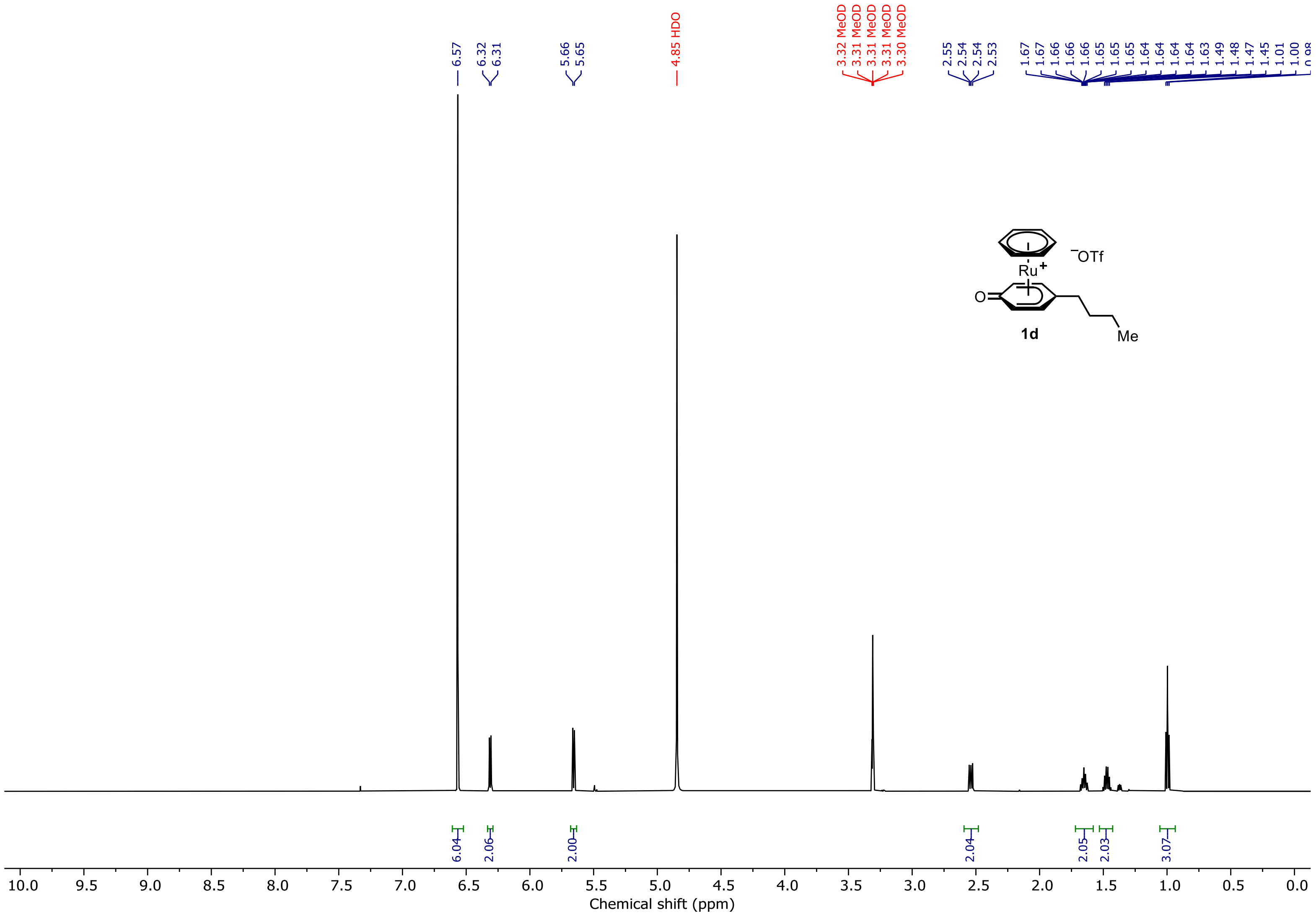

^{13}C NMR of [η^6-benzene-η^5-(4-butyl-1-phenoxo)Ru](OTf) (1d)

CD$_3$OD, 151 MHz, 23 °C.

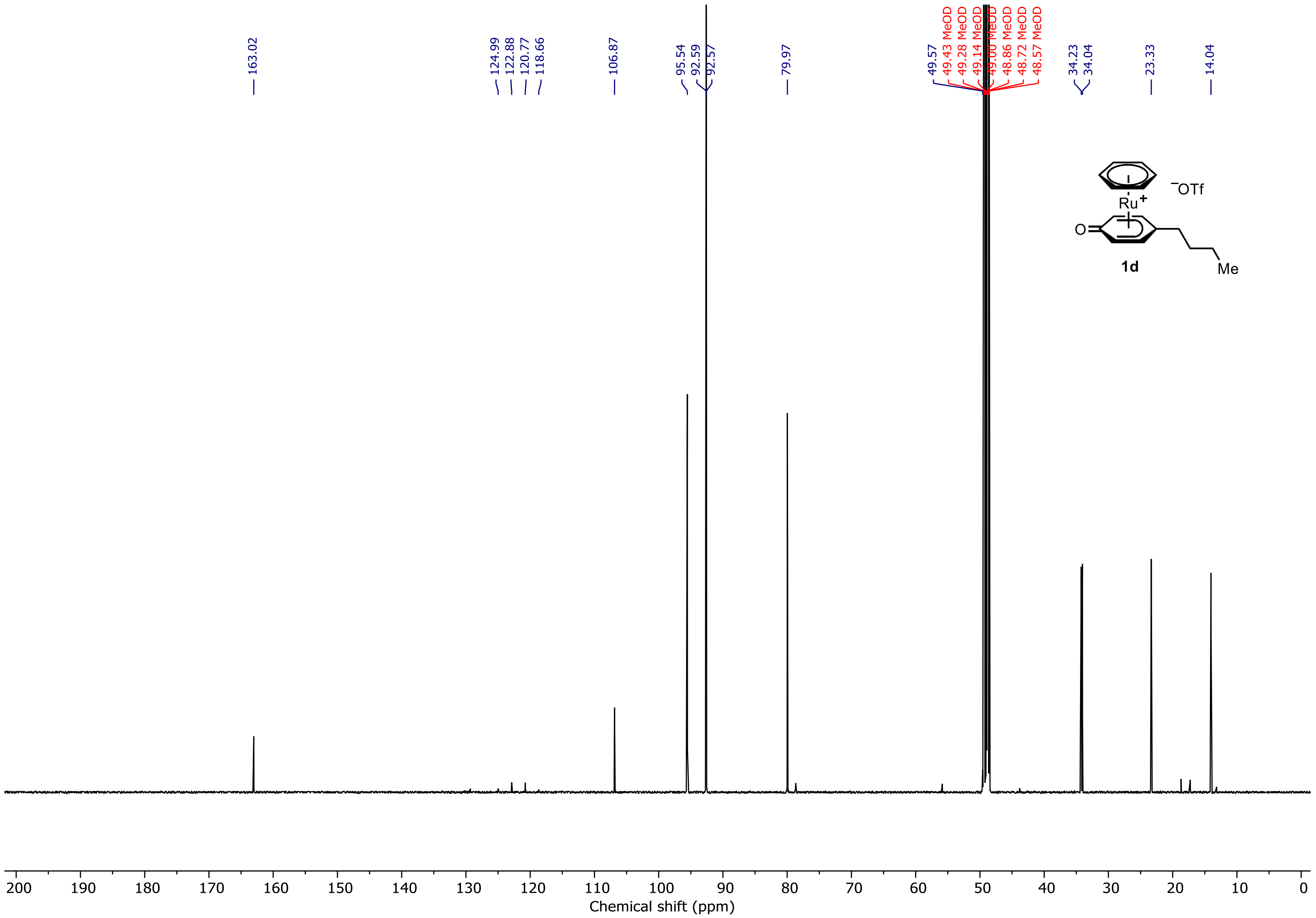

^{19}F NMR of [η^6-benzene-η^5-(4-butyl-1-phenoxo)Ru](OTf) (1d)

CD_3OD, 600 MHz, 23 °C.

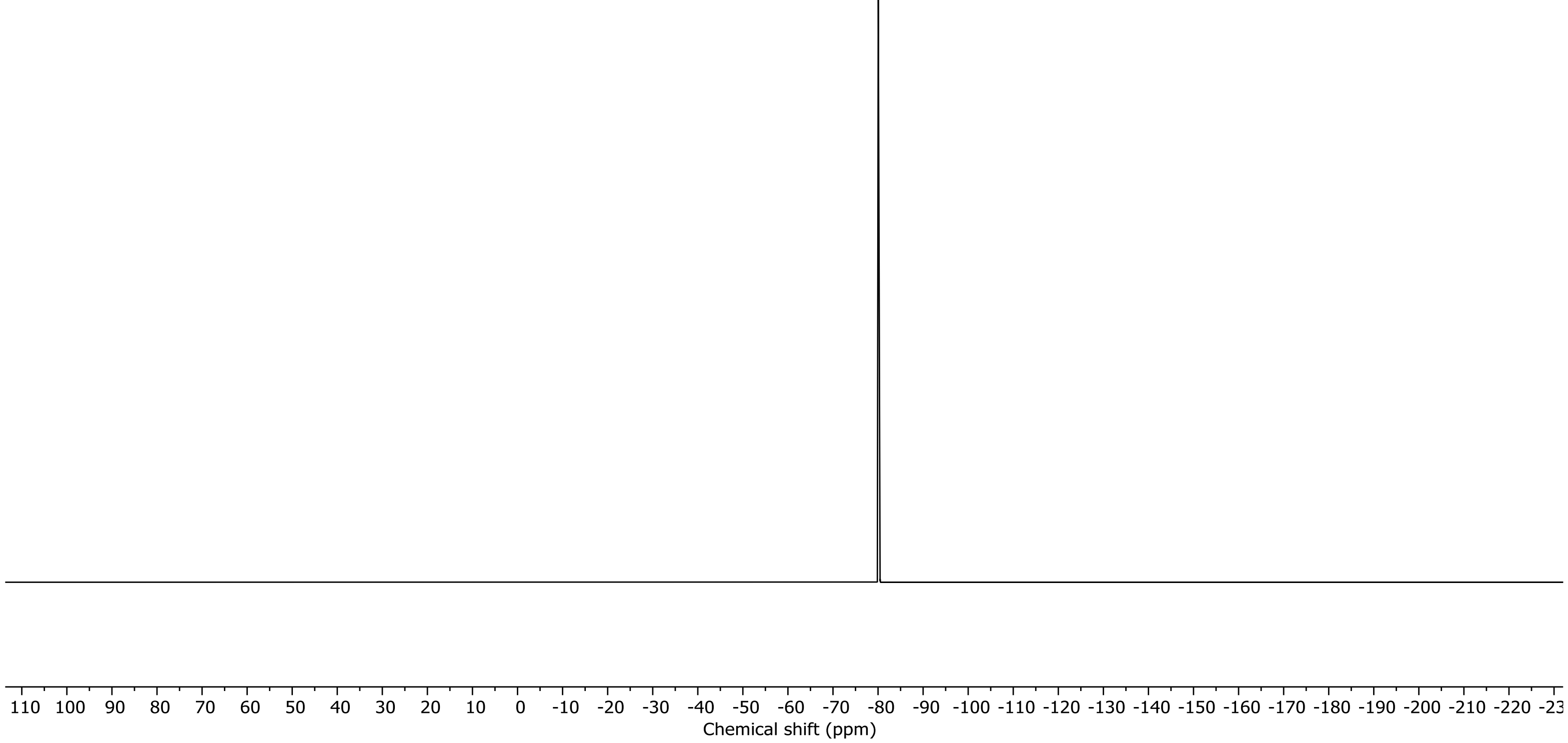

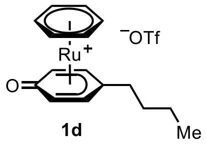

1d

^{1}H NMR of [*η*[6]-benzene-*η*[5]-(4-(2-methoxyethyl)-1-phenoxo)Ru](OTf) (1e)

CD_3OD, 600 MHz, 23 °C.

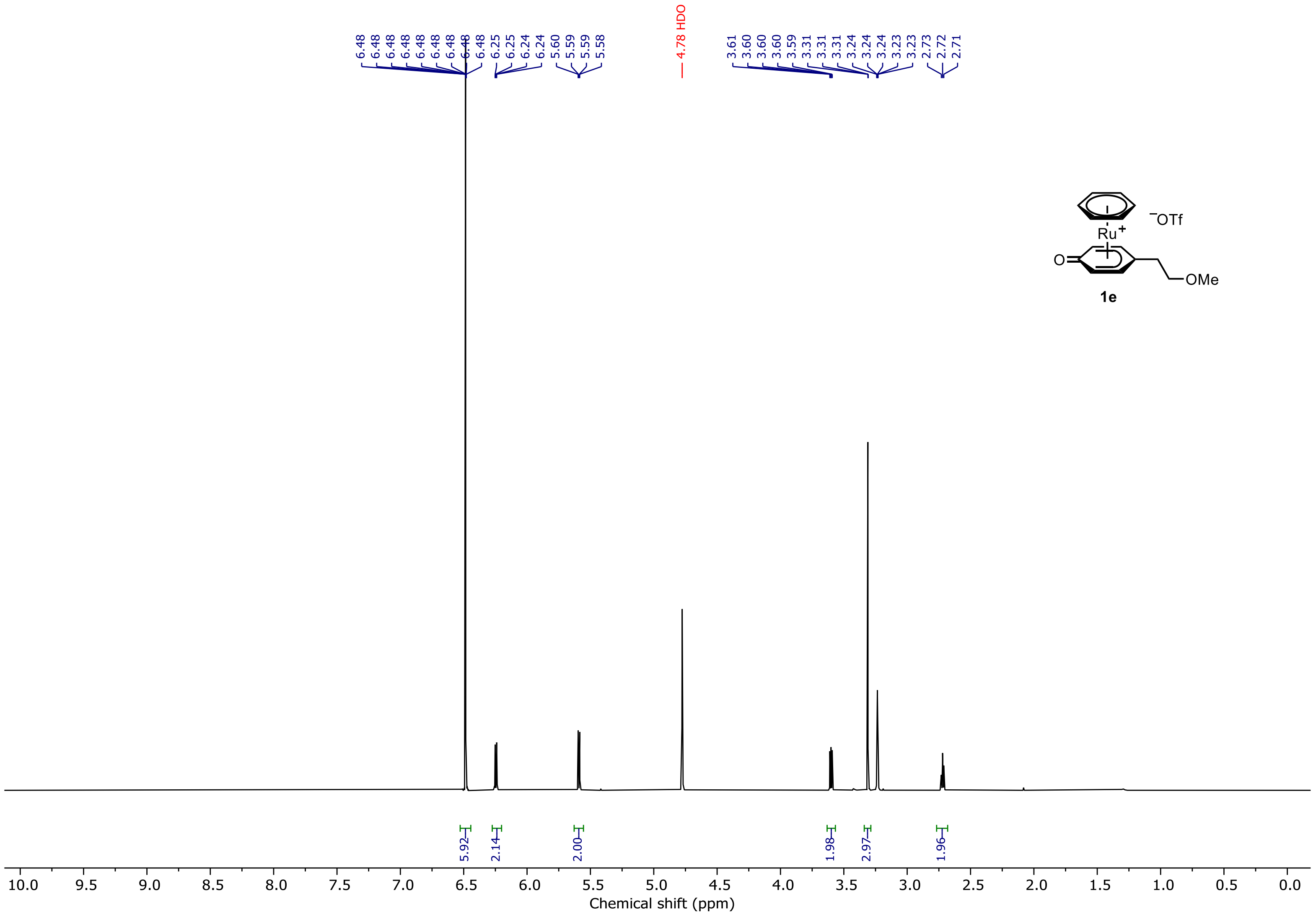

^{13}C NMR of [η^6-benzene-η^5-(4-(2-methoxyethyl)-1-phenoxo)Ru](OTf) (1e)

CD_3OD, 151 MHz, 23 °C.

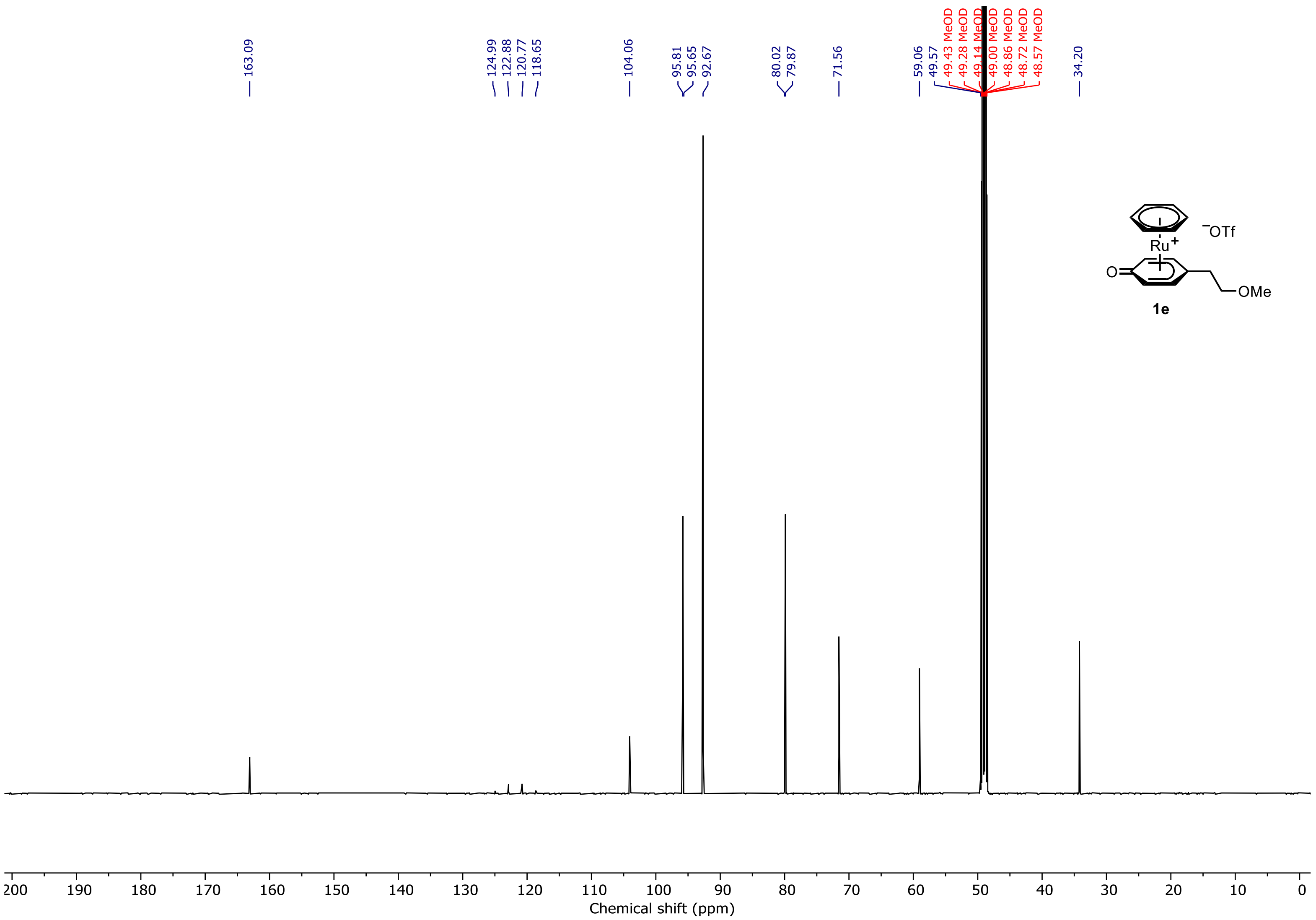

^{19}F NMR of [η^6-benzene-η^5-(4-(2-methoxyethyl)-1-phenoxo)Ru](OTf) (1e)

CD_3OD, 600 MHz, 23 °C.

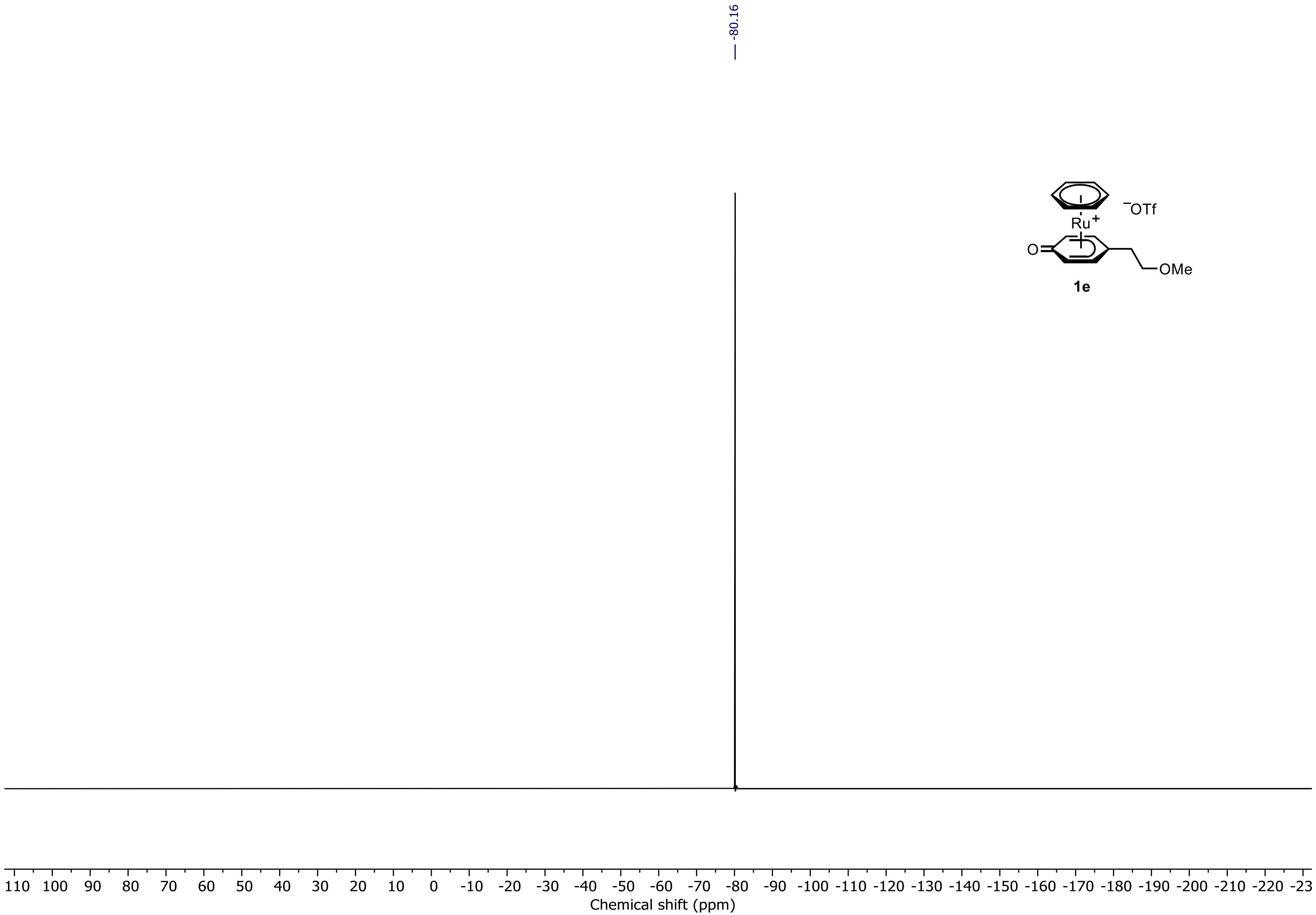

1e

^{1}H NMR of [η^6-benzene-η^5-(2,6-diiodo-1-phenoxo)Ru](OTf) (1f)

CD_3OD, 600 MHz, 23 °C.

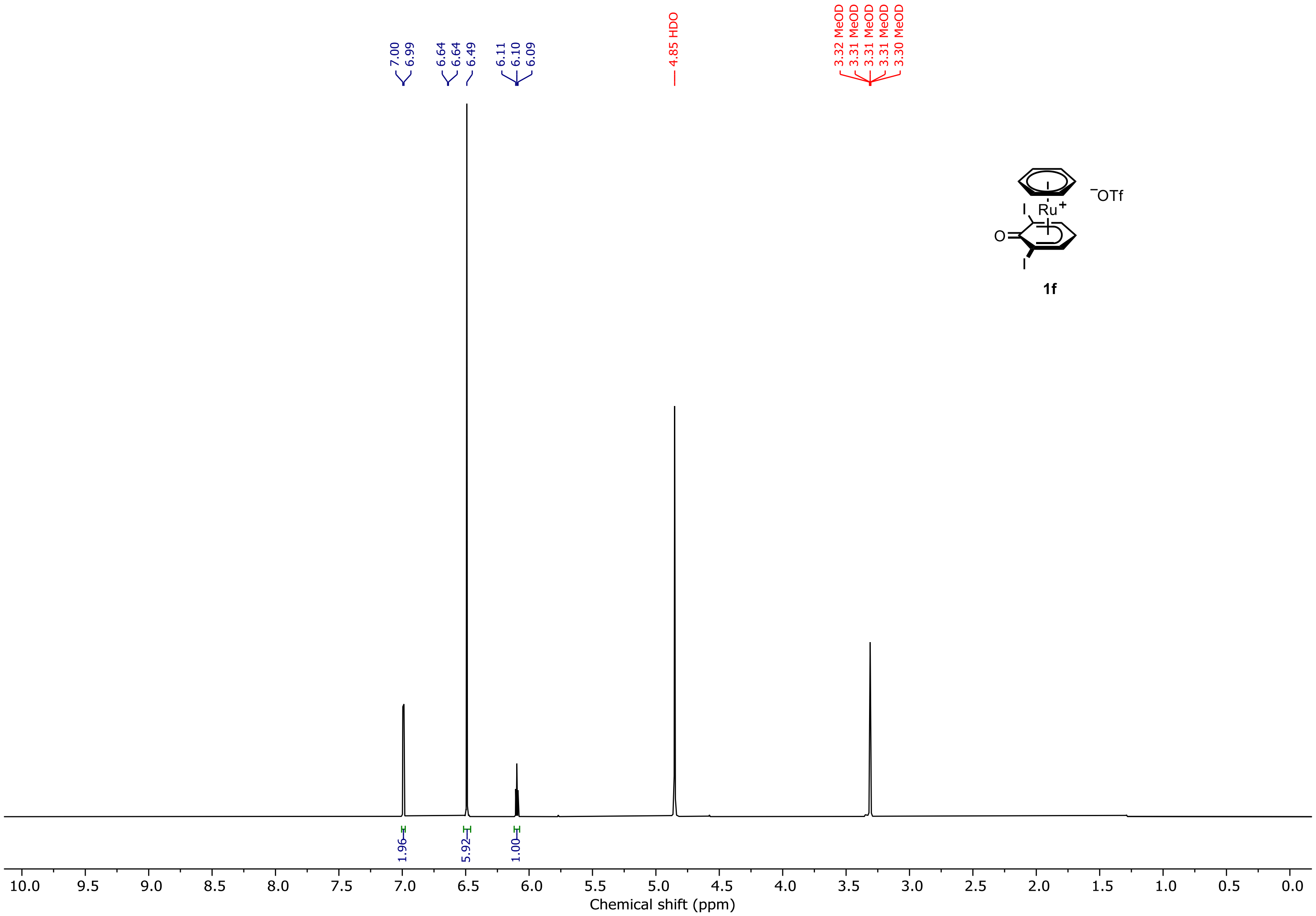

1f

^{13}C NMR of [η^6-benzene-η^5-(2,6-diiodo-1-phenoxo)Ru](OTf) (1f)

CD_3OD, 151 MHz, 23 °C.

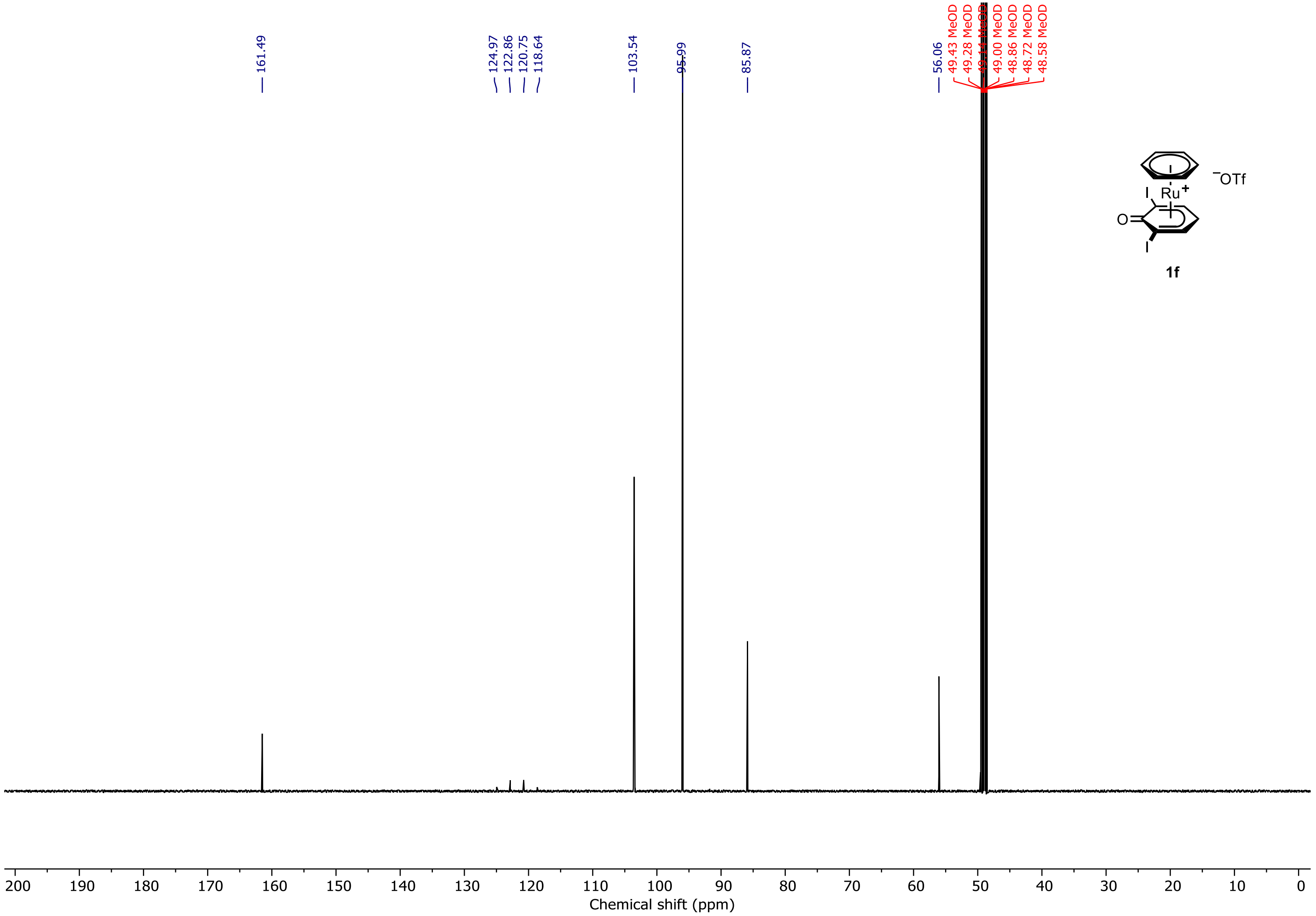

^{19}F NMR of [η^6-benzene-η^5-(2,6-dichloro-1-phenoxo)Ru](OTf) (1f)

CD_3OD, 565 MHz, 23 °C.

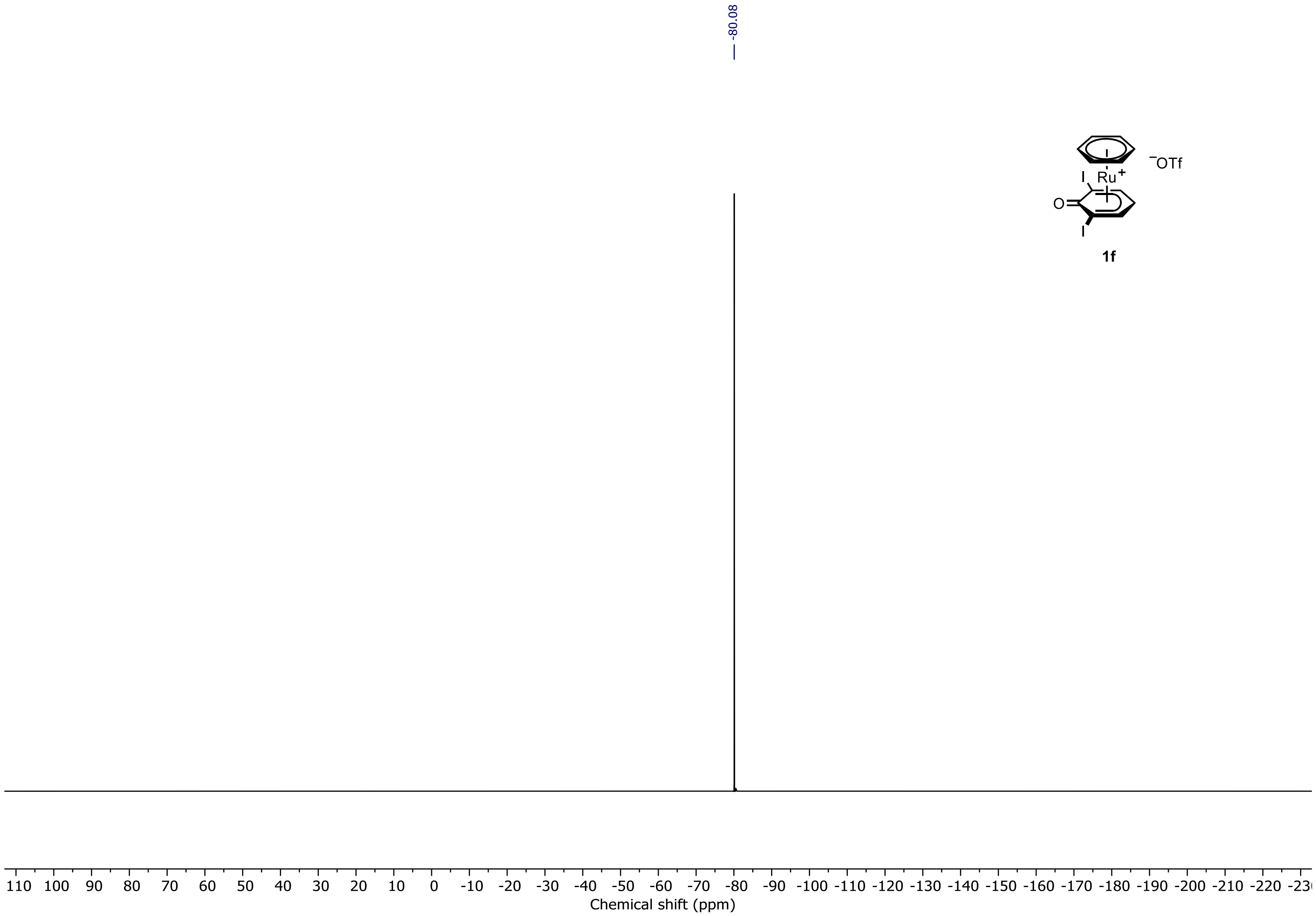

^{1}H NMR of [η^6-benzene-η^5-(2,6-dichloro-1-phenoxo)Ru](OTf) (1g)

CD_3OD, 600 MHz, 23 °C.

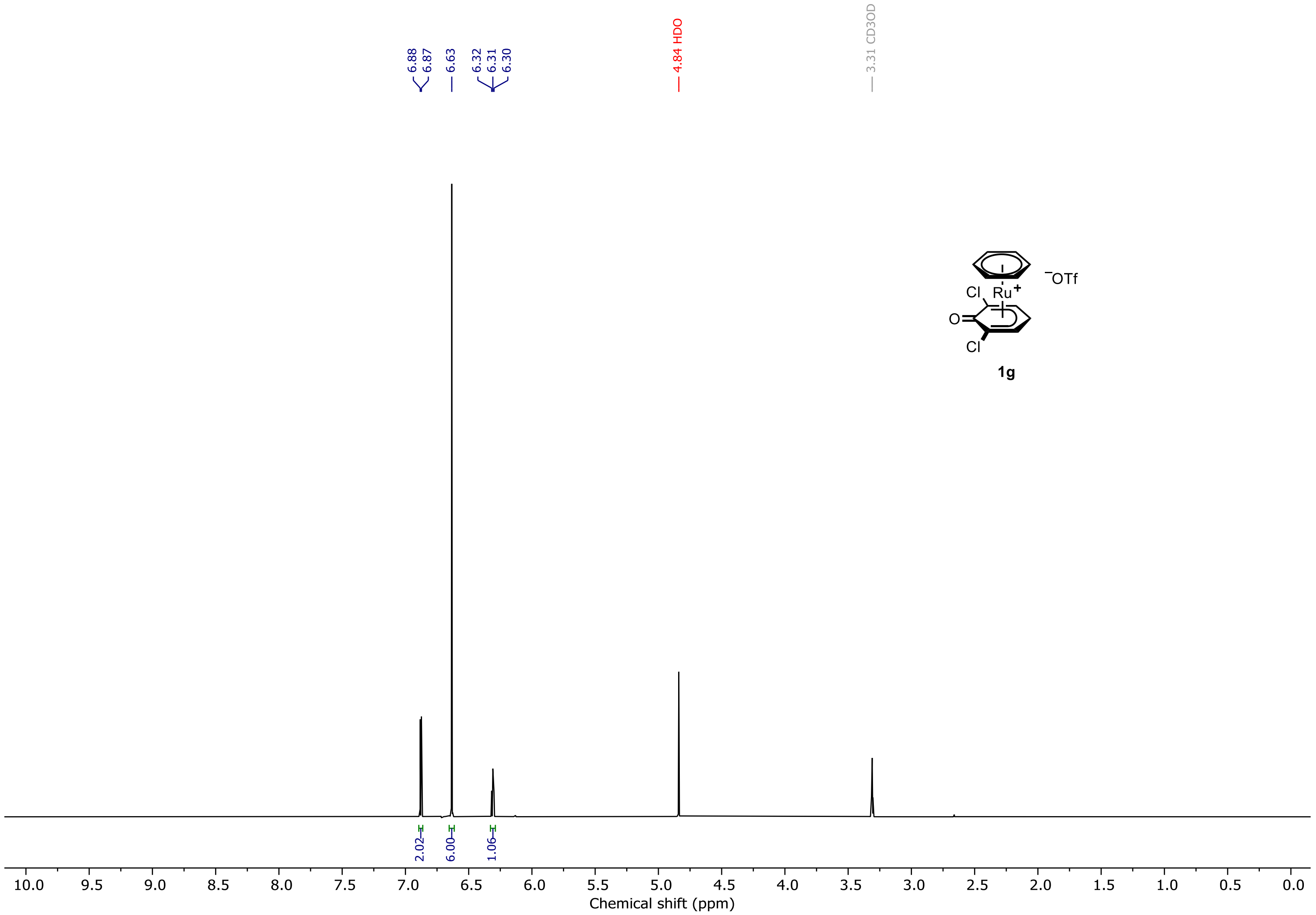

^{13}C NMR of [η^6-benzene-η^5-(2,6-dichloro-1-phenoxo)Ru](OTf) (1g)

CD_3OD, 151 MHz, 23 °C.

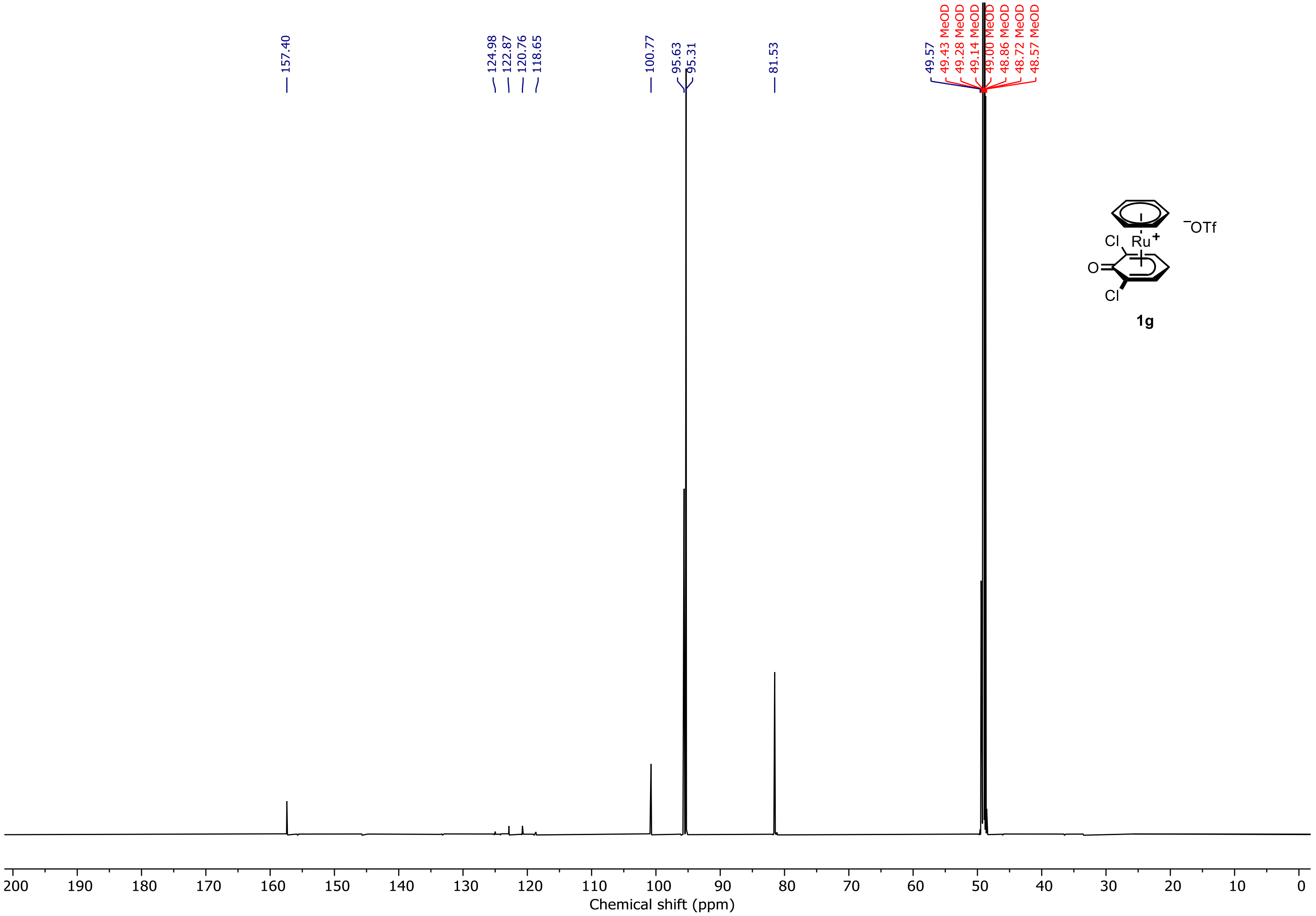

^{19}F NMR of [*η*^{6}-benzene-*η*^{5}-(2,6-dichloro-1-phenoxo)Ru](OTf) (1g)

CD_3OD, 600 MHz, 23 °C.

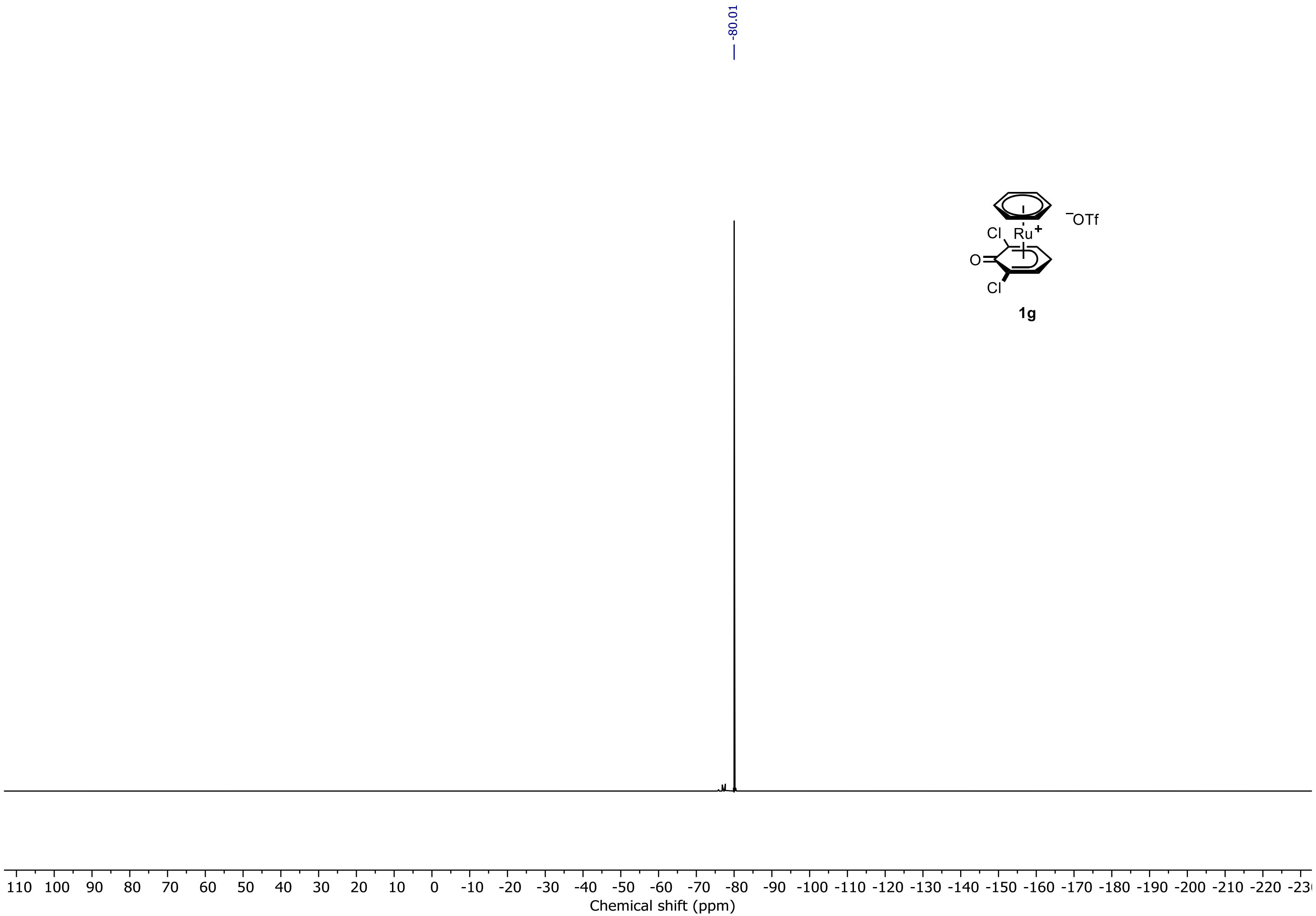

1g

^{1}H NMR of [η^6-benzene-η^5-(4-methyl-1-phenoxo)Ru](OTf) (1h)

CD_3OD, 600 MHz, 23 °C.

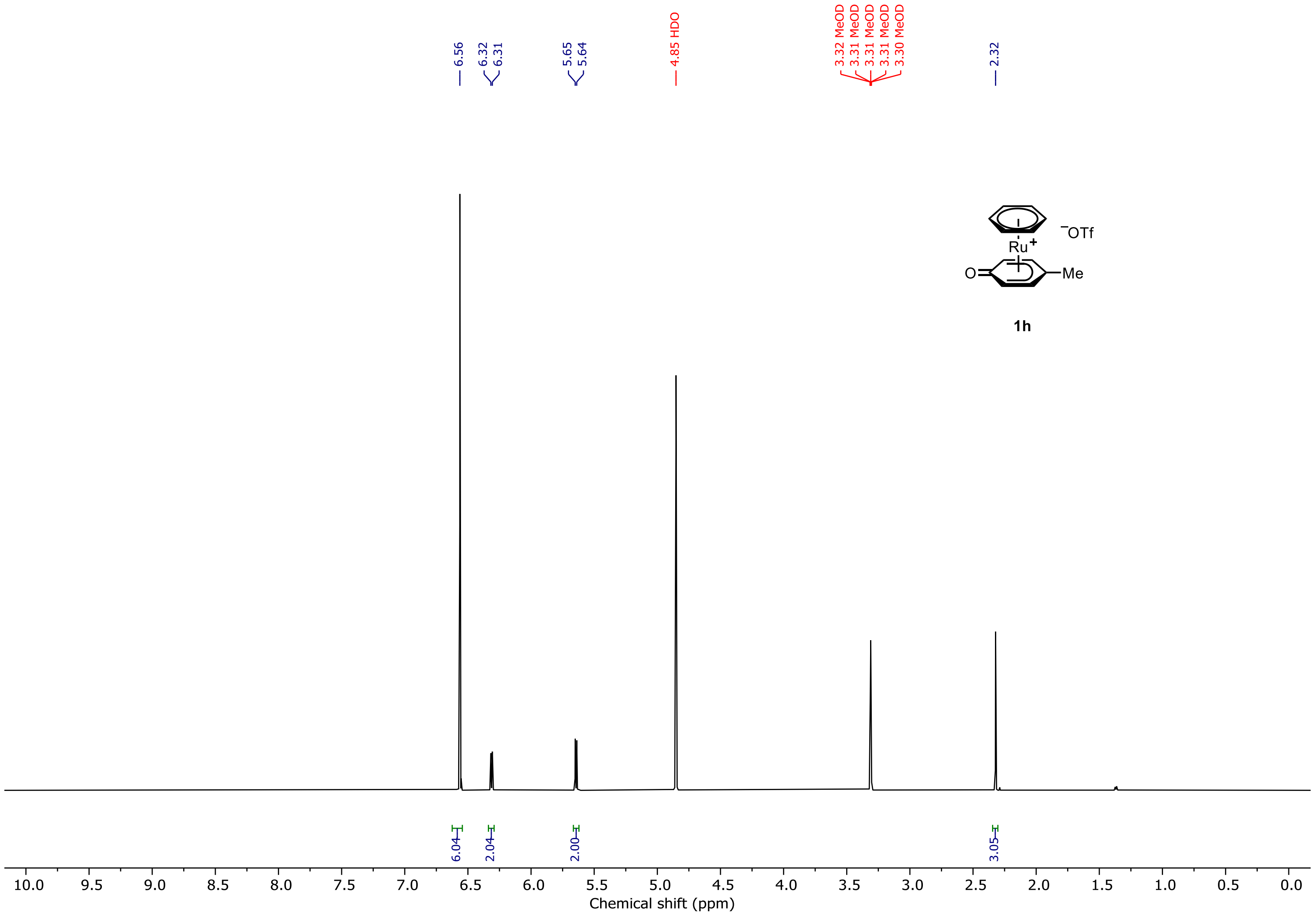

^{13}C NMR of [η^6-benzene-η^5-(4-methyl-1-phenoxo)Ru](OTf) (1h)

CD_3OD, 151 MHz, 23 °C.

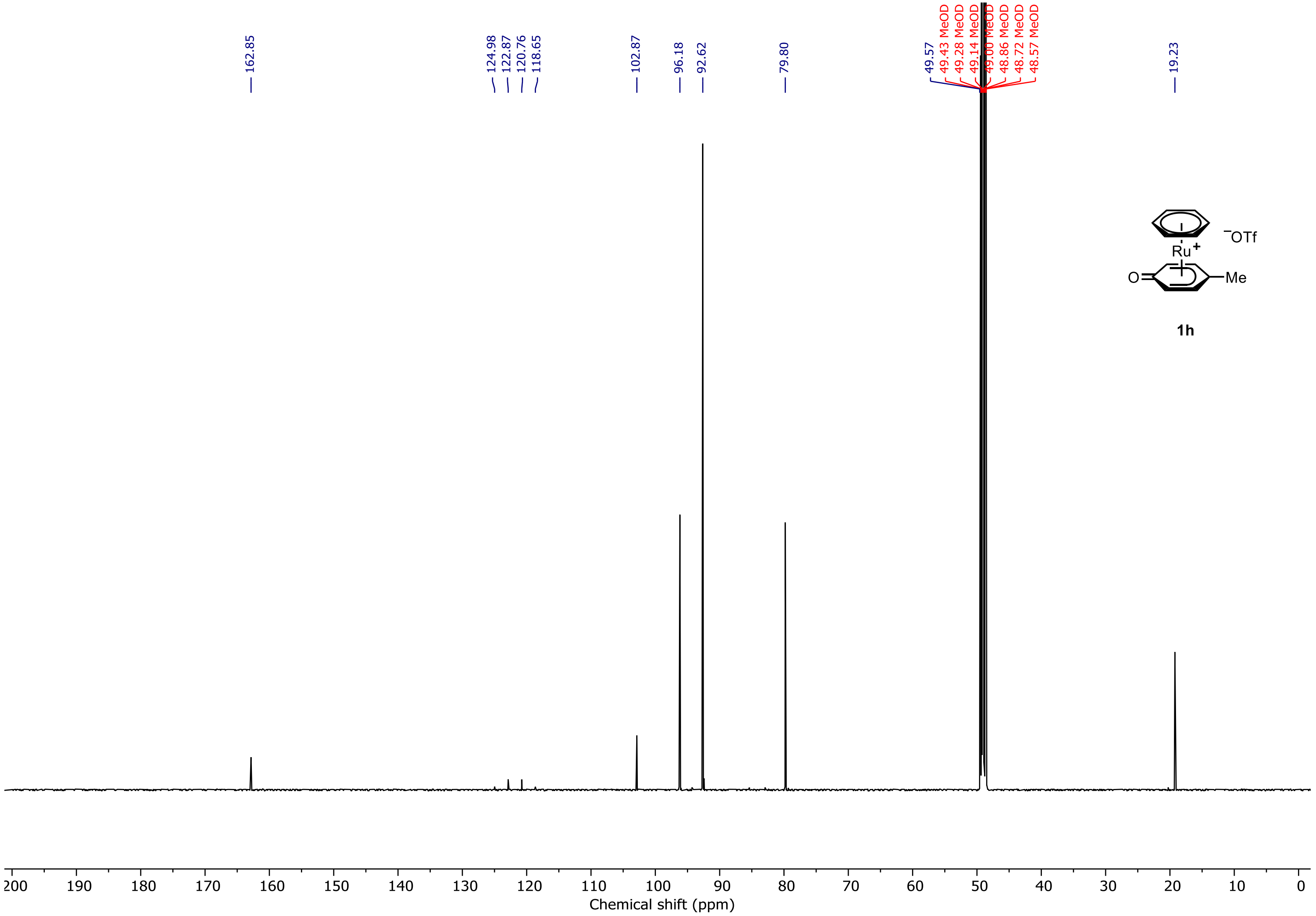

^{19}F NMR of [η^6-benzene-η^5-(4-methyl-1-phenoxo)Ru](OTf) (1h)

CD_3OD, 565 MHz, 23 °C.

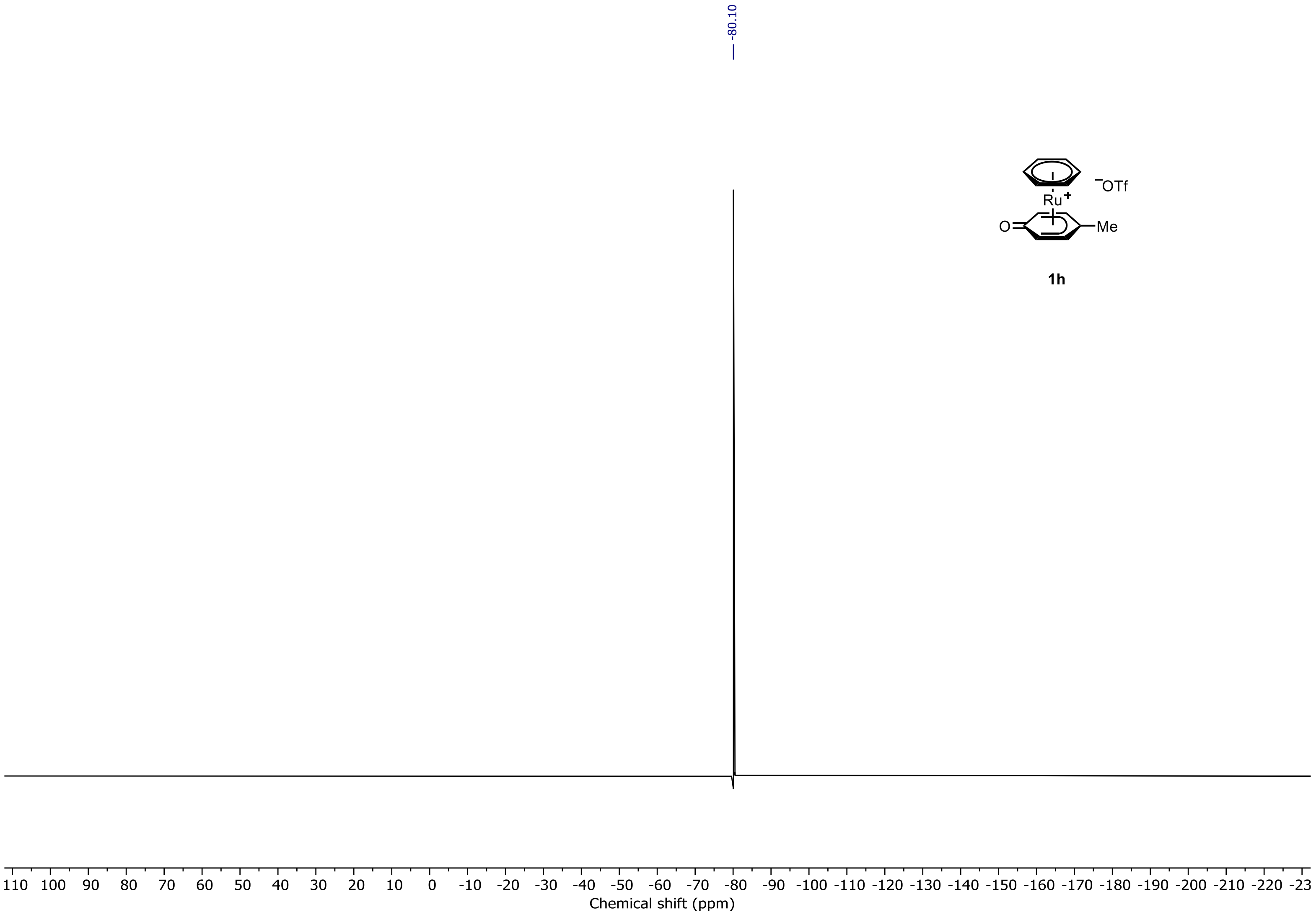

^{1}H NMR of [η^6-benzene-η^5-(4-methoxy-1-phenoxo)Ru](OTf) (1i)

CD_3OD, 600 MHz, 23 °C.

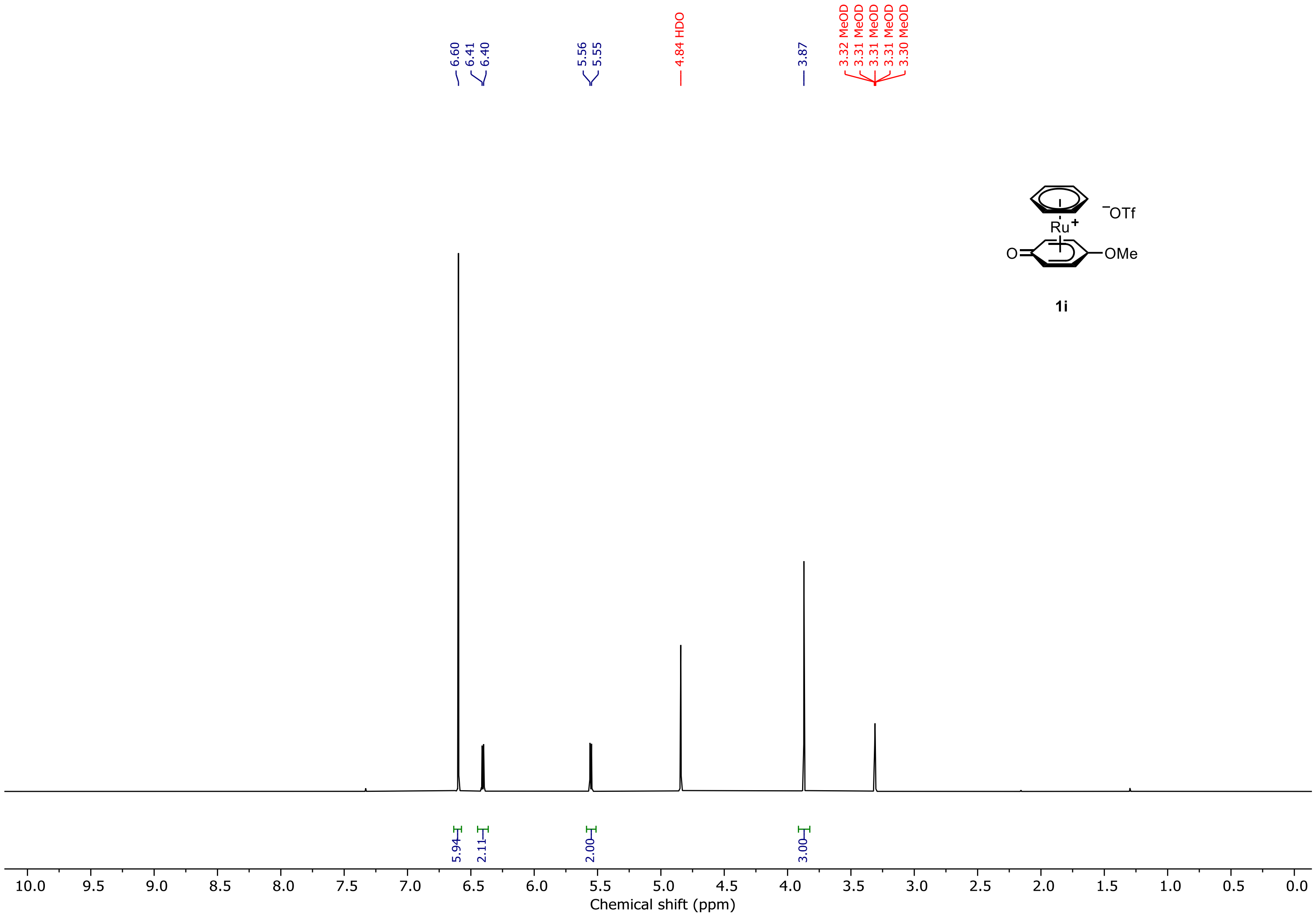

1i

^{13}C NMR of [η^6-benzene-η^5-(4-methoxy-1-phenoxo)Ru](OTf) (1i)

CD_3OD, 151 MHz, 23 °C.

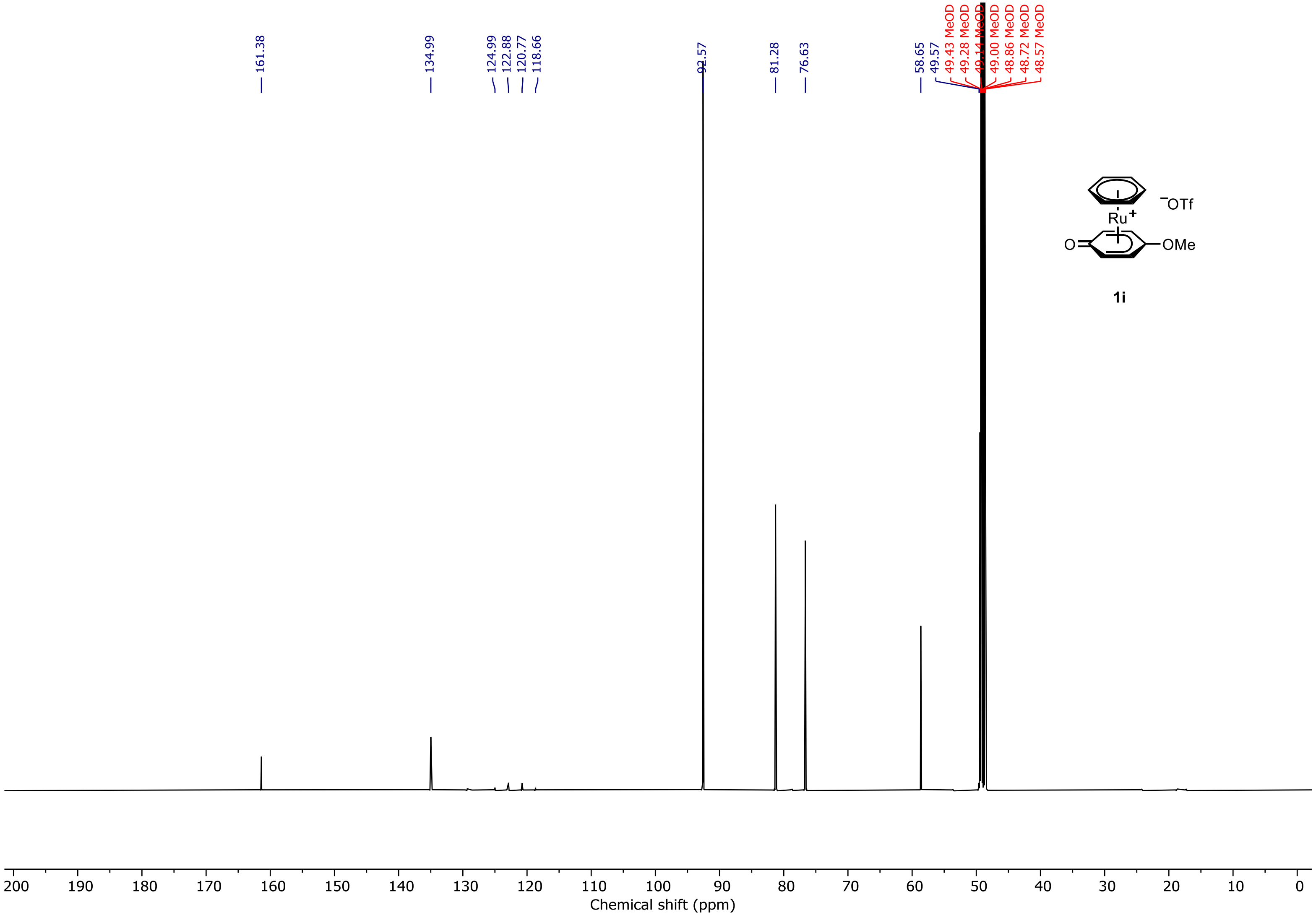

1i

^{19}F NMR of [η^6-benzene-η^5-(4-methoxy-1-phenoxo)Ru](OTf) (1i)

CD$_3$OD, 565 MHz, 23 °C.

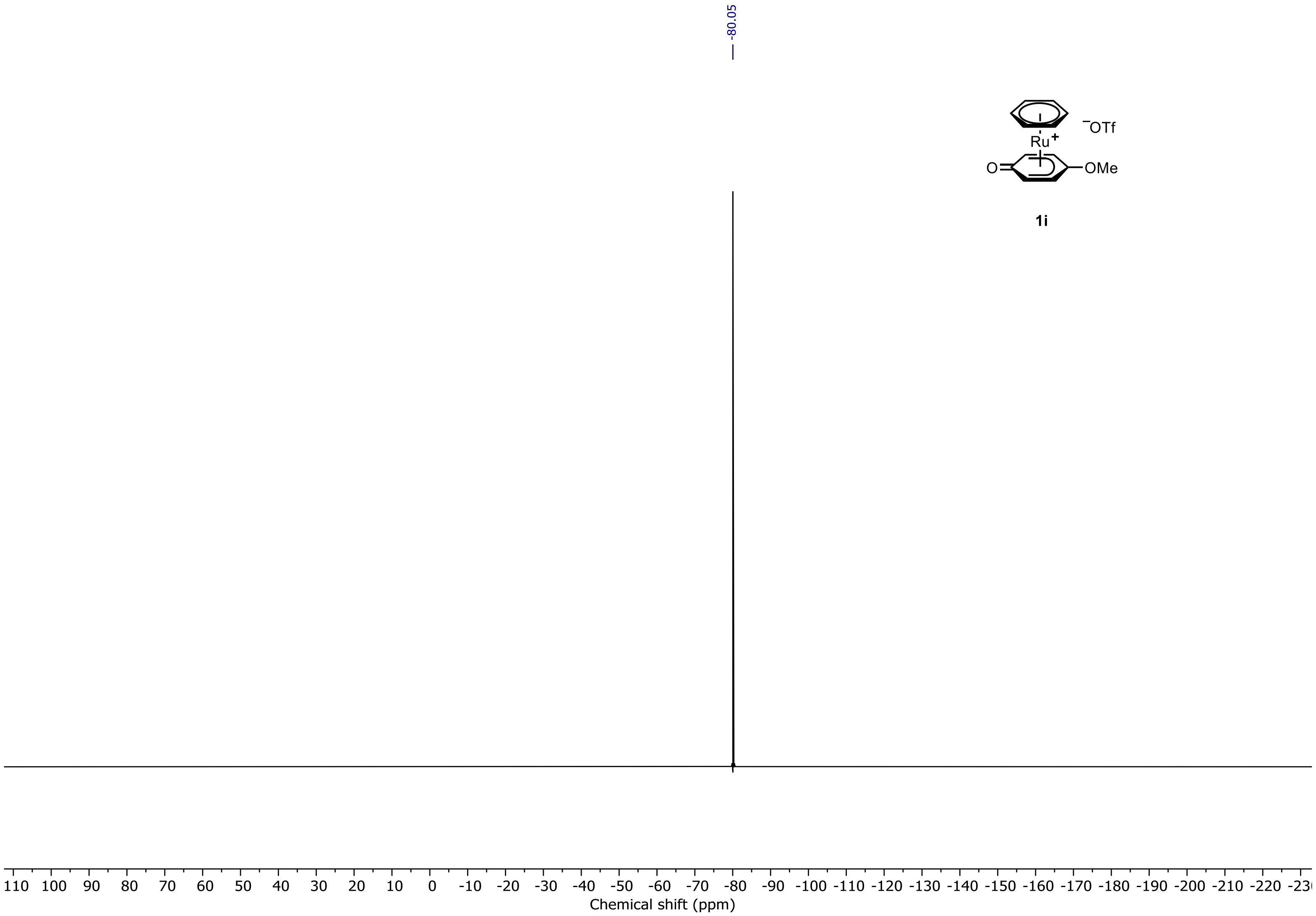

^{1}H NMR of [η^6-benzene-η^5-(4-trifluoromethyl-1-phenoxo)-1-phenoxo)Ru](OTf) (1j)

CD_3CN, 600 MHz, 23 °C.

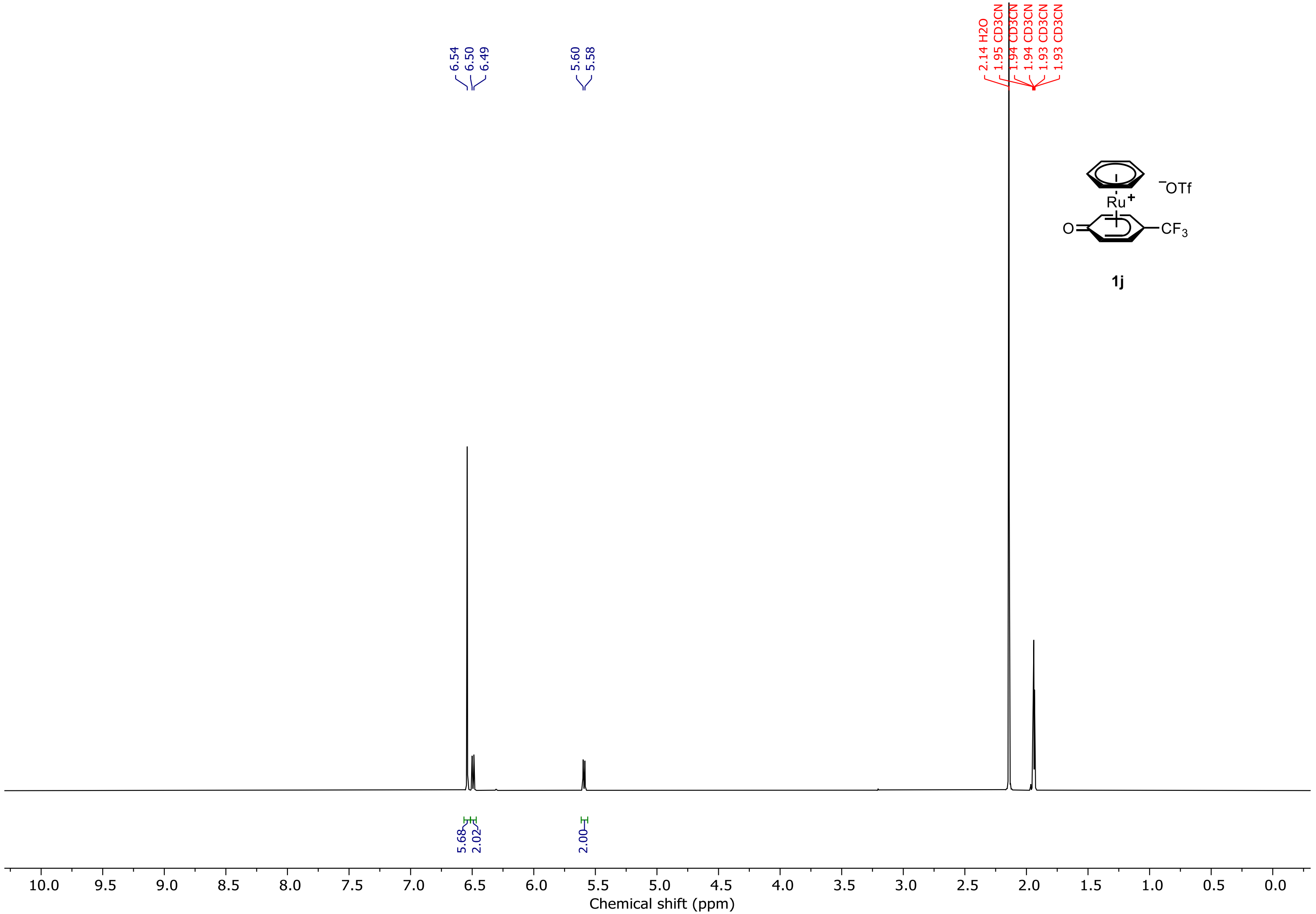

^{13}C NMR of [η^6-benzene-η^5-(4-trifluoromethyl-1-phenoxo)Ru](OTf) (1j)

CD_3CN, 151 MHz, 23 °C.

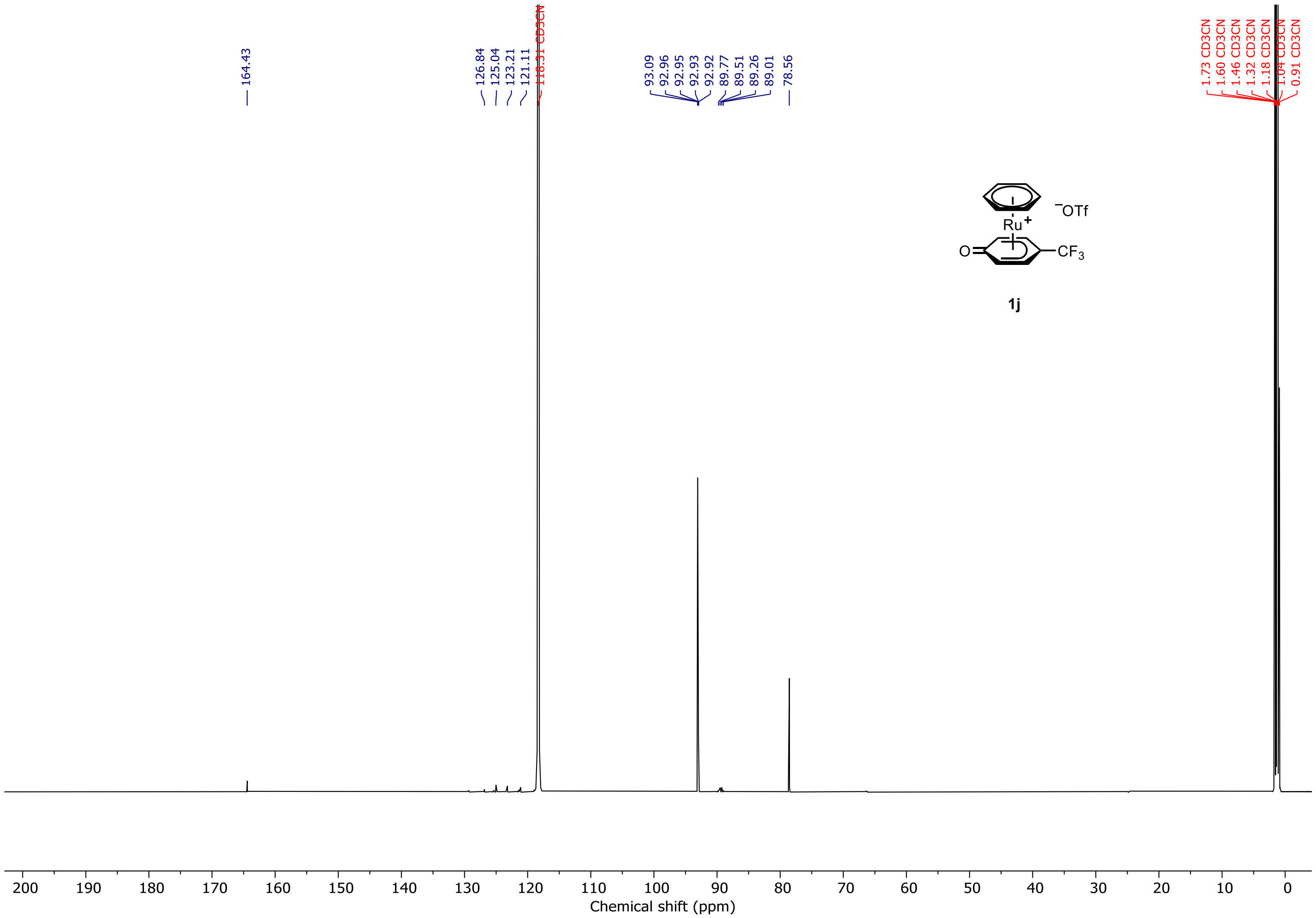

^{19}F NMR of [η^6-benzene-η^5-(4-trifluoromethyl-1-phenoxo)Ru](OTf) (1j)

CD_3CN, 565 MHz, 23 °C.

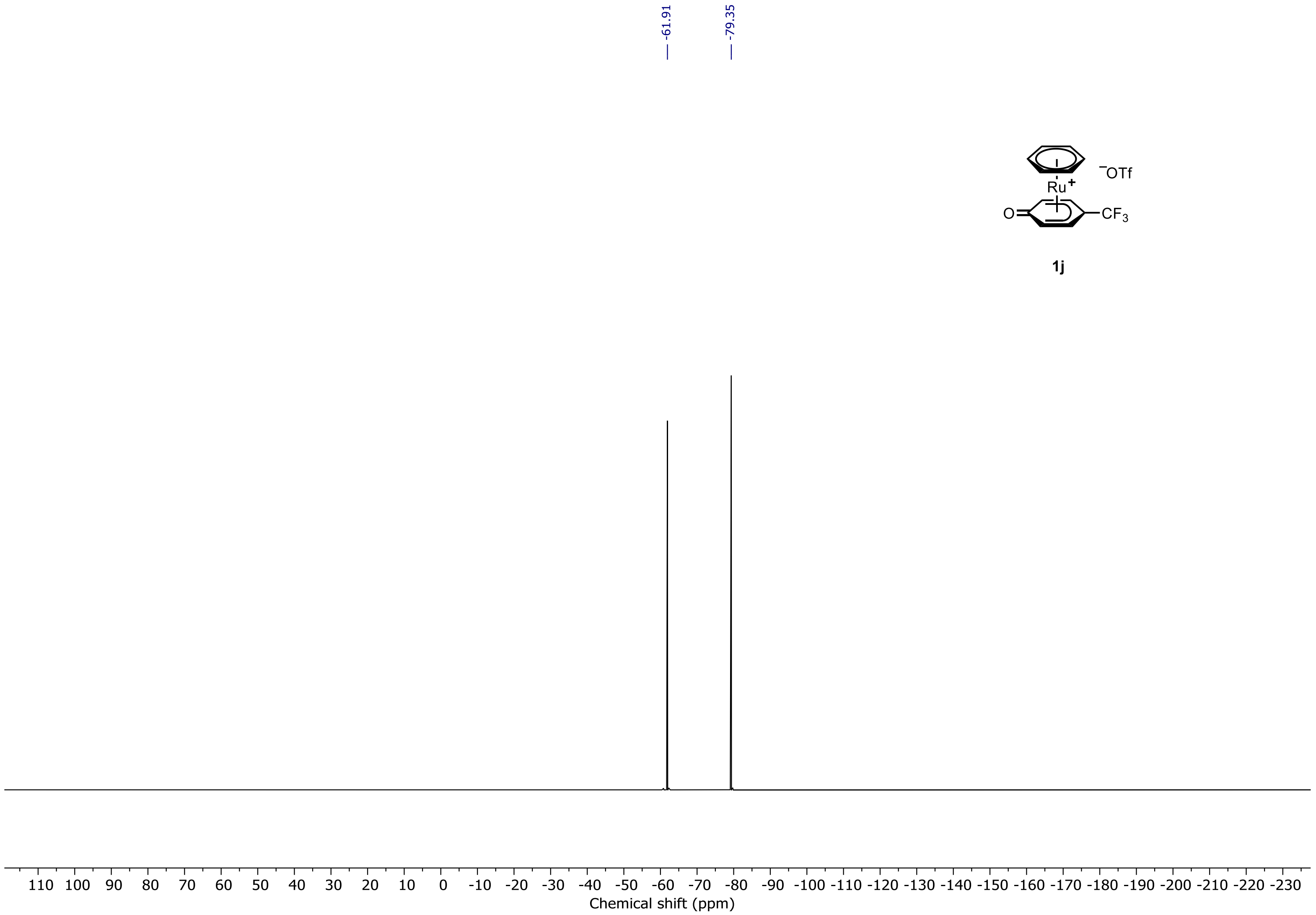

^{1}H NMR of [η^6-benzene-η^5-(2,6-dibromo-1-phenoxo)Ru](OTf) (1k)

CD_3CN, 600 MHz, 23 °C.

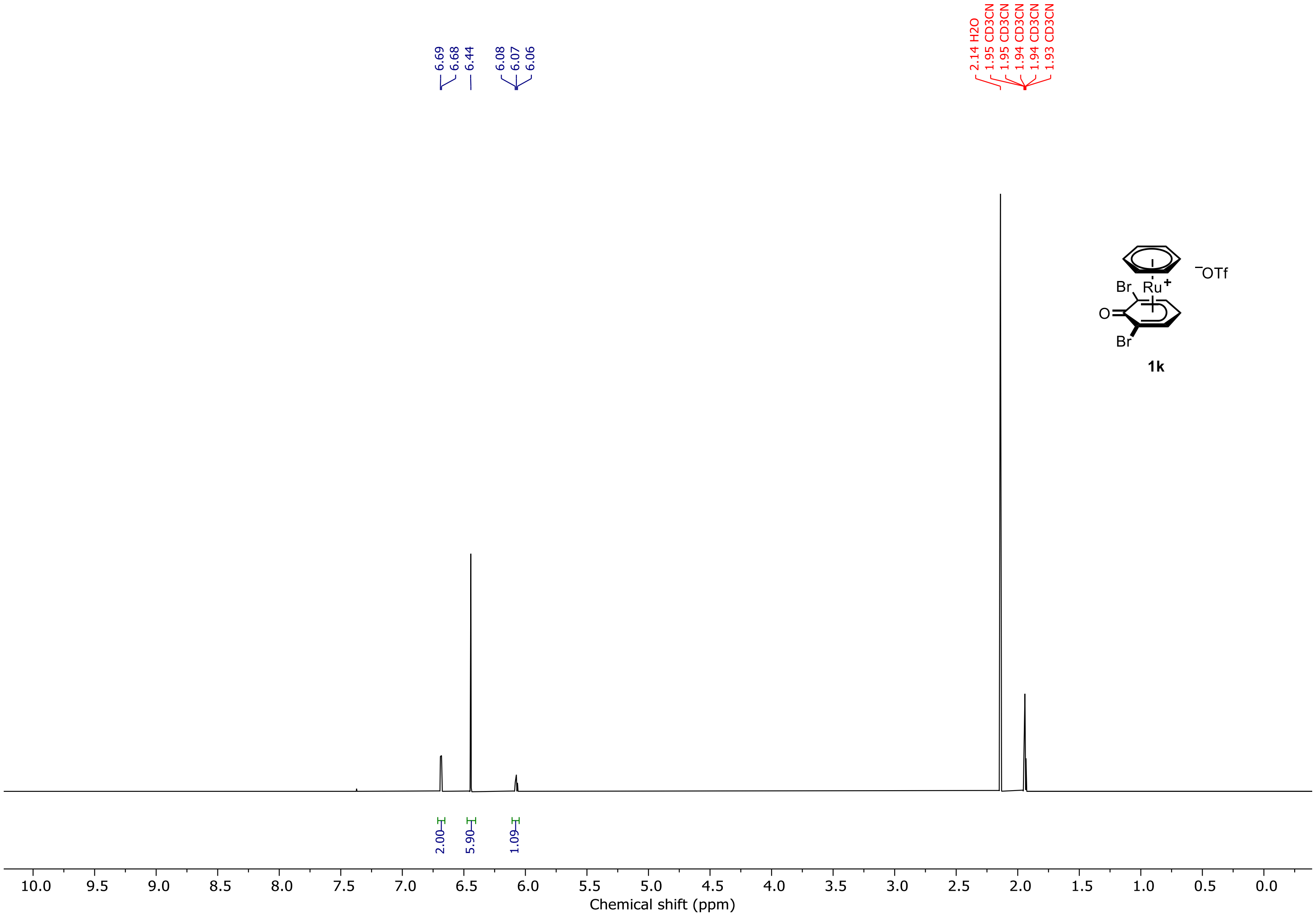

^{13}C NMR of [η^6-benzene-η^5-(2,6-dibromo-1-phenoxo)Ru](OTf) (1k)

CD$_3$CN, 151 MHz, 23 °C.

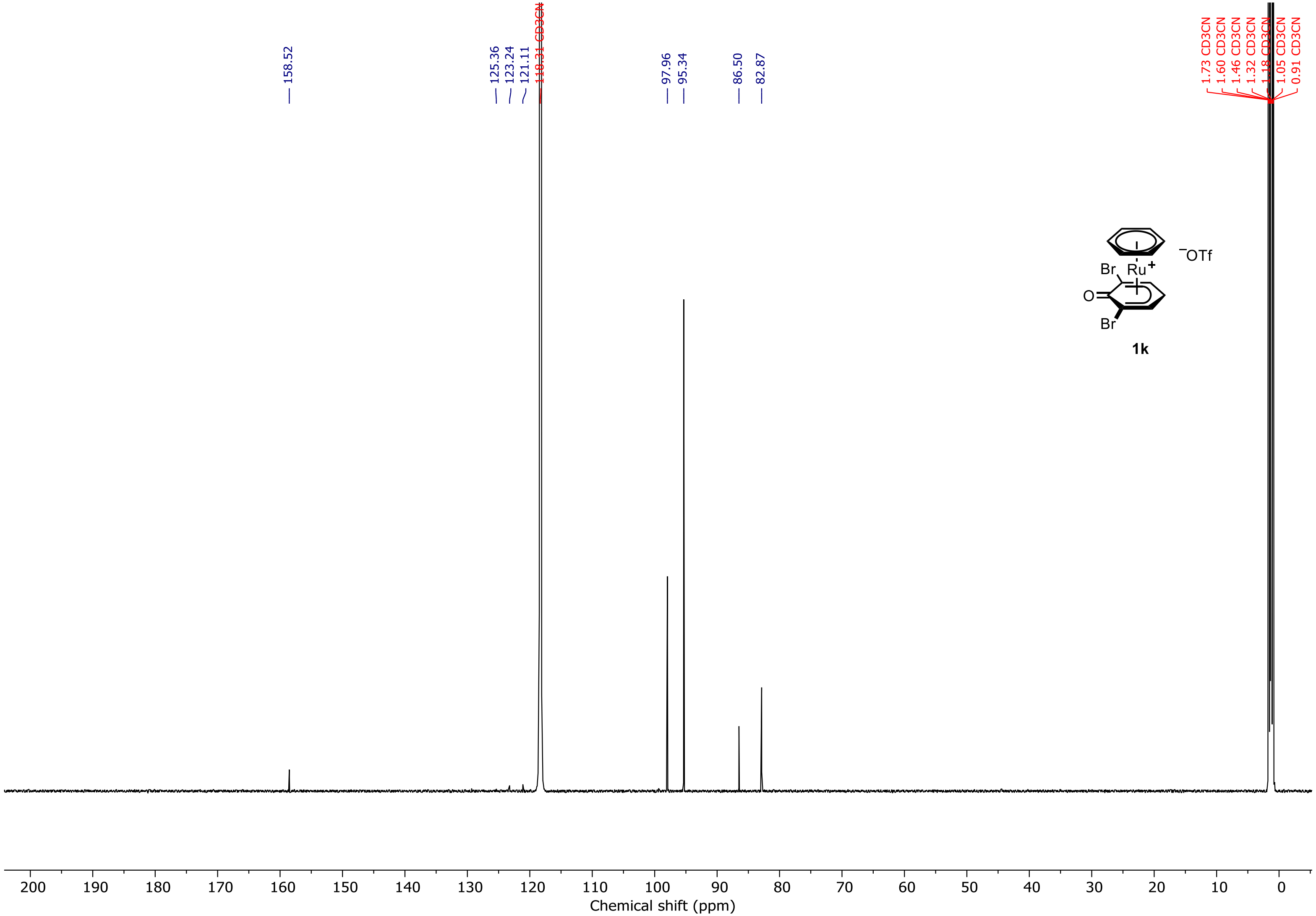

^{19}F NMR of [η^6-benzene-η^5-(2,6-dibromo-1-phenoxo)Ru](OTf) (1k)

CD_3CN, 470 MHz, 23 °C.

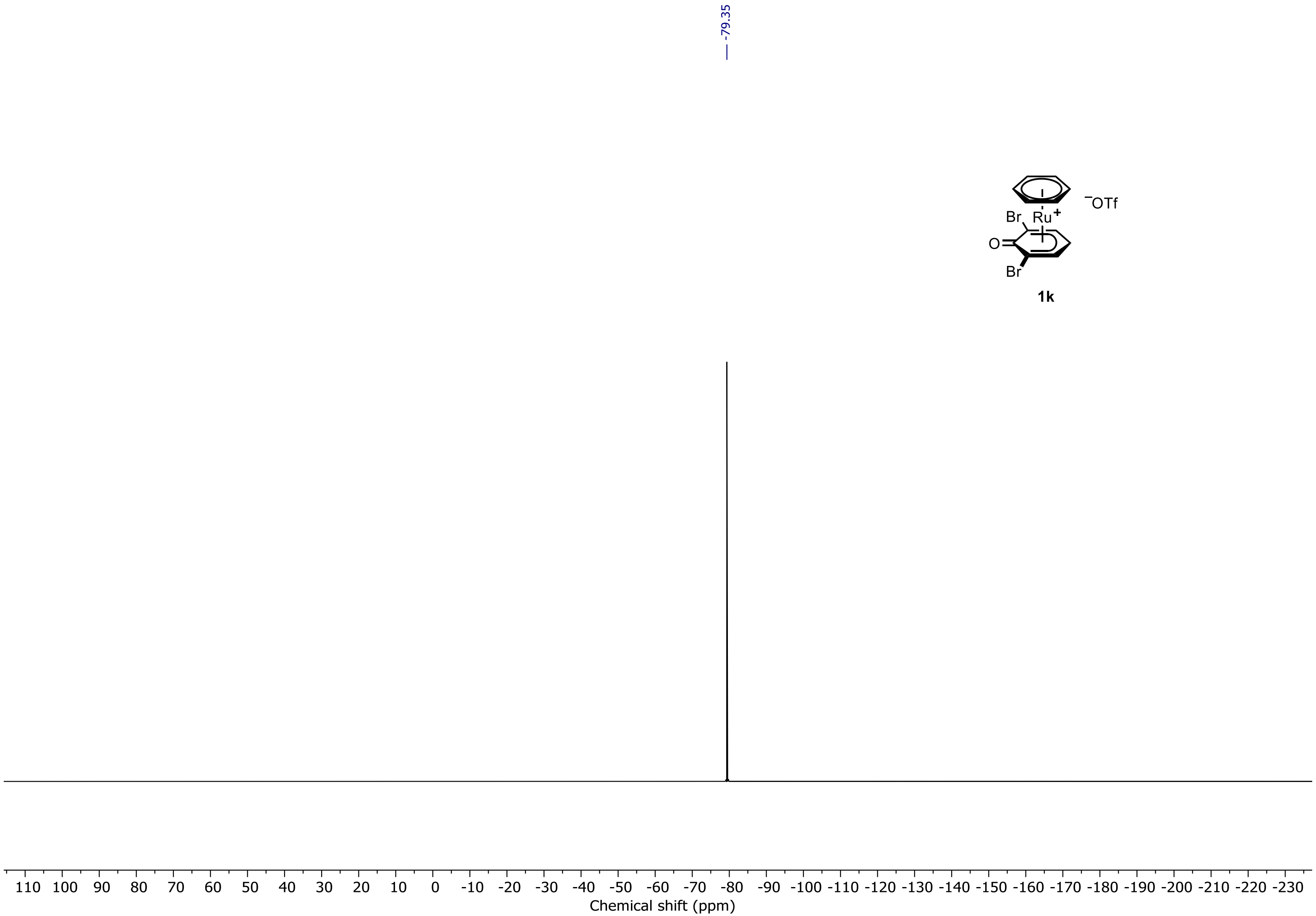

^{1}H NMR of [η^6-benzene-η^5-(4-(N,N-dimethylamino)-1-phenoxo)Ru](OTf) (1l)

CD_3OD, 600 MHz, 23 °C.

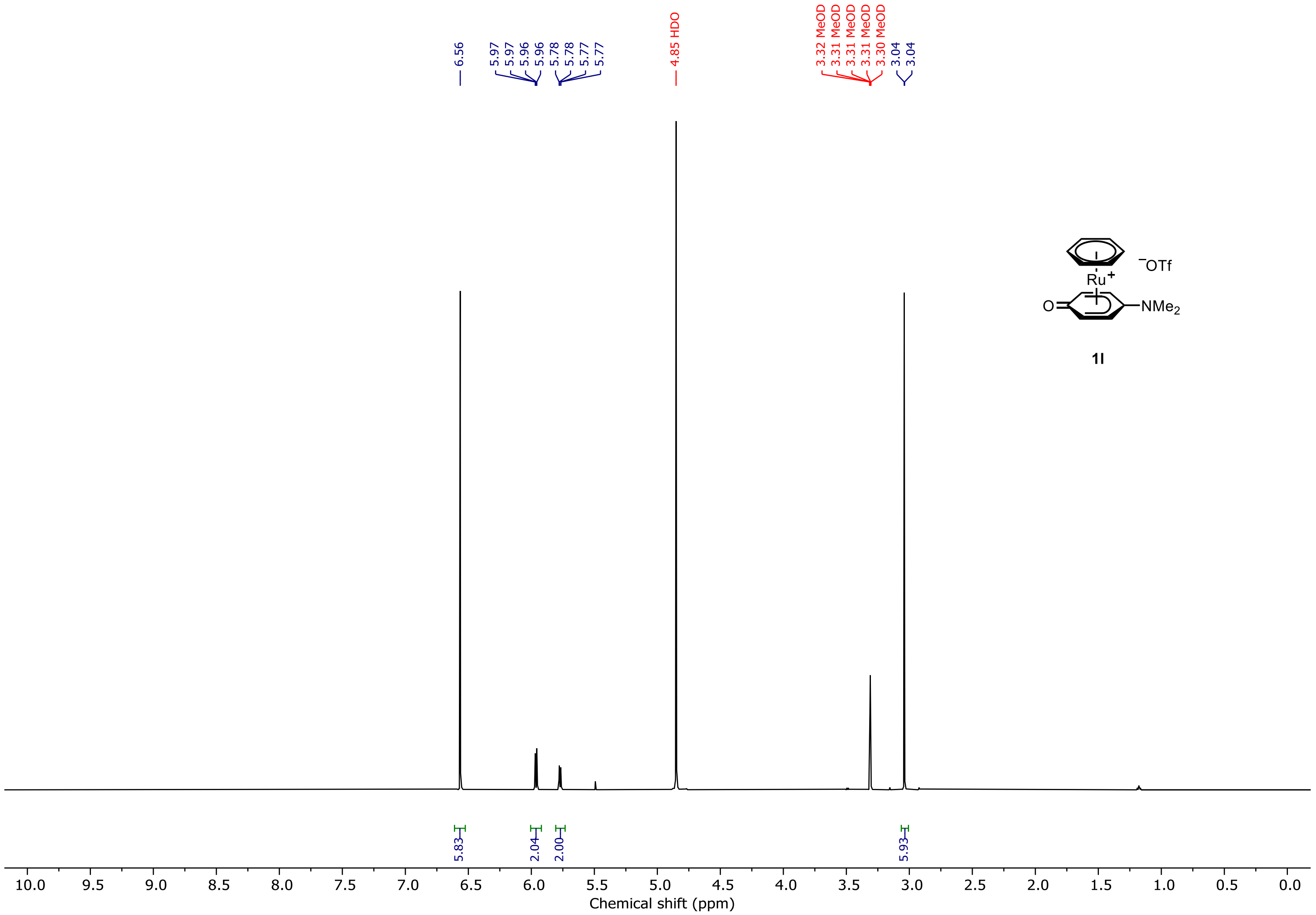

^{13}C NMR of [*η*^{6}-benzene-*η*^{5}-(4-(N,N-dimethylamino)-1-phenoxo)Ru](OTf) (1l)

CD_3OD, 151 MHz, 23 °C.

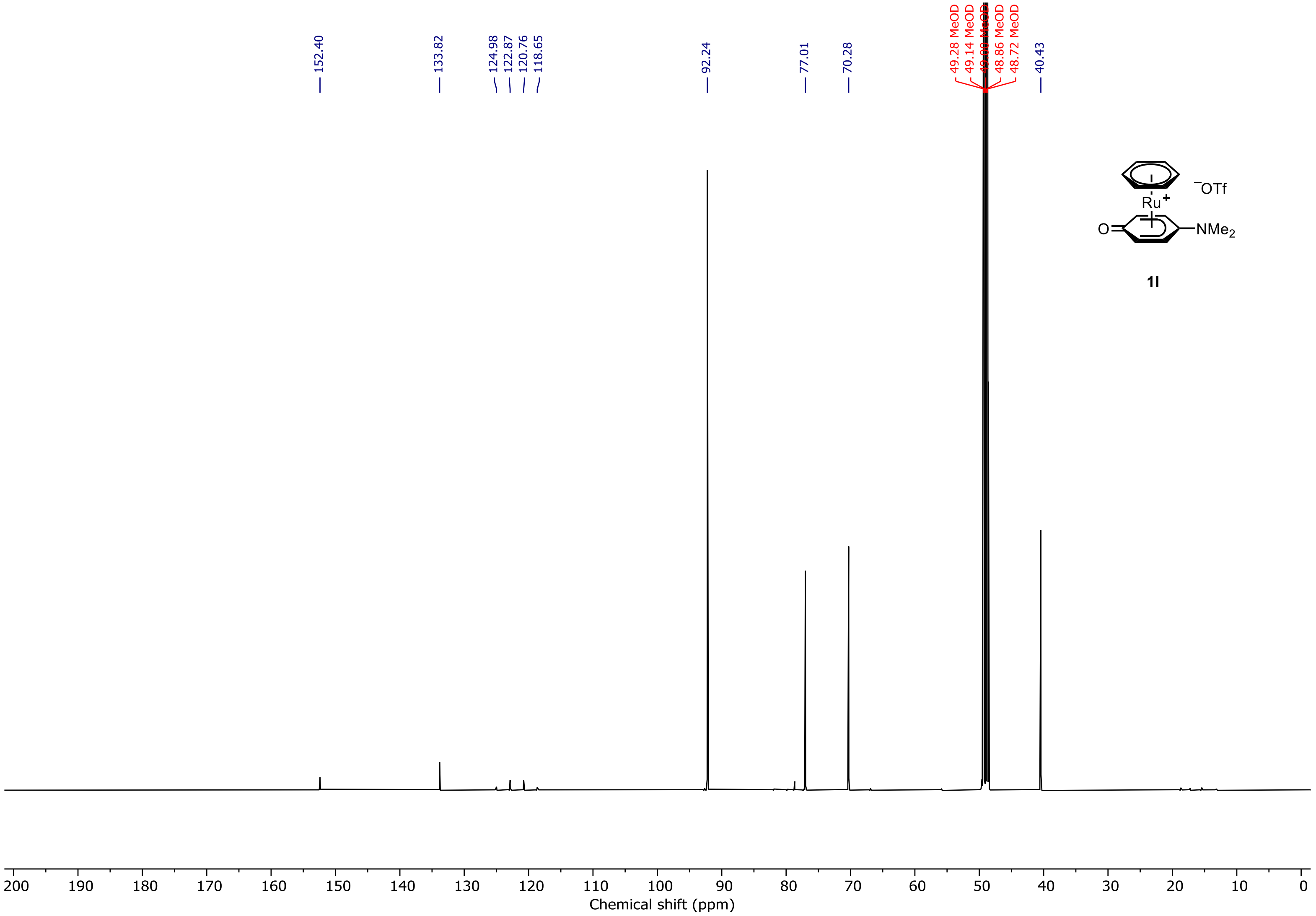

^{19}F NMR of [*η*[6]-benzene-*η*[5]-(4-(N,N-dimethylamino)-1-phenoxo)Ru](OTf) (1l)

CD_3OD, 565 MHz, 23 °C.

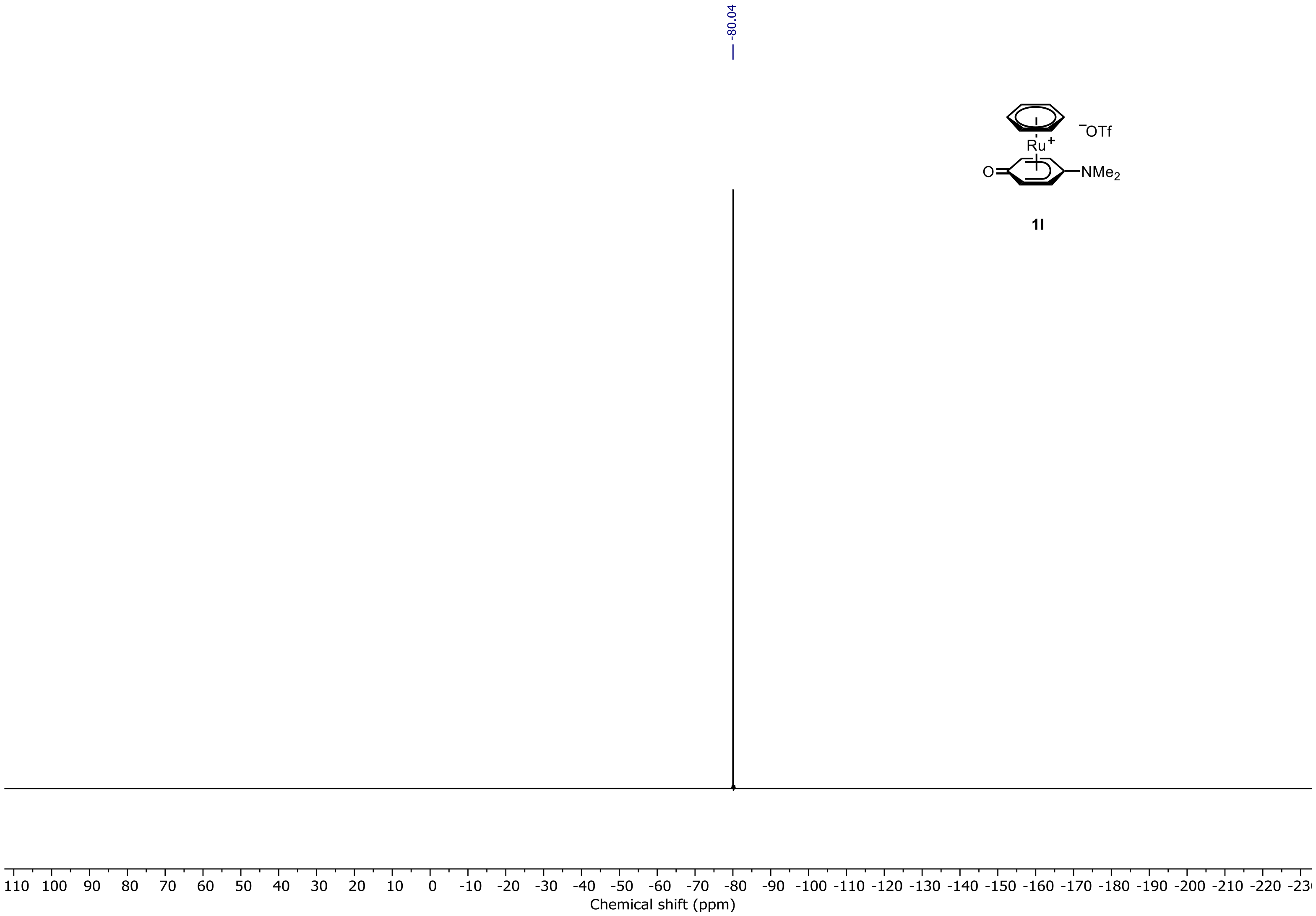

1l

^{1}H NMR of [η^6-benzene-η^5-(1-phenoxo-4-phenyl)Ru](OTf) (SI-1)

CD_3CN, 600 MHz, 23 °C.

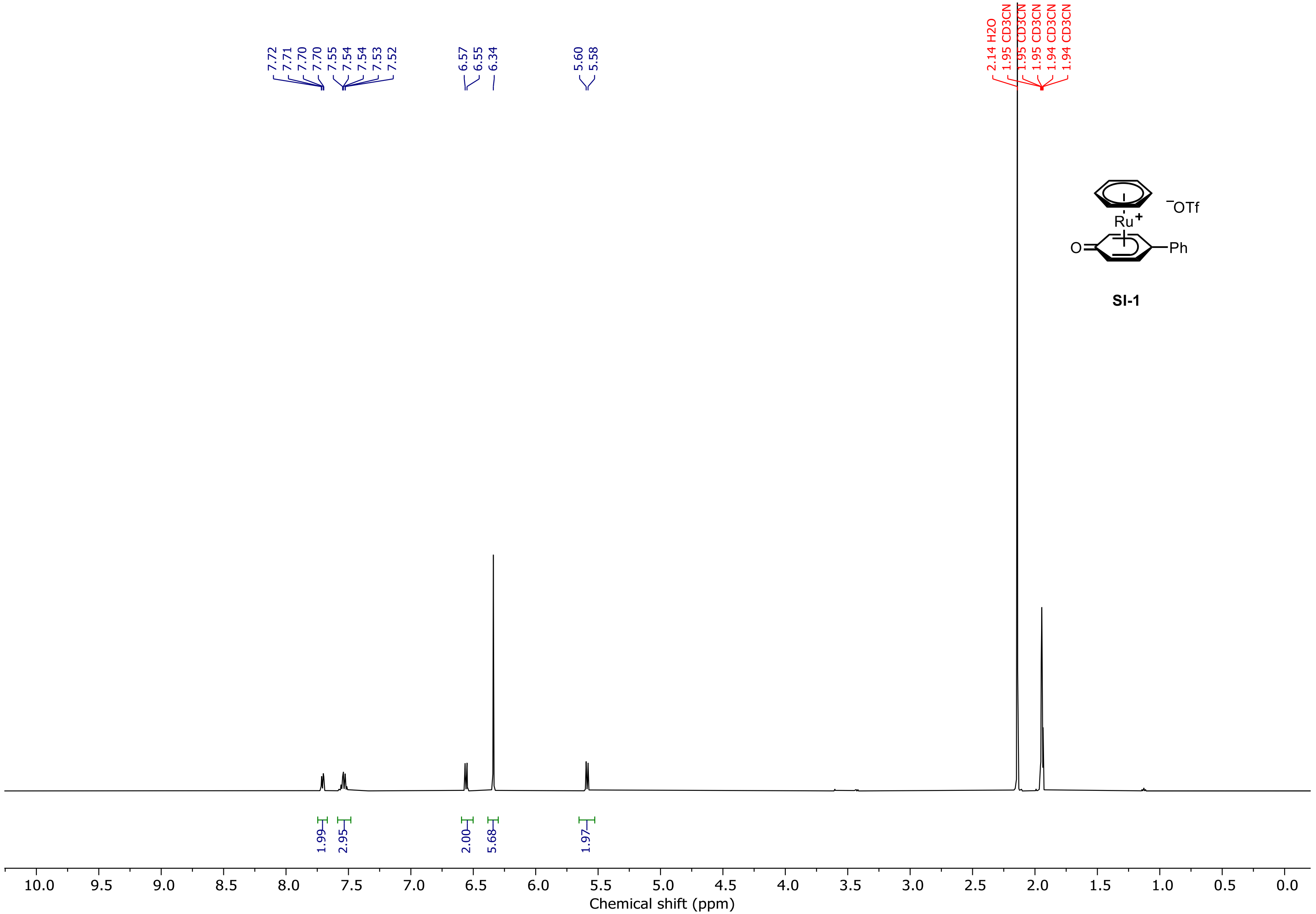

^{13}C NMR of [η^6-benzene-η^5-(1-phenoxo-4-phenyl)Ru](OTf) (SI-1)

CD_3CN, 151 MHz, 23 °C.

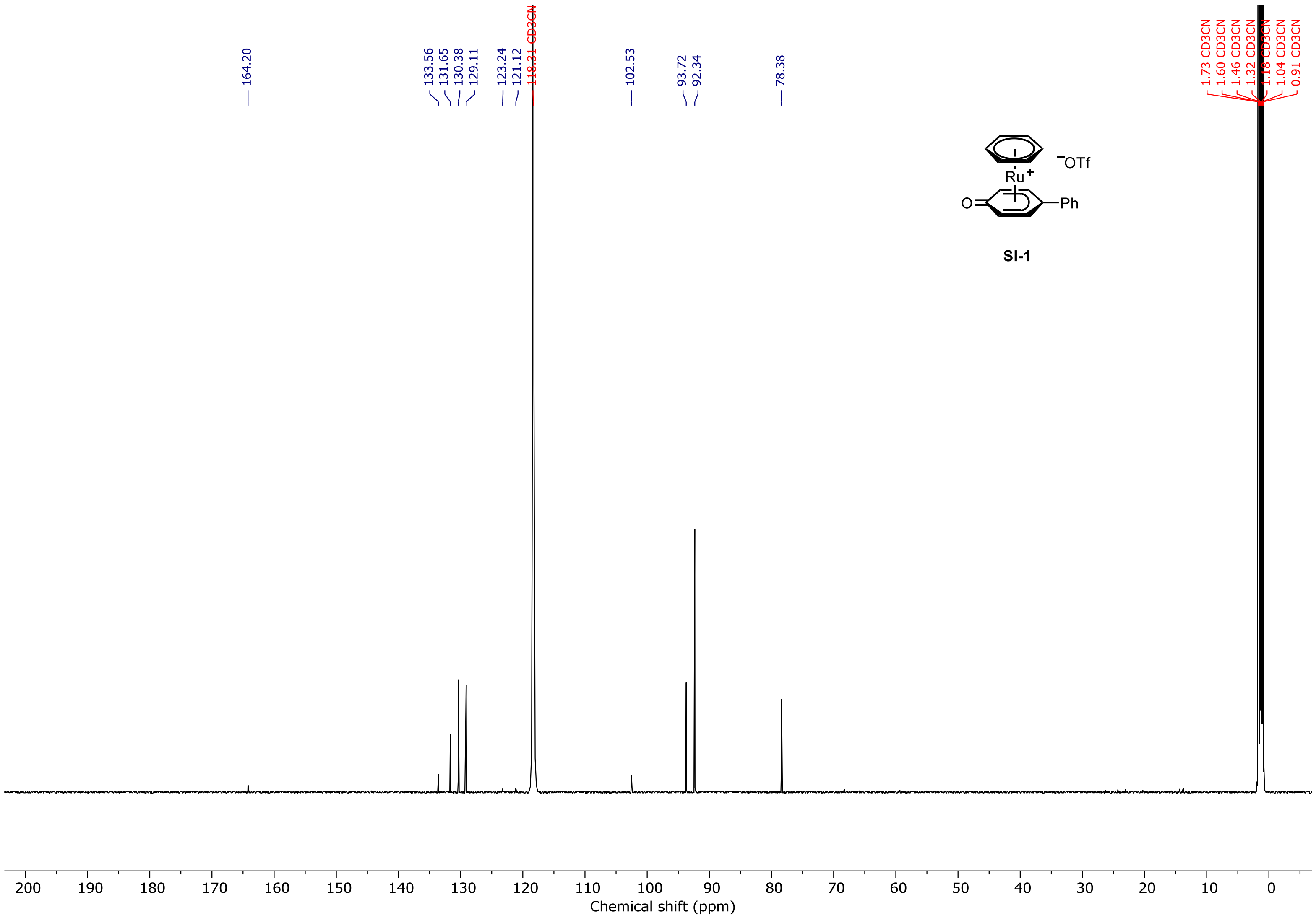

SI-1

^{19}F NMR of [η^6-benzene-η^5-(1-phenoxo-4-phenyl)Ru](OTf) (SI-1)

CD_3CN, 470 MHz, 23 °C.

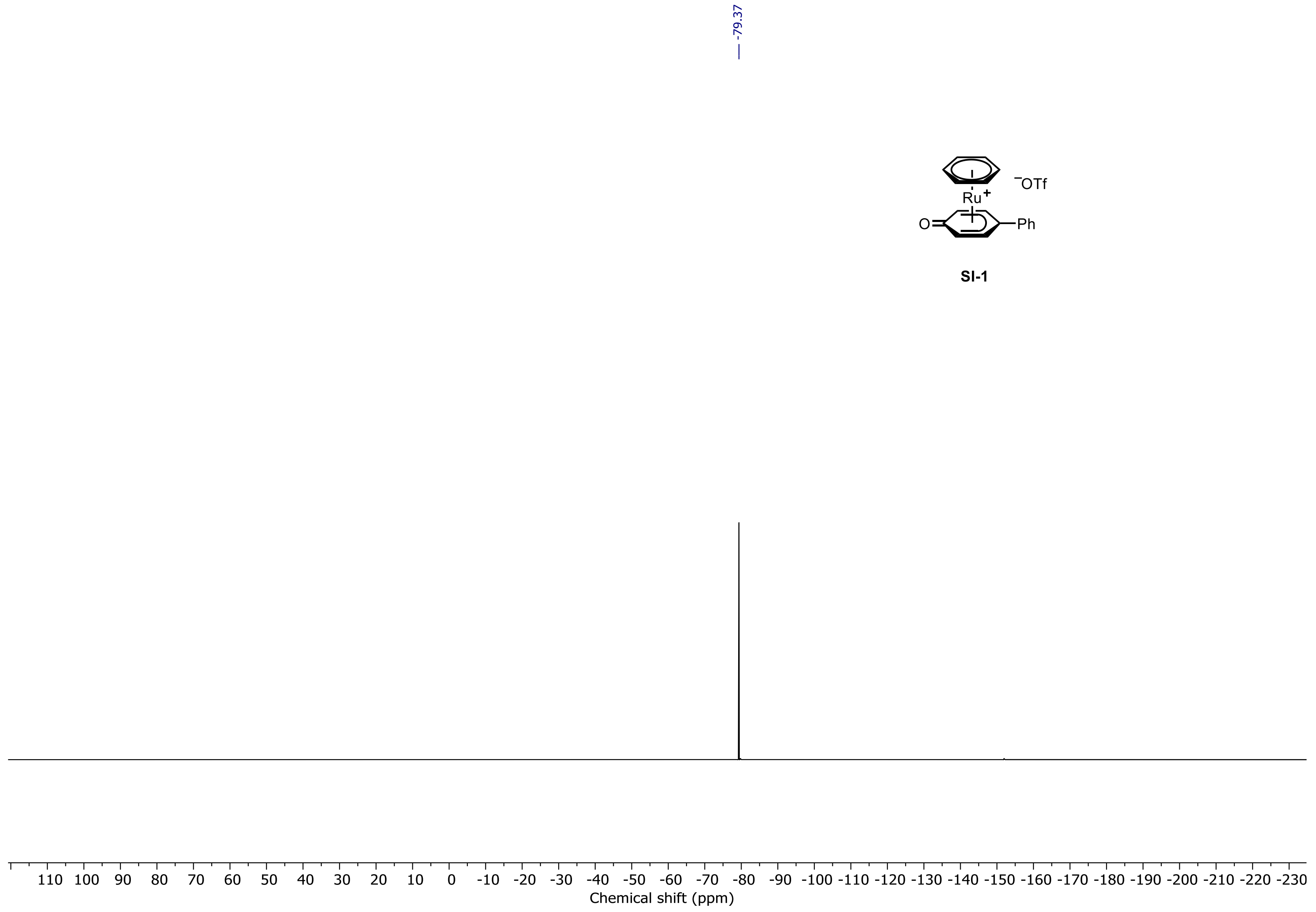

^{1}H NMR of [η^6-benzene-η^5-(3,5-dimethoxy-1-phenoxo)Ru](OTf) (SI-2)

CD_3CN, 600 MHz, 23 °C.

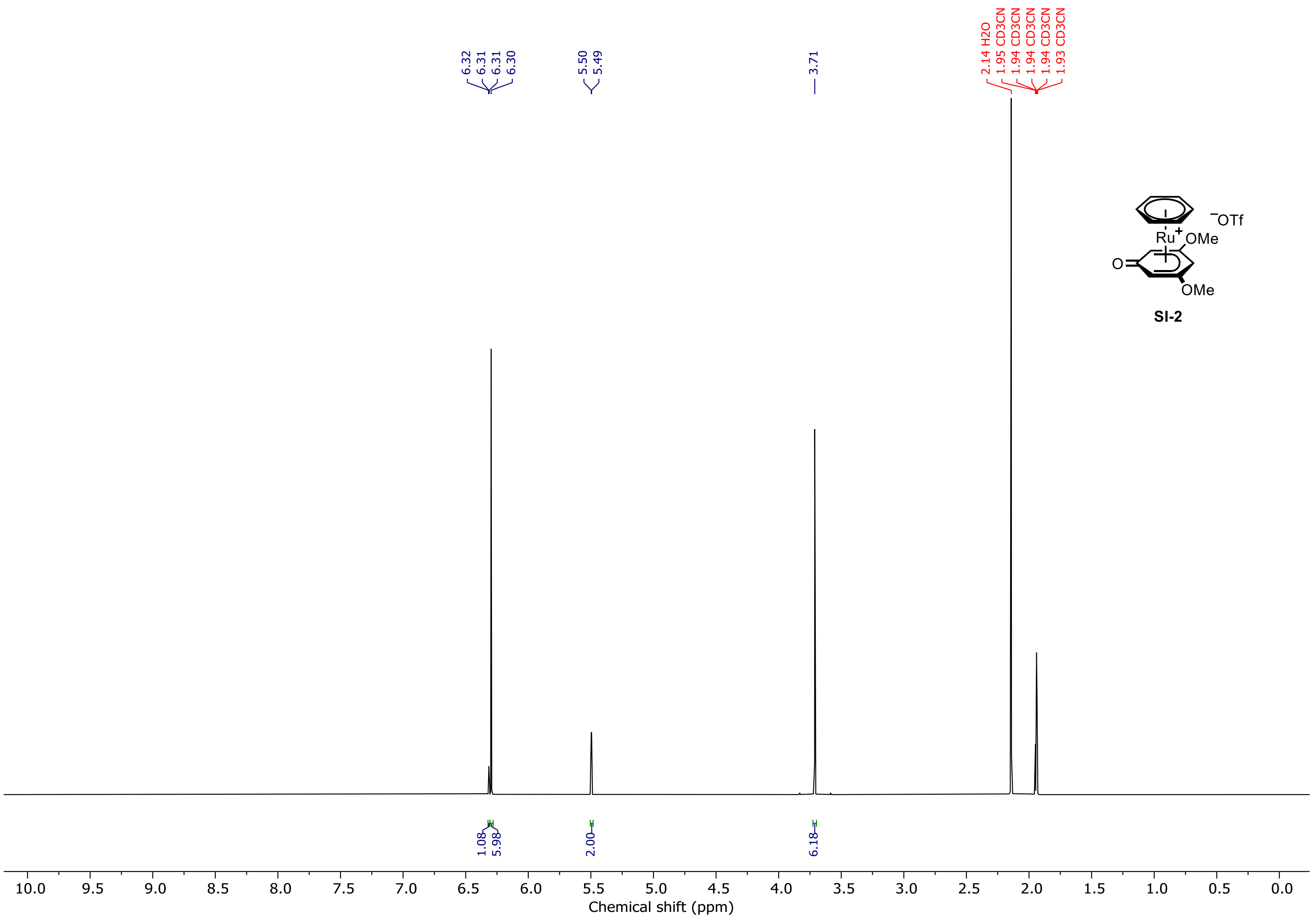

^{13}C NMR of [η^6-benzene-η^5-(3,5-dimethoxy-1-phenoxo)Ru](OTf) (SI-2)

CD_3CN, 151 MHz, 23 °C.

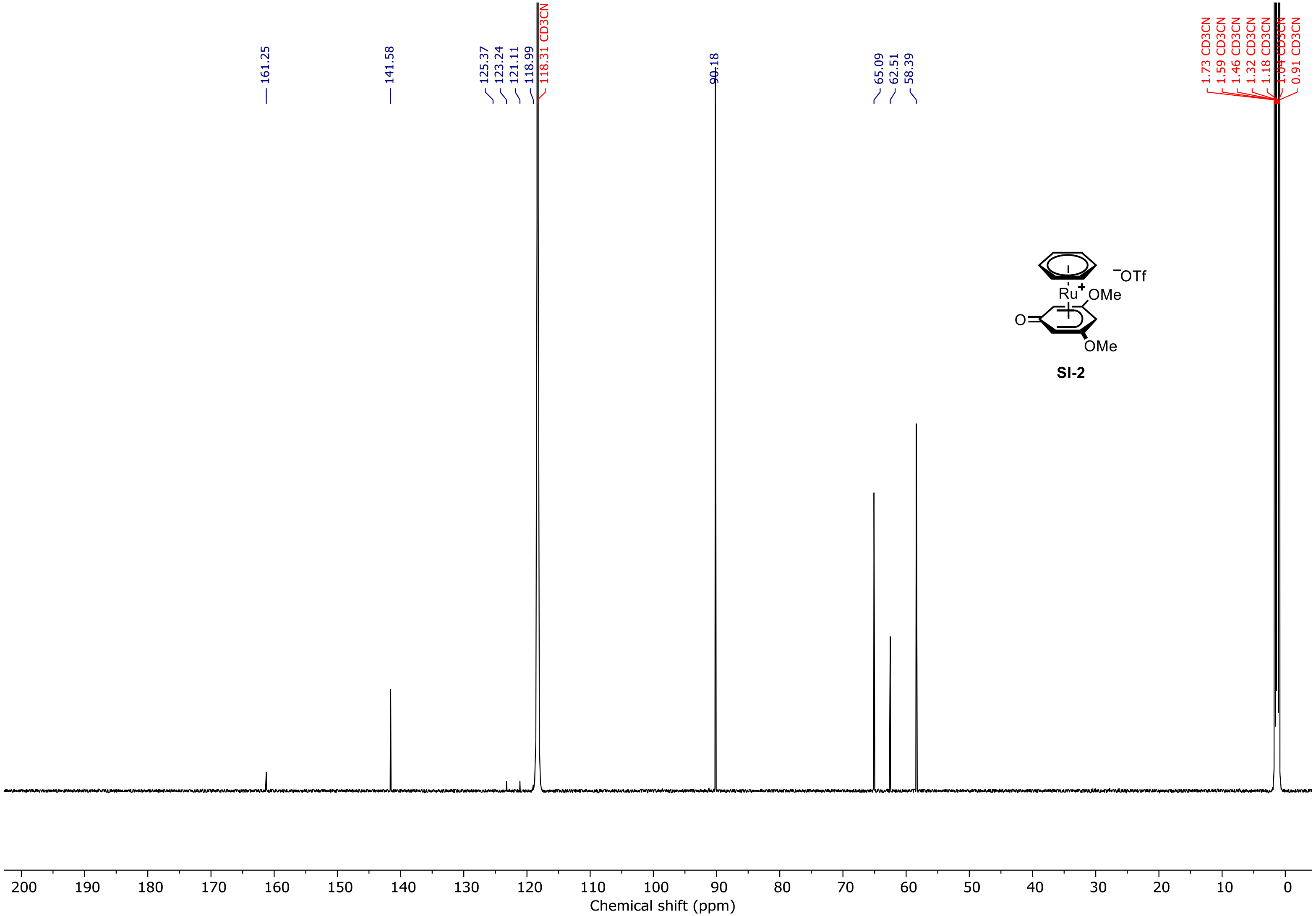

^{19}F NMR of [η^6-benzene-η^5-(3,5-dimethoxy-1-phenoxo)Ru](OTf) (SI-2)

CD_3CN, 470 MHz, 23 °C.

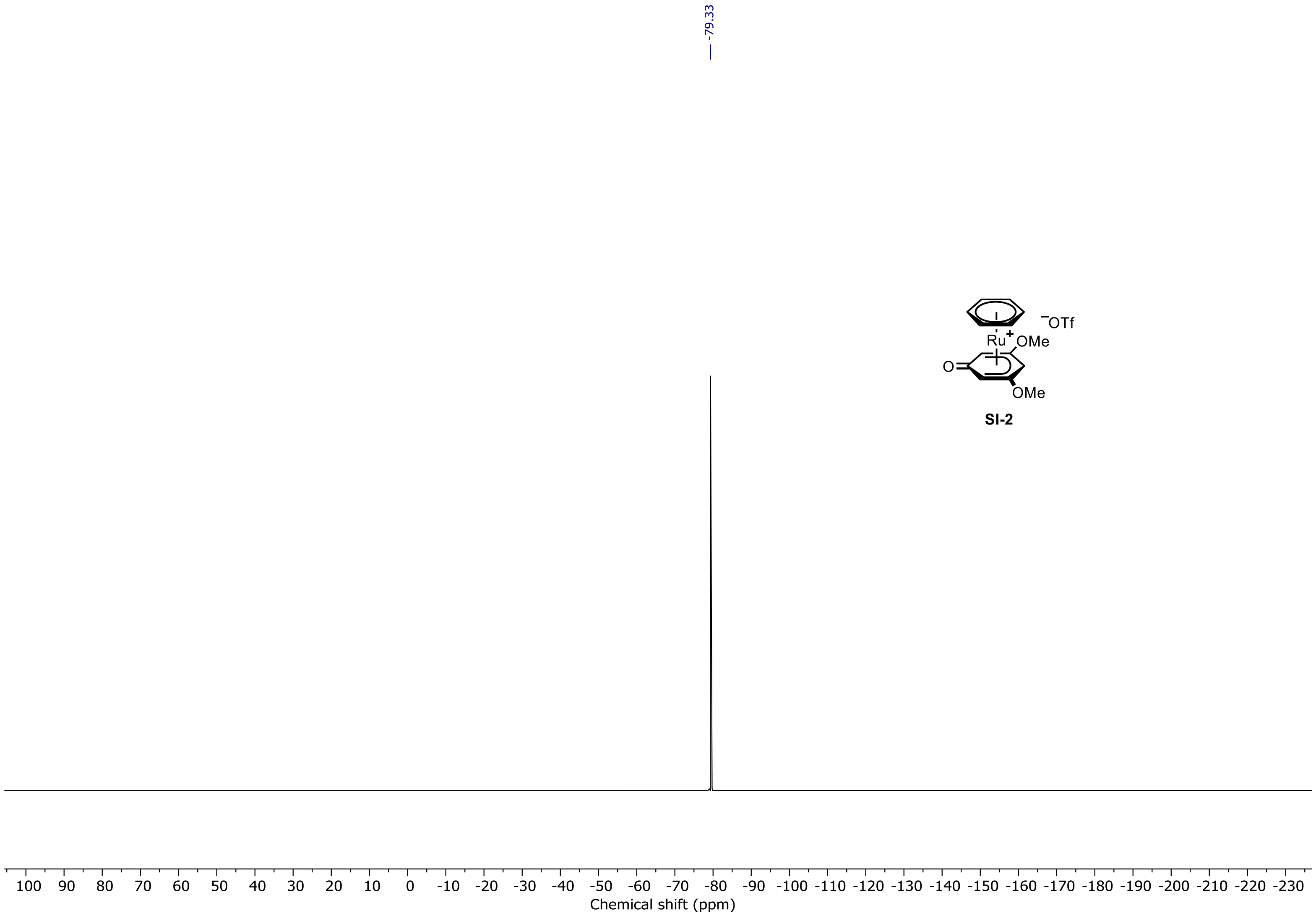

SI-2

^{1}H NMR of [η^6-benzene-η^5-(1-phenoxo-3,4,5-trimethyl)Ru](OTf) (SI-3)

CD_3CN, 600 MHz, 23 °C.

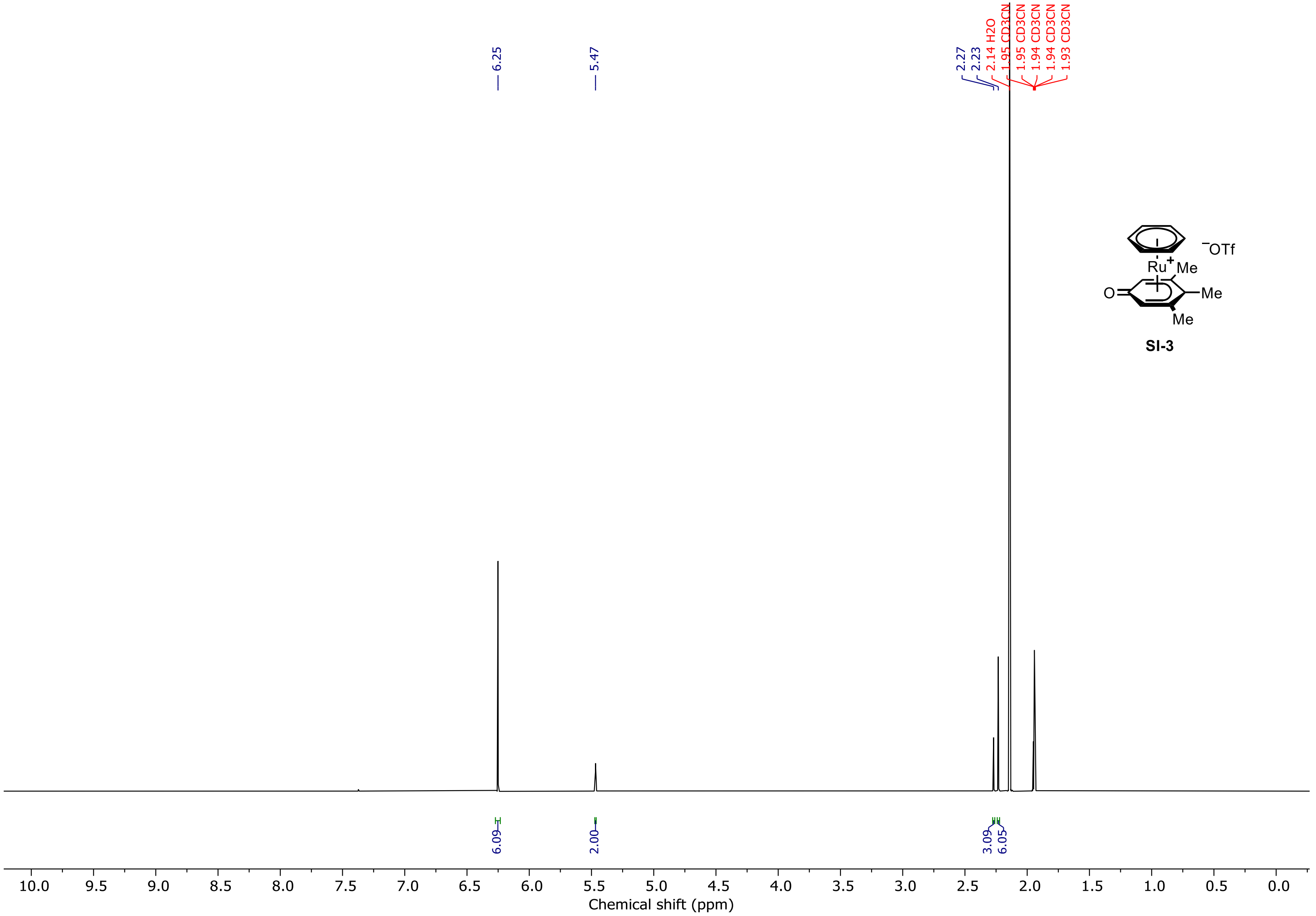

^{13}C NMR of [η^6-benzene-η^5-(1-phenoxo-3,4,5-trimethyl)Ru](OTf) (SI-3)

CD$_3$CN, 151 MHz, 23 °C.

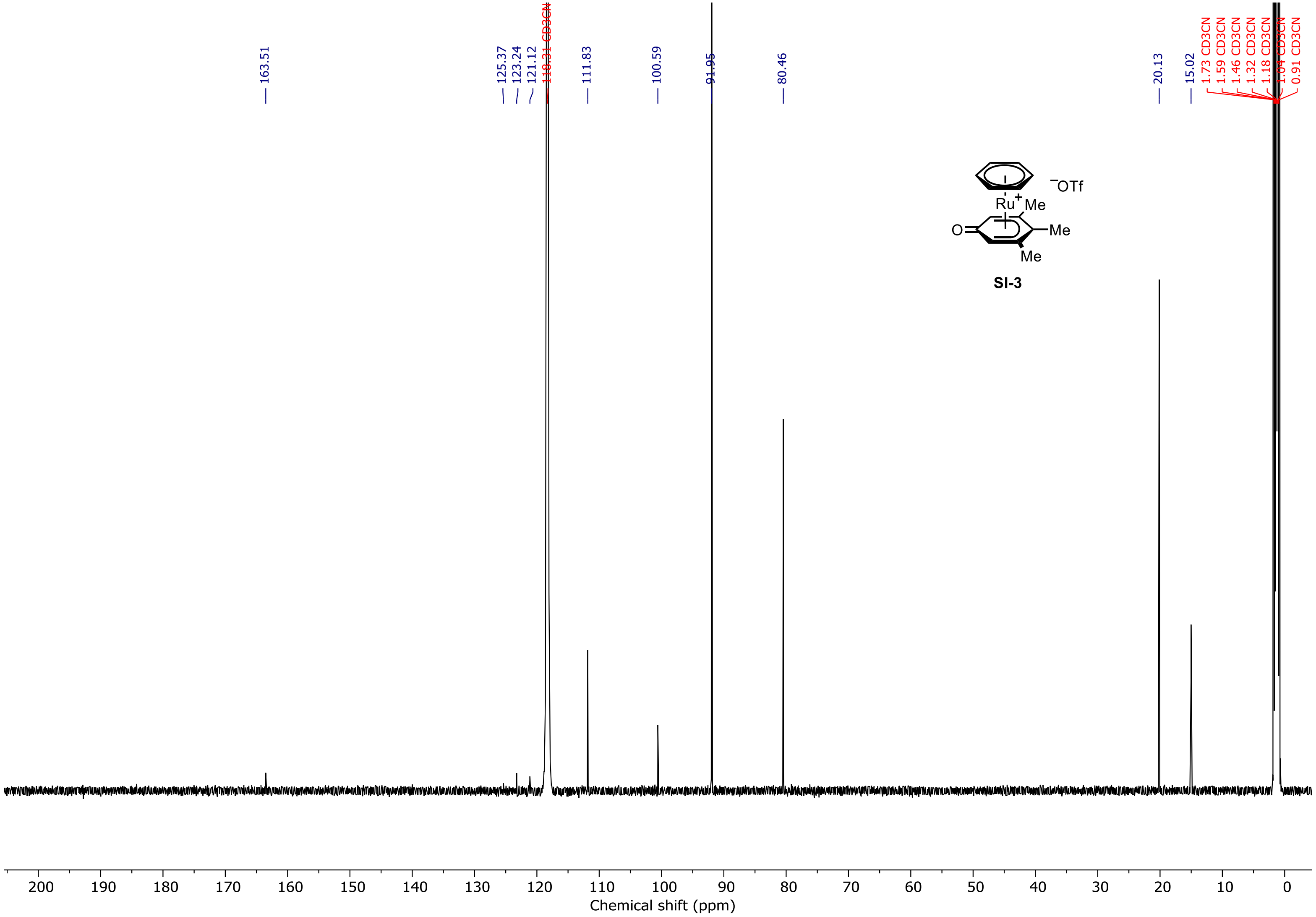

^{19}F NMR of [η^6-benzene-η^5-(1-phenoxo-3,4,5-trimethyl)Ru](OTf) (SI-3)

CD_3CN, 470 MHz, 23 °C.

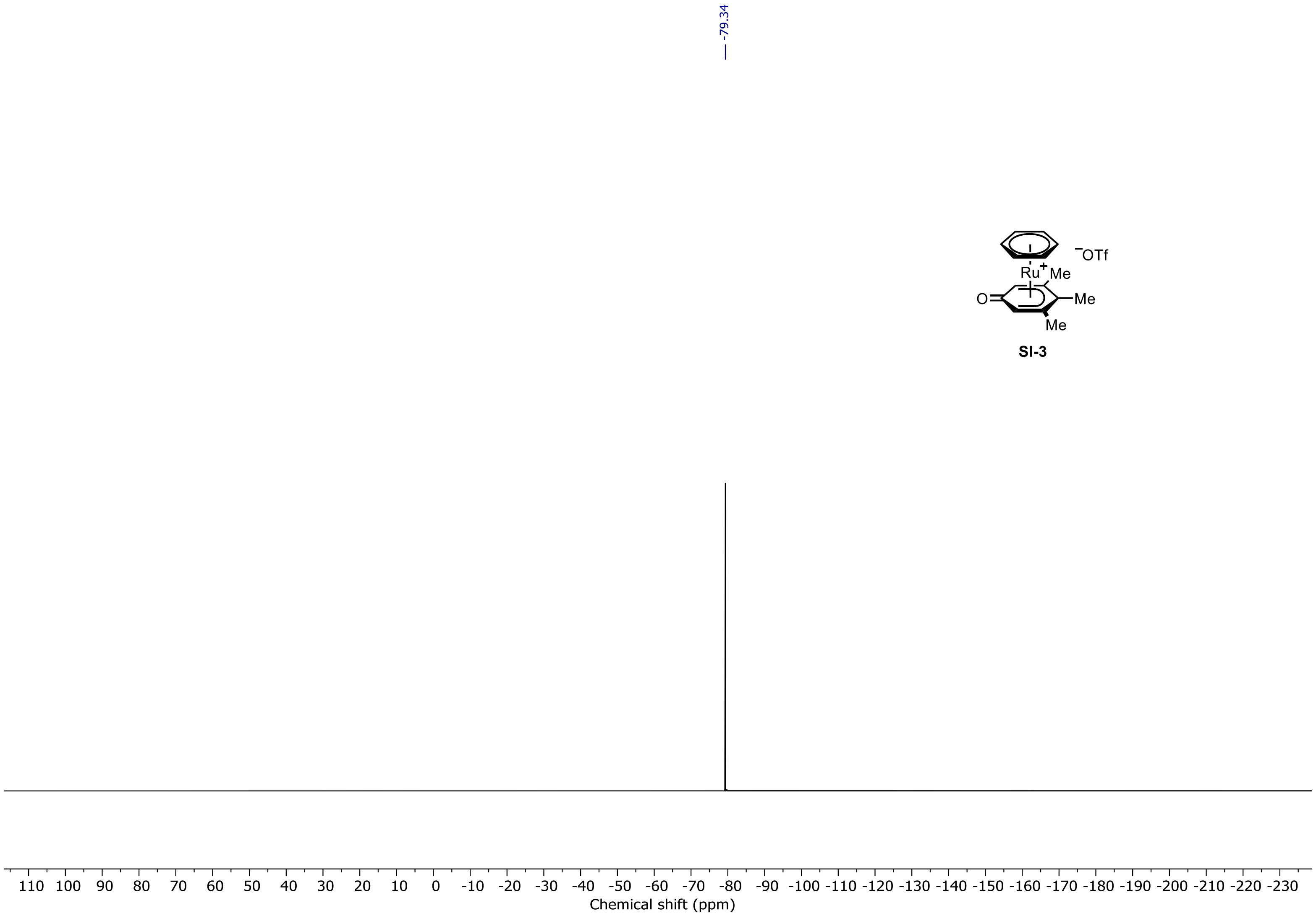

SI-3

^{1}H NMR of [η^6-benzene-η^5-(1-phenoxo-2,4,6-trimethyl)Ru](OTf) (SI-4)

CD$_3$OD, 600 MHz, 23 °C.

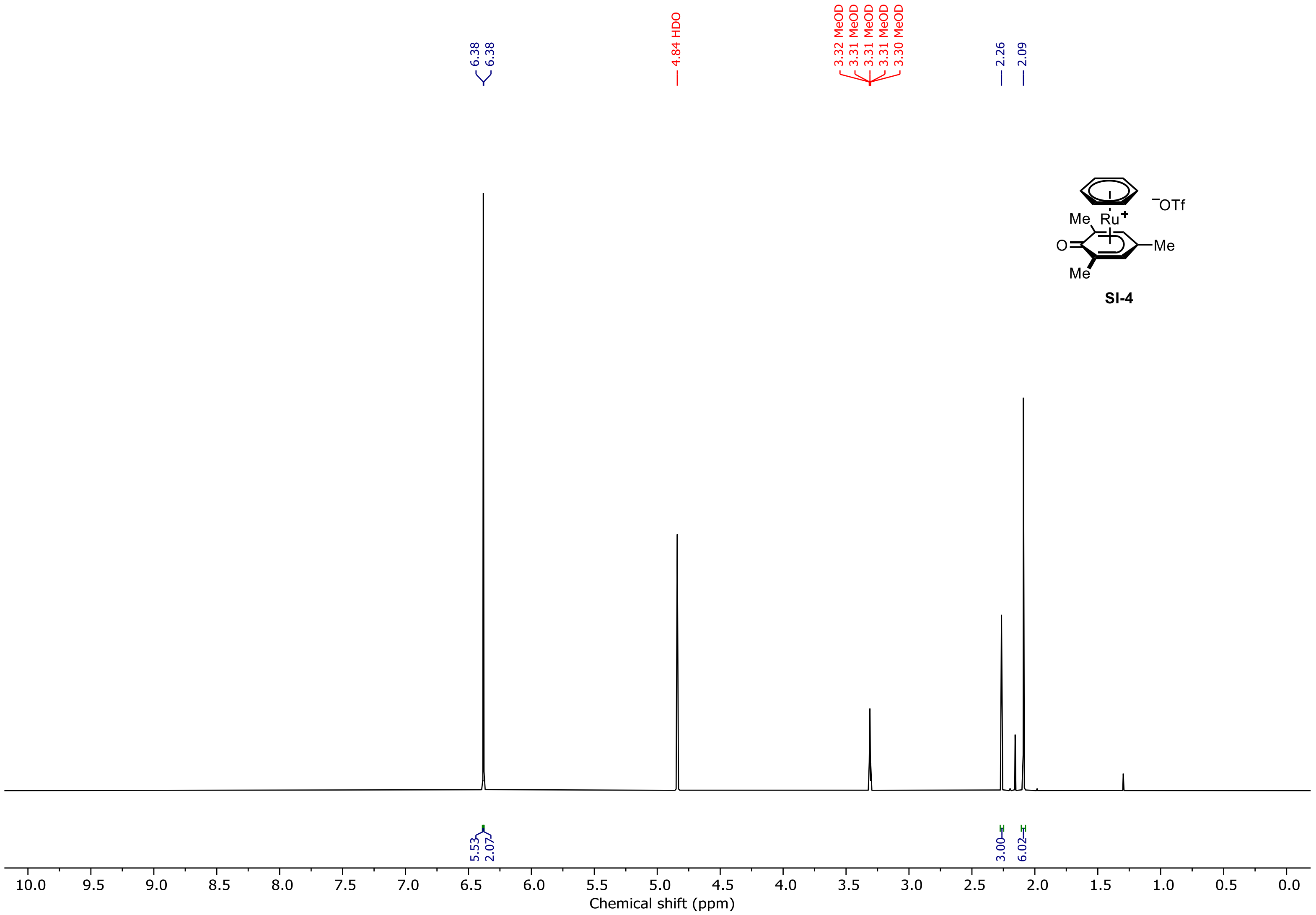

^{13}C NMR of [η^6-benzene-η^5-(1-phenoxo-2,4,6-trimethyl)Ru](OTf) (SI-4)

CD_3OD, 151 MHz, 23 °C.

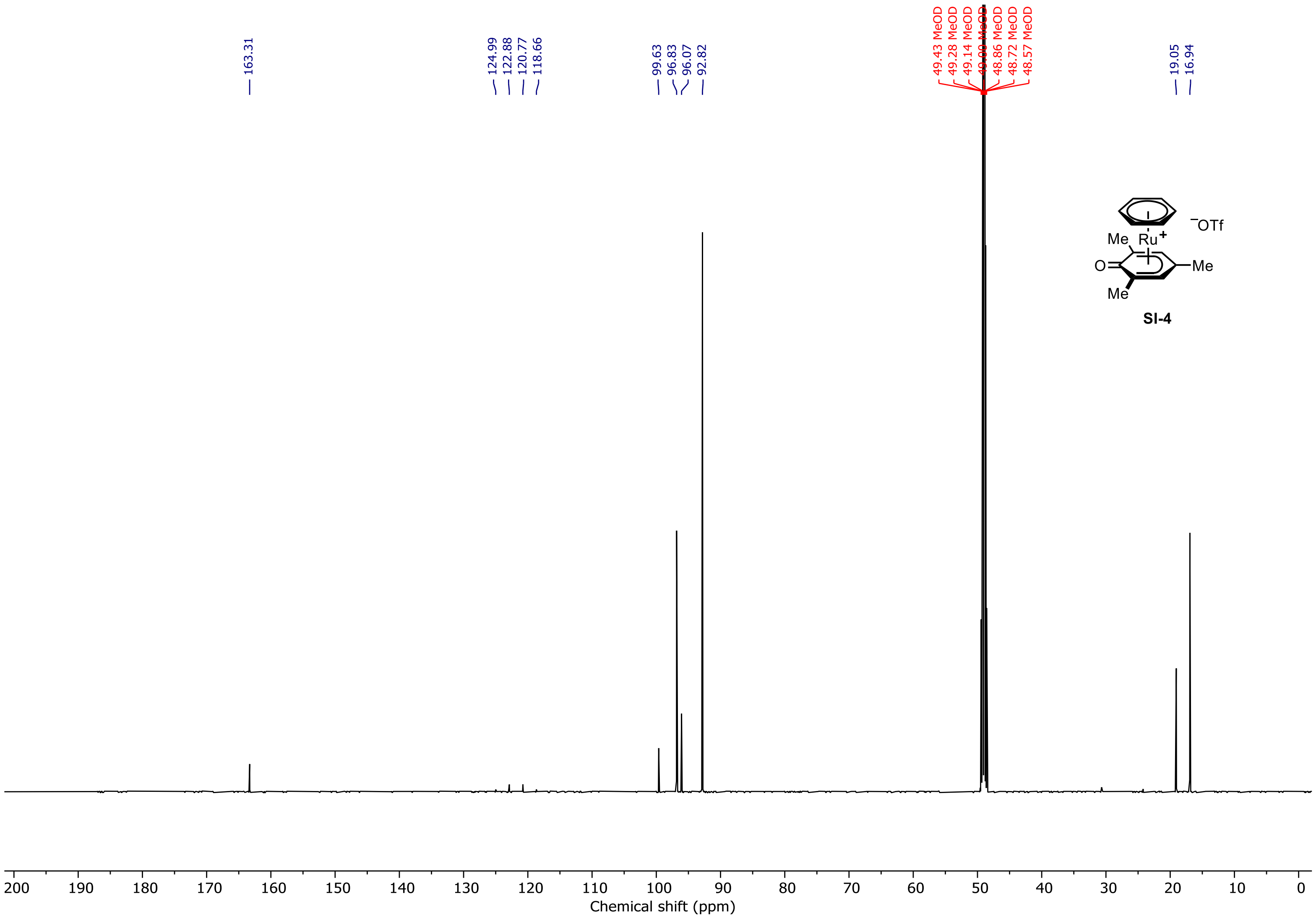

^{19}F NMR of [η^6-benzene-η^5-(1-phenoxo-2,4,6-trimethyl)Ru](OTf) (SI-4)

CD_3OD, 470 MHz, 23 °C.

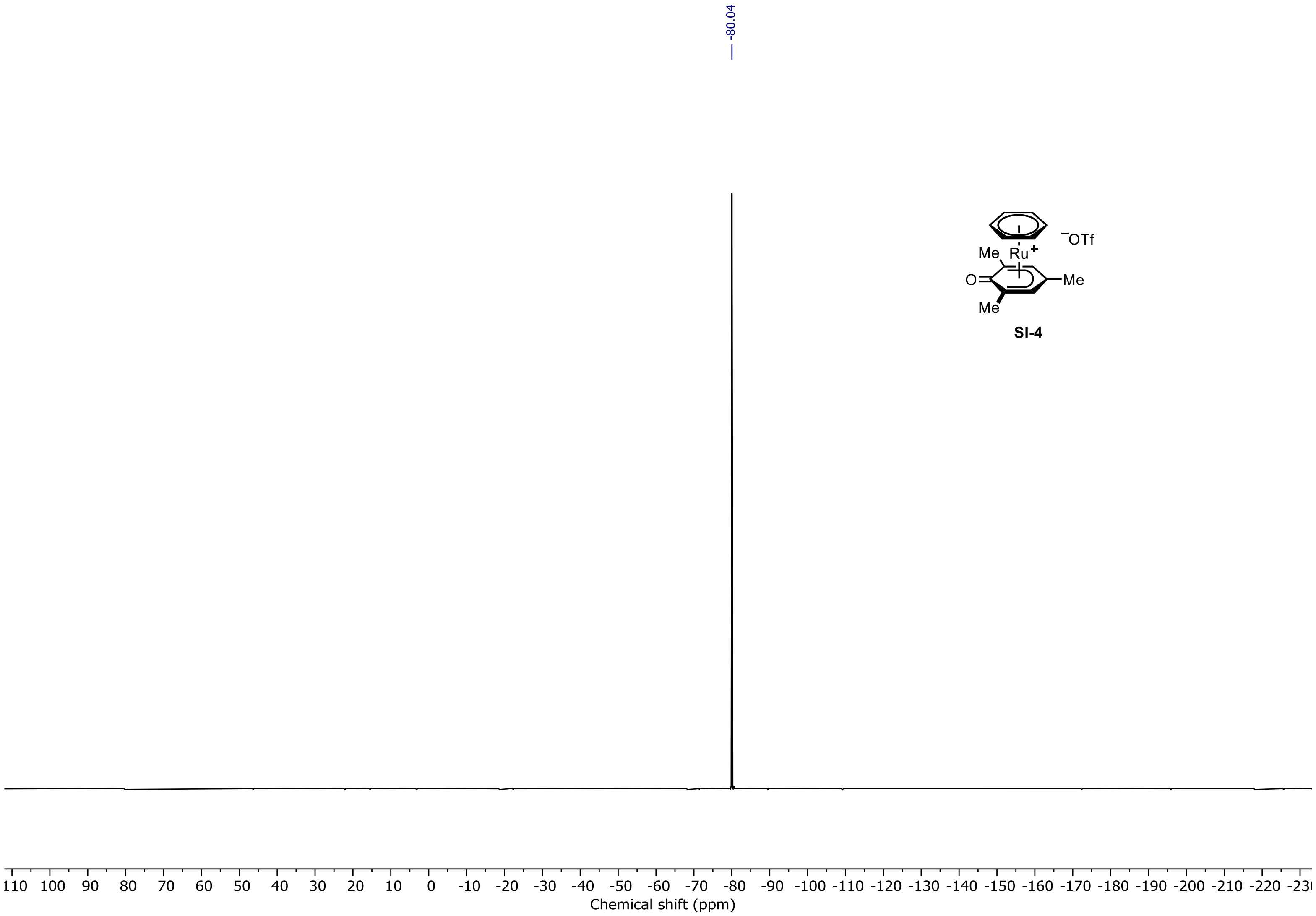

^{1}H NMR of [η^6-benzene-η^5-(1-phenoxo-2,6-dimethoxy)Ru](OTf) (SI-5)

CD_3OD, 600 MHz, 23 °C.

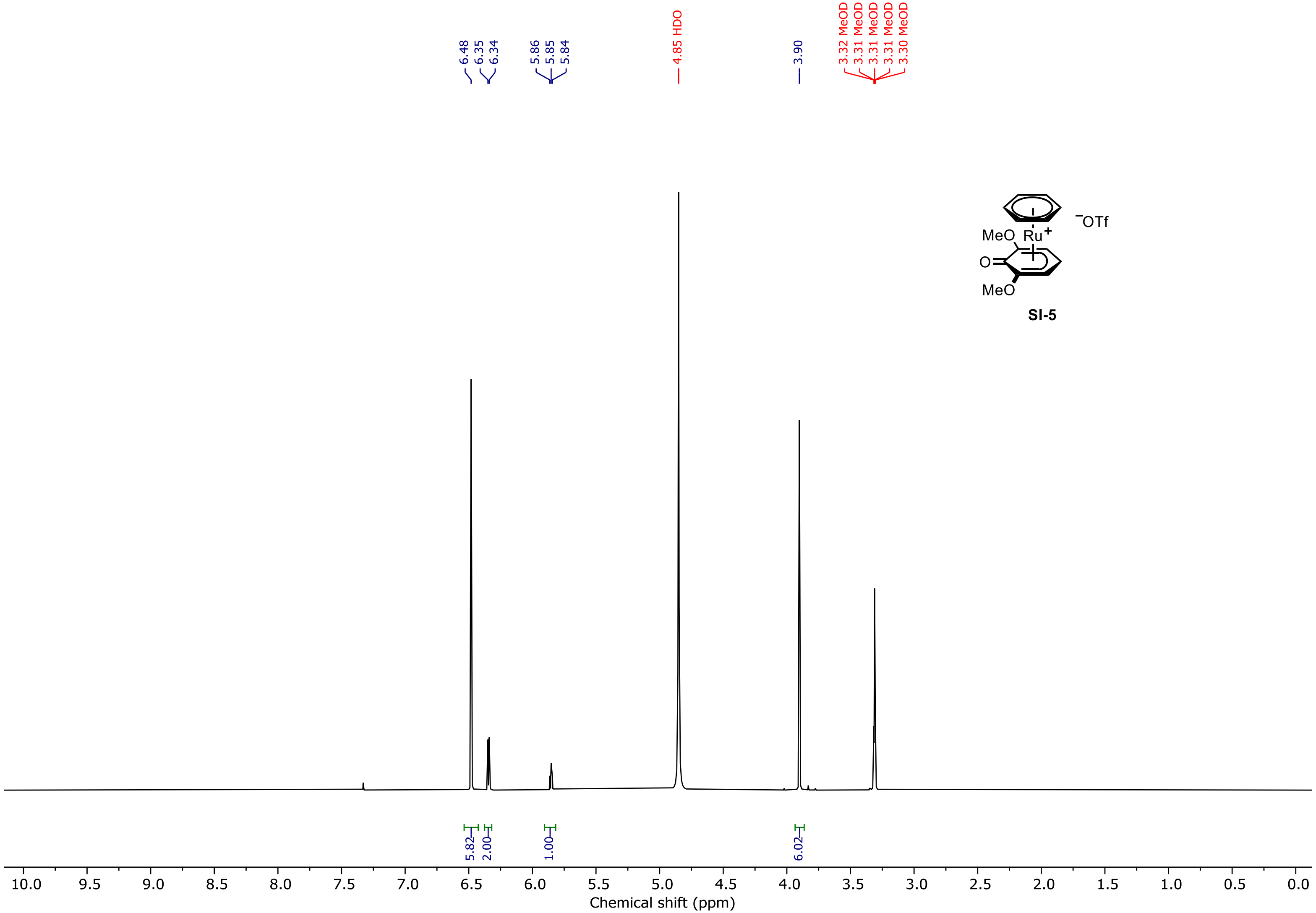

SI-5

^{13}C NMR of [η^6-benzene-η^5-(1-phenoxo-2,6-dimethoxy)Ru](OTf) (SI-5)

CD_3OD, 151 MHz, 23 °C.

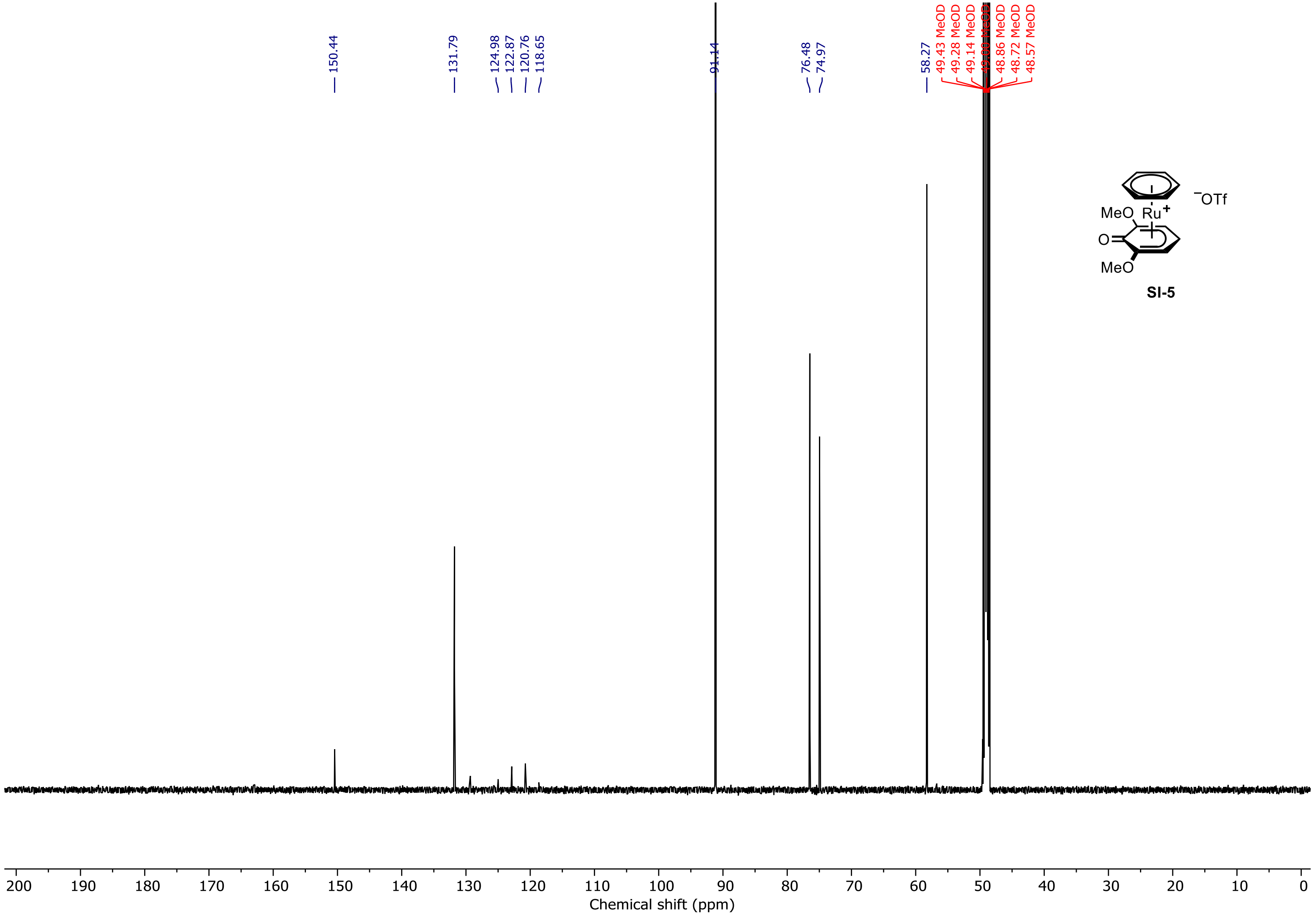

SI-5

^{19}F NMR of [η^6-benzene-η^5-(1-phenoxo-2,6-dimethoxy)Ru](OTf) (SI-5)

CD_3OD, 470 MHz, 23 °C.

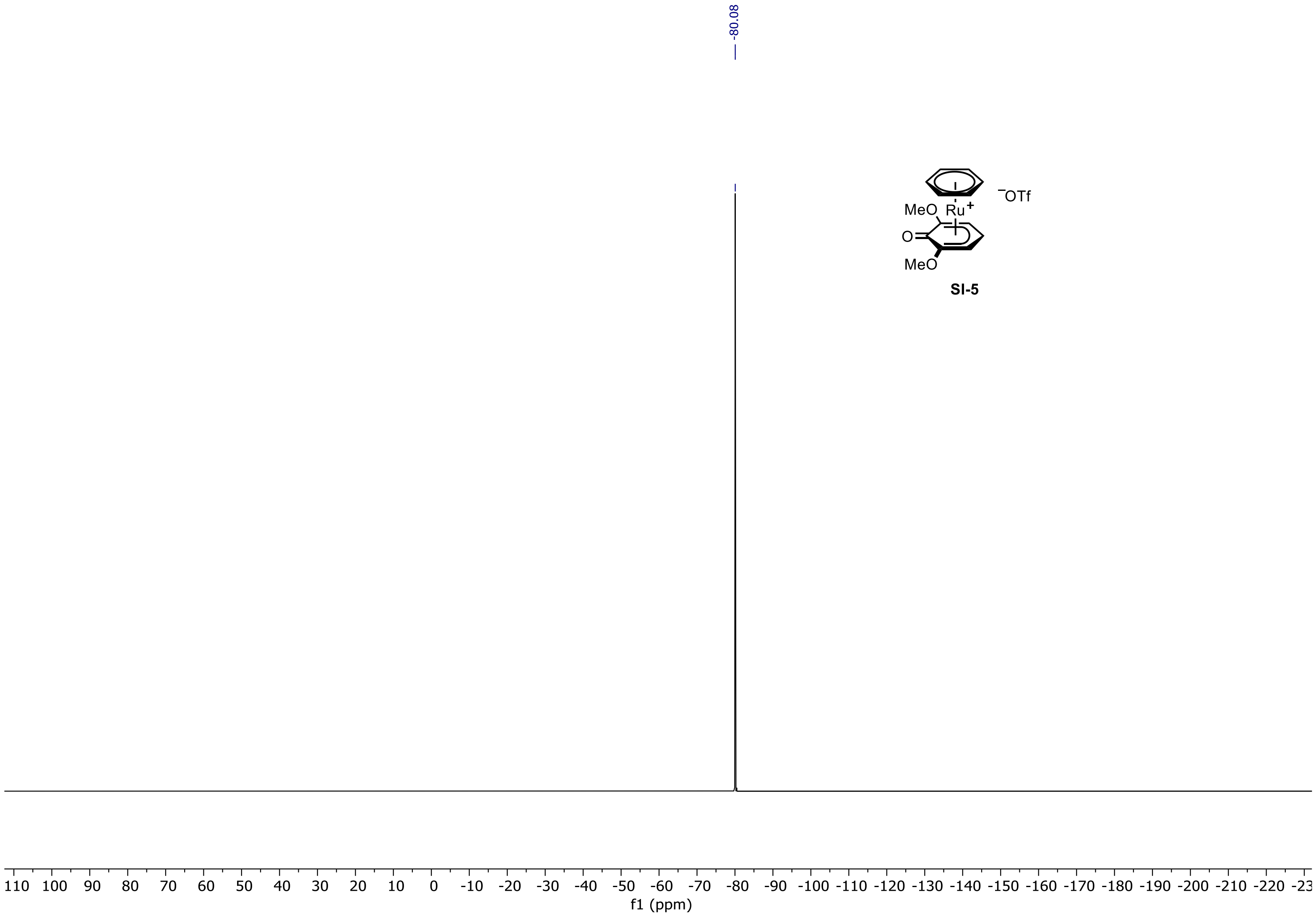

^{1}H NMR of [η^6-benzene-η^5-(4-*t*Bu-1-phenoxo)Ru](OTf) (SI-6)

CD_3OD, 600 MHz, 23 °C.

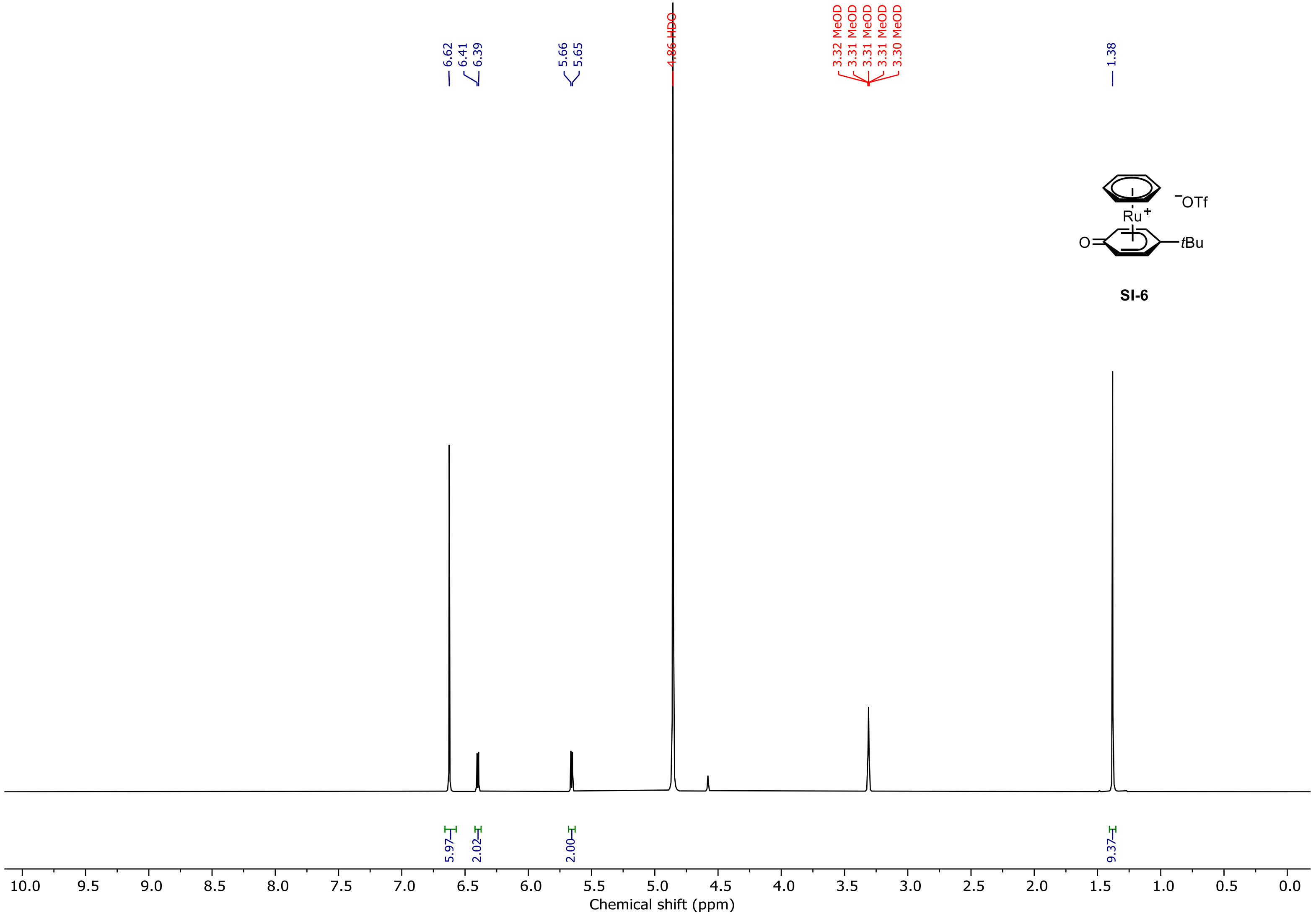

^{13}C NMR of [η^6-benzene-η^5-(4-*t*Bu-1-phenoxo)Ru](OTf) (SI-6)

CD_3OD, 151 MHz, 23 °C.

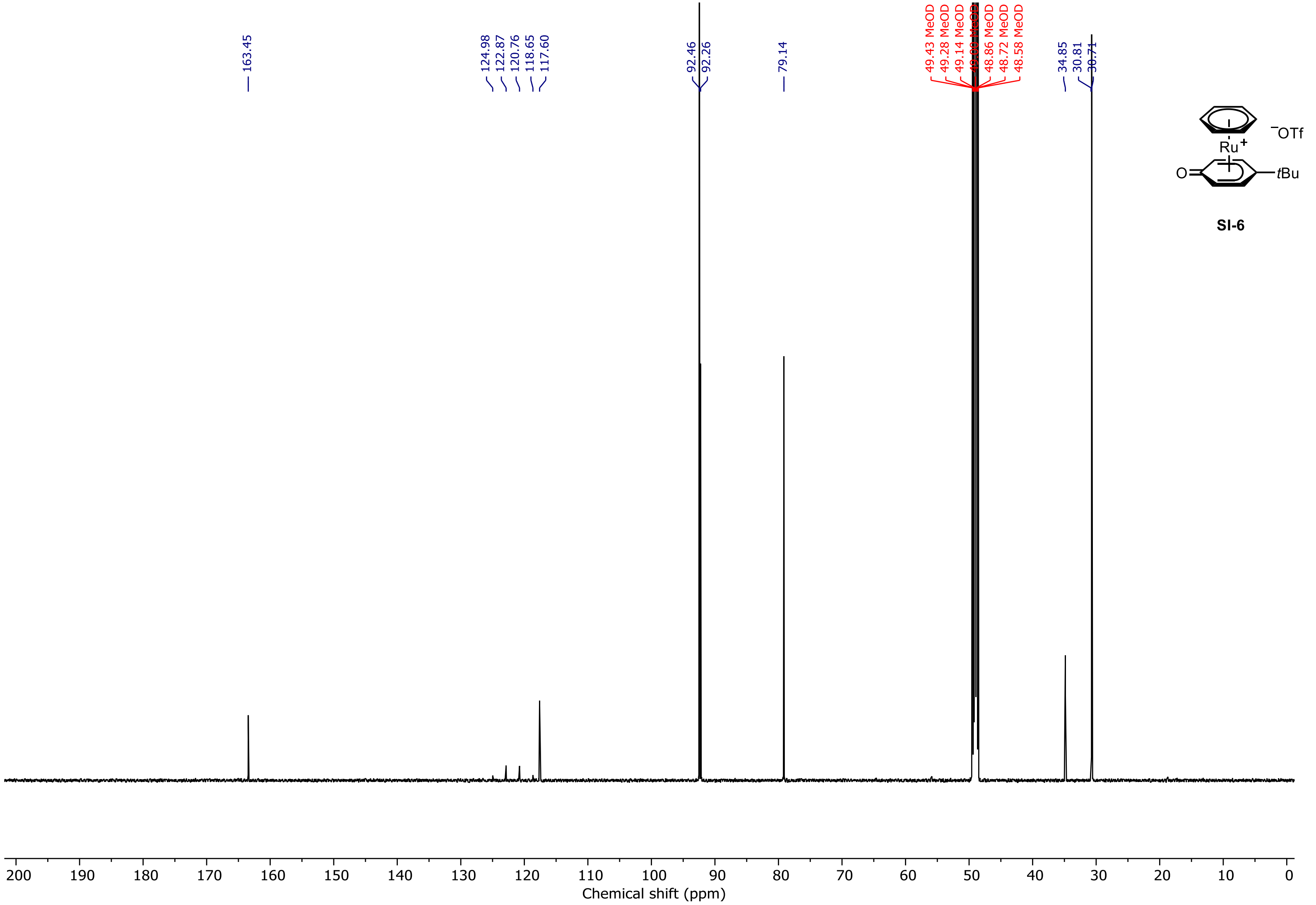

^{19}F NMR of [η^6-benzene-η^5-(4-*t*Bu-1-phenoxo)Ru](OTf) (SI-6)

CD_3OD, 470 MHz, 23 °C.

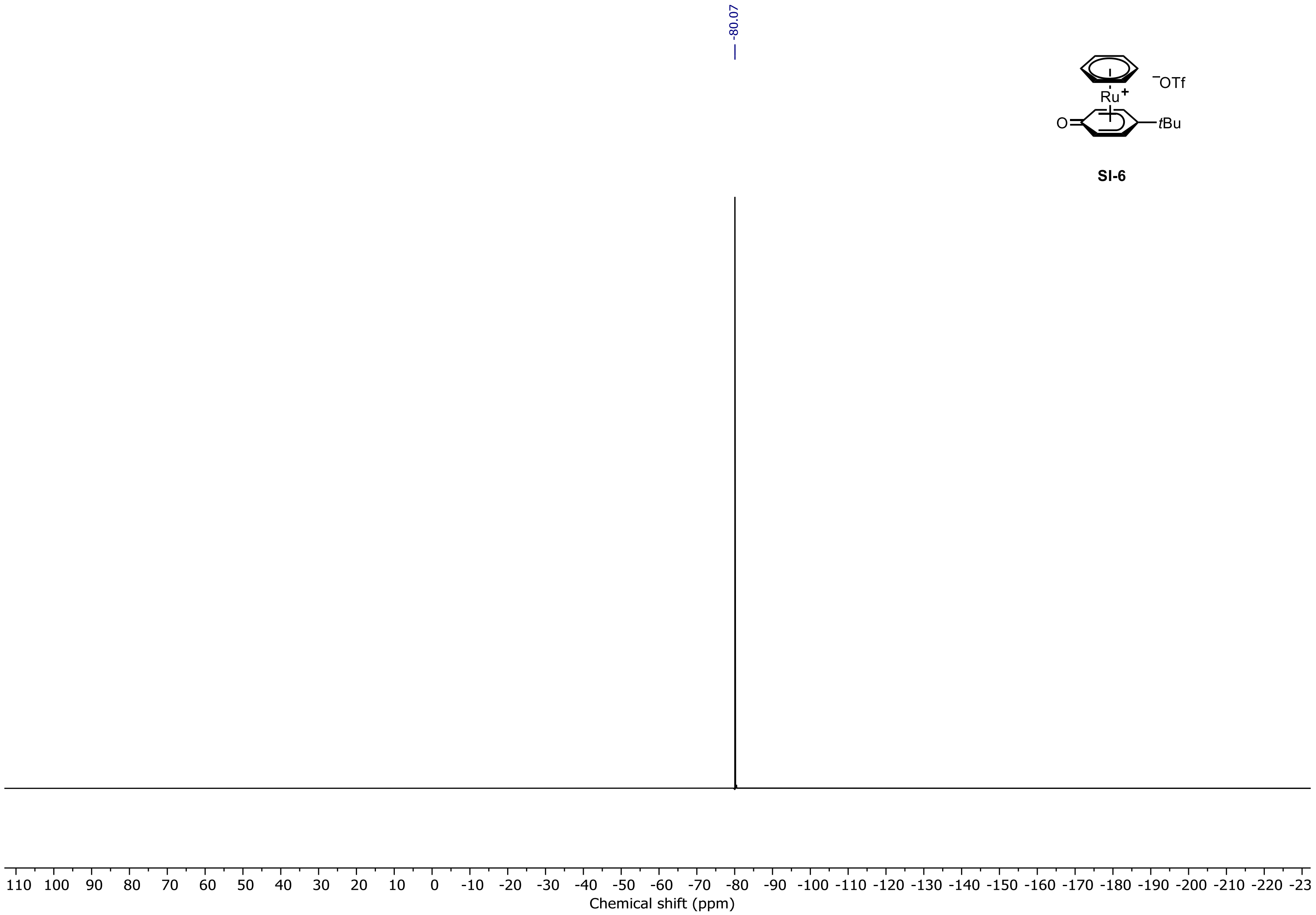

^{1}H NMR of [η^6-benzene-η^5-(4-amino-1-phenoxo)Ru](OTf) (SI-7)

CD_3OD, 600 MHz, 23 °C.

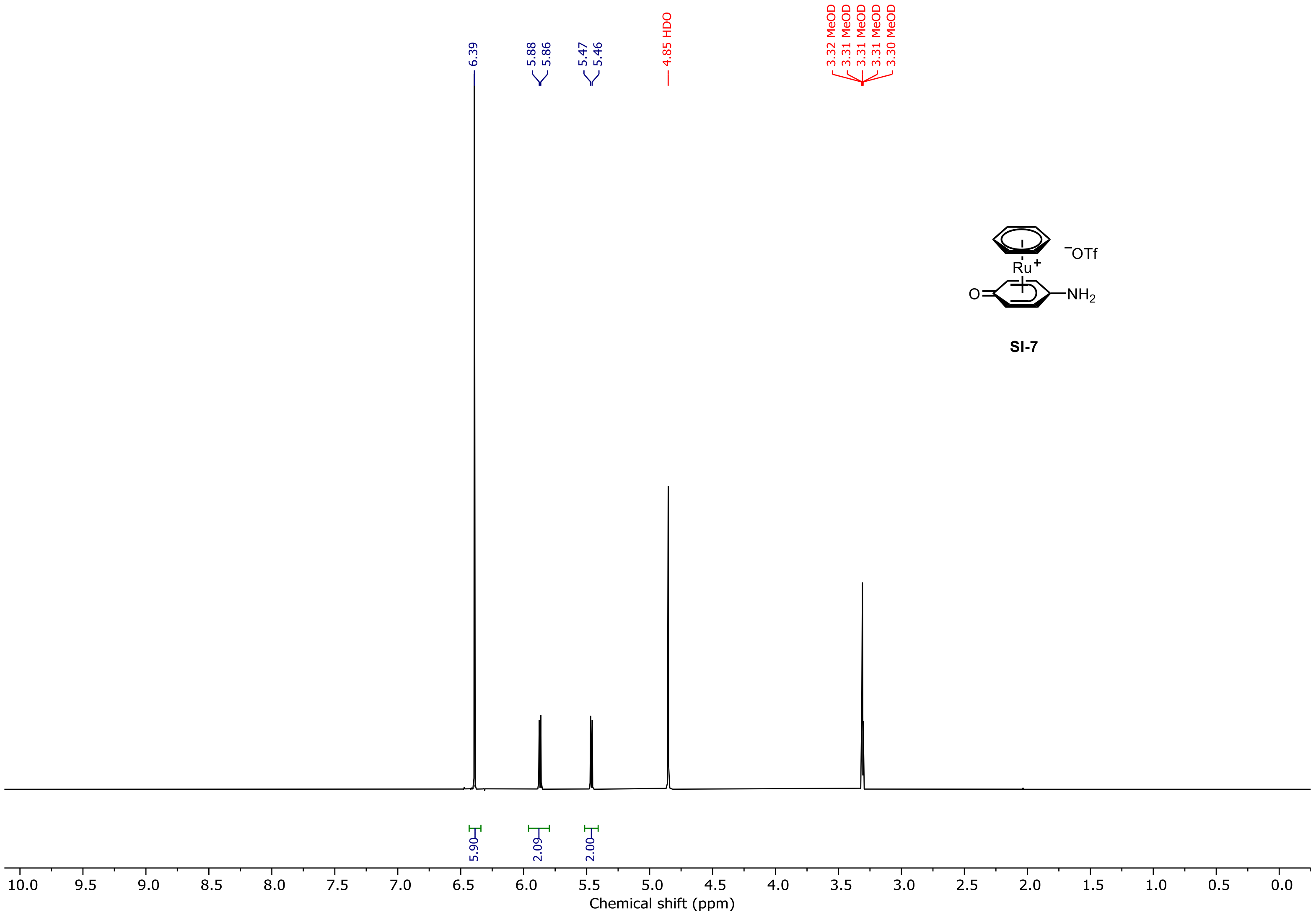

SI-7

^{13}C NMR of [η^6-benzene-η^5-(4-amino-1-phenoxo)Ru](OTf) (SI-7)

CD$_3$OD, 151 MHz, 23 °C.

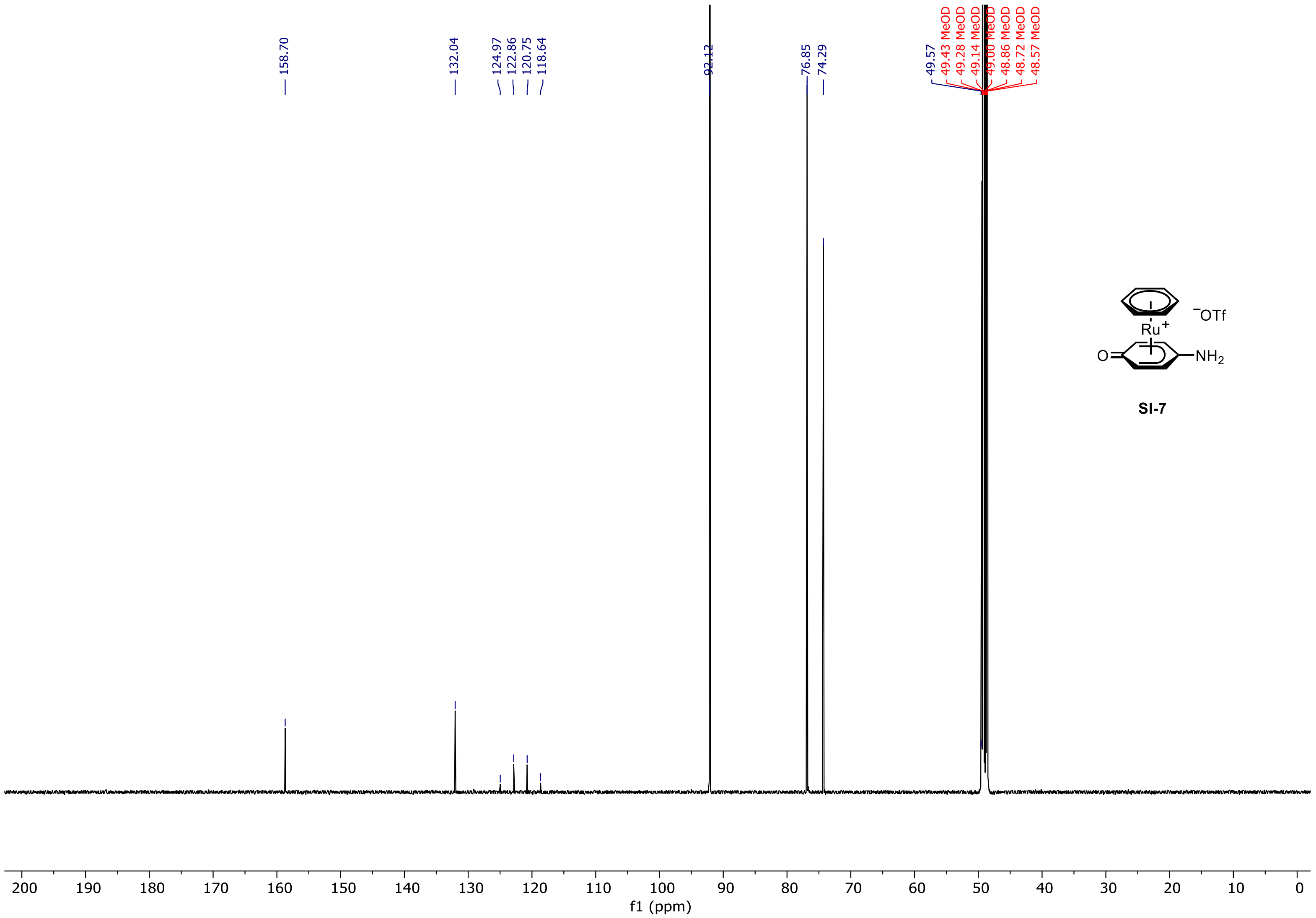

^{19}F NMR of [η^6-benzene-η^5-(4-amino-1-phenoxo)Ru](OTf) (SI-7)

CD_3OD, 470 MHz, 23 °C.

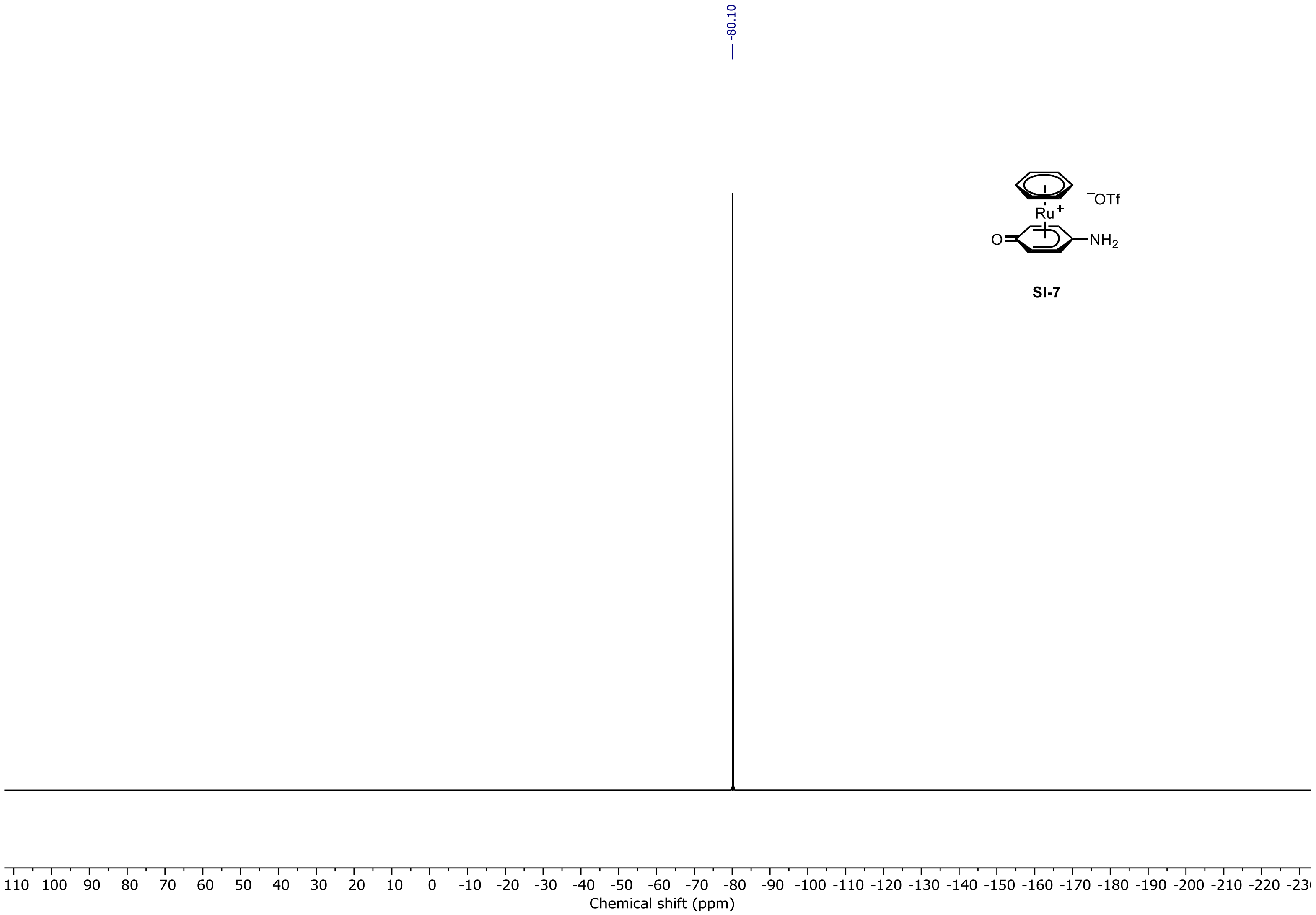

^{1}H NMR of [η^6-ethylbenzoate-η^5-(2,6-dichloro-1-phenoxo)Ru](OTf) (SI-8)

CD_3CN, 600 MHz, 23 °C.

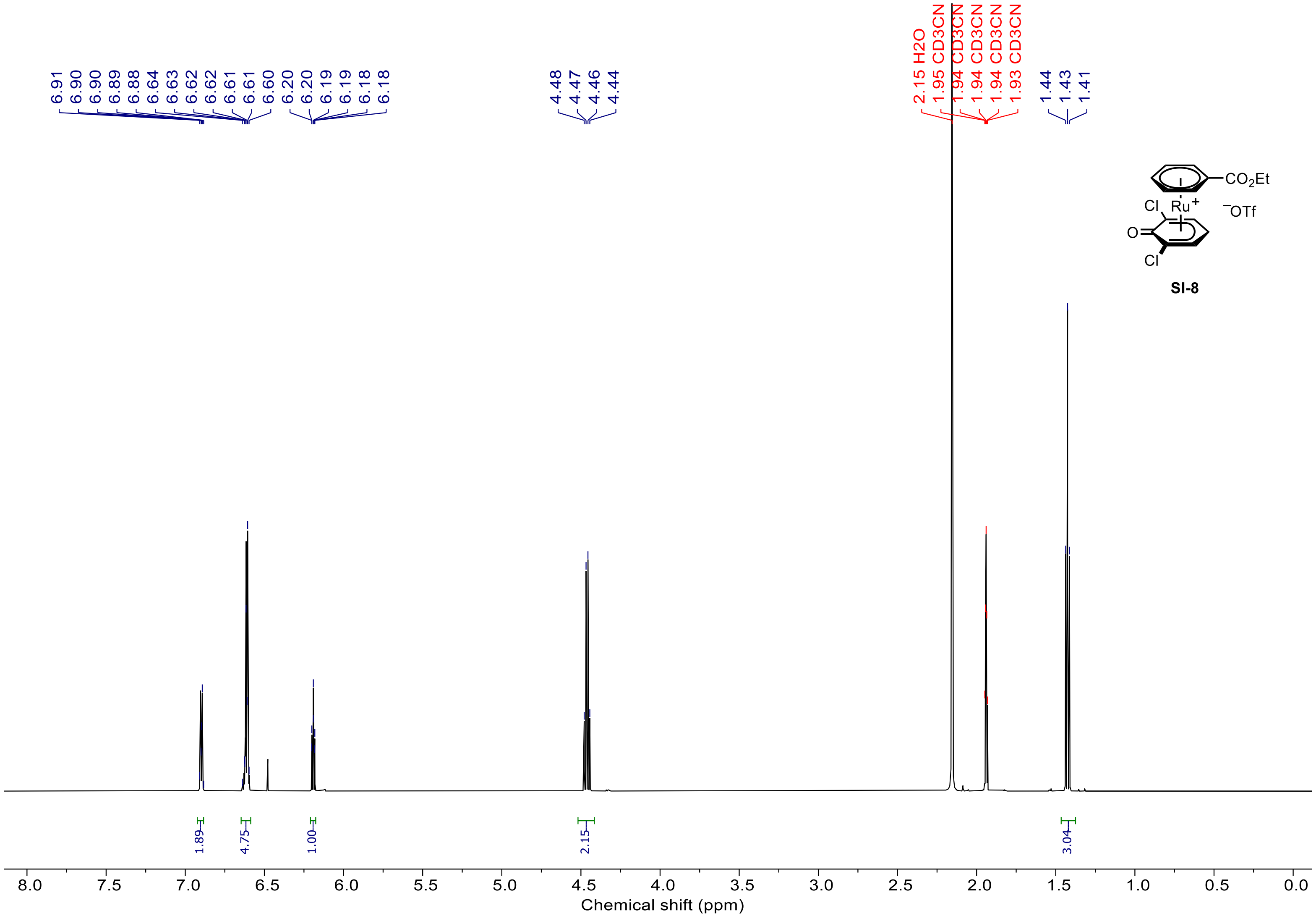

^{13}C NMR of [*η*^{6}-ethylbenzoate-*η*^{5}-(2,6-dichloro-1-phenoxo)Ru](OTf) (SI-8)

CD_3CN, 151 MHz, 23 °C.

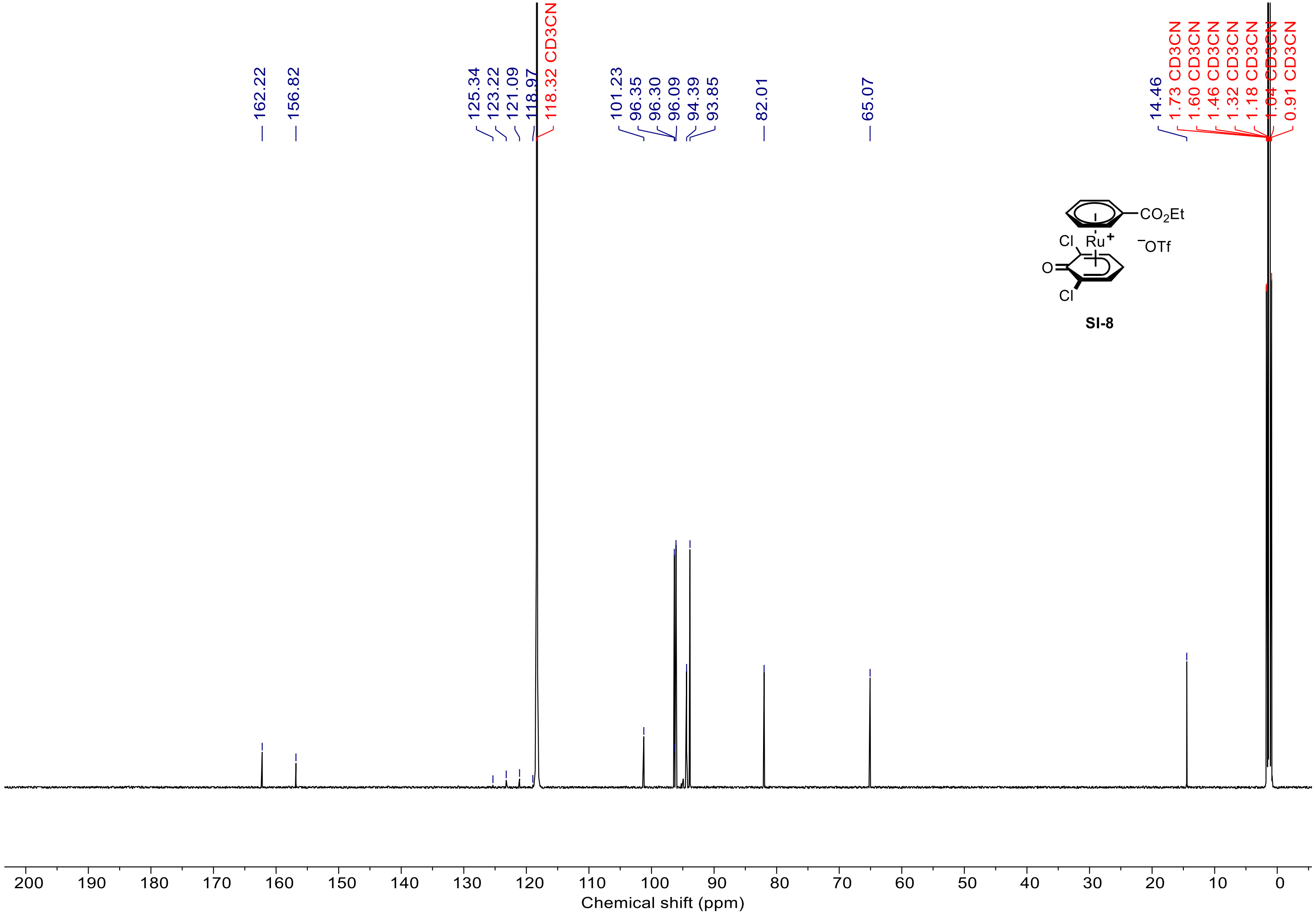

^{19}F NMR of [*η*[6]-ethylbenzoate-*η*[5]-(2,6-dichloro-1-phenoxo)Ru](OTf) (SI-8)

CD_3CN, 470 MHz, 23 °C.

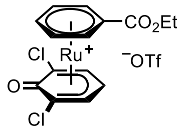

SI-8

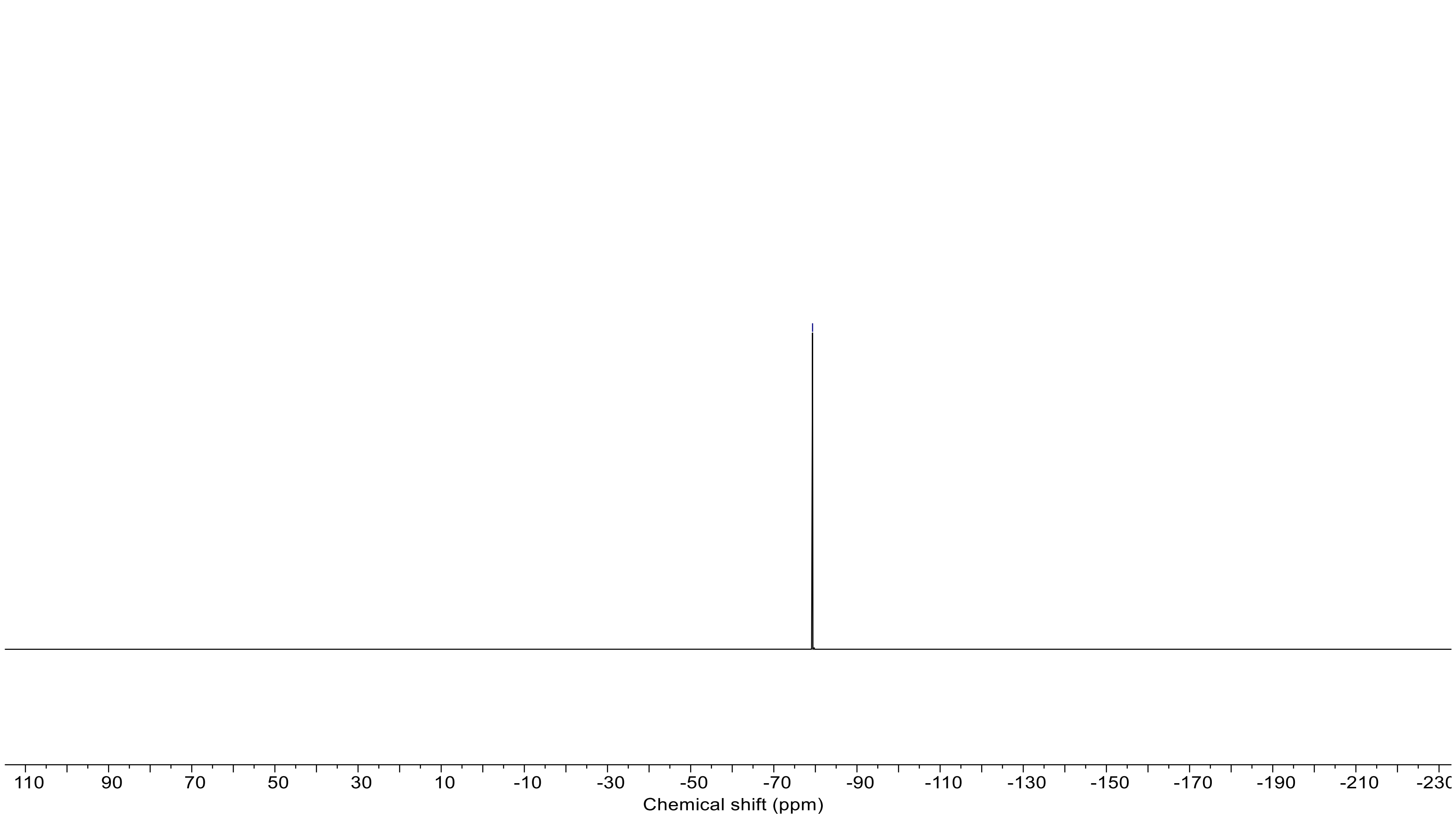

^{1}H NMR of [η^6-benzene-η^5-(2-chloro-1-phenoxo)Ru](OTf) (SI-9)

CD_3CN, 600 MHz, 23 °C.

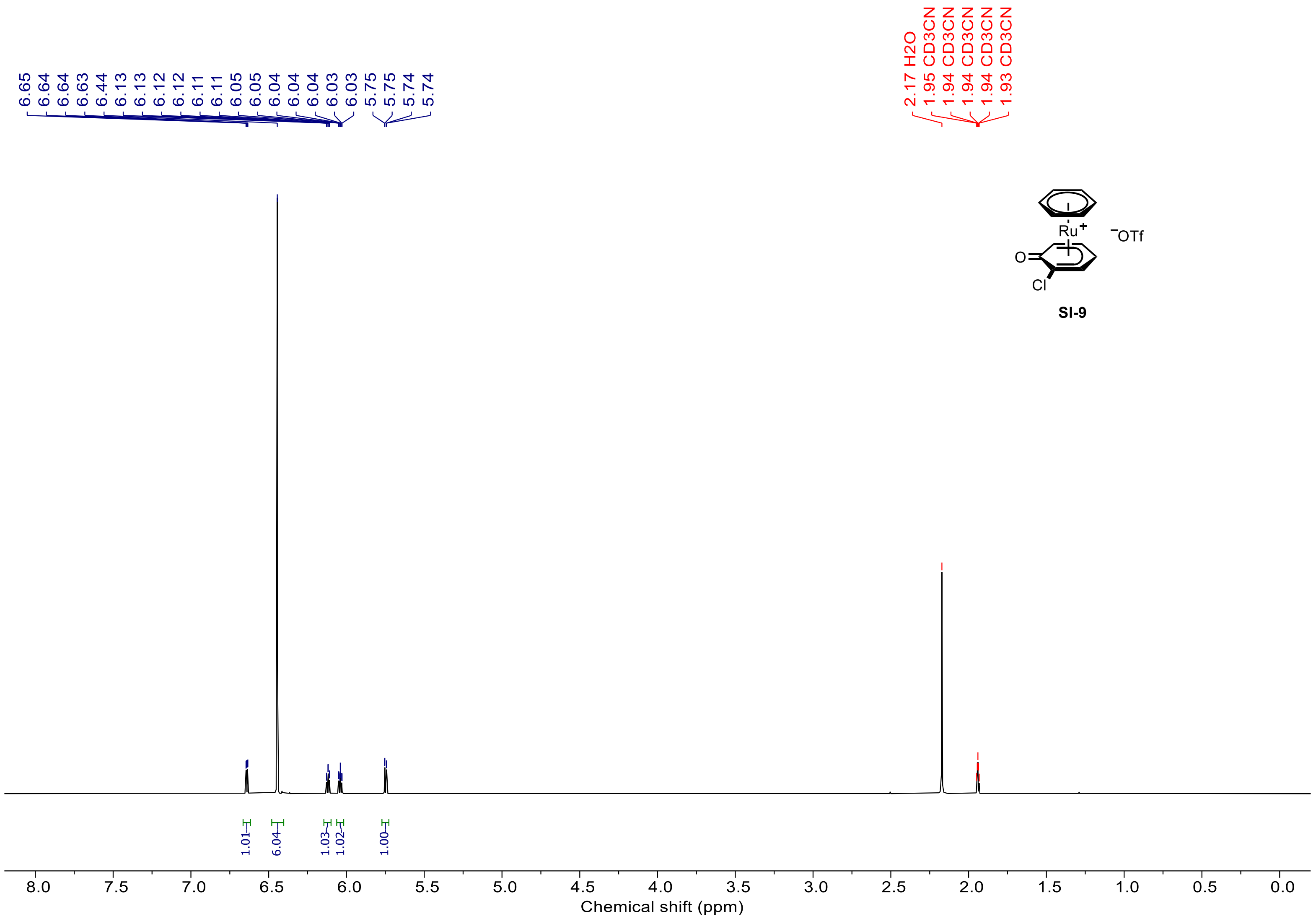

^{13}C NMR of [η^6-benzene-η^5-(2-chloro-1-phenoxo)Ru](OTf) (SI-9)

CD_3CN, 151 MHz, 23 °C.

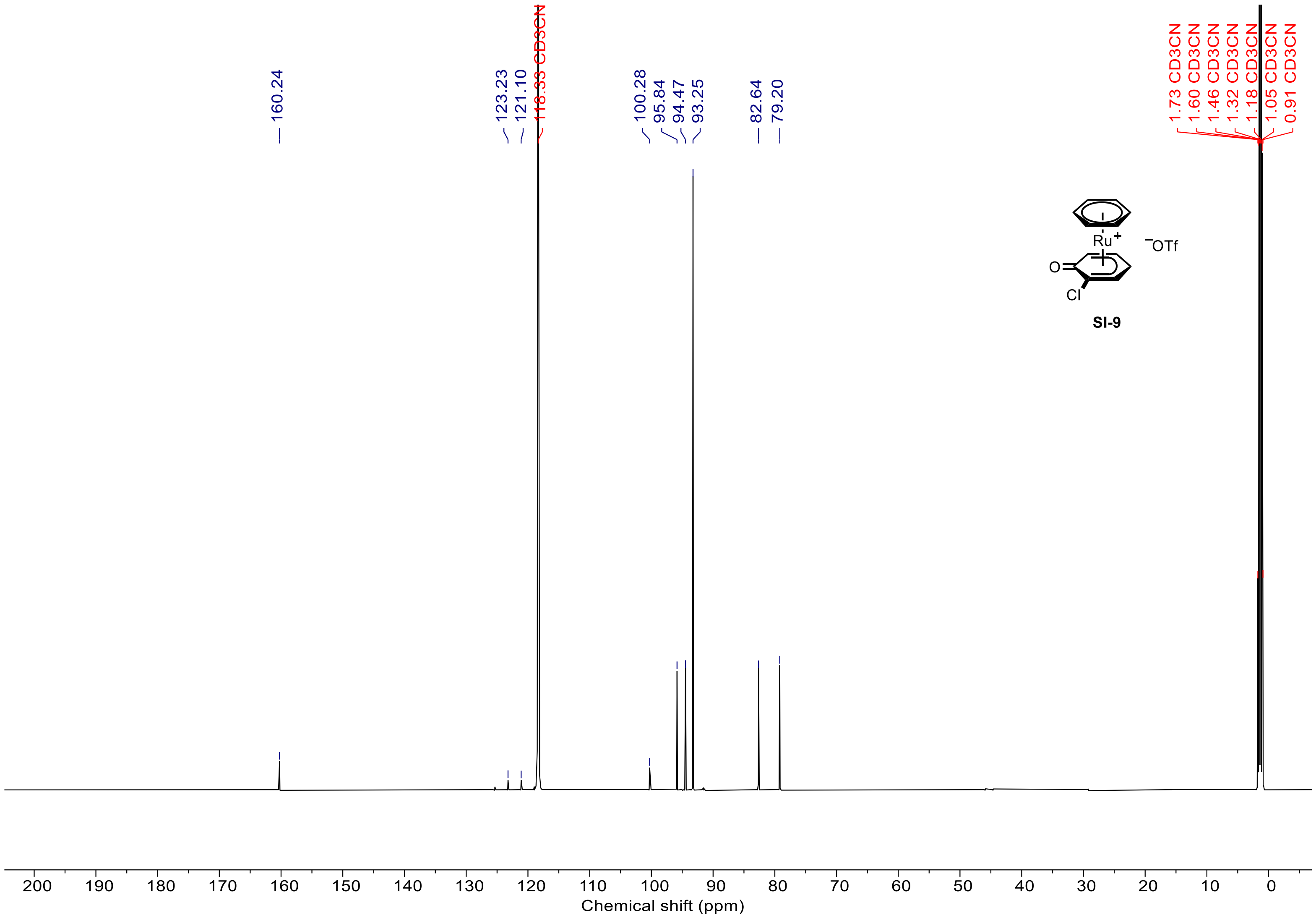

SI-9

^{19}F NMR of [η^6-benzene-η^5-(2-chloro-1-phenoxo)Ru](OTf) (SI-9)

CD_3CN, 470 MHz, 23 °C.

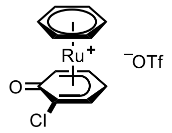

SI-9

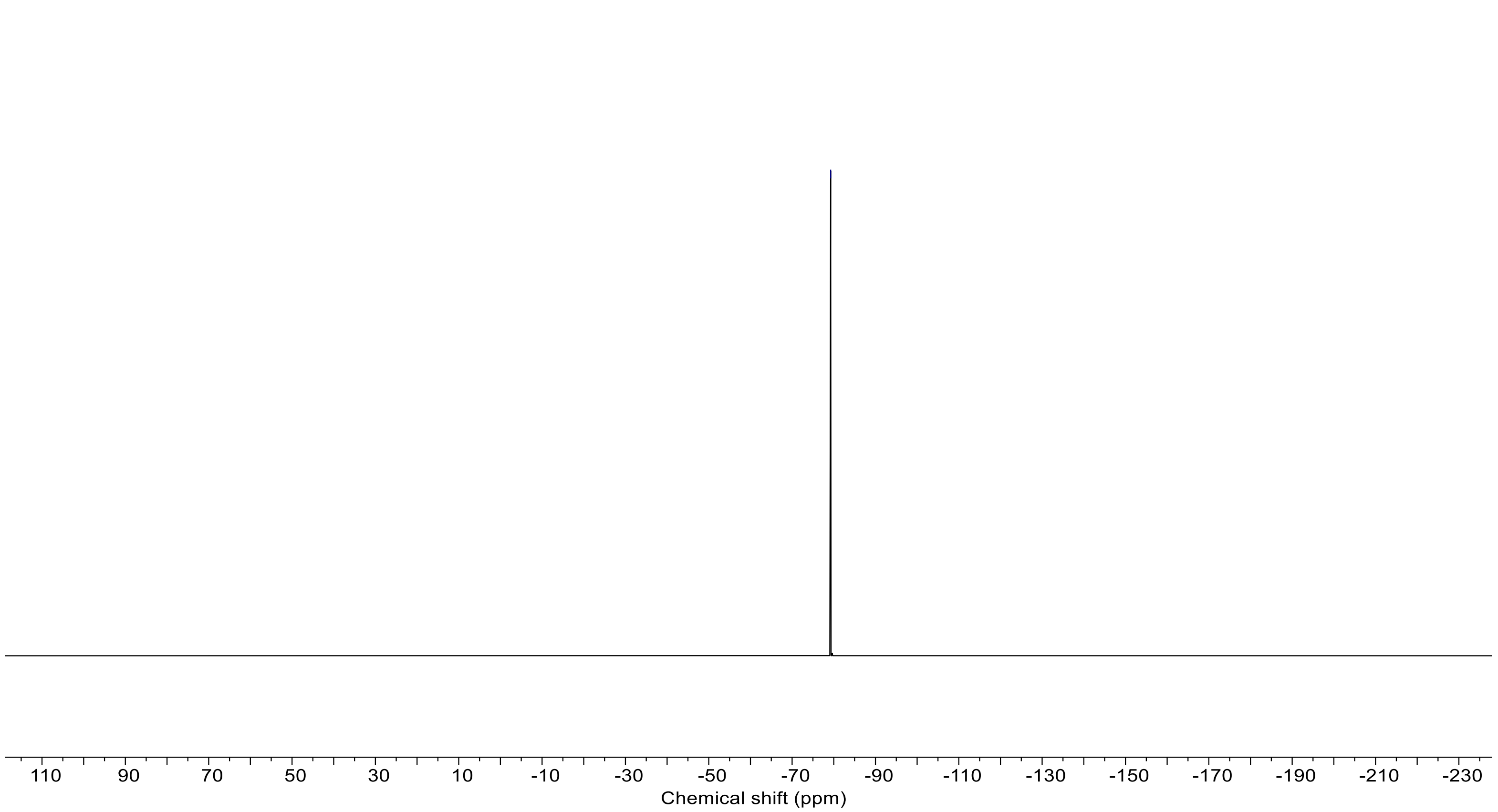

^{1}H NMR of phenyl-epiandrosterone ester (4a)

$CDCl_3$, 500 MHz, 23 °C.

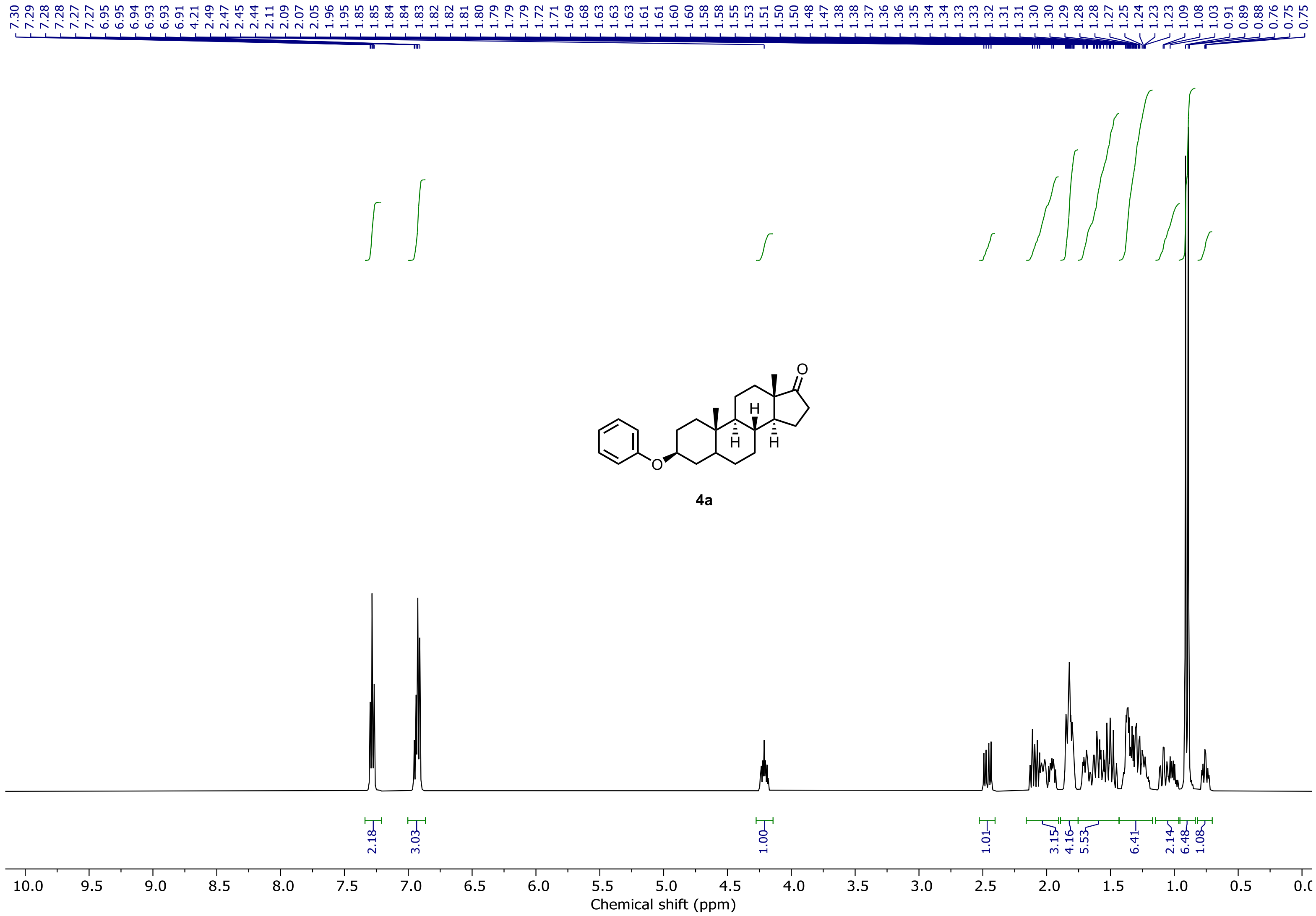

^{13}C NMR of phenyl-epiandrosterone ester (4a)

$CDCl_3$, 125 MHz, 23 °C.

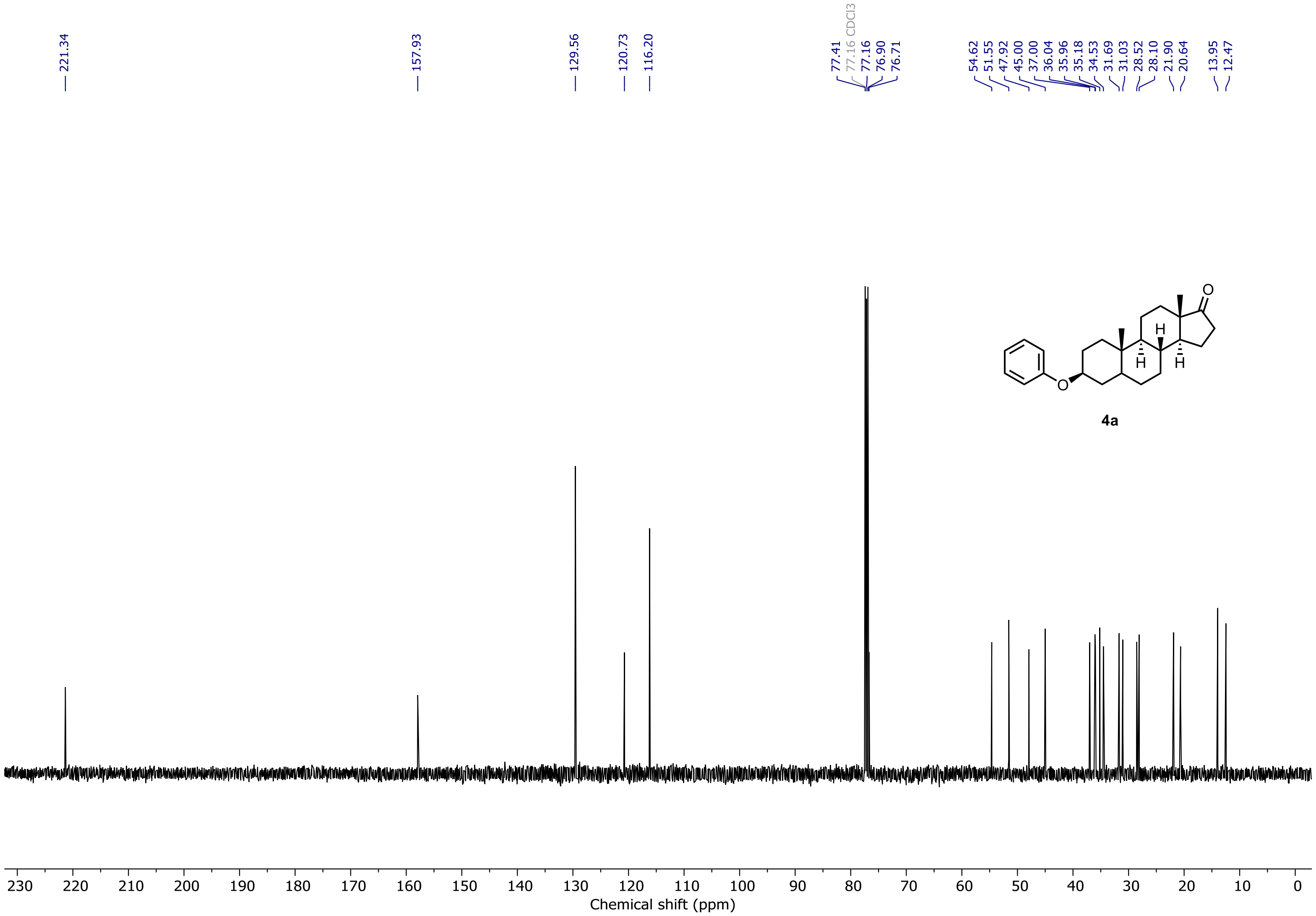

^{1}H NMR of 1-nitro-4-(phenoxymethyl)benzene (4b)

$CDCl_3$, 500 MHz, 23 °C.

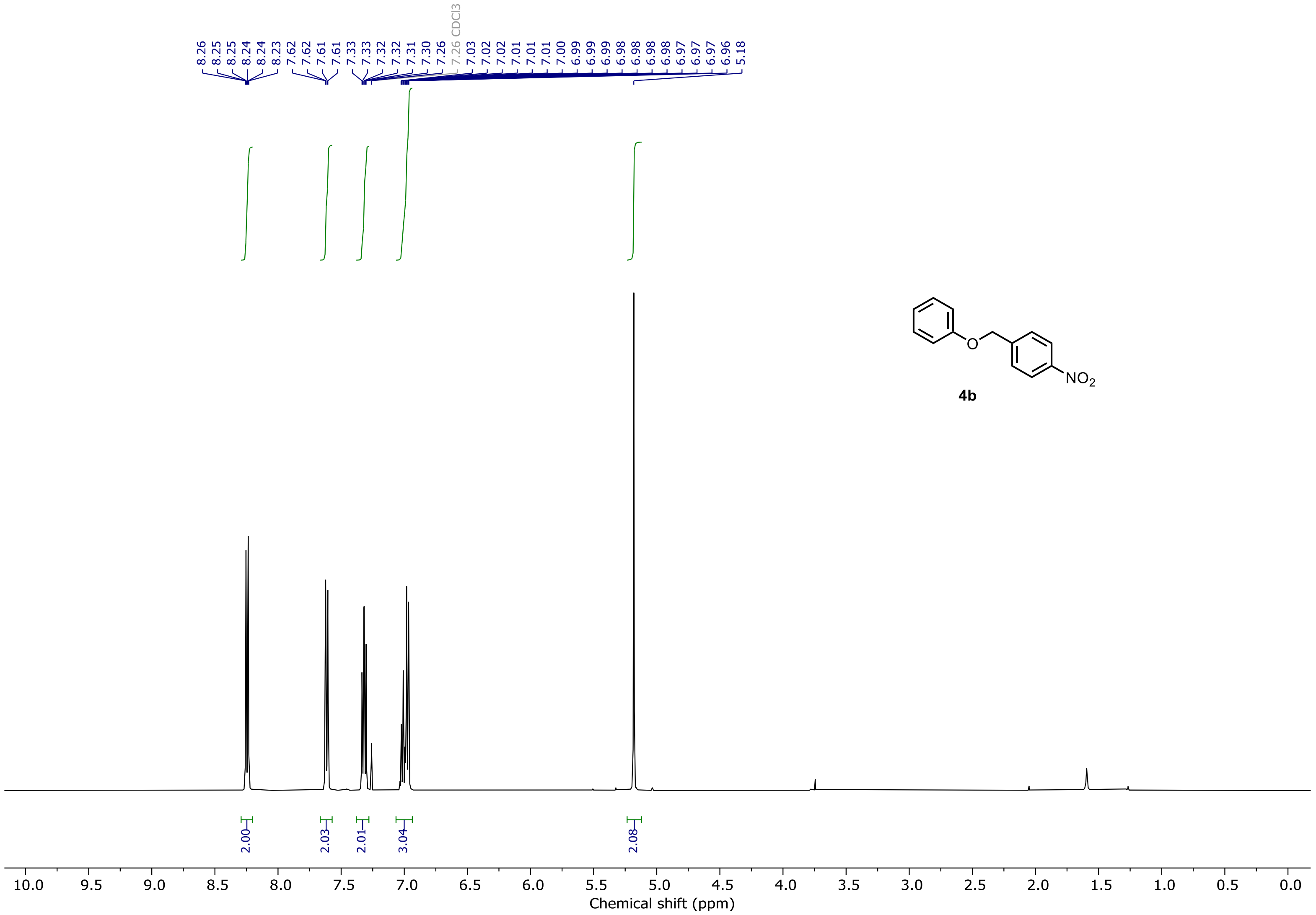

^{13}C NMR of 1-nitro-4-(phenoxymethyl)benzene (4b)

$CDCl_3$, 125 MHz, 23 °C.

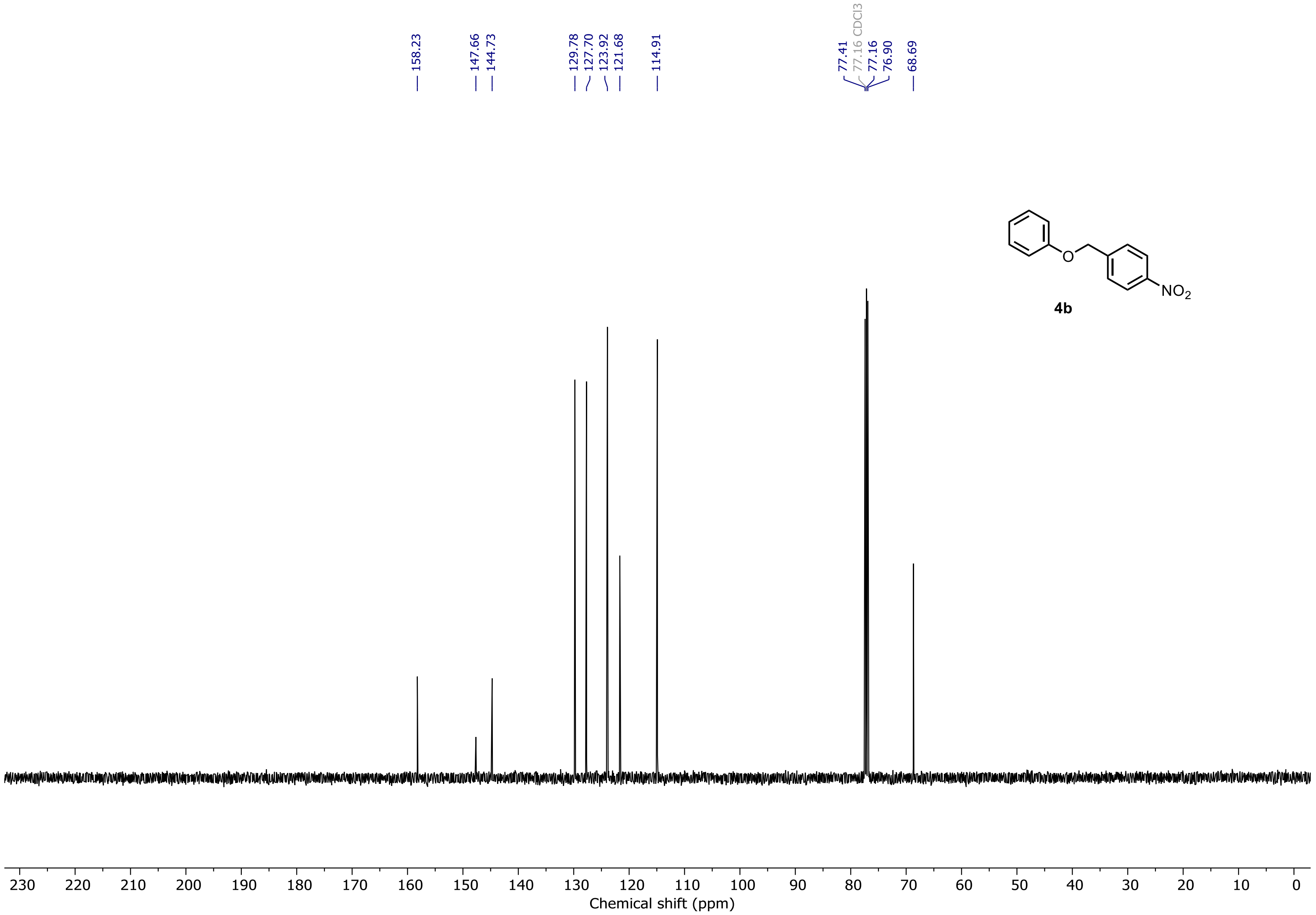

^{1}H NMR of (cyclohexylmethoxy)benzene (4c)

$CDCl_3$, 500 MHz, 23 °C.

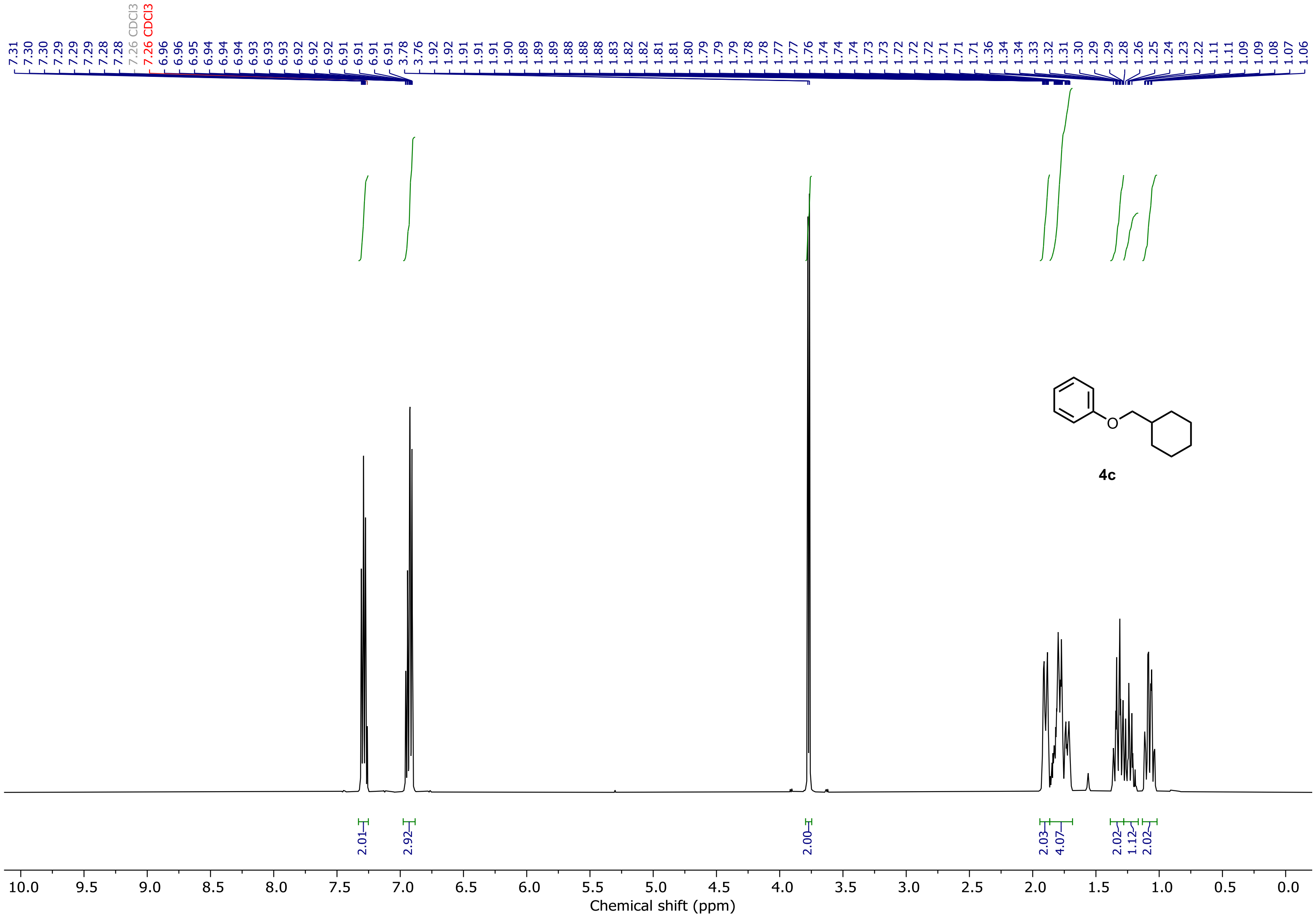

^{13}C NMR of (cyclohexylmethoxy)benzene (4c)

$CDCl_3$, 125 MHz, 23 °C.

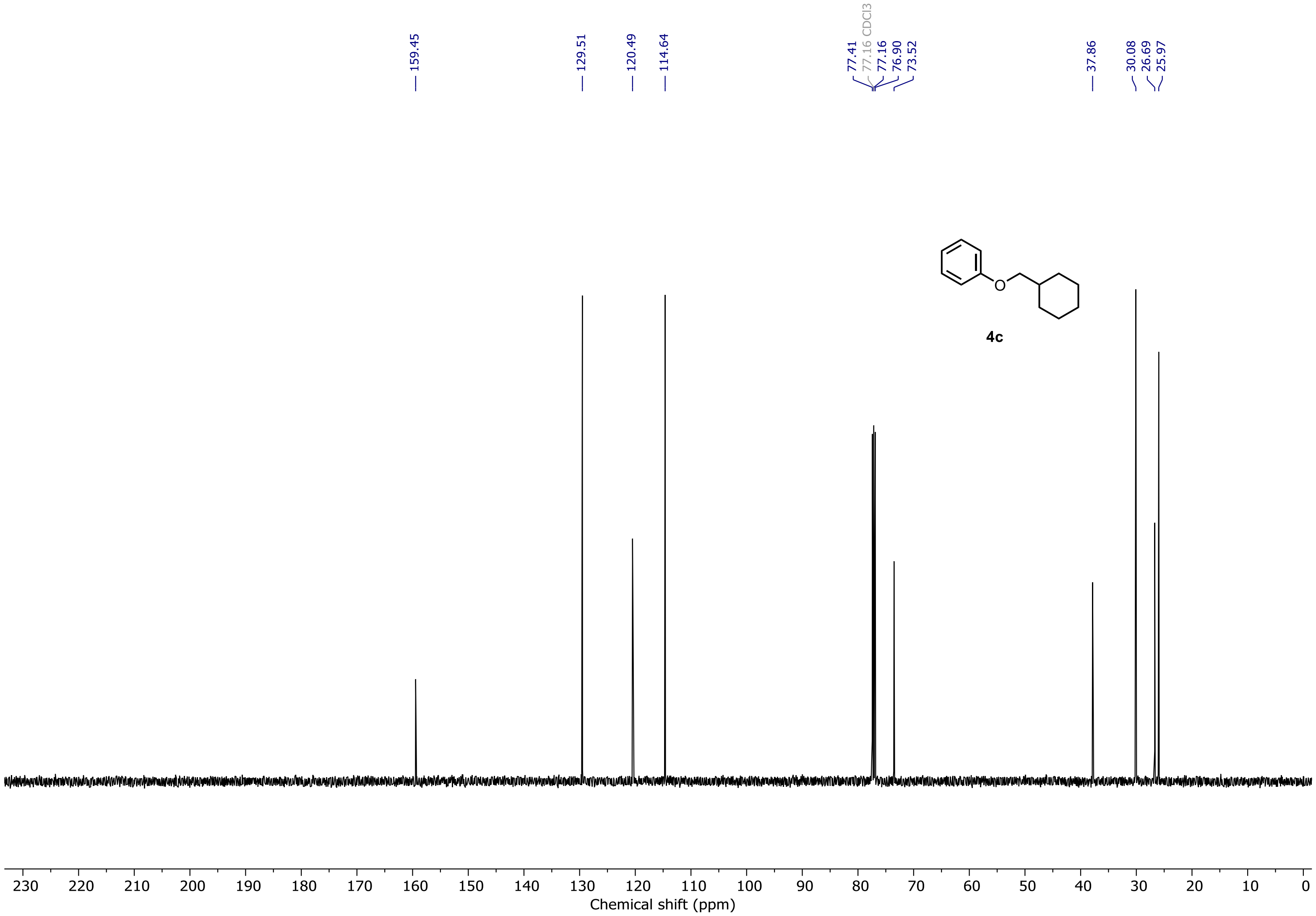

^{1}H NMR of protodecarboxylated Ibuprofen (5a)

$CDCl_3$, 500 MHz, 23 °C.

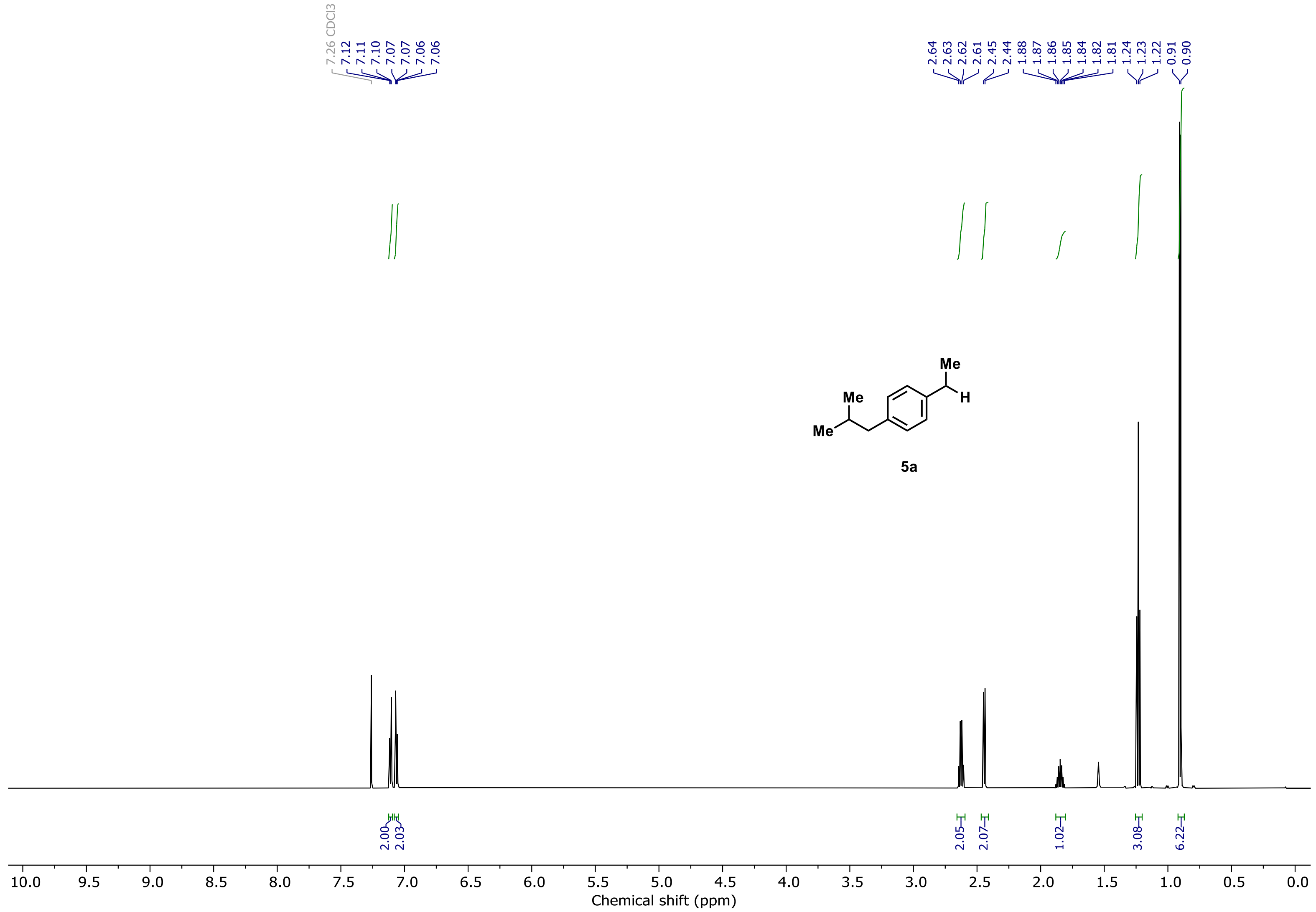

^{13}C NMR of protodecarboxylated Ibuprofen (5a)

$CDCl_3$, 125 MHz, 23 °C.

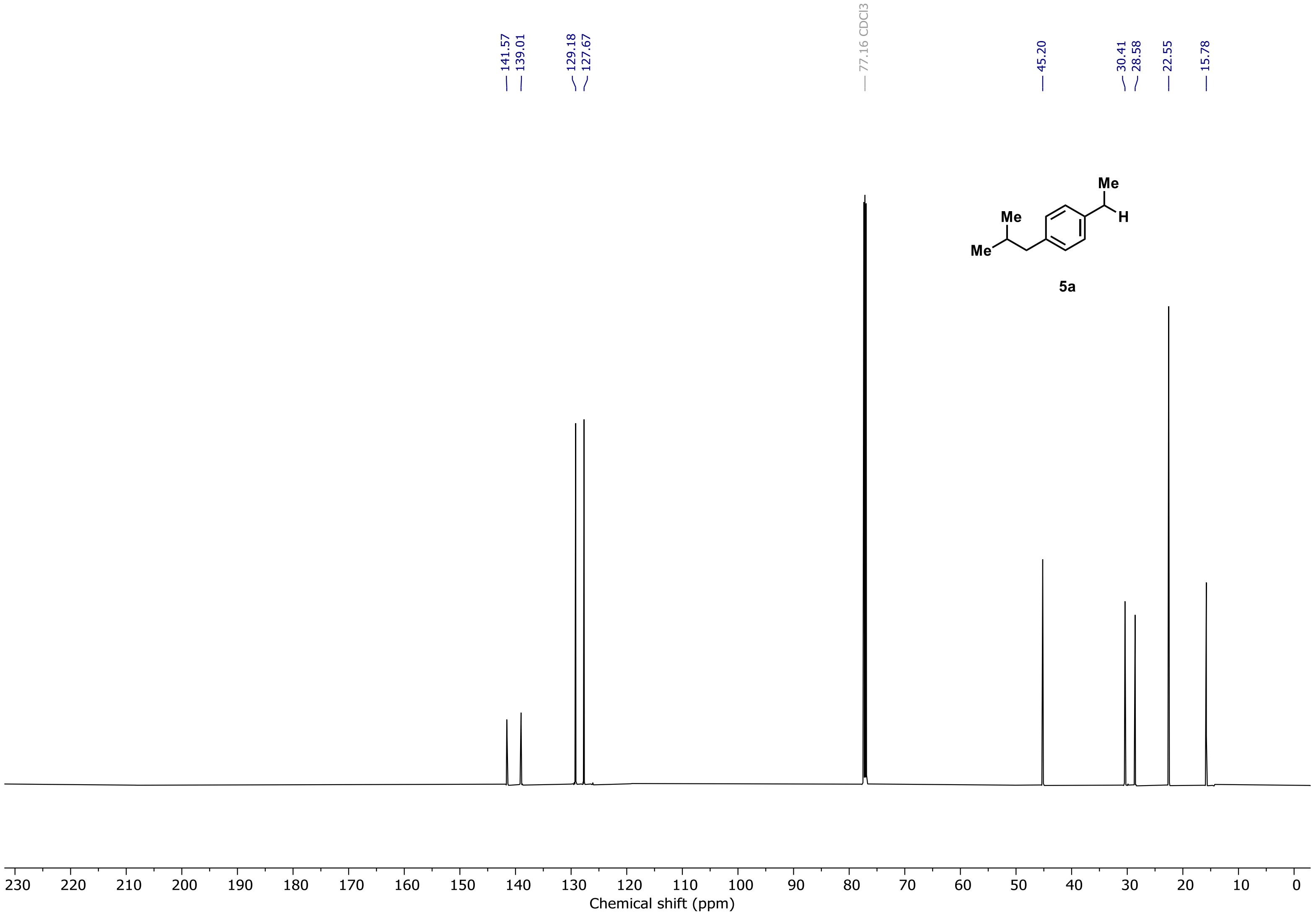

^{1}H NMR of 1-methyl-4-phenylbenzene (5b)

$CDCl_3$, 500 MHz, 23 °C.

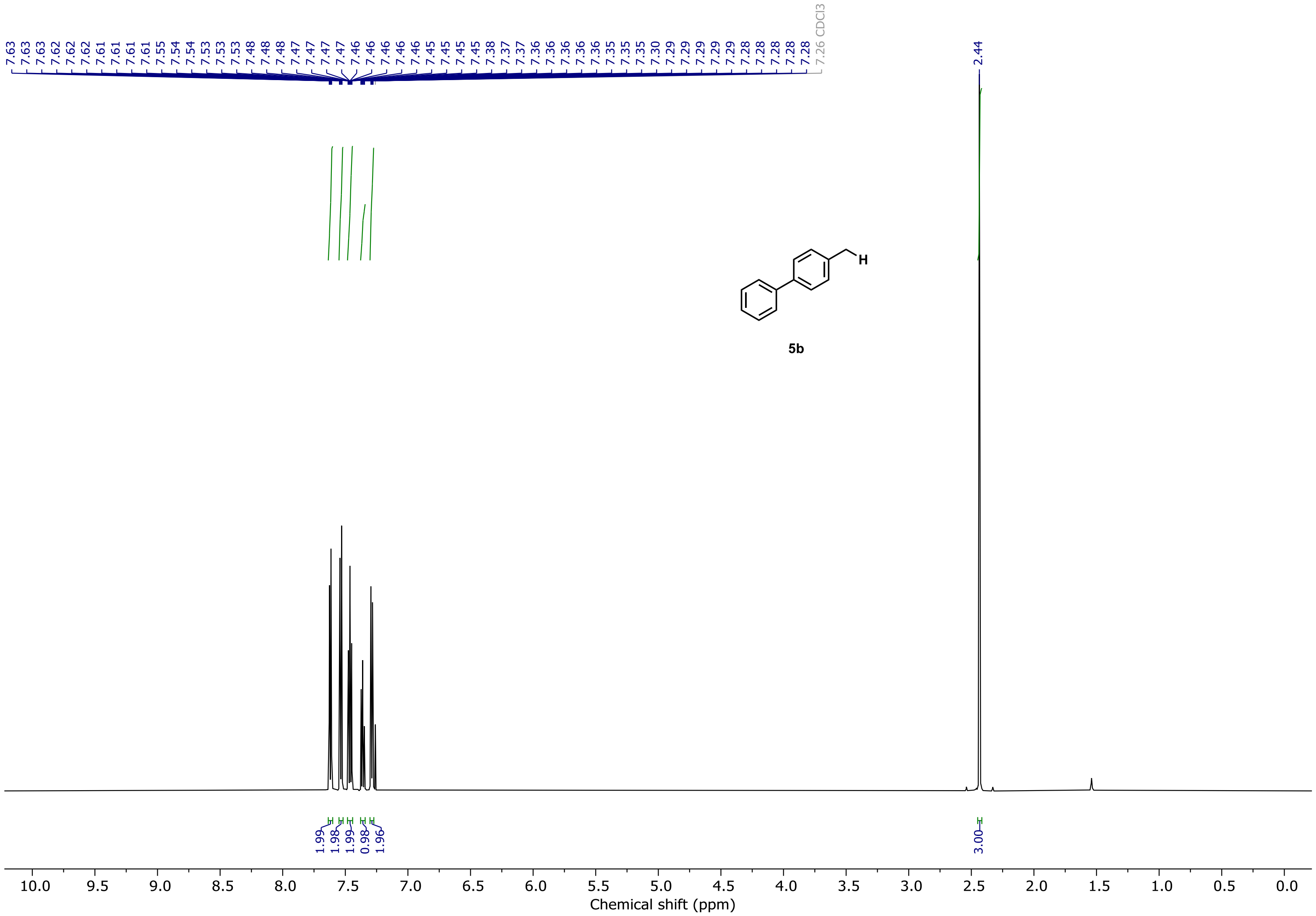

^{13}C NMR of 1-methyl-4-phenylbenzene (5b)

$CDCl_3$, 125 MHz, 23 °C.

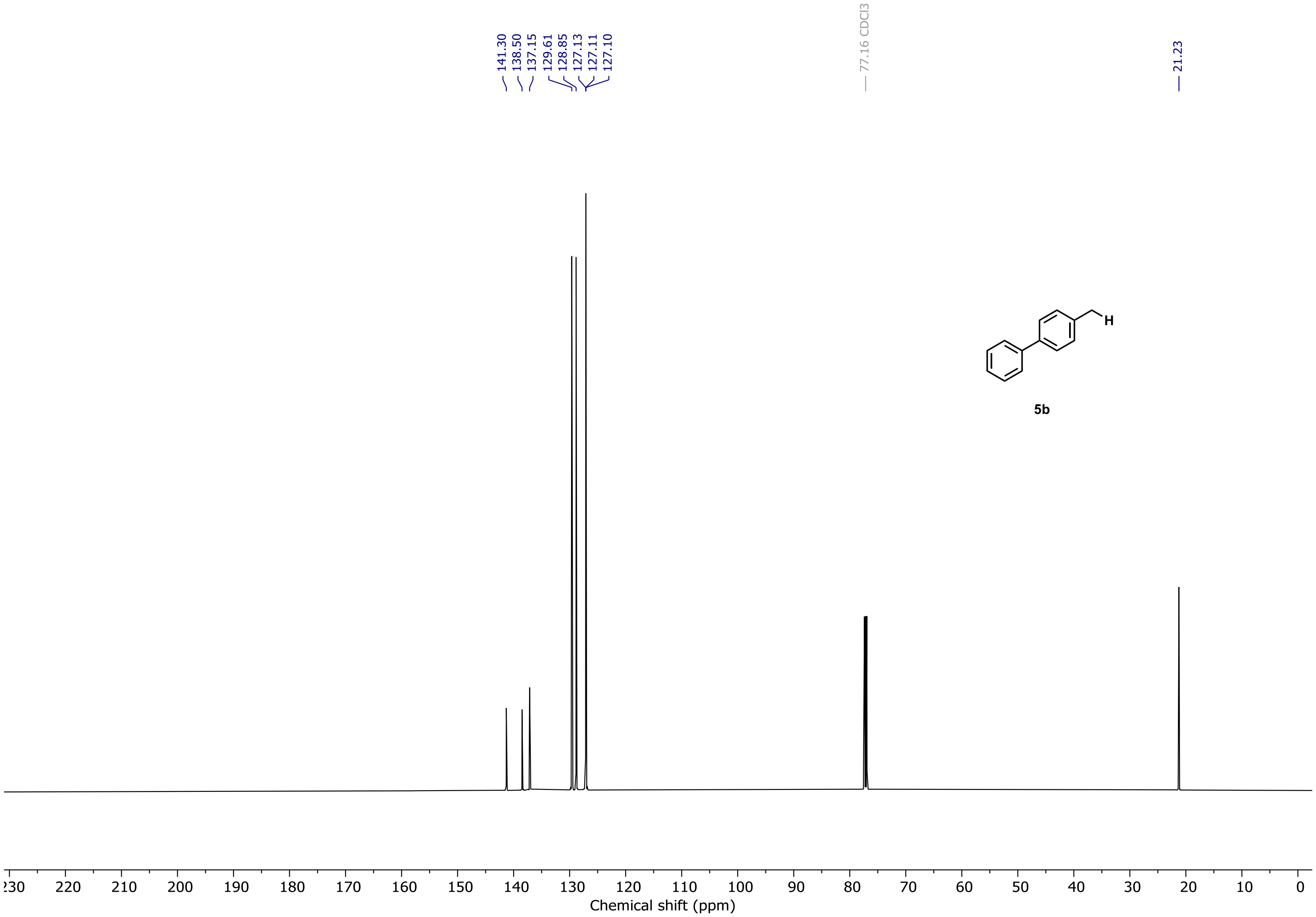

^{1}H NMR of protodecarboxylated phenylmalonic acid (5c)

$CDCl_3$, 500 MHz, 23 °C.

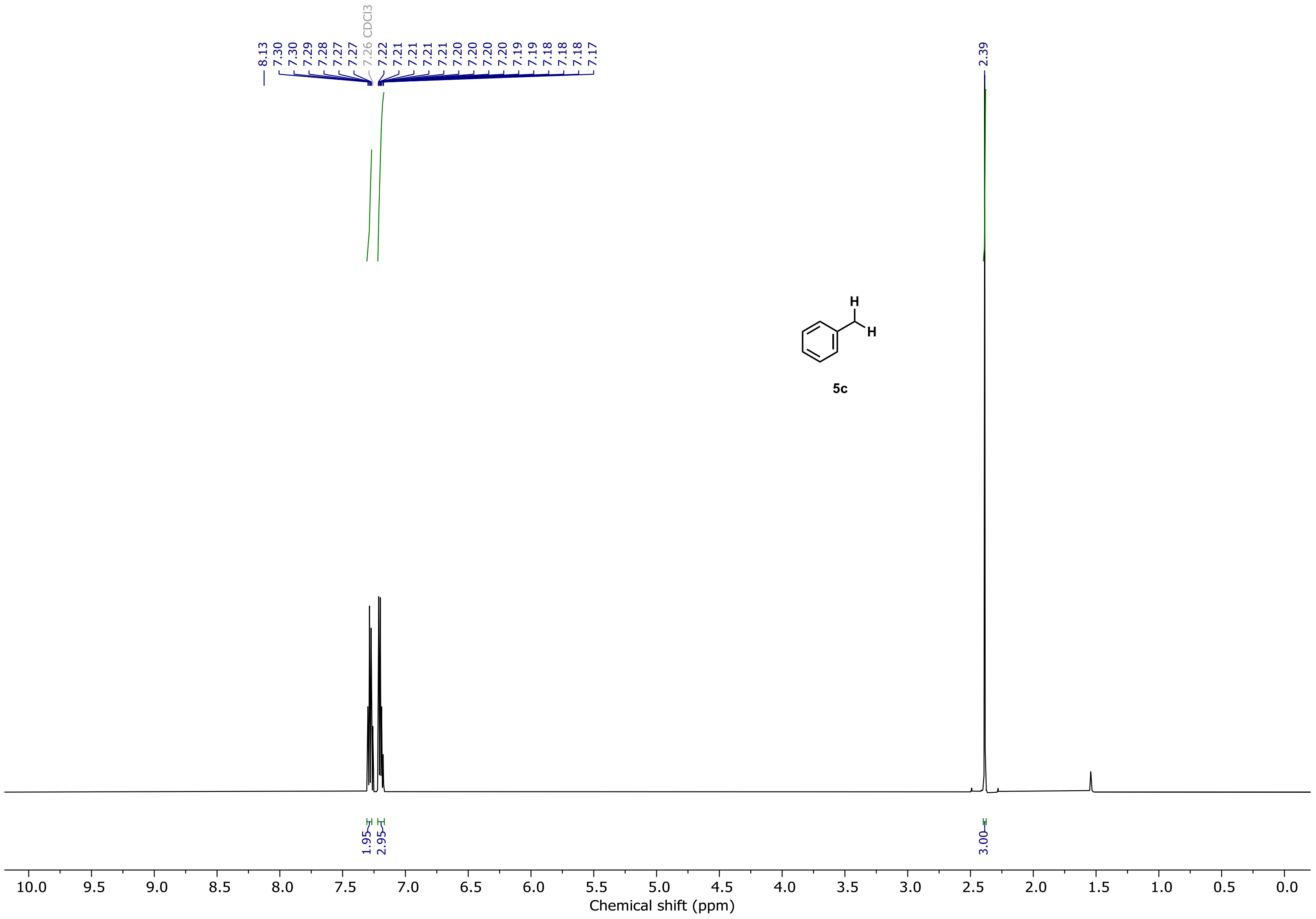

^{13}C NMR of protodecarboxylated phenylmalonic acid (5c)

$CDCl_3$, 125 MHz, 23 °C.

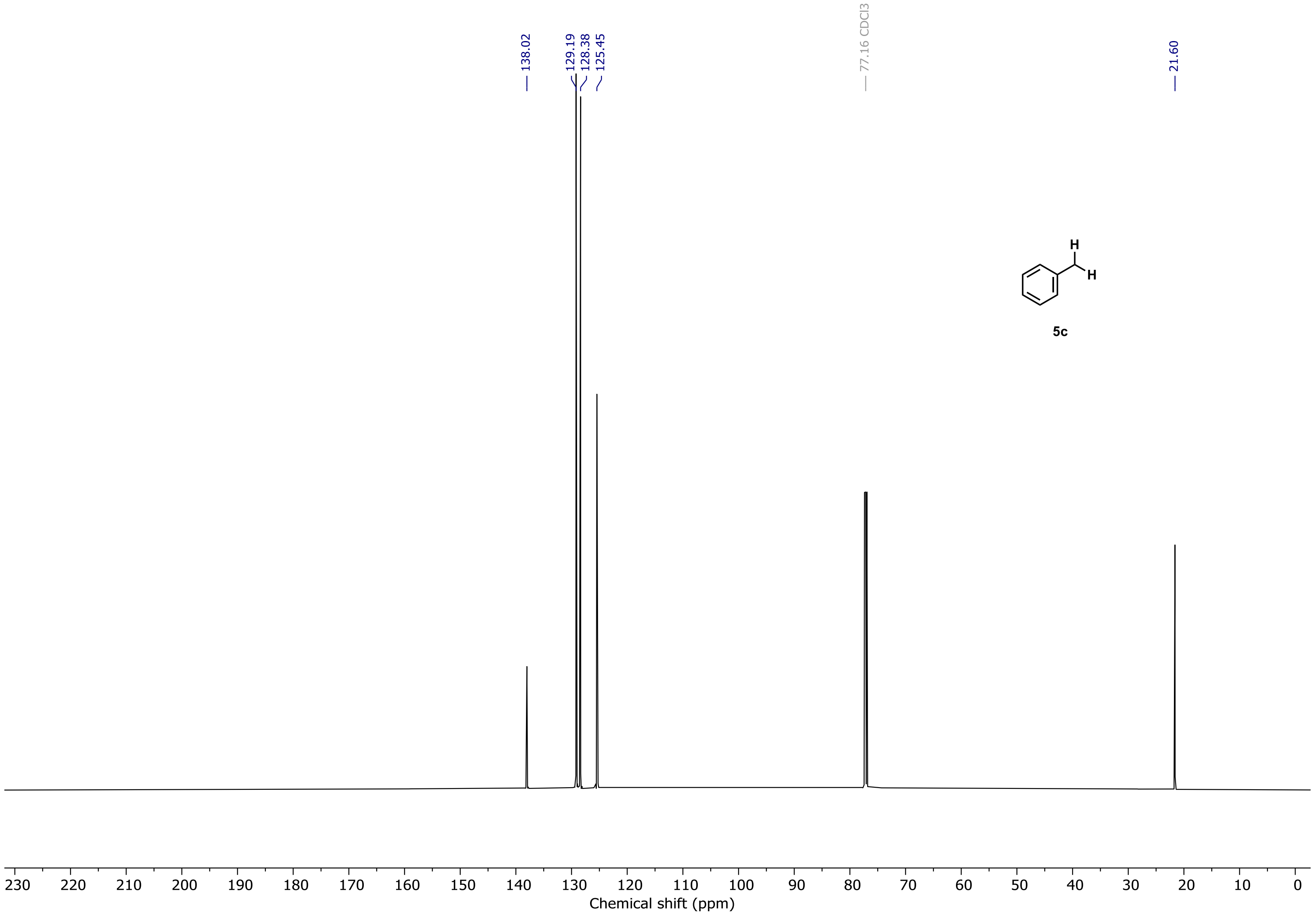

^{1}H NMR of difluoromethylbenzene (5d)

$CDCl_3$, 500, MHz 23 °C.

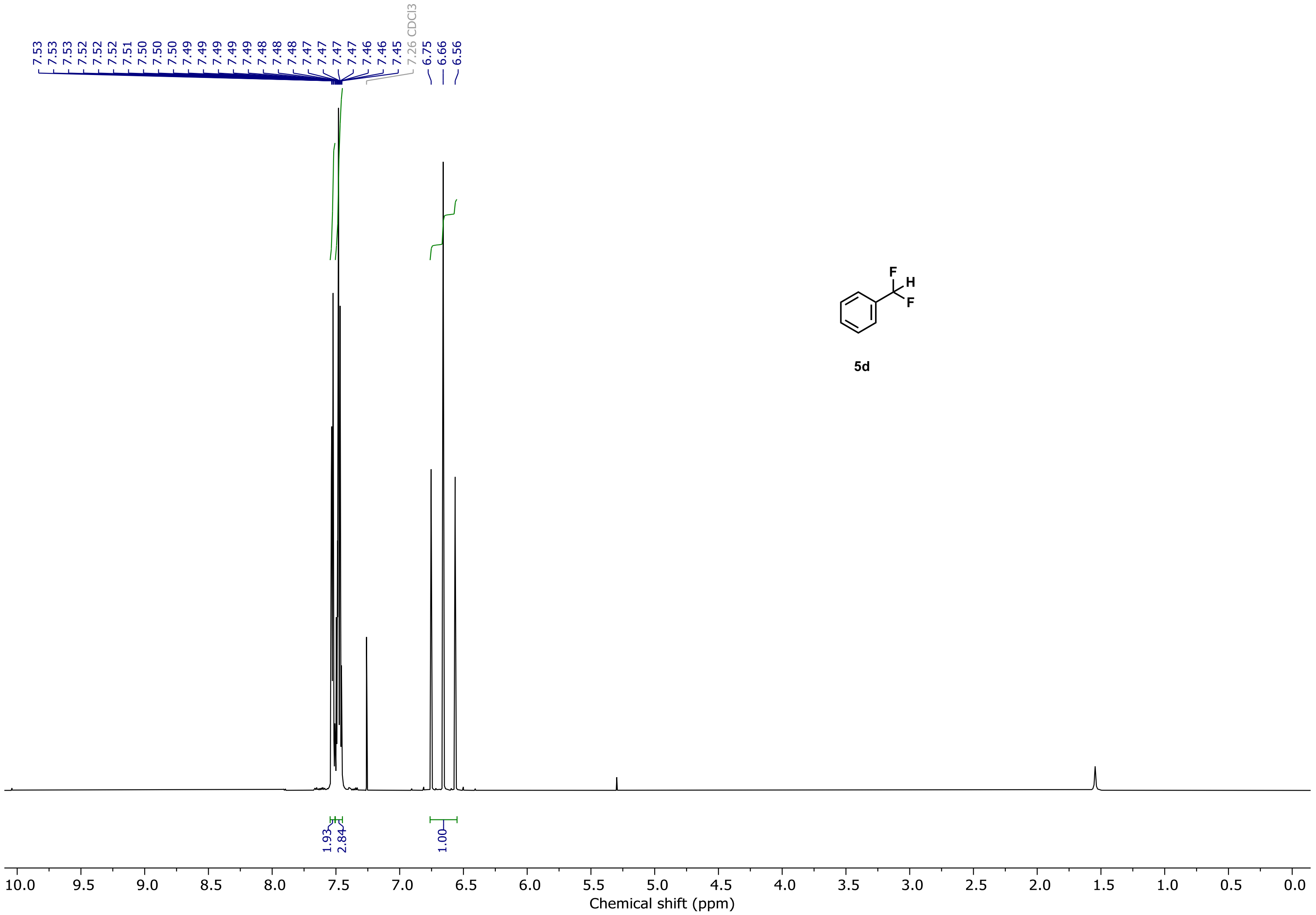

^{13}C NMR of difluoromethylbenzene (5d)

$CDCl_3$, 125 MHz, 23 °C.

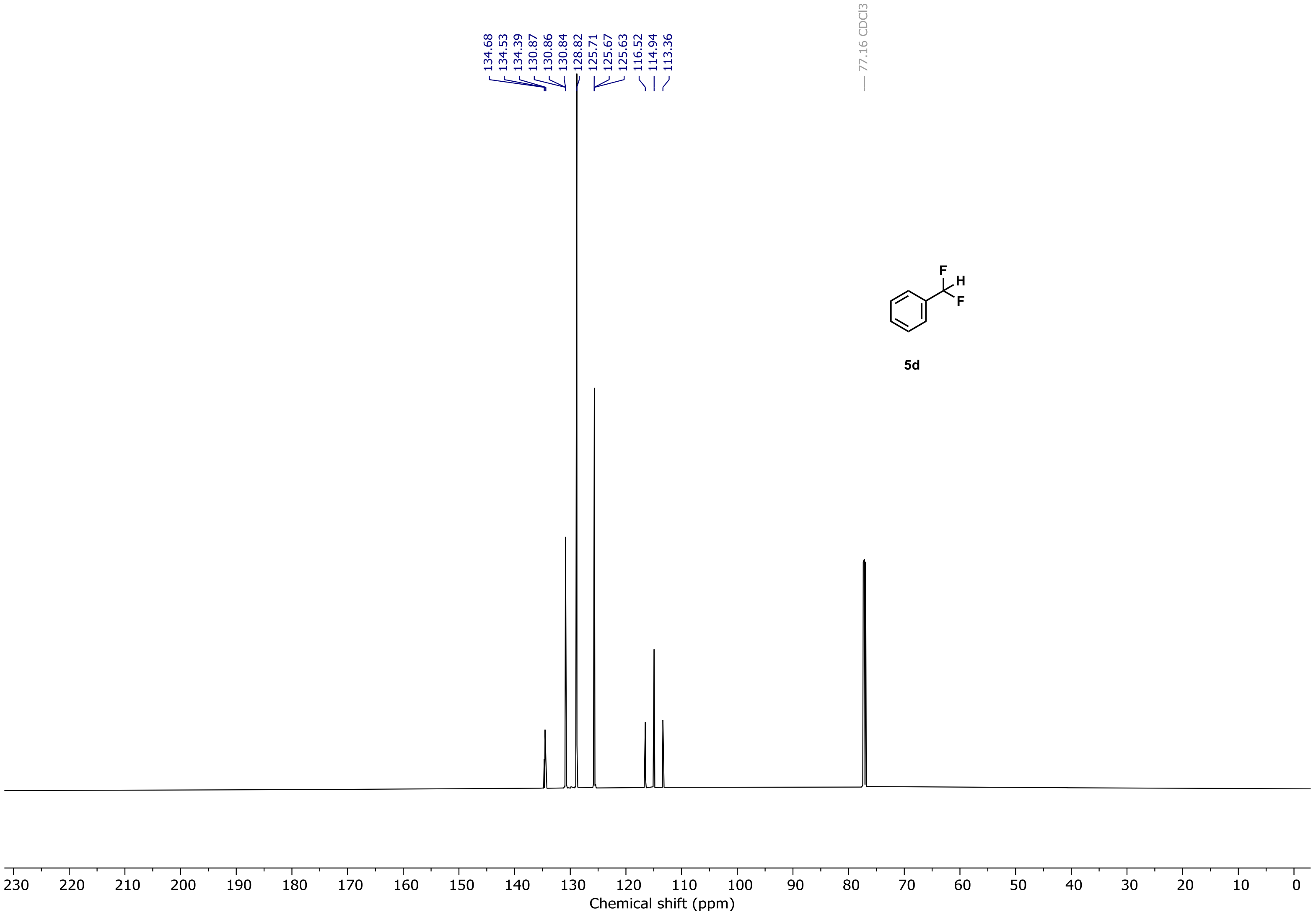

^{19}F NMR of difluoromethylbenzene (5d)

$CDCl_3$, 470 MHz, 23 °C.

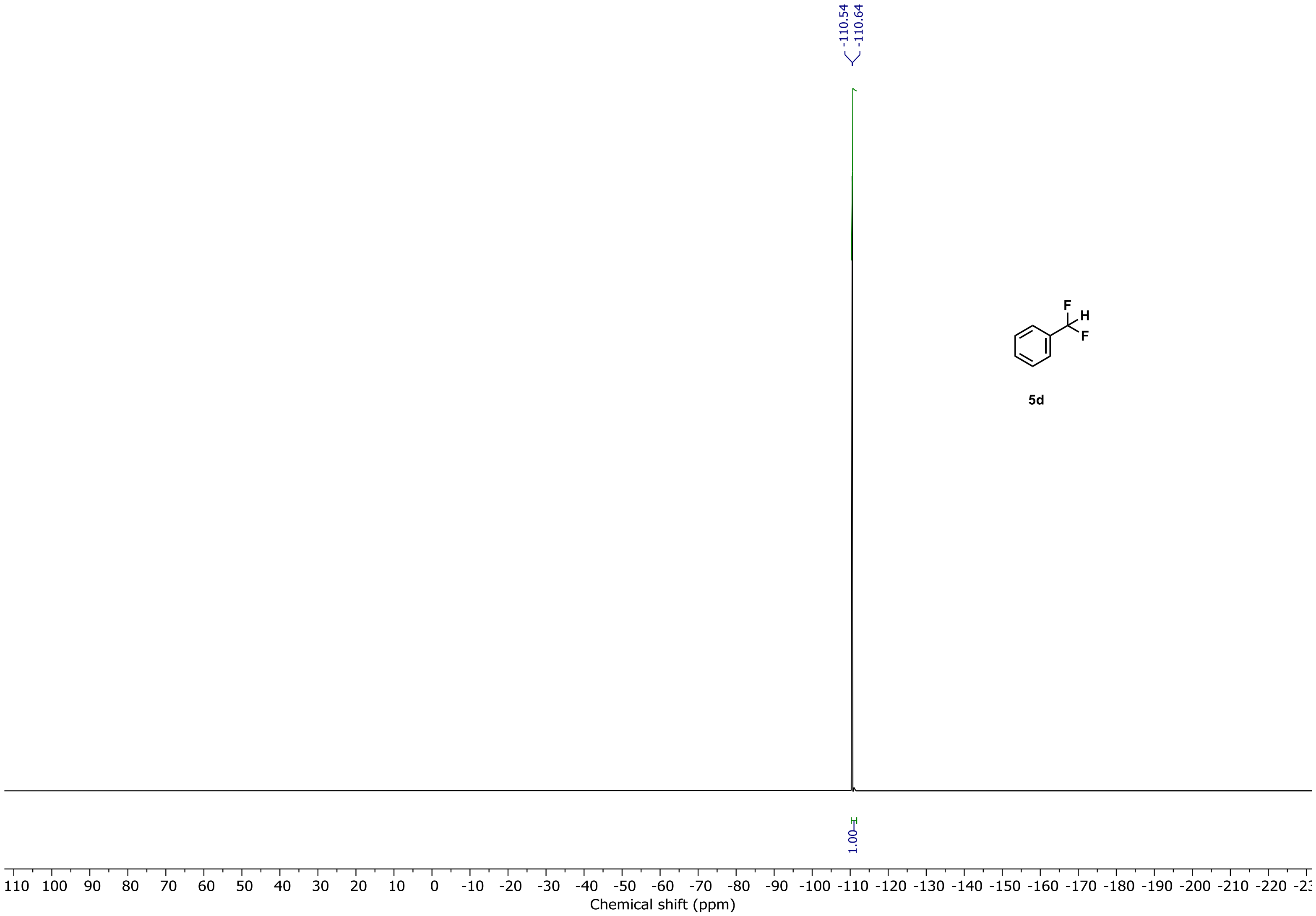

5d

^{1}H NMR of 1,1-diphenylmethane (5e)

$CDCl_3$, 500 MHz, 23 °C.

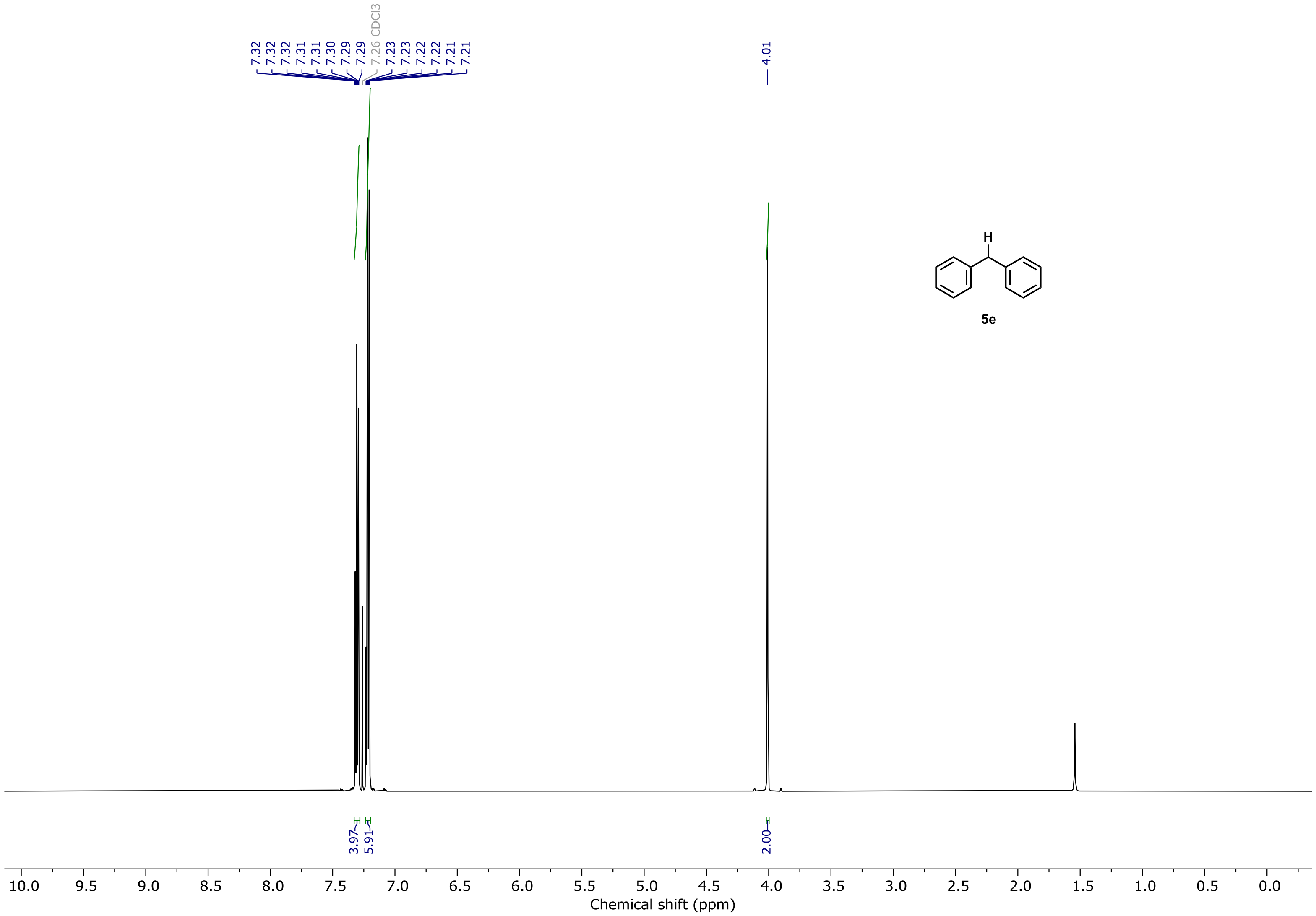

^{13}C NMR of 1,1-diphenylmethane (5e)

$CDCl_3$, 125 MHz, 23 °C.

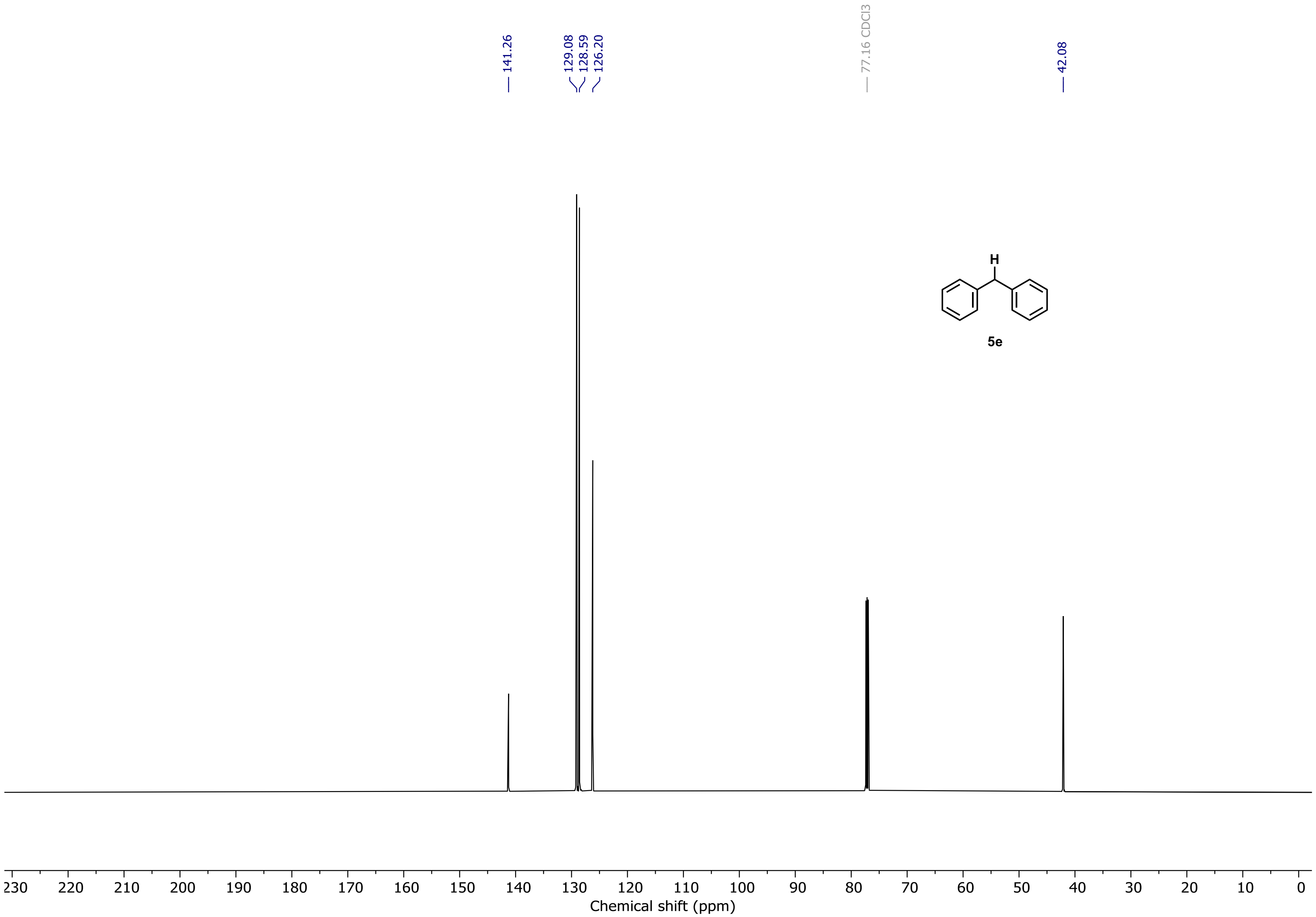

^{1}H NMR of 1,3-dimethylbenzene (5f)

$CDCl_3$, 500 MHz, 23 °C.

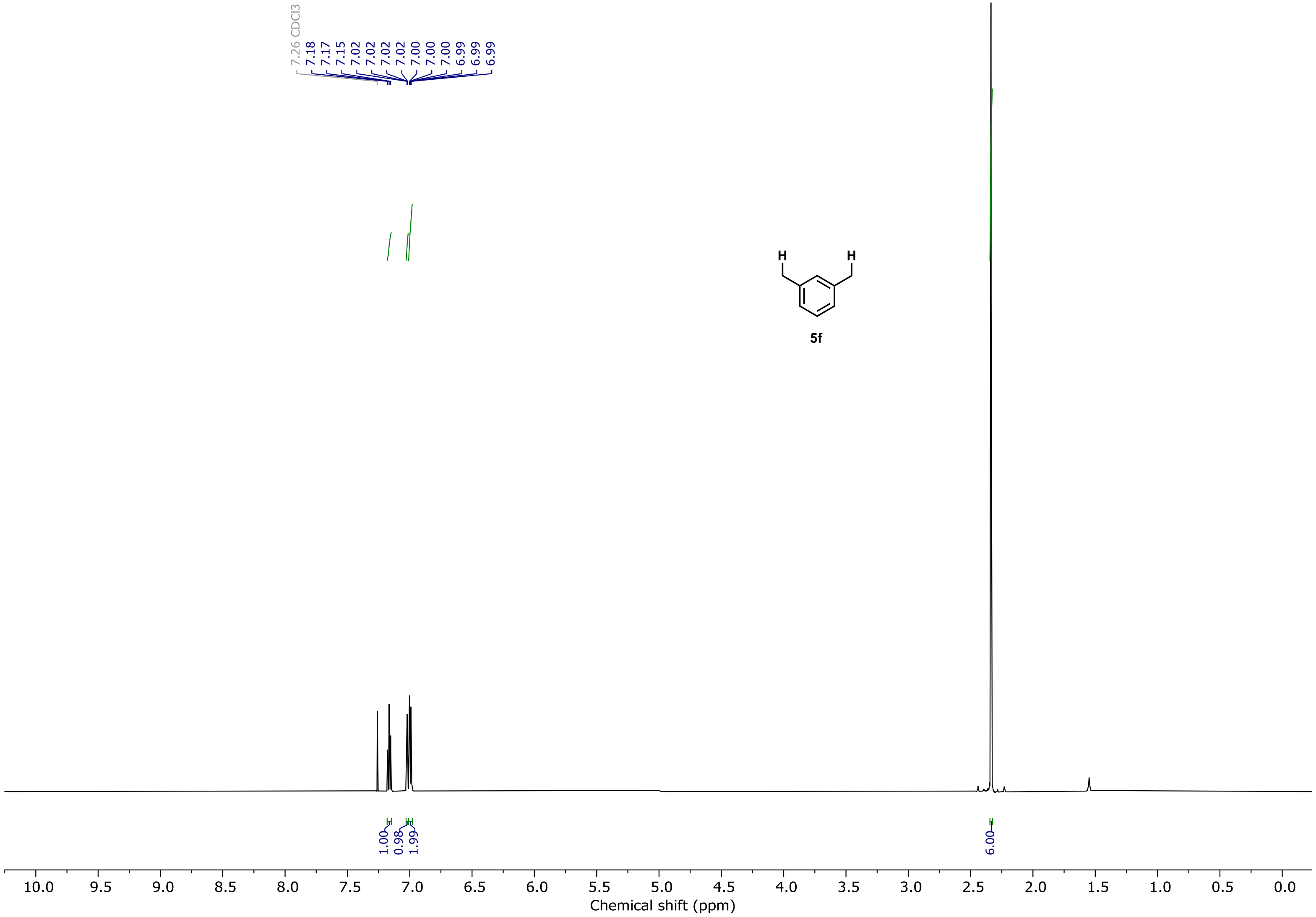

^{13}C NMR of 1,3-dimethylbenzene (5f)

$CDCl_3$, 125 MHz, 23 °C.

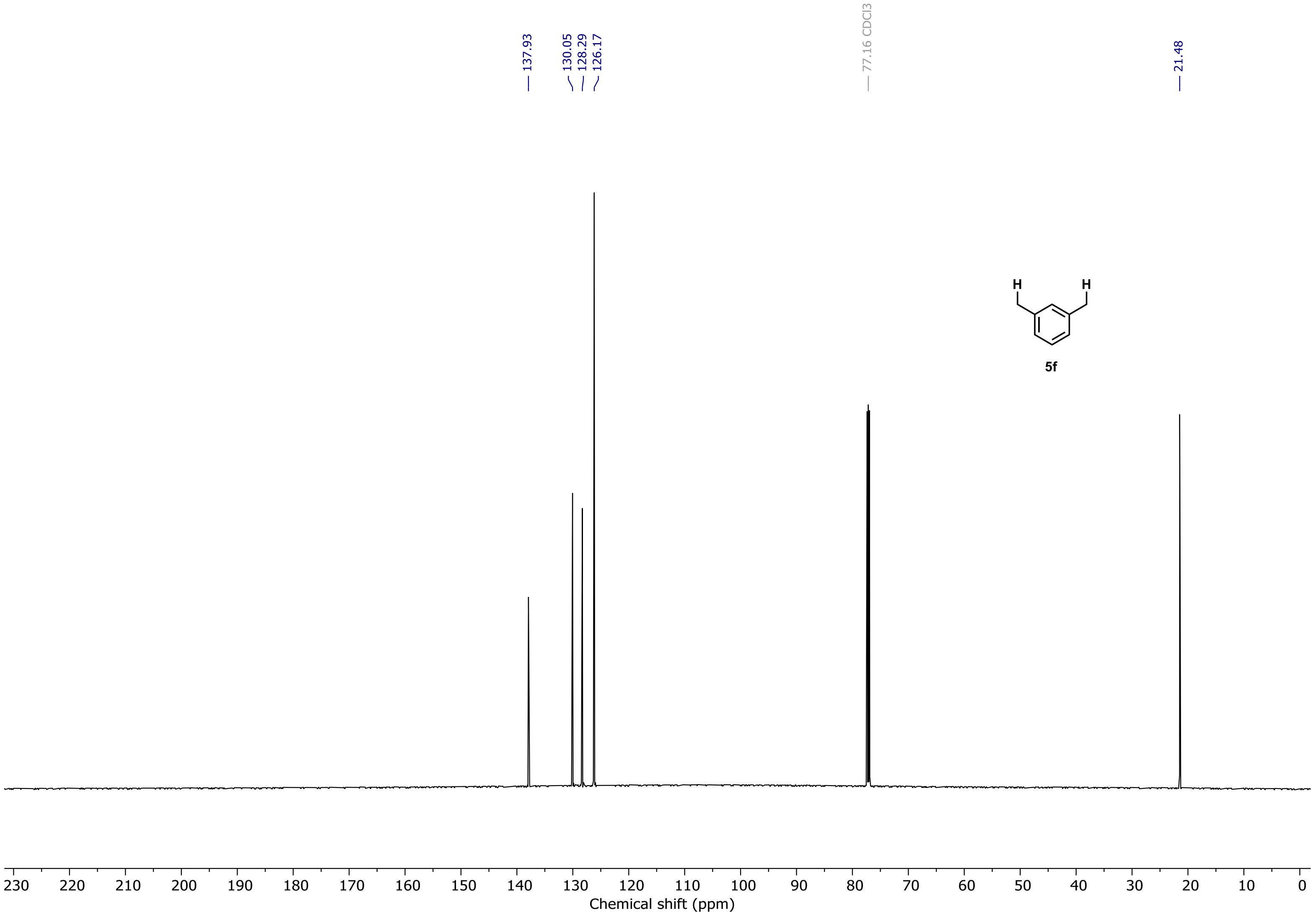

REFERENCES

1. Carbone, A.; Lucas, C. L.; Moody, C. J., Biomimetic synthesis of the apoptosis-inducing thiazinoquinone thiaplidiaquinone A. *J. Org. Chem.* **2012**, *77* (20), 9179–89.
2. Kang, Q. K.; Lin, Y.; Li, Y.; Xu, L.; Li, K.; Shi, H., Catalytic S_NAr hydroxylation and alkoxylation of aryl fluorides. *Angew. Chem. Int. Ed.* **2021**, *60* (37), 20391–20399.
3 Therrien, B.; Thomas, R. W.; Pilkington, M.; Hoffmann, C.; Gilardoni, F.; Weber, J., Synthesis and reactivity of tethered η^1:η^6-(phosphinoarene)ruthenium dichlorides. *Organometallics* **1998**, *17*, 330–337.
4. Hulla, M.; Ortiz, D.; Katsyuba, S.; Vasilyev, D.; Dyson, P. J., Delineation of the critical parameters of salt catalysts in the N-formylation of amines with CO_2. *Chemistry* **2019**, *25* (47), 11074–11079.
5. Bennett, M. A.; Matheson, T. W., A simple preparation of bis-arene-ruthenium cationic complexes, including those containing different arenes. *J. Organomet. Chem.* **1979**, *175* (1), 87–93.
6. Thodarson, P., Determining association constants from titration experiments in supramolecular chemistry. *Chem. Soc. Rev.* **2011**, *40*, 1305−1323.
7. Albertin, G.; Antoniutti, S.; Castro, J.; Gasparetto, G., Pentamethylcyclopentadienyl half-sandwich hydrazine complexes of ruthenium: preparation and reactivity. *New. J. Chem.* **2019**, *43* (6), 2676–2686.
8. Neese, F., Software update: the ORCA program system, version 4.0. *WIREs Comp. Mol. Sci.* **2018**, *8* (1), e1327.
9. Adamo, C.; Barone, V., Toward reliable density functional methods without adjustable parameters: The PBE0 model. *J. Chem. Phys.* **1999**, *110* (13), 6158–6170.
10. Weigend, F.; Ahlrichs, R., Balanced basis sets of split valence, triple zeta valence and quadruple zeta valence quality for H to Rn: Design and assessment of accuracy. *Phys. Chem. Chem. Phys.* **2005,** *7* (18), 3297–3305.
11. Becke, A. D., Density-functional exchange-energy approximation with correct asymptotic behavior. *Phys. Rev. A* **1988**, *38* (6), 3098.
12. Perdew, J. P., Density-functional approximation for the correlation energy of the inhomogeneous electron gas. *Phys. Rev. B* **1986**, *33* (12), 8822.
13. van Lenthe, E.; Baerends, E.-J.; Snijders, J. G., Relativistic regular two‐component Hamiltonians. *J. Chem. Phys.* **1993**, *99* (6), 4597–4610.
14. van Lenthe, E.; Baerends, E.-J.; Snijders, J. G., Relativistic total energy using regular approximations. *J. Chem. Phys.* **1994**, *101* (11), 9783–9792.
15. Van Lenthe, E.; Van Leeuwen, R.; Baerends, E.; Snijders, J., Relativistic regular two‐component Hamiltonians. *Int. J. Quantum Chem.* **1996**, *57* (3), 281–293.
16. Pantazis, D. A.; Chen, X.-Y.; Landis, C. R.; Neese, F., All-electron scalar relativistic basis sets for third-row transition metal atoms. *J. Chem. Theory Comput.* **2008**, *4* (6), 908–919.
17. Lu, T.; Chen, F., Multiwfn: A multifunctional wavefunction analyzer. *J. Comput. Chem.* **2012**, *33* (5), 580-592.
18. Neese, F., Software update: The ORCA program system—Version 5.0. *WIREs Comp. Mol. Sci.* **2022**, *12* (5), e1606.
19. Grimme, S.; Antony, J.; Ehrlich, S.; Krieg, H., A consistent and accurate ab initio parametrization of density functional dispersion correction (DFT-D) for the 94 elements H-Pu. *J. Chem. Phys.* **2010**, *132* (15), 154104.
20. Grimme, S.; Ehrlich, S.; Goerigk, L., Effect of the damping function in dispersion corrected density functional theory. *J. Comput. Chem.* **2011**, *32* (7), 1456–1465.
21. Rolfes, J. D.; Neese, F.; Pantazis, D. A., All‐electron scalar relativistic basis sets for the elements Rb–Xe. *J. Comput. Chem.* **2020**, *41* (20), 1842–1849.
22. Zheng, J.; Xu, X.; Truhlar, D. G., Minimally augmented Karlsruhe basis sets. *Theor. Chem. Acc.* **2011**, *128*, 295–305.
23. Weigend, F., Accurate Coulomb-fitting basis sets for H to Rn. *Phys. Chem. Chem. Phys.* **2006**, *8* (9), 1057–1065.
24. Stoychev, G. L.; Auer, A. A.; Neese, F., Automatic Generation of Auxiliary Basis Sets. *J. Chem. Theory Comput.* **2017**, *13* (2), 554–562.
25. Barone, V.; Cossi, M., Quantum calculation of molecular energies and energy gradients in solution by a conductor solvent model. *J. Phys. Chem. A* **1998**, *102* (11), 1995–2001.
26. Neese, F.; Wennmohs, F.; Hansen, A.; Becker, U., Efficient, approximate and parallel Hartree–Fock and hybrid DFT calculations. A 'chain-of-spheres' algorithm for the Hartree–Fock exchange. *Chem. Phys.* **2009**, *356* (1), 98–109.
27. Izsák, R.; Neese, F., An overlap fitted chain of spheres exchange method. *J. Chem. Phys.* **2011**, *135* (14), 144105.
28. Izsák, R.; Neese, F.; Klopper, W., Robust fitting techniques in the chain of spheres approximation to the Fock exchange: The role of the complementary space. *J. Chem. Phys.* **2013**, *139* (9), 094111.
29. Helmich-Paris, B.; de Souza, B.; Neese, F.; Izsák, R., An improved chain of spheres for exchange algorithm. *J.*

Chem. Phys. **2021**, *155* (10), 104109.
30. Bannwarth, C.; Ehlert, S.; Grimme, S., GFN2-xTB—An Accurate and Broadly Parametrized Self-Consistent Tight-Binding Quantum Chemical Method with Multipole Electrostatics and Density-Dependent Dispersion Contributions. *J. Chem. Theory Comput.* **2019**, *15* (3), 1652–1671.
31. Guo, Y.; Riplinger, C.; Becker, U.; Liakos, D. G.; Minenkov, Y.; Cavallo, L.; Neese, F., Communication: An improved linear scaling perturbative triples correction for the domain based local pair-natural orbital based singles and doubles coupled cluster method [DLPNO-CCSD(T)]. *J. Chem. Phys.* **2018**, *148* (1), 011101.
32. Riplinger, C.; Sandhoefer, B.; Hansen, A.; Neese, F., Natural triple excitations in local coupled cluster calculations with pair natural orbitals. *J. Chem. Phys.* **2013**, *139* (13), 134101.
33. Riplinger, C.; Pinski, P.; Becker, U.; Valeev, E. F.; Neese, F., Sparse maps—A systematic infrastructure for reduced-scaling electronic structure methods. II. Linear scaling domain based pair natural orbital coupled cluster theory. *J. Chem. Phys.* **2016**, *144* (2), 024109.
34. Franzke, Y. J.; Spiske, L.; Pollak, P.; Weigend, F., Segmented Contracted Error-Consistent Basis Sets of Quadruple-ζ Valence Quality for One- and Two-Component Relativistic All-Electron Calculations. *J. Chem. Theory Comput.* **2020**, *16* (9), 5658–5674.
35. Papajak, E.; Truhlar, D. G., Convergent Partially Augmented Basis Sets for Post-Hartree−Fock Calculations of Molecular Properties and Reaction Barrier Heights. *J. Chem. Theory Comput.* **2011**, *7* (1), 10–18.
36. Weigend, F.; Köhn, A.; Hättig, C., Efficient use of the correlation consistent basis sets in resolution of the identity MP2 calculations. *J. Chem. Phys.* **2002**, *116* (8), 3175–3183.
37. Li, Z.; Xiao, Y.; Liu, W., On the spin separation of algebraic two-component relativistic Hamiltonians: Molecular properties. *J. Chem. Phys.* **2014**, *141* (5).
38. Liu, W.; Peng, D., Exact two-component Hamiltonians revisited. *J. Chem. Phys.* **2009**, *131* (3), 031104.
39. Li, Z.; Xiao, Y.; Liu, W., On the spin separation of algebraic two-component relativistic Hamiltonians. *J. Chem. Phys.* **2012**, *137* (15), 154114.
40. Kutzelnigg, W.; Liu, W., Quasirelativistic theory equivalent to fully relativistic theory. *J. Chem. Phys.* **2005**, *123* (24), 241102.
41. Marenich, A. V.; Cramer, C. J.; Truhlar, D. G., Universal solvation model based on solute electron density and on a continuum model of the solvent defined by the bulk dielectric constant and atomic surface tensions. *J. Phys. Chem. B* **2009**, *113* (18), 6378–6396.
42. Glendening, E. D.; Landis, C. R.; Weinhold, F. *Wiley Interdiscip. Rev. Comput. Mol. Sci.* **2012**, *2*, 1–42.
43. Badenhoop, J. K.; Weinhold, F., Natural bond orbital analysis of steric interactions. *J. Chem. Phys.*, **1997**, *107*(14), 5406–5421.
44. Badenhoop, J. K.; Weinhold, F., Natural steric analysis: Ab initio van der Waals radii of atoms and ions. *J. Chem. Phys.*, **1997**, *107*(14), 5422–5432.
45. Badenhoop, J. K.; Weinhold, F., Natural steric analysis of internal rotation barriers. *Int. J. Quantum Chem.*, **1999**, *72*(4), 269–280.
46. *NBO 7.0*; Theoretical Chemistry Institute, University of Wisconsin: Madison, WI, 2018. https://nbo6.chem.wisc.edu/ (accessed 2024-04-05).
47. Schneider, W. B.; Bistoni, G.; Sparta, M.; Saitow, M.; Riplinger, C.; Auer, A. A.; Neese, F. Decomposition of Intermolecular Interaction Energies within the Local Pair Natural Orbital Coupled Cluster Framework. *J. Chem. Theory Comput.* **2016**, *12* (10), 4778–4792.
48. Altun, A.; Saitow, M.; Neese, F.; Bistoni, G. Local Energy Decomposition of Open-Shell Molecular Systems in the Domain-Based Local Pair Natural Orbital Coupled Cluster Framework. *J. Chem. Theory Comput.* **2019**, *15* (3), 1616–1632.
49. Bistoni, G. Finding Chemical Concepts in the Hilbert Space: Coupled Cluster Analyses of Noncovalent Interactions. *WIREs Comput. Mol. Sci.* **2020**, *10* (3), e1442.
50. Bistoni, G.; Altun, A.; Wang, Z.; Neese, F. Local Energy Decomposition Analysis of London Dispersion Effects: From Simple Model Dimers to Complex Biomolecular Assemblies. *Acc. Chem. Res.* **2024**, accepted, DOI: 10.1021/acs.accounts.4c00085
